# Bone Marrow-Infiltrating Human Neuroblastoma Cells Express High Levels of Calprotectin and HLA-G Proteins

**DOI:** 10.1371/journal.pone.0029922

**Published:** 2012-01-09

**Authors:** Fabio Morandi, Paola Scaruffi, Fabio Gallo, Sara Stigliani, Stefano Moretti, Stefano Bonassi, Claudio Gambini, Katia Mazzocco, Paolo Fardin, Riccardo Haupt, Giampaolo Arcamone, on behalf of the Italian Cooperative Group for Neuroblastoma, Vito Pistoia, Gian Paolo Tonini, Maria Valeria Corrias

**Affiliations:** 1 Laboratory of Oncology, Gaslini Institute, Genoa, Italy; 2 Translational Oncopathology, National Cancer Research Institute, Genoa, Italy; 3 Molecular Epidemiology, National Cancer Research Institute, Genoa, Italy; 4 LAMSADE-CNRS, Université Paris Dauphine, Paris, France; 5 Clinical and Molecular Epidemiology, IRCCS San Raffaele Pisana, Rome, Italy; 6 Service of Pathology, Scientific Directorate, Gaslini Institute, Genoa, Italy; 7 Laboratory of Molecular Biology, Scientific Directorate, Gaslini Institute, Genoa, Italy; 8 Laboratory of Epidemiology and Biostatistics Section, Scientific Directorate, Gaslini Institute, Genoa, Italy; 9 Policlinico Università Bari, Bari, Italy; University of Medicine and Dentistry of New Jersey, United States of America

## Abstract

Metastases in the bone marrow (BM) are grim prognostic factors in patients with neuroblastoma (NB). In spite of extensive analysis of primary tumor cells from high- and low-risk NB patients, a characterization of freshly isolated BM-infiltrating metastatic NB cells is still lacking. Our aim was to identify proteins specifically expressed by metastatic NB cells, that may be relevant for prognostic and therapeutic purposes. Sixty-six Italian children over 18 months of age, diagnosed with stage 4 NB, were included in the study. Metastatic NB cells were freshly isolated from patients' BM by positive immunomagnetic bead manipulation using anti-GD2 monoclonal antibody. Gene expression profiles were compared with those obtained from archived NB primary tumors from patients with 5y-follow-up. After validation by RT-qPCR, expression/secretion of the proteins encoded by the up-regulated genes in the BM-infiltrating NB cells was evaluated by flow cytometry and ELISA. Compared to primary tumor cells, BM-infiltrating NB cells down-modulated the expression of *CX3CL1*, *AGT*, *ATP1A2* mRNAs, whereas they up-regulated several genes commonly expressed by various lineages of BM resident cells. BM-infiltrating NB cells expressed indeed the proteins encoded by the top-ranked genes, *S100A8* and *A9* (calprotectin), *CD177* and *CD3*, and secreted the CXCL7 chemokine. BM-infiltrating NB cells also expressed CD271 and HLA-G. We have identified proteins specifically expressed by BM-infiltrating NB cells. Among them, calprotectin, a potent inflammatory protein, and HLA-G, endowed with tolerogenic properties facilitating tumor escape from host immune response, may represent novel biomarkers and/or targets for therapeutic intervention in high-risk NB patients.

## Introduction

Metastasis is the main cause of death in patients with neuroblastoma (NB) [1]–[3]. In spite of aggressive multimodal therapy, children over 18 months of age, presenting at diagnosis with dissemination of malignant neuroblasts in the bone marrow (BM), have a grim prognosis [1], [2]. To achieve efficient target-based therapies for high risk NB patients, characterization of the genetic and biological features of metastatic cells is therefore required.

In recent years, extensive characterization of primary tumor cells from high-risk NB patients has been performed. DNA abnormalities [4], gene expression profiles [5]–[7], and non-coding RNAs expression [8], [9] have been proposed as sensitive indicators of NB tumor aggressiveness. Recently, a great effort has been performed in order to validate the gene classifiers on independent patient cohorts [7], [10]. However, no information is presently available on freshly isolated BM-infiltrating NB cells, with the exception of few features [11], [12]. After *in vitro* culture of BM samples from patients with metastatic disease, Hansford *et al.* [13] isolated cells endowed with high tumorigenic potential, suggesting that metastatic cells may be enriched in tumor initiating cells (TICs). Recently, a gene expression profiling of TICs has been reported [14]. Culture conditions, however, may greatly change the neoplastic cell features. Based on the above considerations, we investigated the transcriptome of NB metastatic cells in comparison with that of primary tumor cells from patients with stage 4 disease. The proteins selectively overexpressed by the BM-infiltrating NB cells may represent novel prognostic markers and potential targets for biologically driven therapy for high risk NB patients.

## Results

### Characterization of BM-infiltrating GD2 positive cells

**Figure 1:**
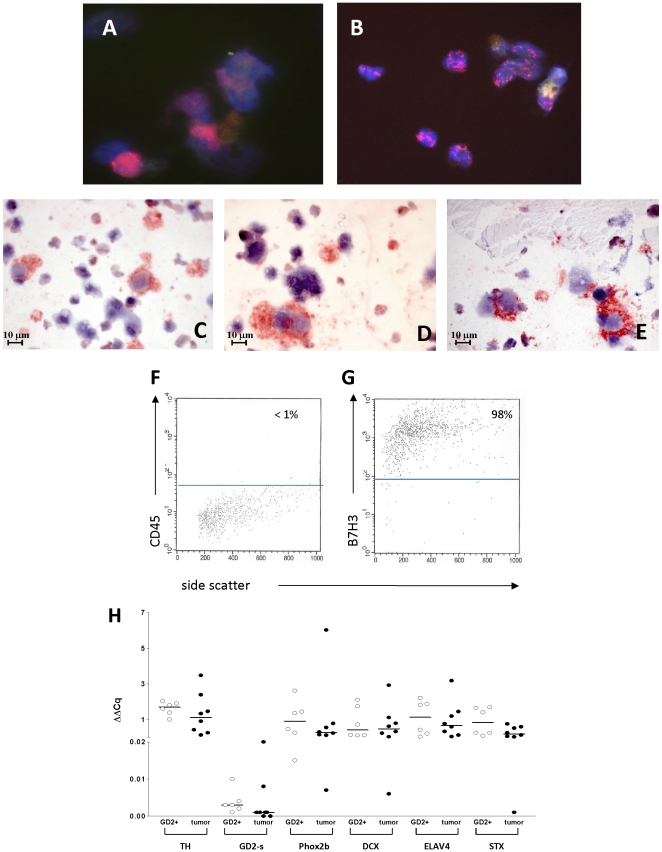
Characteristics of freshly isolated GD2^+^ fractions from stage 4 NB patients. (**A, B**) FISH analysis of two GD2 positive cell preparations from two patients with *MYCN* amplified tumors; (**C, D, E**) Cytospins of a GD2 positive cell preparation tested with anti-GD2 mAb (different from the one used for immunomagnetic bead separation), anti-NB84 mAb, and anti-CD56 mAb, respectively; (**F, G**) cytofluorimetric analysis of a GD2 positive cell preparation stained with anti-CD45 mAb, and anti-B7H3 mAb, respectively. Horizontal bars indicated fluorescence threshold obtained with irrelevant isotype-matched mAbs. Percentage of positive cells are indicated; **H**) RT-qPCR analysis of 6 GD2 positive cell preparations (open circles) and 8 NB primary tumors (closed circles) tested for different NB-specific molecular markers. Horizontal bar indicates the median value of each set.

**Figure 2:**
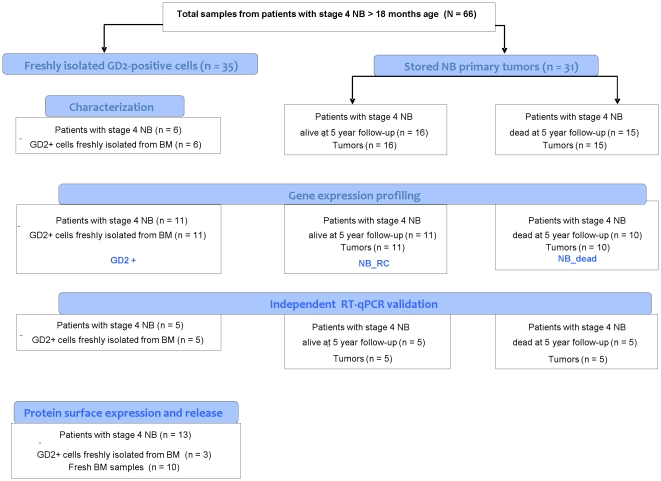
Study design. Freshly isolated GD2 positive cells were obtained by positive immunomagnetic bead manipulation of BM aspirates, as described in M&M section. Primary NB tumors were stored in the Tissue Repository at the Gaslini Institute.

To ascertain whether genes expressed by the GD2 positive fractions can be fully ascribed to the BM-infiltrating NB cells, six freshly isolated GD2 positive cell populations were characterized using different methodologies. As shown in Figure 1AB, FISH analysis performed on GD2 positive cell preparations from two patients with *MYCN* amplification showed presence of *MYCN* amplification. Cytospins of GD2 positive cell preparations were enriched in mononuclear NB cells expressing the NB-specific markers GD2, CD56 [15] and NB84 [16] (Figure 1C, D and E, respectively). Cytofluorimetric analysis showed that less than 5% of the cells recovered by GD2-positive immunomagnetic bead manipulation expressed CD45, whereas more than 95% expressed the B7H3 antigen (Figure 1F and G, respectively). Previous studies showed that B7H3 is not expressed by hematopoietic and stromal BM cells [12], [17]. Finally, all GD2 positive cell preparations expressed the NB-specific molecular markers *TH*, *GD2 synthase*, *Phox2b*, *DCX*, *STX* and *ELAV-4* [18], at the same levels as primary tumors, as assessed by RT-qPCR (Figure 2H). Taken together, these results indicated that the GD2 positive fractions were enriched in metastatic NB cells.

### Gene expression profiling of BM-infiltrating NB cells

**Figure 3:**
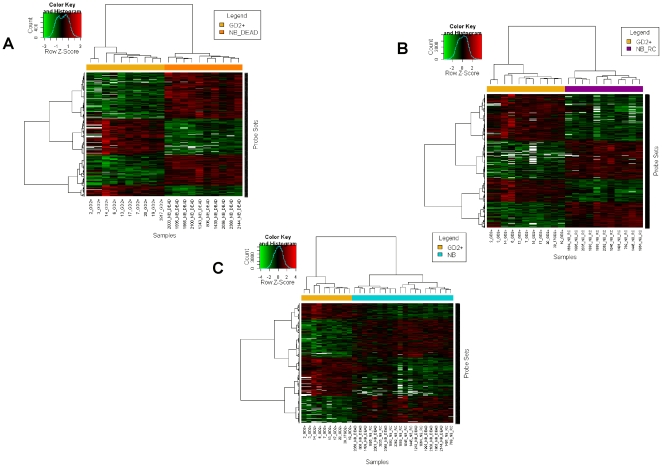
Gene expression profiling. Hierarchically clustered heat maps of differentially expressed probe sets in BM-infiltrating metastatic GD2 positive cells (GD2+) and (**A**) primary tumors from patients who died of stage 4 NB (NB_dead), (**B**) primary tumors from patients with stage 4 NB alive at 5 year follow-up (NB_RC), (**C**) primary tumors from patients with stage 4 NB, irrespective of outcome (NB). Each color patch represents the expression level of genes (row) in that sample (column), with a continuum of expression levels from bright green (lowest) to bright red (highest). Non-detected signals are indicated in white.

To identify genes specifically over- and under-expressed by BM-infiltrating NB cells compared with primary tumor cells, eleven freshly isolated GD2 positive preparations and twenty-one archived NB primary tumors were analyzed by microarrays. Eleven tumors were from alive patients and ten from patients who died of the disease at 5-year follow-up (Figure 2). The genes differently expressed in the three groups were identified by applying the significance analysis of microarrays (SAM) by paired comparisons with a false discovery rate (FDR) = 1%. As shown in Figure 3A–C, the samples from the three groups were placed on different trunks of unsupervised hierarchical clustering dendrograms, which demonstrated that the selected sequences successfully classified the biological specimens.

The expression profile of the BM-infiltrating cells differed from that of primary tumor cells from stage 4 patients dead at 5-year follow-up in 970 probe sets (corresponding to 332 up-regulated and 513 down-regulated unique transcripts), whereas 3158 probe sets (corresponding to 1366 up-regulated and 1253 down-regulated unique transcripts) were differently expressed compared to primary tumor cells from stage 4 patients alive at 5-year follow-up (Table S1). When compared to all twenty-one primary tumors the BM-infiltrating GD2 positive cells differed in 3146 probe sets, corresponding to 1435 down-regulated and 1224 up-regulated unique transcripts (Table S1).

The genes down-modulated in BM-infiltrating cells compared to primary tumor cells, irrespective of patient outcome, not surprisingly were genes necessary to sustain an organized, tri-dimensional structure, as that of a primary tumor, involving cell-matrix signaling and adhesion, cell differentiation and blood vessel development pathways (Table S2). The down-modulation of top-ranked genes (namely, *AGT*, *ATP1A2*, *BLK*, *CX3CL1*, *IGSF1*, *IGFBP3*, and *TNC*) was then tested by RT-qPCR in a subset of samples hybridized to microarrays (5 samples from each group, Figures S1, S2 and S3). *CX3CL1*, *AGT*, *ATP1A2*, and *IGSF1* mRNAs proved to be significantly down-modulated in the BM-infiltrating GD2 positive fractions compared to primary tumor cells (P<0.0001, P = 0.0325, P = 0.0196, P = 0.0047, respectively). These results were further confirmed in an independent set of 15 additional samples (5 for each group, Figures S4, S5 and S6) (P = 0013, P = 0.0012, P = 0.0023, P = 0.0314, respectively). It is noteworthy that *CX3CL1* (also called fractalkine) was down-modulated irrespective of patient outcome, whereas *AGT* and *ATP1A2* were down-modulated only compared to primary tumor cells from patients alive at 5-year follow-up (Figures S2, S3, S5, S6), which suggests that they may represent novel surrogate markers of tumor aggressiveness.

Among the genes significantly up-regulated in the BM-infiltrating GD2 positive cells compared to primary tumor cells (Table 1 and Table S2) several genes were commonly expressed by various lineages of BM resident cells, namely: i) cathelicidine (*CAMP*), myeloperoxidase (*MPO*), myeloid cell differentiation antigen (*MNDA*), *CD177*, *FC receptor*, *CD69* expressed by myelo-monocytic cells; ii) *mu chain*, *CD19*, and *BLK* expressed by B cells and iii) *IL-1β*, *IL8*, and *CXCL7* (*PPBP*) expressed by several hematopoietic and mesenchymal cells. To exclude that these findings were due to the small amount of normal resident BM cells present in our GD2 positive cell fractions, we directly checked whether the BM-infiltrating NB cells expressed the proteins encoded by these genes.

**Table 1:** Top-ranked genes differentially expressed by BM-infiltrating metastatic GD2 positive cells (GD2+) and primary tumor cells from patients with stage 4 NB, dead and alive at 5-year follow-up, selected by SAM analyses.

GeneName	Description	tumors stage 4 alive	GD2+	Diff. Mean
S100A9	Homo sapiens S100 calcium binding protein A9 (calgranulin B) (S100A9), mRNA [NM_002965]	5.66	12.89	−7.23
S100A8	Homo sapiens S100 calcium binding protein A8 (calgranulin A) (S100A8), mRNA [NM_002964]	8.55	15.09	−6.55
CAMP	Homo sapiens cathelicidin antimicrobial peptide (CAMP), mRNA [NM_004345]	5.3	11.52	−6.21
MPO	Homo sapiens myeloperoxidase (MPO), nuclear gene encoding mitochondrial protein, mRNA [NM_000250]	5.21	10.75	−5.54
MNDA	Homo sapiens myeloid cell nuclear differentiation antigen (MNDA), mRNA [NM_002432]	7.37	11.86	−4.49
CD177	full-length cDNA clone CS0DI015YO17 of Placenta Cot 25-normalized of Homo sapiens (human). [CR592446]	4.63	8.94	−4.31
LYZ	Homo sapiens lysozyme (renal amyloidosis) (LYZ), mRNA [NM_000239]	7.63	11.82	−4.19
FCAR	Homo sapiens Fc fragment of IgA, receptor for (FCAR), transcript variant 1, mRNA [NM_002000]	4.08	7.95	−3.87
LILRA2	Homo sapiens leukocyte immunoglobulin-like receptor, subfamily A (with TM domain), member 2 (LILRA2), mRNA [NM_006866]	4.4	8.1	−3.7
PPBP	Homo sapiens pro-platelet basic protein (chemokine (C-X-C motif) ligand 7) (PPBP), mRNA [NM_002704]	5.28	8.88	−3.59
HSH2D	Homo sapiens hematopoietic SH2 domain containing (HSH2D), mRNA [NM_032855]	6.53	9.96	−3.43
IL1B	Homo sapiens interleukin 1, beta (IL1B), mRNA [NM_000576]	7.13	10.55	−3.42
NFE2	Homo sapiens nuclear factor (erythroid-derived 2), 45kDa (NFE2), mRNA [NM_006163]	7.08	10.45	−3.37
TREM1	Homo sapiens triggering receptor expressed on myeloid cells 1 (TREM1), mRNA [NM_018643]	4.39	7.71	−3.32
IL18RAP	Homo sapiens interleukin 18 receptor accessory protein (IL18RAP), mRNA [NM_003853]	6.68	9.92	−3.24
NCF1	Homo sapiens neutrophil cytosolic factor 1, (chronic granulomatous disease, autosomal 1) (NCF1), transcript variant 1, mRNA [NM_000265]	7.54	10.78	−3.24
BLK	Homo sapiens B lymphoid tyrosine kinase (BLK), mRNA [NM_001715]	5.53	8.74	−3.21
P2RY2	Homo sapiens purinergic receptor P2Y, G-protein coupled, 2 (P2RY2), transcript variant 1, mRNA [NM_176072]	5.43	8.58	−3.15
CD19	Homo sapiens CD19 molecule (CD19), mRNA [NM_001770]	7.62	10.64	−3.02
FCRL5	Homo sapiens Fc receptor-like 5 (FCRL5), mRNA [NM_031281]	5.58	8.59	−3

GeneName	Description	tumors stage 4 dead	GD2+	Diff. Mean
RNASE2	Homo sapiens ribonuclease, RNase A family, 2 (liver, eosinophil-derived neurotoxin) (RNASE2), mRNA [NM_002934]	3.64	10.89	−7.25
S100A9	Homo sapiens S100 calcium binding protein A9 (S100A9), mRNA [NM_002965]	6.06	12.98	−6.92
S100A8	Homo sapiens S100 calcium binding protein A8 (S100A8), mRNA [NM_002964]	8.53	15.03	−6.51
S100P	Homo sapiens S100 calcium binding protein P (S100P), mRNA [NM_005980]	6.39	12.44	−6.05
MPO	Homo sapiens myeloperoxidase (MPO), nuclear gene encoding mitochondrial protein, mRNA [NM_000250]	4.77	10.68	−5.91
CAMP	Homo sapiens cathelicidin antimicrobial peptide (CAMP), mRNA [NM_004345]	5.75	11.38	−5.63
CD177	Homo sapiens CD177 molecule (CD177), mRNA [NM_020406]	3.87	9.17	−5.3
MNDA	Homo sapiens myeloid cell nuclear differentiation antigen (MNDA), mRNA [NM_002432]	7.1	12.16	−5.06
AQP9	Homo sapiens aquaporin 9 (AQP9), mRNA [NM_020980]	5.65	10.63	−4.98
RETN	Homo sapiens resistin (RETN), mRNA [NM_020415]	4.95	9.83	−4.88
LYZ	Homo sapiens lysozyme (renal amyloidosis) (LYZ), mRNA [NM_000239]	7.28	11.88	−4.6
LILRA3	Homo sapiens leukocyte immunoglobulin-like receptor, subfamily A (without TM domain), member 3 (LILRA3), mRNA [NM_006865]	5.03	9.48	−4.45
BLK	Homo sapiens B lymphoid tyrosine kinase (BLK), mRNA [NM_001715]	4.38	8.75	−4.37
FCAR	Homo sapiens Fc fragment of IgA, receptor for (FCAR), transcript variant 1, mRNA [NM_002000]	3.72	8.07	−4.35
CD19	Homo sapiens CD19 molecule (CD19), mRNA [NM_001770]	6.38	10.61	−4.23
IL1B	Homo sapiens interleukin 1, beta (IL1B), mRNA [NM_000576]	6.48	10.68	−4.2
CD37	Homo sapiens CD37 molecule (CD37), transcript variant 1, mRNA [NM_001774]	6.46	10.37	−3.91
SIGLEC5	Homo sapiens sialic acid binding Ig-like lectin 5 (SIGLEC5), mRNA [NM_003830]	6.05	9.9	−3.84
VNN2	Homo sapiens vanin 2 (VNN2), transcript variant 1, mRNA [NM_004665]	7.06	10.83	−3.77
CXCL1	Homo sapiens chemokine (C-X-C motif) ligand 1 (melanoma growth stimulating activity, alpha) (CXCL1), mRNA [NM_001511]	6.78	10.52	−3.74
NCF1	Homo sapiens neutrophil cytosolic factor 1, (chronic granulomatous disease, autosomal 1) (NCF1), mRNA [NM_000265]	7.11	10.8	−3.69
FCRL5	Homo sapiens Fc receptor-like 5 (FCRL5), mRNA [NM_031281]	4.87	8.55	−3.68
LILRA5	Homo sapiens leukocyte immunoglobulin-like receptor, subfamily A (with TM domain), member 5 (LILRA5), transcript variant 2, mRNA [NM_181985]	4.64	8.32	−3.68
P2RY2	Homo sapiens purinergic receptor P2Y, G-protein coupled, 2 (P2RY2), transcript variant 1, mRNA [NM_176072]	5.17	8.81	−3.64
CD69	Homo sapiens CD69 molecule (CD69), mRNA [NM_001781]	7.29	10.9	−3.62
IL8RB	Homo sapiens interleukin 8 receptor, beta (IL8RB), mRNA [NM_001557]	4.11	7.51	−3.4

### Expression and/or release of proteins encoded by up-regulated genes in BM-infiltrating GD2 positive cells

The surface membrane expression of CD177, CD37, and calprotectin, encoded by the *S100A8* and *S100A9* genes, was assessed by cytofluorimetric analysis in ten fresh BM samples from stage 4 patients with a median infiltration of 13% of GD2 positive cells (range 2.5–33%), as assessed by anti-GD2 immunocytochemistry [19]. We also tested 3 membrane proteins expressed by NB side population cells, namely: CD133, CD117 (c-kit) and CD271 (p75), and HLA-G, a monomorphic HLA class I molecule, whose expression was not previously found in NB primary tumors [20].

As shown in Table 2, the BM-infiltrating GD2 positive cells co-expressed CD177 and CD37 only in 3/9 and 3/6 BM samples, respectively. Moreover, only 30% of the GD2 positive cells co-expressed each of the two proteins (Table 2). Conversely, the BM-infiltrating GD2 positive cells co-expressed HLA-G and the S100A8/S100A9 complex (calprotectin) in 9/9 and 6/6 BM samples, respectively. Most importantly, 100% of the GD2 positive cells co-expressed both proteins (Table 2). With respect to NB side population markers, 100% of GD2 positive cells in 4/4 BM samples co-expressed p75, approximately 80% of GD2 positive cells co-expressed c-kit in 4/10 samples, while none of 7 GD2 positive cell preparations co-expressed CD133 (Table 2).

**Table 2:** Surface expression and identification of released proteins encoded by genes up-regulated in BM-infiltrating GD2 positive preparations, as assessed by cytofluorimetric analysis ^a^ and ELISA ^b^.

Protein	Number of samples	Positive(N)	%	Mean of co-expression^c^
CD177^a^	9	3	33	30.5
CD37^a^	6	3	50	29.0
HLA-G^a^	9	9	100	100
Calprotectin^a^	6	6	100	100
CD271 (p75)^a,^ d	4	4	100	100
CD117 (c-kit)^a,^ e	10	4	34	77.7
CD133^a,^ f	7	0	0	0
CXCL7^b^	3	3	100	
CXCL1^b^	3	1	33	
IL-1β^b^	3	0	0	

***^c^***percentage of double positive cells.

d Bassili *et al*, Cancer Chemother Pharmacol 2010; 65:1047–56.

e Hirschmann-Jax *et al*, Proc Natl Acad Sci U S A 2004;101:14228–33.

f Komuro *et al*, J Pediatr Surg 2007;42: 2040–45, Tong *et al*, World J Pediatr 2008;4:58–62.

**Figure 4:**
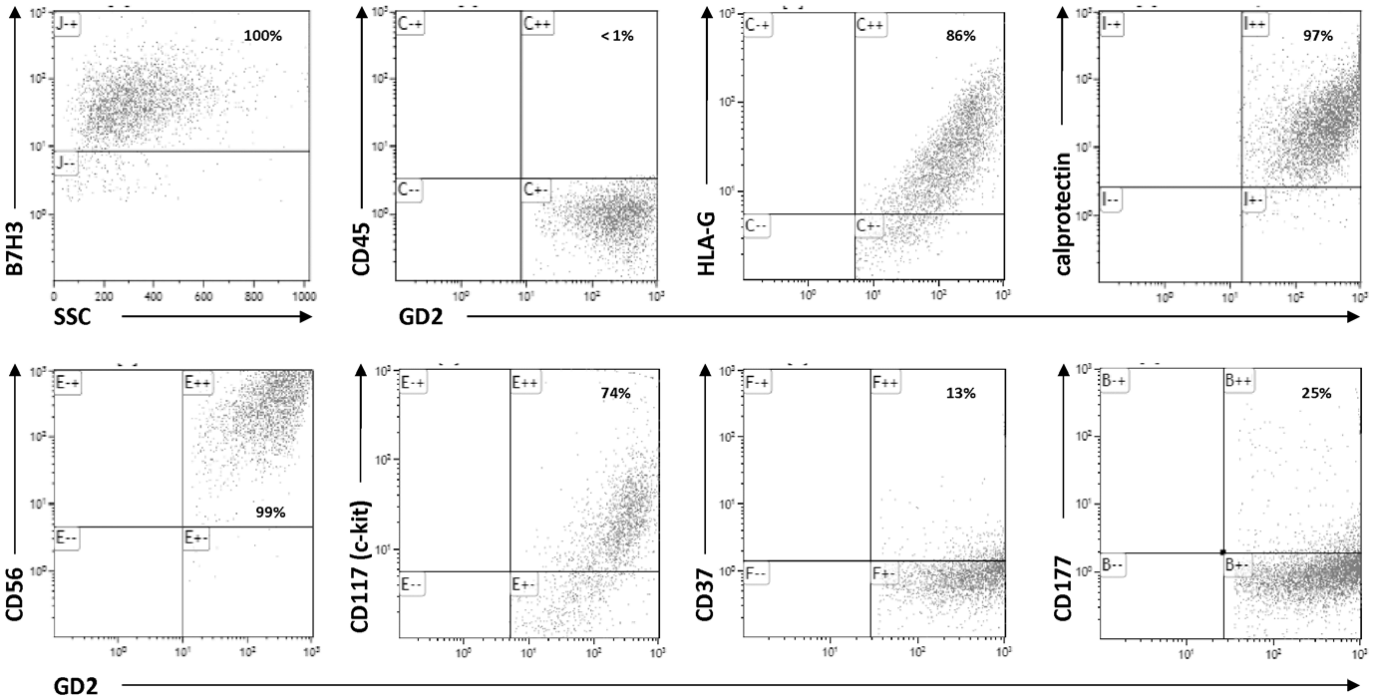
Flow cytometric analysis of BM samples. A representative cytofluorometric analysis of a freshly isolated GD2 positive cell preparation, tested with anti-B7H3 (5B14) and anti-GD2 mAb in combination with: anti-CD45, anti-HLA-G, anti-calprotectin, anti-CD56, anti-CD117 (c-kit), anti-CD37 and anti-CD177. The first plot shows anti-B7H3 specific mAb fluorescence intensity (Y axis) *versus* side-scatter (X axis), whereas all the other plots show specific mAb fluorescence intensity (Y axes) *versus* anti-GD2 specific mAb fluorescence intensity (X axes). Letters indicated in each panel are assigned from Kaluza software to each different single sample analysis. ^++^ indicates double positive cells (upper right panel), ^+−^ and ^−+^ indicate single positive cells (upper left and lower right panels), ^−−^ indicates double negative cells (lower left panel). Percentages of single positive (first plot) or double positive (all the other plots) cells are indicated.

Then we performed FACS analysis on three freshly-isolated GD2 positive cell preparations to further confirm co-expression of HLA-G and calprotectin by the BM-infiltrating NB cells. As shown in Figure 4, the GD2 positive cells were CD45 negative, expressed the B7H4 and CD56 NB-specific markers, and co-expressed HLA-G and calprotectin, whereas c-kit, CD37 and CD177 were co-expressed by a fraction of cells.

Next, we investigated whether freshly isolated BM-infiltrating GD2 positive cells could release IL-1β, CXCL1, and CXCL7 in the culture supernatant. Conditioned media from three preparations cultured for 48 hours were tested by ELISA. As reported in Table 2, all three GD2 positive preparations released CXCL7 (mean = 1.39 µg/mL), 1 out of 3 released CXCL1 (0.91 µg/mL) and none released IL-1β.

## Discussion

The aim of this study was to identify genes and proteins differentially expressed by BM-infiltrating NB cells that could be new potential therapeutic target and/or prognostic factors for high risk NB patients. The results showed a significant down-regulation of genes involved in extracellular matrix-cell adhesion pathways and in blood vessel development. The genes more significantly down-modulated were *CX3CL1*, *AGT*, and *ATP1A2*. CX3CL1 (fractalkine) is a neuronal chemokine capable of inducing immune effector cell migration, adhesion and IFN-γ secretion [21]. Intriguingly, CX3CL1 down-modulation was observed in *MYCN*-amplified primary NB tumors [22], supporting a role in limiting tumor aggressiveness. *AGT* and *ATP1A2* down-modulations were significantly associated with patient outcome, which suggests that expression of these proteins in primary tumors may have a prognostic value. *AGT* encodes angiotensinogen, the precursor of angiotensin, a key protein involved in blood vessel formation, and *ATP1A2* is a sodium-potassium ATPase, implicated in ion-exchange signaling. Further studies in larger cohorts of NB patients, however, are needed to address their prognostic role.

In keeping with the specific aim of our study we focused instead on the up-regulated genes. The BM-infiltrating GD2 positive cells compared to primary tumor cells over-expressed several genes that are normally expressed by different lineages of resident BM cells. Our GD2 positive cell preparations were enriched in CD56+ and NB84+ mononuclear NB cells, showed the same genetic aberration as the primary tumor cells, and expressed NB-specific genes, such as *TH*, *GD2 synthase*, *Phox2b*, *ELAV4* and *DCX*, at the same levels as primary tumor cells. However, they could not be considered pure, therefore to avoid bias we evaluated the expression of the proteins encoded by these genes in fresh BM samples from stage 4 patients, together with proteins specifically expressed by NB cells, by NB cell side population, and NB TICs. Whereas in unprocessed BM samples from healthy individuals GD2 expression is absent [23], in BM samples from stage 4 NB patients GD2 positive cells represented about 20–30% of mononuclear cells and 100% of these cells always co-express NB specific markers, as p75, CD56, and B7H3, thus confirming that the GD2-positive BM-infiltrating cells were indeed metastatic NB cells. Surface expression of CD133 never occurred whereas expression of CD177 and CD37, two clusters of hematopoietic differentiation, was occasionally detected in fractions of BM-infiltrating GD2 positive cells, in a way similar to CD117 (c-kit). Expression of CD37, a member of the tetraspanin family, has been involved in metastasis [24], while nothing is known about the possible role in cancer of CD177, a glycosyl-phosphatidylinositol (GPI)-linked surface protein, normally secreted by neutrophils [25]. Thus, our findings suggest that the role, if any, of CD133, CD117, CD37 and CD177 can not be pivotal in determining the aggressiveness of the metastatic cells. Similarly, CXCL1 secretion was not consistent, even if CXCL1 was shown to accelerate tumor growth, angiogenesis, and stromal recruitment during the metastatic process in melanoma, breast and colon cancer [26].

Conversely, the BM-infiltrating GD2 positive cells released the chemokine CXCL7, encoded by the *PPBP* gene. *CXCL7* was found over-expressed in breast cancer patients [27], and *CXCL7*-transfected breast cancer cells were more invasive than parental cells [28]. More interestingly, all freshly isolated GD2 positive cells co-expressed not only CD56 and B7H3 proteins [29], [30], but also calprotectin and HLA-G. The heterodimeric protein calprotectin, a product of the *S100A8* and *S100A9* genes, is a member of the S100 protein family, composed of small (10–12 kDa) acidic calcium and zinc binding proteins. Calprotectin is normally expressed by phagocytes and polymorphonuclear leukocytes. The S100A8/A9 complex is located in the cytosol and, upon elevation of intracellular calcium levels, is translocated to the cytoskeleton and then to the plasma membrane. Lacking a trans-membrane signaling domain, calprotectin remains attached by non-covalent association and, during inflammation, the S100A8/A9 complex is released into biological fluids. Calprotectin may induce apoptosis of different cell types, through zinc sequestration, and it has been widely used as biomarker of inflammation [31]. Moreover, calprotectin has been identified as a potent ligand of the Toll-like receptor 4 (TLR4) [32], which is responsible for specific response to endogenous danger signals. Since we recently demonstrated [33] that resident BM cells from patients with NB up-regulated the expression of genes involved in inflammation and response to biotic stimuli, it is tempting to speculate that calprotectin may be one of the signals involved.

In spite of its properties as enhancer of innate immune responses, calprotectin was found selectively expressed by invasive breast [34] and thyroid [35] cancer cells. Recently, its expression was associated with cancer progression and metastasis in different human tumors, which revealed that calprotectin is a Janus-faced molecule [36]. Hiratsuka *et al.* [37] demonstrated that the calprotectin-TLR4 axis guides metastatic cell invasion, and facilitates the survival and proliferation of cancer cells at the metastatic site. Thus, calprotectin expressed by BM-infiltrating metastatic NB cells may contribute to their survival and proliferation in the BM.

The BM-infiltrating GD2 positive cells also expressed HLA-G, a monomorphic HLA class Ib molecule, over-expressed in a wide variety of human cancers, endowed with tolerogenic properties facilitating tumor escape from host immune response [38]. Since primary NB tumors did not express HLA-G [20], it is conceivable that HLA-G may contribute to the high aggressiveness of BM-infiltrating NB cells throughout its immunosuppressive activity. Therefore, the search for drugs able to suppress HLA-G expression might increase the efficacy of immunotherapeutic intervention in high risk patients.

Further studies will be necessary to understand whether the expression of calprotectin and HLA-G by BM-infiltrating NB cells is transcriptionally regulated [39] or acquired by mechanisms such as cell fusion [40] or microvesicles [41]. Nonetheless, our study demonstrated that BM-infiltrating NB cells express genes not found in the primary tumor cells of patients with stage 4 disease, which may be responsible in part for their dismal prognosis. Since BM microenvironment in patients with localized and metastatic NB was similar despite their completely different outcomes [33], the identification of proteins specifically expressed by BM-infiltrating metastatic cells represents the only effective tool to find novel therapeutic targets and novel prognostic markers for high risk NB patients. In this view, calprotectin and HLA-G may deserve future studies.

## Materials and Methods

### Study design

Sixty-six Italian children over 18 months of age, diagnosed with stage 4 NB according to INSS criteria [42] were included in the study. Diagnosis and staging were made at the referral center participating in the Italian Cooperative Group for Neuroblastoma. Central review of primary tumor histology was performed at the Gaslini Institute. Diagnostic work-up included evaluation of the primary tumor by appropriate imaging, morphological examination of two BM aspirates and at least one bone trephine biopsy, catecholamine metabolite assay in urine, ferritin and lactate dehydrogenase assays in serum, and evaluation of *MYCN* oncogene status in the primary tumor. Demographic, clinical and follow-up data were retrieved from the Italian Neuroblastoma Registry [43].

The study was approved by the Ethics Committees of each Italian Cooperative Group for Neuroblastoma center (http://www.aieop.org/?q=mappa centri.html). All patients' guardians signed a written consent allowing the use of samples and clinical data for research purposes. The procedures were in accordance with ethical standards and the Helsinki Declaration.

As depicted in Figure 1, archived primary NB tumors and freshly isolated GD2 positive BM-infiltrating NB cells collected at diagnosis were included in the study. All tumors were classified as Schwannian stroma poor NBs according to the International Neuroblastoma Pathology Committee [44]. They contained at least 80% of neuroblastoma cells, as assessed by the pathologist (CG). Three tumors showed *MYCN* amplification, one in the first group and two in the second group. The use of primary tumors stored in the Gaslini tumor repository “Biobanca integrata tessuto-genomica” was approved by the Gaslini's Ethics Committee.

### Immunomagnetic separation of BM-infiltrating NB cells

To isolate BM-infiltrating NB cells, fresh BM samples from patients with stage 4 disease older than 18 months at diagnosis, containing 10–30% of NB cells, as assessed by immunocytochemical analyses [19] were subjected to immunomagnetic bead manipulation, as described in details in Scaruffi *et al.* [33]. The GD2 positive fraction, containing less than 5% of CD45+ hematopoietic cells as assessed by flow cytometry, was centrifuged and the cell pellet subjected to further analyses (Figure 1). The amount of recovered GD2 positive cells was around 10^6^/mL of BM sample.

### Cytofluorimetric analysis

For characterization of GD2 positive cell preparations (Figure 1), surface membrane staining was done using the anti-B7H3 (5B14, kindly provided by Prof. A. Moretta) [30] and the anti-CD45 (Becton Dickinson, San Jose, CA) monoclonal antibodies (mAbs).

To evaluate surface protein expression by GD2 positive cells, whole BM samples from ten stage 4 NB patients and three freshly isolated BM-infiltrating GD2^+^ cells were stained with the following mAbs: anti-GD2 (purified from the G2a hybridoma cell line [45]), anti-B7H3 (5B14), anti-p75 (Novus Biologicals, Littleton, CO), anti-HLA-G PE (Exbio, Prague, CZ), anti-calprotectin FITC (BMA Biomedicals, Augst, Switzerland), anti-CD177 FITC (Becton Dickinson), anti-CD37 FITC (Becton Dickinson), anti-CD133 APC (Miltenyi Biotech, Bergisch Gladbach, Germany), anti-CD117 PE (Miltenyi Biotech), anti-CD45 FITC (Becton Dickinson) and anti-CD56 PC7 (Beckman Coulter, Brea, CA). Irrelevant isotype-matched mAbs, used at the same concentration as the test mAbs, were used as negative control. One×10^4^ events were acquired using a Gallios Cytometer (Beckman Coulter) and data were analyzed with the Kaluza software (Beckman Coulter). The percentage of positive cells was calculated using a threshold line based on the maximum fluorescence intensity obtained with the irrelevant isotype-matched mAb.

### Immunocytochemistry of GD2 positive cell preparations

Immunocytochemical labeling was performed according to standardized procedures for BM samples [46]. Briefly, cytospins of freshly isolated GD2 positive cells were fixed in 4% buffered para-formaldehyde for 10 minutes. After washing with PBS, cytospins were incubated for 30 minutes with proper dilutions of unlabeled anti-GD2 (Becton Dickinson), anti-NB84 and anti-CD56 (both from Dako, Golstrup, Denmark) in 1% PBS/BSA (Gibco, Paisley, UK). After washing in PBS, cytospins were incubated for 30 minutes with an unlabeled secondary antibody (Dako) diluted in 1% PBS/BSA. After washing, staining was obtained by incubation with APAAP complex (Dako) and Fuchsin+Substrate Chromogene System (Dako). Cytospins were counterstained with hematoxylin (Sigma, St Louis, MO) and mounted in aqueous medium (Dako). As negative controls, slides were incubated with mouse normal serum (X0910, Dako) and processed as above.

### FISH analysis

Double colour FISH was performed on interphase nuclei of GD2 positive cell preparations using the ON MYCN(2p24)/LAF(2q11) probe (Kreatech, Amsterdam, The Nederlands). Cytospins were pretreated with RNase and pepsin. Probes were handled according to the manufacturer's protocol: slide and probe combinations were incubated simultaneously on a hot plate at 80°C for 5 min. Overnight incubation at 37°C, washes and DAPI nuclei counterstaining were done according to standard protocol. For each tumor at least 200 nuclei were surveyed. The terminology used was in accordance with the guidelines of the European Neuroblastoma Quality Assessment (ENQUA) group [47].

### Extraction of total RNA

Total RNA was extracted from the GD2 positive cell preparations using the RNeasy Micro kit (Qiagen, Helden, Germany), according to the manufacturer's procedure that includes an RNase-free DNAse I digestion to eliminate contaminating DNA. From primary NB tumors total RNA was extracted as previously described [9]. Quantification and quality control of total RNA were performed by RNA 6000 Nano® and Pico® assays on a 2100 Bioanalyzer (Agilent Technologies, Santa Clara, CA). Only samples with RNA integrity number ≥6.0 were included in the study.

### Gene expression profiling

Five-hundred ng of total RNA were hybridized to Human GE 4x44K v2 Microarray Kit (Agilent Technologies) containing probes for 41,000 human transcripts following the One-color microarray-based gene expression analysis protocol (www.agilent.com), as described in detail in Scaruffi *et al.* [33]. All data is MIAME compliant and that the raw data has been deposited in National Center for Biotechnology Information Gene Expression Omnibus (GEO, http://www.ncbi.nlm.nih.gov/geo/) and are accessible through GEO accession number GSE 25623a.

### Gene Ontology analysis

DAVID software was used to determine biological pathways significantly over-represented in our datasets.

### Reverse transcription and quantitative real-time PCR (RT-qPCR)

RNA (1 µg) was reverse transcribed using random primers and 120 U Superscript III (MMLV) reverse-transcriptase in a total volume of 20 µL (Invitrogen Life Technologies, Carlsbad, CA). cDNA was diluted 10-fold in molecular biology grade water to a final concentration of 5 ng/µL assuming 100% reverse transcription efficiency.

For GD2 positive cell characterization (Figure 1), level of expression of *TH*, *beta-1,4-N-acetyl-galactosaminyl transferase 1* (*GD2-synthase*), *DCX*, *ELAV-4*, *Phox2b* and *STX* was evaluated according to standardized conditions [48], using 8 primary NB tumors as positive controls.

For validation of microarray results (Figure 1), we first assessed *ATP5B* as the best reference gene for both GD2-positive and primary tumor cells by means of geNorm™ Housekeeping Gene Selection Kit and software (v. 3.4) (PrimerDesign Ltd, UK). Then, the expression of *AGT*, *ATP1A2*, *BLK*, *CX3CL1*, *IGFBP3*, *IGSF2* and *TNC* genes was quantified by pre-optimized FAM-labeled TaqMan® Gene Expression assays (Applied Biosystem, Foster City, CA). Negative controls (water as template and RNA reverse-transcribed without MMLV) and a positive control (cDNA from the IMR-32 NB cell line [48] were always included. To determine the expression of each target gene relative to *ATP5B*, the equation 2^−ΔCq^, where ΔCq = (Cq_target gene_−Cq*_β2M_*), was calculated.

A checklist prepared according to the Minimum Information for Publication of Quantitative Real-Time PCR Experiments (MIQE) is submitted as Supplementary Data.

### Secretion of cytokines

Secretion of CXCL1, CXCL7, and IL-1β was assessed by using DuoSet ELISA Development System (R&D Systems, Minneapolis, MN), following manufacturer's protocol. Briefly, supernatants from three freshly separated GD2 positive cell preparations were collected after 48-hour culture in DMEM containing 20% FBS. H_2_SO_4_ 5 M (100 µL/well) was added to stop the reaction and optical densities were measured at 450 nm using Infinite M200 reader (Tecan, Männedorf, Switzerland). The assay's lowest threshold was 7.8 ng/mL for all analytes. Each sample was tested in duplicate.

### Statistical analysis

Statistical analyses of gene expression profiling and of RT-qPCR data were performed as detailed in Scaruffi *et al.* (in press PBC). All significance tests were two tailed and a P value<0.05 was considered as statistically significant.

## Supporting Information

Table S1 Transcripts differentially expressed between BM-infiltrating GD2 positive cells and primary tumors from i) patients dead of stage 4 NB, ii) patients with stage 4 NB alive at 5 year follow-up, and iii) patients with stage 4 NB, irrespective of outcome, selected by SAM analysis.
(PDF)

Table S2 The most significantly (P≤0.05) enriched Gene Ontology Biological Process (GOBP) terms in gene signatures of BM-infiltrating GD2 positive cells and primary NB tumors from stage 4 patients.
(XLS)

Figure S1 Box-and-Whisker plots of *AGT*, *ATP1A2*, *BLK*, *CX3CL1*, *IGSF1*, *IGFBP3*, and *TNC* gene expression values by qPCR (normalized to *ATP5B*, and after logarithmic transformation of original measures) in 5 GD2 positive cells (GD2+) and 5 primary tumors (NB) previously hybridized to microarray. Each box represents the values from the 25^th^ to 75^th^ percentile, the middle line represents the median, and a line extends from the minimum to the maximum value, excluding outliers which are displayed as blue dots.
(TIF)

Figure S2 Box-and-Whisker plots of *AGT*, *ATP1A2*, *BLK*, *CX3CL1*, *IGSF1*, *IGFBP3*, and *TNC* gene expression values by qPCR (normalized to *ATP5B*, and after logarithmic transformation of original measures) in 5 GD2 positive cells (GD2+) and 5 primary tumors from patients dead of disease (NB_dead), previously hybridized to microarrays. Each box represents the values from the 25^th^ to 75^th^ percentile, the middle line represents the median, and a line extends from the minimum to the maximum value.
(TIF)

Figure S3 Box-and-Whisker plots of *AGT*, *ATP1A2*, *BLK*, *CX3CL1*, *IGSF1*, *IGFBP3*, and *TNC* gene expression values by qPCR (normalized to *ATP5B*, and after logarithmic transformation of original measures) in 5 GD2 positive cells (GD2+) and 5 primary tumors from alive patients (NB_alive), previously hybridized to microarrays. Each box represents the values from the 25^th^ to 75^th^ percentile, the middle line represents the median, and a line extends from the minimum to the maximum value, excluding outliers which are displayed as blue dots.
(TIF)

Figure S4 Box-and-Whisker plots of *AGT*, *ATP1A2*, *CX3CL1*, and *IGSF1* gene expression values by qPCR (normalized to *ATP5B*, and after logarithmic transformation of original measures) in 5 GD2 positive cells (GD2+) and 5 primary tumors from stage 4 patients (NB) (independent sample set). Each box represents the values from the 25^th^ to 75^th^ percentile, the middle line represents the median, and a line extends from the minimum to the maximum value, excluding outliers which are displayed as blue dots.
(TIF)

Figure S5 Box-and-Whisker plots of *AGT*, *ATP1A2*, *CX3CL1*, and *IGSF1* gene expression values by qPCR (normalized to *ATP5B*, and after logarithmic transformation of original measures) in 5 GD2 positive cells (GD2+) and 5 primary tumors from stage 4 patients dead of disease (NB_dead) (independent sample set). Each box represents the values from the 25^th^ to 75^th^ percentile, the middle line represents the median, and a line extends from the minimum to the maximum value, excluding outliers which are displayed as blue dots.
(TIF)

Figure S6 Box-and-Whisker plots of *AGT*, *ATP1A2*, *CX3CL1*, and *IGSF1* gene expression values by qPCR (normalized to *ATP5B*, and after logarithmic transformation of original measures) in 5 GD2 positive cells (GD2+) and 5 primary tumors from alive stage 4 patients (NB_alive) (independent sample set). Each box represents the values from the 25^th^ to 75^th^ percentile, the middle line represents the median, and a line extends from the minimum to the maximum value, excluding outliers which are displayed as blue dots.
(TIF)
