# Supplementary material for: Bone Marrow-Infiltrating Human Neuroblastoma Cells Express High Levels of Calprotectin and HLA-G Proteins
Source: PLoS One. 2012 Jan 9;7(1):e29922. doi: 10.1371/journal.pone.0029922 (PMC3253802; doi:10.1371/journal.pone.0029922)
Supplement: Table S1 — Transcripts differentially expressed between BM-infiltrating GD2 positive cells and primary tumors from i) patients dead of stage 4 NB, ii) patients with stage 4 NB alive at 5 year follow-up, and iii) patients with stage 4 NB, irrespective of outcome, selected by SAM analysis. (PDF) [file pone.0029922.s001.pdf]

Supplementary Table 1
Morandi et al

GeneName	Description	MEAN expression stage 4 NB_DEAD	MEAN expression GD2+ cells	Difference of mean expression (stage 4_DEAD - GD2+ cells)
CLEC14A	Homo sapiens C-type lectin domain family 14, member A (CLEC14A), mRNA [NM_175060]	10,7	3,36	7,34
CCL19	Homo sapiens chemokine (C-C motif) ligand 19 (CCL19), mRNA [NM_006274]	12,08	5,37	6,71
VWF	Homo sapiens von Willebrand factor (VWF), mRNA [NM_000552]	13,36	7,05	6,31
MYH11	Homo sapiens myosin, heavy chain 11, smooth muscle (MYH11), transcript variant SM2B, mRNA [NM_001040113]	9,25	3,02	6,23
COL3A1	Homo sapiens collagen, type III, alpha 1 (Ehlers-Danlos syndrome type IV, autosomal dominant) (COL3A1), mRNA [NM_000090]	13,58	7,36	6,22
HIGD1B	Homo sapiens HIG1 domain family, member 1B (HIGD1B), mRNA [NM_016438]	8,55	2,73	5,82
H19	Homo sapiens H19, imprinted maternally expressed untranslated mRNA (H19) on chromosome 11 [NR_002196]	11,89	6,08	5,81
SOD3	Homo sapiens superoxide dismutase 3, extracellular (SOD3), mRNA [NM_003102]	11,58	6,01	5,57
MGP	Homo sapiens matrix Gla protein (MGP), mRNA [NM_000900]	12,3	6,87	5,43
ROBO4	Homo sapiens roundabout homolog 4, magic roundabout (Drosophila) (ROBO4), mRNA [NM_019055]	9,78	4,4	5,38
LOC338328	Homo sapiens high density lipoprotein-binding protein (LOC338328), mRNA [NM_178172]	9,84	4,46	5,38
GJA4	Homo sapiens gap junction protein, alpha 4, 37kDa (connexin 37) (GJA4), mRNA [NM_002060]	9,6	4,23	5,38
SMOC2	Homo sapiens SPARC related modular calcium binding 2 (SMOC2), mRNA [NM_022138]	12,07	6,94	5,13
HEYL	Homo sapiens hairy/enhancer-of-split related with YRPW motif-like (HEYL), mRNA [NM_014571]	11,81	6,69	5,12
AQP1	Homo sapiens aquaporin 1 (Colton blood group) (AQP1), mRNA [NM_198098]	12,91	7,79	5,12
NR2F2	Homo sapiens nuclear receptor subfamily 2, group F, member 2 (NR2F2), mRNA [NM_021005]	11,59	6,57	5,02
PLVAP	Homo sapiens plasmalemma vesicle associated protein (PLVAP), mRNA [NM_031310]	9,1	4,37	4,73
IL33	Homo sapiens interleukin 33 (IL33), mRNA [NM_033439]	8,31	3,62	4,7
UBD	Homo sapiens ubiquitin D (UBD), mRNA [NM_006398]	10,55	5,98	4,57
CCDC102B	Homo sapiens coiled-coil domain containing 102B (CCDC102B), mRNA [NM_024781]	8,77	4,2	4,57
CXorf36	Homo sapiens chromosome X open reading frame 36 (CXorf36), mRNA [NM_024689]	8,17	3,67	4,5
NOSTRIN	Homo sapiens nitric oxide synthase trafficker (NOSTRIN), transcript variant 1, mRNA [NM_052946]	9,17	4,69	4,48
COL4A1	Homo sapiens collagen, type IV, alpha 1 (COL4A1), mRNA [NM_001845]	11,75	7,27	4,48
ADH1C	Homo sapiens alcohol dehydrogenase 1C (class I), gamma polypeptide (ADH1C), mRNA [NM_000669]	7,35	2,88	4,47
VWA1	Homo sapiens von Willebrand factor A domain containing 1 (VWA1), transcript variant 1, mRNA [NM_022834]	12,22	7,8	4,41
THC2678294	FGD5_HUMAN (Q6ZNL6) FYVE, RhoGEF and PH domain-containing protein 5 (Zinc finger FYVE domain-containing protein 23), complete [THC2678294]	9,76	5,36	4,4
PLAT	Homo sapiens plasminogen activator, tissue (PLAT), transcript variant 1, mRNA [NM_000930]	10,82	6,48	4,34

Supplementary Table 1
Morandi et al

TMEM16A	Homo sapiens transmembrane protein 16A (TMEM16A), mRNA [NM_018043]	8,86	4,62	4,25
OLFML2B	Homo sapiens olfactomedin-like 2B (OLFML2B), mRNA [NM_015441]	9,27	5,02	4,25
GUCY1A3	Homo sapiens guanylate cyclase 1, soluble, alpha 3 (GUCY1A3), mRNA [NM_000856]	11,39	7,13	4,25
A2M	Homo sapiens alpha-2-macroglobulin (A2M), mRNA [NM_000014]	14,31	10,24	4,07
MMRN2	Homo sapiens multimerin 2 (MMRN2), mRNA [NM_024756]	8,52	4,48	4,04
CCL21	Homo sapiens chemokine (C-C motif) ligand 21 (CCL21), mRNA [NM_002989]	8,2	4,25	3,95
RARRES2	Homo sapiens retinoic acid receptor responder (tazarotene induced) 2 (RARRES2), mRNA [NM_002889]	10,62	6,72	3,9
PTPRB	Homo sapiens protein tyrosine phosphatase, receptor type, B (PTPRB), mRNA [NM_002837]	7,74	3,87	3,87
FCN3	Homo sapiens ficolin (collagen/fibrinogen domain containing) 3 (Hakata antigen) (FCN3), transcript variant 1, mRNA [NM_003665]	7,67	3,81	3,86
SEMA3G	Homo sapiens sema domain, immunoglobulin domain (Ig), short basic domain, secreted, (semaphorin) 3G (SEMA3G), mRNA [NM_020163]	8,01	4,16	3,85
TNXB	Homo sapiens tenascin XB (TNXB), transcript variant XB-S, mRNA [NM_032470]	11,4	7,57	3,84
NTRK1	Homo sapiens neurotrophic tyrosine kinase, receptor, type 1 (NTRK1), transcript variant 2, mRNA [NM_002529]	11,05	7,22	3,83
C1R	Homo sapiens complement component 1, r subcomponent (C1R), mRNA [NM_001733]	11,14	7,31	3,83
C1S	Homo sapiens complement component 1, s subcomponent (C1S), transcript variant 1, mRNA [NM_001734]	12,7	8,88	3,82
USHBP1	Homo sapiens Usher syndrome 1C binding protein 1 (USHBP1), mRNA [NM_031941]	7,12	3,41	3,71
NPR1	Homo sapiens natriuretic peptide receptor A/guanylate cyclase A (atrionatriuretic peptide receptor A) (NPR1), mRNA [NM_000906]	7,17	3,46	3,71
PTGDS	Homo sapiens prostaglandin D2 synthase 21kDa (brain) (PTGDS), mRNA [NM_000954]	13,25	9,58	3,68
CGNL1	Homo sapiens cingulin-like 1 (CGNL1), mRNA [NM_032866]	10,81	7,13	3,68
INS	Homo sapiens insulin (INS), mRNA [NM_000207]	9,38	5,7	3,67
KCNJ8	Homo sapiens potassium inwardly-rectifying channel, subfamily J, member 8 (KCNJ8), mRNA [NM_004982]	9,26	5,62	3,65
GPR4	Homo sapiens G protein-coupled receptor 4 (GPR4), mRNA [NM_005282]	8,5	4,86	3,65
SYNPO	Synaptopodin, [Source:Uniprot/SWISSPROT;Acc:Q8N3V7] [ENST00000307662]	10,25	6,62	3,63
LRRC32	Homo sapiens leucine rich repeat containing 32 (LRRC32), mRNA [NM_005512]	10,12	6,49	3,63
SPON2	Homo sapiens spondin 2, extracellular matrix protein (SPON2), mRNA [NM_012445]	13,49	9,91	3,58
DOCK6	Homo sapiens dedicator of cytokinesis 6 (DOCK6), mRNA [NM_020812]	9,24	5,66	3,58
GPC3	Homo sapiens glypican 3 (GPC3), mRNA [NM_004484]	7,5	3,93	3,57
SPRY1	Homo sapiens sprouty homolog 1, antagonist of FGF signaling (Drosophila) (SPRY1), transcript variant 2, mRNA [NM_199327]	8,3	4,75	3,55
ENST0000038165	Probable phospholipid-transporting ATPase IB (EC 3,6,3,1) (ATPase class I type 8A member 2) (ML-1), [Source:Uniprot/SWISSPROT;Acc:Q9NTI2] [ENST00000381655]	11,66	8,12	3,54

Supplementary Table 1
Morandi et al

C7	Homo sapiens complement component 7 (C7), mRNA [NM_000587]	8,6	5,06	3,54
PRELP	Homo sapiens proline/arginine-rich end leucine-rich repeat protein (PRELP), transcript variant 1, mRNA [NM_002725]	8,03	4,5	3,53
IGF2	Homo sapiens insulin-like growth factor 2 (somatomedin A) (IGF2), transcript variant 1, mRNA [NM_000612]	14,27	10,75	3,51
MMP11	Homo sapiens matrix metallopeptidase 11 (stromelysin 3) (MMP11), mRNA [NM_005940]	10,58	7,09	3,49
LOC642652	PREDICTED: Homo sapiens hypothetical LOC642652 (LOC642652), mRNA [XR_016196]	10,2	6,76	3,44
CGI-38	Homo sapiens brain specific protein (CGI-38), mRNA [NM_016140]	11,12	7,71	3,41
PDGFRB	Homo sapiens platelet-derived growth factor receptor, beta polypeptide (PDGFRB), mRNA [NM_002609]	11,69	8,29	3,4
GGTLA1	Homo sapiens gamma-glutamyltransferase-like activity 1 (GGTLA1), mRNA [NM_004121]	9,81	6,42	3,4
GNG11	Homo sapiens guanine nucleotide binding protein (G protein), gamma 11 (GNG11), mRNA [NM_004126]	11,5	8,19	3,32
THC2527772	HUMC4AA2 complement component C4A {Homo sapiens} (exp=-1; wgp=0; cg=0), partial (6%) [THC2527772]	8,82	5,52	3,3
PRPF19	Homo sapiens PRP19/PSO4 pre-mRNA processing factor 19 homolog (S, cerevisiae) (PRPF19), mRNA [NM_014502]	9,66	6,38	3,27
ANKRD47	Homo sapiens ankyrin repeat domain 47 (ANKRD47), mRNA [NM_198471]	9,04	5,77	3,27
AOC3	Homo sapiens amine oxidase, copper containing 3 (vascular adhesion protein 1) (AOC3), mRNA [NM_003734]	9,23	5,96	3,27
PTHR1	Homo sapiens parathyroid hormone receptor 1 (PTHR1), mRNA [NM_000316]	7,53	4,28	3,25
FZD4	Homo sapiens frizzled homolog 4 (Drosophila) (FZD4), mRNA [NM_012193]	10,71	7,47	3,25
-	chr2:088891141-088891082	8,52	5,27	3,25
IFI27	Homo sapiens interferon, alpha-inducible protein 27 (IFI27), mRNA [NM_005532]	13,98	10,76	3,23
RGS16	Homo sapiens regulator of G-protein signalling 16 (RGS16), mRNA [NM_002928]	11,6	8,39	3,21
NKD2	Homo sapiens naked cuticle homolog 2 (Drosophila) (NKD2), mRNA [NM_033120]	8,52	5,32	3,21
CYB561	Homo sapiens cytochrome b-561 (CYB561), transcript variant 2, mRNA [NM_001017916]	10,64	7,43	3,21
ESAM	Homo sapiens endothelial cell adhesion molecule (ESAM), mRNA [NM_138961]	8,23	5,03	3,2
BCL2	Homo sapiens B-cell CLL/lymphoma 2 (BCL2), nuclear gene encoding mitochondrial protein, transcript variant alpha, mRNA [NM_000633]	12,01	8,81	3,2
FMO3	Homo sapiens flavin containing monooxygenase 3 (FMO3), transcript variant 2, mRNA [NM_001002294]	7,5	4,33	3,17
GALNTL4	Homo sapiens UDP-N-acetyl-alpha-D-galactosamine:polypeptide N-acetylgalactosaminyltransferase-like 4 (GALNTL4), mRNA [NM_198516]	9,97	6,84	3,13
EGFL7	Homo sapiens EGF-like-domain, multiple 7 (EGFL7), transcript variant 2, mRNA [NM_201446]	10,31	7,2	3,11
COL18A1	Homo sapiens collagen, type XVIII, alpha 1 (COL18A1), transcript variant 1, mRNA [NM_030582]	14,65	11,55	3,11
C1QTNF5	Homo sapiens C1q and tumor necrosis factor related protein 5 (C1QTNF5), mRNA [NM_015645]	10,27	7,15	3,11
TTYH1	Homo sapiens tweety homolog 1 (Drosophila) (TTYH1), transcript variant 1, mRNA [NM_020659]	8,43	5,33	3,1
KIAA1914	Homo sapiens KIAA1914 (KIAA1914), transcript variant 1, mRNA [NM_001001936]	8,97	5,88	3,09

Supplementary Table 1
Morandi et al

FMO1	Homo sapiens flavin containing monooxygenase 1 (FMO1), mRNA [NM_002021]	6,66	3,56	3,09
IGFBP3	Homo sapiens insulin-like growth factor binding protein 3 (IGFBP3), mRNA	11,58	8,52	3,06
ACVRL1	Homo sapiens activin A receptor type II-like 1 (ACVRL1), transcript variant 1, mRNA [NM_000020]	10,93	7,88	3,04
RBP5	Homo sapiens retinol binding protein 5, cellular (RBP5), mRNA [NM_031491]	6,97	3,97	3,01
TGFB3	Homo sapiens transforming growth factor, beta 3 (TGFB3), mRNA [NM_003239]	8,86	5,86	3
C16orf30	Homo sapiens chromosome 16 open reading frame 30 (C16orf30), mRNA [NM_024600]	12,22	9,23	2,99
PLXDC1	Homo sapiens plexin domain containing 1 (PLXDC1), mRNA [NM_020405]	9,95	6,96	2,98
ADAMTS9	Homo sapiens ADAM metallopeptidase with thrombospondin type 1 motif, 9 (ADAMTS9), mRNA [NM_182920]	9,74	6,77	2,98
FLJ36748	Homo sapiens hypothetical protein FLJ36748 (FLJ36748), mRNA [NM_152406]	9,1	6,16	2,94
ENST0000037118[illegible]	Nuclear factor 1 A-type (Nuclear factor 1/A) (NF1-A) (NFI-A) (NF-I/A) (CCAAT-box-binding transcription factor) (CTF) (TGGCA-binding protein), [Source:Uniprot/SWISSPROT;Acc:Q12857] [ENST00000371189]	9,73	6,8	2,93
TENC1	Homo sapiens tensin like C1 domain containing phosphatase (tensin 2) (TENC1), transcript variant 1, mRNA [NM_015319]	8,72	5,8	2,93
CX3CL1	Homo sapiens chemokine (C-X3-C motif) ligand 1 (CX3CL1), mRNA [NM_002996]	7,61	4,69	2,92
CIRBP	Homo sapiens cDNA FLJ46566 fis, clone THYMU3040829, moderately similar to Cold-inducible RNA-binding protein, [AK128423]	10,22	7,3	2,92
BQ897248	AGENCOURT_8122036 Lupski_dorsal_root_ganglion Homo sapiens cDNA clone IMAGE:6179261 5', mRNA sequence [BQ897248]	6,35	3,43	2,91
APLN	Homo sapiens apelin, AGTRL1 ligand (APLN), mRNA [NM_017413]	8,2	5,32	2,89
FILIP1L	Homo sapiens filamin A interacting protein 1-like (FILIP1L), transcript variant 1, mRNA [NM_182909]	8,94	6,06	2,88
DPT	Homo sapiens dermatopontin (DPT), mRNA [NM_001937]	9,35	6,46	2,88
THC2712687	chr11:110468143-110468084	7,07	4,19	2,88
OLFML1	Homo sapiens olfactomedin-like 1 (OLFML1), mRNA [NM_198474]	6,12	3,26	2,86
DKFZP564O0823	Homo sapiens DKFZP564O0823 protein (DKFZP564O0823), mRNA [NM_015393]	8,34	5,48	2,86
AW138903	AW138903 UI-H-BI1-aeq-e-09-0-UI,s1 NCI_CGAP_Sub3 Homo sapiens cDNA clone IMAGE:2720344 3', mRNA sequence [AW138903]	9,45	6,59	2,86
GEFT	Homo sapiens RAC/CDC42 exchange factor (GEFT), transcript variant 1, mRNA [NM_182947]	9,66	6,83	2,83
CLDN5	Homo sapiens claudin 5 (transmembrane protein deleted in velocardiofacial syndrome) (CLDN5), mRNA [NM_003277]	8,82	6,02	2,8
PROCR	Homo sapiens protein C receptor, endothelial (EPCR) (PROCR), mRNA [NM_006404]	9,9	7,1	2,79
TSPAN9	Homo sapiens tetraspanin 9 (TSPAN9), mRNA [NM_006675]	8,15	5,37	2,78
BX097190	BX097190 Soares placenta Nb2HP Homo sapiens cDNA clone IMAGp998G19212, mRNA sequence [BX097190]	12,22	9,43	2,78
TSPAN12	Homo sapiens tetraspanin 12 (TSPAN12), mRNA [NM_012338]	6,57	3,82	2,76
HEPH	Homo sapiens hephaestin (HEPH), transcript variant 2, mRNA [NM_014799]	7,78	5,04	2,74

Supplementary Table 1
Morandi et al

C10orf10	Homo sapiens chromosome 10 open reading frame 10 (C10orf10), mRNA [NM_007021]	11,12	8,39	2,74
FLT1	Homo sapiens fms-related tyrosine kinase 1 (vascular endothelial growth factor/vascular permeability factor receptor) (FLT1), mRNA [NM_002019]	6,75	4,02	2,73
ITGB4	Homo sapiens integrin, beta 4 (ITGB4), transcript variant 1, mRNA [NM_000213]	8,29	5,57	2,72
-	chr8:102266589-102266530	10,34	7,62	2,72
PAPLN	Homo sapiens papilin, proteoglycan-like sulfated glycoprotein (PAPLN), mRNA [NM_173462]	9,52	6,81	2,71
POLR3E	Homo sapiens mRNA for KIAA1452 protein, partial cds, [AB040885]	5,71	3	2,71
HRC	Homo sapiens histidine rich calcium binding protein (HRC), mRNA [NM_002152]	6,53	3,83	2,7
SLCO2B1	Homo sapiens solute carrier organic anion transporter family, member 2B1 (SLCO2B1), mRNA [NM_007256]	10,01	7,32	2,69
RAB6B	Homo sapiens RAB6B, member RAS oncogene family (RAB6B), mRNA [NM_016577]	11,94	9,25	2,69
AL833655	Homo sapiens mRNA; cDNA DKFZp667O0320 (from clone DKFZp667O0320), [AL833655]	7,27	4,58	2,69
DKK3	Homo sapiens dickkopf homolog 3 (Xenopus laevis) (DKK3), transcript variant 1, mRNA [NM_015881]	11,89	9,2	2,69
AK001116	Homo sapiens cDNA FLJ10254 fis, clone HEMBB1000848, [AK001116]	8,58	5,9	2,69
ABLIM3	Homo sapiens actin binding LIM protein family, member 3 (ABLIM3), mRNA [NM_014945]	11,35	8,66	2,69
LAMC3	Homo sapiens laminin, gamma 3 (LAMC3), mRNA [NM_006059]	7,05	4,39	2,67
CCDC48	Homo sapiens coiled-coil domain containing 48 (CCDC48), mRNA [NM_024768]	6,83	4,16	2,67
IHPK3	Homo sapiens inositol hexaphosphate kinase 3 (IHPK3), mRNA [NM_054111]	5,95	3,32	2,64
ITM2C	Homo sapiens integral membrane protein 2C (ITM2C), transcript variant 1, mRNA [NM_030926]	10,92	8,29	2,63
RASIP1	Homo sapiens Ras interacting protein 1 (RASIP1), mRNA [NM_017805]	11	8,38	2,62
ABCG2	Homo sapiens ATP-binding cassette, sub-family G (WHITE), member 2 (ABCG2), mRNA [NM_004827]	7,84	5,22	2,62
TAF5L	Homo sapiens TAF5-like RNA polymerase II, p300/CBP-associated factor (PCAF)-associated factor, 65kDa (TAF5L), transcript variant 1, mRNA [NM_014409]	9,07	6,46	2,61
AF086288	Homo sapiens full length insert cDNA clone ZD48A05, [AF086288]	6,87	4,28	2,6
CLSTN3	Homo sapiens calsyntenin 3 (CLSTN3), mRNA [NM_014718]	8,98	6,38	2,6
HEY1	Homo sapiens hairy/enhancer-of-split related with YRPW motif 1 (HEY1), transcript variant 2, mRNA [NM_001040708]	11,29	8,69	2,59
SEMA5B	Homo sapiens sema domain, seven thrombospondin repeats (type 1 and type 1-like), transmembrane domain (TM) and short cytoplasmic domain, (semaphorin) 5B (SEMA5B), transcript variant 1, mRNA [NM_001031702]	8,99	6,42	2,58
CYYR1	Homo sapiens cysteine/tyrosine-rich 1 (CYYR1), mRNA [NM_052954]	7,47	4,9	2,57
RSPO3	Homo sapiens R-spondin 3 homolog (Xenopus laevis) (RSPO3), mRNA [NM_032784]	6,31	3,76	2,55
HOP	Homo sapiens homeodomain-only protein (HOP), transcript variant 2, mRNA [NM_139211]	8,65	6,1	2,55
AK026826	Homo sapiens cDNA: FLJ23173 fis, clone LNG10019, [AK026826]	7,66	5,11	2,55
-	chr5:134287638-134287579	8,9	6,34	2,55

Supplementary Table 1
Morandi et al

BRD3	Homo sapiens bromodomain containing 3 (BRD3), mRNA [NM_007371]	11,45	8,91	2,54
SLC30A4	Homo sapiens solute carrier family 30 (zinc transporter), member 4 (SLC30A4), mRNA [NM_013309]	6,83	4,31	2,52
RAPGEF3	Homo sapiens Rap guanine nucleotide exchange factor (GEF) 3 (RAPGEF3), mRNA [NM_006105]	6,34	3,82	2,52
CR604283	full-length cDNA clone CS0DF012YB15 of Fetal brain of Homo sapiens (human), [CR604283]	8,83	6,36	2,47
NCALD	Homo sapiens neurocalcin delta (NCALD), transcript variant 7, mRNA [NM_001040630]	8,7	6,24	2,46
PLA2G5	Homo sapiens phospholipase A2, group V (PLA2G5), mRNA [NM_000929]	7,13	4,68	2,45
NFIA	Homo sapiens nuclear factor I/A (NFIA), mRNA [NM_005595]	9,47	7,01	2,45
AZGP1	Homo sapiens alpha-2-glycoprotein 1, zinc-binding (AZGP1), mRNA [NM_001185]	5,91	3,46	2,45
CCDC3	Homo sapiens coiled-coil domain containing 3 (CCDC3), mRNA [NM_031455]	11,56	9,12	2,44
ACP2	Homo sapiens acid phosphatase 2, lysosomal (ACP2), mRNA [NM_001610]	8,56	6,11	2,44
SEPT4	Homo sapiens septin 4 (SEPT4), transcript variant 1, mRNA [NM_004574]	8,82	6,41	2,41
ITGA6	Homo sapiens integrin, alpha 6 (ITGA6), transcript variant 2, mRNA [NM_000210]	9,98	7,58	2,41
CCR3	Homo sapiens chemokine (C-C motif) receptor 3 (CCR3), transcript variant 1, mRNA [NM_001837]	7,46	5,05	2,41
CA314936	UI-CF-FN0-afi-d-17-0-UI,s1 UI-CF-FN0 Homo sapiens cDNA clone UI-CF-FN0-afi-d-17-0-UI 3', mRNA sequence [CA314936]	8,93	6,54	2,4
RASGRP3	Homo sapiens RAS guanyl releasing protein 3 (calcium and DAG-regulated) (RASGRP3), mRNA [NM_170672]	7,38	4,98	2,4
PAQR8	Homo sapiens progestin and adipoQ receptor family member VIII (PAQR8), mRNA [NM_133367]	10,41	8,02	2,4
ZBTB6	Homo sapiens zinc finger and BTB domain containing 6 (ZBTB6), mRNA [NM_006626]	7,55	5,16	2,39
BU561469	AGENCOURT_10278709 NIH_MGC_82 Homo sapiens cDNA clone IMAGE:6592525 5', mRNA sequence [BU561469]	8,38	5,99	2,39
ERG	Homo sapiens v-ets erythroblastosis virus E26 oncogene homolog (avian) (ERG), transcript variant 2, mRNA [NM_004449]	8,33	5,96	2,37
MGC33846	Homo sapiens hypothetical protein MGC33846 (MGC33846), mRNA [NM_175885]	7,69	5,32	2,37
ARHGEF9	Homo sapiens Cdc42 guanine nucleotide exchange factor (GEF) 9 (ARHGEF9), mRNA [NM_015185]	10,13	7,76	2,37
THC2504193	Q15540_HUMAN (Q15540) DCP1 protein , partial (11%) [THC2504193]	8,9	6,55	2,35
PFDN6	Homo sapiens prefoldin subunit 6 (PFDN6), mRNA [NM_014260]	11,88	9,54	2,35
OLFM1	Homo sapiens olfactomedin 1 (OLFM1), transcript variant 1, mRNA [NM_014279]	11,62	9,26	2,35
BC104421	Homo sapiens cDNA clone IMAGE:40004940, [BC104421]	6,87	4,53	2,35
CYP27A1	Homo sapiens cytochrome P450, family 27, subfamily A, polypeptide 1 (CYP27A1), nuclear gene encoding mitochondrial protein, mRNA [NM_000784]	7,98	5,63	2,34
ADAM33	Homo sapiens ADAM metallopeptidase domain 33 (ADAM33), transcript variant 1, mRNA [NM_025220]	7,61	5,27	2,34
COX4I2	Homo sapiens cytochrome c oxidase subunit IV isoform 2 (lung) (COX4I2), nuclear gene encoding mitochondrial protein, mRNA [NM_032609]	7,15	4,85	2,3
ADAMTSL2	Homo sapiens ADAMTS-like 2 (ADAMTSL2), mRNA [NM_014694]	8,84	6,53	2,3

Supplementary Table 1
Morandi et al

ANGPTL2	Homo sapiens angiopoietin-like 2 (ANGPTL2), mRNA [NM_012098]	8,18	5,9	2,28
LPPR4	Homo sapiens plasticity related gene 1 (LPPR4), mRNA [NM_014839]	11,58	9,31	2,27
GUCY1B3	Homo sapiens guanylate cyclase 1, soluble, beta 3 (GUCY1B3), mRNA [NM_000857]	8,85	6,58	2,27
MRGPRE	Homo sapiens MAS-related GPR, member E (MRGPRE), mRNA [NM_001039165]	6,08	3,82	2,26
GPR162	Homo sapiens G protein-coupled receptor 162 (GPR162), transcript variant A-2, mRNA [NM_019858]	9,22	6,96	2,26
C20orf112	Uncharacterized protein C20orf112, [Source:Uniprot/SWISSPROT;Acc:Q96MY1] [ENST00000359676]	8,42	6,17	2,25
AK074696	Homo sapiens cDNA FLJ90215 fis, clone MAMMA1002205, [AK074696]	8,34	6,1	2,24
ENST0000038349(	chr18:5881958-5881899	6,43	4,18	2,24
TDRD10	Homo sapiens tudor domain containing 10 (TDRD10), mRNA [NM_182499]	6,19	3,95	2,23
IGF2AS	Homo sapiens insulin-like growth factor 2 antisense (IGF2AS), mRNA [NM_016412]	7,94	5,72	2,23
PLLP	Homo sapiens transmembrane 4 superfamily member 11 (plasmolipin) (TM4SF11), mRNA [NM_015993]	6,66	4,44	2,22
SLC39A7	Homo sapiens solute carrier family 39 (zinc transporter), member 7 (SLC39A7), transcript variant 1, mRNA [NM_006979]	11,2	8,99	2,21
NOS3	Homo sapiens nitric oxide synthase 3 (endothelial cell) (NOS3), mRNA [NM_000603]	7,56	5,36	2,21
CR626252	full-length cDNA clone CS0DD001YO10 of Neuroblastoma Cot 50-normalized of Homo sapiens (human), [CR626252]	7,51	5,3	2,21
GPRC5C	Homo sapiens G protein-coupled receptor, family C, group 5, member C (GPRC5C), transcript variant 1, mRNA [NM_022036]	6,2	4	2,2
CCDC71	Homo sapiens coiled-coil domain containing 71 (CCDC71), mRNA [NM_022903]	8,27	6,07	2,2
SYTL2	Homo sapiens synaptotagmin-like 2 (SYTL2), transcript variant c, mRNA [NM_206927]	7,56	5,37	2,19
GGTL3	Homo sapiens gamma-glutamyltransferase-like 3 (GGTL3), mRNA [NM_178026]	9,41	7,22	2,19
-	chr17:022320734-022320784	7,24	5,05	2,19
RALGDS	Homo sapiens ral guanine nucleotide dissociation stimulator (RALGDS), transcript variant 2, mRNA [NM_001042368]	10,67	8,49	2,18
THC2633081	chr15:28503020-28502961	8,06	5,87	2,18
RASL12	Homo sapiens RAS-like, family 12 (RASL12), mRNA [NM_016563]	6,97	4,8	2,17
EMID1	Homo sapiens EMI domain containing 1 (EMID1), mRNA [NM_133455]	6,64	4,47	2,17
ATM	Homo sapiens ataxia telangiectasia mutated (includes complementation groups A, C and D) (ATM), transcript variant 1, mRNA [NM_000051]	7,14	4,97	2,17
TNFSF4	Homo sapiens tumor necrosis factor (ligand) superfamily, member 4 (tax-transcriptionally activated glycoprotein 1, 34kDa) (TNFSF4), mRNA [NM_003326]	6,61	4,46	2,15
COLEC11	Homo sapiens collectin sub-family member 11 (COLEC11), transcript variant 2, mRNA [NM_199235]	10,99	8,84	2,15
ATP2B4	Homo sapiens ATPase, Ca++ transporting, plasma membrane 4 (ATP2B4), transcript variant 1, mRNA [NM_001001396]	12,19	10,03	2,15
TNFRSF4	Homo sapiens tumor necrosis factor receptor superfamily, member 4 (TNFRSF4), mRNA [NM_003327]	7,44	5,3	2,14
PROS1	Homo sapiens protein S (alpha) (PROS1), mRNA [NM_000313]	8,82	6,68	2,14

Supplementary Table 1
Morandi et al

HSPA12B	Homo sapiens heat shock 70kD protein 12B (HSPA12B), mRNA [NM_052970]	9,2	7,08	2,13
THC2671230	GCU51450 IgNARC {Ginglymostoma cirratum} (exp=-1; wgp=0; cg=0), partial (3%) [THC2671230]	8,34	6,21	2,13
ZNF264	Homo sapiens zinc finger protein 264 (ZNF264), mRNA [NM_003417]	9,05	6,92	2,12
LOC157562	Homo sapiens mRNA; cDNA DKFZp566J123 (from clone DKFZp566J123), [AL050061]	10,31	8,19	2,12
SEMA4C	Homo sapiens sema domain, immunoglobulin domain (Ig), transmembrane domain (TM) and short cytoplasmic domain, (semaphorin) 4C (SEMA4C), mRNA [NM_017789]	8,74	6,63	2,11
ZFYVE9	Homo sapiens zinc finger, FYVE domain containing 9 (ZFYVE9), transcript variant 3, mRNA [NM_004799]	10,54	8,45	2,1
FZR1	Homo sapiens fizzy/cell division cycle 20 related 1 (Drosophila) (FZR1), mRNA [NM_016263]	9,03	6,93	2,1
AK021467	Homo sapiens cDNA FLJ11405 fis, clone HEMBA1000769, [AK021467]	7,13	5,02	2,1
KIAA1183	Homo sapiens KIAA1183 protein, mRNA (cDNA clone IMAGE:40108157), partial cds, [BC121810]	7,98	5,89	2,09
AK021980	Homo sapiens cDNA FLJ11918 fis, clone HEMBB1000272, [AK021980]	13,19	11,1	2,09
PLEKHG1	Homo sapiens pleckstrin homology domain containing, family G (with RhoGef domain) member 1 (PLEKHG1), mRNA [NM_001029884]	6,72	4,63	2,08
THC2500892	ALU1_HUMAN (P39188) Alu subfamily J sequence contamination warning entry, partial (12%) [THC2500892]	6,93	4,86	2,08
RGAG4	Homo sapiens retrotransposon gag domain containing 4 (RGAG4), mRNA [NM_001024455]	10,02	7,96	2,06
C22orf9	Homo sapiens chromosome 22 open reading frame 9 (C22orf9), transcript variant 1, mRNA [NM_015264]	9,4	7,34	2,06
TTC17	Homo sapiens tetratricopeptide repeat domain 17, mRNA (cDNA clone IMAGE:5298645), complete cds, [BC041893]	10,03	7,98	2,05
AK124281	Homo sapiens cDNA FLJ42287 fis, clone TLIVE2005866, [AK124281]	6,4	4,35	2,05
HSMPP8	Homo sapiens M-phase phosphoprotein, mpp8 (HSMPP8), mRNA [NM_017520]	8,98	6,94	2,04
CHST2	Homo sapiens carbohydrate (N-acetylglucosamine-6-O) sulfotransferase 2 (CHST2), mRNA [NM_004267]	8,62	6,58	2,04
TOM1L1	full-length cDNA clone CS0DD005YN08 of Neuroblastoma Cot 50-normalized of Homo sapiens (human), [CR593166]	7,31	5,27	2,04
AK093729	Homo sapiens cDNA FLJ36410 fis, clone THYMU2010637, [AK093729]	11,82	9,79	2,03
AK023338	Homo sapiens cDNA FLJ13276 fis, clone OVARC1001040, [AK023338]	7,46	5,44	2,02
FLJ38984	Homo sapiens hypothetical protein FLJ38984 (FLJ38984), mRNA [NM_152374]	8,8	6,79	2,01
GPRC5B	Homo sapiens G protein-coupled receptor, family C, group 5, member B (GPRC5B), mRNA [NM_016235]	8,54	6,54	2
THC2686967	chr17:69863473-69863532	6,15	4,14	2
KRTAP6-3	Homo sapiens keratin associated protein 6-3 (KRTAP6-3), mRNA [NM_181605]	6,8	4,81	1,99
CPEB3	Homo sapiens cytoplasmic polyadenylation element binding protein 3 (CPEB3), mRNA [NM_014912]	8,35	6,36	1,99
C1QTNF1	Homo sapiens C1q and tumor necrosis factor related protein 1 (C1QTNF1), mRNA [NM_198594]	6,43	4,44	1,99
ZNF282	Homo sapiens zinc finger protein 282 (ZNF282), mRNA [NM_003575]	7,58	5,6	1,98
SLC22A17	Homo sapiens solute carrier family 22 (organic cation transporter), member 17 (SLC22A17), transcript variant 2, mRNA [NM_016609]	11,01	9,03	1,98
MCAM	Homo sapiens melanoma cell adhesion molecule (MCAM), mRNA [NM_006500]	11,95	9,97	1,98

Supplementary Table 1
Morandi et al

NR1D2	Homo sapiens mRNA; cDNA DKFZp779H1259 (from clone DKFZp779H1259), [BX537786]	8,08	6,12	1,96
APP	Homo sapiens amyloid beta (A4) precursor protein (peptidase nexin-II, Alzheimer disease) (APP), transcript variant 1, mRNA [NM_000484]	10,62	8,66	1,96
-	chrX:018925300-018925252	6,55	4,59	1,96
LZTS1	Homo sapiens leucine zipper, putative tumor suppressor 1 (LZTS1), mRNA [NM_021020]	12,07	10,12	1,95
AK027610	Homo sapiens cDNA FLJ14704 fis, clone NT2RP3000526, [AK027610]	6,72	4,78	1,95
CD47	Homo sapiens CD47 molecule (CD47), transcript variant 1, mRNA [NM_001777]	8,9	6,95	1,95
TTC33	Homo sapiens tetratricopeptide repeat domain 33 (TTC33), mRNA [NM_012382]	6,92	4,98	1,94
DNAJB2	Homo sapiens DnaJ (Hsp40) homolog, subfamily B, member 2 (DNAJB2), transcript variant 1, mRNA [NM_001039550]	9,27	7,34	1,93
THC2709260	chr2:152361700-152361641	9,7	7,77	1,93
BF969441	602271732F1 NIH_MGC_84 Homo sapiens cDNA clone IMAGE:4360158 5', mRNA sequence [BF969441]	7,19	5,26	1,93
BG193790	RST12926 Athersys RAGE Library Homo sapiens cDNA, mRNA sequence [BG193790]	5,74	3,83	1,92
BTBD3	Homo sapiens BTB (POZ) domain containing 3 (BTBD3), transcript variant 1, mRNA [NM_014962]	11,45	9,53	1,92
THC2718406	chr1:176819413-176819354	6,19	4,27	1,92
EXOC3L	Homo sapiens exocyst complex component 3-like (EXOC3L), mRNA [NM_178516]	7,94	6,04	1,89
DTNB	Homo sapiens dystrobrevin, beta (DTNB), transcript variant 3, mRNA [NM_033148]	6,98	5,09	1,89
ENST0000032757	Pyridoxal kinase (EC 2,7,1,35) (Pyridoxine kinase), [Source:Uniprot/SWISSPROT;Acc:O00764] [ENST00000327574]	5,71	3,83	1,88
PPP1R12B	Homo sapiens protein phosphatase 1, regulatory (inhibitor) subunit 12B (PPP1R12B), transcript variant 2, mRNA [NM_032105]	11,85	9,96	1,88
COX6B2	Homo sapiens cytochrome c oxidase subunit VIb polypeptide 2 (testis) (COX6B2), mRNA [NM_144613]	6,82	4,94	1,88
KRT84	Keratin, type II cuticular Hb4 (Hair keratin, type II Hb4) (Keratin- 84), [Source:Uniprot/SWISSPROT;Acc:Q9NSB2] [ENST00000257951]	6,79	4,92	1,87
IFNA4	Homo sapiens interferon, alpha 4 (IFNA4), mRNA [NM_021068]	5,95	4,09	1,87
GPLD1	Homo sapiens glycosylphosphatidylinositol specific phospholipase D1 (GPLD1), transcript variant 1, mRNA [NM_001503]	6,35	4,48	1,87
ZNF337	Homo sapiens zinc finger protein 337 (ZNF337), mRNA [NM_015655]	9,54	7,68	1,86
OPN1LW	Homo sapiens opsin 1 (cone pigments), long-wave-sensitive (color blindness, protan) (OPN1LW), mRNA [NM_020061]	6,32	4,46	1,86
HSU79275	Human clone 23947 mRNA, partial cds, [U79275]	7,46	5,61	1,85
RAD23B	Homo sapiens RAD23 homolog B (S, cerevisiae) (RAD23B), mRNA [NM_002874]	7,53	5,68	1,85
MFHAS1	Homo sapiens malignant fibrous histiocytoma amplified sequence 1 (MFHAS1), mRNA [NM_004225]	7,1	5,25	1,85
AK021933	Homo sapiens cDNA FLJ11871 fis, clone HEMBA1007052, [AK021933]	5,97	4,12	1,85
TMEM19	Homo sapiens transmembrane protein 19 (TMEM19), mRNA [NM_018279]	6,17	4,33	1,84
OR5H1	Homo sapiens olfactory receptor, family 5, subfamily H, member 1 (OR5H1), mRNA [NM_001005338]	6,25	4,41	1,84
KRTAP13-4	Homo sapiens keratin associated protein 13-4 (KRTAP13-4), mRNA [NM_181600]	5,92	4,08	1,84

HDAC11	Homo sapiens histone deacetylase 11 (HDAC11), mRNA [NM_024827]	7,07	5,24	1,83
ZNF652	Homo sapiens zinc finger protein 652 (ZNF652), mRNA [NM_014897]	9,5	7,68	1,82
PTPN3	Homo sapiens protein tyrosine phosphatase, non-receptor type 3 (PTPN3), mRNA [NM_002829]	8,41	6,59	1,82
CALM1	Homo sapiens calmodulin 1 (phosphorylase kinase, delta) (CALM1), mRNA [NM_006888]	11,95	10,13	1,82
BSPRY	Homo sapiens B-box and SPRY domain containing (BSPRY), mRNA [NM_017688]	6,53	4,71	1,82
AI682237	AI682237 wa71f04,x1 Soares_NFL_T_GBC_S1 Homo sapiens cDNA clone IMAGE:2301631 3', mRNA sequence [AI682237]	5,96	4,15	1,82
HIVEP2	Homo sapiens human immunodeficiency virus type I enhancer binding protein 2 (HIVEP2), mRNA [NM_006734]	12,17	10,36	1,81
PTCH2	Homo sapiens patched homolog 2 (Drosophila) (PTCH2), mRNA [NM_003738]	6,47	4,68	1,79
HSA251708	Homo sapiens partial mRNA for putative microtubule-binding protein, [AJ251708]	5,97	4,18	1,79
KIAA0664	Homo sapiens KIAA0664 (KIAA0664), mRNA [NM_015229]	9,89	8,09	1,79
ITGA1	Homo sapiens integrin, alpha 1 (ITGA1), mRNA [NM_181501]	9,52	7,73	1,79
CD2BP2	Homo sapiens mRNA for KIAA1178 protein, partial cds, [AB033004]	11,62	9,84	1,78
C11orf72	Homo sapiens chromosome 11 open reading frame 72 (C11orf72), mRNA [NM_173578]	5,78	4	1,78
KIAA1155	Homo sapiens cDNA FLJ37016 fis, clone BRACE2010632, [AK094335]	7,74	5,96	1,78
-	chr21:031918525-031918466	11,04	9,26	1,78
OPN4	Homo sapiens opsin 4 (melanopsin) (OPN4), transcript variant 2, mRNA [NM_001030015]	5,87	4,1	1,77
IL17RE	Homo sapiens interleukin 17 receptor E (IL17RE), transcript variant 5, mRNA [NM_153483]	7,4	5,63	1,77
PANK2	Homo sapiens mRNA; cDNA DKFZp547J0513 (from clone DKFZp547J0513), [AL713654]	6,18	4,42	1,76
NOPE	Homo sapiens likely ortholog of mouse neighbor of Punc E11 (NOPE), mRNA [NM_020962]	5,5	3,73	1,76
-	chr9:035845887-035845946	8,39	6,63	1,76
ENST0000029941[illegible]	Q7D724_MYCTU (Q7D724) PE_PGRS family protein (PE-PGRS FAMILY PROTEIN), partial (3%) [THC2672762]	5,65	3,9	1,75
TAAR2	Homo sapiens trace amine associated receptor 2 (TAAR2), transcript variant 1, mRNA [NM_001033080]	5,77	4,03	1,75
PDAP1	Homo sapiens PDGFA associated protein 1 (PDAP1), mRNA [NM_014891]	11,01	9,26	1,75
KLK2	Homo sapiens kallikrein-related peptidase 2 (KLK2), transcript variant 1, mRNA [NM_005551]	5,84	4,09	1,75
BM703463	UI-E-CL1-afe-e-20-0-UI,r1 UI-E-CL1 Homo sapiens cDNA clone UI-E-CL1-afe-e-20-0-UI 5', mRNA sequence [BM703463]	6,38	4,64	1,74
ZFP95	Homo sapiens zinc finger protein 95 homolog (mouse) (ZFP95), transcript variant 1, mRNA [NM_014569]	8,6	6,86	1,74
THC2531058	Q47K95_THEFY (Q47K95) Single-strand binding protein, partial (6%) [THC2531058]	7,02	5,29	1,73
SEMA4F	Homo sapiens sema domain, immunoglobulin domain (Ig), transmembrane domain (TM) and short cytoplasmic domain, (semaphorin) 4F (SEMA4F), mRNA [NM_004263]	9,77	8,04	1,73
PPAP2A	Homo sapiens phosphatidic acid phosphatase type 2A (PPAP2A), transcript variant 2, mRNA [NM_176895]	11,63	9,9	1,73

Supplementary Table 1
Morandi et al

KIF3C	Homo sapiens kinesin family member 3C (KIF3C), mRNA [NM_002254]	8,55	6,82	1,73
C1orf90	Homo sapiens chromosome 1 open reading frame 90 (C1orf90), mRNA [NM_032648]	8,22	6,49	1,73
-	chr20:058977227-058977286	6,18	4,44	1,73
KIAA0265	Human mRNA for KIAA0265 gene, partial cds, [D87454]	10,03	8,31	1,72
M69012	Human guanine nucleotide-binding regulatory protein (G-y-2-alpha) mRNA, partial cds, [M69012]	8,18	6,47	1,72
TINAGL1	Homo sapiens tubulointerstitial nephritis antigen-like 1 (TINAGL1), mRNA [NM_022164]	5,88	4,16	1,72
HNRPAB	Homo sapiens heterogeneous nuclear ribonucleoprotein A/B (HNRPAB), transcript variant 2, mRNA [NM_004499]	10,33	8,61	1,72
-	chr11:113048984-113049043	5,98	4,26	1,72
UGT2B10	Homo sapiens UDP glucuronosyltransferase 2 family, polypeptide B10 (UGT2B10), mRNA [NM_001075]	6,67	4,96	1,7
FAM70B	Homo sapiens family with sequence similarity 70, member B (FAM70B), mRNA [NM_182614]	7	5,3	1,7
AK022252	Homo sapiens cDNA FLJ12190 fis, clone MAMMA1000842, [AK022252]	7,25	5,55	1,7
-	chr8:008049184-008049125	7,8	6,1	1,7
-	chr7:022653000-022652941	6,9	5,19	1,7
PRRT1	Homo sapiens proline-rich transmembrane protein 1 (PRRT1), mRNA [NM_030651]	6,51	4,82	1,69
NOTCH4	Homo sapiens Notch homolog 4 (Drosophila) (NOTCH4), mRNA [NM_004557]	7,52	5,83	1,69
DRD2	Homo sapiens dopamine receptor D2 (DRD2), transcript variant 1, mRNA [NM_000795]	6,58	4,89	1,69
HSD17B1	Homo sapiens hydroxysteroid (17-beta) dehydrogenase 1 (HSD17B1), mRNA [NM_000413]	7,46	5,78	1,68
-	chr18:073390284-073390343	6,18	4,5	1,68
NAP1L4	Homo sapiens nucleosome assembly protein 1-like 4 (NAP1L4), mRNA [NM_005969]	10,75	9,09	1,67
MOBKL2C	Homo sapiens MOB1, Mps One Binder kinase activator-like 2C (yeast) (MOBKL2C), transcript variant 1, mRNA [NM_145279]	8,87	7,2	1,67
MAGED4	Homo sapiens melanoma antigen family D, 4 (MAGED4), transcript variant 1, mRNA [NM_030801]	13,39	11,72	1,67
GBA2	Homo sapiens glucosidase, beta (bile acid) 2 (GBA2), mRNA [NM_020944]	8,92	7,25	1,67
AF086527	Homo sapiens full length insert cDNA clone ZE05A03, [AF086527]	5,88	4,2	1,67
CBX7	Homo sapiens chromobox homolog 7 (CBX7), mRNA [NM_175709]	10,83	9,16	1,67
FLJ12993	Homo sapiens cDNA FLJ12993 fis, clone NT2RP3000197, [AK023055]	7	5,33	1,67
ADCK2	Homo sapiens aarF domain containing kinase 2 (ADCK2), mRNA [NM_052853]	8,55	6,88	1,67
IL9R	Homo sapiens interleukin 9 receptor (IL9R), transcript variant 2, mRNA [NM_176786]	6,05	4,39	1,66
SUOX	Homo sapiens sulfite oxidase (SUOX), nuclear gene encoding mitochondrial protein, transcript variant 1, mRNA [NM_000456]	8,65	7	1,65
RER1	Homo sapiens RER1 retention in endoplasmic reticulum 1 homolog (S, cerevisiae) (RER1), mRNA [NM_007033]	7,88	6,23	1,65
LOC654780	Homo sapiens cDNA FLJ38012 fis, clone CTONG2012554, [AK095331]	6,2	4,55	1,65
BICD1	Homo sapiens bicaudal D homolog 1 (Drosophila) (BICD1), transcript variant 2, mRNA [NM_001003398]	8,71	7,05	1,65
STK32C	Homo sapiens serine/threonine kinase 32C (STK32C), mRNA [NM_173575]	10,73	9,09	1,64
GPR161	Homo sapiens G protein-coupled receptor 161 (GPR161), transcript variant 2, mRNA [NM_153832]	9,75	8,11	1,64

Supplementary Table 1
Morandi et al

ACAD10	Homo sapiens acyl-Coenzyme A dehydrogenase family, member 10 (ACAD10), mRNA [NM_025247]	8,17	6,53	1,64
SLC45A1	Proton-associated sugar transporter A (PAST-A) (Solute carrier family 45 member 1) (Deleted in neuroblastoma 5 protein) (DNb-5), [Source:Uniprot/SWISSPROT;Acc:Q9Y2W3] [ENST00000377479]	6,03	4,4	1,63
ST3GAL4	Homo sapiens ST3 beta-galactoside alpha-2,3-sialyltransferase 4 (ST3GAL4), mRNA [NM_006278]	8,45	6,82	1,63
GPR146	Homo sapiens G protein-coupled receptor 146 (GPR146), mRNA [NM_138445]	8,2	6,57	1,63
APOLD1	Homo sapiens apolipoprotein L domain containing 1 (APOLD1), mRNA [NM_030817]	10,72	9,09	1,63
AY010113	Homo sapiens unknown mRNA sequence, [AY010113]	5,89	4,27	1,62
PACSIN2	Homo sapiens protein kinase C and casein kinase substrate in neurons 2 (PACSIN2), mRNA [NM_007229]	10,13	8,51	1,62
PNMA2	Homo sapiens paraneoplastic antigen MA2 (PNMA2), mRNA [NM_007257]	11,34	9,72	1,62
MUC3A	Homo sapiens mucin (MUC-3) mRNA, partial cds, [M55405]	6,79	5,16	1,62
THC2693398	ALU1_HUMAN (P39188) Alu subfamily J sequence contamination warning entry, partial (11%) [THC2693398]	7,83	6,21	1,62
THC2643320	Q306F7_HUMAN (Q306F7) Down syndrome encephalopathy related protein 1, partial (11%) [THC2643320]	9,4	7,79	1,61
MYOG	Homo sapiens myogenin (myogenic factor 4) (MYOG), mRNA [NM_002479]	5,96	4,35	1,61
ENST00000381929	Homo sapiens mucin 4 (MUC4) mRNA, partial cds, [AF177925]	6,24	4,63	1,61
KIAA0738	Homo sapiens KIAA0738 gene product (KIAA0738), mRNA [NM_014719]	10,63	9,02	1,61
ZSCAN1	Homo sapiens zinc finger and SCAN domain containing 1 (ZSCAN1), mRNA [NM_182572]	5,67	4,07	1,6
STARD9	StAR-related lipid transfer protein 9 (StARD9) (START domain- containing protein 9) (Fragment), [Source:Uniprot/SWISSPROT;Acc:Q9P2P6] [ENST00000290607]	7,26	5,67	1,59
ZNF182	Homo sapiens zinc finger protein 182 (ZNF182), transcript variant 1, mRNA [NM_006962]	10,01	8,43	1,59
DCAMKL2	Homo sapiens doublecortin and CaM kinase-like 2 (DCAMKL2), transcript variant 1, mRNA [NM_001040260]	9,74	8,15	1,59
DOCK9	Homo sapiens dedicator of cytokinesis 9 (DOCK9), mRNA [NM_015296]	8,87	7,28	1,59
ZFP90	Homo sapiens zinc finger protein 90 homolog (mouse) (ZFP90), mRNA [NM_133458]	8	6,42	1,58
PPP1R3F	Homo sapiens protein phosphatase 1, regulatory (inhibitor) subunit 3F (PPP1R3F), mRNA [NM_033215]	11,3	9,72	1,58
NPFFR1	Homo sapiens neuropeptide FF receptor 1 (NPFFR1), mRNA [NM_022146]	5,42	3,84	1,58
C6orf89	Homo sapiens chromosome 6 open reading frame 89 (C6orf89), mRNA [NM_152734]	9,55	7,97	1,58
AK129982	Homo sapiens cDNA FLJ26472 fis, clone KDN04506, [AK129982]	7,91	6,32	1,58
TSPAN1	Homo sapiens tetraspanin 1 (TSPAN1), mRNA [NM_005727]	6,99	5,42	1,57
LOC401010	Homo sapiens hypothetical LOC401010 (LOC401010) on chromosome 2 [NR_002826]	6,43	4,86	1,57
SLC44A1	Choline transporter-like protein 1 (Solute carrier family 44 member 1) (CD92 antigen) (CDw92), [Source:Uniprot/SWISSPROT;Acc:Q8WWI5] [ENST00000361080]	9,35	7,78	1,57

Supplementary Table 1
Morandi et al

THC2557626	ALU1_HUMAN (P39188) Alu subfamily J sequence contamination warning entry, partial (14%) [THC2557626]	5,64	4,07	1,57
SAP18	Homo sapiens Sin3A-associated protein, 18kDa (SAP18), mRNA [NM_005870]	9,67	8,11	1,56
THC2614136	chr7:104530798-104530739	7,12	5,56	1,56
AIRE	Homo sapiens autoimmune regulator (autoimmune polyendocrinopathy candidiasis ectodermal dystrophy) (AIRE), transcript variant AIRE-2, mRNA [NM_000658]	5,74	4,19	1,55
-	chr8:000735716-000735657	7,96	6,4	1,55
THC2593103	Q6NT14_HUMAN (Q6NT14) ZNF80 protein (Fragment), partial (6%) [THC2593103]	8,53	6,99	1,54
ENST0000035837[illegible]	Mucin-5B precursor (Mucin-5 subtype B, tracheobronchial) (High molecular weight salivary mucin MG1) (Sublingual gland mucin), [Source:Uniprot/SWISSPROT;Acc:Q9HC84] [ENST00000358378]	5,84	4,32	1,53
PLG	Homo sapiens plasminogen (PLG), mRNA [NM_000301]	6,46	4,93	1,53
CHRNB2	Homo sapiens cholinergic receptor, nicotinic, beta 2 (neuronal) (CHRNB2), mRNA [NM_000748]	6,98	5,45	1,53
SFRS16	Homo sapiens cDNA FLJ90109 fis, clone HEMBA1006544, [AK074590]	8	6,47	1,53
ENST0000037867[illegible]	apical protein 2 [Source:RefSeq_peptide;Acc:NP_597713] [ENST00000378679]	7,68	6,15	1,53
RXRB	Homo sapiens retinoid X receptor, beta (RXRB), mRNA [NM_021976]	10,09	8,57	1,52
GPR37L1	Homo sapiens G protein-coupled receptor 37 like 1 (GPR37L1), mRNA [NM_004767]	5,78	4,26	1,52
CLCN1	Homo sapiens chloride channel 1, skeletal muscle (Thomsen disease, autosomal dominant) (CLCN1), mRNA [NM_000083]	5,86	4,34	1,52
-	chr9:130434933-130434990	10,45	8,93	1,52
AL080233	Homo sapiens mRNA; cDNA DKFZp586L111 (from clone DKFZp586L111), [AL080233]	5,68	4,17	1,51
CR609588	full-length cDNA clone CS0DD009YL11 of Neuroblastoma Cot 50-normalized of Homo sapiens (human), [CR609588]	5,61	4,1	1,51
ENST0000035673[illegible]	chr8:144861173-144861114	5,64	4,13	1,51
AI915259	AI915259 tx06b08,x1 NCI_CGAP_Ut4 Homo sapiens cDNA clone IMAGE:2268375 3' similar to gb:J03278 BETA PLATELET-DERIVED GROWTH FACTOR RECEPTOR PRECURSOR (HUMAN);, mRNA sequence [AI915259]	6,33	4,81	1,51
PTK2	Homo sapiens PTK2 protein tyrosine kinase 2 (PTK2), transcript variant 1, mRNA [NM_153831]	9,63	8,13	1,5
KIAA0152	Homo sapiens KIAA0152 (KIAA0152), mRNA [NM_014730]	8,52	7,02	1,5
AKT3	RAC-gamma serine/threonine-protein kinase (EC 2,7,11,1) (RAC-PK-gamma) (Protein kinase Akt-3) (Protein kinase B, gamma) (PKB gamma) (STK-2), [Source:Uniprot/SWISSPROT;Acc:Q9Y243] [ENST00000366539]	10,84	9,36	1,49
BX648950	Homo sapiens mRNA; cDNA DKFZp686E1648 (from clone DKFZp686E1648), [BX648950]	11,09	9,6	1,49
POLE	Homo sapiens DNA polymerase epsilon catalytic subunit isoform a (POLE1) mRNA, partial cds, [AF128541]	5,95	4,46	1,49
THC2647765	chr10:105212189-105212248	6,03	4,54	1,49
PTP4A3	Homo sapiens protein tyrosine phosphatase type IVA, member 3 (PTP4A3), transcript variant 1, mRNA [NM_032611]	8,33	6,85	1,48
USP31	Homo sapiens mRNA for KIAA1203 protein, partial cds, [AB033029]	9,4	7,92	1,48

Supplementary Table 1
Morandi et al

ACADS	Homo sapiens acyl-Coenzyme A dehydrogenase, C-2 to C-3 short chain (ACADS), nuclear gene encoding mitochondrial protein, mRNA [NM_000017]	7,67	6,19	1,48
THC2732966	chrX:071478680-071478739	5,96	4,47	1,48
CR992331	CR992331 RZPD no,9016 Homo sapiens cDNA clone RZPDp9016A0141 5', mRNA sequence [CR992331]	7,07	5,6	1,47
DKFZP564C196	Homo sapiens mRNA; cDNA DKFZp564C196 (from clone DKFZp564C196), [AL050020]	6,23	4,77	1,46
PRIMA1	Homo sapiens proline rich membrane anchor 1 (PRIMA1), mRNA [NM_178013]	7,48	6,04	1,44
CNDP2	Homo sapiens CNDP dipeptidase 2 (metallopeptidase M20 family) (CNDP2), mRNA [NM_018235]	10,57	9,13	1,44
SF3B3	Homo sapiens splicing factor 3b, subunit 3, 130kDa (SF3B3), mRNA [NM_012426]	10,89	9,46	1,43
KRTAP2-1	Homo sapiens partial mRNA for keratin associated protein (KRTAP2,1A gene), [AJ296345]	6,34	4,91	1,43
KIAA0329	Homo sapiens KIAA0329 (KIAA0329), mRNA [NM_014844]	10,05	8,62	1,43
KIAA0082	Homo sapiens KIAA0082 (KIAA0082), mRNA [NM_015050]	10,74	9,31	1,43
HISPPD2A	Homo sapiens histidine acid phosphatase domain containing 2A (HISPPD2A), transcript variant 4, mRNA [NM_001024463]	8,7	7,27	1,43
AK057015	Homo sapiens cDNA FLJ32453 fis, clone SKMUS2001703, [AK057015]	9,04	7,61	1,43
ENST0000037367	Membrane-associated nucleic acid-binding protein (RING finger protein 164), [Source:Uniprot/SWISSPROT;Acc:Q9HBD1] [ENST00000373670]	10,46	9,04	1,42
SMARCD2	Homo sapiens SWI/SNF related, matrix associated, actin dependent regulator of chromatin, subfamily d, member 2 (SMARCD2), mRNA [NM_003077]	9,86	8,45	1,41
NDUFC2	Homo sapiens NADH dehydrogenase (ubiquinone) 1, subcomplex unknown, 2, 14,5kDa (NDUFC2), mRNA [NM_004549]	9,01	7,6	1,41
AK090442	Homo sapiens mRNA for FLJ00363 protein, [AK090442]	5,96	4,55	1,41
JRK	Homo sapiens jerky homolog (mouse) (JRK), transcript variant 1, mRNA [NM_003724]	7,94	6,53	1,41
GJA12	Homo sapiens gap junction protein, alpha 12, 47kDa (GJA12), mRNA [NM_020435]	9,26	7,85	1,41
AK091784	Homo sapiens cDNA FLJ34465 fis, clone HLUNG2003061, [AK091784]	7,61	6,19	1,41
CR619805	full-length cDNA clone CS0DI022YH23 of Placenta Cot 25-normalized of Homo sapiens (human), [CR619805]	6,75	5,35	1,41
BX362821	BX362821 BX362821 Homo sapiens HELA CELLS COT 25-NORMALIZED Homo sapiens cDNA clone CS0DK004YJ19 3-PRIME, mRNA sequence [BX362821]	6,33	4,92	1,41
TNFAIP8L1	Homo sapiens tumor necrosis factor, alpha-induced protein 8-like 1 (TNFAIP8L1), mRNA [NM_152362]	6,64	5,24	1,4
MADCAM1	Homo sapiens mucosal vascular addressin cell adhesion molecule 1 (MADCAM1), transcript variant 1, mRNA [NM_130760]	6,38	4,98	1,4
KIAA0913	full-length cDNA clone CS0DI059YG13 of Placenta Cot 25-normalized of Homo sapiens (human), [CR610954]	6,82	5,42	1,4
CR610885	full-length cDNA clone CS0DC019YC18 of Neuroblastoma Cot 25-normalized of Homo sapiens (human), [CR610885]	8,83	7,43	1,4
THC2761218	CN167_HUMAN (Q9P1J3) Protein C14orf167, partial (18%) [THC2761218]	5,72	4,32	1,4
THC2729899	chr4:83623649-83623590	10,14	8,74	1,4
PHC1	Homo sapiens polyhomeotic homolog 1 (Drosophila) (PHC1), mRNA [NM_004426]	8,38	6,98	1,39

Supplementary Table 1
Morandi et al

TEX2	Homo sapiens testis expressed sequence 2 (TEX2), mRNA [NM_018469]	11,74	10,36	1,38
KCTD2	Homo sapiens potassium channel tetramerisation domain containing 2 (KCTD2), mRNA [NM_015353]	11,44	10,06	1,38
FBXW4	Homo sapiens F-box and WD-40 domain protein 4 (FBXW4), mRNA [NM_022039]	8,81	7,44	1,38
CELSR2	Homo sapiens cadherin, EGF LAG seven-pass G-type receptor 2 (flamingo homolog, Drosophila) (CELSR2), mRNA [NM_001408]	6,63	5,25	1,38
-	chr15_random:000081180-000081237	5,86	4,48	1,38
C14orf152	Homo sapiens chromosome 14 open reading frame 152 (C14orf152), mRNA [NM_138344]	6,54	5,17	1,37
CR745430	CR745430 Soares_testis_NHT Homo sapiens cDNA clone IMAGp971P1077 ; IMAGE:1839490 5', mRNA sequence [CR745430]	6,39	5,02	1,37
-	chr2:001516473-001516532	5,97	4,6	1,37
SLC23A2	Homo sapiens solute carrier family 23 (nucleobase transporters), member 2 (SLC23A2), transcript variant 2, mRNA [NM_203327]	9,45	8,1	1,36
RAB35	Homo sapiens RAB35, member RAS oncogene family (RAB35), mRNA [NM_006861]	13,3	11,95	1,36
HS1BP3	Homo sapiens HCLS1 binding protein 3 (HS1BP3), mRNA [NM_022460]	8,87	7,5	1,36
GPR42	Homo sapiens G protein-coupled receptor 42 (GPR42), mRNA [NM_005305]	5,75	4,39	1,36
CHD8	Homo sapiens chromodomain helicase DNA binding protein 8 (CHD8), mRNA [NM_020920]	8,92	7,57	1,36
LOC146346	Homo sapiens cDNA FLJ32797 fis, clone TESTI2002467, [AK057359]	10,74	9,38	1,36
ENST0000030045[illegible]	CDNA FLJ20694 fis, clone KAIA2518, [Source:Uniprot/SPTREMBL;Acc:Q9NWQ1] [ENST00000300458]	5,98	4,62	1,36
LMAN1L	Homo sapiens lectin, mannose-binding, 1 like (LMAN1L), mRNA [NM_021819]	6,41	5,06	1,35
LOC644974	Homo sapiens cDNA FLJ11976 fis, clone HEMBB1001253, [AK022038]	7,72	6,38	1,35
VPS35	Homo sapiens vacuolar protein sorting 35 homolog (S, cerevisiae) (VPS35), mRNA [NM_018206]	10,97	9,63	1,34
SIRT3	Homo sapiens sirtuin (silent mating type information regulation 2 homolog) 3 (S, cerevisiae) (SIRT3), transcript variant 1, mRNA [NM_012239]	9,4	8,06	1,34
MECP2	Homo sapiens methyl CpG binding protein 2 (Rett syndrome) (MECP2), mRNA [NM_004992]	9,52	8,18	1,34
KRTAP4-12	Homo sapiens keratin associated protein 4-12 (KRTAP4-12), mRNA [NM_031854]	5,23	3,89	1,34
KLHDC5	Homo sapiens kelch domain containing 5 (KLHDC5), mRNA [NM_020782]	11,01	9,67	1,34
CDIPT	Homo sapiens CDP-diacylglycerol--inositol 3-phosphatidyltransferase (phosphatidylinositol synthase) (CDIPT), mRNA [NM_006319]	10,68	9,33	1,34
LOC728450	OTTHUMP00000030286 (Fragment), [Source:Uniprot/SPTREMBL;Acc:Q9NU29] [ENST00000358396]	6,27	4,94	1,33
PRMT7	Homo sapiens protein arginine methyltransferase 7 (PRMT7), mRNA [NM_019023]	7,34	6,01	1,33
C17orf58	Homo sapiens chromosome 17 open reading frame 58 (C17orf58), transcript variant 1, mRNA [NM_181655]	8,73	7,41	1,33
AF268617	Homo sapiens POU 5 domain protein (POU5FLC12) mRNA, complete cds, [AF268617]	5,68	4,36	1,32
LPIN3	Homo sapiens lipin 3 (LPIN3), mRNA [NM_022896]	5,47	4,15	1,32
AK096196	Homo sapiens cDNA FLJ38877 fis, clone MESAN2015277, [AK096196]	6,32	5	1,32
LOC441120	Homo sapiens similar to LOC285679 protein (LOC441120), mRNA [NM_001013718]	6,23	4,92	1,31

Supplementary Table 1
Morandi et al

INTS5	Homo sapiens integrator complex subunit 5 (INTS5), mRNA [NM_030628]	7,78	6,47	1,31
CLDN7	Homo sapiens claudin 7 (CLDN7), mRNA [NM_001307]	5,59	4,27	1,31
ELF5	Homo sapiens cDNA FLJ90152 fis, clone HEMBB1002051, weakly similar to Homo sapiens Ets transcription factor ESE-2b mRNA, [AK074633]	6,64	5,32	1,31
ZNF346	Homo sapiens zinc finger protein 346 (ZNF346), mRNA [NM_012279]	5,89	4,59	1,3
SCNN1B	Homo sapiens sodium channel, nonvoltage-gated 1, beta (Liddle syndrome) (SCNN1B), mRNA [NM_000336]	6,14	4,84	1,3
HDHD3	Homo sapiens haloacid dehalogenase-like hydrolase domain containing 3 (HDHD3), mRNA [NM_031219]	6,28	4,98	1,3
FRMD1	Homo sapiens FERM domain containing 1 (FRMD1), mRNA [NM_024919]	5,49	4,19	1,3
AA282192	AA282192 zs89b10,r1 NCI_CGAP_GCB1 Homo sapiens cDNA clone IMAGE:704635 5', mRNA sequence [AA282192]	7,04	5,74	1,3
GOT2	Human mitochondrial aspartate aminotransferase mRNA, complete cds, [M22632]	9,84	8,54	1,29
VPS13D	Homo sapiens vacuolar protein sorting 13 homolog D (S, cerevisiae) (VPS13D), transcript variant 1, mRNA [NM_015378]	6,49	5,2	1,29
DAB1	Homo sapiens disabled homolog 1 (Drosophila) (DAB1), mRNA [NM_021080]	6,23	4,93	1,29
AP3M2	Homo sapiens adaptor-related protein complex 3, mu 2 subunit (AP3M2), mRNA [NM_006803]	10,78	9,49	1,29
ABHD6	Homo sapiens abhydrolase domain containing 6 (ABHD6), mRNA [NM_020676]	9,85	8,56	1,29
-	chr9:005408444-005408385	6,42	5,14	1,29
-	chr10:006608677-006608618	7,24	5,95	1,29
WDR7	Homo sapiens WD repeat domain 7 (WDR7), transcript variant 1, mRNA [NM_015285]	10,56	9,28	1,28
SNAP29	Homo sapiens synaptosomal-associated protein, 29kDa (SNAP29), mRNA [NM_004782]	7	5,73	1,28
MYADML	Homo sapiens myeloid-associated differentiation marker-like (MYADML) on chromosome 2 [NR_003143]	5,93	4,65	1,28
CKAP5	Homo sapiens cytoskeleton associated protein 5 (CKAP5), transcript variant 1, mRNA [NM_001008938]	9,19	7,91	1,28
C10orf46	Homo sapiens chromosome 10 open reading frame 46 (C10orf46), mRNA [NM_153810]	9,7	8,42	1,28
AGPAT6	Homo sapiens 1-acylglycerol-3-phosphate O-acyltransferase 6 (lysophosphatidic acid acyltransferase, zeta) (AGPAT6), mRNA [NM_178819]	10,81	9,53	1,28
SPG7	Homo sapiens spastic paraplegia 7, paraplegin (pure and complicated autosomal recessive) (SPG7), nuclear gene encoding mitochondrial protein, transcript variant 1, mRNA [NM_003119]	9,69	8,41	1,27
FLJ00049	Homo sapiens mRNA for FLJ00049 protein, partial cds, [AK024457]	6,57	5,29	1,27
LENG9	Homo sapiens leukocyte receptor cluster (LRC) member 9 (LENG9), mRNA [NM_198988]	5,89	4,63	1,27
EGLN2	Homo sapiens egl nine homolog 2 (C, elegans) (EGLN2), transcript variant 3, mRNA [NM_080732]	11,15	9,88	1,27
FLJ38379	Homo sapiens cDNA FLJ38379 fis, clone FEBRA2002986, [AK095698]	7,23	5,96	1,27
BC021053	Homo sapiens cDNA clone IMAGE:2960979, **** WARNING: chimeric clone ****, [BC021053]	8,52	7,26	1,25
TMEM9	Homo sapiens transmembrane protein 9 (TMEM9), mRNA [NM_016456]	12,06	10,82	1,24
SETX	Homo sapiens senataxin (SETX), mRNA [NM_015046]	10,39	9,15	1,24

Supplementary Table 1
Morandi et al

LOC56757	Homo sapiens cDNA: FLJ23595 fis, clone LNG15262, [AK027248]	10,05	8,8	1,24
KIAA0828	Homo sapiens adenosylhomocysteinase 3 (KIAA0828), mRNA [NM_015328]	9,99	8,76	1,23
THC2699738	BC031068 AADAT protein {Homo sapiens} (exp=-1; wgp=0; cg=0), partial (8%) [THC2699738]	5,9	4,67	1,23
FBXW11	Homo sapiens F-box and WD-40 domain protein 11 (FBXW11), transcript variant 3, mRNA [NM_012300]	12,36	11,14	1,22
C19orf19	Homo sapiens chromosome 19 open reading frame 19 (C19orf19), mRNA [NM_182577]	7,44	6,22	1,22
JAK3	Homo sapiens Janus kinase 3 (a protein tyrosine kinase, leukocyte) (JAK3), mRNA [NM_000215]	7,13	5,92	1,21
BCDIN3	Homo sapiens bin3, bicoid-interacting 3, homolog (Drosophila) (BCDIN3), mRNA [NM_019606]	9,1	7,89	1,21
THC2713663	chr4:80448740-80448799	6,3	5,09	1,21
RAB14	Homo sapiens RAB14, member RAS oncogene family (RAB14), mRNA [NM_016322]	11,1	9,9	1,2
FLT4	Homo sapiens fms-related tyrosine kinase 4 (FLT4), transcript variant 1, mRNA [NM_182925]	6,45	5,26	1,19
UBE2Z	Homo sapiens ubiquitin-conjugating enzyme E2Z (putative) (UBE2Z), mRNA [NM_023079]	8,75	7,56	1,18
PIK3R4	Homo sapiens phosphoinositide-3-kinase, regulatory subunit 4, p150 (PIK3R4), mRNA [NM_014602]	8,7	7,52	1,18
KIF13B	Homo sapiens kinesin family member 13B (KIF13B), mRNA [NM_015254]	11,99	10,81	1,18
DNAL4	Homo sapiens dynein, axonemal, light chain 4 (DNAL4), mRNA [NM_005740]	7,99	6,81	1,18
TFF1	Homo sapiens trefoil factor 1 (breast cancer, estrogen-inducible sequence expressed in) (TFF1), mRNA [NM_003225]	6,4	5,24	1,17
PLCG1	Homo sapiens phospholipase C, gamma 1 (PLCG1), transcript variant 1, mRNA [NM_002660]	9,38	8,2	1,17
ENST0000033352[illegible]	Homo sapiens C21orf86 protein (C21orf86) mRNA, complete cds, [AF426264]	5,84	4,67	1,17
ZMYM3	Homo sapiens zinc finger, MYM-type 3 (ZMYM3), transcript variant 1, mRNA [NM_005096]	8,47	7,3	1,16
GRB2	Homo sapiens growth factor receptor-bound protein 2 (GRB2), transcript variant 1, mRNA [NM_002086]	11,55	10,38	1,16
SART3	Homo sapiens squamous cell carcinoma antigen recognized by T cells 3 (SART3), mRNA [NM_014706]	10,38	9,23	1,15
C7orf42	Homo sapiens chromosome 7 open reading frame 42 (C7orf42), mRNA [NM_017994]	13,36	12,21	1,15
C6orf106	Homo sapiens chromosome 6 open reading frame 106 (C6orf106), transcript variant 1, mRNA [NM_024294]	8,94	7,79	1,15
C17orf62	Homo sapiens chromosome 17 open reading frame 62 (C17orf62), mRNA [NM_001033046]	11,09	9,93	1,15
CCDC25	Homo sapiens coiled-coil domain containing 25 (CCDC25), mRNA [NM_018246]	9,4	8,26	1,13
LOC147650	Homo sapiens clone DNA92254 ALLA2487 (UNQ2487) mRNA, complete cds, [AY358799]	6,29	5,17	1,12
KIAA0146	full-length cDNA clone CS0DK002YC19 of HeLa cells Cot 25-normalized of Homo sapiens (human), [CR614430]	9,82	8,71	1,11
BM725480	BM725480 UI-E-EJ0-aie-p-22-0-UI,r1 UI-E-EJ0 Homo sapiens cDNA clone UI-E-EJ0-aie-p-22-0-UI 5', mRNA sequence [BM725480]	9,45	8,34	1,11
APTX	Homo sapiens aprataxin (APTX), transcript variant 1, mRNA [NM_175073]	10,59	9,49	1,1
SUPV3L1	Homo sapiens suppressor of var1, 3-like 1 (S, cerevisiae) (SUPV3L1), mRNA [NM_003171]	9,96	8,87	1,09
SCARF1	Homo sapiens scavenger receptor class F, member 1 (SCARF1), transcript variant 4, mRNA [NM_145351]	7,23	6,14	1,08

Supplementary Table 1
Morandi et al

ST13	Homo sapiens suppression of tumorigenicity 13 (colon carcinoma) (Hsp70 interacting protein) (ST13), mRNA [NM_003932]	11,75	10,68	1,06
GPR77	Homo sapiens G protein-coupled receptor 77 (GPR77), mRNA [NM_018485]	5,95	4,9	1,06
FTSJ3	Homo sapiens FtsJ homolog 3 (E, coli) (FTSJ3), mRNA [NM_017647]	12	10,94	1,06
FAM109A	Homo sapiens family with sequence similarity 109, member A (FAM109A), mRNA [NM_144671]	8,74	7,68	1,06
KCTD10	Homo sapiens potassium channel tetramerisation domain containing 10 (KCTD10), mRNA [NM_031954]	10,36	9,31	1,05
DGCR2	Homo sapiens DiGeorge syndrome critical region gene 2 (DGCR2), mRNA [NM_005137]	13,3	12,25	1,05
CSNK2A2	Homo sapiens casein kinase 2, alpha prime polypeptide (CSNK2A2), mRNA [NM_001896]	9,52	8,47	1,04
WHSC1L1	Homo sapiens Wolf-Hirschhorn syndrome candidate 1-like 1 (WHSC1L1), transcript variant short, mRNA [NM_017778]	9,87	8,84	1,03
PRSS8	Homo sapiens protease, serine, 8 (prostasin) (PRSS8), mRNA [NM_002773]	6,32	5,29	1,03
CLASP1	Homo sapiens cytoplasmic linker associated protein 1 (CLASP1), mRNA [NM_015282]	10,32	9,28	1,03
AP2A2	Homo sapiens adaptor-related protein complex 2, alpha 2 subunit (AP2A2), mRNA [NM_012305]	9,38	8,37	1,01
PQLC1	Homo sapiens PQ loop repeat containing 1 (PQLC1), mRNA [NM_025078]	9,7	8,71	0,99
REXO4	Homo sapiens REX4, RNA exonuclease 4 homolog (S, cerevisiae) (REXO4), mRNA [NM_020385]	9,49	8,52	0,97
OPRD1	Homo sapiens opioid receptor, delta 1 (OPRD1), mRNA [NM_000911]	5,44	4,48	0,95
KLHL12	Homo sapiens kelch-like 12 (Drosophila) (KLHL12), mRNA [NM_021633]	10,89	9,98	0,92
TCEA2	Homo sapiens transcription elongation factor A (SII), 2 (TCEA2), transcript variant 1, mRNA [NM_003195]	11,88	10,97	0,91
PPIL2	Homo sapiens peptidylprolyl isomerase (cyclophilin)-like 2 (PPIL2), transcript variant 2, mRNA [NM_148175]	9,88	8,97	0,91
BAZ1B	Homo sapiens bromodomain adjacent to zinc finger domain, 1B (BAZ1B), mRNA [NM_032408]	11,74	10,84	0,9
CXXC1	Homo sapiens CXXC finger 1 (PHD domain) (CXXC1), mRNA [NM_014593]	10,48	9,59	0,89
CHMP7	Homo sapiens CHMP family, member 7 (CHMP7), mRNA [NM_152272]	10,28	9,39	0,89
BANP	Homo sapiens BTG3 associated nuclear protein (BANP), transcript variant 1, mRNA [NM_017869]	7,81	6,93	0,87
CLPX	Homo sapiens ClpX caseinolytic peptidase X homolog (E, coli) (CLPX), mRNA [NM_006660]	9,74	10,62	-0,88
DDHD1	Homo sapiens DDHD domain containing 1 (DDHD1), mRNA [NM_030637]	6,05	6,97	-0,91
PCBD2	Homo sapiens pterin-4 alpha-carbinolamine dehydratase/dimerization cofactor of hepatocyte nuclear factor 1 alpha (TCF1) 2 (PCBD2), mRNA [NM_032151]	8,28	9,2	-0,92
ABCB7	Homo sapiens ATP-binding cassette, sub-family B (MDR/TAP), member 7 (ABCB7), nuclear gene encoding mitochondrial protein, mRNA [NM_004299]	9,48	10,47	-0,99
ZNF780B	Homo sapiens zinc finger protein 780B (ZNF780B), mRNA [NM_001005851]	7,33	8,38	-1,04
RAF1	Homo sapiens v-raf-1 murine leukemia viral oncogene homolog 1 (RAF1), mRNA [NM_002880]	10,91	11,98	-1,07
APOM	Homo sapiens apolipoprotein M (APOM), mRNA [NM_019101]	6,17	7,26	-1,08
NCOA4	Homo sapiens nuclear receptor coactivator 4 (NCOA4), mRNA [NM_005437]	9,35	10,46	-1,1
MED28	Homo sapiens tumor-related protein mRNA, complete cds, [AF317680]	8,26	9,44	-1,17

Supplementary Table 1
Morandi et al

UXT	Homo sapiens ubiquitously-expressed transcript (UXT), transcript variant 2, mRNA [NM_004182]	12,05	13,24	-1,19
C2orf25	Homo sapiens chromosome 2 open reading frame 25 (C2orf25), mRNA [NM_015702]	11,35	12,53	-1,19
CDC42SE2	Homo sapiens CDC42 small effector 2 (CDC42SE2), transcript variant 1, mRNA [NM_020240]	8,34	9,53	-1,19
AFF4	Homo sapiens AF4/FMR2 family, member 4 (AFF4), mRNA [NM_014423]	6,41	7,6	-1,19
FKBP1A	Homo sapiens FK506 binding protein 1A, 12kDa (FKBP1A), transcript variant 12A, mRNA [NM_054014]	8,09	9,29	-1,2
C1orf58	Homo sapiens chromosome 1 open reading frame 58 (C1orf58), mRNA [NM_144695]	5,23	6,43	-1,2
UBE1L2	Homo sapiens ubiquitin-activating enzyme E1-like 2 (UBE1L2), mRNA [NM_018227]	6,33	7,54	-1,21
MGC4093	Homo sapiens hypothetical protein MGC4093 (MGC4093), mRNA [NM_030578]	8,78	9,99	-1,21
CSNK1G3	Homo sapiens casein kinase 1, gamma 3 (CSNK1G3), transcript variant 4, mRNA [NM_001044723]	7,19	8,41	-1,23
LIN7C	Homo sapiens lin-7 homolog C (C, elegans) (LIN7C), mRNA [NM_018362]	7,38	8,63	-1,26
HSPA4	Homo sapiens heat shock 70kDa protein 4 (HSPA4), transcript variant 1, mRNA [NM_002154]	12,52	13,84	-1,31
ASB7	Homo sapiens ankyrin repeat and SOCS box-containing 7 (ASB7), transcript variant 1, mRNA [NM_024708]	5,59	6,9	-1,31
CD46	Homo sapiens CD46 molecule, complement regulatory protein (CD46), transcript variant n, mRNA [NM_172350]	6,65	7,97	-1,33
MRPS30	Homo sapiens mitochondrial ribosomal protein S30 (MRPS30), nuclear gene encoding mitochondrial protein, mRNA [NM_016640]	7,77	9,11	-1,34
SNX5	Homo sapiens sorting nexin 5 (SNX5), transcript variant 2, mRNA [NM_014426]	11,03	12,38	-1,35
RBM3	Homo sapiens RNA binding motif (RNP1, RRM) protein 3 (RBM3), transcript variant 2, mRNA [NM_001017430]	11,38	12,74	-1,35
FUSIP1	Homo sapiens FUS interacting protein (serine/arginine-rich) 1 (FUSIP1), transcript variant 1, mRNA [NM_006625]	8,38	9,75	-1,37
-	chr7:072331752-072331693	5,97	7,34	-1,37
FAM119A	Homo sapiens family with sequence similarity 119, member A (FAM119A), mRNA [NM_145280]	8,12	9,5	-1,38
SFRS6	Homo sapiens splicing factor, arginine/serine-rich 6 (SFRS6), mRNA [NM_006275]	8,55	9,94	-1,39
CA5B	Homo sapiens carbonic anhydrase VB, mitochondrial (CA5B), nuclear gene encoding mitochondrial protein, mRNA [NM_007220]	5,72	7,11	-1,39
UBE2D1	Homo sapiens ubiquitin-conjugating enzyme E2D 1 (UBC4/5 homolog, yeast) (UBE2D1), mRNA [NM_003338]	7,87	9,27	-1,4
CLEC2D	Homo sapiens C-type lectin domain family 2, member D (CLEC2D), transcript variant 2, mRNA [NM_001004419]	11,64	13,05	-1,4
BE970058	BE970058 601680172F1 NIH_MGC_78 Homo sapiens cDNA clone IMAGE:3950464 5', mRNA sequence [BE970058]	9,19	10,6	-1,4
RASA2	Homo sapiens RAS p21 protein activator 2 (RASA2), mRNA [NM_006506]	5,6	7,01	-1,41
DERL2	Homo sapiens Der1-like domain family, member 2 (DERL2), mRNA [NM_016041]	10,17	11,58	-1,42
NR2C2	Homo sapiens nuclear receptor subfamily 2, group C, member 2 (NR2C2), mRNA [NM_003298]	7,13	8,57	-1,44
LOC652411	PREDICTED: Homo sapiens similar to laminin receptor 1 (ribosomal protein SA) (LOC652411), mRNA [XR_019314]	8,55	10	-1,45

Supplementary Table 1
Morandi et al

GTF3C3	Homo sapiens transcription factor IIIC102 short isoform mRNA, complete cds, [AF465407]	5,71	7,16	-1,45
AK022443	Homo sapiens cDNA FLJ12381 fis, clone MAMMA1002566, [AK022443]	8,56	10,01	-1,45
2'-PDE	Homo sapiens 2'-phosphodiesterase (2'-PDE), mRNA [NM_177966]	4,77	6,22	-1,45
SEPX1	Homo sapiens selenoprotein X, 1 (SEPX1), mRNA [NM_016332]	10,45	11,91	-1,46
CAT	Homo sapiens catalase (CAT), mRNA [NM_001752]	9,69	11,16	-1,48
THC2639689	chr3:71401016-71400957	8,41	9,91	-1,5
-	chr20:033791888-033792225	4,09	5,59	-1,5
DDX3X	Homo sapiens DEAD (Asp-Glu-Ala-Asp) box polypeptide 3, X-linked (DDX3X), mRNA [NM_001356]	6,79	8,3	-1,51
STT3B	Homo sapiens STT3, subunit of the oligosaccharyltransferase complex, homolog B (S, cerevisiae) (STT3B), mRNA [NM_178862]	6,98	8,5	-1,52
LRIG2	Homo sapiens leucine-rich repeats and immunoglobulin-like domains 2 (LRIG2), mRNA [NM_014813]	7,4	8,92	-1,52
LOC440180	PREDICTED: Homo sapiens similar to zinc finger, CCHC domain containing 7 (LOC440180), mRNA [XR_018312]	5	6,53	-1,53
C15orf29	Homo sapiens chromosome 15 open reading frame 29 (C15orf29), mRNA [NM_024713]	8,8	10,33	-1,53
BE156557	BE156557 QV0-HT0368-090200-099-d10 HT0368 Homo sapiens cDNA, mRNA sequence [BE156557]	6,64	8,17	-1,53
VTI1A	Homo sapiens vesicle transport through interaction with t-SNAREs homolog 1A (yeast) (VTI1A), transcript variant 2, mRNA [NM_145206]	7,32	8,86	-1,54
LOC392522	PREDICTED: Homo sapiens similar to ribosomal protein L18a (LOC392522), mRNA [XR_018292]	11,65	13,2	-1,55
PSEN1	Homo sapiens PSN1 gene, alternative transcript, [AJ008005]	5,86	7,41	-1,55
BTG1	Homo sapiens B-cell translocation gene 1, anti-proliferative (BTG1), mRNA [NM_001731]	11,59	13,14	-1,55
LOC644422	PREDICTED: Homo sapiens similar to arginine [XR_019449]	7,47	9,04	-1,57
STK4	Homo sapiens serine/threonine kinase 4 (STK4), mRNA [NM_006282]	4,72	6,29	-1,57
THC2525408	AV738929 AV738929 CB Homo sapiens cDNA clone CBCCMG04 5', mRNA sequence [AV738929]	6,87	8,44	-1,57
LOC730850	PREDICTED: Homo sapiens similar to peptidylprolyl isomerase A isoform 1 (LOC730850), mRNA [XR_015255]	7,65	9,23	-1,58
TBRG1	Homo sapiens transforming growth factor beta regulator 1, mRNA (cDNA clone IMAGE:5212572), complete cds, [BC032312]	8,92	10,5	-1,58
C10orf12	Homo sapiens chromosome 10 open reading frame 12 (C10orf12), mRNA [NM_015652]	4,75	6,33	-1,58
ATP11B	Homo sapiens ATPase, Class VI, type 11B (ATP11B), mRNA [NM_014616]	7,15	8,73	-1,58
C20orf199	Homo sapiens cDNA FLJ42181 fis, clone THYMU2031368, [AK124175]	5,56	7,15	-1,59
CF529502	CF529502 UI-1-BC1p-ash-d-10-0-UI,s1 NCI_CGAP_Pl3 Homo sapiens cDNA clone UI-1-BC1p-ash-d-10-0-UI 3', mRNA sequence [CF529502]	9,6	11,19	-1,59
AV722457	AV722457 HTB Homo sapiens cDNA clone HTBAMH03 5', mRNA sequence [AV722457]	5,98	7,57	-1,6
SLC6A6	Homo sapiens solute carrier family 6 (neurotransmitter transporter, taurine), member 6 (SLC6A6), mRNA [NM_003043]	8,81	10,42	-1,61
CA948108	CA948108 iq21e07,x1 HR85 islet Homo sapiens cDNA clone IMAGE: 3', mRNA sequence [CA948108]	5,1	6,71	-1,61
CCNT2	Homo sapiens cyclin T2 (CCNT2), transcript variant b, mRNA [NM_058241]	5,64	7,27	-1,64

Supplementary Table 1
Morandi et al

BE168511	BE168511 QV3-HT0514-220300-127-d06 HT0514 Homo sapiens cDNA, mRNA sequence [BE168511]	5,27	6,91	-1,64
FLJ10213	Homo sapiens hypothetical protein FLJ10213 (FLJ10213), mRNA [NM_018029]	9,5	11,16	-1,66
C16orf74	Homo sapiens chromosome 16 open reading frame 74, mRNA (cDNA clone MGC:17624 IMAGE:3855543), complete cds, [BC009078]	5,89	7,55	-1,66
ENST0000038299(	chr16:221400-221459	4,76	6,43	-1,66
FLJ20186	Homo sapiens hypothetical protein FLJ20186 (FLJ20186), transcript variant 2, mRNA [NM_017702]	9,89	11,56	-1,67
DOCK11	Homo sapiens dedicator of cytokinesis 11 (DOCK11), mRNA [NM_144658]	4,8	6,47	-1,67
RAB27A	Homo sapiens RAB27A, member RAS oncogene family (RAB27A), transcript variant 1, mRNA [NM_004580]	5,64	7,32	-1,68
ELOVL5	Homo sapiens ELOVL family member 5, elongation of long chain fatty acids (FEN1/Elo2, SUR4/Elo3-like, yeast) (ELOVL5), mRNA [NM_021814]	4,87	6,56	-1,69
MTP18	Homo sapiens mitochondrial protein 18 kDa (MTP18), nuclear gene encoding mitochondrial protein, transcript variant 1, mRNA [NM_016498]	7,86	9,57	-1,71
LOC285074	Homo sapiens hypothetical protein LOC285074 (LOC285074), mRNA [NM_001012626]	9,44	11,14	-1,71
-	chrX_random:000899134-000899193	5,59	7,31	-1,71
MCL1	Homo sapiens myeloid cell leukemia sequence 1 (BCL2-related) (MCL1), transcript variant 1, mRNA [NM_021960]	6,92	8,64	-1,72
HIST1H2BD	Homo sapiens histone cluster 1, H2bd (HIST1H2BD), transcript variant 1, mRNA [NM_021063]	8,32	10,04	-1,72
BCL2L13	Homo sapiens BCL2-like 13 (apoptosis facilitator) (BCL2L13), nuclear gene encoding mitochondrial protein, mRNA [NM_015367]	6,44	8,16	-1,72
X05126	Human fibroblast mRNA fragment with Alu sequence (pRHF11), [X05126]	6,11	7,85	-1,73
ALG13	Homo sapiens asparagine-linked glycosylation 13 homolog (S, cerevisiae) (ALG13), mRNA [NM_018466]	8,29	10,02	-1,73
ANKRD44	Homo sapiens ankyrin repeat domain 44 (ANKRD44), mRNA [NM_153697]	5,97	7,7	-1,73
NLRP1	Homo sapiens NLR family, pyrin domain containing 1 (NLRP1), transcript variant 1, mRNA [NM_033004]	6,73	8,48	-1,74
THC2532862	chr3:10309228-10309287	5,74	7,49	-1,75
WBSCR16	Homo sapiens Williams-Beuren syndrome chromosome region 16, mRNA (cDNA clone MGC:49849 IMAGE:5785681), complete cds, [BC040695]	5,34	7,1	-1,76
UBE2W	Homo sapiens ubiquitin-conjugating enzyme E2W (putative) (UBE2W), transcript variant 1, mRNA [NM_001001481]	6,39	8,15	-1,76
ENST0000032847	Homo sapiens PRO1843 mRNA, complete cds, [AF119854]	8,21	9,97	-1,77
ATP11C	Homo sapiens ATPase, Class VI, type 11C (ATP11C), transcript variant 2, mRNA [NM_001010986]	5,44	7,21	-1,77
RNF141	Homo sapiens ring finger protein 141 (RNF141), mRNA [NM_016422]	8,52	10,3	-1,78
PPP1R15B	Homo sapiens protein phosphatase 1, regulatory (inhibitor) subunit 15B (PPP1R15B), mRNA [NM_032833]	6,16	7,94	-1,78
TGFB1	Homo sapiens transforming growth factor, beta 1 (Camurati-Engelmann disease) (TGFB1), mRNA [NM_000660]	7,34	9,15	-1,8
HPS1	Homo sapiens Hermansky-Pudlak syndrome 1 (HPS1), transcript variant 1, mRNA [NM_000195]	6,45	8,26	-1,8
EIF2C4	Homo sapiens eukaryotic translation initiation factor 2C, 4 (EIF2C4), mRNA [NM_017629]	6,95	8,75	-1,8

Supplementary Table 1
Morandi et al

BE696323	BE696323 RC3-CT0347-010700-019-c08 CT0347 Homo sapiens cDNA, mRNA sequence [BE696323]	5,35	7,15	-1,8
RP11-114G1,1	Homo sapiens Sp3 transcription factor pseudogene, mRNA (cDNA clone MGC:43345 IMAGE:5266022), complete cds, [BC036697]	6,36	8,17	-1,81
FLJ39660	Homo sapiens mRNA; cDNA DKFZp434P055 (from clone DKFZp434P055), [AL834537]	7,1	8,91	-1,81
MAP2K3	Homo sapiens mitogen-activated protein kinase kinase 3 (MAP2K3), transcript variant B, mRNA [NM_145109]	12,13	13,94	-1,81
CEBPG	Homo sapiens CCAAT/enhancer binding protein (C/EBP), gamma (CEBPG), mRNA [NM_001806]	10,14	11,95	-1,81
-	chr17:037939683-037939742	11,37	13,18	-1,81
CRSP3	Homo sapiens cofactor required for Sp1 transcriptional activation, subunit 3, 130kDa (CRSP3), transcript variant 2, mRNA [NM_015979]	5,75	7,57	-1,82
-	chr22:019635752-019635693	5,92	7,73	-1,82
SERPINB8	Homo sapiens serpin peptidase inhibitor, clade B (ovalbumin), member 8 (SERPINB8), transcript variant 2, mRNA [NM_198833]	8,37	10,2	-1,83
HIST1H2BC	Homo sapiens histone cluster 1, H2bc (HIST1H2BC), mRNA [NM_003526]	6,76	8,6	-1,84
CR749652	Homo sapiens mRNA; cDNA DKFZp686I07120 (from clone DKFZp686I07120), [CR749652]	9,12	10,97	-1,85
CFLAR	Homo sapiens FLAME-1 mRNA, complete cds, [AF009616]	8,13	9,99	-1,86
ZFY	Homo sapiens zinc finger protein, Y-linked (ZFY), mRNA [NM_003411]	5,29	7,16	-1,87
USP3	Homo sapiens ubiquitin specific peptidase 3 (USP3), mRNA [NM_006537]	8,5	10,37	-1,87
-	chrX:041093244-041093185	3,81	5,68	-1,87
LOC646626	PREDICTED: Homo sapiens hypothetical LOC646626 (LOC646626), mRNA [XM_942822]	7,08	8,97	-1,88
SPFH1	Homo sapiens SPFH domain family, member 1 (SPFH1), mRNA [NM_006459]	5,58	7,45	-1,88
BX641010	Homo sapiens mRNA; cDNA DKFZp686G01227 (from clone DKFZp686G01227), [BX641010]	7,06	8,94	-1,88
-	chr5:108092085-108092144	7,12	9	-1,88
-	chr4:120598637-120598694	4,19	6,07	-1,88
LOC645592	PREDICTED: Homo sapiens similar to peptidylprolyl isomerase A isoform 1 (LOC645592), mRNA [XR_019387]	7,28	9,17	-1,89
PRKAA1	Homo sapiens protein kinase, AMP-activated, alpha 1 catalytic subunit (PRKAA1), transcript variant 2, mRNA [NM_206907]	7,17	9,06	-1,89
KCNN4	Homo sapiens potassium intermediate/small conductance calcium-activated channel, subfamily N, member 4 (KCNN4), mRNA [NM_002250]	7,81	9,7	-1,89
PHC3	Homo sapiens polyhomeotic homolog 3 (Drosophila) (PHC3), mRNA [NM_024947]	6,26	8,16	-1,89
CMTM7	Homo sapiens CKLF-like MARVEL transmembrane domain containing 7 (CMTM7), transcript variant 1, mRNA [NM_138410]	9,87	11,76	-1,89
U2AF1	Homo sapiens cDNA FLJ13527 fis, clone PLACE1006076, [AK023589]	5,46	7,35	-1,89
NIN	Homo sapiens ninein (GSK3B interacting protein) (NIN), transcript variant 4, mRNA [NM_016350]	7,56	9,46	-1,9
THC2552359	Q4RCF0_TETNG (Q4RCF0) Chromosome undetermined SCAF19066, whole genome shotgun sequence, (Fragment), partial (5%) [THC2552359]	7,43	9,34	-1,91
HIST1H2BB	Homo sapiens histone cluster 1, H2bb (HIST1H2BB), mRNA [NM_021062]	8,41	10,32	-1,91
CCNL1	Homo sapiens cyclin L1 (CCNL1), mRNA [NM_020307]	9,93	11,84	-1,91
GLRX	Homo sapiens glutaredoxin (thioltransferase) (GLRX), mRNA [NM_002064]	10,71	12,63	-1,92

Supplementary Table 1
Morandi et al

ZMYM2	Homo sapiens mRNA; cDNA DKFZp564B162 (from clone DKFZp564B162), [AL136621]	8,31	10,25	-1,93
GOPC	Homo sapiens golgi associated PDZ and coiled-coil motif containing (GOPC), transcript variant 1, mRNA [NM_020399]	7	8,93	-1,93
DHFR	Homo sapiens dihydrofolate reductase (DHFR), mRNA [NM_000791]	6,43	8,36	-1,93
-	chr9:094036521-094036462	5,23	7,16	-1,93
MAP3K7IP3	Homo sapiens mitogen-activated protein kinase kinase kinase 7 interacting protein 3 (MAP3K7IP3), mRNA [NM_152787]	4,35	6,29	-1,94
KLHL7	Homo sapiens kelch-like 7 (Drosophila), mRNA (cDNA clone IMAGE:3899090), complete cds, [BC009555]	7,64	9,58	-1,94
CV339166	CV339166 MR0-BT2503-050601-101-a04 BT2503 Homo sapiens cDNA, mRNA sequence [CV339166]	4,66	6,6	-1,94
HIST1H2BG	Homo sapiens histone cluster 1, H2bg (HIST1H2BG), mRNA [NM_003518]	7,25	9,2	-1,95
ATP13A3	Probable cation-transporting ATPase 13A3 (EC 3,6,3,-) (ATPase family homolog up-regulated in senescence cells 1), [Source:Uniprot/SWISSPROT;Acc:Q9H7F0] [ENST00000256031]	5,6	7,57	-1,96
MAN1A1	Homo sapiens mannosidase, alpha, class 1A, member 1 (MAN1A1), mRNA [NM_005907]	5,25	7,21	-1,96
FAM8A1	Homo sapiens family with sequence similarity 8, member A1 (FAM8A1), mRNA [NM_016255]	5,05	7,01	-1,96
TIA1	Homo sapiens TIA1 cytotoxic granule-associated RNA binding protein (TIA1), transcript variant 1, mRNA [NM_022037]	8,19	10,16	-1,97
BU684362	BU684362 UI-CF-EN0-aco-n-23-0-UI,s1 UI-CF-EN0 Homo sapiens cDNA clone UI-CF-EN0-aco-n-23-0-UI 3', mRNA sequence [BU684362]	4,58	6,56	-1,98
HIST1H2BO	Homo sapiens histone cluster 1, H2bo (HIST1H2BO), mRNA [NM_003527]	9,75	11,74	-1,99
AF116702	Homo sapiens PRO2446 mRNA, complete cds, [AF116702]	6,51	8,5	-2
HIST1H2AK	Homo sapiens histone cluster 1, H2ak (HIST1H2AK), mRNA [NM_003510]	10,91	12,91	-2
FEM1C	Homo sapiens fem-1 homolog c (C, elegans) (FEM1C), mRNA [NM_020177]	4,26	6,26	-2
AW797858	AW797858 CM0-UM0042-130300-280-d11 UM0042 Homo sapiens cDNA, mRNA sequence [AW797858]	6,99	8,99	-2
MICB	Homo sapiens MHC class I polypeptide-related sequence B (MICB), mRNA [NM_005931]	6,7	8,72	-2,02
FLJ39779	Homo sapiens FLJ39779 protein (FLJ39779), mRNA [NM_207442]	4,56	6,59	-2,02
SMPDL3A	Homo sapiens sphingomyelin phosphodiesterase, acid-like 3A (SMPDL3A), mRNA [NM_006714]	5,15	7,18	-2,03
FAM101B	Homo sapiens family with sequence similarity 101, member B (FAM101B), mRNA [NM_182705]	7,4	9,43	-2,03
-	chrX:067908109-067908168	6,42	8,44	-2,03
AU184995	AU184995 AU184995 Homo sapiens T-cell library (Sugita Y) Homo sapiens cDNA clone B00751-019, mRNA sequence [AU184995]	6,2	8,22	-2,03
THC2701748	Q5SVL1_HUMAN (Q5SVL1) Caspase 7, apoptosis-related cysteine protease, partial (25%) [THC2701748]	4,53	6,57	-2,04
SKP2	Homo sapiens S-phase kinase-associated protein 2 (p45) (SKP2), transcript variant 2, mRNA [NM_032637]	6,6	8,66	-2,06
HIST1H2BH	Homo sapiens histone cluster 1, H2bh (HIST1H2BH), mRNA [NM_003524]	9,98	12,05	-2,06
CR617865	full-length cDNA clone CS0DF021YD16 of Fetal brain of Homo sapiens (human), [CR617865]	7,13	9,18	-2,06

SPECC1	Homo sapiens sperm antigen with calponin homology and coiled-coil domains 1 (SPECC1), transcript variant NSP5beta3alpha, mRNA [NM_152904]	8,49	10,57	-2,08
GABPB2	Homo sapiens GA binding protein transcription factor, beta subunit 2 (GABPB2), transcript variant gamma-1, mRNA [NM_002041]	7,5	9,6	-2,09
LOC642123	Homo sapiens cDNA FLJ46881 fis, clone UTERU3015647, moderately similar to Embigin precursor, [AK128714]	5,99	8,08	-2,09
MLSTD2	Homo sapiens male sterility domain containing 2 (MLSTD2), mRNA [NM_032228]	5,93	8,03	-2,11
SERPINB1	Homo sapiens serpin peptidase inhibitor, clade B (ovalbumin), member 1 (SERPINB1), mRNA [NM_030666]	9,83	11,95	-2,12
RP5-1022P6,2	Homo sapiens hypothetical protein KIAA1434 (KIAA1434), mRNA [NM_019593]	9,91	12,03	-2,12
U25029	Human glucocorticoid receptor alpha mRNA, variant 3' UTR, [U25029]	7,29	9,42	-2,13
IL17RA	Homo sapiens interleukin 17 receptor A (IL17RA), mRNA [NM_014339]	8,39	10,53	-2,14
FAM122B	Homo sapiens family with sequence similarity 122B (FAM122B), mRNA [NM_145284]	6,4	8,54	-2,14
CCDC109B	Homo sapiens coiled-coil domain containing 109B (CCDC109B), mRNA [NM_017918]	7,61	9,75	-2,14
ARID5B	Homo sapiens AT rich interactive domain 5B (MRF1-like) (ARID5B), mRNA [NM_032199]	6,33	8,48	-2,15
RAP2C	Homo sapiens RAP2C, member of RAS oncogene family (RAP2C), mRNA [NM_021183]	5,17	7,35	-2,18
THC2674691	BOLA2_HUMAN (Q9H3K6) BolA-like protein 2, partial (33%) [THC2674691]	7,11	9,29	-2,18
THC2519484	S72008 CDC10 homolog {Homo sapiens} (exp=-1; wgp=0; cg=0), partial (9%) [THC2519484]	4,31	6,5	-2,2
TOB1	Homo sapiens, clone IMAGE:4133978, mRNA, [BC015064]	7,11	9,31	-2,2
BC020539	Homo sapiens, clone IMAGE:3048919, mRNA, partial cds, [BC020539]	8,45	10,67	-2,22
HK2	Homo sapiens hexokinase 2 (HK2), mRNA [NM_000189]	5,83	8,06	-2,23
CD79A	Homo sapiens CD79a molecule, immunoglobulin-associated alpha (CD79A), transcript variant 1, mRNA [NM_001783]	8,82	11,04	-2,23
LOC196264	QQRG2966 (Hypothetical protein LOC196264), [Source:Uniprot/SPTREMBL;Acc:Q6UWV2] [ENST00000278949]	6,09	8,34	-2,25
VCPIP1	Homo sapiens valosin containing protein (p97)/p47 complex interacting protein 1 (VCPIP1), mRNA [NM_025054]	6,07	8,32	-2,25
RP2	Homo sapiens retinitis pigmentosa 2 (X-linked recessive) (RP2), mRNA [NM_006915]	4,77	7,04	-2,27
C10orf54	Homo sapiens chromosome 10 open reading frame 54 (C10orf54), mRNA [NM_022153]	9,2	11,47	-2,27
ZBTB7B	Homo sapiens zinc finger and BTB domain containing 7B (ZBTB7B), mRNA [NM_015872]	5,61	7,89	-2,28
BF858887	BF858887 RC5-FT0194-071200-023-E10 FT0194 Homo sapiens cDNA, mRNA sequence [BF858887]	3,92	6,2	-2,28
AI381562	AI381562 te76g06,x1 Soares_NFL_T_GBC_S1 Homo sapiens cDNA clone IMAGE:2092666 3', mRNA sequence [AI381562]	4,82	7,11	-2,3
CCNB1	Homo sapiens cyclin B1 (CCNB1), mRNA [NM_031966]	9,41	11,73	-2,31
PTP4A1	Homo sapiens protein tyrosine phosphatase type IVA, member 1 (PTP4A1), mRNA [NM_003463]	6,12	8,43	-2,32
KIAA0179	Homo sapiens KIAA0179 (KIAA0179), mRNA [NM_015056]	6,74	9,05	-2,32
AL832717	Homo sapiens mRNA; cDNA DKFZp313B039 (from clone DKFZp313B039), [AL832717]	7,15	9,48	-2,33

Supplementary Table 1
Morandi et al

COTL1	Homo sapiens coactosin-like 1 (Dictyostelium) (COTL1), mRNA [NM_021149]	6,69	9,01	-2,33
EMP3	Homo sapiens epithelial membrane protein 3 (EMP3), mRNA [NM_001425]	9,2	11,57	-2,36
XRCC6BP1	Homo sapiens XRCC6 binding protein 1, mRNA (cDNA clone IMAGE:5742519), complete cds, [BC033881]	5,06	7,43	-2,37
BC009353	Homo sapiens immediate early response 2, mRNA (cDNA clone IMAGE:4297793), **** WARNING: chimeric clone ****, [BC009353]	4,68	7,05	-2,37
-	chr19:050721774-050721715	5,89	8,26	-2,37
SERTAD2	Homo sapiens SERTA domain containing 2 (SERTAD2), mRNA [NM_014755]	4,47	6,84	-2,38
-	chr1:120341166-120340857	9,44	11,83	-2,38
PHCA	Homo sapiens phytoceramidase, alkaline (PHCA), mRNA [NM_018367]	3,57	5,97	-2,4
ZFAND5	Homo sapiens zinc finger, AN1-type domain 5 (ZFAND5), mRNA [NM_006007]	10,57	12,98	-2,41
SERP1	Homo sapiens stress-associated endoplasmic reticulum protein 1 (SERP1), mRNA [NM_014445]	5,5	7,91	-2,41
FEM1B	Homo sapiens fem-1 homolog b (C, elegans) (FEM1B), mRNA [NM_015322]	6,61	9,04	-2,43
THC2694227	chr2:149166620-149166679	4,22	6,65	-2,43
THC2689175	AB002334 Start codon is not identified {Homo sapiens} (exp=-1; wgp=0; cg=0), partial (9%) [THC2689175]	6,13	8,56	-2,43
CNN2	Homo sapiens calponin 2 (CNN2), transcript variant 1, mRNA [NM_004368]	6,89	9,35	-2,46
HIST2H2AC	Homo sapiens histone cluster 2, H2ac (HIST2H2AC), mRNA [NM_003517]	10,72	13,19	-2,47
HIST2H2AA4	Homo sapiens histone cluster 2, H2aa4 (HIST2H2AA4), mRNA [NM_001040874]	7,96	10,43	-2,47
AI571129	AI571129 tn85e01,x1 NCI_CGAP_Ut2 Homo sapiens cDNA clone IMAGE:2176344 3', mRNA sequence [AI571129]	6,11	8,57	-2,47
BG950086	BG950086 CM1-CT0377-020101-710-g05 CT0377 Homo sapiens cDNA, mRNA sequence [BG950086]	5,57	8,05	-2,48
TLE3	Homo sapiens transducin-like enhancer of split 3 (E(sp1) homolog, Drosophila) (TLE3), mRNA [NM_005078]	7,1	9,6	-2,5
SLPI	Homo sapiens secretory leukocyte peptidase inhibitor (SLPI), mRNA [NM_003064]	6,55	9,05	-2,5
AJ227863	Homo sapiens partial mRNA; ID YG39-2B, [AJ227863]	8,46	10,96	-2,5
THC2690931	AF235005 suppression of tumorigenicity 16 protein {Homo sapiens} (exp=-1; wgp=0; cg=0), partial (13%) [THC2690931]	4,52	7,02	-2,5
JMY	Homo sapiens junction-mediating and regulatory protein (JMY), mRNA [NM_152405]	5,8	8,31	-2,51
ADAM9	Homo sapiens ADAM metallopeptidase domain 9 (meltrin gamma) (ADAM9), transcript variant 2, mRNA [NM_001005845]	5,89	8,42	-2,52
SOD2	Homo sapiens superoxide dismutase 2, mitochondrial (SOD2), nuclear gene encoding mitochondrial protein, transcript variant 2, mRNA [NM_001024465]	10,34	12,88	-2,54
PBEF1	Homo sapiens pre-B-cell colony enhancing factor 1 (PBEF1), mRNA [NM_005746]	9,47	12,01	-2,54
NUAK2	Homo sapiens NUAK family, SNF1-like kinase, 2 (NUAK2), mRNA [NM_030952]	6,79	9,33	-2,54
ELF4	Homo sapiens E74-like factor 4 (ets domain transcription factor) (ELF4), mRNA [NM_001421]	8,93	11,48	-2,54
BF089603	BF089603 CM2-HT0946-140900-364-f11 HT0946 Homo sapiens cDNA, mRNA sequence [BF089603]	6,09	8,64	-2,55
TRIB1	Homo sapiens tribbles homolog 1 (Drosophila) (TRIB1), mRNA [NM_025195]	5,34	7,9	-2,56
LRRC25	Homo sapiens leucine rich repeat containing 25 (LRRC25), mRNA [NM_145256]	7,33	9,89	-2,56

Supplementary Table 1
Morandi et al

ABL1	Homo sapiens v-abl Abelson murine leukemia viral oncogene homolog 1 (ABL1), transcript variant b, mRNA [NM_007313]	4,41	7,01	-2,6
PLK1	Homo sapiens polo-like kinase 1 (Drosophila) (PLK1), mRNA [NM_005030]	9,24	11,84	-2,6
MXD1	Homo sapiens MAX dimerization protein 1 (MXD1), mRNA [NM_002357]	7,36	9,96	-2,6
ROPN1L	Homo sapiens ropporin 1-like (ROPN1L), mRNA [NM_031916]	5,16	7,77	-2,61
PYGL	Homo sapiens phosphorylase, glycogen; liver (Hers disease, glycogen storage disease type VI) (PYGL), mRNA [NM_002863]	8,21	10,82	-2,61
CEBPE	Homo sapiens CCAAT/enhancer binding protein (C/EBP), epsilon (CEBPE), mRNA [NM_001805]	6,87	9,49	-2,62
BM547196	BM547196 AGENCOURT_6499364 NIH_MGC_124 Homo sapiens cDNA clone IMAGE:5730270 5', mRNA sequence [BM547196]	10,55	13,17	-2,62
FNDC3B	Homo sapiens fibronectin type III domain containing 3B, mRNA (cDNA clone IMAGE:3882800), complete cds, [BC012204]	4,53	7,16	-2,63
CEBPB	Homo sapiens CCAAT/enhancer binding protein (C/EBP), beta (CEBPB), mRNA [NM_005194]	11,96	14,62	-2,65
HIST2H2AB	Homo sapiens histone cluster 2, H2ab (HIST2H2AB), mRNA [NM_175065]	8,78	11,44	-2,66
RAC2	Homo sapiens ras-related C3 botulinum toxin substrate 2 (rho family, small GTP binding protein Rac2) (RAC2), mRNA [NM_002872]	10,14	12,81	-2,67
THC2670384	chr13:24775303-24775362	8,76	11,44	-2,68
TMC8	Homo sapiens mRNA for FLJ00400 protein, [AK090478]	7,63	10,32	-2,69
-	chr11:062379527-062379586	3,75	6,43	-2,69
ZCCHC6	Homo sapiens zinc finger, CCHC domain containing 6 (ZCCHC6), mRNA [NM_024617]	4,44	7,13	-2,7
UCN2	Homo sapiens urocortin 2 (UCN2), mRNA [NM_033199]	9,58	12,28	-2,7
THC2577566	chr8:030060416-030060475	9,79	12,49	-2,7
THC2517558	BC026690 CD97 antigen, isoform 2 precursor {Homo sapiens} (exp=-1; wgp=0; cg=0), partial (15%) [THC2517558]	6,64	9,33	-2,7
THC2539541	MUSTCAYK T-cell receptor alpha chain {Mus musculus} (exp=-1; wgp=0; cg=0), partial (11%) [THC2539541]	5,06	7,77	-2,71
THC2534530	AF235023 chromosome condensation protein G {Homo sapiens} (exp=0; wgp=1; cg=0), partial (3%) [THC2534530]	6,81	9,52	-2,71
SYTL1	Homo sapiens synaptotagmin-like 1 (SYTL1), mRNA [NM_032872]	6,77	9,49	-2,73
SUSD3	Homo sapiens sushi domain containing 3 (SUSD3), mRNA [NM_145006]	8,49	11,22	-2,73
CEBPA	Homo sapiens CCAAT/enhancer binding protein (C/EBP), alpha (CEBPA), mRNA [NM_004364]	11,22	13,97	-2,75
NCF4	Homo sapiens neutrophil cytosolic factor 4, 40kDa (NCF4), transcript variant 1, mRNA [NM_000631]	5,67	8,45	-2,78
NOD2	Homo sapiens nucleotide-binding oligomerization domain containing 2 (NOD2), mRNA [NM_022162]	5,01	7,81	-2,8
SLC30A1	Homo sapiens solute carrier family 30 (zinc transporter), member 1 (SLC30A1), mRNA [NM_021194]	6,77	9,58	-2,81
KIF23	Homo sapiens kinesin family member 23 (KIF23), transcript variant 1, mRNA [NM_138555]	8,42	11,23	-2,81
CDA	Homo sapiens cytidine deaminase (CDA), mRNA [NM_001785]	6,11	8,92	-2,81
THC2559929	Q9UP60_HUMAN (Q9UP60) SNC73 protein, partial (54%) [THC2559929]	7,82	10,64	-2,82

Supplementary Table 1
Morandi et al

IGHD	Homo sapiens cDNA FLJ46727 fis, clone TRACH3019058, highly similar to Ig delta chain C region, [AK128568]	4,2	7,02	-2,82
ITGAL	Homo sapiens integrin, alpha L (antigen CD11A (p180), lymphocyte function-associated antigen 1; alpha polypeptide) (ITGAL), mRNA [NM_002209]	5,68	8,52	-2,84
STXBP2	Homo sapiens syntaxin binding protein 2 (STXBP2), mRNA [NM_006949]	4,06	6,92	-2,86
LOC728965	Homo sapiens mRNA; cDNA DKFZp666D074 (from clone DKFZp666D074) [AL833005]	8,71	11,58	-2,87
HIST1H2AD	Homo sapiens histone cluster 1, H2ad (HIST1H2AD), mRNA [NM_021065]	10,41	13,29	-2,89
ENST0000032467	Protein phosphatase Slingshot homolog 2 (EC 3,1,3,48) (EC 3,1,3,16) (SSH-2L) (hSSH-2L), [Source:Uniprot/SWISSPROT;Acc:Q76I76] [ENST00000324677]	5,82	8,73	-2,9
OTUD1	Homo sapiens mRNA, clone: TH020D07, [AB188491]	5,51	8,42	-2,91
BST1	Homo sapiens bone marrow stromal cell antigen 1 (BST1), mRNA [NM_004334]	7,37	10,28	-2,91
MLC1	Homo sapiens megalencephalic leukoencephalopathy with subcortical cysts 1 (MLC1), transcript variant 1, mRNA [NM_015166]	5,98	8,92	-2,94
ID2	Homo sapiens inhibitor of DNA binding 2, dominant negative helix-loop-helix protein (ID2), mRNA [NM_002166]	9,28	12,22	-2,94
IKZF1	Homo sapiens PRO0758 mRNA, complete cds, [AF116605]	5,36	8,32	-2,96
ZNF206	Homo sapiens zinc finger protein 206 (ZNF206), mRNA [NM_032805]	9,68	12,65	-2,97
RAB11FIP1	Homo sapiens RAB11 family interacting protein 1 (class I) (RAB11FIP1), transcript variant 2, mRNA [NM_001002233]	6,69	9,66	-2,97
AF034187	Homo sapiens clone 2,2H12 Ndr Ser/Thr kinase-like protein mRNA, partial cds, [AF034187]	6,08	9,07	-2,99
SPI1	Homo sapiens spleen focus forming virus (SFFV) proviral integration oncogene spi1 (SPI1), mRNA [NM_003120]	4,34	7,37	-3,03
TAGAP	Homo sapiens T-cell activation GTPase activating protein (TAGAP), transcript variant 2, mRNA [NM_054114]	6,13	9,22	-3,09
FGR	Homo sapiens Gardner-Rasheed feline sarcoma viral (v-fgr) oncogene homolog (FGR), transcript variant 2, mRNA [NM_001042747]	5,02	8,15	-3,13
C6orf25	G6b protein precursor, [Source:Uniprot/SWISSPROT;Acc:O95866] [ENST00000375806]	5,36	8,49	-3,13
FAM129C	Homo sapiens family with sequence similarity 129, member C (FAM129C), mRNA [NM_173544]	5,88	9,01	-3,14
KLF6	Homo sapiens Kruppel-like factor 6 (KLF6), transcript variant 1, mRNA [NM_001008490]	5,45	8,61	-3,16
CST7	Homo sapiens cystatin F (leukocystatin) (CST7), mRNA [NM_003650]	4,61	7,78	-3,17
ICAM3	Homo sapiens intercellular adhesion molecule 3 (ICAM3), mRNA [NM_002162]	9,44	12,65	-3,2
C21orf96	Homo sapiens cDNA: FLJ20856 fis, clone ADKA01509, [AK024509]	4,46	7,66	-3,2
NFKBIZ	Homo sapiens nuclear factor of kappa light polypeptide gene enhancer in B-cells inhibitor, zeta (NFKBIZ), transcript variant 1, mRNA [NM_031419]	6,9	10,11	-3,21
APOBEC3B	Homo sapiens apolipoprotein B mRNA editing enzyme, catalytic polypeptide-like 3B (APOBEC3B), mRNA [NM_004900]	6,09	9,3	-3,21
LOC392617	PREDICTED: Homo sapiens similar to slit homolog 1 (LOC392617), mRNA [XM_374386]	9,8	13,02	-3,22
RIN3	Homo sapiens cDNA FLJ11700 fis, clone HEMBA1005050, [AK021762]	4,6	7,85	-3,25

Supplementary Table 1
Morandi et al

PTPRE	Homo sapiens protein tyrosine phosphatase, receptor type, E (PTPRE), transcript variant 1, mRNA [NM_006504]	7,37	10,63	-3,26
ANPEP	Homo sapiens alanyl (membrane) aminopeptidase (aminopeptidase N, aminopeptidase M, microsomal aminopeptidase, CD13, p150) (ANPEP), mRNA [NM_001150]	6,28	9,54	-3,26
CD69	Homo sapiens CD69 molecule (CD69), mRNA [NM_001781]	7,12	10,43	-3,3
LGALS12	Homo sapiens lectin, galactoside-binding, soluble, 12 (galectin 12) (LGALS12), mRNA [NM_033101]	4,68	8	-3,32
ZBTB24	Homo sapiens zinc finger and BTB domain containing 24 (ZBTB24), mRNA [NM_014797]	5,66	9,01	-3,35
LOC158830	Homo sapiens similar to Ab2-183 (LOC158830), mRNA [NM_001025265]	5,57	8,92	-3,35
IL8RB	Homo sapiens interleukin 8 receptor, beta (IL8RB), mRNA [NM_001557]	4,11	7,51	-3,4
G0S2	Homo sapiens G0/G1switch 2 (G0S2), mRNA [NM_015714]	9,49	12,89	-3,4
ZC3H12A	Homo sapiens zinc finger CCCH-type containing 12A (ZC3H12A), mRNA [NM_025079]	6,89	10,32	-3,42
PRG1	Homo sapiens proteoglycan 1, secretory granule (PRG1), mRNA [NM_002727]	10,65	14,12	-3,47
TMEM154	Homo sapiens transmembrane protein 154 (TMEM154), mRNA [NM_152680]	4,48	7,96	-3,48
KRT23	Homo sapiens keratin 23 (histone deacetylase inducible) (KRT23), mRNA [NM_015515]	4,4	7,9	-3,5
ENST0000035835(	Runt-related transcription factor 1 (Core-binding factor, alpha 2 subunit) (CBF-alpha 2) (Acute myeloid leukemia 1 protein) (Oncogene AML-1) (Polyomavirus enhancer-binding protein 2 alpha B subunit) (PEBP2-alpha B) (PEA2-alpha B) (SL3-3 enhancer,,,	4,71	8,22	-3,51
HDC	Homo sapiens histidine decarboxylase (HDC), mRNA [NM_002112]	4,2	7,73	-3,53
MYO1G	Homo sapiens mRNA for FLJ00121 protein, [AK074050]	5,22	8,82	-3,6
POU2AF1	Homo sapiens POU domain, class 2, associating factor 1 (POU2AF1), mRNA [NM_006235]	6,28	9,9	-3,63
P2RY2	Homo sapiens purinergic receptor P2Y, G-protein coupled, 2 (P2RY2), transcript variant 1, mRNA [NM_176072]	5,17	8,81	-3,64
LILRA5	Homo sapiens leukocyte immunoglobulin-like receptor, subfamily A (with TM domain), member 5 (LILRA5), transcript variant 2, mRNA [NM_181985]	4,64	8,32	-3,68
FCRL5	Homo sapiens Fc receptor-like 5 (FCRL5), mRNA [NM_031281]	4,87	8,55	-3,68
NCF1	Homo sapiens neutrophil cytosolic factor 1, (chronic granulomatous disease, autosomal 1) (NCF1), mRNA [NM_000265]	7,11	10,8	-3,69
CSTA	Homo sapiens cystatin A (stefin A) (CSTA), mRNA [NM_005213]	7,96	11,67	-3,71
NFE2	Homo sapiens nuclear factor (erythroid-derived 2), 45kDa (NFE2), mRNA [NM_006163]	6,93	10,66	-3,73
CXCL1	Homo sapiens chemokine (C-X-C motif) ligand 1 (melanoma growth stimulating activity, alpha) (CXCL1), mRNA [NM_001511]	6,78	10,52	-3,74
ALOX5AP	Homo sapiens arachidonate 5-lipoxygenase-activating protein (ALOX5AP), mRNA [NM_001629]	8,82	12,56	-3,74
VNN2	Homo sapiens vanin 2 (VNN2), transcript variant 1, mRNA [NM_004665]	7,06	10,83	-3,77
IGHM	human full-length cDNA clone CS0DD006YL02 of Neuroblastoma of Homo sapiens (human), [BX161420]	8,1	11,88	-3,78
C11orf21	Homo sapiens C11orf21 mRNA, complete cds, [AB029488]	6,21	9,99	-3,78

Supplementary Table 1
Morandi et al

STK17B	Homo sapiens serine/threonine kinase 17b (apoptosis-inducing) (STK17B), mRNA [NM_004226]	6,77	10,57	-3,8
IL18RAP	Homo sapiens interleukin 18 receptor accessory protein (IL18RAP), mRNA [NM_003853]	6,24	10,06	-3,82
SIGLEC5	Homo sapiens sialic acid binding Ig-like lectin 5 (SIGLEC5), mRNA [NM_003830]	6,05	9,9	-3,84
SLC16A3	Homo sapiens pp10472 mRNA, complete cds, [AF318321]	5,06	8,95	-3,89
IL1B	Homo sapiens interleukin 1, beta (IL1B), mRNA [NM_000576]	6,29	10,18	-3,89
CD37	Homo sapiens CD37 molecule (CD37), transcript variant 1, mRNA [NM_001774]	6,46	10,37	-3,91
FPR1	Homo sapiens formyl peptide receptor 1 (FPR1), mRNA [NM_002029]	7,27	11,19	-3,93
MGST1	Homo sapiens microsomal glutathione S-transferase 1 (MGST1), transcript variant 1c, mRNA [NM_145791]	5,28	9,33	-4,05
AV756170	AV756170 BM Homo sapiens cDNA clone BMFBGA09 5', mRNA sequence [AV756170]	5,25	9,32	-4,08
THC2541839	chr7:105693173-105693232	4,31	8,5	-4,2
CD19	Homo sapiens CD19 molecule (CD19), mRNA [NM_001770]	6,38	10,61	-4,23
FCN2	Homo sapiens ficolin (collagen/fibrinogen domain containing lectin) 2 (hucolin) (FCN2), transcript variant SV0, mRNA [NM_004108]	4,49	8,76	-4,27
SERPINB2	Plasminogen activator inhibitor 2 precursor (PAI-2) (Placental plasminogen activator inhibitor) (Monocyte Arg-serpin) (Urokinase inhibitor), [Source:Uniprot/SWISSPROT;Acc:P05120] [ENST00000299502]	5,35	9,63	-4,28
FCAR	Homo sapiens Fc fragment of IgA, receptor for (FCAR), transcript variant 1, mRNA [NM_002000]	3,72	8,07	-4,35
BLK	Homo sapiens B lymphoid tyrosine kinase (BLK), mRNA [NM_001715]	4,38	8,75	-4,37
FOLR3	Homo sapiens folate receptor 3 (gamma) (FOLR3), mRNA [NM_000804]	3,65	8,03	-4,38
LILRA3	Homo sapiens leukocyte immunoglobulin-like receptor, subfamily A (without TM domain), member 3 (LILRA3), mRNA [NM_006865]	5,03	9,48	-4,45
HSH2D	Homo sapiens hematopoietic SH2 domain containing (HSH2D), mRNA [NM_032855]	5,67	10,14	-4,47
LYZ	Homo sapiens lysozyme (renal amyloidosis) (LYZ), mRNA [NM_000239]	7,28	11,88	-4,6
RETN	Homo sapiens resistin (RETN), mRNA [NM_020415]	4,95	9,83	-4,88
AQP9	Homo sapiens aquaporin 9 (AQP9), mRNA [NM_020980]	5,65	10,63	-4,98
THC2530077	ALU5_HUMAN (P39192) Alu subfamily SC sequence contamination warning entry, partial (9%) [THC2530077]	7,33	12,37	-5,04
MNDA	Homo sapiens myeloid cell nuclear differentiation antigen (MNDA), mRNA [NM_002432]	7,1	12,16	-5,06
RGR	Homo sapiens Ral-GDS related protein Rgr (Rgr), mRNA [NM_153615]	6,8	11,87	-5,07
FCN1	Homo sapiens ficolin (collagen/fibrinogen domain containing) 1 (FCN1), mRNA [NM_002003]	6,64	11,93	-5,29
CD177	Homo sapiens CD177 molecule (CD177), mRNA [NM_020406]	3,87	9,17	-5,3
ENST0000031677[illegible]	Homo sapiens HSPC102 mRNA, partial cds, [AF161365]	3,65	8,99	-5,34
LOC643332	similar to Nonsecretory ribonuclease precursor (Ribonuclease US) (Eosinophil-derived neurotoxin) (RNase UpI-2) (Ribonuclease 2) (RNase 2) (LOC643332), mRNA [Source:RefSeq_dna;Acc:XR_016287] [ENST00000258817]	3,7	9,26	-5,57
CAMP	Homo sapiens cathelicidin antimicrobial peptide (CAMP), mRNA [NM_004345]	5,75	11,38	-5,63

Supplementary Table 1
Morandi et al

MPO	Homo sapiens myeloperoxidase (MPO), nuclear gene encoding mitochondrial protein, mRNA [NM_000250]	4,77	10,68	-5,91
BI026064	BI026064 CM0-MT0374-060201-774-h11 MT0374 Homo sapiens cDNA, mRNA sequence [BI026064]	4,83	10,85	-6,02
S100P	Homo sapiens S100 calcium binding protein P (S100P), mRNA [NM_005980]	6,39	12,44	-6,05
S100A8	Homo sapiens S100 calcium binding protein A8 (S100A8), mRNA [NM_002964]	8,53	15,03	-6,51
S100A9	Homo sapiens S100 calcium binding protein A9 (S100A9), mRNA [NM_002965]	6,06	12,98	-6,92
RNASE2	Homo sapiens ribonuclease, RNase A family, 2 (liver, eosinophil-derived neurotoxin) (RNASE2), mRNA [NM_002934]	3,64	10,89	-7,25

Supplemental Table 1
Morandi et al

GeneName	Description	MEAN expression stage 4_ALIVE	MEAN expression GD2+ cells	Difference of mean expression (stage 4_ALIVE - GD2+ cells)
FAM103A1	Homo sapiens family with sequence similarity 103, member A1 (FAM103A1), mRNA [NM_031452]	10.5	11,16	-0,67
DDX20	Homo sapiens DEAD (Asp-Glu-Ala-Asp) box polypeptide 20 (DDX20), mRNA [NM_007204]	9.2	9,89	-0,69
FKBP3	Homo sapiens FK506 binding protein 3, 25kDa (FKBP3), mRNA [NM_002013]	12.51	13,22	-0,71
COMMD1	Homo sapiens copper metabolism (Murr1) domain containing 1 (COMMD1), mRNA [NM_152516]	11.35	12,06	-0,71
C7orf28A	Homo sapiens chromosome 7 open reading frame 28A (C7orf28A), mRNA [NM_015622]	12.29	13,01	-0,72
DHX36	Homo sapiens DEAH (Asp-Glu-Ala-His) box polypeptide 36 (DHX36), mRNA [NM_020865]	10.69	11,42	-0,73
SNW1	Homo sapiens SNW domain containing 1 (SNW1), mRNA [NM_012245]	11.47	12,22	-0,76
USP16	Homo sapiens ubiquitin specific peptidase 16 (USP16), transcript variant 1, mRNA [NM_006447]	11.1	11,87	-0,77
SSNA1	Homo sapiens Sjogren's syndrome nuclear autoantigen 1 (SSNA1), mRNA [NM_003731]	10.68	11,45	-0,77
SNRPG	Homo sapiens small nuclear ribonucleoprotein polypeptide G (SNRPG), mRNA [NM_003096]	10.78	11,56	-0,78
RFWD2	Homo sapiens ring finger and WD repeat domain 2 (RFWD2), transcript variant 1, mRNA [NM_022457]	10.34	11,13	-0,79
BXDC5	Homo sapiens brix domain containing 5 (BXDC5), mRNA [NM_025065]	9.87	10,66	-0,79
PDCD7	Homo sapiens programmed cell death 7 (PDCD7), mRNA [NM_005707]	8.54	9,35	-0,80
MRP63	Homo sapiens mitochondrial ribosomal protein 63 (MRP63), nuclear gene encoding mitochondrial protein, mRNA [NM_024026]	12.2	13,00	-0,80
LARP2	Homo sapiens La ribonucleoprotein domain family, member 2 (LARP2), transcript variant 2, mRNA [NM_178043]	6.65	7,45	-0,80
COMMD4	Homo sapiens COMM domain containing 4 (COMMD4), mRNA [NM_017828]	8.93	9,72	-0,80
SLC25A32	Homo sapiens solute carrier family 25, member 32 (SLC25A32), mRNA [NM_030780]	9.96	10,77	-0,81
NUTF2	Homo sapiens nuclear transport factor 2 (NUTF2), mRNA [NM_005796]	11.15	11,96	-0,81
MTHFD1	Homo sapiens methylenetetrahydrofolate dehydrogenase (NADP+ dependent) 1, methenyltetrahydrofolate cyclohydrolase, formyltetrahydrofolate synthetase (MTHFD1), mRNA [NM_005956]	10.67	11,50	-0,83
PSMA3	Homo sapiens proteasome (prosome, macropain) subunit, alpha type, 3 (PSMA3), transcript variant 1, mRNA [NM_002788]	11.19	12,03	-0,84
MLX	Homo sapiens MAX-like protein X (MLX), transcript variant 3, mRNA [NM_170607]	9.31	10,15	-0,84
CLPX	Homo sapiens ClpX caseinolytic peptidase X homolog (E. coli) (CLPX), mRNA [NM_006660]	9.61	10,45	-0,84
TSN	Homo sapiens translin (TSN), mRNA [NM_004622]	10.02	10,87	-0,85
SF3B1	Homo sapiens splicing factor 3b, subunit 1, 155kDa (SF3B1), transcript variant 1, mRNA [NM_012433]	12.69	13,54	-0,85
NOLC1	Homo sapiens nucleolar and coiled-body phosphoprotein 1 (NOLC1), mRNA [NM_004741]	9.14	9,99	-0,85
MARK3	Homo sapiens MAP/microtubule affinity-regulating kinase 3 (MARK3), mRNA [NM_002376]	9.43	10,28	-0,85
CHCHD5	Homo sapiens coiled-coil-helix-coiled-coil-helix domain containing 5 (CHCHD5), mRNA [NM_032309]	8.85	9,69	-0,85
C18orf21	Homo sapiens chromosome 18 open reading frame 21 (C18orf21), mRNA [NM_031446]	10.5	11,35	-0,85
ACTL6A	Homo sapiens actin-like 6A (ACTL6A), transcript variant 3, mRNA [NM_178042]	10.97	11,82	-0,85
ZNF326	Homo sapiens zinc finger protein 326 (ZNF326), transcript variant 1, mRNA [NM_182976]	11.01	11,87	-0,86

Supplemental Table 1
Morandi et al

RG9MTD1	Homo sapiens RNA (guanine-9-) methyltransferase domain containing 1 (RG9MTD1), mRNA [NM_017819]	10.34	11,20	-0,86
PPWD1	Homo sapiens peptidylprolyl isomerase domain and WD repeat containing 1 (PPWD1), mRNA [NM_015342]	10.17	11,03	-0,86
CSE1L	Homo sapiens CSE1 chromosome segregation 1-like (yeast) (CSE1L), mRNA [NM_001316]	11.71	12,57	-0,86
C14orf153	Homo sapiens chromosome 14 open reading frame 153 (C14orf153), mRNA [NM_032374]	10.87	11,74	-0,86
AKAP8	Homo sapiens A kinase (PRKA) anchor protein 8 (AKAP8), mRNA [NM_005858]	10.35	11,20	-0,86
BE064950	BE064950 RC1-BT0313-130400-016-d09 BT0313 Homo sapiens cDNA, mRNA sequence [BE064950]	8.23	9,09	-0,86
ZNF317	Homo sapiens zinc finger protein 317 (ZNF317), mRNA [NM_020933]	8.91	9,78	-0,87
RAF1	Homo sapiens v-raf-1 murine leukemia viral oncogene homolog 1 (RAF1), mRNA [NM_002880]	10.47	11,34	-0,87
TIAL1	Homo sapiens TIA1 cytotoxic granule-associated RNA binding protein-like 1 (TIAL1), transcript variant 1, mRNA [NM_003252]	9.22	10,09	-0,87
SNRPD3	Homo sapiens small nuclear ribonucleoprotein D3 polypeptide 18kDa (SNRPD3), mRNA [NM_004175]	12.19	13,06	-0,87
RNUXA	Homo sapiens RNA U, small nuclear RNA export adaptor (phosphorylation regulated) (RNUXA), mRNA [NM_032177]	10.35	11,22	-0,87
PSMC2	Homo sapiens proteasome (prosome, macropain) 26S subunit, ATPase, 2 (PSMC2), mRNA [NM_002803]	11.63	12,50	-0,87
NFYC	Homo sapiens nuclear transcription factor Y, gamma (NFYC), mRNA [NM_014223]	8.12	8,99	-0,87
MSH2	Homo sapiens mutS homolog 2, colon cancer, nonpolyposis type 1 (E. coli) (MSH2), mRNA [NM_000251]	10.44	11,31	-0,87
MRPL53	Homo sapiens mitochondrial ribosomal protein L53 (MRPL53), nuclear gene encoding mitochondrial protein, mRNA [NM_053050]	11.24	12,10	-0,87
MAGOH	Homo sapiens mago-nashi homolog, proliferation-associated (Drosophila) (MAGOH), mRNA [NM_002370]	9.92	10,80	-0,87
FUSIP1	Homo sapiens FUS interacting protein (serine/arginine-rich) 1 (FUSIP1), transcript variant 1, mRNA [NM_006625]	10.72	11,59	-0,87
C18orf10	Homo sapiens chromosome 18 open reading frame 10 (C18orf10), mRNA [NM_015476]	9.4	10,26	-0,87
ATP5S	Homo sapiens ATP synthase, H+ transporting, mitochondrial F0 complex, subunit s (factor B) (ATP5S), nuclear gene encoding mitochondrial protein, transcript variant 3, mRNA [NM_015684]	8.37	9,24	-0,87
ZNF562	Homo sapiens zinc finger protein 562 (ZNF562), mRNA [NM_017656]	6.26	7,14	-0,88
IFRD2	Homo sapiens interferon-related developmental regulator 2 (IFRD2), mRNA [NM_006764]	9.7	10,59	-0,88
BTBD7	Homo sapiens BTB (POZ) domain containing 7 (BTBD7), transcript variant 2, mRNA [NM_018167]	6.76	7,64	-0,88
ZNF558	Homo sapiens zinc finger protein 558 (ZNF558), mRNA [NM_144693]	8.87	9,77	-0,89
STX10	Homo sapiens syntaxin 10 (STX10), mRNA [NM_003765]	9.19	10,08	-0,89
RFP2	Homo sapiens ret finger protein 2 (RFP2), transcript variant 3, mRNA [NM_213590]	10.16	11,05	-0,89
MRPS18C	Homo sapiens mitochondrial ribosomal protein S18C (MRPS18C), nuclear gene encoding mitochondrial protein, mRNA [NM_016067]	11.2	12,09	-0,89
PRO1853	Homo sapiens hypothetical protein PRO1853 (PRO1853), transcript variant 2, mRNA [NM_018607]	9.63	10,52	-0,89
C1orf41	Homo sapiens chromosome 1 open reading frame 41 (C1orf41), mRNA [NM_016126]	10.82	11,71	-0,89
ZRF1	Homo sapiens zuotin related factor 1 (ZRF1), mRNA [NM_014377]	11.3	12,20	-0,90
ZNF655	Homo sapiens zinc finger protein 655 (ZNF655), transcript variant 6, mRNA [NM_001009956]	10.33	11,22	-0,90
ZNF639	Homo sapiens zinc finger protein 639 (ZNF639), mRNA [NM_016331]	8.34	9,24	-0,90
SAP30L	Homo sapiens SAP30-like (SAP30L), mRNA [NM_024632]	10.19	11,09	-0,90

Supplemental Table 1
Morandi et al

PLEKHJ1	Homo sapiens pleckstrin homology domain containing, family J member 1 (PLEKHJ1), mRNA [NM_018049]	9.93	10,84	-0,90
OSBPL8	Homo sapiens oxysterol binding protein-like 8 (OSBPL8), transcript variant 1, mRNA [NM_020841]	9.2	10,11	-0,90
C16orf70	Homo sapiens chromosome 16 open reading frame 70 (C16orf70), mRNA [NM_025187]	9.07	9,97	-0,90
ATP5J2	Homo sapiens ATP synthase, H+ transporting, mitochondrial F0 complex, subunit F2 (ATP5J2), nuclear gene encoding mitochondrial protein, transcript variant 1, mRNA [NM_004889]	11.76	12,66	-0,90
SNRPD1	Homo sapiens small nuclear ribonucleoprotein D1 polypeptide 16kDa (SNRPD1), mRNA [NM_006938]	11.58	12,49	-0,91
DNAJB11	Homo sapiens DnaJ (Hsp40) homolog, subfamily B, member 11 (DNAJB11), mRNA [NM_016306]	11.45	12,36	-0,91
POP4	Homo sapiens processing of precursor 4, ribonuclease P/MRP subunit (S. cerevisiae) (POP4), mRNA [NM_006627]	10.51	11,42	-0,92
LZIC	Homo sapiens leucine zipper and CTNNBIP1 domain containing (LZIC), mRNA [NM_032368]	10.77	11,69	-0,92
MGC21675	Homo sapiens hypothetical protein MGC21675 (MGC21675), mRNA [NM_052861]	7.17	8,09	-0,92
FLJ10803	Homo sapiens hypothetical protein FLJ10803 (FLJ10803), mRNA [NM_018224]	9.13	10,05	-0,92
FANCM	Homo sapiens Fanconi anemia, complementation group M (FANCM), mRNA [NM_020937]	8.3	9,21	-0,92
DDX47	Homo sapiens DEAD (Asp-Glu-Ala-Asp) box polypeptide 47 (DDX47), transcript variant 1, mRNA [NM_016355]	12.49	13,41	-0,92
RPL26L1	Homo sapiens ribosomal protein L26-like 1 (RPL26L1), mRNA [NM_016093]	11.24	12,18	-0,93
NDUFV2	Homo sapiens NADH dehydrogenase (ubiquinone) flavoprotein 2, 24kDa (NDUFV2), mRNA [NM_021074]	11.94	12,87	-0,93
IMPA1	Homo sapiens inositol(myo)-1(or 4)-monophosphatase 1 (IMPA1), mRNA [NM_005536]	10.06	11,00	-0,93
ENST00000242224	Homo sapiens hypothetical protein MGC13098, mRNA (cDNA clone IMAGE:4074285), partial cds. [BC062722]	7.48	8,40	-0,93
C6orf49	Homo sapiens chromosome 6 open reading frame 49 (C6orf49), mRNA [NM_013397]	11.46	12,39	-0,93
C3orf17	Homo sapiens chromosome 3 open reading frame 17 (C3orf17), transcript variant 1, mRNA [NM_015412]	10.97	11,90	-0,93
C18orf25	Homo sapiens chromosome 18 open reading frame 25 (C18orf25), transcript variant 1, mRNA [NM_145055]	7.05	7,99	-0,93
YRDC	Homo sapiens yrdC domain containing (E. coli) (YRDC), mRNA [NM_024640]	10.51	11,45	-0,94
TRAF7	Homo sapiens TNF receptor-associated factor 7 (TRAF7), transcript variant 1, mRNA [NM_032271]	6.5	7,44	-0,94
RPS28	Homo sapiens ribosomal protein S28 (RPS28), mRNA [NM_001031]	13.04	13,98	-0,94
KIAA1008	Homo sapiens KIAA1008 (KIAA1008), mRNA [NM_014953]	7.36	8,30	-0,94
HNRPC	Homo sapiens heterogeneous nuclear ribonucleoprotein C (C1/C2) (HNRPC), transcript variant 1, mRNA [NM_031314]	12.69	13,63	-0,94
FBXO9	Homo sapiens F-box protein 9 (FBXO9), transcript variant 3, mRNA [NM_033481]	11,00	11,95	-0,94
CDK7	Homo sapiens cyclin-dependent kinase 7 (MO15 homolog, Xenopus laevis, cdk-activating kinase) (CDK7), mRNA [NM_001799]	10.6	11,54	-0,94
CPSF6	Homo sapiens cleavage and polyadenylation specific factor 6, 68kDa (CPSF6), mRNA [NM_007007]	9.07	10,01	-0,94
AK096674	Homo sapiens cDNA FLJ39355 fis, clone PEBLM2003426. [AK096674]	7.57	8,52	-0,94
CNOT1	Homo sapiens CCR4-NOT transcription complex, subunit 1 (CNOT1), transcript variant 1, mRNA [NM_016284]	8.36	9,30	-0,94
PPP1CC	Homo sapiens protein phosphatase 1, catalytic subunit, gamma isoform (PPP1CC), mRNA [NM_002710]	12.4	13,35	-0,95
PRKRA	Homo sapiens protein kinase, interferon-inducible double stranded RNA dependent activator (PRKRA), mRNA [NM_003690]	10.45	11,40	-0,95
POMZP3	Homo sapiens POM (POM121 homolog, rat) and ZP3 fusion (POMZP3), transcript variant 2, mRNA [NM_152992]	7.32	8,26	-0,95

Supplemental Table 1
Morandi et al

NHP2L1	Homo sapiens NHP2 non-histone chromosome protein 2-like 1 (S. cerevisiae) (NHP2L1), transcript variant 1, mRNA [NM_005008]	11.58	12,53	-0,95
MCTS1	Homo sapiens malignant T cell amplified sequence 1 (MCTS1), mRNA [NM_014060]	10.88	11,83	-0,95
DNM2	Homo sapiens dynamin 2 (DNM2), transcript variant 1, mRNA [NM_001005360]	9.65	10,60	-0,95
ACP1	Homo sapiens acid phosphatase 1, soluble (ACP1), transcript variant 3, mRNA [NM_004300]	11.23	12,18	-0,95
CRKL	Homo sapiens v-crk sarcoma virus CT10 oncogene homolog (avian)-like (CRKL), mRNA [NM_005207]	5.03	5,99	-0,96
UTP11L	Homo sapiens UTP11-like, U3 small nucleolar ribonucleoprotein, (yeast) (UTP11L), mRNA [NM_016037]	10.18	11,14	-0,96
UBPH	Homo sapiens ubiquitin-binding protein homolog (UBPH), mRNA [NM_019116]	9.29	10,25	-0,96
TCEA1	Homo sapiens transcription elongation factor A (SII), 1 (TCEA1), transcript variant 1, mRNA [NM_006756]	11.67	12,64	-0,96
MKLN1	Homo sapiens muskelin 1, intracellular mediator containing kelch motifs (MKLN1), mRNA [NM_013255]	7.45	8,40	-0,96
LOC150223	Homo sapiens hypothetical protein LOC150223 (LOC150223), transcript variant 2, mRNA [NM_001017965]	9.67	10,64	-0,96
FAM45A	Homo sapiens family with sequence similarity 45, member A (FAM45A), mRNA [NM_207009]	9.11	10,07	-0,96
CRIPT	Homo sapiens cysteine-rich PDZ-binding protein (CRIPT), mRNA [NM_014171]	10.56	11,52	-0,96
ABCB7	Homo sapiens ATP-binding cassette, sub-family B (MDR/TAP), member 7 (ABCB7), nuclear gene encoding mitochondrial protein, mRNA [NM_004299]	9.36	10,32	-0,96
SLC35B1	Homo sapiens solute carrier family 35, member B1 (SLC35B1), mRNA [NM_005827]	10.48	11,45	-0,97
RNF2	Homo sapiens ring finger protein 2 (RNF2), mRNA [NM_007212]	6.72	7,69	-0,97
PMPCA	Homo sapiens peptidase (mitochondrial processing) alpha (PMPCA), nuclear gene encoding mitochondrial protein, mRNA [NM_015160]	10.54	11,52	-0,97
TP53AP1	Homo sapiens mRNA for P53TG1-B, complete cds. [AB007456]	8.17	9,15	-0,97
ITCH	Homo sapiens itchy homolog E3 ubiquitin protein ligase (mouse) (ITCH), mRNA [NM_031483]	7.96	8,93	-0,97
IFT20	Homo sapiens intraflagellar transport 20 homolog (Chlamydomonas) (IFT20), mRNA [NM_174887]	9.46	10,43	-0,97
FBXL12	Homo sapiens F-box and leucine-rich repeat protein 12 (FBXL12), mRNA [NM_017703]	9.82	10,80	-0,97
EDC3	Homo sapiens enhancer of mRNA decapping 3 homolog (S. cerevisiae) (EDC3), mRNA [NM_025083]	7.38	8,35	-0,97
C1orf124	Homo sapiens chromosome 1 open reading frame 124 (C1orf124), transcript variant 1, mRNA [NM_032018]	6.31	7,28	-0,97
AK2	Homo sapiens adenylate kinase 2 (AK2), transcript variant AK2B, mRNA [NM_013411]	8.98	9,95	-0,97
ZNF561	Homo sapiens zinc finger protein 561 (ZNF561), mRNA [NM_152289]	7.42	8,40	-0,98
SF3B14	Homo sapiens splicing factor 3B, 14 kDa subunit (SF3B14), mRNA [NM_016047]	11.93	12,92	-0,98
ROCK1	Homo sapiens Rho-associated, coiled-coil containing protein kinase 1, mRNA (cDNA clone IMAGE:5269982), complete cds. [BC041849]	8.89	9,87	-0,98
PCID1	Homo sapiens PCI domain containing 1 (herpesvirus entry mediator) (PCID1), mRNA [NM_006360]	12.67	13,66	-0,98
MRPL47	Homo sapiens mitochondrial ribosomal protein L47 (MRPL47), nuclear gene encoding mitochondrial protein, transcript variant 1, mRNA [NM_020409]	11.18	12,16	-0,98
MDH2	Homo sapiens malate dehydrogenase 2, NAD (mitochondrial) (MDH2), mRNA [NM_005918]	12.63	13,61	-0,98
HBXIP	Homo sapiens hepatitis B virus x interacting protein (HBXIP), mRNA [NM_006402]	12.61	13,59	-0,98
HSPA14	Homo sapiens heat shock 70kDa protein 14 (HSPA14), transcript variant 1, mRNA [NM_016299]	9.63	10,61	-0,98

Supplemental Table 1
Morandi et al

CHCHD2	Homo sapiens coiled-coil-helix-coiled-coil-helix domain containing 2 (CHCHD2), mRNA [NM_016139]	12.75	13,73	-0,98
TMEM14B	Homo sapiens transmembrane protein 14B (TMEM14B), mRNA [NM_030969]	11.53	12,52	-0,99
MRPS14	Homo sapiens mitochondrial ribosomal protein S14 (MRPS14), nuclear gene encoding mitochondrial protein, mRNA [NM_022100]	9.34	10,33	-0,99
HDAC8	Homo sapiens histone deacetylase 8 (HDAC8), mRNA [NM_018486]	7.55	8,55	-0,99
GSTZ1	Homo sapiens glutathione transferase zeta 1 (maleylacetoacetate isomerase) (GSTZ1), transcript variant 1, mRNA [NM_145870]	8.8	9,79	-0,99
CDK4	Homo sapiens cyclin-dependent kinase 4 (CDK4), mRNA [NM_000075]	11.63	12,62	-0,99
SETD8	Homo sapiens SET domain containing (lysine methyltransferase) 8 (SETD8), mRNA [NM_020382]	6.52	7,51	-1,00
NUP88	Homo sapiens nucleoporin 88kDa (NUP88), mRNA [NM_002532]	10.33	11,33	-1,00
MRPL4	Homo sapiens mitochondrial ribosomal protein L4 (MRPL4), nuclear gene encoding mitochondrial protein, transcript variant 1, mRNA [NM_015956]	8.77	9,77	-1,00
KLHL18	Homo sapiens kelch-like 18 (Drosophila) (KLHL18), mRNA [NM_025010]	7.97	8,97	-1,00
C15orf15	Homo sapiens chromosome 15 open reading frame 15 (C15orf15), mRNA [NM_016304]	10.48	11,48	-1,00
ZDHHC20	Homo sapiens cDNA FLJ25952 fis, clone SYN00911. [AK098818]	11.02	12,02	-1,00
BC007968	Homo sapiens cDNA clone IMAGE:4304485. [BC007968]	5.24	6,24	-1,00
USP1	Homo sapiens ubiquitin specific peptidase 1 (USP1), transcript variant 1, mRNA [NM_003368]	10.72	11,73	-1,01
SNRPB2	Homo sapiens small nuclear ribonucleoprotein polypeptide B'' (SNRPB2), transcript variant 1, mRNA [NM_003092]	9.84	10,85	-1,01
OPA1	Homo sapiens optic atrophy 1 (autosomal dominant) (OPA1), nuclear gene encoding mitochondrial protein, transcript variant 8, mRNA [NM_130837]	7.98	8,99	-1,01
MRPS36	Homo sapiens mitochondrial ribosomal protein S36 (MRPS36), nuclear gene encoding mitochondrial protein, mRNA [NM_033281]	11.04	12,05	-1,01
HSPC023	Homo sapiens HSPC023 protein (HSPC023), mRNA [NM_014047]	9.56	10,57	-1,01
CHCHD5	Homo sapiens coiled-coil-helix-coiled-coil-helix domain containing 5 (CHCHD5), mRNA [NM_032309]	8.61	9,62	-1,01
CD641036	CD641036 AGENCOURT_14532748 NIH_MGC_191 Homo sapiens cDNA clone IMAGE:30415087 5', mRNA sequence [CD641036]	10.5	11,51	-1,01
SFRS11	Homo sapiens splicing factor, arginine/serine-rich 11 (SFRS11), mRNA [NM_004768]	11.94	12,96	-1,02
MGC70863	Homo sapiens similar to RPL23AP7 protein (MGC70863), transcript variant 1, mRNA [NM_203477]	12.15	13,16	-1,02
MGC71993	Homo sapiens similar to DNA segment, Chr 11, Brigham & Womens Genetics 0434 expressed (MGC71993), mRNA [NM_001004333]	11.25	12,28	-1,02
PPM1B	Homo sapiens protein phosphatase 1B (formerly 2C), magnesium-dependent, beta isoform (PPM1B), transcript variant 1, mRNA [NM_002706]	9.88	10,91	-1,02
PXMP4	Homo sapiens peroxisomal membrane protein 4, 24kDa (PXMP4), transcript variant 1, mRNA [NM_007238]	7.36	8,39	-1,02
OTUB1	Homo sapiens OTU domain, ubiquitin aldehyde binding 1 (OTUB1), transcript variant 1, mRNA [NM_017670]	9.41	10,42	-1,02
IMMP1L	Homo sapiens IMP1 inner mitochondrial membrane peptidase-like (S. cerevisiae) (IMMP1L), mRNA [NM_144981]	7.88	8,90	-1,02
COPS4	Homo sapiens COP9 constitutive photomorphogenic homolog subunit 4 (Arabidopsis) (COPS4), mRNA [NM_016129]	10.47	11,49	-1,02
CCDC72	Homo sapiens coiled-coil domain containing 72 (CCDC72), mRNA [NM_015933]	13.38	14,40	-1,02
CENPP	Homo sapiens centromere protein P (CENPP), mRNA [NM_001012267]	8.73	9,75	-1,02

ENST00000310344	chr7:142526877-142526818	12.99	14,01	-1,02
THC2306718	chr3:10309228-10309287	6.44	7,47	-1,02
BX346853	BX346853 BX346853 Homo sapiens NEUROBLASTOMA COT 50-NORMALIZED Homo sapiens cDNA clone CS0DD003YG22 5-PRIME, mRNA sequence [BX346853]	11.45	12,47	-1,02
SNRPA	Homo sapiens small nuclear ribonucleoprotein polypeptide A (SNRPA), mRNA [NM_004596]	10.34	11,38	-1,03
PPP2R2A	Homo sapiens protein phosphatase 2 (formerly 2A), regulatory subunit B (PR 52), alpha isoform (PPP2R2A), mRNA [NM_002717]	9.63	10,65	-1,03
LOC407835	Homo sapiens mitogen-activated protein kinase kinase 2 pseudogene (LOC407835) on chromosome 7 [NR_002144]	11.48	12,51	-1,03
LSM8	Homo sapiens LSM8 homolog, U6 small nuclear RNA associated (S. cerevisiae) (LSM8), mRNA [NM_016200]	10.75	11,78	-1,03
EXOSC8	Homo sapiens exosome component 8 (EXOSC8), mRNA [NM_181503]	10.61	11,64	-1,03
C14orf112	Homo sapiens chromosome 14 open reading frame 112 (C14orf112), mRNA [NM_016468]	12.3	13,34	-1,03
ANKRD13A	Homo sapiens ankyrin repeat domain 13A (ANKRD13A), mRNA [NM_033121]	9.77	10,80	-1,03
ENST00000240820	PREDICTED: Homo sapiens similar to peptidylprolyl isomerase A isoform 1 (LOC131691), mRNA [XM_929754]	10.94	11,99	-1,04
SLC25A20	Homo sapiens solute carrier family 25 (carnitine/acylcarnitine translocase), member 20 (SLC25A20), nuclear gene encoding mitochondrial protein, mRNA [NM_000387]	8.72	9,76	-1,04
SNRPB	Homo sapiens small nuclear ribonucleoprotein polypeptides B and B1 (SNRPB), transcript variant 1, mRNA [NM_198216]	10.05	11,09	-1,04
SNRPC	Homo sapiens small nuclear ribonucleoprotein polypeptide C (SNRPC), mRNA [NM_003093]	12.64	13,68	-1,04
SH3GL1	Homo sapiens SH3-domain GRB2-like 1 (SH3GL1), mRNA [NM_003025]	6.61	7,66	-1,04
RECQL	Homo sapiens RecQ protein-like (DNA helicase Q1-like) (RECQL), transcript variant 2, mRNA [NM_032941]	9.18	10,21	-1,04
RAE1	Homo sapiens RAE1 RNA export 1 homolog (S. pombe) (RAE1), transcript variant 2, mRNA [NM_001015885]	9.48	10,53	-1,04
PUS7	Homo sapiens pseudouridylate synthase 7 homolog (S. cerevisiae) (PUS7), mRNA [NM_019042]	9.83	10,87	-1,04
PPFIA1	Homo sapiens protein tyrosine phosphatase, receptor type, f polypeptide (PTPRF), interacting protein (liprin), alpha 1 (PPFIA1), transcript variant 1, mRNA [NM_177423]	7.16	8,20	-1,04
METTL6	Homo sapiens methyltransferase like 6 (METTL6), mRNA [NM_152396]	8.23	9,27	-1,04
METAP2	Homo sapiens methionyl aminopeptidase 2 (METAP2), mRNA [NM_006838]	12.62	13,66	-1,04
LOC96610	Homo sapiens hypothetical protein similar to KIAA0187 gene product (LOC96610), mRNA [NM_080926]	9.64	10,68	-1,04
DKFZP686A10121	Homo sapiens hypothetical protein (DKFZP686A10121), mRNA [NM_033107]	8.68	9,72	-1,04
C7orf28B	Homo sapiens chromosome 7 open reading frame 28B (C7orf28B), mRNA [NM_198097]	12.61	13,64	-1,04
CHRAC1	Homo sapiens chromatin accessibility complex 1 (CHRAC1), mRNA [NM_017444]	7.59	8,63	-1,04
-	chr15:076740862-076740802	11.05	12,10	-1,04
BC047708	Homo sapiens, clone IMAGE:5750141, mRNA. [BC047708]	6.39	7,44	-1,05
ZNF8	Homo sapiens zinc finger protein 8 (ZNF8), mRNA [NM_021089]	7.89	8,94	-1,05
WDR68	Homo sapiens WD repeat domain 68 (WDR68), mRNA [NM_005828]	8.7	9,75	-1,05
WDR36	Homo sapiens WD repeat domain 36 (WDR36), mRNA [NM_139281]	7.44	8,49	-1,05
USP39	Homo sapiens ubiquitin specific peptidase 39 (USP39), mRNA [NM_006590]	12.77	13,81	-1,05
TIMM9	Homo sapiens translocase of inner mitochondrial membrane 9 homolog (yeast) (TIMM9), mRNA [NM_012460]	11.14	12,19	-1,05
TXN	Homo sapiens thioredoxin (TXN), mRNA [NM_003329]	12.56	13,61	-1,05

Supplemental Table 1
Morandi et al

SMC1A	Homo sapiens structural maintenance of chromosomes 1A (SMC1A), mRNA [NM_006306]	9.83	10,88	-1,05
SEPHS1	Homo sapiens selenophosphate synthetase 1 (SEPHS1), mRNA [NM_012247]	10.43	11,47	-1,05
PMPCB	Homo sapiens peptidase (mitochondrial processing) beta (PMPCB), nuclear gene encoding mitochondrial protein, mRNA [NM_004279]	11.82	12,87	-1,05
MAPK6	Homo sapiens mitogen-activated protein kinase 6 (MAPK6), mRNA [NM_002748]	9.77	10,81	-1,05
DNTTIP1	Homo sapiens deoxynucleotidyltransferase, terminal, interacting protein 1 (DNTTIP1), mRNA [NM_052951]	11.21	12,26	-1,05
CCDC5	Homo sapiens coiled-coil domain containing 5 (spindle associated) (CCDC5), mRNA [NM_138443]	10.44	11,49	-1,05
C17orf49	Homo sapiens chromosome 17 open reading frame 49 (C17orf49), mRNA [NM_174893]	10.52	11,57	-1,05
ASNA1	Homo sapiens arsA arsenite transporter, ATP-binding, homolog 1 (bacterial) (ASNA1), mRNA [NM_004317]	8.99	10,03	-1,05
ASB7	Homo sapiens ankyrin repeat and SOCS box-containing 7 (ASB7), transcript variant 1, mRNA [NM_024708]	6.13	7,18	-1,05
BM979607	BM979607 UI-CF-DU1-adt-k-11-0-UI.s1 UI-CF-DU1 Homo sapiens cDNA clone UI-CF-DU1-adt-k-11-0-UI 3', mRNA sequence [BM979607]	7.15	8,20	-1,05
TDP1	Homo sapiens tyrosyl-DNA phosphodiesterase 1 (TDP1), transcript variant 1, mRNA [NM_018319]	9.28	10,34	-1,06
ZBED5	Homo sapiens transposon-derived Buster1 transposase-like protein gene (LOC58486), mRNA [NM_021211]	11.56	12,63	-1,06
MEA1	Homo sapiens male-enhanced antigen 1 (MEA1), mRNA [NM_014623]	11.67	12,73	-1,06
FAM29A	Homo sapiens family with sequence similarity 29, member A (FAM29A), mRNA [NM_017645]	8.5	9,56	-1,06
-	chrX:064959698-064959635	8.13	9,19	-1,06
RNF113A	Homo sapiens ring finger protein 113A (RNF113A), mRNA [NM_006978]	11.43	12,49	-1,07
PSEN1	Homo sapiens PSN1 gene, alternative transcript. [AJ008005]	6.2	7,27	-1,07
ING2	Homo sapiens inhibitor of growth family, member 2 (ING2), mRNA [NM_001564]	9.32	10,39	-1,07
HRB	Homo sapiens HIV-1 Rev binding protein (HRB), mRNA [NM_004504]	9.94	11,02	-1,07
HP1BP3	Homo sapiens heterochromatin protein 1, binding protein 3 (HP1BP3), mRNA [NM_016287]	8.15	9,22	-1,07
CDC42SE2	Homo sapiens CDC42 small effector 2 (CDC42SE2), transcript variant 1, mRNA [NM_020240]	8.28	9,35	-1,07
RARS	Homo sapiens arginyl-tRNA synthetase (RARS), mRNA [NM_002887]	12.62	13,69	-1,07
DNAJC8	full-length cDNA clone CS0DF007YK17 of Fetal brain of Homo sapiens (human). [CR619944]	8.88	9,96	-1,07
MAPK7	Homo sapiens mitogen-activated protein kinase 7 (MAPK7), transcript variant 1, mRNA [NM_139033]	9.93	11,01	-1,08
FAM33A	Homo sapiens family with sequence similarity 33, member A (FAM33A), mRNA [NM_182620]	7.05	8,13	-1,08
COPS8	Homo sapiens COP9 constitutive photomorphogenic homolog subunit 8 (Arabidopsis) (COPS8), transcript variant 2, mRNA [NM_198189]	10.22	11,30	-1,08
C20orf20	Homo sapiens chromosome 20 open reading frame 20 (C20orf20), mRNA [NM_018270]	6.67	7,75	-1,08
CGGBP1	Homo sapiens CGG triplet repeat binding protein 1 (CGGBP1), transcript variant 1, mRNA [NM_001008390]	10.75	11,83	-1,08
BG571083	BG571083 602591689F1 NIH_MGC_79 Homo sapiens cDNA clone IMAGE:4713911 5', mRNA sequence [BG571083]	4.5	5,57	-1,08
BE540788	601065075F1 NIH_MGC_10 Homo sapiens cDNA clone IMAGE:3451387 5', mRNA sequence [BE540788]	6.32	7,40	-1,08
TIPRL	Homo sapiens TIP41, TOR signalling pathway regulator-like (S. cerevisiae) (TIPRL), transcript variant 1, mRNA [NM_152902]	10.26	11,35	-1,09
SYNJ2BP	Homo sapiens synaptojanin 2 binding protein (SYNJ2BP), mRNA [NM_018373]	8.81	9,90	-1,09
SVH	Homo sapiens SVH protein (SVH), mRNA [NM_031905]	9.68	10,77	-1,09

Supplemental Table 1
Morandi et al

PPM1A	Homo sapiens protein phosphatase 1A (formerly 2C), magnesium-dependent, alpha isoform (PPM1A), transcript variant 2, mRNA [NM_177951]	6.89	7,98	-1,09
PTEN	Homo sapiens phosphatase and tensin homolog (mutated in multiple advanced cancers 1) (PTEN), mRNA [NM_000314]	9.01	10,11	-1,09
NDUFS4	Homo sapiens NADH dehydrogenase (ubiquinone) Fe-S protein 4, 18kDa (NADH-coenzyme Q reductase) (NDUFS4), mRNA [NM_002495]	12.11	13,21	-1,09
MRPL42	Homo sapiens mitochondrial ribosomal protein L42 (MRPL42), nuclear gene encoding mitochondrial protein, transcript variant 3, mRNA [NM_172178]	8.63	9,72	-1,09
EEF1E1	Homo sapiens eukaryotic translation elongation factor 1 epsilon 1 (EEF1E1), mRNA [NM_004280]	9.73	10,83	-1,09
COX4NB	Homo sapiens COX4 neighbor (COX4NB), mRNA [NM_006067]	11.38	12,47	-1,09
C1QBP	Homo sapiens complement component 1, q subcomponent binding protein (C1QBP), nuclear gene encoding mitochondrial protein, mRNA [NM_001212]	12.91	14,00	-1,09
BUB3	Homo sapiens BUB3 budding uninhibited by benzimidazoles 3 homolog (yeast) (BUB3), transcript variant 2, mRNA [NM_001007793]	10.53	11,62	-1,09
ALG9	Homo sapiens asparagine-linked glycosylation 9 homolog (S. cerevisiae, alpha- 1,2-mannosyltransferase) (ALG9), mRNA [NM_024740]	8.28	9,37	-1,09
-	chr5:056251958-056252017	7.5	8,59	-1,09
SNRPE	Homo sapiens small nuclear ribonucleoprotein polypeptide E (SNRPE), mRNA [NM_003094]	11.23	12,33	-1,10
RAP80	Homo sapiens receptor associated protein 80 (RAP80), mRNA [NM_016290]	11.86	12,96	-1,10
POP1	Homo sapiens processing of precursor 1, ribonuclease P/MRP subunit (S. cerevisiae) (POP1), mRNA [NM_015029]	7.6	8,69	-1,10
MTPN	Homo sapiens myotrophin (MTPN), mRNA [NM_145808]	11.02	12,12	-1,10
FKBP1A	Homo sapiens FK506 binding protein 1A, 12kDa (FKBP1A), transcript variant 12A, mRNA [NM_054014]	8.18	9,28	-1,10
ENST00000330227	chrX:112572103-112572162	5.85	6,95	-1,10
-	chr5:068665802-068665863	12.63	13,73	-1,10
ZDHHC3	Homo sapiens zinc finger, DHHC-type containing 3 (ZDHHC3), mRNA [NM_016598]	7.63	8,75	-1,11
TXNL4B	Homo sapiens thioredoxin-like 4B (TXNL4B), mRNA [NM_017853]	6.46	7,57	-1,11
FLJ11021	Homo sapiens similar to splicing factor, arginine/serine-rich 4 (FLJ11021), transcript variant 2, mRNA [NM_198261]	12.01	13,12	-1,11
RASA2	Homo sapiens RAS p21 protein activator 2 (RASA2), mRNA [NM_006506]	5.8	6,91	-1,11
C6orf206	Homo sapiens cDNA FLJ30845 fis, clone FEBRA2002727. [AK055407]	5.08	6,19	-1,11
CA438802	UI-H-DH0-aus-b-12-0-UI.s1 NCI_CGAP_DH0 Homo sapiens cDNA clone UI-H-DH0-aus-b-12-0-UI 3', mRNA sequence [CA438802]	5.84	6,96	-1,12
UCHL3	Homo sapiens ubiquitin carboxyl-terminal esterase L3 (ubiquitin thiolesterase) (UCHL3), mRNA [NM_006002]	9.42	10,54	-1,12
TFAM	Homo sapiens transcription factor A, mitochondrial (TFAM), mRNA [NM_003201]	9.05	10,18	-1,12
LOC374395	Homo sapiens similar to RIKEN cDNA 1810059G22 (LOC374395), mRNA [NM_199337]	7.26	8,38	-1,12
MAPK14	Homo sapiens mitogen-activated protein kinase 14 (MAPK14), transcript variant 3, mRNA [NM_139013]	5.76	6,88	-1,12
ITGB3BP	Homo sapiens integrin beta 3 binding protein (beta3-endonexin) (ITGB3BP), mRNA [NM_014288]	7.79	8,92	-1,12
EIF2B1	Homo sapiens eukaryotic translation initiation factor 2B, subunit 1 alpha, 26kDa (EIF2B1), mRNA [NM_001414]	11.61	12,73	-1,12
DRB1	Homo sapiens developmentally regulated RNA-binding protein 1 (DRB1), mRNA [NM_152945]	9.24	10,36	-1,12
DDT	Homo sapiens D-dopachrome tautomerase (DDT), mRNA [NM_001355]	12.25	13,37	-1,12
CREBBP	Homo sapiens CREB binding protein (Rubinstein-Taybi syndrome) (CREBBP), mRNA [NM_004380]	7.76	8,88	-1,12

Supplemental Table 1
Morandi et al

C7orf11	Homo sapiens chromosome 7 open reading frame 11 (C7orf11), mRNA [NM_138701]	10.89	12,01	-1,12
SWS1	Homo sapiens cDNA clone IMAGE:5312898. [BC066344]	9.98	11,10	-1,12
ANKRD49	Homo sapiens ankyrin repeat domain 49 (ANKRD49), mRNA [NM_017704]	8.49	9,62	-1,12
ZNHIT3	Homo sapiens zinc finger, HIT type 3 (ZNHIT3), transcript variant 2, mRNA [NM_004773]	12.97	14,09	-1,13
TXNL2	Homo sapiens thioredoxin-like 2 (TXNL2), mRNA [NM_006541]	10.45	11,58	-1,13
SLBP	Homo sapiens stem-loop (histone) binding protein (SLBP), mRNA [NM_006527]	10.44	11,57	-1,13
SRP9	Homo sapiens signal recognition particle 9kDa (SRP9), mRNA [NM_003133]	10.66	11,79	-1,13
PSMD8	Homo sapiens proteasome (prosome, macropain) 26S subunit, non-ATPase, 8 (PSMD8), mRNA [NM_002812]	12.07	13,19	-1,13
ORMDL2	Homo sapiens ORM1-like 2 (S. cerevisiae) (ORMDL2), mRNA [NM_014182]	10.57	11,69	-1,13
MLH1	Homo sapiens mutL homolog 1, colon cancer, nonpolyposis type 2 (E. coli) (MLH1), mRNA [NM_000249]	11.72	12,84	-1,13
MRPL39	Homo sapiens mitochondrial ribosomal protein L39 (MRPL39), nuclear gene encoding mitochondrial protein, transcript variant 1, mRNA [NM_017446]	9.52	10,65	-1,13
DHPS	Homo sapiens deoxyhypusine synthase (DHPS), transcript variant 3, mRNA [NM_013407]	9.54	10,67	-1,13
FLJ13195	Homo sapiens cDNA FLJ13195 fis, clone NT2RP3004424, weakly similar to Homo sapiens mRNA for stromal antigen 3 (STAG3 gene). [AK023257]	6.11	7,24	-1,13
BC023989	Homo sapiens cDNA clone IMAGE:3931276, partial cds. [BC023989]	7.74	8,87	-1,13
CDK5RAP2	Homo sapiens CDK5 regulatory subunit associated protein 2 (CDK5RAP2), transcript variant 1, mRNA [NM_018249]	9.84	10,97	-1,13
THC2370457	ALU1_HUMAN (P39188) Alu subfamily J sequence contamination warning entry, partial (3%) [THC2370439]	8.88	10,01	-1,13
LOC440309	PREDICTED: Homo sapiens hypothetical LOC440309 (LOC440309), mRNA [XM_498623]	11.29	12,43	-1,14
YPEL5	Homo sapiens yippee-like 5 (Drosophila) (YPEL5), mRNA [NM_016061]	11.34	12,48	-1,14
NGLY1	Homo sapiens N-glycanase 1 (NGLY1), mRNA [NM_018297]	10.94	12,09	-1,14
HMGN2	Homo sapiens high-mobility group nucleosomal binding domain 2 (HMGN2), mRNA [NM_005517]	13.03	14,16	-1,14
CLEC2D	Homo sapiens C-type lectin domain family 2, member D (CLEC2D), transcript variant 2, mRNA [NM_001004419]	11.74	12,88	-1,14
C14orf166	Homo sapiens chromosome 14 open reading frame 166 (C14orf166), mRNA [NM_016039]	13.46	14,60	-1,14
FAM122C	Homo sapiens cDNA FLJ33292 fis, clone BNGH42000130, weakly similar to Homo sapiens beta glucuronidase isoform. [AK090611]	5.77	6,92	-1,14
BC037740	Homo sapiens cDNA clone IMAGE:5263531. [BC037740]	8.73	9,87	-1,14
-	chr17:035777834-035777893	11.08	12,22	-1,14
XRCC6BP1	Homo sapiens XRCC6 binding protein 1 (XRCC6BP1), mRNA [NM_033276]	8.32	9,47	-1,15
WDR26	Homo sapiens WD repeat domain 26 (WDR26), mRNA [NM_025160]	10.23	11,38	-1,15
TIMM17A	Homo sapiens translocase of inner mitochondrial membrane 17 homolog A (yeast) (TIMM17A), mRNA [NM_006335]	12.39	13,54	-1,15
SIAH2	Homo sapiens seven in absentia homolog 2 (Drosophila) (SIAH2), mRNA [NM_005067]	8.31	9,46	-1,15
PSMB3	Homo sapiens proteasome (prosome, macropain) subunit, beta type, 3 (PSMB3), mRNA [NM_002795]	11.57	12,73	-1,15
PSMC4	Homo sapiens proteasome (prosome, macropain) 26S subunit, ATPase, 4 (PSMC4), transcript variant 1, mRNA [NM_006503]	10.57	11,73	-1,15
SMG1	Homo sapiens PI-3-kinase-related kinase SMG-1 (SMG1), mRNA [NM_015092]	7.2	8,35	-1,15
MRPL54	Homo sapiens mitochondrial ribosomal protein L54 (MRPL54), nuclear gene encoding mitochondrial protein, mRNA [NM_172251]	10.19	11,33	-1,15

Supplemental Table 1
Morandi et al

MTF1	Homo sapiens metal-regulatory transcription factor 1 (MTF1), mRNA [NM_005955]	10.49	11,64	-1,15
ENY2	Homo sapiens enhancer of yellow 2 homolog (Drosophila) (ENY2), mRNA [NM_020189]	11.47	12,62	-1,15
C17orf37	Homo sapiens chromosome 17 open reading frame 37 (C17orf37), mRNA [NM_032339]	11.9	13,05	-1,15
C14orf10	Homo sapiens chromosome 14 open reading frame 10 (C14orf10), mRNA [NM_017917]	11.46	12,62	-1,15
BC053632	Homo sapiens cDNA clone IMAGE:6500775, partial cds. [BC053632]	6.91	8,07	-1,15
BRWD3	Homo sapiens bromodomain and WD repeat domain containing 3 (BRWD3), mRNA [NM_153252]	4.99	6,14	-1,15
SEP15	Homo sapiens 15 kDa selenoprotein (SEP15), transcript variant 1, mRNA [NM_004261]	12.28	13,44	-1,15
-	chrX:040964554-040964495	4.44	5,58	-1,15
-	chr4:120736792-120736849	4.92	6,07	-1,15
ZIK1	Homo sapiens zinc finger protein interacting with K protein 1 (ZIK1), mRNA [NM_001010879]	7.27	8,44	-1,16
UCK2	Homo sapiens uridine-cytidine kinase 2 (UCK2), mRNA [NM_012474]	10.06	11,23	-1,16
TMEM85	Homo sapiens transmembrane protein 85 (TMEM85), mRNA [NM_016454]	11.78	12,94	-1,16
TMEM161B	Homo sapiens transmembrane protein 161B (TMEM161B), mRNA [NM_153354]	8.84	10,00	-1,16
TDG	Homo sapiens thymine-DNA glycosylase (TDG), mRNA [NM_003211]	11.05	12,21	-1,16
SRPK1	Homo sapiens SFRS protein kinase 1 (SRPK1), mRNA [NM_003137]	11.47	12,63	-1,16
SDCCAG3	Homo sapiens serologically defined colon cancer antigen 3 (SDCCAG3), transcript variant 2, mRNA [NM_006643]	10.14	11,30	-1,16
KLHDC2	Homo sapiens kelch domain containing 2 (KLHDC2), mRNA [NM_014315]	10.85	12,01	-1,16
FLJ10213	Homo sapiens hypothetical protein FLJ10213 (FLJ10213), mRNA [NM_018029]	9.92	11,08	-1,16
HIGD2A	Homo sapiens HIG1 domain family, member 2A (HIGD2A), mRNA [NM_138820]	9.82	10,98	-1,16
ENST00000332361	GB|AE006463.1|AAK61228.1 60S ribosomal protein L23A like [Homo sapiens] [NP366270]	13.12	14,27	-1,16
-	chr7:043087125-043087184	12.04	13,20	-1,16
LOC647065	PREDICTED: Homo sapiens hypothetical protein LOC647065 (LOC647065), mRNA [XM_934270]	8.48	9,65	-1,17
ZFX	Homo sapiens zinc finger protein, X-linked (ZFX), mRNA [NM_003410]	4.42	5,58	-1,17
TBC1D7	Homo sapiens TBC1 domain family, member 7 (TBC1D7), mRNA [NM_016495]	10.46	11,63	-1,17
SFRS2	Homo sapiens splicing factor, arginine/serine-rich 2 (SFRS2), mRNA [NM_003016]	11.92	13,09	-1,17
SH3GLB1	Homo sapiens SH3-domain GRB2-like endophilin B1 (SH3GLB1), mRNA [NM_016009]	10.91	12,08	-1,17
RAN	Homo sapiens RAN, member RAS oncogene family (RAN), mRNA [NM_006325]	8.4	9,57	-1,17
LOC163131	Homo sapiens hypothetical BC331191_1 (LOC163131), mRNA [NM_001005851]	7.14	8,31	-1,17
E2F3	Homo sapiens E2F transcription factor 3 (E2F3), mRNA [NM_001949]	10.12	11,29	-1,17
C11orf51	Homo sapiens chromosome 11 open reading frame 51 (C11orf51), mRNA [NM_014042]	8.04	9,21	-1,17
AOF1	Homo sapiens amine oxidase (flavin containing) domain 1 (AOF1), mRNA [NM_153042]	5.65	6,82	-1,17
THC2377221	ALU2_HUMAN (P39189) Alu subfamily SB sequence contamination warning entry, partial (4%) [THC2377221]	7.03	8,20	-1,17
POLR2D	Homo sapiens polymerase (RNA) II (DNA directed) polypeptide D (POLR2D), mRNA [NM_004805]	9.3	10,48	-1,18
LOC128977	Homo sapiens hypothetical protein LOC128977 (LOC128977), mRNA [NM_173793]	8.96	10,15	-1,18
CCNK	Homo sapiens cyclin K (CCNK), mRNA [NM_003858]	7.93	9,10	-1,18

Supplemental Table 1
Morandi et al

CTDSPL2	Homo sapiens CTD (carboxy-terminal domain, RNA polymerase II, polypeptide A) small phosphatase like 2 (CTDSPL2), mRNA [NM_016396]	7.67	8,85	-1,18
C14orf2	Homo sapiens chromosome 14 open reading frame 2 (C14orf2), mRNA [NM_004894]	10.8	11,98	-1,18
AGPAT5	Homo sapiens 1-acylglycerol-3-phosphate O-acyltransferase 5 (lysophosphatidic acid acyltransferase, epsilon) (AGPAT5), mRNA [NM_018361]	7.65	8,83	-1,18
YY1	Homo sapiens YY1 transcription factor (YY1), mRNA [NM_003403]	10.19	11,38	-1,19
UQCRC1	Homo sapiens ubiquinol-cytochrome c reductase core protein I (UQCRC1), mRNA [NM_003365]	11.68	12,88	-1,19
SNX5	Homo sapiens sorting nexin 5 (SNX5), transcript variant 2, mRNA [NM_014426]	11,00	12,19	-1,19
HSPA4	Homo sapiens heat shock 70kDa protein 4 (HSPA4), transcript variant 1, mRNA [NM_002154]	12.39	13,58	-1,19
AF086286	Homo sapiens full length insert cDNA clone ZD47C12. [AF086286]	6.65	7,85	-1,19
DLEU8	Homo sapiens deleted in lymphocytic leukemia 8 (DLEU8), mRNA [NM_024570]	8.75	9,93	-1,19
C10orf137	Homo sapiens chromosome 10 open reading frame 137 (C10orf137), mRNA [NM_015608]	4.91	6,09	-1,19
CSNK1A1	Homo sapiens casein kinase 1, alpha 1 (CSNK1A1), transcript variant 2, mRNA [NM_001892]	10.12	11,31	-1,19
IMPDH1	Human IMP dehydrogenase type 1 mRNA complete cds. [J05272]	5.11	6,31	-1,20
ZDHHC12	Homo sapiens zinc finger, DHHC-type containing 12 (ZDHHC12), mRNA [NM_032799]	7.79	8,99	-1,20
ZCCHC8	Homo sapiens zinc finger, CCHC domain containing 8 (ZCCHC8), mRNA [NM_017612]	10.21	11,40	-1,20
SMC5	Homo sapiens structural maintenance of chromosomes 5 (SMC5), mRNA [NM_015110]	7.19	8,38	-1,20
SON	Homo sapiens SON DNA binding protein (SON), transcript variant b, mRNA [NM_032195]	7.5	8,70	-1,20
RCBTB1	Homo sapiens regulator of chromosome condensation (RCC1) and BTB (POZ) domain containing protein 1 (RCBTB1), mRNA [NM_018191]	5.2	6,40	-1,20
ODF2	Homo sapiens outer dense fiber of sperm tails 2 (ODF2), transcript variant 1, mRNA [NM_002540]	9.09	10,29	-1,20
NOLA2	Homo sapiens nucleolar protein family A, member 2 (H/ACA small nucleolar RNPs) (NOLA2), transcript variant 1, mRNA [NM_017838]	11.56	12,75	-1,20
LOC646200	PREDICTED: Homo sapiens similar to 60S ribosomal protein L22 (Heparin binding protein HBp15), transcript variant 1 (LOC646200), mRNA [XM_930460]	12.7	13,91	-1,21
AA807805	nu88h02.s1 NCI_CGAP_Alv1 Homo sapiens cDNA clone IMAGE:1217811, mRNA sequence [AA807805]	11.5	12,71	-1,21
ZNF256	Homo sapiens zinc finger protein 256 (ZNF256), mRNA [NM_005773]	8.62	9,83	-1,21
SNF8	Homo sapiens SNF8, ESCRT-II complex subunit, homolog (S. cerevisiae) (SNF8), mRNA [NM_007241]	12.94	14,15	-1,21
SAR1B	Homo sapiens SAR1 gene homolog B (S. cerevisiae) (SAR1B), transcript variant 2, mRNA [NM_016103]	8.64	9,85	-1,21
PRPF18	Homo sapiens PRP18 pre-mRNA processing factor 18 homolog (S. cerevisiae) (PRPF18), mRNA [NM_003675]	10.37	11,58	-1,21
HNRPA3	Homo sapiens heterogeneous nuclear ribonucleoprotein A3 (HNRPA3), mRNA [NM_194247]	12.79	14,00	-1,21
GRPEL2	Homo sapiens GrpE-like 2, mitochondrial (E. coli) (GRPEL2), nuclear gene encoding mitochondrial protein, mRNA [NM_152407]	8.07	9,28	-1,21
BRP44	Homo sapiens brain protein 44 (BRP44), mRNA [NM_015415]	11.19	12,41	-1,21
BTG1	Homo sapiens B-cell translocation gene 1, anti-proliferative (BTG1), mRNA [NM_001731]	11.76	12,97	-1,21
ARD1A	Homo sapiens ARD1 homolog A, N-acetyltransferase (S. cerevisiae) (ARD1A), mRNA [NM_003491]	9.55	10,76	-1,21
-	chr5:133445380-133445321	4.31	5,52	-1,21
-	chr18:004993089-004993026	8.36	9,57	-1,21

Supplemental Table 1
Morandi et al

ENST00000340534	PREDICTED: Homo sapiens similar to peptidylprolyl isomerase A isoform 1 (LOC128192), mRNA [XM_060887]	9.7	10,92	-1,22
PEX13	Homo sapiens peroxisome biogenesis factor 13 (PEX13), mRNA [NM_002618]	8.55	9,77	-1,22
ING3	Homo sapiens inhibitor of growth family, member 3 (ING3), transcript variant 1, mRNA [NM_019071]	10.49	11,71	-1,22
EIF3S2	Homo sapiens eukaryotic translation initiation factor 3, subunit 2 beta, 36kDa (EIF3S2), mRNA [NM_003757]	11.71	12,93	-1,22
BUD31	Homo sapiens BUD31 homolog (yeast) (BUD31), mRNA [NM_003910]	10.98	12,20	-1,22
C12orf47	full-length cDNA clone CS0DJ012YG18 of T cells (Jurkat cell line) Cot 10-normalized of Homo sapiens (human). [CR620831]	9.08	10,30	-1,22
ENST00000332498	chr8:33946689-33946630	10.17	11,39	-1,22
-	chr8:026491575-026491516	8.64	9,86	-1,22
ZP3	Homo sapiens zona pellucida glycoprotein 3 (sperm receptor) (ZP3), mRNA [NM_007155]	9.55	10,78	-1,23
ZNF207	Homo sapiens zinc finger protein 207 (ZNF207), transcript variant 1, mRNA [NM_003457]	9.74	10,97	-1,23
BMSC-MCP	Homo sapiens PNC1 protein (BMSC-MCP), mRNA [NM_032315]	10.64	11,87	-1,23
LRIG2	Homo sapiens leucine-rich repeats and immunoglobulin-like domains 2 (LRIG2), mRNA [NM_014813]	7.54	8,78	-1,23
JTB	Homo sapiens jumping translocation breakpoint (JTB), mRNA [NM_006694]	12.54	13,78	-1,23
C17orf39	Homo sapiens chromosome 17 open reading frame 39 (C17orf39), mRNA [NM_024052]	6.81	8,05	-1,23
SBNO1	Homo sapiens cDNA FLJ23676 fis, clone HEP08548, highly similar to Homo sapiens mRNA for MOP-3. [AK074256]	5.84	7,07	-1,23
AK000901	Homo sapiens cDNA FLJ10039 fis, clone HEMBA1000975. [AK000901]	6.47	7,69	-1,23
BOLA2	Homo sapiens bolA-like 2 (E. coli) (BOLA2), transcript variant 2, mRNA [NM_001031833]	9.93	11,16	-1,23
ARIH1	Homo sapiens ariadne homolog, ubiquitin-conjugating enzyme E2 binding protein, 1 (Drosophila) (ARIH1), mRNA [NM_005744]	6.77	7,99	-1,23
-	chr6:160147507-160147448	9.14	10,37	-1,23
-	chr19:057338148-057338089	8.61	9,84	-1,23
-	chr14:103216468-103216409	8.59	9,82	-1,23
AV645774	AV645774 AV645774 GLC Homo sapiens cDNA clone GLCAEF02 3', mRNA sequence [AV645774]	5.82	7,04	-1,23
AI457687	AI457687 tj48g10.x1 Soares_NSF_F8_9W_OT_PA_P_S1 Homo sapiens cDNA clone IMAGE:2144802 3' similar to TR:O22899 O22899 RNA HELICASE ISOLOG. ;, mRNA sequence [AI457687]	4.98	6,21	-1,23
AA768672	AA768672 oa67h04.s1 NCI_CGAP_GCB1 Homo sapiens cDNA clone IMAGE:1317367 3', mRNA sequence [AA768672]	5.68	6,91	-1,23
ZNF614	Homo sapiens zinc finger protein 614 (ZNF614), mRNA [NM_025040]	6.54	7,78	-1,24
WDHD1	Homo sapiens WD repeat and HMG-box DNA binding protein 1 (WDHD1), transcript variant 1, mRNA [NM_007086]	7.68	8,92	-1,24
TFRC	Homo sapiens transferrin receptor (p90, CD71) (TFRC), mRNA [NM_003234]	9.84	11,08	-1,24
PPARBP	Homo sapiens PPAR binding protein, mRNA (cDNA clone IMAGE:4822636), complete cds. [BC060758]	8.3	9,54	-1,24
PEX10	Homo sapiens peroxisome biogenesis factor 10 (PEX10), transcript variant 2, mRNA [NM_002617]	6.15	7,39	-1,24
MCM6	Homo sapiens MCM6 minichromosome maintenance deficient 6 (MIS5 homolog, S. pombe) (S. cerevisiae) (MCM6), mRNA [NM_005915]	11.53	12,76	-1,24
KIAA1143	Homo sapiens KIAA1143 (KIAA1143), mRNA [NM_020696]	8.58	9,81	-1,24
EEF1B2	Homo sapiens eukaryotic translation elongation factor 1 beta 2 (EEF1B2), transcript variant 1, mRNA [NM_001959]	12.83	14,07	-1,24
DHX15	Homo sapiens DEAH (Asp-Glu-Ala-His) box polypeptide 15 (DHX15), mRNA [NM_001358]	10.98	12,22	-1,24

Supplemental Table 1
Morandi et al

2'-PDE	Homo sapiens 2'-phosphodiesterase (2'-PDE), mRNA [NM_177966]	6.27	7,51	-1,24
-	chr6:150391611-150391674	10.52	11,76	-1,24
-	chr18:055642588-055642973	13.03	14,27	-1,24
-	chr15:033201170-033201111	11.03	12,28	-1,24
BX095281	BX095281 Soares melanocyte 2NbHM Homo sapiens cDNA clone IMAGp998E03596, mRNA sequence [BX095281]	6.34	7,57	-1,24
BQ008507	BQ008507 UI-H-ED1-ayk-d-19-0-UI.s1 NCI_CGAP_ED1 Homo sapiens cDNA clone IMAGE:5839842 3', mRNA sequence [BQ008507]	4.69	5,93	-1,24
ENST00000333731	PREDICTED: Homo sapiens similar to eukaryotic translation initiation factor 3, subunit 5 (epsilon) (LOC390282), mRNA [XM_372447]	11.47	12,71	-1,25
ZNF628	Homo sapiens zinc finger protein 628 (ZNF628), mRNA [NM_033113]	8.32	9,56	-1,25
TALDO1	Homo sapiens transaldolase 1 (TALDO1), mRNA [NM_006755]	10.91	12,16	-1,25
RBM25	Homo sapiens RNA binding motif protein 25 (RBM25), mRNA [NM_021239]	10.19	11,44	-1,25
RLF	Homo sapiens rearranged L-myc fusion (RLF), mRNA [NM_012421]	9.34	10,58	-1,25
PSRC1	Homo sapiens proline/serine-rich coiled-coil 1 (PSRC1), transcript variant 1, mRNA [NM_032636]	8.36	9,60	-1,25
IREB2	Homo sapiens iron-responsive element binding protein 2 (IREB2), mRNA [NM_004136]	6.9	8,15	-1,25
HBP1	Homo sapiens HMG-box transcription factor 1 (HBP1), mRNA [NM_012257]	7.7	8,95	-1,25
MTHFSD	Homo sapiens cDNA FLJ43025 fis, clone BRTHA2018707. [AK125015]	7.31	8,56	-1,25
CSNK1G3	Homo sapiens casein kinase 1, gamma 3 (CSNK1G3), transcript variant 1, mRNA [NM_004384]	6.95	8,20	-1,25
BCL2A1	Homo sapiens BCL2-related protein A1 (BCL2A1), mRNA [NM_004049]	10.51	11,76	-1,25
ADAR	Homo sapiens adenosine deaminase, RNA-specific (ADAR), transcript variant 1, mRNA [NM_001111]	6.1	7,35	-1,25
ANP32E	Homo sapiens acidic (leucine-rich) nuclear phosphoprotein 32 family, member E (ANP32E), mRNA [NM_030920]	7.48	8,72	-1,25
-	chr5:173873675-173873736	11.03	12,28	-1,25
PPIL5	Homo sapiens peptidylprolyl isomerase (cyclophilin)-like 5 (PPIL5), transcript variant 3, mRNA [NM_203467]	6.15	7,42	-1,26
MGC5242	Homo sapiens hypothetical protein MGC5242 (MGC5242), mRNA [NM_024033]	8.91	10,17	-1,26
GNA12	Homo sapiens guanine nucleotide binding protein (G protein) alpha 12 (GNA12), mRNA [NM_007353]	4.68	5,94	-1,26
DOCK7	Homo sapiens dedicator of cytokinesis 7 (DOCK7), mRNA [NM_033407]	5.31	6,57	-1,26
CCNT2	Homo sapiens cyclin T2 (CCNT2), transcript variant b, mRNA [NM_058241]	5.91	7,17	-1,26
ANKRD2	Homo sapiens ankyrin repeat domain 2 (stretch responsive muscle) (ANKRD2), mRNA [NM_020349]	7.95	9,21	-1,26
-	chr15:091078132-091078069	11.62	12,88	-1,26
-	chr1:021531720-021531661	7.24	8,50	-1,26
AA779434	AA779434 af22b10.s1 Soares_total_fetus_Nb2HF8_9w Homo sapiens cDNA clone IMAGE:1032379 3', mRNA sequence [AA779434]	7.81	9,07	-1,26
N75427	za82f07.s1 Soares_fetal_lung_NbHL19W Homo sapiens cDNA clone IMAGE:299077 3' similar to gb:X69654 40S RIBOSOMAL PROTEIN S26 (HUMAN);, mRNA sequence [N75427]	9.36	10,64	-1,27
POLE3	Homo sapiens polymerase (DNA directed), epsilon 3 (p17 subunit) (POLE3), mRNA [NM_017443]	10.65	11,92	-1,27
MARK2	Homo sapiens MAP/microtubule affinity-regulating kinase 2 (MARK2), transcript variant 2, mRNA [NM_004954]	5.83	7,10	-1,27
LIN7C	Homo sapiens lin-7 homolog C (C. elegans) (LIN7C), mRNA [NM_018362]	7.84	9,11	-1,27

Supplemental Table 1
Morandi et al

FAM96A	Homo sapiens family with sequence similarity 96, member A (FAM96A), transcript variant 1, mRNA [NM_032231]	10.94	12,21	-1,27
CCNE1	Homo sapiens cyclin E1 (CCNE1), transcript variant 1, mRNA [NM_001238]	10.25	11,53	-1,27
COQ2	Homo sapiens coenzyme Q2 homolog, prenyltransferase (yeast) (COQ2), mRNA [NM_015697]	6.89	8,16	-1,27
C2orf25	Homo sapiens chromosome 2 open reading frame 25 (C2orf25), mRNA [NM_015702]	11.04	12,31	-1,27
CHAF1B	Homo sapiens chromatin assembly factor 1, subunit B (p60) (CHAF1B), mRNA [NM_005441]	8.88	10,16	-1,27
BCAS2	Homo sapiens breast carcinoma amplified sequence 2 (BCAS2), mRNA [NM_005872]	10.27	11,55	-1,27
-	chrX:076695738-076695797	5.86	7,13	-1,27
ENST00000335078	chr16:25373196-25373161	7.07	8,34	-1,27
-	chr10:093417022-093416962	10.9	12,17	-1,27
CA337741	CA337741 NISC_lw05f11.y1 COGENE 4PA1 Homo sapiens cDNA clone IMAGE:5609612 5', mRNA sequence [CA337741]	5.57	6,84	-1,27
THC2339084	Q93HV7 (Q93HV7) Probable dihydrolipoamide acyltransferase (Fragment), partial (5%) [THC2339084]	8.42	9,70	-1,28
YWHAE	Homo sapiens tyrosine 3-monooxygenase/tryptophan 5-monooxygenase activation protein, epsilon polypeptide (YWHAE), mRNA [NM_006761]	8.23	9,51	-1,28
POLR2J	Homo sapiens polymerase (RNA) II (DNA directed) polypeptide J, 13.3kDa (POLR2J), mRNA [NM_006234]	11.55	12,83	-1,28
NUDT21	Homo sapiens nudix (nucleoside diphosphate linked moiety X)-type motif 21 (NUDT21), mRNA [NM_007006]	9.38	10,66	-1,28
NUDCD2	Homo sapiens NudC domain containing 2 (NUDCD2), mRNA [NM_145266]	10.69	11,97	-1,28
MT	Homo sapiens malonyl-CoA:acyl carrier protein transacylase, mitochondrial (MT), nuclear gene encoding mitochondrial protein, transcript variant 2, mRNA [NM_014507]	8.98	10,26	-1,28
MGC5370	Homo sapiens hypothetical protein MGC5370, mRNA (cDNA clone IMAGE:3049213), partial cds. [BC006795]	7.66	8,93	-1,28
DR1	Homo sapiens down-regulator of transcription 1, TBP-binding (negative cofactor 2) (DR1), mRNA [NM_001938]	8.14	9,43	-1,28
C14orf130	Homo sapiens chromosome 14 open reading frame 130 (C14orf130), transcript variant 1, mRNA [NM_018108]	9.96	11,24	-1,28
CDCA3	Homo sapiens cell division cycle associated 3 (CDCA3), mRNA [NM_031299]	6.92	8,20	-1,28
-	chr5:065320262-065320218	5.46	6,74	-1,28
LOC644422	PREDICTED: Homo sapiens similar to arginine [XM_930254]	7.63	8,92	-1,29
UQCRFS1	Homo sapiens ubiquinol-cytochrome c reductase, Rieske iron-sulfur polypeptide 1 (UQCRFS1), mRNA [NM_006003]	12.5	13,79	-1,29
FLJ20516	Homo sapiens timeless-interacting protein (FLJ20516), mRNA [NM_017858]	9.07	10,36	-1,29
GMPPB	Homo sapiens mRNA for KIAA1851 protein, partial cds. [AB058754]	7.4	8,69	-1,29
INCENP	Homo sapiens inner centromere protein antigens 135/155kDa (INCENP), transcript variant 2, mRNA [NM_020238]	8.14	9,44	-1,29
ELOVL5	Homo sapiens ELOVL family member 5, elongation of long chain fatty acids (FEN1/Elo2, SUR4/Elo3-like, yeast) (ELOVL5), mRNA [NM_021814]	5.26	6,55	-1,29
C20orf24	Homo sapiens chromosome 20 open reading frame 24 (C20orf24), transcript variant 1, mRNA [NM_018840]	12.05	13,34	-1,29
C1orf51	Homo sapiens chromosome 1 open reading frame 51 (C1orf51), mRNA [NM_144697]	6.47	7,75	-1,29
C1orf144	Homo sapiens chromosome 1 open reading frame 144 (C1orf144), mRNA [NM_015609]	10.22	11,51	-1,29
UBE2D3	Homo sapiens ubiquitin-conjugating enzyme E2D 3 (UBC4/5 homolog, yeast) (UBE2D3), transcript variant 2, mRNA [NM_181886]	9.83	11,13	-1,30
UBA52	Homo sapiens ubiquitin A-52 residue ribosomal protein fusion product 1 (UBA52), transcript variant 1, mRNA [NM_001033930]	10.54	11,83	-1,30
SLC16A1	Homo sapiens solute carrier family 16 (monocarboxylic acid transporters), member 1 (SLC16A1), mRNA [NM_003051]	8.17	9,47	-1,30

Supplemental Table 1
Morandi et al

SFXN1	Homo sapiens sideroflexin 1 (SFXN1), mRNA [NM_022754]	8.24	9,54	-1,30
RNF12	Homo sapiens ring finger protein 12 (RNF12), transcript variant 1, mRNA [NM_016120]	7.36	8,66	-1,30
AF118084	Homo sapiens PRO1914 mRNA, complete cds. [AF118084]	4.91	6,20	-1,30
PAPD4	Homo sapiens PAP associated domain containing 4 (PAPD4), mRNA [NM_173797]	6.7	7,99	-1,30
ORMDL1	Homo sapiens ORM1-like 1 (S. cerevisiae) (ORMDL1), mRNA [NM_016467]	11.59	12,89	-1,30
DUSP12	Homo sapiens dual specificity phosphatase 12 (DUSP12), mRNA [NM_007240]	11.51	12,81	-1,30
C16orf61	Homo sapiens chromosome 16 open reading frame 61 (C16orf61), mRNA [NM_020188]	11.28	12,58	-1,30
CEP76	Homo sapiens centrosomal protein 76kDa (CEP76), mRNA [NM_024899]	6.71	8,01	-1,30
ATPAF2	Homo sapiens ATP synthase mitochondrial F1 complex assembly factor 2 (ATPAF2), nuclear gene encoding mitochondrial protein, mRNA [NM_145691]	8.72	10,02	-1,30
-	chr6:135982506-135982447	7.93	9,22	-1,30
THC2347074	chr3:31547505-31547446	5.74	7,04	-1,30
THC2267012	chr14:076422777-076422719	12.03	13,34	-1,30
AA725860	AA725860 ai23f08.s1 Soares_testis_NHT Homo sapiens cDNA clone 1343655 3', mRNA sequence [AA725860]	5,00	6,30	-1,30
ENST00000306716	PREDICTED: Homo sapiens similar to peptidylprolyl isomerase A isoform 1 (LOC643997), mRNA [XM_292963]	10.72	12,03	-1,31
LOC401876	PREDICTED: Homo sapiens similar to 40S ribosomal protein S16 (LOC401876), mRNA [XM_497534]	13.02	14,34	-1,31
VPS25	Homo sapiens vacuolar protein sorting 25 homolog (S. cerevisiae) (VPS25), mRNA [NM_032353]	10.27	11,58	-1,31
SMARCD1	Homo sapiens SWI/SNF related, matrix associated, actin dependent regulator of chromatin, subfamily d, member 1 (SMARCD1), transcript variant 2, mRNA [NM_139071]	5.24	6,55	-1,31
SPATA5L1	Homo sapiens spermatogenesis associated 5-like 1 (SPATA5L1), mRNA [NM_024063]	9.67	10,98	-1,31
MGC13017	Homo sapiens similar to RIKEN cDNA A430101B06 gene (MGC13017), mRNA [NM_080656]	10.33	11,64	-1,31
NOLA3	Homo sapiens nucleolar protein family A, member 3 (H/ACA small nucleolar RNPs) (NOLA3), mRNA [NM_018648]	12.54	13,85	-1,31
ENST00000306024	Homo sapiens MDS017 (MDS017) mRNA, complete cds. [AF182418]	10.86	12,17	-1,31
DENR	Homo sapiens density-regulated protein (DENR), mRNA [NM_003677]	8.87	10,18	-1,31
C9orf46	Homo sapiens chromosome 9 open reading frame 46 (C9orf46), mRNA [NM_018465]	9.13	10,43	-1,31
-	chr1:100764630-100764569	10.47	11,78	-1,31
AI630435	AI630435 ad10b05.y1 Hembase; Erythroid Progenitor Cells (LCB:ad library) Homo sapiens cDNA clone ad10b05 random, mRNA sequence [AI630435]	9.93	11,24	-1,31
LOC643433	PREDICTED: Homo sapiens similar to 60S ribosomal protein L29 (Cell surface heparin binding protein HIP), transcript variant 2 (LOC643433), mRNA [XM_932704]	13.04	14,35	-1,32
KIAA0241	Homo sapiens KIAA0241 (KIAA0241), mRNA [NM_015060]	5.04	6,36	-1,32
LOC203547	Homo sapiens hypothetical protein LOC203547 (LOC203547), mRNA [NM_001017980]	7.9	9,22	-1,32
GSR	Homo sapiens glutathione reductase (GSR), mRNA [NM_000637]	8.34	9,65	-1,32
CFLAR	Homo sapiens FLAME-1 mRNA, complete cds. [AF009616]	8.5	9,83	-1,32
C9orf156	Homo sapiens chromosome 9 open reading frame 156 (C9orf156), mRNA [NM_016481]	8.38	9,70	-1,32
CAPN1	Homo sapiens cDNA FLJ12257 fis, clone MAMMA1001501, highly similar to CALPAIN 1, LARGE [CATALYTIC] SUBUNIT (EC 3.4.22.17). [AK022319]	8.12	9,44	-1,32
SIVA	Homo sapiens CD27-binding (Siva) protein (SIVA), transcript variant 1, mRNA [NM_006427]	11.76	13,09	-1,32
BID	Homo sapiens BH3 interacting domain death agonist (BID), transcript variant 1, mRNA [NM_197966]	8.85	10,17	-1,32
-	chr4:039797740-039797679	6.29	7,61	-1,32

BG205572	RST25001 Athersys RAGE Library Homo sapiens cDNA, mRNA sequence [BG205572]	4.86	6,19	-1,33
LOC388272	Homo sapiens similar to RIKEN cDNA 4921524J17 (LOC388272), mRNA [NM_001001436]	9.68	11,01	-1,33
NDUFA4	Homo sapiens NADH dehydrogenase (ubiquinone) 1 alpha subcomplex, 4, 9kDa (NDUFA4), nuclear gene encoding mitochondrial protein, mRNA [NM_002489]	13.3	14,62	-1,33
EIF3S5	Homo sapiens eukaryotic translation initiation factor 3, subunit 5 epsilon, 47kDa (EIF3S5), mRNA [NM_003754]	12.42	13,74	-1,33
ATP5L	Homo sapiens ATP synthase, H+ transporting, mitochondrial F0 complex, subunit G (ATP5L), nuclear gene encoding mitochondrial protein, mRNA [NM_006476]	8.8	10,13	-1,33
-	chr10:097344598-097344659	9.72	11,05	-1,33
COL14A1	Human udulin 2 mRNA, 3' end. [M64109]	6.13	7,47	-1,34
ENST00000217537	Homo sapiens zinc finger protein 516, mRNA (cDNA clone IMAGE:3872546), partial cds. [BC108287]	7.65	9,00	-1,34
UXT	Homo sapiens ubiquitously-expressed transcript (UXT), transcript variant 2, mRNA [NM_004182]	11.65	12,99	-1,34
SDHB	Homo sapiens succinate dehydrogenase complex, subunit B, iron sulfur (Ip) (SDHB), mRNA [NM_003000]	10.7	12,04	-1,34
HUS1B	Homo sapiens HUS1 checkpoint homolog b (S. pombe) (HUS1B), mRNA [NM_148959]	5.47	6,81	-1,34
CNIH4	Homo sapiens cornichon homolog 4 (Drosophila) (CNIH4), mRNA [NM_014184]	11.16	12,50	-1,34
C18orf17	Homo sapiens chromosome 18 open reading frame 17, mRNA (cDNA clone IMAGE:5582870), partial cds. [BC032684]	8.16	9,50	-1,34
C14orf106	Homo sapiens chromosome 14 open reading frame 106 (C14orf106), mRNA [NM_018353]	7.03	8,36	-1,34
BC039021	Homo sapiens cDNA clone IMAGE:6043059, partial cds. [BC039021]	7.75	9,09	-1,34
BC019667	Homo sapiens cDNA clone IMAGE:4453251, partial cds. [BC019667]	7.66	9,00	-1,34
XRN1	Homo sapiens 5'-3' exoribonuclease 1 (XRN1), mRNA [NM_019001]	4.67	6,01	-1,34
AA176161	zp23h07.s1 Stratagene neuroepithelium (#937231) Homo sapiens cDNA clone IMAGE:610333 3', mRNA sequence [AA176161]	6.48	7,83	-1,35
THC2406099	HUMUBCP pro-ubiquitin {Homo sapiens;} , partial (39%) [THC2406099]	11.67	13,02	-1,35
TKT	Homo sapiens transketolase (Wernicke-Korsakoff syndrome) (TKT), mRNA [NM_001064]	13.37	14,72	-1,35
SFRS7	Homo sapiens splicing factor, arginine/serine-rich 7, 35kDa (SFRS7), mRNA [NM_001031684]	12.8	14,14	-1,35
SPCS1	Homo sapiens signal peptidase complex subunit 1 homolog (S. cerevisiae) (SPCS1), mRNA [NM_014041]	10.93	12,28	-1,35
SIAH1	Homo sapiens seven in absentia homolog 1 (Drosophila) (SIAH1), transcript variant 1, mRNA [NM_003031]	8.99	10,34	-1,35
RHOG	Homo sapiens ras homolog gene family, member G (rho G) (RHOG), mRNA [NM_001665]	9.48	10,83	-1,35
FAM76B	Homo sapiens family with sequence similarity 76, member B (FAM76B), mRNA [NM_144664]	7.52	8,87	-1,35
AK098185	Homo sapiens cDNA FLJ40866 fis, clone TRACH2018718. [AK098185]	5.47	6,81	-1,35
BC039097	Homo sapiens cDNA clone IMAGE:4823416. [BC039097]	7.53	8,88	-1,35
BC007306	Homo sapiens cDNA clone IMAGE:3352215, **** WARNING: chimeric clone ****. [BC007306]	5.4	6,75	-1,35
-	chr6:154990189-154990130	4.81	6,15	-1,35
-	chr5:108092085-108092144	7.52	8,87	-1,35
-	chr3:025766034-025766097	12.33	13,68	-1,35
BF366211	BF366211 IL2-NT0099-300500-096-H08 NT0099 Homo sapiens cDNA, mRNA sequence [BF366211]	6.23	7,58	-1,35
SMAP1	Homo sapiens stromal membrane-associated protein 1 (SMAP1), mRNA [NM_021940]	8.07	9,43	-1,36
SRA1	Homo sapiens steroid receptor RNA activator 1 (SRA1), mRNA [NM_001035235]	9.89	11,25	-1,36

RMND5A	Homo sapiens required for meiotic nuclear division 5 homolog A (S. cerevisiae) (RMND5A), mRNA [NM_022780]	7.48	8,84	-1,36
MPHOSPH9	Homo sapiens M-phase phosphoprotein 9 (MPHOSPH9), mRNA [NM_022782]	6.94	8,30	-1,36
MAD2L2	Homo sapiens MAD2 mitotic arrest deficient-like 2 (yeast) (MAD2L2), mRNA [NM_006341]	10.04	11,40	-1,36
KIAA1333	Homo sapiens KIAA1333 (KIAA1333), mRNA [NM_017769]	7.16	8,52	-1,36
ILF2	Homo sapiens interleukin enhancer binding factor 2, 45kDa (ILF2), mRNA [NM_004515]	13.16	14,52	-1,36
C14orf168	Homo sapiens chromosome 14 open reading frame 168 (C14orf168), mRNA [NM_031427]	6.98	8,34	-1,36
HSUP1	Homo sapiens cDNA FLJ42181 fis, clone THYMU2031368. [AK124175]	5.77	7,13	-1,36
AK092559	Homo sapiens cDNA FLJ35240 fis, clone PROST2002425, moderately similar to ZINC FINGER PROTEIN 136. [AK092559]	6.01	7,38	-1,36
CKS1B	Homo sapiens CDC28 protein kinase regulatory subunit 1B (CKS1B), mRNA [NM_001826]	11.95	13,31	-1,36
TGFB1	Homo sapiens transforming growth factor, beta 1 (Camurati-Engelmann disease) (TGFB1), mRNA [NM_000660]	7.42	8,79	-1,37
SCC-112	Homo sapiens SCC-112 protein, mRNA (cDNA clone IMAGE:5273075), complete cds. [BC041361]	7.38	8,76	-1,37
RBM18	Homo sapiens RNA binding motif protein 18 (RBM18), mRNA [NM_033117]	8.21	9,58	-1,37
PCNA	Homo sapiens proliferating cell nuclear antigen (PCNA), transcript variant 1, mRNA [NM_002592]	10.93	12,30	-1,37
HIST1H2AG	Homo sapiens histone 1, H2ag (HIST1H2AG), mRNA [NM_021064]	10.95	12,32	-1,37
DNMT2	Homo sapiens DNA (cytosine-5-)-methyltransferase 2 (DNMT2), transcript variant a, mRNA [NM_004412]	5.68	7,05	-1,37
CXXC5	Homo sapiens cDNA clone IMAGE:3528660, **** WARNING: chimeric clone ****. [BC013025]	6.66	8,03	-1,37
SLC6A6	Homo sapiens solute carrier family 6 (neurotransmitter transporter, taurine), member 6 (SLC6A6), mRNA [NM_003043]	8.79	10,17	-1,38
RFC4	Homo sapiens replication factor C (activator 1) 4, 37kDa (RFC4), transcript variant 1, mRNA [NM_002916]	10.43	11,81	-1,38
AF147723	Homo sapiens lipopolysaccharide specific response-68 protein (LSR68) mRNA, complete cds. [AF147723]	6.78	8,16	-1,38
RP11-484I6.3	Homo sapiens hypothetical protein BC015148 (LOC93081), mRNA [NM_138779]	9.11	10,48	-1,38
LOC90580	Homo sapiens hypothetical protein BC011833 (LOC90580), mRNA [NM_138358]	9.33	10,71	-1,38
DENND1A	Homo sapiens DENN/MADD domain containing 1A (DENND1A), transcript variant 2, mRNA [NM_024820]	5.8	7,18	-1,38
CA5B	Homo sapiens carbonic anhydrase VB, mitochondrial (CA5B), nuclear gene encoding mitochondrial protein, mRNA [NM_007220]	5.6	6,98	-1,38
BNIP2	Homo sapiens BCL2/adenovirus E1B 19kDa interacting protein 2 (BNIP2), mRNA [NM_004330]	8.86	10,24	-1,38
ATP5B	Homo sapiens ATP synthase, H+ transporting, mitochondrial F1 complex, beta polypeptide (ATP5B), nuclear gene encoding mitochondrial protein, mRNA [NM_001686]	12.45	13,83	-1,38
HACL1	Homo sapiens 2-hydroxyacyl-CoA lyase 1 (HACL1), mRNA [NM_012260]	9.83	11,20	-1,38
-	chr3:115305098-115305037	5.57	6,95	-1,38
ENST00000328644	chr20:60947586-60947645	7.02	8,40	-1,38
THC2388671	RL1X_HUMAN (Q02543) 60S ribosomal protein L18a, partial (98%) [THC2388671]	11.54	12,93	-1,39
YKT6	Homo sapiens YKT6 v-SNARE homolog (S. cerevisiae) (YKT6), mRNA [NM_006555]	8.06	9,46	-1,39
RY1	Homo sapiens putative nucleic acid binding protein RY-1 (RY1), mRNA [NM_006857]	10.16	11,56	-1,39
POLA2	Homo sapiens polymerase (DNA directed), alpha 2 (70kD subunit) (POLA2), mRNA [NM_002689]	7.94	9,33	-1,39
PIGA	Homo sapiens phosphatidylinositol glycan anchor biosynthesis, class A (paroxysmal nocturnal hemoglobinuria) (PIGA), transcript variant 1, mRNA [NM_002641]	8.32	9,71	-1,39

Supplemental Table 1
Morandi et al

GPSM2	Homo sapiens G-protein signalling modulator 2 (AGS3-like, C. elegans) (GPSM2), mRNA [NM_013296]	8,00	9,39	-1,39
ENST00000278949	Homo sapiens cDNA FLJ38080 fis, clone CTONG2016185. [AK095399]	6.82	8,21	-1,39
AL536879	AL536879 AL536879 Homo sapiens FETAL BRAIN Homo sapiens cDNA clone CS0DF020YM21 3-PRIME, mRNA sequence [AL536879]	6.93	8,32	-1,39
SLC25A5	Homo sapiens solute carrier family 25 (mitochondrial carrier; adenine nucleotide translocator), member 5 (SLC25A5), mRNA [NM_001152]	11.12	12,52	-1,40
PX19	Homo sapiens px19-like protein (PX19), mRNA [NM_013237]	11.18	12,58	-1,40
AF119911	Homo sapiens PRO2975 mRNA, complete cds. [AF119911]	4.35	5,75	-1,40
MAPKAPK3	Homo sapiens mitogen-activated protein kinase-activated protein kinase 3 (MAPKAPK3), mRNA [NM_004635]	8.81	10,21	-1,40
LOC285074	Homo sapiens hypothetical protein LOC285074 (LOC285074), mRNA [NM_001012626]	9.54	10,94	-1,40
H2AFZ	Homo sapiens H2A histone family, member Z (H2AFZ), mRNA [NM_002106]	12.02	13,43	-1,40
EIF5A	Homo sapiens eukaryotic translation initiation factor 5A (EIF5A), mRNA [NM_001970]	8.36	9,76	-1,40
ARMC8	Homo sapiens armadillo repeat containing 8 (ARMC8), transcript variant 3, mRNA [NM_213654]	8.13	9,53	-1,40
THC2442931	chr9:128536667-128536608	9.53	10,93	-1,40
-	chr6:036677849-036677790	5.13	6,52	-1,40
-	chr3:139845444-139845388	10.9	12,30	-1,40
USP3	Homo sapiens ubiquitin specific peptidase 3 (USP3), mRNA [NM_006537]	4.55	5,97	-1,41
SFRS10	Homo sapiens splicing factor, arginine/serine-rich 10 (transformer 2 homolog, Drosophila) (SFRS10), mRNA [NM_004593]	11.96	13,38	-1,41
SAAL1	Homo sapiens serum amyloid A-like 1 (SAAL1), mRNA [NM_138421]	8.81	10,22	-1,41
NAT13	Homo sapiens N-acetyltransferase 13 (NAT13), mRNA [NM_025146]	9.18	10,58	-1,41
GPX7	Homo sapiens glutathione peroxidase 7 (GPX7), mRNA [NM_015696]	7.58	8,99	-1,41
EID3	Homo sapiens E1A-like inhibitor of differentiation 3 (EID3), mRNA [NM_001008394]	8.06	9,48	-1,41
COPS3	Homo sapiens COP9 constitutive photomorphogenic homolog subunit 3 (Arabidopsis) (COPS3), mRNA [NM_003653]	11.88	13,30	-1,41
USF2	Homo sapiens clone TCCCIA00046 mRNA sequence. [AY007087]	4.42	5,84	-1,41
ASB1	Homo sapiens ankyrin repeat and SOCS box-containing 1 (ASB1), mRNA [NM_001040445]	7.87	9,28	-1,41
BQ233242	BQ233242 AGENCOURT_7283259 NIH_MGC_70 Homo sapiens cDNA clone IMAGE:6017276 5', mRNA sequence [BQ233242]	8.56	9,97	-1,41
THC2310680	AF262221 SNARE Vti1a protein {Rattus norvegicus;} , complete [THC2310680]	7.24	8,66	-1,41
SNRPA1	Homo sapiens small nuclear ribonucleoprotein polypeptide A' (SNRPA1), mRNA [NM_003090]	9.78	11,20	-1,42
SCYL2	Homo sapiens SCY1-like 2 (S. cerevisiae) (SCYL2), mRNA [NM_017988]	10.01	11,43	-1,42
C1orf112	Homo sapiens chromosome 1 open reading frame 112 (C1orf112), mRNA [NM_018186]	7.98	9,40	-1,42
NT5DC2	Homo sapiens 5'-nucleotidase domain containing 2 (NT5DC2), mRNA [NM_022908]	10.58	12,00	-1,42
W05707	W05707 za87h03.r1 Soares_fetal_lung_NbHL19W Homo sapiens cDNA clone IMAGE:299573 5' similar to SW:TCPD_MOUSE P80315 T-COMPLEX PROTEIN 1, DELTA SUBUNIT ;, mRNA sequence [W05707]	4.61	6,04	-1,43
AI698357	tx64a04.x1 NCI_CGAP_Ut1 Homo sapiens cDNA clone IMAGE:2274318 3', mRNA sequence [AI698357]	7.49	8,92	-1,43
X05126	Human fibroblast mRNA fragment with Alu sequence (pRHF11). [X05126]	6.33	7,76	-1,43
AJ227863	Homo sapiens partial mRNA; ID YG39-2B. [AJ227863]	9.48	10,91	-1,43

Supplemental Table 1
Morandi et al

NFYA	Homo sapiens nuclear transcription factor Y, alpha (NFYA), transcript variant 1, mRNA [NM_002505]	5.53	6,96	-1,43
MIER1	Homo sapiens mesoderm induction early response 1 homolog (Xenopus laevis) (MIER1), mRNA [NM_020948]	8.76	10,19	-1,43
LSM3	Homo sapiens LSM3 homolog, U6 small nuclear RNA associated (S. cerevisiae) (LSM3), mRNA [NM_014463]	10.29	11,72	-1,43
LOC440983	Homo sapiens hypothetical gene supported by BC066916, mRNA (cDNA clone IMAGE:4838452). [BC066916]	10.67	12,09	-1,43
AK124072	Homo sapiens cDNA FLJ42078 fis, clone SYNOV2020085. [AK124072]	7.44	8,87	-1,43
ACN9	Homo sapiens ACN9 homolog (S. cerevisiae) (ACN9), mRNA [NM_020186]	9.19	10,62	-1,43
AA126789	AA126789 zn87e10.s1 Stratagene lung carcinoma 937218 Homo sapiens cDNA clone IMAGE:565194 3', mRNA sequence [AA126789]	6.17	7,60	-1,43
XRCC4	Homo sapiens X-ray repair complementing defective repair in Chinese hamster cells 4 (XRCC4), transcript variant 3, mRNA [NM_022550]	7.73	9,17	-1,44
RNMT	Homo sapiens RNA (guanine-7-) methyltransferase (RNMT), mRNA [NM_003799]	7,00	8,44	-1,44
ARHGDIA	Homo sapiens Rho GDP dissociation inhibitor (GDI) alpha (ARHGDIA), mRNA [NM_004309]	7.25	8,69	-1,44
PHC3	Homo sapiens polyhomeotic like 3 (Drosophila) (PHC3), mRNA [NM_024947]	6.59	8,03	-1,44
MNS1	Homo sapiens meiosis-specific nuclear structural 1 (MNS1), mRNA [NM_018365]	6.99	8,43	-1,44
AK097724	Homo sapiens cDNA FLJ40405 fis, clone TESTI2037382. [AK097724]	6.08	7,52	-1,44
CR603215	full-length cDNA clone CS0DI023YC17 of Placenta Cot 25-normalized of Homo sapiens (human). [CR603215]	10.01	11,45	-1,44
BG952851	BG952851 PM4-CT0545-200101-001-e11 CT0545 Homo sapiens cDNA, mRNA sequence [BG952851]	6.69	8,14	-1,44
CCDC99	Homo sapiens coiled-coil domain containing 99 (CCDC99), mRNA [NM_017785]	7.41	8,86	-1,45
C16orf33	Homo sapiens chromosome 16 open reading frame 33 (C16orf33), mRNA [NM_024571]	10.25	11,70	-1,45
CHML	Homo sapiens choroideremia-like (Rab escort protein 2) (CHML), mRNA [NM_001821]	6.23	7,68	-1,45
CANT1	Homo sapiens calcium activated nucleotidase 1 (CANT1), mRNA [NM_138793]	5.7	7,15	-1,45
ANKRD44	Homo sapiens ankyrin repeat domain 44 (ANKRD44), mRNA [NM_153697]	6.19	7,64	-1,45
-	chr8:038360092-038360151	6.26	7,70	-1,45
THC2336812	BF930236 IL5-NT0227-111200-319-g04 NT0227 Homo sapiens cDNA, mRNA sequence [BF930236]	4.53	5,98	-1,45
LOC646626	PREDICTED: Homo sapiens hypothetical protein LOC647393 (LOC647393), mRNA [XM_942822]	7.39	8,86	-1,46
RPL34	Homo sapiens ribosomal protein L34 (RPL34), transcript variant 2, mRNA [NM_033625]	11.9	13,35	-1,46
BX641027	Homo sapiens mRNA; cDNA DKFZp686O10247 (from clone DKFZp686O10247). [BX641027]	8.59	10,06	-1,46
RP4-747L4.3	Homo sapiens hypothetical protein MGC12538, mRNA (cDNA clone MGC:12538 IMAGE:3839075), complete cds. [BC007072]	7.08	8,54	-1,46
APOM	Homo sapiens apolipoprotein M (APOM), mRNA [NM_019101]	7.94	9,40	-1,46
-	chr20:061297510-061297569	7.33	8,79	-1,46
THC2436745	RSF1_HUMAN (Q9NS23) Ras association domain family 1 (Ras association, RalGDS/AF-6, domain family 1), partial (11%) [THC2436745]	6.44	7,91	-1,47
STT3B	Homo sapiens STT3, subunit of the oligosaccharyltransferase complex, homolog B (S. cerevisiae) (STT3B), mRNA [NM_178862]	6.82	8,29	-1,47
SP1	Homo sapiens Sp1 transcription factor (SP1), mRNA [NM_138473]	5.17	6,64	-1,47
RPL35	Homo sapiens ribosomal protein L35 (RPL35), mRNA [NM_007209]	12.72	14,19	-1,47

RHOA	Homo sapiens ras homolog gene family, member A (RHOA), mRNA [NM_001664]	12.09	13,56	-1,47
NIN	Homo sapiens ninein (GSK3B interacting protein) (NIN), transcript variant 4, mRNA [NM_016350]	7.82	9,29	-1,47
IFRD1	Homo sapiens interferon-related developmental regulator 1 (IFRD1), transcript variant 2, mRNA [NM_001007245]	10.68	12,15	-1,47
NP107055	GB|AC004079.1|AAB97952.1 40% similar to yeast high mobility group-like nuclear protein, P32495 (PID:g417360) [NP107055]	5.44	6,90	-1,47
THC2371907	CCHU cytochrome c [validated] - human {Homo sapiens;} , partial (85%) [THC2371907]	11,00	12,47	-1,47
AW079854	AW079854 xe54e05.x1 NCI_CGAP_Ut3 Homo sapiens cDNA clone IMAGE:2611712 3', mRNA sequence [AW079854]	8.26	9,74	-1,47
U63542	Human putative FAP protein mRNA, partial cds. [U63542]	4.61	6,09	-1,48
VRK1	Homo sapiens vaccinia related kinase 1 (VRK1), mRNA [NM_003384]	8.86	10,35	-1,48
UFM1	Homo sapiens ubiquitin-fold modifier 1 (UFM1), mRNA [NM_016617]	7.65	9,12	-1,48
RSHL2	Homo sapiens radial spokehead-like 2 (RSHL2), mRNA [NM_031924]	7.77	9,25	-1,48
MASTL	Homo sapiens microtubule associated serine/threonine kinase-like (MASTL), mRNA [NM_032844]	6.24	7,72	-1,48
CAMTA1	Homo sapiens hypothetical protein SB141 mRNA, complete cds. [AY037153]	11.54	13,02	-1,48
DLEU1	Homo sapiens deleted in lymphocytic leukemia, 1 (DLEU1) on chromosome 13 [NR_002605]	6.85	8,33	-1,48
COPE	Homo sapiens coatomer protein complex, subunit epsilon (COPE), transcript variant 1, mRNA [NM_007263]	10.56	12,04	-1,48
C4orf15	Homo sapiens chromosome 4 open reading frame 15 (C4orf15), mRNA [NM_024511]	7.3	8,78	-1,48
C15orf29	Homo sapiens chromosome 15 open reading frame 29 (C15orf29), mRNA [NM_024713]	8.74	10,22	-1,48
-	chr18:003968255-003968196	11.25	12,73	-1,48
-	chr12:038147358-038147297	5.57	7,05	-1,48
BX360933	BX360933 BX360933 Homo sapiens PLACENTA COT 25-NORMALIZED Homo sapiens cDNA clone CS0DI077YB17 3-PRIME, mRNA sequence [BX360933]	6.08	7,56	-1,48
TBRG1	Homo sapiens transforming growth factor beta regulator 1, mRNA (cDNA clone IMAGE:5212572), complete cds. [BC032312]	8.82	10,31	-1,49
SEPT11	Homo sapiens septin 11 (SEPT11), mRNA [NM_018243]	8.54	10,03	-1,49
CCDC59	Homo sapiens coiled-coil domain containing 59 (CCDC59), mRNA [NM_014167]	10.75	12,24	-1,49
CNAP1	Homo sapiens chromosome condensation-related SMC-associated protein 1 (CNAP1), mRNA [NM_014865]	9.83	11,32	-1,49
CENPO	Homo sapiens centromere protein O (CENPO), mRNA [NM_024322]	6.44	7,93	-1,49
-	chr18:046238833-046238893	11.61	13,09	-1,49
TMEM33	Homo sapiens transmembrane protein 33 (TMEM33), mRNA [NM_018126]	8.38	9,88	-1,50
RHOF	Homo sapiens ras homolog gene family, member F (in filopodia) (RHOF), mRNA [NM_019034]	5,00	6,50	-1,50
PDK1	Homo sapiens pyruvate dehydrogenase kinase, isozyme 1 (PDK1), nuclear gene encoding mitochondrial protein, mRNA [NM_002610]	5.03	6,53	-1,50
IER3IP1	Homo sapiens PRO2309 mRNA, complete cds. [AF119875]	6.97	8,48	-1,50
PPIH	Homo sapiens peptidylprolyl isomerase H (cyclophilin H) (PPIH), mRNA [NM_006347]	6.53	8,04	-1,50
HIST1H2AM	Homo sapiens histone 1, H2am (HIST1H2AM), mRNA [NM_003514]	10.81	12,31	-1,50
FAM101B	Homo sapiens family with sequence similarity 101, member B (FAM101B), mRNA [NM_182705]	7.82	9,32	-1,50
DTYMK	Homo sapiens deoxythymidylate kinase (thymidylate kinase) (DTYMK), mRNA [NM_012145]	9.55	11,06	-1,50
CTPS	Homo sapiens CTP synthase (CTPS), mRNA [NM_001905]	10.61	12,11	-1,50

Supplemental Table 1
Morandi et al

CHAF1A	Homo sapiens chromatin assembly factor 1, subunit A (p150) (CHAF1A), mRNA [NM_005483]	9.91	11,41	-1,50
CBLL1	Homo sapiens Cas-Br-M (murine) ecotropic retroviral transforming sequence-like 1 (CBLL1), mRNA [NM_024814]	6.51	8,01	-1,50
ENST00000356170	chr20:1304142-1300833	8.21	9,71	-1,50
-	chr14:066922345-066922286	7.03	8,54	-1,50
ZNF313	Homo sapiens zinc finger protein 313 (ZNF313), mRNA [NM_018683]	9.63	11,15	-1,51
RNF170	Homo sapiens ring finger protein 170, mRNA (cDNA clone MGC:40251 IMAGE:5200847), complete cds. [BC032393]	4.87	6,38	-1,51
RABGEF1	Homo sapiens RAB guanine nucleotide exchange factor (GEF) 1 (RABGEF1), mRNA [NM_014504]	7.94	9,46	-1,51
PANK3	Homo sapiens pantothenate kinase 3 (PANK3), mRNA [NM_024594]	7.35	8,86	-1,51
HSPC159	Homo sapiens HSPC159 protein (HSPC159), mRNA [NM_014181]	7.59	9,10	-1,51
DERL2	Homo sapiens Der1-like domain family, member 2 (DERL2), mRNA [NM_016041]	9.86	11,38	-1,51
CYB5R4	Homo sapiens cytochrome b5 reductase 4 (CYB5R4), mRNA [NM_016230]	8.38	9,89	-1,51
AK023559	Homo sapiens cDNA FLJ13497 fis, clone PLACE1004518. [AK023559]	6.17	7,68	-1,51
CR612178	full-length cDNA clone CS0DI015YM13 of Placenta Cot 25-normalized of Homo sapiens (human). [CR612178]	12.57	14,08	-1,51
-	chr15:042434782-042434841	9.78	11,29	-1,51
BX457728	BX457728 BX457728 Homo sapiens NEUROBLASTOMA Homo sapiens cDNA clone CS0DA003YD09 3-PRIME, mRNA sequence [BX457728]	6.8	8,31	-1,51
RPS2	Homo sapiens OK/KNS-cl.7 mRNA for ribosomal protein S2, complete cds. [AB065089]	8.92	10,44	-1,52
NAPG	Homo sapiens N-ethylmaleimide-sensitive factor attachment protein, gamma (NAPG), mRNA [NM_003826]	7.22	8,74	-1,52
KNTC1	Homo sapiens kinetochore associated 1 (KNTC1), mRNA [NM_014708]	8.12	9,65	-1,52
MGC24039	Homo sapiens hypothetical protein MGC24039, mRNA (cDNA clone IMAGE:4286826), complete cds. [BC020855]	6.28	7,80	-1,52
FLJ22624	Homo sapiens FLJ22624 protein (FLJ22624), mRNA [NM_024808]	6.41	7,93	-1,52
ALS2CR4	Homo sapiens amyotrophic lateral sclerosis 2 (juvenile) chromosome region, candidate 4 (ALS2CR4), mRNA [NM_152388]	9.83	11,35	-1,52
-	chr13:031998075-031998134	6.61	8,12	-1,52
AV737563	AV737563 CB Homo sapiens cDNA clone CBCCNA11 5', mRNA sequence [AV737563]	9.4	10,92	-1,52
UBE2S	Homo sapiens ubiquitin-conjugating enzyme E2S (UBE2S), mRNA [NM_014501]	11.88	13,41	-1,53
TMEM70	Homo sapiens transmembrane protein 70 (TMEM70), transcript variant 1, mRNA [NM_017866]	9.66	11,18	-1,53
RPA3	Homo sapiens replication protein A3, 14kDa (RPA3), mRNA [NM_002947]	10.87	12,39	-1,53
PTPRJ	Homo sapiens protein tyrosine phosphatase, receptor type, J (PTPRJ), mRNA [NM_002843]	6.06	7,59	-1,53
MALT1	Homo sapiens mucosa associated lymphoid tissue lymphoma translocation gene 1 (MALT1), transcript variant 1, mRNA [NM_006785]	7.11	8,64	-1,53
MCFP	Homo sapiens mitochondrial carrier family protein (MCFP), mRNA [NM_018843]	6.23	7,75	-1,53
ENAH	Homo sapiens enabled homolog (Drosophila) (ENAH), transcript variant 2, mRNA [NM_018212]	6.54	8,08	-1,53
CV339166	CV339166 MR0-BT2503-050601-101-a04 BT2503 Homo sapiens cDNA, mRNA sequence [CV339166]	4.96	6,49	-1,53
SERPINB8	Homo sapiens serpin peptidase inhibitor, clade B (ovalbumin), member 8 (SERPINB8), transcript variant 2, mRNA [NM_198833]	5.56	7,10	-1,54
SAR1A	Homo sapiens SAR1 gene homolog A (S. cerevisiae) (SAR1A), mRNA [NM_020150]	11.09	12,64	-1,54

Supplemental Table 1
Morandi et al

RPL22L1	Homo sapiens ribosomal protein L22-like 1, mRNA (cDNA clone IMAGE:4865966). [BC049823]	12.2	13,74	-1,54
C1orf96	Homo sapiens chromosome 1 open reading frame 96, mRNA (cDNA clone MGC:32857 IMAGE:4731004), complete cds. [BC039241]	5.06	6,61	-1,54
ADAM9	Homo sapiens ADAM metallopeptidase domain 9 (meltrin gamma) (ADAM9), transcript variant 2, mRNA [NM_001005845]	6.82	8,36	-1,54
AV738929	AV738929 AV738929 CB Homo sapiens cDNA clone CBCCMG04 5', mRNA sequence [AV738929]	6.69	8,23	-1,54
THC2340907	ALU2_HUMAN (P39189) Alu subfamily SB sequence contamination warning entry, partial (7%) [THC2340907]	5.45	6,99	-1,54
UBE2D2	Homo sapiens ubiquitin-conjugating enzyme E2D 2 (UBC4/5 homolog, yeast) (UBE2D2), transcript variant 2, mRNA [NM_181838]	9.54	11,08	-1,55
SUV39H2	Homo sapiens suppressor of variegation 3-9 homolog 2 (Drosophila) (SUV39H2), mRNA [NM_024670]	8.29	9,85	-1,55
H2AFX	Homo sapiens H2A histone family, member X (H2AFX), mRNA [NM_002105]	11.73	13,29	-1,55
BC002350	Homo sapiens cDNA clone IMAGE:2820510. [BC002350]	5.34	6,89	-1,55
THC2307535	GDIR_HUMAN (P52565) Rho GDP-dissociation inhibitor 1 (Rho GDI 1) (Rho-GDI alpha), partial (67%) [THC2307535]	7.48	9,04	-1,55
ENST00000331979	chr5:78842034-78842093	10.86	12,41	-1,55
BE739632	BE739632 601556329T1 NIH_MGC_58 Homo sapiens cDNA clone IMAGE:3826255 3', mRNA sequence [BE739632]	6.76	8,32	-1,55
UBE2D1	Homo sapiens ubiquitin-conjugating enzyme E2D 1 (UBC4/5 homolog, yeast) (UBE2D1), mRNA [NM_003338]	7.47	9,03	-1,56
SEPX1	Homo sapiens selenoprotein X, 1 (SEPX1), mRNA [NM_016332]	10.09	11,65	-1,56
HELLS	Homo sapiens helicase, lymphoid-specific (HELLS), mRNA [NM_018063]	6.36	7,92	-1,56
GLRX	Homo sapiens glutaredoxin (thioltransferase) (GLRX), mRNA [NM_002064]	10.87	12,43	-1,56
FAM119A	Homo sapiens family with sequence similarity 119, member A (FAM119A), mRNA [NM_145280]	7.82	9,38	-1,56
ERGIC1	Homo sapiens endoplasmic reticulum-golgi intermediate compartment (ERGIC) 1 (ERGIC1), transcript variant 2, mRNA [NM_020462]	5.49	7,06	-1,56
DDX3X	Homo sapiens DEAD (Asp-Glu-Ala-Asp) box polypeptide 3, X-linked (DDX3X), mRNA [NM_001356]	6.66	8,22	-1,56
C16orf55	Homo sapiens chromosome 16 open reading frame 55 (C16orf55), mRNA [NM_153025]	7.48	9,04	-1,56
AK054718	Homo sapiens cDNA FLJ30156 fis, clone BRACE2000487. [AK054718]	6.66	8,22	-1,56
-	chr5:052745584-052745525	6.91	8,47	-1,56
BM983822	BM983822 UI-CF-DU1-aay-e-18-0-UI.s1 UI-CF-DU1 Homo sapiens cDNA clone UI-CF-DU1-aay-e-18-0-UI 3', mRNA sequence [BM983822]	8.95	10,51	-1,56
AI143116	qb76c03.x1 Soares_fetal_heart_NbHH19W Homo sapiens cDNA clone IMAGE:1706020 3', mRNA sequence [AI143116]	9.99	11,56	-1,57
ALDOAP2	Human aldolase pseudogene mRNA, complete cds. [M21191]	9.42	10,99	-1,57
WDR51A	Homo sapiens WD repeat domain 51A (WDR51A), mRNA [NM_015426]	8.31	9,88	-1,57
LOC51136	Homo sapiens PTD016 protein (LOC51136), mRNA [NM_016125]	6.52	8,09	-1,57
PAFAH1B1	Homo sapiens platelet-activating factor acetylhydrolase, isoform Ib, alpha subunit 45kDa (PAFAH1B1), mRNA [NM_000430]	9.98	11,55	-1,57
MRPS30	Homo sapiens mitochondrial ribosomal protein S30 (MRPS30), nuclear gene encoding mitochondrial protein, mRNA [NM_016640]	7.4	8,97	-1,57
JMY	Homo sapiens junction-mediating and regulatory protein (JMY), mRNA [NM_152405]	6.69	8,26	-1,57
DOT1L	Homo sapiens DOT1-like, histone H3 methyltransferase (S. cerevisiae) (DOT1L), mRNA [NM_032482]	7.08	8,65	-1,57
-	chr2:020277298-020277237	10.31	11,88	-1,57

Supplemental Table 1
Morandi et al

-	chr17:037939683-037939742	11.37	12,94	-1,57
ENST00000332534	chr1:94479773-94479832	7.57	9,14	-1,57
BE168511	BE168511 QV3-HT0514-220300-127-d06 HT0514 Homo sapiens cDNA, mRNA sequence [BE168511]	5.17	6,74	-1,57
BE165955	BE165955 MR3-HT0487-140200-112-g02 HT0487 Homo sapiens cDNA, mRNA sequence [BE165955]	5.94	7,51	-1,57
ZBTB25	Homo sapiens zinc finger and BTB domain containing 25 (ZBTB25), mRNA [NM_006977]	5.43	7,00	-1,58
UBE2W	Homo sapiens ubiquitin-conjugating enzyme E2W (putative) (UBE2W), transcript variant 1, mRNA [NM_001001481]	6.32	7,90	-1,58
PX19	Homo sapiens px19-like protein (PX19), mRNA [NM_013237]	11.44	13,02	-1,58
PURB	Homo sapiens purine-rich element binding protein B (PURB), mRNA [NM_033224]	8.14	9,71	-1,58
LOC124491	Homo sapiens LOC124491 (LOC124491), mRNA [NM_145254]	8.98	10,56	-1,58
IFP38	Homo sapiens IFP38 (IFP38), mRNA [NM_031943]	6.68	8,26	-1,58
AY129015	Homo sapiens clone FP17889 unknown mRNA. [AY129015]	5.21	6,79	-1,58
ATAD2	Homo sapiens ATPase family, AAA domain containing 2 (ATAD2), mRNA [NM_014109]	6.83	8,41	-1,58
-	chr7:087839895-087839836	7.43	9,01	-1,58
THC2415133	chr1:207819859-207819800	5.35	6,93	-1,58
BG943724	BG943724 ax41c02.x1 Hembase; Erythroid Progenitor Cells (LCB:ax library) Homo sapiens cDNA clone ax41c02 random, mRNA sequence [BG943724]	5.99	7,57	-1,58
BF513730	BF513730 UI-H-BW1-amy-e-05-0-UI.s1 NCI_CGAP_Sub7 Homo sapiens cDNA clone IMAGE:3071696 3', mRNA sequence [BF513730]	5.8	7,37	-1,58
AL522622	AL522622 AL522622 Homo sapiens NEUROBLASTOMA COT 10-NORMALIZED Homo sapiens cDNA clone CS0DB009YE01 3-PRIME, mRNA sequence [AL522622]	6.96	8,54	-1,58
ENST00000294383	Homo sapiens ubiquitin specific peptidase 24, mRNA (cDNA clone IMAGE:4995223), complete cds. [BC029660]	8.24	9,83	-1,59
BQ337821	BQ337821 PM3-NN1082-230400-001-d08 NN1082 Homo sapiens cDNA, mRNA sequence [BQ337821]	6.2	7,79	-1,59
AI090167	AI090167 qb33g12.x1 Soares_pregnant_uterus_NbHPU Homo sapiens cDNA clone IMAGE:1698118 3', mRNA sequence [AI090167]	6.18	7,77	-1,59
THC2270231	PBEF_HUMAN (P43490) Pre-B cell enhancing factor precursor, partial (20%) [THC2270231]	9.47	11,07	-1,60
LIN7A	Homo sapiens lin-7 homolog A (C. elegans) (LIN7A), mRNA [NM_004664]	6.04	7,65	-1,60
GNA13	Homo sapiens guanine nucleotide binding protein (G protein), alpha 13 (GNA13), mRNA [NM_006572]	6.84	8,44	-1,60
SLC35F5	Homo sapiens cDNA: FLJ22004 fis, clone HEP06871. [AK025657]	5.25	6,86	-1,60
-	chr9:092076255-092076196	5.5	7,09	-1,60
-	chr12:093417590-093417649	5.07	6,67	-1,60
BX415272	BX415272 Homo sapiens THYMUS Homo sapiens cDNA clone CS0CAP005YK02 3-PRIME, mRNA sequence [BX415272]	4.85	6,45	-1,60
BF085047	BF085047 PM0-GN0018-130900-003-c12 GN0018 Homo sapiens cDNA, mRNA sequence [BF085047]	5.14	6,74	-1,60
AA743218	AA743218 ny21c02.s1 NCI_CGAP_GCB1 Homo sapiens cDNA clone IMAGE:1272386 3' similar to gb:S87759 PROTEIN PHOSPHATASE 2C ALPHA ISOFORM (HUMAN);, mRNA sequence [AA743218]	6.89	8,49	-1,60
tcag7.441	PREDICTED: Homo sapiens similar to Ubiquinol-cytochrome c reductase iron-sulfur subunit, mitochondrial precursor (Rieske iron-sulfur protein) (RISP) (Liver regeneration-related protein LRRGT00195) (LOC647123), mRNA [XM_930141]	7.8	9,41	-1,61
ZFAND5	Homo sapiens zinc finger, AN1-type domain 5 (ZFAND5), mRNA [NM_006007]	8.24	9,85	-1,61
WIBG	Homo sapiens within bgcn homolog (Drosophila), mRNA (cDNA clone IMAGE:3897762), partial cds. [BC009627]	7.1	8,71	-1,61

ENST00000331406	Homo sapiens v-myb myeloblastosis viral oncogene homolog (avian)-like 1, transcript variant 6, mRNA (cDNA clone MGC:120061 IMAGE:40018969), complete cds. [BC101188]	4.67	6,28	-1,61
UBE2J1	Homo sapiens ubiquitin-conjugating enzyme E2, J1 (UBC6 homolog, yeast) (UBE2J1), mRNA [NM_016021]	7.52	9,13	-1,61
RPL23A	Homo sapiens ribosomal protein L23a (RPL23A), mRNA [NM_000984]	10.22	11,83	-1,61
AJ009817	Homo sapiens mRNA for AMMECR1 protein, alternative exon 2. [AJ009817]	4.69	6,29	-1,61
AF131777	Homo sapiens clone 25061 mRNA sequence. [AF131777]	5.32	6,94	-1,61
THC2375545	chr5:158622080-158622021	5.83	7,44	-1,61
THC2370450	AA805504 oc12e06.s1 NCI_CGAP_GCB1 Homo sapiens cDNA clone IMAGE:1340674 3' similar to contains Alu repetitive element;, mRNA sequence [AA805504]	4.9	6,51	-1,61
AA418814	AA418814 zw01a02.s1 Soares_NhHMPu_S1 Homo sapiens cDNA clone IMAGE:767978 3', mRNA sequence [AA418814]	9.08	10,68	-1,61
VTI1A	Homo sapiens vesicle transport through interaction with t-SNAREs homolog 1A (yeast) (VTI1A), mRNA [NM_145206]	7.23	8,85	-1,62
TXNDC10	Homo sapiens thioredoxin domain containing 10 (TXNDC10), mRNA [NM_019022]	6.18	7,80	-1,62
TAF10	Homo sapiens TAF10 RNA polymerase II, TATA box binding protein (TBP)-associated factor, 30kDa (TAF10), mRNA [NM_006284]	10.15	11,77	-1,62
SAP30	Homo sapiens Sin3A-associated protein, 30kDa (SAP30), mRNA [NM_003864]	10.73	12,35	-1,62
NAT12	Homo sapiens N-acetyltransferase 12 (NAT12), mRNA [NM_001011713]	6.04	7,65	-1,62
NHLRC2	Homo sapiens mRNA; cDNA DKFZp451L096 (from clone DKFZp451L096). [BX647641]	6.06	7,68	-1,62
GNG5	Homo sapiens guanine nucleotide binding protein (G protein), gamma 5 (GNG5), mRNA [NM_005274]	9.32	10,93	-1,62
EIF2C4	Homo sapiens eukaryotic translation initiation factor 2C, 4 (EIF2C4), mRNA [NM_017629]	6.96	8,58	-1,62
KCTD9	Homo sapiens clone FLB9630 PRO2603 mRNA, complete cds. [AF130091]	7.15	8,77	-1,62
ENST00000368426	Homo sapiens cDNA FLJ45990 fis, clone RECTM2001519. [AK127884]	6.13	7,75	-1,62
-	chr2:108769763-108769822	8.2	9,82	-1,62
-	chr19:000642078-000642137	8.24	9,87	-1,62
-	chr11:016726277-016726218	4.89	6,50	-1,62
BE826587	BE826587 QV1-EN0042-300500-224-e04 EN0042 Homo sapiens cDNA, mRNA sequence [BE826587]	5.4	7,03	-1,62
AW804491	AW804491 QV0-UM0093-170400-191-d05 UM0093 Homo sapiens cDNA, mRNA sequence [AW804491]	7.34	8,96	-1,62
YOD1	Homo sapiens YOD1 OTU deubiquinating enzyme 1 homolog (S. cerevisiae) (YOD1), mRNA [NM_018566]	4.69	6,32	-1,63
TIFA	Homo sapiens TRAF-interacting protein with a forkhead-associated domain (TIFA), mRNA [NM_052864]	7.38	9,01	-1,63
AF116702	Homo sapiens PRO2446 mRNA, complete cds. [AF116702]	6.76	8,39	-1,63
MLSTD2	Homo sapiens male sterility domain containing 2 (MLSTD2), mRNA [NM_032228]	4.77	6,40	-1,63
LSM11	Homo sapiens LSM11, U7 small nuclear RNA associated (LSM11), mRNA [NM_173491]	6.26	7,90	-1,63
KNTC2	Homo sapiens kinetochore associated 2 (KNTC2), mRNA [NM_006101]	10.23	11,86	-1,63
HIST3H2BB	Homo sapiens histone 3, H2bb (HIST3H2BB), mRNA [NM_175055]	9.38	11,01	-1,63
CCNL1	Homo sapiens cyclin L1 (CCNL1), mRNA [NM_020307]	10.16	11,79	-1,63
C14orf138	Homo sapiens chromosome 14 open reading frame 138 (C14orf138), transcript variant 1, mRNA [NM_024558]	8.14	9,77	-1,63
AW858928	AW858928 RC3-CT0347-210400-016-c07 CT0347 Homo sapiens cDNA, mRNA sequence [AW858928]	5.04	6,68	-1,63
AI857589	AI857589 wk95b07.x1 NCI_CGAP_Lu19 Homo sapiens cDNA clone IMAGE:2423125 3' similar to contains Alu repetitive element;contains element MER22 repetitive element ;, mRNA sequence [AI857589]	5.33	6,96	-1,63

Supplemental Table 1
Morandi et al

RP11-114G1.1	Homo sapiens Sp3 transcription factor pseudogene, mRNA (cDNA clone MGC:43345 IMAGE:5266022), complete cds. [BC036697]	6.44	8,08	-1,64
PDK3	Homo sapiens pyruvate dehydrogenase kinase, isozyme 3 (PDK3), mRNA [NM_005391]	5.25	6,90	-1,64
CKLF	Homo sapiens chemokine-like factor (CKLF), transcript variant 4, mRNA [NM_181641]	9.04	10,68	-1,64
AK026485	Homo sapiens cDNA: FLJ22832 fis, clone KAIA4195. [AK026485]	4.64	6,29	-1,64
THC2407230	chr17:52346808-52346867	8.01	9,65	-1,64
TPI1	Homo sapiens triosephosphate isomerase 1 (TPI1), mRNA [NM_000365]	12.75	14,40	-1,65
AY170823	Homo sapiens transformation-related protein 2 mRNA, complete cds. [AY170823]	9.86	11,51	-1,65
PGAP1	Homo sapiens GPI deacylase (PGAP1), mRNA [NM_024989]	5.39	7,05	-1,65
BC007809	Homo sapiens cDNA clone IMAGE:4298560. [BC007809]	8.93	10,58	-1,65
ANKHD1	Homo sapiens ankyrin repeat and KH domain containing 1 (ANKHD1), transcript variant 3, mRNA [NM_024668]	8.73	10,38	-1,65
THC2277728	chr5:122978247-122978231	4.75	6,40	-1,65
BU633383	BU633383 UI-H-FL1-bgu-h-03-0-UI.s1 NCI_CGAP_FL1 Homo sapiens cDNA clone UI-H-FL1-bgu-h-03-0-UI 3', mRNA sequence [BU633383]	9.83	11,48	-1,65
BE816155	BE816155 RC0-BN0198-220500-021-f04 BN0198 Homo sapiens cDNA, mRNA sequence [BE816155]	8.17	9,82	-1,65
BE005242	BE005242 CM1-BN0116-030400-171-h08 BN0116 Homo sapiens cDNA, mRNA sequence [BE005242]	4.92	6,57	-1,65
AA424402	AA424402 zv82e11.s1 Soares_total_fetus_Nb2HF8_9w Homo sapiens cDNA clone IMAGE:760172 3' similar to TR:G483355 G483355 ;, mRNA sequence [AA424402]	9.39	11,05	-1,65
ZSCAN2	Homo sapiens zinc finger and SCAN domain containing 2 (ZSCAN2), transcript variant 2, mRNA [NM_017894]	7.37	9,03	-1,66
SYNCRIP	Homo sapiens synaptotagmin binding, cytoplasmic RNA interacting protein (SYNCRIP), mRNA [NM_006372]	10.19	11,85	-1,66
SHCBP1	Homo sapiens SHC SH2-domain binding protein 1 (SHCBP1), mRNA [NM_024745]	5.99	7,65	-1,66
NR2C2	Homo sapiens nuclear receptor subfamily 2, group C, member 2 (NR2C2), mRNA [NM_003298]	6.71	8,38	-1,66
NR2C1	Homo sapiens nuclear receptor subfamily 2, group C, member 1 (NR2C1), transcript variant 1, mRNA [NM_003297]	7.25	8,91	-1,66
EIF4EBP1	Homo sapiens eukaryotic translation initiation factor 4E binding protein 1 (EIF4EBP1), mRNA [NM_004095]	11.31	12,97	-1,66
ENST00000379884	Homo sapiens clone MC56H myosin-reactive immunoglobulin heavy chain variable region mRNA, partial cds. [AF035022]	6.53	8,18	-1,66
C14orf145	Homo sapiens chromosome 14 open reading frame 145 (C14orf145), mRNA [NM_152446]	6.07	7,72	-1,66
-	chr2:038424427-038424486	6.16	7,82	-1,66
-	chr1:231821170-231821111	5.7	7,35	-1,66
ZMYM5	Homo sapiens zinc finger, MYM-type 5 (ZMYM5), transcript variant 1, mRNA [NM_001039650]	6.86	8,53	-1,67
RFC5	Homo sapiens replication factor C (activator 1) 5, 36.5kDa (RFC5), transcript variant 2, mRNA [NM_181578]	9.29	10,96	-1,67
PHTF2	Homo sapiens putative homeodomain transcription factor 2 (PHTF2), mRNA [NM_020432]	5.88	7,55	-1,67
FLJ25416	Homo sapiens hypothetical protein FLJ25416 (FLJ25416), mRNA [NM_145018]	6.6	8,28	-1,67
-	chrX:067774405-067774464	6.6	8,27	-1,67
WBSCR16	Homo sapiens Williams-Beuren syndrome chromosome region 16, mRNA (cDNA clone MGC:49849 IMAGE:5785681), complete cds. [BC040695]	5.34	7,02	-1,68
TMPO	Homo sapiens thymopoietin (TMPO), transcript variant 2, mRNA [NM_001032283]	6.32	8,00	-1,68
STK38L	Homo sapiens serine/threonine kinase 38 like (STK38L), mRNA [NM_015000]	7.46	9,14	-1,68
PCGF5	Homo sapiens polycomb group ring finger 5, mRNA (cDNA clone IMAGE:3640258), complete cds. [BC007377]	5.84	7,52	-1,68

Supplemental Table 1
Morandi et al

BC000604	Homo sapiens mRNA similar to cisplatin resistance-associated overexpressed protein (cDNA clone IMAGE:3346964). [BC000604]	5.1	6,78	-1,68
KPNA2	Homo sapiens karyopherin alpha 2 (RAG cohort 1, importin alpha 1) (KPNA2), mRNA [NM_002266]	12.97	14,65	-1,68
IL17RA	Homo sapiens interleukin 17 receptor A (IL17RA), mRNA [NM_014339]	8.68	10,36	-1,68
FLJ20186	Homo sapiens hypothetical protein FLJ20186 (FLJ20186), transcript variant 2, mRNA [NM_017702]	9.68	11,36	-1,68
FANCB	Homo sapiens Fanconi anemia, complementation group B (FANCB), transcript variant 2, mRNA [NM_152633]	5.59	7,27	-1,68
BCL2L13	Homo sapiens BCL2-like 13 (apoptosis facilitator) (BCL2L13), nuclear gene encoding mitochondrial protein, mRNA [NM_015367]	6.39	8,07	-1,68
ABHD5	Homo sapiens abhydrolase domain containing 5 (ABHD5), mRNA [NM_016006]	7.69	9,38	-1,68
-	chr5:115926217-115926276	10.48	12,16	-1,68
RNF141	Homo sapiens ring finger protein 141 (RNF141), mRNA [NM_016422]	8.43	10,12	-1,69
PIAS2	Homo sapiens protein inhibitor of activated STAT, 2 (PIAS2), transcript variant beta, mRNA [NM_004671]	7.29	8,98	-1,69
AL136830	Homo sapiens mRNA; cDNA DKFZp434D1428 (from clone DKFZp434D1428). [AL136830]	5.09	6,78	-1,69
TMC8	Homo sapiens mRNA for FLJ00400 protein. [AK090478]	8.47	10,16	-1,69
DA180164	DA180164 BRAMY2 Homo sapiens cDNA clone BRAMY2044797 5', mRNA sequence [DA180164]	4.86	6,55	-1,69
-	chr6:054597720-054597781	8.03	9,71	-1,69
THC2342810	chr1:168187097-168187038	5.1	6,79	-1,69
CA948108	CA948108 iq21e07.x1 HR85 islet Homo sapiens cDNA clone IMAGE: 3', mRNA sequence [CA948108]	4.85	6,55	-1,69
THC2282018	Q9F8M7 (Q9F8M7) DTDP-glucose 4,6-dehydratase (Fragment), partial (11%) [THC2282018]	5.6	7,30	-1,70
ZBTB7B	Homo sapiens zinc finger and BTB domain containing 7B (ZBTB7B), mRNA [NM_015872]	6.12	7,82	-1,70
PTTG3	Homo sapiens pituitary tumor-transforming 3 (PTTG3) on chromosome 8 [NR_002734]	5.39	7,09	-1,70
MSI2	Homo sapiens musashi homolog 2 (Drosophila) (MSI2), transcript variant 1, mRNA [NM_138962]	7.77	9,47	-1,70
MYBL1	H.sapiens a-myb mRNA. [X66087]	7.63	9,33	-1,70
-	chr19:063419579-063419638	5.95	7,66	-1,70
-	chr15:041347566-041347505	8.88	10,58	-1,70
-	chr11:017171617-017171676	10.69	12,39	-1,70
BE970058	BE970058 601680172F1 NIH_MGC_78 Homo sapiens cDNA clone IMAGE:3950464 5', mRNA sequence [BE970058]	8.62	10,32	-1,70
THC2407148	Q6D545 (Q6D545) Transposase transposon tn1721 (Fragment), partial (12%) [THC2407148]	7.16	8,87	-1,71
ENST00000361500	PREDICTED: Homo sapiens similar to nucleolar protein family A, member 3 (LOC645738), mRNA [XM_928749]	8.09	9,80	-1,71
ZNF273	Homo sapiens zinc finger protein 273 (ZNF273), transcript variant 1, mRNA [NM_021148]	5.24	6,95	-1,71
TNFRSF19L	Homo sapiens tumor necrosis factor receptor superfamily, member 19-like (TNFRSF19L), transcript variant 1, mRNA [NM_032871]	6.32	8,03	-1,71
PRR8	Homo sapiens proline rich 8, mRNA (cDNA clone MGC:20460 IMAGE:4542314), complete cds. [BC011923]	6.7	8,41	-1,71
AF116620	Homo sapiens PRO1068 mRNA, complete cds. [AF116620]	4.66	6,37	-1,71
MCFD2	Homo sapiens multiple coagulation factor deficiency 2 (MCFD2), mRNA [NM_139279]	8.1	9,81	-1,71
-	chr12:007610833-007610969	6.7	8,42	-1,71
CK570365	c50-1 cDNA subtractive library of human rectum adencarcinoma Homo sapiens cDNA, mRNA sequence [CK570365]	8.14	9,85	-1,71
AW291149	UI-H-BI2-agb-c-11-0-UI.s1 NCI_CGAP_Sub4 Homo sapiens cDNA clone IMAGE:2723709 3', mRNA sequence [AW291149]	6.68	8,40	-1,72

BC015962	Homo sapiens, clone IMAGE:4081125, mRNA, partial cds. [BC015962]	8.24	9,96	-1,72
WDR76	Homo sapiens WD repeat domain 76 (WDR76), mRNA [NM_024908]	4.8	6,53	-1,72
UHRF1	Homo sapiens ubiquitin-like, containing PHD and RING finger domains, 1 (UHRF1), mRNA [NM_013282]	5.9	7,62	-1,72
-	chr17:040946223-040946159	4.89	6,61	-1,72
-	chr10:044888819-044888878	7.85	9,57	-1,72
THC2338243	Q6PGA0 (Q6PGA0) Rcor3 protein (Fragment), partial (8%) [THC2338243]	7.16	8,89	-1,73
FLJ39660	Homo sapiens mRNA; cDNA DKFZp434P055 (from clone DKFZp434P055). [AL834537]	7.02	8,75	-1,73
ENST00000356931	Homo sapiens cDNA FLJ43464 fis, clone OCBBF2036225. [AK125453]	6.73	8,46	-1,73
SPECC1	Homo sapiens spectrin domain with coiled-coils 1 (SPECC1), transcript variant NSP5beta3alpha, mRNA [NM_152904]	8.63	10,37	-1,74
SKIL	Homo sapiens SKI-like (SKIL), mRNA [NM_005414]	6.37	8,10	-1,74
PRKAA1	Homo sapiens protein kinase, AMP-activated, alpha 1 catalytic subunit (PRKAA1), transcript variant 2, mRNA [NM_206907]	7.24	8,98	-1,74
HIST1H2AJ	Homo sapiens histone 1, H2aj (HIST1H2AJ), mRNA [NM_021066]	7.79	9,53	-1,74
DHFR	Homo sapiens dihydrofolate reductase (DHFR), mRNA [NM_000791]	5.56	7,30	-1,74
C15orf20	Homo sapiens chromosome 15 open reading frame 20 (C15orf20), mRNA [NM_025049]	8.89	10,63	-1,74
-	chr4:153830138-153830079	9.23	10,97	-1,74
-	chr2:232145329-232145388	6.8	8,54	-1,74
-	chr1:042626846-042626905	9.88	11,63	-1,74
BE696323	BE696323 RC3-CT0347-010700-019-c08 CT0347 Homo sapiens cDNA, mRNA sequence [BE696323]	5.32	7,06	-1,74
AA291137	AA291137 zs46b08.s1 NCI_CGAP_GCB1 Homo sapiens cDNA clone IMAGE:700503 3', mRNA sequence [AA291137]	5.62	7,36	-1,74
AK021546	Homo sapiens cDNA FLJ11484 fis, clone HEMBA1001835. [AK021546]	5.33	7,09	-1,75
CNOT6	Homo sapiens CCR4-NOT transcription complex, subunit 6 (CNOT6), mRNA [NM_015455]	5.48	7,23	-1,75
-	chr7:072138467-072138408	5.49	7,23	-1,75
-	chr6:114290669-114290610	7.98	9,74	-1,75
-	chr13:049571595-049571654	7.06	8,81	-1,75
BQ374929	BQ374929 MR1-TN0045-060900-005-d11 TN0045 Homo sapiens cDNA, mRNA sequence [BQ374929]	9.84	11,59	-1,75
BE156557	BE156557 QV0-HT0368-090200-099-d10 HT0368 Homo sapiens cDNA, mRNA sequence [BE156557]	6.13	7,88	-1,75
MXD1	Homo sapiens MAX dimerization protein 1 (MXD1), mRNA [NM_002357]	10.72	12,48	-1,76
LRRC57	Homo sapiens leucine rich repeat containing 57 (LRRC57), mRNA [NM_153260]	7.16	8,92	-1,76
KLHDC7B	Homo sapiens kelch domain containing 7B (KLHDC7B), mRNA [NM_138433]	6.78	8,54	-1,76
ENST00000269290	Homo sapiens HSPC254 mRNA, partial cds. [AF161372]	6.83	8,59	-1,76
EIF4A2	Homo sapiens eukaryotic translation initiation factor 4A, isoform 2, mRNA (cDNA clone IMAGE:5269135). [BC039344]	4.62	6,38	-1,76
DBF4	Homo sapiens DBF4 homolog (S. cerevisiae) (DBF4), mRNA [NM_006716]	7.05	8,81	-1,76
CENPH	Homo sapiens centromere protein H (CENPH), mRNA [NM_022909]	7.67	9,43	-1,76
BM968705	BM968705 UI-CF-DU1-aak-f-20-0-UI.s1 UI-CF-DU1 Homo sapiens cDNA clone UI-CF-DU1-aak-f-20-0-UI 3', mRNA sequence [BM968705]	5.62	7,38	-1,76
AA065042	AA065042 zm12g12.s1 Stratagene pancreas (#937208) Homo sapiens cDNA clone IMAGE:525478 3', mRNA sequence [AA065042]	4.94	6,71	-1,76
RPE	Homo sapiens ribulose-5-phosphate-3-epimerase (RPE), transcript variant 2, mRNA [NM_006916]	8.09	9,86	-1,77

Supplemental Table 1
Morandi et al

MDM4	Homo sapiens Mdm4, transformed 3T3 cell double minute 4, p53 binding protein (mouse) (MDM4), mRNA [NM_002393]	8.13	9,90	-1,77
FAM8A1	Homo sapiens family with sequence similarity 8, member A1 (FAM8A1), mRNA [NM_016255]	5.07	6,84	-1,77
C15orf42	Homo sapiens chromosome 15 open reading frame 42, mRNA (cDNA clone IMAGE:3940845), partial cds. [BC002881]	4.82	6,59	-1,77
SMCHD1	Homo sapiens cDNA FLJ44350 fis, clone TRACH3006228. [AK126324]	9.09	10,86	-1,77
APBB1IP	Homo sapiens amyloid beta (A4) precursor protein-binding, family B, member 1 interacting protein (APBB1IP), mRNA [NM_019043]	8.84	10,61	-1,77
ZCSL2	Homo sapiens zinc finger, CSL-type containing 2 (ZCSL2), mRNA [NM_206831]	8.33	10,11	-1,78
TRIM59	Homo sapiens tripartite motif-containing 59 (TRIM59), mRNA [NM_173084]	7.59	9,37	-1,78
CDKN2C	Homo sapiens cyclin-dependent kinase inhibitor 2C (p18, inhibits CDK4) (CDKN2C), transcript variant 1, mRNA [NM_001262]	11.43	13,20	-1,78
CR613972	full-length cDNA clone CS0DI009YA14 of Placenta Cot 25-normalized of Homo sapiens (human). [CR613972]	9.78	11,56	-1,78
AL562818	AL562818 Homo sapiens NEUROBLASTOMA COT 25-NORMALIZED Homo sapiens cDNA clone CS0DC017YH14 3-PRIME, mRNA sequence [AL562818]	5.54	7,32	-1,78
AI885257	AI885257 wl91f02.x1 NCI_CGAP_Brn25 Homo sapiens cDNA clone IMAGE:2432283 3', mRNA sequence [AI885257]	4.39	6,16	-1,78
AK090405	Homo sapiens mRNA for FLJ00291 protein. [AK090405]	5.52	7,31	-1,79
HIST1H3D	Homo sapiens histone 1, H3d (HIST1H3D), mRNA [NM_003530]	8.79	10,58	-1,79
CR617865	full-length cDNA clone CS0DF021YD16 of Fetal brain of Homo sapiens (human). [CR617865]	7.17	8,96	-1,79
PPP4R2	Homo sapiens protein phosphatase 4, regulatory subunit 2 (PPP4R2), mRNA [NM_174907]	5.89	7,69	-1,80
KIAA1794	Homo sapiens KIAA1794 (KIAA1794), mRNA [NM_018193]	9.2	11,00	-1,80
EAF2	Homo sapiens ELL associated factor 2 (EAF2), mRNA [NM_018456]	6.31	8,11	-1,80
EHD1	Homo sapiens EH-domain containing protein testilin mRNA, complete cds. [AF099011]	6.95	8,75	-1,80
-	chr5:032258029-032258093	7.07	8,87	-1,80
ENST00000382990	chr16:221400-221459	4.64	6,44	-1,80
CA314451	CA314451 UI-CF-FN0-afh-a-09-0-UI.s1 UI-CF-FN0 Homo sapiens cDNA clone UI-CF-FN0-afh-a-09-0-UI 3', mRNA sequence [CA314451]	7.94	9,74	-1,80
BX460543	BX460543 BX460543 Homo sapiens FETAL BRAIN Homo sapiens cDNA clone CS0DF015YP13 3-PRIME, mRNA sequence [BX460543]	4.87	6,67	-1,80
AI887037	wl96a02.x1 NCI_CGAP_Brn25 Homo sapiens cDNA clone IMAGE:2432714 3' similar to gb:J03827 Y BOX BINDING PROTEIN-1 (HUMAN);, mRNA sequence [AI887037]	6.35	8,16	-1,81
NFKBIZ	Homo sapiens nuclear factor of kappa light polypeptide gene enhancer in B-cells inhibitor, zeta (NFKBIZ), transcript variant 1, mRNA [NM_031419]	8.23	10,04	-1,81
GLT28D1	Homo sapiens glycosyltransferase 28 domain containing 1 (GLT28D1), mRNA [NM_018466]	8.06	9,87	-1,81
EIF2C2	Homo sapiens eukaryotic translation initiation factor 2C, 2 (EIF2C2), mRNA [NM_012154]	8.96	10,77	-1,81
MYCBP	Homo sapiens c-myc binding protein (MYCBP), mRNA [NM_012333]	7.84	9,65	-1,81
CD44	Homo sapiens CD44 molecule (Indian blood group) (CD44), transcript variant 1, mRNA [NM_000610]	11.28	13,08	-1,81
-	chr15:059932948-059933007	5.64	7,45	-1,81
BU753102	BU753102 UI-1-BB1-aii-b-10-0-UI.s1 NCI_CGAP_Pl5 Homo sapiens cDNA clone UI-1-BB1-aii-b-10-0-UI 3', mRNA sequence [BU753102]	4.54	6,35	-1,81
THC2274391	RIFK_HUMAN (Q969G6) Riboflavin kinase (ATP:riboflavin 5'-phosphotransferase) (Flavokinase) , complete [THC2274391]	8.05	9,86	-1,82

Supplemental Table 1
Morandi et al

THC2402213	Q8CHU4 (Q8CHU4) Pi16 protein (Fragment), partial (5%) [THC2402213]	5.17	6,98	-1,82
ODC1	Homo sapiens ornithine decarboxylase 1 (ODC1), mRNA [NM_002539]	12.71	14,53	-1,82
KTI12	Homo sapiens KTI12 homolog, chromatin associated (S. cerevisiae) (KTI12), mRNA [NM_138417]	6.46	8,28	-1,82
HMGB2	Homo sapiens high-mobility group box 2 (HMGB2), mRNA [NM_002129]	13.31	15,13	-1,82
FTHL17	Homo sapiens ferritin, heavy polypeptide-like 17 (FTHL17), mRNA [NM_031894]	9.97	11,78	-1,82
C14orf32	Homo sapiens chromosome 14 open reading frame 32 (C14orf32), mRNA [NM_144578]	7.71	9,53	-1,82
CEBPG	Homo sapiens CCAAT/enhancer binding protein (C/EBP), gamma (CEBPG), mRNA [NM_001806]	9.93	11,75	-1,82
ATP11B	Homo sapiens ATPase, Class VI, type 11B (ATP11B), mRNA [NM_014616]	6.76	8,58	-1,82
-	chr12:051687676-051687617	4.87	6,70	-1,82
HIF1A	Homo sapiens hypoxia-inducible factor 1, alpha subunit (basic helix-loop-helix transcription factor) (HIF1A), transcript variant 2, mRNA [NM_181054]	9.51	11,34	-1,83
HIST3H2A	Homo sapiens histone 3, H2a (HIST3H2A), mRNA [NM_033445]	10.38	12,21	-1,83
ECT2	Homo sapiens epithelial cell transforming sequence 2 oncogene (ECT2), mRNA [NM_018098]	9.93	11,77	-1,83
C10orf54	Homo sapiens chromosome 10 open reading frame 54 (C10orf54), mRNA [NM_022153]	9.43	11,26	-1,83
CENPE	Homo sapiens centromere protein E, 312kDa (CENPE), mRNA [NM_001813]	8.08	9,91	-1,83
CB306038	CB306038 UI-CF-EN1-aeh-b-15-0-UI.s1 UI-CF-EN1 Homo sapiens cDNA clone UI-CF-EN1-aeh-b-15-0-UI 3', mRNA sequence [CB306038]	8.11	9,94	-1,83
TIA1	Homo sapiens TIA1 cytotoxic granule-associated RNA binding protein (TIA1), transcript variant 1, mRNA [NM_022037]	8.19	10,03	-1,84
IMPACT	Homo sapiens Impact homolog (mouse) (IMPACT), mRNA [NM_018439]	8.09	9,93	-1,84
TCP11L2	Homo sapiens t-complex 11 (mouse) like 2 (TCP11L2), mRNA [NM_152772]	5.14	6,99	-1,85
RNASEH2A	Homo sapiens ribonuclease H2, large subunit (RNASEH2A), mRNA [NM_006397]	10.12	11,98	-1,85
ORC6L	Homo sapiens origin recognition complex, subunit 6 like (yeast) (ORC6L), mRNA [NM_014321]	7.6	9,45	-1,85
NUDT1	Homo sapiens nudix (nucleoside diphosphate linked moiety X)-type motif 1 (NUDT1), transcript variant 2B, mRNA [NM_198949]	11.59	13,44	-1,85
HIST1H2BE	Homo sapiens histone 1, H2be (HIST1H2BE), mRNA [NM_003523]	9.99	11,83	-1,85
CKAP2	Homo sapiens cytoskeleton associated protein 2 (CKAP2), mRNA [NM_018204]	9.17	11,01	-1,85
ENST00000256031	Homo sapiens cDNA FLJ90613 fis, clone PLACE1001949, highly similar to Probable cation-transporting ATPase 3 (EC3.6.3.-). [AK075094]	5.43	7,28	-1,85
ATP11C	Homo sapiens ATPase, Class VI, type 11C (ATP11C), transcript variant 2, mRNA [NM_001010986]	5.24	7,10	-1,85
THC2281304	chr21:33618456-33618397	6.97	8,82	-1,85
ENST00000323198	chr15:29499977-29500036	4.65	6,49	-1,85
AI922845	wo14g02.x1 NCI_CGAP_Pan1 Homo sapiens cDNA clone IMAGE:2455346 3' similar to gb:J04543 ANNEXIN VII (HUMAN);, mRNA sequence [AI922845]	6.23	8,09	-1,86
PBEF1	Homo sapiens pre-B-cell colony enhancing factor 1 (PBEF1), transcript variant 1, mRNA [NM_005746]	9.82	11,67	-1,86
FGD3	Homo sapiens FYVE, RhoGEF and PH domain containing 3 (FGD3), mRNA [NM_033086]	5.13	6,99	-1,86
FABP5	Homo sapiens fatty acid binding protein 5 (psoriasis-associated) (FABP5), mRNA [NM_001444]	7.27	9,12	-1,86
C16orf75	Homo sapiens chromosome 16 open reading frame 75 (C16orf75), mRNA [NM_152308]	9.49	11,35	-1,86

C15orf23	Homo sapiens chromosome 15 open reading frame 23, mRNA (cDNA clone IMAGE:3952251), partial cds. [BC004543]	10.12	11,98	-1,86
CEP152	Homo sapiens centrosomal protein 152kDa (CEP152), mRNA [NM_014985]	6.36	8,21	-1,86
CDC40	Homo sapiens cell division cycle 40 homolog (S. cerevisiae) (CDC40), mRNA [NM_015891]	6.94	8,80	-1,86
-	chr2:204462936-204462877	5.63	7,49	-1,86
BI009763	BI009763 MR0-RT0026-160401-104-h09 RT0026 Homo sapiens cDNA, mRNA sequence [BI009763]	6.12	7,98	-1,86
AL079999	AL079999 DKFZp586P2018_r1 586 (synonym: hute1) Homo sapiens cDNA clone DKFZp586P2018 5', mRNA sequence [AL079999]	5.45	7,31	-1,86
UBE2T	Homo sapiens ubiquitin-conjugating enzyme E2T (putative) (UBE2T), mRNA [NM_014176]	11.17	13,04	-1,87
TTC32	Homo sapiens tetratricopeptide repeat domain 32 (TTC32), mRNA [NM_001008237]	9.6	11,47	-1,87
CRSP3	Homo sapiens cofactor required for Sp1 transcriptional activation, subunit 3, 130kDa (CRSP3), transcript variant 2, mRNA [NM_015979]	5.63	7,50	-1,87
CEBPB	Homo sapiens CCAAT/enhancer binding protein (C/EBP), beta (CEBPB), mRNA [NM_005194]	12.85	14,72	-1,87
ZNF197	Homo sapiens zinc finger protein 197 (ZNF197), transcript variant 1, mRNA [NM_006991]	6.1	7,98	-1,88
TSPO	Homo sapiens translocator protein (18kDa) (TSPO), transcript variant PBR-S, mRNA [NM_007311]	9.69	11,57	-1,88
SMC4	Homo sapiens structural maintenance of chromosomes 4 (SMC4), transcript variant 1, mRNA [NM_005496]	8.73	10,61	-1,88
RP6-213H19.1	Homo sapiens serine/threonine protein kinase MST4 (MST4), transcript variant 1, mRNA [NM_016542]	7.97	9,85	-1,88
GMNN	Homo sapiens geminin, DNA replication inhibitor (GMNN), mRNA [NM_015895]	10.38	12,27	-1,88
TSPAN10	Homo sapiens tetraspanin 10 (TSPAN10), mRNA [NM_031945]	11.81	13,71	-1,89
SIN3B	Homo sapiens SIN3 homolog B, transcription regulator (yeast), mRNA (cDNA clone IMAGE:4417458), complete cds. [BC063531]	7.56	9,44	-1,89
SEPT9	Homo sapiens septin 9 (SEPT9), mRNA [NM_006640]	6.52	8,41	-1,89
IER5	Homo sapiens immediate early response 5 (IER5), mRNA [NM_016545]	12.7	14,59	-1,89
TTYH2	Homo sapiens cDNA clone IMAGE:3834434, **** WARNING: chimeric clone ****. [BC021035]	8.4	10,28	-1,89
-	chr11:087781236-087781175	10.22	12,11	-1,89
-	chr10:028765637-028765696	10.13	12,02	-1,89
BF378976	BF378976 QV1-UM0099-020400-141-b12_1 UM0099 Homo sapiens cDNA, mRNA sequence [BF378976]	8.12	10,01	-1,89
BC020539	Homo sapiens, clone IMAGE:3048919, mRNA, partial cds. [BC020539]	8.75	10,65	-1,90
TRIO	Homo sapiens triple functional domain (PTPRF interacting) (TRIO), mRNA [NM_007118]	6.23	8,13	-1,90
PLP2	Homo sapiens proteolipid protein 2 (colonic epithelium-enriched) (PLP2), mRNA [NM_002668]	8.47	10,37	-1,90
NFIL3	Homo sapiens nuclear factor, interleukin 3 regulated (NFIL3), mRNA [NM_005384]	10.76	12,67	-1,90
MTP18	Homo sapiens mitochondrial protein 18 kDa (MTP18), nuclear gene encoding mitochondrial protein, transcript variant 1, mRNA [NM_016498]	7.55	9,44	-1,90
ID2	Homo sapiens inhibitor of DNA binding 2, dominant negative helix-loop-helix protein (ID2), mRNA [NM_002166]	10.59	12,49	-1,90
HIST1H2BN	Homo sapiens histone 1, H2bn (HIST1H2BN), mRNA [NM_003520]	8.89	10,79	-1,90
C20orf106	Homo sapiens chromosome 20 open reading frame 106 (C20orf106), mRNA [NM_001012971]	6.55	8,45	-1,90
AA780798	ag14d07.s1 Gessler Wilms tumor Homo sapiens cDNA clone IMAGE:1070317 3' similar to gb:J00314_rna2 TUBULIN BETA-1 CHAIN (HUMAN);, mRNA sequence [AA780798]	8.68	10,58	-1,90
HDAC4	Homo sapiens histone deacetylase 4 (HDAC4), mRNA [NM_006037]	6.14	8,04	-1,91

Supplemental Table 1
Morandi et al

SERPINB1	Homo sapiens serpin peptidase inhibitor, clade B (ovalbumin), member 1 (SERPINB1), mRNA [NM_030666]	9.83	11,75	-1,92
PA2G4	Homo sapiens proliferation-associated 2G4, 38kDa (PA2G4), mRNA [NM_006191]	5.08	6,99	-1,92
MAX	Homo sapiens MYC associated factor X (MAX), transcript variant 4, mRNA [NM_145114]	4.91	6,83	-1,92
MAP3K7IP3	Homo sapiens mitogen-activated protein kinase kinase kinase 7 interacting protein 3 (MAP3K7IP3), mRNA [NM_152787]	4.27	6,19	-1,92
G0S2	Homo sapiens G0/G1switch 2 (G0S2), mRNA [NM_015714]	10.88	12,80	-1,92
ADRBK1	Homo sapiens adrenergic, beta, receptor kinase 1 (ADRBK1), mRNA [NM_001619]	7.92	9,84	-1,92
T12588	CHR90108 Chromosome 9 exon II Homo sapiens cDNA clone P94_53 5' and 3', mRNA sequence [T12588]	10.44	12,36	-1,92
MGC23909	Homo sapiens hypothetical protein MGC23909 (MGC23909), mRNA [NM_174909]	8.41	10,35	-1,93
THC2307560	chr6:30799814-30799755	8.04	9,97	-1,93
-	chr6:026164200-026164151	9.39	11,32	-1,93
THC2292160	chr14:080756606-080756665	8.28	10,20	-1,93
ENST00000229270	Human triosephosphate isomerase mRNA, complete cds. [M10036]	10.26	12,19	-1,94
MNAB	Homo sapiens mRNA; cDNA DKFZp667B165 (from clone DKFZp667B165). [AL833177]	5.29	7,23	-1,94
HIST2H2AC	Homo sapiens histone 2, H2ac (HIST2H2AC), mRNA [NM_003517]	8.78	10,72	-1,94
HIST1H2BF	Homo sapiens histone 1, H2bf (HIST1H2BF), mRNA [NM_003522]	10.19	12,13	-1,94
RIN3	Homo sapiens cDNA FLJ11700 fis, clone HEMBA1005050. [AK021762]	5.91	7,85	-1,94
THC2282717	Q87726 (Q87726) Glycoprotein 120 (Fragment), partial (13%) [THC2282717]	5.98	7,93	-1,95
SPIB	Homo sapiens Spi-B transcription factor (Spi-1/PU.1 related) (SPIB), mRNA [NM_003121]	6.71	8,65	-1,95
PRC1	Homo sapiens protein regulator of cytokinesis 1 (PRC1), transcript variant 1, mRNA [NM_003981]	10.61	12,56	-1,95
HIST1H2BL	Homo sapiens histone 1, H2bl (HIST1H2BL), mRNA [NM_003519]	10.9	12,85	-1,95
CDKN3	Homo sapiens cyclin-dependent kinase inhibitor 3 (CDK2-associated dual specificity phosphatase) (CDKN3), mRNA [NM_005192]	8.36	10,31	-1,95
PRO1073	Homo sapiens clone alpha_est218/52C1 mRNA sequence. [AF001542]	7.75	9,70	-1,95
C10orf12	Homo sapiens chromosome 10 open reading frame 12 (C10orf12), mRNA [NM_015652]	4.31	6,26	-1,95
ENST00000357303	Homo sapiens cDNA FLJ37034 fis, clone BRACE2011478. [AK094353]	5.13	7,08	-1,95
CCRN4L	Homo sapiens CCR4 carbon catabolite repression 4-like (S. cerevisiae) (CCRN4L), mRNA [NM_012118]	5.76	7,71	-1,95
-	chr2:058390987-058390928	4.71	6,66	-1,95
AI537201	AI537201 tp06e06.x1 NCI_CGAP_Gas4 Homo sapiens cDNA clone IMAGE:2187010 3', mRNA sequence [AI537201]	8.25	10,20	-1,95
SUSD3	Homo sapiens sushi domain containing 3 (SUSD3), mRNA [NM_145006]	9.06	11,02	-1,96
SERP1	Homo sapiens stress-associated endoplasmic reticulum protein 1 (SERP1), mRNA [NM_014445]	5.82	7,78	-1,96
HIST1H2BM	Homo sapiens histone 1, H2bm (HIST1H2BM), mRNA [NM_003521]	8.31	10,27	-1,96
GMFB	Homo sapiens glia maturation factor, beta (GMFB), mRNA [NM_004124]	6.12	8,07	-1,96
FAM54A	Homo sapiens family with sequence similarity 54, member A (FAM54A), mRNA [NM_138419]	6.46	8,42	-1,96
-	chrX:110673968-110673909	10.99	12,95	-1,96
SPBC25	Homo sapiens spindle pole body component 25 homolog (S. cerevisiae) (SPBC25), mRNA [NM_020675]	7.62	9,59	-1,97
ROPN1L	Homo sapiens ropporin 1-like (ROPN1L), mRNA [NM_031916]	5.74	7,71	-1,97
PTP4A1	Homo sapiens protein tyrosine phosphatase type IVA, member 1 (PTP4A1), mRNA [NM_003463]	6.44	8,41	-1,97

AJ293393	Homo sapiens mRNA differentially expressed in malignant melanoma, clone MM D3. [AJ293393]	5.84	7,81	-1,97
CDC2	Homo sapiens cell division cycle 2, G1 to S and G2 to M (CDC2), transcript variant 1, mRNA [NM_001786]	8.11	10,08	-1,97
MFSD2	Homo sapiens cDNA FLJ14490 fis, clone MAMMA1002886. [AK027396]	4.56	6,53	-1,97
-	chr3:151184905-151184968	5.59	7,56	-1,97
-	chr22:019630306-019630247	5.69	7,65	-1,97
THC2301371	Q864S5 (Q864S5) Peptidylprolyl isomerase A, partial (91%) [THC2301371]	6.95	8,93	-1,98
SGOL1	Homo sapiens shugoshin-like 1 (S. pombe) (SGOL1), transcript variant A1, mRNA [NM_001012409]	5.32	7,30	-1,98
HIST2H2BE	Homo sapiens histone 2, H2be (HIST2H2BE), mRNA [NM_003528]	8.12	10,10	-1,98
HIST1H2AH	Homo sapiens histone 1, H2ah (HIST1H2AH), mRNA [NM_080596]	10.14	12,12	-1,98
GALR3	Homo sapiens galanin receptor 3 (GALR3), mRNA [NM_003614]	12.66	14,64	-1,98
CA420826	CA420826 UI-H-FG0-bcr-c-23-0-UI.s1 NCI_CGAP_EN1_2 Homo sapiens cDNA clone UI-H-FG0-bcr-c-23-0-UI 3', mRNA sequence [CA420826]	6.24	8,23	-1,98
BE646426	BE646426 7e86f01.x1 NCI_CGAP_CLL1 Homo sapiens cDNA clone IMAGE:3292057 3', mRNA sequence [BE646426]	9.25	11,23	-1,98
ZCCHC6	Homo sapiens zinc finger, CCHC domain containing 6 (ZCCHC6), mRNA [NM_024617]	5.05	7,04	-1,99
KLF6	Homo sapiens Kruppel-like factor 6 (KLF6), transcript variant 1, mRNA [NM_001008490]	6.54	8,53	-1,99
GOPC	Homo sapiens golgi associated PDZ and coiled-coil motif containing (GOPC), transcript variant 1, mRNA [NM_020399]	6.81	8,80	-1,99
EMP3	Homo sapiens epithelial membrane protein 3 (EMP3), mRNA [NM_001425]	9.32	11,31	-1,99
C11orf17	Homo sapiens chromosome 11 open reading frame 17 (C11orf17), transcript variant 1, mRNA [NM_182901]	7.16	9,14	-1,99
BAG4	Homo sapiens BCL2-associated athanogene 4 (BAG4), mRNA [NM_004874]	4.55	6,54	-1,99
-	chr22:018087351-018087292	12.11	14,10	-1,99
RPL39L	Homo sapiens ribosomal protein L39-like (RPL39L), mRNA [NM_052969]	8.71	10,71	-2,00
HIST1H2BO	Homo sapiens histone 1, H2bo (HIST1H2BO), mRNA [NM_003527]	9.72	11,72	-2,00
ENST00000330189	chr10:76519397-76519338	7.09	9,09	-2,00
AU184995	AU184995 AU184995 Homo sapiens T-cell library (Sugita Y) Homo sapiens cDNA clone B00751-019, mRNA sequence [AU184995]	6.08	8,08	-2,00
AL555100	AL555100 AL555100 Homo sapiens HELA CELLS COT 25-NORMALIZED Homo sapiens cDNA clone CS0DK007YP02 3-PRIME, mRNA sequence [AL555100]	8.01	10,02	-2,01
WDR62	Homo sapiens WD repeat domain 62 (WDR62), mRNA [NM_173636]	6.81	8,83	-2,02
RAP2C	Homo sapiens RAP2C, member of RAS oncogene family (RAP2C), mRNA [NM_021183]	5.23	7,25	-2,02
PPP1R15B	Homo sapiens protein phosphatase 1, regulatory (inhibitor) subunit 15B (PPP1R15B), mRNA [NM_032833]	5.79	7,81	-2,02
HIST1H2BK	Homo sapiens histone 1, H2bk (HIST1H2BK), mRNA [NM_080593]	8.88	10,90	-2,02
CR620804	full-length cDNA clone CS0DL005YE02 of B cells (Ramos cell line) Cot 25-normalized of Homo sapiens (human). [CR620804]	5.22	7,24	-2,02
-	chr15:056766772-056766831	6.39	8,41	-2,02
RAC2	Homo sapiens ras-related C3 botulinum toxin substrate 2 (rho family, small GTP binding protein Rac2) (RAC2), mRNA [NM_002872]	10.59	12,62	-2,03
NRAS	Homo sapiens neuroblastoma RAS viral (v-ras) oncogene homolog (NRAS), mRNA [NM_002524]	7.06	9,09	-2,03

Supplemental Table 1
Morandi et al

CF529502	CF529502 UI-1-BC1p-ash-d-10-0-UI.s1 NCI_CGAP_Pl3 Homo sapiens cDNA clone UI-1-BC1p-ash-d-10-0-UI 3', mRNA sequence [CF529502]	9,00	11,03	-2,03
AI216457	qh07g09.x1 Soares_NFL_T_GBC_S1 Homo sapiens cDNA clone IMAGE:1844032 3' similar to SW:RL39_HUMAN P02404 60S RIBOSOMAL PROTEIN L39. [2] SW:RL39_HUMAN ;, mRNA sequence [AI216457]	6.07	8,10	-2,04
ZWINT	Homo sapiens ZW10 interactor (ZWINT), transcript variant 4, mRNA [NM_001005414]	8.94	10,99	-2,05
TMEM48	Homo sapiens transmembrane protein 48 (TMEM48), mRNA [NM_018087]	7.2	9,25	-2,05
HIST1H2BD	Homo sapiens histone 1, H2bd (HIST1H2BD), transcript variant 1, mRNA [NM_021063]	7.99	10,04	-2,05
CEP55	Homo sapiens centrosomal protein 55kDa (CEP55), mRNA [NM_018131]	7.53	9,58	-2,05
BC068044	Homo sapiens cDNA clone IMAGE:6380649, containing frame-shift errors. [BC068044]	8.06	10,11	-2,05
CN391963	CN391963 17000599942841 GRN_PRENEU Homo sapiens cDNA 5', mRNA sequence [CN391963]	4.33	6,38	-2,05
-	chrX:071278427-071278486	9.02	11,07	-2,05
POLQ	Homo sapiens polymerase (DNA directed), theta (POLQ), mRNA [NM_199420]	6.55	8,61	-2,06
BX641010	Homo sapiens mRNA; cDNA DKFZp686G01227 (from clone DKFZp686G01227). [BX641010]	6.49	8,55	-2,06
HIST1H2BH	Homo sapiens histone 1, H2bh (HIST1H2BH), mRNA [NM_003524]	9.93	11,99	-2,06
BX449754	BX449754 Homo sapiens NEUROBLASTOMA Homo sapiens cDNA clone CS0DA007YA13 3-PRIME, mRNA sequence [BX449754]	4.19	6,25	-2,06
LRRC25	Homo sapiens leucine rich repeat containing 25 (LRRC25), mRNA [NM_145256]	7.53	9,60	-2,07
H2BFS	Homo sapiens H2B histone family, member S (H2BFS), mRNA [NM_017445]	7.77	9,84	-2,07
CEP27	Homo sapiens centrosomal protein 27kDa (CEP27), mRNA [NM_018097]	6.52	8,59	-2,07
KIF20A	Homo sapiens kinesin family member 20A (KIF20A), mRNA [NM_005733]	7.46	9,53	-2,08
GCA	Homo sapiens grancalcin, EF-hand calcium binding protein (GCA), mRNA [NM_012198]	9.43	11,51	-2,08
GABPB2	Homo sapiens GA binding protein transcription factor, beta subunit 2 (GABPB2), transcript variant gamma-1, mRNA [NM_002041]	7.39	9,48	-2,08
C1orf135	Homo sapiens chromosome 1 open reading frame 135 (C1orf135), mRNA [NM_024037]	5.51	7,59	-2,08
CDT1	Homo sapiens chromatin licensing and DNA replication factor 1 (CDT1), mRNA [NM_030928]	10.61	12,69	-2,08
BC060806	Homo sapiens cDNA clone MGC:71628 IMAGE:30336414, complete cds. [BC060806]	6.8	8,88	-2,08
-	chrX:108391930-108391989	8.88	10,96	-2,08
THC2302090	chr9:126679841-126679782	6.88	8,96	-2,08
-	chr19:050721774-050721715	6.07	8,14	-2,08
BG001037	BG001037 RC5-GN0132-131100-012-E05 GN0132 Homo sapiens cDNA, mRNA sequence [BG001037]	6.38	8,46	-2,08
ENDOG	Homo sapiens endonuclease G (ENDOG), nuclear gene encoding mitochondrial protein, mRNA [NM_004435]	9.97	12,06	-2,09
ACSS2	Homo sapiens acyl-CoA synthetase short-chain family member 2 (ACSS2), transcript variant 1, mRNA [NM_018677]	6.53	8,62	-2,09
THC2363295	chr5:178974076-178974135	8.89	10,98	-2,09
CCNA2	Homo sapiens cyclin A2 (CCNA2), mRNA [NM_001237]	6.27	8,37	-2,10
CENPM	Homo sapiens centromere protein M (CENPM), transcript variant 2, mRNA [NM_001002876]	6.73	8,83	-2,10
AK128714	Homo sapiens cDNA FLJ46881 fis, clone UTERU3015647, moderately similar to Embigin precursor. [AK128714]	8.18	10,28	-2,10
-	chrX:149832761-149832901	9.09	11,19	-2,10
-	chr17:025067488-025067427	7.29	9,39	-2,10

Supplemental Table 1
Morandi et al

AV739735	AV739735 CB Homo sapiens cDNA clone CBCCMH08 5', mRNA sequence [AV739735]	8.08	10,18	-2,10
UHG	Homo sapiens U22 snoRNA host gene (UHG) on chromosome 11 [NR_003098]	11.61	13,73	-2,11
-	chrX:134886172-134886231	5.54	7,65	-2,11
AL571926	AL571926 Homo sapiens PLACENTA COT 25-NORMALIZED Homo sapiens cDNA clone CS0DI029YJ06 3-PRIME, mRNA sequence [AL571926]	7.06	9,18	-2,11
TLE3	Homo sapiens transducin-like enhancer of split 3 (E(sp1) homolog, Drosophila) (TLE3), mRNA [NM_005078]	7.4	9,51	-2,12
BUB1	Homo sapiens BUB1 budding uninhibited by benzimidazoles 1 homolog (yeast) (BUB1), mRNA [NM_004336]	7.11	9,24	-2,12
CA306742	CA306742 UI-H-FT1-bht-f-03-0-UI.s1 NCI_CGAP_FT1 Homo sapiens cDNA clone UI-H-FT1-bht-f-03-0-UI 3', mRNA sequence [CA306742]	5.01	7,13	-2,12
ABL1	Homo sapiens v-abl Abelson murine leukemia viral oncogene homolog 1 (ABL1), transcript variant b, mRNA [NM_007313]	4.84	6,97	-2,13
HIST1H2BB	Homo sapiens histone 1, H2bb (HIST1H2BB), mRNA [NM_021062]	8.13	10,26	-2,13
VCPIP1	Homo sapiens valosin containing protein (p97)/p47 complex interacting protein 1 (VCPIP1), mRNA [NM_025054]	6.07	8,20	-2,14
RP2	Homo sapiens retinitis pigmentosa 2 (X-linked recessive) (RP2), mRNA [NM_006915]	4.84	6,98	-2,14
ZNFN1A1	Homo sapiens PRO0758 mRNA, complete cds. [AF116605]	6.11	8,25	-2,14
FAM72A	Homo sapiens family with sequence similarity 72, member A (FAM72A), mRNA [NM_207418]	6.56	8,70	-2,14
DLG7	Homo sapiens discs, large homolog 7 (Drosophila) (DLG7), mRNA [NM_014750]	7.79	9,94	-2,14
THC2287287	chr9:68389989-68388033	4.97	7,12	-2,14
HIST1H2AK	Homo sapiens histone 1, H2ak (HIST1H2AK), mRNA [NM_003510]	10.67	12,82	-2,15
DEPDC1	Homo sapiens DEP domain containing 1 (DEPDC1), mRNA [NM_017779]	7.71	9,87	-2,15
CENTG3	Homo sapiens centaurin, gamma 3 (CENTG3), transcript variant 2, mRNA [NM_001042535]	11.52	13,67	-2,15
CEBPE	Homo sapiens CCAAT/enhancer binding protein (C/EBP), epsilon (CEBPE), mRNA [NM_001805]	7.19	9,34	-2,15
THC2318533	chr2:108550140-108550199	6.46	8,61	-2,15
BM547196	BM547196 AGENCOURT_6499364 NIH_MGC_124 Homo sapiens cDNA clone IMAGE:5730270 5', mRNA sequence [BM547196]	10.75	12,90	-2,15
BF803156	BF803156 CM0-CI0139-011100-675-h03 CI0139 Homo sapiens cDNA, mRNA sequence [BF803156]	5.97	8,12	-2,15
SERTAD2	Homo sapiens SERTA domain containing 2 (SERTAD2), mRNA [NM_014755]	4.78	6,95	-2,17
ENST00000328474	Homo sapiens PRO1843 mRNA, complete cds. [AF119854]	7.6	9,76	-2,17
LILRA4	Homo sapiens leukocyte immunoglobulin-like receptor, subfamily A (with TM domain), member 4 (LILRA4), mRNA [NM_012276]	6.09	8,26	-2,17
RP5-1022P6.2	Homo sapiens hypothetical protein KIAA1434 (KIAA1434), mRNA [NM_019593]	9.76	11,94	-2,17
LOC137107	PREDICTED: Homo sapiens similar to ribosomal protein L10a (LOC137107), mRNA [XM_070233]	6.13	8,31	-2,18
THC2455389	O02979 (O02979) ORF2280 gene homolog (Fragment), partial (18%) [THC2455389]	5.47	7,64	-2,18
SPCS3	Homo sapiens signal peptidase complex subunit 3 homolog (S. cerevisiae) (SPCS3), mRNA [NM_021928]	9.93	12,11	-2,18
THC2437039	ALU1_HUMAN (P39188) Alu subfamily J sequence contamination warning entry, partial (7%) [THC2437039]	5.87	8,05	-2,18
IGHM	human full-length cDNA clone CS0DD006YL02 of Neuroblastoma of Homo sapiens (human). [BX161420]	9.61	11,81	-2,20
AURKA	Homo sapiens aurora kinase A (AURKA), transcript variant 1, mRNA [NM_198433]	7.71	9,92	-2,20
-	chr19:047057048-047056751	8.67	10,86	-2,20
BG007597	BG007597 QV4-GN0250-281100-608-g10 GN0250 Homo sapiens cDNA, mRNA sequence [BG007597]	6.65	8,85	-2,20

Supplemental Table 1
Morandi et al

KLK3	Homo sapiens prostate-specific antigen variant 2 mRNA, complete cds, alternatively spliced. [AF335478]	11.68	13,89	-2,21
PLK1	Homo sapiens polo-like kinase 1 (Drosophila) (PLK1), mRNA [NM_005030]	5.87	8,09	-2,21
NCF2	Homo sapiens neutrophil cytosolic factor 2 (65kDa, chronic granulomatous disease, autosomal 2) (NCF2), mRNA [NM_000433]	8.86	11,07	-2,21
OTUD1	Homo sapiens mRNA, clone: TH020D07. [AB188491]	6.23	8,44	-2,21
-	chr4:025356751-025355340	10.35	12,56	-2,21
SYTL1	Homo sapiens synaptotagmin-like 1 (SYTL1), mRNA [NM_032872]	7.12	9,34	-2,22
RAD54L	Homo sapiens RAD54-like (S. cerevisiae) (RAD54L), mRNA [NM_003579]	7.98	10,20	-2,22
KIF11	Homo sapiens kinesin family member 11 (KIF11), mRNA [NM_004523]	5.86	8,08	-2,22
E2F7	Homo sapiens E2F transcription factor 7 (E2F7), mRNA [NM_203394]	6.65	8,88	-2,22
RRM2	Homo sapiens ribonucleotide reductase M2 polypeptide (RRM2), mRNA [NM_001034]	7.24	9,47	-2,23
EAF1	Homo sapiens ELL associated factor 1 (EAF1), mRNA [NM_033083]	4.29	6,52	-2,23
-	chr7:032284770-032284919	9.26	11,49	-2,23
TOB1	Homo sapiens, clone IMAGE:4133978, mRNA. [BC015064]	7.02	9,26	-2,24
SLPI	Homo sapiens secretory leukocyte peptidase inhibitor (SLPI), mRNA [NM_003064]	6.83	9,08	-2,24
PYGL	Homo sapiens phosphorylase, glycogen; liver (Hers disease, glycogen storage disease type VI) (PYGL), mRNA [NM_002863]	8.39	10,63	-2,24
CEACAM4	Homo sapiens carcinoembryonic antigen-related cell adhesion molecule 4 (CEACAM4), mRNA [NM_001817]	5.8	8,04	-2,24
ADM	Homo sapiens adrenomedullin (ADM), mRNA [NM_001124]	11.45	13,69	-2,24
ENST00000333066	chr7:1559689-1559748	10.39	12,63	-2,24
THC2317808	Q8CFL1 (Q8CFL1) Stxbp4 protein (Syntaxin binding protein 4), partial (3%) [THC2317808]	8.27	10,52	-2,25
THC2278097	Q7PWX9 (Q7PWX9) ENSANGP00000004168 (Fragment), partial (73%) [THC2278097]	4.53	6,78	-2,25
STXBP2	Homo sapiens syntaxin binding protein 2 (STXBP2), mRNA [NM_006949]	4.53	6,78	-2,25
FNDC3B	Homo sapiens fibronectin type III domain containing 3B, mRNA (cDNA clone IMAGE:3882800), complete cds. [BC012204]	4.81	7,06	-2,25
FEM1B	Homo sapiens fem-1 homolog b (C. elegans) (FEM1B), mRNA [NM_015322]	6.85	9,10	-2,25
CENPN	Homo sapiens centromere protein N (CENPN), mRNA [NM_018455]	7.35	9,60	-2,25
-	chr6:134756077-134756016	4.22	6,48	-2,25
UTS2R	Homo sapiens urotensin 2 receptor (UTS2R), mRNA [NM_018949]	12.18	14,44	-2,26
PTTG2	Homo sapiens pituitary tumor-transforming 2 (PTTG2), mRNA [NM_006607]	8.73	10,99	-2,26
CR749652	Homo sapiens mRNA; cDNA DKFZp686I07120 (from clone DKFZp686I07120). [CR749652]	8.59	10,85	-2,26
AK092421	Homo sapiens cDNA FLJ35102 fis, clone PLACE6006474, weakly similar to ADHESIVE PLAQUE MATRIX PROTEIN PRECURSOR. [AK092421]	8.6	10,86	-2,26
DKFZP434I0714	PREDICTED: Homo sapiens hypothetical protein DKFZP434I0714 (DKFZP434I0714), mRNA [XM_929673]	9.9	12,17	-2,27
ORM2	Homo sapiens orosomucoid 2 (ORM2), mRNA [NM_000608]	5.16	7,43	-2,27
NEK2	Homo sapiens NIMA (never in mitosis gene a)-related kinase 2 (NEK2), mRNA [NM_002497]	6.96	9,23	-2,27
GTSE1	Homo sapiens G-2 and S-phase expressed 1 (GTSE1), mRNA [NM_016426]	7.46	9,73	-2,27
FCRLM2	Homo sapiens Fc receptor-like and mucin-like 2, mRNA (cDNA clone MGC:71141 IMAGE:3529386), complete cds. [BC067080]	11.8	14,07	-2,27
AA593970	AA593970 nn01c05.s1 NCI_CGAP_Co9 Homo sapiens cDNA clone IMAGE:1076456 3', mRNA sequence [AA593970]	8.59	10,86	-2,27

Supplemental Table 1
Morandi et al

KIAA0179	Homo sapiens KIAA0179 (KIAA0179), mRNA [NM_015056]	6.67	8,95	-2,28
DYNLL2	Homo sapiens dynein, light chain, LC8-type 2 (DYNLL2), mRNA [NM_080677]	10.12	12,40	-2,28
MYO1G	Homo sapiens myosin IG (MYO1G), mRNA [NM_033054]	6.52	8,81	-2,29
EZH2	Homo sapiens enhancer of zeste homolog 2 (Drosophila) (EZH2), transcript variant 1, mRNA [NM_004456]	10.18	12,46	-2,29
THC2342574	chr13:24775303-24775362	9.06	11,35	-2,29
IRX5	Homo sapiens iroquois homeobox protein 5 (IRX5), mRNA [NM_005853]	5.58	7,88	-2,30
IRX3	Homo sapiens iroquois homeobox protein 3 (IRX3), mRNA [NM_024336]	8.9	11,20	-2,30
ELF4	Homo sapiens E74-like factor 4 (ets domain transcription factor) (ELF4), mRNA [NM_001421]	9,00	11,30	-2,30
F5	Homo sapiens coagulation factor V (proaccelerin, labile factor) (F5), mRNA [NM_000130]	6.13	8,43	-2,30
HIST1H2BC	Homo sapiens histone 1, H2bc (HIST1H2BC), mRNA [NM_003526]	6.32	8,63	-2,31
FAM122B	Homo sapiens family with sequence similarity 122B (FAM122B), mRNA [NM_145284]	6,00	8,31	-2,31
-	chr14:072673264-072673205	11.88	14,19	-2,31
AI381562	AI381562 te76g06.x1 Soares_NFL_T_GBC_S1 Homo sapiens cDNA clone IMAGE:2092666 3', mRNA sequence [AI381562]	4.59	6,90	-2,31
SKP2	Homo sapiens S-phase kinase-associated protein 2 (p45) (SKP2), transcript variant 2, mRNA [NM_032637]	6.25	8,57	-2,32
MELK	Homo sapiens maternal embryonic leucine zipper kinase (MELK), mRNA [NM_014791]	6.74	9,05	-2,32
C6orf173	Homo sapiens chromosome 6 open reading frame 173 (C6orf173), mRNA [NM_001012507]	8.01	10,33	-2,32
PHF19	Homo sapiens PHD finger protein 19 (PHF19), transcript variant 2, mRNA [NM_001009936]	9.28	11,61	-2,33
HIST1H1D	Homo sapiens histone 1, H1d (HIST1H1D), mRNA [NM_005320]	8.73	11,06	-2,33
AK092681	Homo sapiens cDNA FLJ35362 fis, clone SKMUS2000330. [AK092681]	4.89	7,22	-2,33
NYREN18	full-length cDNA clone CS0DJ007YF12 of T cells (Jurkat cell line) Cot 10-normalized of Homo sapiens (human). [CR606629]	5.32	7,65	-2,33
TPX2	Homo sapiens TPX2, microtubule-associated, homolog (Xenopus laevis) (TPX2), mRNA [NM_012112]	8.47	10,81	-2,34
RAB11FIP1	Homo sapiens RAB11 family interacting protein 1 (class I) (RAB11FIP1), transcript variant 2, mRNA [NM_001002233]	7.21	9,56	-2,34
C12orf48	Homo sapiens chromosome 12 open reading frame 48 (C12orf48), mRNA [NM_017915]	7.28	9,62	-2,34
SERPINA1	Homo sapiens serpin peptidase inhibitor, clade A (alpha-1 antiproteinase, antitrypsin), member 1 (SERPINA1), transcript variant 2, mRNA [NM_001002236]	7.41	9,77	-2,35
CDC20	Homo sapiens CDC20 cell division cycle 20 homolog (S. cerevisiae) (CDC20), mRNA [NM_001255]	7.52	9,87	-2,35
ASF1B	Homo sapiens ASF1 anti-silencing function 1 homolog B (S. cerevisiae) (ASF1B), mRNA [NM_018154]	6.97	9,32	-2,35
SLC11A1	Homo sapiens solute carrier family 11 (proton-coupled divalent metal ion transporters), member 1 (SLC11A1), mRNA [NM_000578]	5.27	7,63	-2,36
CEBPA	Homo sapiens CCAAT/enhancer binding protein (C/EBP), alpha (CEBPA), mRNA [NM_004364]	11.3	13,66	-2,36
-	chr19:038524297-038524356	6.7	9,07	-2,37
-	chr1:241351629-241351688	6.12	8,48	-2,37
LOC440731	PREDICTED: Homo sapiens hypothetical LOC440731, transcript variant 3 (LOC440731), mRNA [XM_933697]	5.25	7,63	-2,38
MIDN	Homo sapiens midnolin (MIDN), mRNA [NM_177401]	4.16	6,54	-2,38
CD69	Homo sapiens CD69 molecule (CD69), mRNA [NM_001781]	8.21	10,60	-2,38
THC2443571	AF032119 hCASK {Homo sapiens;} , partial (13%) [THC2443571]	5.9	8,28	-2,38
ARHGAP15	Homo sapiens Rho GTPase activating protein 15 (ARHGAP15), mRNA [NM_018460]	5.22	7,61	-2,39

AL832717	Homo sapiens mRNA; cDNA DKFZp313B039 (from clone DKFZp313B039). [AL832717]	7.01	9,40	-2,39
C1orf55	Homo sapiens chromosome 1 open reading frame 55 (C1orf55), mRNA [NM_152608]	6.48	8,87	-2,40
CASC5	Homo sapiens cancer susceptibility candidate 5 (CASC5), transcript variant 1, mRNA [NM_170589]	6.52	8,91	-2,40
THC2314346	Q6P4C2 (Q6P4C2) RBBP6 protein (Fragment), partial (31%) [THC2314346]	8.01	10,42	-2,41
ZNF206	Homo sapiens zinc finger protein 206 (ZNF206), mRNA [NM_032805]	9.91	12,33	-2,42
SPI1	Homo sapiens spleen focus forming virus (SFFV) proviral integration oncogene spi1 (SPI1), mRNA [NM_003120]	4.93	7,35	-2,42
ITGAL	Homo sapiens integrin, alpha L (antigen CD11A (p180), lymphocyte function-associated antigen 1; alpha polypeptide) (ITGAL), mRNA [NM_002209]	5.92	8,34	-2,42
THC2438327	chr1:35403520-35403579	10.41	12,83	-2,42
E2F1	Homo sapiens E2F transcription factor 1 (E2F1), mRNA [NM_005225]	8.19	10,62	-2,43
PKMYT1	Homo sapiens protein kinase, membrane associated tyrosine/threonine 1 (PKMYT1), transcript variant 2, mRNA [NM_182687]	10.12	12,55	-2,44
KLHL7	Homo sapiens kelch-like 7 (Drosophila), mRNA (cDNA clone IMAGE:3899090), complete cds. [BC009555]	7.06	9,50	-2,44
AV722457	AV722457 HTB Homo sapiens cDNA clone HTBAMH03 5', mRNA sequence [AV722457]	4.85	7,32	-2,47
HIST1H1E	Homo sapiens histone 1, H1e (HIST1H1E), mRNA [NM_005321]	8.42	10,90	-2,48
WNK1	Homo sapiens WNK lysine deficient protein kinase 1, mRNA (cDNA clone IMAGE:4650586), complete cds. [BC094862]	4.56	7,05	-2,49
CDC25C	Homo sapiens cell division cycle 25C (CDC25C), transcript variant 1, mRNA [NM_001790]	6.08	8,57	-2,49
BCNP1	Homo sapiens B-cell novel protein 1 (BCNP1), mRNA [NM_173544]	6.59	9,08	-2,49
BE138567	xr77d10.x2 NCI_CGAP_Ov26 Homo sapiens cDNA clone IMAGE:2766163 3', mRNA sequence [BE138567]	6.32	8,82	-2,50
SNORD22	Homo sapiens small nucleolar RNA, C/D box 22 (SNORD22) on chromosome 11 [NR_000008]	6.48	8,98	-2,50
LOC158830	Homo sapiens similar to Ab2-183 (LOC158830), mRNA [NM_001025265]	6.26	8,77	-2,51
HIST1H2BG	Homo sapiens histone 1, H2bg (HIST1H2BG), mRNA [NM_003518]	6.83	9,34	-2,51
ARHGAP11A	Homo sapiens Rho GTPase activating protein 11A (ARHGAP11A), transcript variant 1, mRNA [NM_014783]	5.7	8,21	-2,52
NPAS3	Homo sapiens neuronal PAS domain protein 3 (NPAS3), transcript variant 2, mRNA [NM_173159]	11.73	14,25	-2,52
GINS2	Homo sapiens GINS complex subunit 2 (Psf2 homolog) (GINS2), mRNA [NM_016095]	8.86	11,40	-2,54
SOD2	Homo sapiens superoxide dismutase 2, mitochondrial (SOD2), nuclear gene encoding mitochondrial protein, transcript variant 1, mRNA [NM_000636]	10.1	12,64	-2,55
BF089603	BF089603 CM2-HT0946-140900-364-f11 HT0946 Homo sapiens cDNA, mRNA sequence [BF089603]	6.07	8,62	-2,55
BST1	Homo sapiens bone marrow stromal cell antigen 1 (BST1), mRNA [NM_004334]	7.56	10,12	-2,56
BX409884	BX409884 Homo sapiens NEUROBLASTOMA Homo sapiens cDNA clone CL0BB005ZC10 5-PRIME, mRNA sequence [BX409884]	5.27	7,83	-2,56
HIST2H2AB	Homo sapiens histone 2, H2ab (HIST2H2AB), mRNA [NM_175065]	8.87	11,43	-2,57
ESPL1	Homo sapiens extra spindle poles like 1 (S. cerevisiae) (ESPL1), mRNA [NM_012291]	8.39	10,96	-2,57
AW797858	AW797858 CM0-UM0042-130300-280-d11 UM0042 Homo sapiens cDNA, mRNA sequence [AW797858]	6.21	8,78	-2,57
SPAG5	Homo sapiens sperm associated antigen 5 (SPAG5), mRNA [NM_006461]	10.15	12,74	-2,58
-	chr5:151164351-151164292	7.36	9,93	-2,58

HMMR	Homo sapiens hyaluronan-mediated motility receptor (RHAMM) (HMMR), transcript variant 1, mRNA [NM_012484]	8.02	10,61	-2,59
FLJ40542	Homo sapiens cDNA FLJ40542 fis, clone THYMU2000057. [AK097861]	4.47	7,06	-2,59
SPAG9	Homo sapiens sperm associated antigen 9, mRNA (cDNA clone IMAGE:3351321), complete cds. [BC007524]	6.17	8,77	-2,60
NUSAP1	Homo sapiens nucleolar and spindle associated protein 1 (NUSAP1), transcript variant 1, mRNA [NM_016359]	6.66	9,26	-2,60
BRRN1	Homo sapiens barren homolog 1 (Drosophila) (BRRN1), mRNA [NM_015341]	7.71	10,31	-2,60
HIST1H2BJ	Homo sapiens histone 1, H2bj (HIST1H2BJ), mRNA [NM_021058]	6.83	9,44	-2,61
FANCD2	Homo sapiens Fanconi anemia, complementation group D2 (FANCD2), transcript variant 2, mRNA [NM_001018115]	7.36	9,97	-2,61
CDCA5	Homo sapiens cell division cycle associated 5 (CDCA5), mRNA [NM_080668]	9.37	11,98	-2,61
CKS2	Homo sapiens CDC28 protein kinase regulatory subunit 2 (CKS2), mRNA [NM_001827]	9.56	12,17	-2,61
ALOX5	Homo sapiens arachidonate 5-lipoxygenase (ALOX5), mRNA [NM_000698]	8.77	11,38	-2,61
AL833005	Homo sapiens mRNA; cDNA DKFZp666D074 (from clone DKFZp666D074) [AL833005]	9.29	11,91	-2,62
FGR	Homo sapiens Gardner-Rasheed feline sarcoma viral (v-fgr) oncogene homolog (FGR), mRNA [NM_005248]	5.42	8,04	-2,62
ENST00000299289	chr6:31802393-31802452	5.48	8,10	-2,62
PRTN3	Homo sapiens proteinase 3 (serine proteinase, neutrophil, Wegener granulomatosis autoantigen) (PRTN3), mRNA [NM_002777]	5.01	7,63	-2,63
AV749257	AV749257 AV749257 NPC Homo sapiens cDNA clone NPCCTB10 5', mRNA sequence [AV749257]	4.96	7,59	-2,63
PRAM1	Homo sapiens PML-RARA regulated adaptor molecule 1 (PRAM1), mRNA [NM_032152]	8.19	10,83	-2,64
ICAM3	Homo sapiens intercellular adhesion molecule 3 (ICAM3), mRNA [NM_002162]	9.84	12,48	-2,64
TMEM154	Homo sapiens transmembrane protein 154 (TMEM154), mRNA [NM_152680]	5.05	7,71	-2,66
BUB1B	Homo sapiens BUB1 budding uninhibited by benzimidazoles 1 homolog beta (yeast) (BUB1B), mRNA [NM_001211]	7.27	9,93	-2,66
BX350256	BX350256 BX350256 Homo sapiens PLACENTA COT 25-NORMALIZED Homo sapiens cDNA clone CS0DI081YM18 3-PRIME, mRNA sequence [BX350256]	5.48	8,14	-2,66
THC2375394	chr21:42304489-42304430	4.21	6,89	-2,68
AI571129	AI571129 tn85e01.x1 NCI_CGAP_Ut2 Homo sapiens cDNA clone IMAGE:2176344 3', mRNA sequence [AI571129]	5.76	8,44	-2,68
AL833005	Homo sapiens mRNA; cDNA DKFZp666D074 (from clone DKFZp666D074) [AL833005]	8.97	11,66	-2,69
KIF4A	Homo sapiens kinesin family member 4A (KIF4A), mRNA [NM_012310]	6.6	9,30	-2,69
KIF2C	Homo sapiens kinesin family member 2C (KIF2C), mRNA [NM_006845]	9.23	11,92	-2,69
POU2AF1	Homo sapiens POU domain, class 2, associating factor 1 (POU2AF1), mRNA [NM_006235]	7.16	9,86	-2,70
OIP5	Homo sapiens Opa interacting protein 5 (OIP5), mRNA [NM_007280]	8.04	10,74	-2,70
CHEK1	Homo sapiens CHK1 checkpoint homolog (S. pombe) (CHEK1), mRNA [NM_001274]	7.07	9,80	-2,72
HK2	Homo sapiens hexokinase 2 (HK2), mRNA [NM_000189]	5.14	7,87	-2,73
KIF15	Homo sapiens kinesin family member 15 (KIF15), mRNA [NM_020242]	7.23	9,97	-2,74
CDCA1	Homo sapiens cell division cycle associated 1 (CDCA1), transcript variant 1, mRNA [NM_145697]	8.78	11,52	-2,74
ANXA8	Homo sapiens annexin A8 (ANXA8), mRNA [NM_001630]	5.09	7,83	-2,74
PPIG	Homo sapiens peptidylprolyl isomerase G (cyclophilin G), mRNA (cDNA clone IMAGE:3461499), complete cds. [BC001555]	5.19	7,95	-2,75
-	chr1:162723477-162723536	5.46	8,21	-2,75

Supplemental Table 1
Morandi et al

PLAUR	Homo sapiens plasminogen activator, urokinase receptor (PLAUR), transcript variant 3, mRNA [NM_001005377]	7.58	10,34	-2,76
IL1B	Homo sapiens interleukin 1, beta (IL1B), mRNA [NM_000576]	7.26	10,02	-2,76
TAGAP	Homo sapiens T-cell activation GTPase activating protein (TAGAP), transcript variant 2, mRNA [NM_054114]	6.34	9,11	-2,77
HIST1H1C	Homo sapiens histone 1, H1c (HIST1H1C), mRNA [NM_005319]	10.87	13,64	-2,77
HCAP-G	Homo sapiens chromosome condensation protein G (HCAP-G), mRNA [NM_022346]	8.74	11,52	-2,78
PLEKHK1	Homo sapiens pleckstrin homology domain containing, family K member 1 (PLEKHK1), mRNA [NM_145307]	5.71	8,49	-2,79
MLC1	Homo sapiens megalencephalic leukoencephalopathy with subcortical cysts 1 (MLC1), transcript variant 1, mRNA [NM_015166]	5.96	8,75	-2,79
HIST2H2AA3	Homo sapiens histone 2, H2aa3 (HIST2H2AA3), mRNA [NM_003516]	11.94	14,73	-2,79
GSG2	Homo sapiens cDNA FLJ32129 fis, clone PEBLM2000213, weakly similar to Mus musculus genes for integrin aM290, hapsin. [AK056691]	7.16	9,95	-2,79
FLJ22662	Homo sapiens hypothetical protein FLJ22662 (FLJ22662), mRNA [NM_024829]	8.62	11,43	-2,81
ZC3H12A	Homo sapiens zinc finger CCCH-type containing 12A (ZC3H12A), mRNA [NM_025079]	7.46	10,29	-2,83
TROAP	Homo sapiens trophinin associated protein (tastin) (TROAP), mRNA [NM_005480]	9.3	12,14	-2,83
HIST1H4A	Homo sapiens histone 1, H4a (HIST1H4A), mRNA [NM_003538]	5.23	8,08	-2,85
CDA	Homo sapiens cytidine deaminase (CDA), mRNA [NM_001785]	5.98	8,84	-2,85
AF034187	Homo sapiens clone 2.2H12 Ndr Ser/Thr kinase-like protein mRNA, partial cds. [AF034187]	6.3	9,15	-2,85
THC2414638	chr3:13430459-13430400	5.62	8,46	-2,85
PTTG1	Homo sapiens pituitary tumor-transforming 1 (PTTG1), mRNA [NM_004219]	11.45	14,32	-2,87
CD37	Homo sapiens CD37 molecule (CD37), transcript variant 1, mRNA [NM_001774]	7.31	10,18	-2,87
ENST00000251375	Homo sapiens leucocyte immunoglobulin-like receptor-6b (LIR-6) mRNA, complete cds. [AF025529]	5.1	7,98	-2,88
-	chr17:037971521-037971466	4.87	7,76	-2,88
PTPRE	Homo sapiens protein tyrosine phosphatase, receptor type, E (PTPRE), transcript variant 1, mRNA [NM_006504]	7.63	10,53	-2,90
PLAC8	Homo sapiens placenta-specific 8 (PLAC8), mRNA [NM_016619]	6.03	8,94	-2,90
THC2400121	PBEF_HUMAN (P43490) Pre-B cell enhancing factor precursor, partial (16%) [THC2400121]	5.49	8,42	-2,92
TYMS	Homo sapiens thymidylate synthetase (TYMS), mRNA [NM_001071]	10.03	12,95	-2,92
CCNB1	Homo sapiens cyclin B1 (CCNB1), mRNA [NM_031966]	8.65	11,58	-2,93
BM930248	BM930248 UI-E-EJ1-ajh-b-03-0-UI.r1 UI-E-EJ1 Homo sapiens cDNA clone UI-E-EJ1-ajh-b-03-0-UI 5', mRNA sequence [BM930248]	4.74	7,67	-2,93
ZBTB24	Homo sapiens zinc finger and BTB domain containing 24 (ZBTB24), mRNA [NM_014797]	5.96	8,90	-2,94
MAD2L1	Homo sapiens MAD2 mitotic arrest deficient-like 1 (yeast) (MAD2L1), mRNA [NM_002358]	8.43	11,37	-2,94
ASPM	Homo sapiens asp (abnormal spindle)-like, microcephaly associated (Drosophila) (ASPM), mRNA [NM_018136]	7.69	10,65	-2,96
LGALS12	Homo sapiens lectin, galactoside-binding, soluble, 12 (galectin 12) (LGALS12), mRNA [NM_033101]	4.89	7,86	-2,97
SLC30A1	Homo sapiens solute carrier family 30 (zinc transporter), member 1 (SLC30A1), mRNA [NM_021194]	6.56	9,54	-2,99
HIST1H2AD	Homo sapiens histone 1, H2ad (HIST1H2AD), mRNA [NM_021065]	10.32	13,33	-3,00
FCRL5	Homo sapiens Fc receptor-like 5 (FCRL5), mRNA [NM_031281]	5.58	8,59	-3,00
ALOX5AP	Homo sapiens arachidonate 5-lipoxygenase-activating protein (ALOX5AP), mRNA [NM_001629]	6.88	9,88	-3,00

Supplemental Table 1
Morandi et al

GPR84	Homo sapiens G protein-coupled receptor 84 (GPR84), mRNA [NM_020370]	4.56	7,57	-3,01
CENPF	Homo sapiens centromere protein F, 350/400ka (mitosin) (CENPF), mRNA [NM_016343]	10.14	13,16	-3,01
CD19	Homo sapiens CD19 molecule (CD19), mRNA [NM_001770]	7.62	10,64	-3,02
CDCA2	Homo sapiens cell division cycle associated 2 (CDCA2), mRNA [NM_152562]	7.53	10,58	-3,04
ENST00000324677	Homo sapiens slingshot homolog 2 (Drosophila), mRNA (cDNA clone IMAGE:4101583), complete cds. [BC011636]	5.57	8,64	-3,06
C11orf21	Homo sapiens C11orf21 mRNA, complete cds. [AB029488]	6.77	9,87	-3,09
FAM64A	Homo sapiens family with sequence similarity 64, member A (FAM64A), mRNA [NM_019013]	9.08	12,18	-3,10
ANPEP	Homo sapiens alanyl (membrane) aminopeptidase (aminopeptidase N, aminopeptidase M, microsomal aminopeptidase, CD13, p150) (ANPEP), mRNA [NM_001150]	6.36	9,46	-3,10
MLF1IP	Homo sapiens MLF1 interacting protein (MLF1IP), mRNA [NM_024629]	8.19	11,30	-3,11
-	chr4:015359368-015359309	6.3	9,42	-3,12
TNNT1	Homo sapiens troponin T type 1 (skeletal, slow), mRNA (cDNA clone MGC:104241 IMAGE:4247379), complete cds. [BC107798]	4.94	8,09	-3,15
P2RY2	Homo sapiens purinergic receptor P2Y, G-protein coupled, 2 (P2RY2), transcript variant 1, mRNA [NM_176072]	5.43	8,58	-3,15
SLC16A3	Homo sapiens pp10472 mRNA, complete cds. [AF318321]	5.71	8,86	-3,15
BM979049	BM979049 UI-CF-DU1-adl-i-11-0-UI.s1 UI-CF-DU1 Homo sapiens cDNA clone UI-CF-DU1-adl-i-11-0-UI 3', mRNA sequence [BM979049]	5.68	8,83	-3,15
PRG1	Homo sapiens proteoglycan 1, secretory granule (PRG1), mRNA [NM_002727]	10.76	13,94	-3,18
CDC45L	Homo sapiens CDC45 cell division cycle 45-like (S. cerevisiae) (CDC45L), mRNA [NM_003504]	9.35	12,54	-3,19
KIAA0101	Homo sapiens KIAA0101 (KIAA0101), transcript variant 1, mRNA [NM_014736]	8.86	12,07	-3,21
BLK	Homo sapiens B lymphoid tyrosine kinase (BLK), mRNA [NM_001715]	5.53	8,74	-3,21
FPR1	Homo sapiens formyl peptide receptor 1 (FPR1), mRNA [NM_002029]	7.82	11,05	-3,23
NCF1	Homo sapiens neutrophil cytosolic factor 1, (chronic granulomatous disease, autosomal 1) (NCF1), transcript variant 1, mRNA [NM_000265]	7.54	10,78	-3,24
IL18RAP	Homo sapiens interleukin 18 receptor accessory protein (IL18RAP), mRNA [NM_003853]	6.68	9,92	-3,24
CDC6	Homo sapiens CDC6 cell division cycle 6 homolog (S. cerevisiae) (CDC6), mRNA [NM_001254]	7.51	10,75	-3,24
KCNJ15	Homo sapiens potassium inwardly-rectifying channel, subfamily J, member 15 (KCNJ15), transcript variant 1, mRNA [NM_170736]	4.05	7,33	-3,28
MS4A1	Homo sapiens membrane-spanning 4-domains, subfamily A, member 1 (MS4A1), transcript variant 1, mRNA [NM_152866]	4.99	8,27	-3,28
CST7	Homo sapiens cystatin F (leukocystatin) (CST7), mRNA [NM_003650]	4.45	7,75	-3,30
CDCA8	Homo sapiens cell division cycle associated 8 (CDCA8), mRNA [NM_018101]	9.22	12,52	-3,30
TREM1	Homo sapiens triggering receptor expressed on myeloid cells 1 (TREM1), mRNA [NM_018643]	4.39	7,71	-3,32
AV756170	AV756170 BM Homo sapiens cDNA clone BMFBGA09 5', mRNA sequence [AV756170]	5.94	9,26	-3,32
UBE2C	Homo sapiens ubiquitin-conjugating enzyme E2C (UBE2C), transcript variant 6, mRNA [NM_181803]	10.14	13,48	-3,33
KRT23	Homo sapiens keratin 23 (histone deacetylase inducible) (KRT23), mRNA [NM_015515]	4.49	7,82	-3,33
STK17B	Homo sapiens serine/threonine kinase 17b (apoptosis-inducing) (STK17B), mRNA [NM_004226]	7.16	10,51	-3,34
NFE2	Homo sapiens nuclear factor (erythroid-derived 2), 45kDa (NFE2), mRNA [NM_006163]	7.08	10,45	-3,37
SIGLEC5	Homo sapiens sialic acid binding Ig-like lectin 5 (SIGLEC5), mRNA [NM_003830]	6.29	9,68	-3,39

Supplemental Table 1
Morandi et al

DKFZP586P0123	Homo sapiens hypothetical protein (DKFZP586P0123), mRNA [NM_015531]	3.99	7,39	-3,40
KIF23	Homo sapiens kinesin family member 23 (KIF23), transcript variant 1, mRNA [NM_138555]	7.67	11,09	-3,42
HSH2D	Homo sapiens hematopoietic SH2 domain containing (HSH2D), mRNA [NM_032855]	6.53	9,96	-3,43
AY358224	Homo sapiens clone DNA213069 RTFV9368 (UNQ9368) mRNA, complete cds. [AY358224]	4.52	7,96	-3,44
CCNB2	Homo sapiens cyclin B2 (CCNB2), mRNA [NM_004701]	9.11	12,59	-3,48
MBD2	Homo sapiens methyl-CpG binding domain protein 2 (MBD2), transcript variant testis-specific, mRNA [NM_015832]	4.9	8,43	-3,53
PPBP	Homo sapiens pro-platelet basic protein (chemokine (C-X-C motif) ligand 7) (PPBP), mRNA [NM_002704]	5.28	8,88	-3,59
BC007606	Homo sapiens cDNA clone IMAGE:3351130, complete cds. [BC007606]	7.6	11,20	-3,60
THC2266672	1305349A cystic fibrosis antigen. {Homo sapiens;} , complete [THC2266672]	3.46	7,11	-3,65
LILRA2	Homo sapiens leukocyte immunoglobulin-like receptor, subfamily A (with TM domain), member 2 (LILRA2), mRNA [NM_006866]	4.4	8,10	-3,70
BIRC5	Homo sapiens baculoviral IAP repeat-containing 5 (survivin) (BIRC5), transcript variant 3, mRNA [NM_001012271]	10.76	14,51	-3,75
VNN2	Homo sapiens vanin 2 (VNN2), transcript variant 1, mRNA [NM_004665]	6.96	10,74	-3,78
TAGAP	Homo sapiens T-cell activation GTPase activating protein (TAGAP), transcript variant 3, mRNA [NM_138810]	4.83	8,61	-3,78
GPR109B	Homo sapiens G protein-coupled receptor 109B (GPR109B), mRNA [NM_006018]	5.17	9,02	-3,85
FCAR	Homo sapiens Fc fragment of IgA, receptor for (FCAR), transcript variant 1, mRNA [NM_002000]	4.08	7,95	-3,87
CSTA	Homo sapiens cystatin A (stefin A) (CSTA), mRNA [NM_005213]	7.67	11,56	-3,89
APOBEC3B	Homo sapiens apolipoprotein B mRNA editing enzyme, catalytic polypeptide-like 3B (APOBEC3B), mRNA [NM_004900]	5.26	9,18	-3,92
THC2271582	ALU5_HUMAN (P39192) Alu subfamily SC sequence contamination warning entry, partial (9%) [THC2271582]	8.46	12,39	-3,93
PRG3	Homo sapiens proteoglycan 3 (PRG3), mRNA [NM_006093]	4.93	8,94	-4,00
FCN1	Homo sapiens ficolin (collagen/fibrinogen domain containing) 1 (FCN1), mRNA [NM_002003]	6.24	10,35	-4,12
FOLR3	Homo sapiens folate receptor 3 (gamma) (FOLR3), mRNA [NM_000804]	3.72	7,86	-4,14
LYZ	Homo sapiens lysozyme (renal amyloidosis) (LYZ), mRNA [NM_000239]	7.63	11,82	-4,19
FCN2	Homo sapiens ficolin (collagen/fibrinogen domain containing lectin) 2 (hucolin) (FCN2), transcript variant SV0, mRNA [NM_004108]	4.37	8,62	-4,25
LILRA3	Homo sapiens leukocyte immunoglobulin-like receptor, subfamily A (without TM domain), member 3 (LILRA3), mRNA [NM_006865]	5.03	9,32	-4,29
CD177	full-length cDNA clone CS0DI015YO17 of Placenta Cot 25-normalized of Homo sapiens (human). [CR592446]	4.63	8,94	-4,31
RETN	Homo sapiens resistin (RETN), mRNA [NM_020415]	5.32	9,74	-4,43
MNDA	Homo sapiens myeloid cell nuclear differentiation antigen (MNDA), mRNA [NM_002432]	7.37	11,86	-4,49
MCEMP1	Homo sapiens mast cell-expressed membrane protein 1 (MCEMP1), mRNA [NM_174918]	4.62	9,16	-4,54
RGR	Homo sapiens Ral-GDS related protein Rgr (Rgr), mRNA [NM_153615]	7.18	11,83	-4,65
CTSG	Homo sapiens cathepsin G (CTSG), mRNA [NM_001911]	6.66	11,56	-4,90
ENST00000316772	Homo sapiens HSPC102 mRNA, partial cds. [AF161365]	3.59	8,81	-5,22
THC2283518	ALU5_HUMAN (P39192) Alu subfamily SC sequence contamination warning entry, partial (8%) [THC2283518]	4.13	9,45	-5,32
ARG1	Homo sapiens arginase, liver (ARG1), mRNA [NM_000045]	4.37	9,74	-5,37
LTF	Homo sapiens lactotransferrin (LTF), mRNA [NM_002343]	5.32	10,74	-5,41

Supplemental Table 1
Morandi et al

ENST00000258817	GB|X55989.1|CAA39461.1 eosinophil cationic-related protein [NP103298]	3.65	9,14	-5,49
MPO	Homo sapiens myeloperoxidase (MPO), nuclear gene encoding mitochondrial protein, mRNA [NM_000250]	5.21	10,75	-5,54
AQP9	Homo sapiens aquaporin 9 (AQP9), mRNA [NM_020980]	4.64	10,20	-5,56
S100P	Homo sapiens S100 calcium binding protein P (S100P), mRNA [NM_005980]	6.68	12,46	-5,78
CLEC4D	Homo sapiens C-type lectin domain family 4, member D (CLEC4D), mRNA [NM_080387]	3.6	9,47	-5,87
CAMP	Homo sapiens cathelicidin antimicrobial peptide (CAMP), mRNA [NM_004345]	5.3	11,52	-6,21
S100A8	Homo sapiens S100 calcium binding protein A8 (calgranulin A) (S100A8), mRNA [NM_002964]	8.55	15,09	-6,55
BI026064	BI026064 CM0-MT0374-060201-774-h11 MT0374 Homo sapiens cDNA, mRNA sequence [BI026064]	4.09	10,81	-6,72
S100A9	Homo sapiens S100 calcium binding protein A9 (calgranulin B) (S100A9), mRNA [NM_002965]	5.66	12,89	-7,23

Supplemental Table 1
Morandi et al

GeneName	Description	MEAN expression stage 4 NBs	MEAN expression GD2+ cells	Difference expression mean (stage 4 NBs - GD2+ cells)
CLEC14A	Homo sapiens C-type lectin domain family 14, member A (CLEC14A), mRNA [NM_175060]	10,917	3,346	7,57
MYH11	Homo sapiens myosin, heavy chain 11, smooth muscle (MYH11), transcript variant SM2B, mRNA [NM_001040113]	10,268	3,003	7,27
CCL19	Homo sapiens chemokine (C-C motif) ligand 19 (CCL19), mRNA [NM_006274]	12,561	5,349	7,21
CD34	Homo sapiens CD34 molecule (CD34), transcript variant 2, mRNA [NM_001773]	10,135	3,551	6,58
VWF	Homo sapiens von Willebrand factor (VWF), mRNA [NM_000552]	13,577	7,021	6,56
COL3A1	Homo sapiens collagen, type III, alpha 1 (Ehlers-Danlos syndrome type IV, autosomal dominant) (COL3A1), mRNA [NM_000090]	13,557	7,329	6,23
SOD3	Homo sapiens superoxide dismutase 3, extracellular (SOD3), mRNA [NM_003102]	12,054	5,985	6,07
LOC338328	Homo sapiens high density lipoprotein-binding protein (LOC338328), mRNA [NM_178172]	10,449	4,439	6,01
HIGD1B	Homo sapiens HIG1 domain family, member 1B (HIGD1B), mRNA [NM_016438]	8,663	2,715	5,95
ROBO4	Homo sapiens roundabout homolog 4, magic roundabout (Drosophila) (ROBO4), mRNA [NM_019055]	10,208	4,382	5,83
MGP	Homo sapiens matrix Gla protein (MGP), mRNA [NM_000900]	12,536	6,84	5,70
NR2F2	Homo sapiens nuclear receptor subfamily 2, group F, member 2 (NR2F2), mRNA [NM_021005]	12,198	6,541	5,66
GJA4	Homo sapiens gap junction protein, alpha 4, 37kDa (connexin 37) (GJA4), mRNA [NM_002060]	9,743	4,207	5,54
THBS2	Human thrombospondin 2 (THBS2) mRNA, complete cds. [L12350]	12,457	7,005	5,45
SMOC2	Homo sapiens SPARC related modular calcium binding 2 (SMOC2), mRNA [NM_022138]	12,339	6,906	5,43
H19	Homo sapiens H19, imprinted maternally expressed untranslated mRNA (H19) on chromosome 11 [NR_002196]	11,48	6,05	5,43
HEYL	Homo sapiens hairy/enhancer-of-split related with YRPW motif-like (HEYL), mRNA [NM_014571]	12,06	6,664	5,40
AQP1	Homo sapiens aquaporin 1 (Colton blood group) (AQP1), mRNA [NM_198098]	13,07	7,76	5,31
ATP1A2	Homo sapiens ATPase, Na+/K+ transporting, alpha 2 (+) polypeptide (ATP1A2), mRNA [NM_000702]	8,332	3,136	5,20
ADH1C	Homo sapiens alcohol dehydrogenase 1C (class I), gamma polypeptide (ADH1C), mRNA [NM_000669]	7,994	2,872	5,12
CYR61	Homo sapiens cysteine-rich, angiogenic inducer, 61 (CYR61), mRNA [NM_001554]	12,035	6,922	5,11
IL33	Homo sapiens interleukin 33 (IL33), mRNA [NM_033439]	8,614	3,602	5,01
NOSTRIN	Homo sapiens nitric oxide synthase trafficker (NOSTRIN), transcript variant 1, mRNA [NM_052946]	9,63	4,675	4,96
PLVAP	Homo sapiens plasmalemma vesicle associated protein (PLVAP), mRNA [NM_031310]	9,28	4,351	4,93
CCDC102B	Homo sapiens coiled-coil domain containing 102B (CCDC102B), mRNA [NM_024781]	9,091	4,184	4,91
TNXB	Homo sapiens tenascin XB (TNXB), transcript variant XB-S, mRNA [NM_032470]	12,399	7,533	4,87
PCDH12	Homo sapiens protocadherin 12 (PCDH12), mRNA [NM_016580]	8,071	3,219	4,85
VWA1	Homo sapiens von Willebrand factor A domain containing 1 (VWA1), transcript variant 1, mRNA [NM_022834]	12,555	7,771	4,78
PLAT	Homo sapiens plasminogen activator, tissue (PLAT), transcript variant 1, mRNA [NM_000930]	11,157	6,451	4,71
PTRF	Homo sapiens polymerase I and transcript release factor (PTRF), mRNA [NM_012232]	14,73	10,035	4,70
C7	Homo sapiens complement component 7 (C7), mRNA [NM_000587]	9,715	5,035	4,68
UBD	Homo sapiens ubiquitin D (UBD), mRNA [NM_006398]	10,603	5,959	4,64

Supplemental Table 1
Morandi et al

ACTA2	Homo sapiens actin, alpha 2, smooth muscle, aorta (ACTA2), mRNA [NM_001613]	12,802	8,173	4,63
COL4A1	Homo sapiens collagen, type IV, alpha 1 (COL4A1), mRNA [NM_001845]	11,858	7,237	4,62
DSCR1L1	Homo sapiens Down syndrome critical region gene 1-like 1 (DSCR1L1), mRNA [NM_005822]	11,311	6,695	4,62
OLFML2B	Homo sapiens olfactomedin-like 2B (OLFML2B), mRNA [NM_015441]	9,571	4,998	4,57
CXorf36	Homo sapiens chromosome X open reading frame 36 (CXorf36), mRNA [NM_024689]	8,213	3,658	4,56
COL1A2	Homo sapiens collagen, type I, alpha 2 (COL1A2), mRNA [NM_000089]	13,559	9,045	4,51
TMEM16A	Homo sapiens transmembrane protein 16A (TMEM16A), mRNA [NM_018043]	9,093	4,597	4,50
RARRES2	Homo sapiens retinoic acid receptor responder (tazarotene induced) 2 (RARRES2), mRNA [NM_002889]	11,133	6,695	4,44
CCL21	Homo sapiens chemokine (C-C motif) ligand 21 (CCL21), mRNA [NM_002989]	8,664	4,235	4,43
THC2678294	FGD5_HUMAN (Q6ZNL6) FYVE, RhoGEF and PH domain-containing protein 5 (Zinc finger FYVE domain-containing protein 23), complete [THC2678294]	9,764	5,341	4,42
PLN	Homo sapiens phospholamban (PLN), mRNA [NM_002667]	7,442	3,044	4,40
C1R	Homo sapiens complement component 1, r subcomponent (C1R), mRNA [NM_001733]	11,652	7,282	4,37
GUCY1A3	Homo sapiens guanylate cyclase 1, soluble, alpha 3 (GUCY1A3), mRNA [NM_000856]	11,471	7,1	4,37
A2M	Homo sapiens alpha-2-macroglobulin (A2M), mRNA [NM_000014]	14,559	10,196	4,36
C1S	Homo sapiens complement component 1, s subcomponent (C1S), transcript variant 1, mRNA [NM_001734]	13,177	8,847	4,33
PTPRB	Homo sapiens protein tyrosine phosphatase, receptor type, B (PTPRB), mRNA [NM_002837]	8,138	3,85	4,29
TM4SF1	Homo sapiens transmembrane 4 L six family member 1 (TM4SF1), mRNA [NM_014220]	10,497	6,282	4,22
SV2B	Homo sapiens synaptic vesicle glycoprotein 2B (SV2B), mRNA [NM_014848]	11,35	7,142	4,21
FBLN5	Homo sapiens fibulin 5 (FBLN5), mRNA [NM_006329]	10,127	5,944	4,18
MMRN2	Homo sapiens multimerin 2 (MMRN2), mRNA [NM_024756]	8,627	4,458	4,17
CXCL14	Homo sapiens chemokine (C-X-C motif) ligand 14 (CXCL14), mRNA [NM_004887]	9,193	5,03	4,16
USHBP1	Homo sapiens Usher syndrome 1C binding protein 1 (USHBP1), mRNA [NM_031941]	7,553	3,398	4,16
PRELP	Homo sapiens proline/arginine-rich end leucine-rich repeat protein (PRELP), transcript variant 1, mRNA [NM_002725]	8,636	4,482	4,15
SYNPO	Synaptopodin. [Source:Uniprot/SWISSPROT;Acc:Q8N3V7] [ENST00000307662]	10,641	6,591	4,05
PTGDS	Homo sapiens prostaglandin D2 synthase 21kDa (brain) (PTGDS), mRNA [NM_000954]	13,564	9,535	4,03
CGNL1	Homo sapiens cingulin-like 1 (CGNL1), mRNA [NM_032866]	11,122	7,104	4,02
GGTLA1	Homo sapiens gamma-glutamyltransferase-like activity 1 (GGTLA1), mRNA [NM_004121]	10,4	6,389	4,01
LRRC32	Homo sapiens leucine rich repeat containing 32 (LRRC32), mRNA [NM_005512]	10,465	6,461	4,00
SEMA3G	Homo sapiens sema domain, immunoglobulin domain (Ig), short basic domain, secreted, (semaphorin) 3G (SEMA3G), mRNA [NM_020163]	8,136	4,139	4,00
CLDN5	Homo sapiens claudin 5 (transmembrane protein deleted in velocardiofacial syndrome) (CLDN5), mRNA [NM_003277]	12,577	8,589	3,99
NPR1	Homo sapiens natriuretic peptide receptor A/guanylate cyclase A (atrionatriuretic peptide receptor A) (NPR1), mRNA [NM_000906]	7,425	3,441	3,98
KCNE4	Homo sapiens potassium voltage-gated channel, Isk-related family, member 4 (KCNE4), mRNA [NM_080671]	8,916	4,944	3,97
GPC3	Homo sapiens glypican 3 (GPC3), mRNA [NM_004484]	7,86	3,911	3,95
THC2527772	HUMC4AA2 complement component C4A {Homo sapiens} (exp=-1; wgp=0; cg=0), partial (6%) [THC2527772]	9,435	5,5	3,94

Supplemental Table 1
Morandi et al

COL27A1	Homo sapiens cDNA FLJ11895 fis, clone HEMBA1007301, weakly similar to COLLAGEN ALPHA 1(III) CHAIN. [AK021957]	10,405	6,481	3,92
LOC642652	PREDICTED: Homo sapiens hypothetical LOC642652 (LOC642652), mRNA [XR_016196]	10,654	6,735	3,92
SPRY1	Homo sapiens sprouty homolog 1, antagonist of FGF signaling (Drosophila) (SPRY1), transcript variant 2, mRNA [NM_199327]	8,606	4,732	3,87
GPR4	Homo sapiens G protein-coupled receptor 4 (GPR4), mRNA [NM_005282]	8,708	4,837	3,87
FCN3	Homo sapiens ficolin (collagen/fibrinogen domain containing) 3 (Hakata antigen) (FCN3), transcript variant 1, mRNA [NM_003665]	7,639	3,793	3,85
S100B	Homo sapiens S100 calcium binding protein B (S100B), mRNA [NM_006272]	9,459	5,631	3,83
DCN	Homo sapiens decorin (DCN), transcript variant A1, mRNA [NM_001920]	12,998	9,171	3,83
ISLR	Homo sapiens immunoglobulin superfamily containing leucine-rich repeat (ISLR), transcript variant 1, mRNA [NM_005545]	11,541	7,714	3,83
CCL15	Homo sapiens chemokine (C-C motif) ligand 15 (CCL15), transcript variant 2, mRNA [NM_004167]	8,927	5,105	3,82
AGT	Homo sapiens angiotensinogen (serpin peptidase inhibitor, clade A, member 8) (AGT), mRNA [NM_000029]	8,861	5,08	3,78
KIAA0672	Homo sapiens KIAA0672 gene product (KIAA0672), mRNA [NM_014859]	10,113	6,342	3,77
AK000872	Homo sapiens cDNA FLJ10010 fis, clone HEMBA1000302. [AK000872]	7,382	3,613	3,77
TTYH1	Homo sapiens tweety homolog 1 (Drosophila) (TTYH1), transcript variant 1, mRNA [NM_020659]	9,068	5,309	3,76
AOC3	Homo sapiens amine oxidase, copper containing 3 (vascular adhesion protein 1) (AOC3), mRNA [NM_003734]	9,697	5,939	3,76
MYCT1	myc target 1 [Source:RefSeq_peptide;Acc:NP_079383] [ENST00000367245]	6,924	3,177	3,75
FMO3	Homo sapiens flavin containing monooxygenase 3 (FMO3), transcript variant 2, mRNA [NM_001002294]	8,046	4,315	3,73
FXYD1	Homo sapiens FXYD domain containing ion transport regulator 1 (phospholemman) (FXYD1), transcript variant a, mRNA [NM_005031]	6,676	2,948	3,73
DKFZp434B1231	Homo sapiens eEF1A2 binding protein (DKFZp434B1231), mRNA [NM_178275]	6,872	3,147	3,73
PDGFRB	Homo sapiens platelet-derived growth factor receptor, beta polypeptide (PDGFRB), mRNA [NM_002609]	11,977	8,258	3,72
DOCK6	Homo sapiens dedicator of cytokinesis 6 (DOCK6), mRNA [NM_020812]	9,343	5,632	3,71
DPT	Homo sapiens dermatopontin (DPT), mRNA [NM_001937]	10,145	6,436	3,71
ANKRD47	Homo sapiens ankyrin repeat domain 47 (ANKRD47), mRNA [NM_198471]	9,453	5,748	3,70
ITIH5	Homo sapiens inter-alpha (globulin) inhibitor H5 (ITIH5), transcript variant 1, mRNA [NM_030569]	8,278	4,579	3,70
NTRK1	Homo sapiens neurotrophic tyrosine kinase, receptor, type 1 (NTRK1), transcript variant 2, mRNA [NM_002529]	10,881	7,185	3,70
EHD2	Homo sapiens EH-domain containing 2 (EHD2), mRNA [NM_014601]	10,278	6,589	3,69
ADCY2	Homo sapiens adenylate cyclase 2 (brain) (ADCY2), mRNA [NM_020546]	7,75	4,064	3,69
ALDH1A3	Homo sapiens aldehyde dehydrogenase 1 family, member A3 (ALDH1A3), mRNA [NM_000693]	9,461	5,813	3,65
CGI-38	Homo sapiens brain specific protein (CGI-38), mRNA [NM_016140]	11,292	7,674	3,62
C10orf116	Homo sapiens chromosome 10 open reading frame 116 (C10orf116), mRNA [NM_006829]	11,111	7,5	3,61
KCNJ8	Homo sapiens potassium inwardly-rectifying channel, subfamily J, member 8 (KCNJ8), mRNA [NM_004982]	9,205	5,594	3,61
CFH	Homo sapiens complement factor H (CFH), transcript variant 2, mRNA [NM_001014975]	9,978	6,384	3,60
GNG11	Homo sapiens guanine nucleotide binding protein (G protein), gamma 11 (GNG11), mRNA [NM_004126]	11,728	8,151	3,58

Supplemental Table 1
Morandi et al

PLEKHA4	Homo sapiens pleckstrin homology domain containing, family A (phosphoinositide binding specific) member 4 (PLEKHA4), mRNA [NM_020904]	8,657	5,105	3,55
SPON2	Homo sapiens spondin 2, extracellular matrix protein (SPON2), mRNA [NM_012445]	13,422	9,869	3,55
-	chr2:088891141-088891082	8,786	5,243	3,54
C10orf10	Homo sapiens chromosome 10 open reading frame 10 (C10orf10), mRNA [NM_007021]	11,891	8,351	3,54
ENST00000381655	Probable phospholipid-transporting ATPase IB (EC 3.6.3.1) (ATPase class I type 8A member 2) (ML-1). [Source:Uniprot/SWISSPROT;Acc:Q9NTI2] [ENST00000381655]	11,597	8,084	3,51
MALL	Homo sapiens mal, T-cell differentiation protein-like (MALL), mRNA [NM_005434]	8,733	5,221	3,51
EGFL7	Homo sapiens EGF-like-domain, multiple 7 (EGFL7), transcript variant 2, mRNA [NM_201446]	10,654	7,165	3,49
ENST00000303979	chrY:22040811-22040969	6,592	3,114	3,48
FZD4	Homo sapiens frizzled homolog 4 (Drosophila) (FZD4), mRNA [NM_012193]	10,91	7,435	3,48
NKD2	Homo sapiens naked cuticle homolog 2 (Drosophila) (NKD2), mRNA [NM_033120]	8,767	5,294	3,47
CCDC48	Homo sapiens coiled-coil domain containing 48 (CCDC48), mRNA [NM_024768]	7,604	4,146	3,46
KIAA1914	Homo sapiens KIAA1914 (KIAA1914), transcript variant 1, mRNA [NM_001001936]	9,306	5,852	3,45
IGFBP3	Homo sapiens insulin-like growth factor binding protein 3 (IGFBP3), mRNA	11,933	8,483	3,45
PAPLN	Homo sapiens papilin, proteoglycan-like sulfated glycoprotein (PAPLN), mRNA [NM_173462]	10,219	6,781	3,44
RBP5	Homo sapiens retinol binding protein 5, cellular (RBP5), mRNA [NM_031491]	7,375	3,948	3,43
NID2	Homo sapiens nidogen 2 (osteonidogen) (NID2), mRNA [NM_007361]	9,567	6,151	3,42
SCN2B	Homo sapiens sodium channel, voltage-gated, type II, beta (SCN2B), mRNA [NM_004588]	8,505	5,107	3,40
CPXM2	Homo sapiens carboxypeptidase X (M14 family), member 2 (CPXM2), mRNA [NM_198148]	9,908	6,514	3,39
DKFZP564O0823	Homo sapiens DKFZP564O0823 protein (DKFZP564O0823), mRNA [NM_015393]	8,848	5,458	3,39
ENST00000371189	Nuclear factor 1 A-type (Nuclear factor 1/A) (NF1-A) (NFI-A) (NF-I/A) (CCAAT-box-binding transcription factor) (CTF) (TGGCA-binding protein). [Source:Uniprot/SWISSPROT;Acc:Q12857] [ENST00000371189]	10,155	6,767	3,39
MMP11	Homo sapiens matrix metallopeptidase 11 (stromelysin 3) (MMP11), mRNA [NM_005940]	10,436	7,059	3,38
THC2712687	chr11:110468143-110468084	7,549	4,173	3,38
TENC1	Homo sapiens tensin like C1 domain containing phosphatase (tensin 2) (TENC1), transcript variant 1, mRNA [NM_015319]	9,146	5,771	3,37
PLXDC1	Homo sapiens plexin domain containing 1 (PLXDC1), mRNA [NM_020405]	10,29	6,933	3,36
EDNRB	Homo sapiens endothelin receptor type B (EDNRB), transcript variant 2, mRNA [NM_003991]	8,536	5,183	3,35
PTHR1	Homo sapiens parathyroid hormone receptor 1 (PTHR1), mRNA [NM_000316]	7,61	4,258	3,35
COL18A1	Homo sapiens collagen, type XVIII, alpha 1 (COL18A1), transcript variant 1, mRNA [NM_030582]	14,845	11,496	3,35
RGS16	Homo sapiens regulator of G-protein signalling 16 (RGS16), mRNA [NM_002928]	11,705	8,356	3,35
KIAA1462	Novel protein. [Source:Uniprot/SPTREMBL;Acc:Q5T992] [ENST00000375377]	10,989	7,642	3,35
NDRG2	Homo sapiens NDRG family member 2 (NDRG2), transcript variant 1, mRNA [NM_201535]	13,074	9,728	3,35
SCN4B	Homo sapiens sodium channel, voltage-gated, type IV, beta (SCN4B), mRNA [NM_174934]	9,159	5,858	3,30
INS	Homo sapiens insulin (INS), mRNA [NM_000207]	8,971	5,678	3,29

Supplemental Table 1
Morandi et al

GALNTL4	Homo sapiens UDP-N-acetyl-alpha-D-galactosamine:polypeptide N-acetylgalactosaminyltransferase-like 4 (GALNTL4), mRNA [NM_198516]	10,087	6,809	3,28
MOXD1	Homo sapiens monooxygenase, DBH-like 1 (MOXD1), transcript variant 2, mRNA [NM_015529]	9,566	6,289	3,28
MYLK	Homo sapiens myosin, light chain kinase (MYLK), transcript variant 1, mRNA [NM_053025]	11,713	8,436	3,28
LAMB2	Homo sapiens laminin, beta 2 (laminin S) (LAMB2), mRNA [NM_002292]	12,873	9,608	3,27
ACVRL1	Homo sapiens activin A receptor type II-like 1 (ACVRL1), transcript variant 1, mRNA [NM_000020]	11,1	7,849	3,25
MATN2	Homo sapiens matrilin 2 (MATN2), transcript variant 2, mRNA [NM_030583]	9,79	6,54	3,25
FOXD2	Homo sapiens forkhead box D2 (FOXD2), mRNA [NM_004474]	7,298	4,052	3,25
C1QTNF5	Homo sapiens C1q and tumor necrosis factor related protein 5 (C1QTNF5), mRNA [NM_015645]	10,366	7,124	3,24
CFHR3	Homo sapiens complement factor H-related 3 (CFHR3), mRNA [NM_021023]	8,466	5,236	3,23
TIE1	Homo sapiens tyrosine kinase with immunoglobulin-like and EGF-like domains 1 (TIE1), mRNA [NM_005424]	8,003	4,775	3,23
TNC	Homo sapiens tenascin C (hexabrachion) (TNC), mRNA [NM_002160]	11,253	8,028	3,23
TGFB3	Homo sapiens transforming growth factor, beta 3 (TGFB3), mRNA [NM_003239]	9,061	5,837	3,22
IGF2	Homo sapiens insulin-like growth factor 2 (somatomedin A) (IGF2), transcript variant 1, mRNA [NM_000612]	13,928	10,706	3,22
ESAM	Homo sapiens endothelial cell adhesion molecule (ESAM), mRNA [NM_138961]	8,228	5,009	3,22
GPM6B	Homo sapiens glycoprotein M6B (GPM6B), transcript variant 2, mRNA [NM_001001996]	7,988	4,769	3,22
THC2545097	IBP7_HUMAN (Q16270) Insulin-like growth factor-binding protein 7 precursor (IGFBP-7) (IBP-7) (IGF-binding protein 7) (MAC25 protein) (Prostacyclin-stimulating factor) (PGI2-stimulating factor) (IGFBP-rP1), partial (58%) [THC2545097]	13,933	10,73	3,20
NLF2	PREDICTED: Homo sapiens nuclear localized factor 2 (NLF2), mRNA [XM_940314]	10,887	7,686	3,20
SPON1	Homo sapiens spondin 1, extracellular matrix protein (SPON1), mRNA [NM_006108]	9,308	6,117	3,19
CYB561	Homo sapiens cytochrome b-561 (CYB561), transcript variant 2, mRNA [NM_001017916]	10,586	7,399	3,19
ATP1B2	Homo sapiens ATPase, Na+/K+ transporting, beta 2 polypeptide (ATP1B2), mRNA [NM_001678]	9,137	5,962	3,18
LAMA4	Homo sapiens laminin, alpha 4 (LAMA4), mRNA [NM_002290]	12,775	9,603	3,17
CX3CL1	Homo sapiens chemokine (C-X3-C motif) ligand 1 (CX3CL1), mRNA [NM_002996]	7,828	4,672	3,16
TFPI	Homo sapiens tissue factor pathway inhibitor (lipoprotein-associated coagulation inhibitor) (TFPI), transcript variant 1, mRNA [NM_006287]	10,548	7,405	3,14
FLJ36748	Homo sapiens hypothetical protein FLJ36748 (FLJ36748), mRNA [NM_152406]	9,266	6,131	3,14
ABLIM3	Homo sapiens actin binding LIM protein family, member 3 (ABLIM3), mRNA [NM_014945]	11,743	8,623	3,12
LHFP	Homo sapiens lipoma HMGIC fusion partner (LHFP), mRNA [NM_005780]	12,756	9,637	3,12
C16orf30	Homo sapiens chromosome 16 open reading frame 30 (C16orf30), mRNA [NM_024600]	12,304	9,194	3,11
C3	Homo sapiens complement component 3 (C3), mRNA [NM_000064]	10,863	7,758	3,11
PRPF19	Homo sapiens PRP19/PSO4 pre-mRNA processing factor 19 homolog (S. cerevisiae) (PRPF19), mRNA [NM_014502]	9,46	6,357	3,10
ALDH1A1	Homo sapiens aldehyde dehydrogenase 1 family, member A1 (ALDH1A1), mRNA [NM_000689]	9,908	6,807	3,10
HRC	Homo sapiens histidine rich calcium binding protein (HRC), mRNA [NM_002152]	6,913	3,813	3,10
IGSF1	Homo sapiens immunoglobulin superfamily, member 1 (IGSF1), transcript variant 1, mRNA [NM_001555]	7,015	3,917	3,10

Supplemental Table 1
Morandi et al

RHBDF1	Homo sapiens rhomboid 5 homolog 1 (Drosophila) (RHBDF1), mRNA [NM_022450]	9,244	6,149	3,10
THC2559002	chr17:037808033-037808092	9,344	6,249	3,10
MYO5C	Homo sapiens myosin VC (MYO5C), mRNA [NM_018728]	6,273	3,186	3,09
LAMC3	Homo sapiens laminin, gamma 3 (LAMC3), mRNA [NM_006059]	7,451	4,366	3,09
C10orf56	Homo sapiens chromosome 10 open reading frame 56 (C10orf56), mRNA [NM_153367]	8,622	5,539	3,08
ITGB4	Homo sapiens integrin, beta 4 (ITGB4), transcript variant 1, mRNA [NM_000213]	8,626	5,546	3,08
LGI4	Homo sapiens leucine-rich repeat LGI family, member 4 (LGI4), mRNA [NM_139284]	10,021	6,944	3,08
INHBB	Homo sapiens inhibin, beta B (activin AB beta polypeptide) (INHBB), mRNA [NM_002193]	9,603	6,536	3,07
SGEF	Homo sapiens infant liver cDNA, clone:HMFN1864, full insert sequence. [AB073386]	7,447	4,381	3,07
HEPH	Homo sapiens hephaestin (HEPH), transcript variant 2, mRNA [NM_014799]	8,081	5,02	3,06
C4B	Homo sapiens complement component 4B (Childo blood group) (C4B), mRNA [NM_001002029]	10,59	7,535	3,06
IFI27	Homo sapiens interferon, alpha-inducible protein 27 (IFI27), mRNA [NM_005532]	13,611	10,562	3,05
FILIP1L	Homo sapiens filamin A interacting protein 1-like (FILIP1L), transcript variant 1, mRNA [NM_182909]	9,081	6,035	3,05
-	chr17:071265428-071265488	9,243	6,199	3,05
SOX8	Homo sapiens SRY (sex determining region Y)-box 8 (SOX8), mRNA [NM_014587]	9,031	5,99	3,04
ZNF366	Zinc finger protein 366. [Source:Uniprot/SWISSPROT;Acc:Q8N895] [ENST00000380632]	6,691	3,662	3,03
RASIP1	Homo sapiens Ras interacting protein 1 (RASIP1), mRNA [NM_017805]	11,362	8,343	3,02
AL833655	Homo sapiens mRNA; cDNA DKFZp667O0320 (from clone DKFZp667O0320). [AL833655]	7,578	4,56	3,02
ADRA1B	Homo sapiens adrenergic, alpha-1B-, receptor (ADRA1B), mRNA [NM_000679]	9,61	6,597	3,01
NFIA	Homo sapiens nuclear factor I/A (NFIA), mRNA [NM_005595]	9,993	6,984	3,01
SLCO2B1	Homo sapiens solute carrier organic anion transporter family, member 2B1 (SLCO2B1), mRNA [NM_007256]	10,296	7,292	3,00
HOXB9	Homo sapiens homeobox B9 (HOXB9), mRNA [NM_024017]	5,963	2,962	3,00
GEFT	Homo sapiens RAC/CDC42 exchange factor (GEFT), transcript variant 1, mRNA [NM_182947]	9,803	6,804	3,00
AK026826	Homo sapiens cDNA: FLJ23173 fis, clone LNG10019. [AK026826]	8,083	5,092	2,99
LOC645277	PREDICTED: Homo sapiens hypothetical LOC645277 (LOC645277), mRNA [XM_928321]	6,677	3,693	2,98
FMO1	Homo sapiens flavin containing monooxygenase 1 (FMO1), mRNA [NM_002021]	6,526	3,547	2,98
ITIH3	Homo sapiens inter-alpha (globulin) inhibitor H3 (ITIH3), mRNA [NM_002217]	7,218	4,241	2,98
SULT4A1	Homo sapiens sulfotransferase family 4A, member 1 (SULT4A1), mRNA [NM_014351]	10,163	7,196	2,97
BCL2	Homo sapiens B-cell CLL/lymphoma 2 (BCL2), nuclear gene encoding mitochondrial protein, transcript variant alpha, mRNA [NM_000633]	11,728	8,773	2,96
OLFML1	Homo sapiens olfactomedin-like 1 (OLFML1), mRNA [NM_198474]	6,195	3,247	2,95
AK124080	Homo sapiens cDNA FLJ42086 fis, clone TESOP1000127. [AK124080]	5,932	2,986	2,95
SPRY4	Homo sapiens sprouty homolog 4 (Drosophila) (SPRY4), mRNA [NM_030964]	8,81	5,867	2,94
TMEM176B	Homo sapiens transmembrane protein 176B (TMEM176B), mRNA [NM_014020]	11,092	8,155	2,94
CCDC3	Homo sapiens coiled-coil domain containing 3 (CCDC3), mRNA [NM_031455]	12,018	9,086	2,93
RAPGEF3	Homo sapiens Rap guanine nucleotide exchange factor (GEF) 3 (RAPGEF3), mRNA [NM_006105]	6,723	3,801	2,92

Supplemental Table 1
Morandi et al

FMOD	Homo sapiens fibromodulin (FMOD), mRNA [NM_002023]	11,78	8,864	2,92
AW138903	AW138903 UI-H-BI1-aeq-e-09-0-UI.s1 NCI_CGAP_Sub3 Homo sapiens cDNA clone IMAGE:2720344 3', mRNA sequence [AW138903]	9,476	6,56	2,92
KIAA1576	Homo sapiens KIAA1576 protein (KIAA1576), mRNA [NM_020927]	12,857	9,94	2,92
C2orf40	Homo sapiens chromosome 2 open reading frame 40 (C2orf40), mRNA [NM_032411]	7,299	4,386	2,91
BX097190	BX097190 Soares placenta Nb2HP Homo sapiens cDNA clone IMAGp998G19212, mRNA sequence [BX097190]	12,303	9,392	2,91
IGF1	Homo sapiens insulin-like growth factor 1 (somatomedin C) (IGF1), mRNA [NM_000618]	9,787	6,884	2,90
PI16	Homo sapiens peptidase inhibitor 16 (PI16), mRNA [NM_153370]	7,455	4,564	2,89
COL14A1	Homo sapiens collagen, type XIV, alpha 1 (undulin) (COL14A1), mRNA [NM_021110]	7,949	5,06	2,89
SNCG	Homo sapiens synuclein, gamma (breast cancer-specific protein 1) (SNCG), mRNA [NM_003087]	8,093	5,212	2,88
AF086288	Homo sapiens full length insert cDNA clone ZD48A05. [AF086288]	7,132	4,257	2,88
RERG	Homo sapiens RAS-like, estrogen-regulated, growth inhibitor (RERG), mRNA [NM_032918]	7,905	5,03	2,88
C20orf160	Homo sapiens chromosome 20 open reading frame 160 (C20orf160), mRNA [NM_080625]	7,542	4,669	2,87
PROCR	Homo sapiens protein C receptor, endothelial (EPCR) (PROCR), mRNA [NM_006404]	9,948	7,074	2,87
BQ897248	AGENCOURT_8122036 Lupski_dorsal_root_ganglion Homo sapiens cDNA clone IMAGE:6179261 5', mRNA sequence [BQ897248]	6,269	3,42	2,85
NEFL	Homo sapiens neurofilament, light polypeptide 68kDa (NEFL), mRNA [NM_006158]	10,921	8,077	2,84
C1orf115	Homo sapiens chromosome 1 open reading frame 115 (C1orf115), mRNA [NM_024709]	6,127	3,288	2,84
DKK3	Homo sapiens dickkopf homolog 3 (Xenopus laevis) (DKK3), transcript variant 1, mRNA [NM_015881]	12,003	9,164	2,84
CXCL9	Homo sapiens chemokine (C-X-C motif) ligand 9 (CXCL9), mRNA [NM_002416]	8,368	5,536	2,83
CPNE6	Homo sapiens copine VI (neuronal) (CPNE6), mRNA [NM_006032]	9,012	6,182	2,83
COL20A1	Homo sapiens collagen, type XX, alpha 1 (COL20A1), mRNA [NM_020882]	7,767	4,94	2,83
TSPAN12	Homo sapiens tetraspanin 12 (TSPAN12), mRNA [NM_012338]	6,626	3,8	2,83
FKBP10	Homo sapiens FK506 binding protein 10, 65 kDa (FKBP10), mRNA [NM_021939]	11,851	9,031	2,82
-	chr8:102266589-102266530	10,403	7,588	2,82
RPESP	Homo sapiens RPE-spondin (RPESP), mRNA [NM_153225]	7,599	4,784	2,81
APLN	Homo sapiens apelin, AGTRL1 ligand (APLN), mRNA [NM_017413]	8,108	5,295	2,81
SYT4	Homo sapiens synaptotagmin IV (SYT4), mRNA [NM_020783]	13,272	10,46	2,81
GRRP1	Homo sapiens glycine/arginine rich protein 1 (GRRP1), mRNA [NM_024869]	9,47	6,66	2,81
ABCG2	Homo sapiens ATP-binding cassette, sub-family G (WHITE), member 2 (ABCG2), mRNA [NM_004827]	8	5,201	2,80
COL6A2	Homo sapiens collagen, type VI, alpha 2 (COL6A2), transcript variant 2C2a', mRNA [NM_058175]	9,847	7,049	2,80
ABCB1	Homo sapiens ATP-binding cassette, sub-family B (MDR/TAP), member 1 (ABCB1), mRNA [NM_000927]	7,662	4,87	2,79
FLT1	Homo sapiens fms-related tyrosine kinase 1 (vascular endothelial growth factor/vascular permeability factor receptor) (FLT1), mRNA [NM_002019]	6,796	4,005	2,79
TMEM176A	Homo sapiens transmembrane protein 176A (TMEM176A), mRNA [NM_018487]	11,25	8,462	2,79
ERG	Homo sapiens v-ets erythroblastosis virus E26 oncogene homolog (avian) (ERG), transcript variant 2, mRNA [NM_004449]	8,727	5,939	2,79
-	chr10:049893011-049892822	7,778	4,996	2,78
BGN	Homo sapiens biglycan (BGN), mRNA [NM_001711]	8,187	5,409	2,78

Supplemental Table 1
Morandi et al

CIRBP	Homo sapiens cDNA FLJ46566 fis, clone THYMU3040829, moderately similar to Cold-inducible RNA-binding protein. [AK128423]	10,041	7,265	2,78
GIMAP8	Homo sapiens GTPase, IMAP family member 8 (GIMAP8), mRNA [NM_175571]	8,891	6,118	2,77
SEMA5B	Homo sapiens sema domain, seven thrombospondin repeats (type 1 and type 1-like), transmembrane domain (TM) and short cytoplasmic domain, (semaphorin) 5B (SEMA5B), transcript variant 1, mRNA [NM_001031702]	9,161	6,389	2,77
PLA2G5	Homo sapiens phospholipase A2, group V (PLA2G5), mRNA [NM_000929]	7,426	4,658	2,77
TSPAN9	Homo sapiens tetraspanin 9 (TSPAN9), mRNA [NM_006675]	8,109	5,345	2,76
CLSTN3	Homo sapiens calsyntenin 3 (CLSTN3), mRNA [NM_014718]	9,117	6,356	2,76
KIAA0319	Homo sapiens KIAA0319 (KIAA0319), mRNA [NM_014809]	7,743	4,993	2,75
-	chr12:118565139-118565198	8,22	5,47	2,75
HOP	Homo sapiens homeodomain-only protein (HOP), transcript variant 2, mRNA [NM_139211]	8,821	6,075	2,75
APOL6	Homo sapiens apolipoprotein L, 6 (APOL6), mRNA [NM_030641]	7,834	5,095	2,74
WNT6	Homo sapiens wingless-type MMTV integration site family, member 6 (WNT6), mRNA [NM_006522]	9,494	6,756	2,74
AATK	Homo sapiens cDNA FLJ16758 fis, clone BRACE3038687, moderately similar to Homo sapiens apoptosis-associated tyrosine kinase (AATK). [AK131529]	13,107	10,375	2,73
CARD10	Homo sapiens caspase recruitment domain family, member 10 (CARD10), mRNA [NM_014550]	10,213	7,484	2,73
SLC30A4	Homo sapiens solute carrier family 30 (zinc transporter), member 4 (SLC30A4), mRNA [NM_013309]	7,02	4,293	2,73
FBLN2	Homo sapiens fibulin 2 (FBLN2), transcript variant 1, mRNA [NM_001004019]	9,929	7,204	2,72
DNASE1L3	Homo sapiens deoxyribonuclease I-like 3 (DNASE1L3), mRNA [NM_004944]	9,71	6,996	2,71
FILIP1	Homo sapiens filamin A interacting protein 1 (FILIP1), mRNA [NM_015687]	7,458	4,749	2,71
SDPR	Homo sapiens serum deprivation response (phosphatidylserine binding protein) (SDPR), mRNA [NM_004657]	6,835	4,132	2,70
EML2	Homo sapiens echinoderm microtubule associated protein like 2 (EML2), mRNA [NM_012155]	9,918	7,219	2,70
ADAMTS9	Homo sapiens ADAM metallopeptidase with thrombospondin type 1 motif, 9 (ADAMTS9), mRNA [NM_182920]	9,433	6,74	2,69
RNASE1	Homo sapiens ribonuclease, RNase A family, 1 (pancreatic) (RNASE1), transcript variant 3, mRNA [NM_198232]	10,975	8,296	2,68
CR604283	full-length cDNA clone CS0DF012YB15 of Fetal brain of Homo sapiens (human). [CR604283]	8,998	6,333	2,67
BCAN	Homo sapiens brevican, mRNA (cDNA clone IMAGE:3618761), partial cds. [BC005081]	6,728	4,067	2,66
ITM2C	Homo sapiens integral membrane protein 2C (ITM2C), transcript variant 1, mRNA [NM_030926]	10,915	8,257	2,66
RSPO3	Homo sapiens R-spondin 3 homolog (Xenopus laevis) (RSPO3), mRNA [NM_032784]	6,402	3,745	2,66
RAB6B	Homo sapiens RAB6B, member RAS oncogene family (RAB6B), mRNA [NM_016577]	11,863	9,212	2,65
SASH1	Homo sapiens SAM and SH3 domain containing 1 (SASH1), mRNA [NM_015278]	11,749	9,105	2,64
-	chr17:022320734-022320784	7,67	5,025	2,64
POLR3E	Homo sapiens mRNA for KIAA1452 protein, partial cds. [AB040885]	5,626	2,983	2,64
ENST00000370548	Heparan sulfate 2-O-sulfotransferase 1 (EC 2.8.2.-) (2-O-sulfotransferase) (2OST). [Source:Uniprot/SWISSPROT;Acc:Q7LGA3] [ENST00000370548]	7,122	4,504	2,62
LOC342979	PREDICTED: Homo sapiens hypothetical LOC342979 (LOC342979), mRNA [XM_292820]	8,001	5,385	2,62
TRPM3	Homo sapiens transient receptor potential cation channel, subfamily M, member 3 (TRPM3), transcript variant 9, mRNA [NM_001007471]	6,686	4,078	2,61

Supplemental Table 1
Morandi et al

CYYR1	Homo sapiens cysteine/tyrosine-rich 1 (CYYR1), mRNA [NM_052954]	7,491	4,884	2,61
-	chr3:014589698-014589540	7,894	5,288	2,61
NCALD	Homo sapiens neurocalcin delta (NCALD), transcript variant 7, mRNA [NM_001040630]	8,815	6,213	2,60
TMEM16B	Homo sapiens transmembrane protein 16B (TMEM16B), mRNA [NM_020373]	7,355	4,754	2,60
LHX6	Homo sapiens LIM homeobox 6 (LHX6), transcript variant 1, mRNA [NM_014368]	8,085	5,486	2,60
ITGA6	Homo sapiens integrin, alpha 6 (ITGA6), transcript variant 2, mRNA [NM_000210]	10,144	7,546	2,60
TINAGL1	Homo sapiens tubulointerstitial nephritis antigen-like 1 (TINAGL1), mRNA [NM_022164]	7,158	4,568	2,59
SEPP1	Homo sapiens selenoprotein P, plasma, 1 (SEPP1), mRNA [NM_005410]	12,619	10,032	2,59
SYTL2	Homo sapiens synaptotagmin-like 2 (SYTL2), transcript variant c, mRNA [NM_206927]	7,925	5,344	2,58
RASGRP3	Homo sapiens RAS guanyl releasing protein 3 (calcium and DAG-regulated) (RASGRP3), mRNA [NM_170672]	7,53	4,958	2,57
LOC595101	Homo sapiens mRNA; cDNA DKFZp686H21113 (from clone DKFZp686H21113). [CR627362]	8,007	5,458	2,55
PCDH21	Homo sapiens protocadherin 21 (PCDH21), mRNA [NM_033100]	7,266	4,722	2,54
F2R	Homo sapiens coagulation factor II (thrombin) receptor (F2R), mRNA [NM_001992]	7,914	5,372	2,54
SLAMF8	Homo sapiens SLAM family member 8 (SLAMF8), mRNA [NM_020125]	9,682	7,142	2,54
THBS4	Homo sapiens thrombospondin 4 (THBS4), mRNA [NM_003248]	10,273	7,734	2,54
JAG1	Homo sapiens jagged 1 (Alagille syndrome) (JAG1), mRNA [NM_000214]	10,583	8,047	2,54
PDGFB	Homo sapiens platelet-derived growth factor beta polypeptide (simian sarcoma viral (v-sis) oncogene homolog) (PDGFB), transcript variant 1, mRNA [NM_002608]	8,063	5,528	2,54
ADAMTSL2	Homo sapiens ADAMTS-like 2 (ADAMTSL2), mRNA [NM_014694]	9,035	6,505	2,53
FLJ22536	Homo sapiens cDNA FLJ12803 fis, clone NT2RP2002172. [AK022865]	10,902	8,373	2,53
BU561469	AGENCOURT_10278709 NIH_MGC_82 Homo sapiens cDNA clone IMAGE:6592525 5', mRNA sequence [BU561469]	8,493	5,963	2,53
ZNF683	Homo sapiens zinc finger protein 683 (ZNF683), mRNA [NM_173574]	8,612	6,083	2,53
IHPK3	Homo sapiens inositol hexaphosphate kinase 3 (IHPK3), mRNA [NM_054111]	5,827	3,303	2,52
BRD3	Homo sapiens bromodomain containing 3 (BRD3), mRNA [NM_007371]	11,395	8,875	2,52
CA314936	UI-CF-FN0-afi-d-17-0-UI.s1 UI-CF-FN0 Homo sapiens cDNA clone UI-CF-FN0-afi-d-17-0-UI 3', mRNA sequence [CA314936]	9,004	6,509	2,50
CHST2	Homo sapiens carbohydrate (N-acetylglucosamine-6-O) sulfotransferase 2 (CHST2), mRNA [NM_004267]	9,043	6,549	2,49
PON3	Homo sapiens paraoxonase 3 (PON3), mRNA [NM_000940]	6,609	4,116	2,49
SH3D19	Homo sapiens SH3 domain protein D19 (SH3D19), mRNA [NM_001009555]	7,868	5,378	2,49
BST2	Homo sapiens bone marrow stromal cell antigen 2 (BST2), mRNA [NM_004335]	11,582	9,092	2,49
ENST00000308894	Homo sapiens HSPC324 mRNA, partial cds. [AF161442]	7,044	4,559	2,49
FGD5	Homo sapiens FYVE, RhoGEF and PH domain containing 5 (FGD5), mRNA [NM_152536]	6,581	4,104	2,48
-	chr12:088278154-088278213	8,454	5,976	2,48
ACP2	Homo sapiens acid phosphatase 2, lysosomal (ACP2), mRNA [NM_001610]	8,565	6,089	2,48
MGC33846	Homo sapiens hypothetical protein MGC33846 (MGC33846), mRNA [NM_175885]	7,77	5,294	2,48
C14orf78	Homo sapiens chromosome 14 open reading frame 78, mRNA (cDNA clone IMAGE:30387958), complete cds. [BC090889]	7,831	5,365	2,47
C1orf170	Homo sapiens cDNA FLJ41861 fis, clone NTONG2008672. [AK123855]	7,124	4,66	2,46

Supplemental Table 1
Morandi et al

ATP2B4	Homo sapiens ATPase, Ca++ transporting, plasma membrane 4 (ATP2B4), transcript variant 1, mRNA [NM_001001396]	12,454	9,992	2,46
COLEC11	Homo sapiens collectin sub-family member 11 (COLEC11), transcript variant 2, mRNA [NM_199235]	11,26	8,799	2,46
ANKRD38	Homo sapiens ankyrin repeat domain 38 (ANKRD38), mRNA [NM_181712]	8,141	5,681	2,46
MRGPRE	Homo sapiens MAS-related GPR, member E (MRGPRE), mRNA [NM_001039165]	6,262	3,804	2,46
SEPT4	Homo sapiens septin 4 (), transcript variant 1, mRNA [NM_004574]	8,842	6,387	2,46
ARHGEF9	Homo sapiens Cdc42 guanine nucleotide exchange factor (GEF) 9 (ARHGEF9), mRNA [NM_015185]	10,185	7,731	2,45
GLI1	Homo sapiens glioma-associated oncogene homolog 1 (zinc finger protein) (GLI1), mRNA [NM_005269]	6,769	4,322	2,45
ADAM33	Homo sapiens ADAM metallopeptidase domain 33 (ADAM33), transcript variant 1, mRNA [NM_025220]	7,685	5,248	2,44
PLEKHG1	Homo sapiens pleckstrin homology domain containing, family G (with RhoGef domain) member 1 (PLEKHG1), mRNA [NM_001029884]	7,05	4,615	2,44
FBXL17	Homo sapiens F-box and leucine-rich repeat protein 17, mRNA (cDNA clone IMAGE:4215262), partial cds. [BC018548]	7,793	5,363	2,43
CR616309	full-length cDNA clone CS0DF015YK23 of Fetal brain of Homo sapiens (human). [CR616309]	7,193	4,765	2,43
HTRA3	Homo sapiens HtrA serine peptidase 3 (HTRA3), mRNA [NM_053044]	11,109	8,684	2,43
LOC339396	PREDICTED: Homo sapiens similar to neurofilament, heavy polypeptide (LOC339396), mRNA [XR_018787]	7,341	4,916	2,43
BHLHB3	Homo sapiens basic helix-loop-helix domain containing, class B, 3 (BHLHB3), mRNA [NM_030762]	8,955	6,532	2,42
TDRD10	Homo sapiens tudor domain containing 10 (TDRD10), mRNA [NM_182499]	6,359	3,938	2,42
-	chr16:019036724-019036783	8,31	5,89	2,42
KCNJ10	Homo sapiens potassium inwardly-rectifying channel, subfamily J, member 10 (KCNJ10), mRNA [NM_002241]	6,789	4,387	2,40
ANKRD25	Homo sapiens ankyrin repeat domain 25 (ANKRD25), mRNA [NM_015493]	8,339	5,94	2,40
GPRC5C	Homo sapiens G protein-coupled receptor, family C, group 5, member C (GPRC5C), transcript variant 1, mRNA [NM_022036]	6,372	3,982	2,39
GIMAP5	Homo sapiens GTPase, IMAP family member 5 (GIMAP5), mRNA [NM_018384]	9,437	7,047	2,39
VIPR2	Homo sapiens vasoactive intestinal peptide receptor 2 (VIPR2), mRNA [NM_003382]	6,848	4,459	2,39
CCDC8	Homo sapiens coiled-coil domain containing 8 (CCDC8), mRNA [NM_032040]	7,947	5,559	2,39
PLXND1	Homo sapiens plexin D1 (PLXND1), mRNA [NM_015103]	7,338	4,96	2,38
KIAA1274	Homo sapiens KIAA1274 (KIAA1274), mRNA [NM_014431]	8,228	5,858	2,37
HEY1	Homo sapiens hairy/enhancer-of-split related with YRPW motif 1 (HEY1), transcript variant 2, mRNA [NM_001040708]	11,018	8,657	2,36
KIAA0485	Homo sapiens mRNA, chromosome 1 specific transcript KIAA0485. [AB007954]	8,179	5,821	2,36
RASL12	Homo sapiens RAS-like, family 12 (RASL12), mRNA [NM_016563]	7,135	4,779	2,36
GDF10	Homo sapiens growth differentiation factor 10 (GDF10), mRNA [NM_004962]	7,009	4,656	2,35
-	chr5:134287638-134287579	8,667	6,314	2,35
ENST00000357603	C14orf78 protein (Fragment). [Source:Uniprot/SPTREMBL;Acc:Q96EX9] [ENST00000357603]	6,022	3,67	2,35
PLA2G2D	Homo sapiens phospholipase A2, group IID (PLA2G2D), mRNA [NM_012400]	6,351	4,002	2,35
GPR124	Homo sapiens G protein-coupled receptor 124 (GPR124), mRNA [NM_032777]	10,347	8,004	2,34
C1orf34	Homo sapiens chromosome 1 open reading frame 34, mRNA (cDNA clone IMAGE:4827153), partial cds. [BC028374]	8,452	6,111	2,34
ARHGEF16	Homo sapiens Rho guanine exchange factor (GEF) 16 (ARHGEF16), mRNA [NM_014448]	6,221	3,88	2,34

Supplemental Table 1
Morandi et al

PLA2G4C	Homo sapiens phospholipase A2, group IVC (cytosolic, calcium-independent) (PLA2G4C), mRNA [NM_003706]	9,72	7,382	2,34
ANGPTL2	Homo sapiens angiopoietin-like 2 (ANGPTL2), mRNA [NM_012098]	8,208	5,871	2,34
SLC7A4	Homo sapiens solute carrier family 7 (cationic amino acid transporter, y+ system), member 4 (SLC7A4), mRNA [NM_004173]	8,889	6,557	2,33
WFDC1	Homo sapiens WAP four-disulfide core domain 1 (WFDC1), mRNA [NM_021197]	7,152	4,82	2,33
THC2598362	Q59F29_HUMAN (Q59F29) PI-3-kinase-related kinase SMG-1 isoform 1homolog (Fragment), partial (4%) [THC2598362]	8,78	6,452	2,33
SPOCK2	Homo sapiens sparc/osteonectin, cwcv and kazal-like domains proteoglycan (testican) 2 (SPOCK2), mRNA [NM_014767]	8,327	6,007	2,32
FLJ38359	Homo sapiens cDNA FLJ38359 fis, clone FEBRA2000321. [AK095678]	10,03	7,716	2,31
SVEP1	Homo sapiens sushi, von Willebrand factor type A, EGF and pentraxin domain containing 1 (SVEP1), mRNA [NM_153366]	8,702	6,392	2,31
-	chr1:201976875-201976816	8,143	5,835	2,31
PFKFB3	Homo sapiens 6-phosphofructo-2-kinase/fructose-2,6-biphosphatase 3 (PFKFB3), mRNA [NM_004566]	7,54	5,237	2,30
PAQR8	Homo sapiens progestin and adipoQ receptor family member VIII (PAQR8), mRNA [NM_133367]	10,286	7,983	2,30
THC2622092	Q5PP12_ARATH (Q5PP12) At5g40730, partial (23%) [THC2622092]	7,825	5,524	2,30
TAF5L	Homo sapiens TAF5-like RNA polymerase II, p300/CBP-associated factor (PCAF)-associated factor, 65kDa (TAF5L), transcript variant 1, mRNA [NM_014409]	8,73	6,433	2,30
ZNF264	Homo sapiens zinc finger protein 264 (ZNF264), mRNA [NM_003417]	9,187	6,894	2,29
AK001116	Homo sapiens cDNA FLJ10254 fis, clone HEMBB1000848. [AK001116]	8,162	5,871	2,29
FLJ31485	Homo sapiens cDNA FLJ31485 fis, clone NT2NE2001698. [AK056047]	5,709	3,417	2,29
AK022045	Homo sapiens cDNA FLJ11983 fis, clone HEMBB1001337. [AK022045]	8,143	5,854	2,29
CD163L1	Homo sapiens CD163 molecule-like 1 (CD163L1), mRNA [NM_174941]	9,288	7,004	2,28
STON1	Homo sapiens stonin 1 (STON1), mRNA [NM_006873]	8,485	6,202	2,28
AK024470	Homo sapiens mRNA for FLJ00063 protein, partial cds. [AK024470]	7,117	4,835	2,28
LOC157562	Homo sapiens mRNA; cDNA DKFZp566J123 (from clone DKFZp566J123). [AL050061]	10,438	8,156	2,28
CLEC3B	Homo sapiens C-type lectin domain family 3, member B (CLEC3B), mRNA [NM_003278]	12,547	10,267	2,28
PROS1	Homo sapiens protein S (alpha) (PROS1), mRNA [NM_000313]	8,929	6,649	2,28
EGFL8	Homo sapiens EGF-like-domain, multiple 8 (EGFL8), mRNA [NM_030652]	8,275	5,996	2,28
CILP	Homo sapiens cartilage intermediate layer protein, nucleotide pyrophosphohydrolase (CILP), mRNA [NM_003613]	8,194	5,917	2,28
CCR3	Homo sapiens chemokine (C-C motif) receptor 3 (CCR3), transcript variant 1, mRNA [NM_001837]	7,304	5,027	2,28
SLC39A7	Homo sapiens solute carrier family 39 (zinc transporter), member 7 (SLC39A7), transcript variant 1, mRNA [NM_006979]	11,225	8,951	2,27
CR626252	full-length cDNA clone CS0DD001YO10 of Neuroblastoma Cot 50-normalized of Homo sapiens (human). [CR626252]	7,552	5,279	2,27
A4GALT	Homo sapiens alpha 1,4-galactosyltransferase (globotriaosylceramide synthase) (A4GALT), mRNA [NM_017436]	8,23	5,956	2,27
FBXO2	Homo sapiens F-box protein 2 (FBXO2), mRNA [NM_012168]	11,015	8,746	2,27
SYNGR3	Homo sapiens synaptogyrin 3 (SYNGR3), mRNA [NM_004209]	10,609	8,345	2,27
GRIN2C	Homo sapiens glutamate receptor, ionotropic, N-methyl D-aspartate 2C (GRIN2C), mRNA [NM_000835]	8,549	6,286	2,26
PDLIM5	Homo sapiens PDZ and LIM domain 5 (PDLIM5), transcript variant 1, mRNA [NM_006457]	9,059	6,804	2,26

Supplemental Table 1
Morandi et al

EDA	Homo sapiens ectodysplasin A (EDA), transcript variant 1, mRNA [NM_001399]	7,481	5,231	2,25
AK123704	Homo sapiens cDNA FLJ41710 fis, clone HLUNG2011041, weakly similar to Basic proline-rich peptide IB-8a. [AK123704]	7,973	5,723	2,25
AK091904	Homo sapiens cDNA FLJ34585 fis, clone KIDNE2008758. [AK091904]	9,124	6,875	2,25
PFDN6	Homo sapiens prefoldin subunit 6 (PFDN6), mRNA [NM_014260]	11,742	9,496	2,25
TNFRSF4	Homo sapiens tumor necrosis factor receptor superfamily, member 4 (TNFRSF4), mRNA [NM_003327]	7,521	5,277	2,24
INDO	Homo sapiens indoleamine-pyrrole 2,3 dioxygenase (INDO), mRNA [NM_002164]	7,68	5,442	2,24
KIAA1183	Homo sapiens KIAA1183 protein, mRNA (cDNA clone IMAGE:40108157), partial cds. [BC121810]	8,106	5,868	2,24
ZBTB46	Homo sapiens cDNA FLJ44766 fis, clone BRACE3032537. [AK126720]	8,485	6,249	2,24
HSPA12B	Homo sapiens heat shock 70kD protein 12B (HSPA12B), mRNA [NM_052970]	9,282	7,047	2,24
PLCD4	Homo sapiens phospholipase C, delta 4 (PLCD4), mRNA [NM_032726]	9,792	7,557	2,24
C1QTNF1	Homo sapiens C1q and tumor necrosis factor related protein 1 (C1QTNF1), mRNA [NM_198594]	6,647	4,417	2,23
MCAM	Homo sapiens melanoma cell adhesion molecule (MCAM), mRNA [NM_006500]	12,154	9,924	2,23
BX647543	Homo sapiens mRNA; cDNA DKFZp779F2345 (from clone DKFZp779F2345). [BX647543]	7,092	4,863	2,23
EHD3	Homo sapiens EH-domain containing 3 (EHD3), mRNA [NM_014600]	9,257	7,031	2,23
ATM	Homo sapiens ataxia telangiectasia mutated (includes complementation groups A, C and D) (ATM), transcript variant 1, mRNA [NM_000051]	7,179	4,953	2,23
ANGPT2	Homo sapiens angiopoietin 2 (ANGPT2), mRNA [NM_001147]	9,129	6,905	2,22
MRVI1	Homo sapiens murine retrovirus integration site 1 homolog (MRVI1), transcript variant 2, mRNA [NM_130385]	8,207	5,987	2,22
PDLIM2	Homo sapiens mRNA for FLJ00072 protein. [AK074031]	6,061	3,841	2,22
NOS3	Homo sapiens nitric oxide synthase 3 (endothelial cell) (NOS3), mRNA [NM_000603]	7,548	5,334	2,21
GPRC5B	Homo sapiens G protein-coupled receptor, family C, group 5, member B (GPRC5B), mRNA [NM_016235]	8,719	6,511	2,21
RETSAT	Homo sapiens retinol saturase (all-trans-retinol 13,14-reductase) (RETSAT), mRNA [NM_017750]	8,509	6,303	2,21
LOC644596	Homo sapiens cDNA FLJ41308 fis, clone BRAMY2042612. [AK123302]	12,663	10,458	2,21
FABP3	Homo sapiens fatty acid binding protein 3, muscle and heart (mammary-derived growth inhibitor) (FABP3), mRNA [NM_004102]	8,675	6,469	2,21
BX537510	Homo sapiens mRNA; cDNA DKFZp686M1488 (from clone DKFZp686M1488). [BX537510]	6,172	3,97	2,20
HSMPP8	Homo sapiens M-phase phosphoprotein, mpp8 (HSMPP8), mRNA [NM_017520]	9,114	6,914	2,20
SEMA4C	Homo sapiens sema domain, immunoglobulin domain (Ig), transmembrane domain (TM) and short cytoplasmic domain, (semaphorin) 4C (SEMA4C), mRNA [NM_017789]	8,8	6,602	2,20
LAMA5	Homo sapiens laminin, alpha 5 (LAMA5), mRNA [NM_005560]	8,362	6,167	2,20
ANK3	Homo sapiens ankyrin 3, node of Ranvier (ankyrin G) (ANK3), transcript variant 1, mRNA [NM_020987]	12,645	10,45	2,20
AK074696	Homo sapiens cDNA FLJ90215 fis, clone MAMMA1002205. [AK074696]	8,269	6,078	2,19
CYP27A1	Homo sapiens cytochrome P450, family 27, subfamily A, polypeptide 1 (CYP27A1), nuclear gene encoding mitochondrial protein, mRNA [NM_000784]	7,801	5,61	2,19
COX4I2	Homo sapiens cytochrome c oxidase subunit IV isoform 2 (lung) (COX4I2), nuclear gene encoding mitochondrial protein, mRNA [NM_032609]	7,017	4,834	2,18
PLXNA4A	Homo sapiens mRNA for KIAA1550 protein, partial cds. [AB046770]	9,42	7,237	2,18

Supplemental Table 1
Morandi et al

SORBS2	Homo sapiens sorbin and SH3 domain containing 2 (SORBS2), transcript variant 2, mRNA [NM_021069]	10,215	8,037	2,18
THC2500892	ALU1_HUMAN (P39188) Alu subfamily J sequence contamination warning entry, partial (12%) [THC2500892]	7,007	4,837	2,17
THC2504193	Q15540_HUMAN (Q15540) DCP1 protein , partial (11%) [THC2504193]	8,686	6,519	2,17
APP	Homo sapiens amyloid beta (A4) precursor protein (peptidase nexin-II, Alzheimer disease) (APP), transcript variant 1, mRNA [NM_000484]	10,79	8,625	2,17
GEM	Homo sapiens GTP binding protein overexpressed in skeletal muscle (GEM), transcript variant 1, mRNA [NM_005261]	10,29	8,128	2,16
PPP1R12B	Homo sapiens protein phosphatase 1, regulatory (inhibitor) subunit 12B (PPP1R12B), transcript variant 2, mRNA [NM_032105]	12,085	9,923	2,16
GGTL3	Homo sapiens gamma-glutamyltransferase-like 3 (GGTL3), mRNA [NM_178026]	9,349	7,192	2,16
THC2633081	chr15:28503020-28502961	8,005	5,849	2,16
THC2756393	GIMA5_HUMAN (Q96F15) GTPase, IMAP family member 5 (Immunity-associated nucleotide 4-like 1 protein) (Immunity-associated protein 3) (IAN-5), partial (36%) [THC2756393]	6,677	4,524	2,15
PHLDA1	Homo sapiens pleckstrin homology-like domain, family A, member 1 (PHLDA1), mRNA [NM_007350]	9,473	7,32	2,15
RALGDS	Homo sapiens ral guanine nucleotide dissociation stimulator (RALGDS), transcript variant 2, mRNA [NM_001042368]	10,611	8,458	2,15
MAP7	Homo sapiens microtubule-associated protein 7 (MAP7), mRNA [NM_003980]	9,03	6,88	2,15
CXCR7	Homo sapiens chemokine (C-X-C motif) receptor 7 (CXCR7), transcript variant 1, mRNA [NM_001047841]	10,566	8,418	2,15
LGI3	Homo sapiens leucine-rich repeat LGI family, member 3 (LGI3), mRNA [NM_139278]	9,7	7,552	2,15
OPN4	Homo sapiens opsin 4 (melanopsin) (OPN4), transcript variant 2, mRNA [NM_001030015]	6,226	4,083	2,14
FLJ20152	Homo sapiens hypothetical protein FLJ20152 (FLJ20152), transcript variant 1, mRNA [NM_001034850]	11,874	9,745	2,13
RGS11	Homo sapiens regulator of G-protein signalling 11, mRNA (cDNA clone IMAGE:5303082), complete cds. [BC040504]	6,254	4,125	2,13
CNKSR3	Homo sapiens CNKSR family member 3 (CNKSR3), mRNA [NM_173515]	6,401	4,275	2,13
PLLP	Homo sapiens transmembrane 4 superfamily member 11 (plasmolipin) (TM4SF11), mRNA [NM_015993]	6,548	4,423	2,13
NOTCH4	Homo sapiens Notch homolog 4 (Drosophila) (NOTCH4), mRNA [NM_004557]	11,006	8,882	2,12
EMID1	Homo sapiens EMI domain containing 1 (EMID1), mRNA [NM_133455]	6,574	4,452	2,12
CCND2	Homo sapiens cyclin D2 (CCND2), mRNA [NM_001759]	9,894	7,774	2,12
LOC644662	PREDICTED: Homo sapiens hypothetical LOC644662, transcript variant 2 (LOC644662), mRNA [XM_933903]	7,088	4,973	2,11
ASTN2	Homo sapiens astrotactin 2 (ASTN2), transcript variant 1, mRNA [NM_014010]	12,229	10,119	2,11
ZBTB6	Homo sapiens zinc finger and BTB domain containing 6 (ZBTB6), mRNA [NM_006626]	7,253	5,143	2,11
AEBP1	Homo sapiens AE binding protein 1 (AEBP1), mRNA [NM_001129]	12,926	10,817	2,11
C20orf112	Uncharacterized protein C20orf112. [Source:Uniprot/SWISSPROT;Acc:Q96MY1] [ENST00000359676]	8,248	6,141	2,11
NRCAM	Homo sapiens neuronal cell adhesion molecule (NRCAM), transcript variant 1, mRNA [NM_001037132]	11,518	9,414	2,10
BC070125	Homo sapiens cDNA clone MGC:88103 IMAGE:4693019, complete cds. [BC070125]	6,286	4,184	2,10
MOBKL2B	Homo sapiens MOB1, Mps One Binder kinase activator-like 2B (yeast) (MOBKL2B), mRNA [NM_024761]	7,895	5,796	2,10
PPFIBP1	Homo sapiens PTPRF interacting protein, binding protein 1 (liprin beta 1) (PPFIBP1), transcript variant 1, mRNA [NM_003622]	8,335	6,237	2,10
FZR1	Homo sapiens fizzy/cell division cycle 20 related 1 (Drosophila) (FZR1), mRNA [NM_016263]	8,993	6,902	2,09

Supplemental Table 1
Morandi et al

AK124281	Homo sapiens cDNA FLJ42287 fis, clone TLIVE2005866. [AK124281]	6,421	4,329	2,09
CPEB3	Homo sapiens cytoplasmic polyadenylation element binding protein 3 (CPEB3), mRNA [NM_014912]	8,423	6,333	2,09
ENST0000038349(	chr18:5881958-5881899	6,251	4,163	2,09
NRXN3	Homo sapiens neurexin 3 (NRXN3), transcript variant alpha, mRNA [NM_004796]	9,981	7,895	2,09
TTYH2	Homo sapiens tweety homolog 2 (Drosophila) (TTYH2), transcript variant 1, mRNA [NM_032646]	6,279	4,194	2,09
BG193790	RST12926 Athersys RAGE Library Homo sapiens cDNA, mRNA sequence [BG193790]	5,895	3,809	2,09
THC2686967	chr17:69863473-69863532	6,211	4,126	2,09
EFNA1	Homo sapiens ephrin-A1 (EFNA1), transcript variant 1, mRNA [NM_004428]	9,343	7,258	2,08
OLFM1	Homo sapiens olfactomedin 1 (OLFM1), transcript variant 1, mRNA [NM_014279]	11,309	9,225	2,08
AK021467	Homo sapiens cDNA FLJ11405 fis, clone HEMBA1000769. [AK021467]	7,079	5,002	2,08
RGAG4	Homo sapiens retrotransposon gag domain containing 4 (RGAG4), mRNA [NM_001024455]	10,002	7,925	2,08
ITGA1	Homo sapiens integrin, alpha 1 (ITGA1), mRNA [NM_181501]	9,769	7,694	2,08
ARHGAP26	Homo sapiens Rho GTPase activating protein 26 (ARHGAP26), mRNA [NM_015071]	7,858	5,785	2,07
STAB2	Homo sapiens stabilin 2 (STAB2), mRNA [NM_017564]	5,982	3,909	2,07
IGF2AS	Homo sapiens insulin-like growth factor 2 antisense (IGF2AS), mRNA [NM_016412]	7,761	5,694	2,07
EXOC3L	Homo sapiens exocyst complex component 3-like (EXOC3L), mRNA [NM_178516]	8,085	6,018	2,07
PRKAR1B	Homo sapiens protein kinase, cAMP-dependent, regulatory, type I, beta (PRKAR1B), mRNA [NM_002735]	9,555	7,489	2,07
C22orf9	Homo sapiens chromosome 22 open reading frame 9 (C22orf9), transcript variant 1, mRNA [NM_015264]	9,37	7,313	2,06
AK021980	Homo sapiens cDNA FLJ11918 fis, clone HEMBB1000272. [AK021980]	13,107	11,051	2,06
GUCY1B3	Homo sapiens guanylate cyclase 1, soluble, beta 3 (GUCY1B3), mRNA [NM_000857]	8,608	6,552	2,06
AK091028	Homo sapiens cDNA FLJ33709 fis, clone BRAWH2007890. [AK091028]	6,86	4,806	2,05
GSN	Homo sapiens gelsolin (amyloidosis, Finnish type) (GSN), transcript variant 2, mRNA [NM_198252]	13,42	11,371	2,05
RNASEL	Homo sapiens ribonuclease L (2',5'-oligoisoadenylate synthetase-dependent) (RNASEL), mRNA [NM_021133]	9,379	7,329	2,05
MYOG	Homo sapiens myogenin (myogenic factor 4) (MYOG), mRNA [NM_002479]	5,797	3,749	2,05
ZFYVE9	Homo sapiens zinc finger, FYVE domain containing 9 (ZFYVE9), transcript variant 3, mRNA [NM_004799]	10,457	8,409	2,05
ARVCF	Homo sapiens armadillo repeat gene deletes in velocardiofacial syndrome (ARVCF), mRNA [NM_001670]	10,737	8,69	2,05
SERPING1	Homo sapiens serpin peptidase inhibitor, clade G (C1 inhibitor), member 1, (angioedema, hereditary) (SERPING1), transcript variant 1, mRNA [NM_000062]	8,817	6,771	2,05
C5orf4	Homo sapiens chromosome 5 open reading frame 4 (C5orf4), transcript variant 2, mRNA [NM_032385]	10,505	8,464	2,04
THC2666687	ALU1_HUMAN (P39188) Alu subfamily J sequence contamination warning entry, partial (4%) [THC2666687]	8,759	6,72	2,04
ENST0000032590(	Homo sapiens hypothetical protein MGC39606, mRNA (cDNA clone MGC:39606 IMAGE:5260968), complete cds. [BC051704]	10,95	8,909	2,04
PPAP2A	Homo sapiens phosphatidic acid phosphatase type 2A (PPAP2A), transcript variant 2, mRNA [NM_176895]	11,897	9,859	2,04
THC2709260	chr2:152361700-152361641	9,768	7,733	2,04
HRH1	Homo sapiens histamine receptor H1 (HRH1), mRNA [NM_000861]	6,898	4,866	2,03
KRTAP6-3	Homo sapiens keratin associated protein 6-3 (KRTAP6-3), mRNA [NM_181605]	6,818	4,786	2,03

GPR20	Homo sapiens G protein-coupled receptor 20 (GPR20), mRNA [NM_005293]	7,714	5,684	2,03
-	chr11:057138384-057138441	10,491	8,461	2,03
TBXA2R	Homo sapiens thromboxane A2 receptor (TBXA2R), transcript variant 2, mRNA [NM_001060]	8,612	6,587	2,03
AK023338	Homo sapiens cDNA FLJ13276 fis, clone OVARC1001040. [AK023338]	7,435	5,414	2,02
WNT4	Homo sapiens wingless-type MMTV integration site family, member 4 (WNT4), mRNA [NM_030761]	8,75	6,733	2,02
NLGN3	Homo sapiens neuroligin 3 (NLGN3), mRNA [NM_018977]	7,965	5,953	2,01
GIPC3	Homo sapiens mRNA; cDNA DKFZp686J1198 (from clone DKFZp686J1198). [BX648927]	5,654	3,644	2,01
CCDC71	Homo sapiens coiled-coil domain containing 71 (CCDC71), mRNA [NM_022903]	8,05	6,042	2,01
C9orf109	Homo sapiens cDNA FLJ45609 fis, clone BRTHA3023590. [AK127516]	6,973	4,97	2,00
CCR5	Homo sapiens chemokine (C-C motif) receptor 5 (CCR5), mRNA [NM_000579]	8,416	6,415	2,00
CR595668	full-length cDNA clone CS0DB003YC18 of Neuroblastoma Cot 10-normalized of Homo sapiens (human). [CR595668]	8,125	6,129	2,00
KIAA0367	Homo sapiens KIAA0367 (KIAA0367), mRNA [NM_015225]	12,059	10,063	2,00
BY798288	BY798288 Homo sapiens eye Homo sapiens cDNA clone HE3347.seq 5', mRNA sequence [BY798288]	8,299	6,306	1,99
ASB9	Homo sapiens ankyrin repeat and SOCS box-containing 9 (ASB9), transcript variant 1, mRNA [NM_001031739]	6,005	4,019	1,99
LOH11CR2A	Homo sapiens loss of heterozygosity, 11, chromosomal region 2, gene A (LOH11CR2A), transcript variant 1, mRNA [NM_014622]	7,977	5,995	1,98
FAM43A	Homo sapiens family with sequence similarity 43, member A (FAM43A), mRNA [NM_153690]	8,232	6,254	1,98
AK098511	Homo sapiens cDNA FLJ25645 fis, clone SYN00113. [AK098511]	7,556	5,58	1,98
BC104421	Homo sapiens cDNA clone IMAGE:40004940. [BC104421]	6,482	4,507	1,97
TTC17	Homo sapiens tetratricopeptide repeat domain 17, mRNA (cDNA clone IMAGE:5298645), complete cds. [BC041893]	9,921	7,947	1,97
ENST00000355748	Endogenous retrovirus H D1 leader region/integrase-derived ORF1, ORF2, and putative envelope protein (Endogenous retrovirus H protease/integrase-derived ORF1, ORF2, and putative envelope protein). [Source:Uniprot/SPTREMBL;Acc:O00627] [ENST00000355748]	7,405	5,434	1,97
PPP2R2C	Homo sapiens protein phosphatase 2 (formerly 2A), regulatory subunit B (PR 52), gamma isoform (PPP2R2C), transcript variant 1, mRNA [NM_020416]	7,018	5,049	1,97
AIFM2	Homo sapiens apoptosis-inducing factor, mitochondrion-associated, 2 (AIFM2), mRNA [NM_032797]	9,518	7,549	1,97
GPR162	Homo sapiens G protein-coupled receptor 162 (GPR162), transcript variant A-2, mRNA [NM_019858]	8,901	6,932	1,97
ITGA9	Homo sapiens integrin, alpha 9 (ITGA9), mRNA [NM_002207]	7,473	5,505	1,97
OPN1LW	Homo sapiens opsin 1 (cone pigments), long-wave-sensitive (color blindness, protan) (OPN1LW), mRNA [NM_020061]	6,407	4,44	1,97
F8	Homo sapiens coagulation factor VIII, procoagulant component (hemophilia A) (F8), transcript variant 1, mRNA [NM_000132]	6,103	4,138	1,97
PTCH2	Homo sapiens patched homolog 2 (Drosophila) (PTCH2), mRNA [NM_003738]	6,619	4,657	1,96
TXLNB	Homo sapiens taxilin beta (TXLNB), mRNA [NM_153235]	5,741	3,782	1,96
PDZK1	Homo sapiens PDZ domain containing 1 (PDZK1), mRNA [NM_002614]	6,682	4,726	1,96
OR5H1	Homo sapiens olfactory receptor, family 5, subfamily H, member 1 (OR5H1), mRNA [NM_001005338]	6,347	4,392	1,96
CTSC	Homo sapiens cathepsin C (CTSC), transcript variant 2, mRNA [NM_148170]	9,783	7,831	1,95
SLC22A17	Homo sapiens solute carrier family 22 (organic cation transporter), member 17 (SLC22A17), transcript variant 2, mRNA [NM_016609]	10,942	8,995	1,95
THC2515651	chr3:131321861-131321920	8,597	6,653	1,94
HSU79275	Human clone 23947 mRNA, partial cds. [U79275]	7,529	5,587	1,94

Supplemental Table 1
Morandi et al

-	chrX:018925300-018925252	6,51	4,568	1,94
SPTB	Homo sapiens spectrin, beta, erythrocytic (includes spherocytosis, clinical type I) (SPTB), transcript variant 1, mRNA [NM_001024858]	6,497	4,556	1,94
-	chr18:073390284-073390343	6,425	4,483	1,94
PGM2L1	Homo sapiens phosphoglucomutase 2-like 1 (PGM2L1), mRNA [NM_173582]	10,843	8,904	1,94
-	chr2:242076896-242076837	7,456	5,517	1,94
AI682237	AI682237 wa71f04.x1 Soares_NFL_T_GBC_S1 Homo sapiens cDNA clone IMAGE:2301631 3', mRNA sequence [AI682237]	6,065	4,128	1,94
ENST00000367087	Fc alpha/mu receptor (Fragment). [Source:Uniprot/SPTREMBL;Acc:Q8WWV5] [ENST00000367087]	5,822	3,886	1,94
CACNG3	Homo sapiens calcium channel, voltage-dependent, gamma subunit 3 (CACNG3), mRNA [NM_006539]	5,966	4,03	1,94
CRB3	Homo sapiens crumbs homolog 3 (Drosophila) (CRB3), transcript variant 3, mRNA [NM_174881]	5,933	3,996	1,94
FLJ38984	Homo sapiens hypothetical protein FLJ38984 (FLJ38984), mRNA [NM_152374]	8,702	6,765	1,94
NOPE	Homo sapiens likely ortholog of mouse neighbor of Punc E11 (NOPE), mRNA [NM_020962]	5,65	3,717	1,93
PIK3R1	Homo sapiens phosphoinositide-3-kinase, regulatory subunit 1 (p85 alpha) (PIK3R1), transcript variant 1, mRNA [NM_181523]	10,893	8,962	1,93
ENST0000038192	Homo sapiens mucin 4 (MUC4) mRNA, partial cds. [AF177925]	6,538	4,61	1,93
CSPG5	Homo sapiens chondroitin sulfate proteoglycan 5 (neuroglycan C) (CSPG5), mRNA [NM_006574]	7,159	5,233	1,93
GSTM3	Homo sapiens glutathione S-transferase M3 (brain) (GSTM3), mRNA [NM_000849]	7,253	5,328	1,93
IL32	Homo sapiens interleukin 32 (IL32), transcript variant 1, mRNA [NM_001012631]	9,662	7,738	1,92
LOC400451	Homo sapiens hypothetical gene supported by AK075564; BC060873 (LOC400451), mRNA [NM_207446]	11,482	9,559	1,92
NXPH3	Homo sapiens neurexophilin 3 (NXPH3), mRNA [NM_007225]	5,836	3,918	1,92
C11orf72	Homo sapiens chromosome 11 open reading frame 72 (C11orf72), mRNA [NM_173578]	5,9	3,984	1,92
AF052115	Homo sapiens clone 23688 mRNA sequence. [AF052115]	7,015	5,103	1,91
DNAJB2	Homo sapiens DnaJ (Hsp40) homolog, subfamily B, member 2 (DNAJB2), transcript variant 1, mRNA [NM_001039550]	9,215	7,304	1,91
STARD8	Homo sapiens START domain containing 8 (STARD8), mRNA [NM_014725]	7,638	5,728	1,91
MDFI	Homo sapiens MyoD family inhibitor (MDFI), mRNA [NM_005586]	7,791	5,882	1,91
-	chr20:058977227-058977286	6,333	4,424	1,91
HSPA1A	Homo sapiens heat shock 70kDa protein 1A (HSPA1A), mRNA [NM_005345]	12,387	10,479	1,91
AK022252	Homo sapiens cDNA FLJ12190 fis, clone MAMMA1000842. [AK022252]	7,431	5,524	1,91
PHYHD1	Homo sapiens phytanoyl-CoA dioxygenase domain containing 1 (PHYHD1), mRNA [NM_174933]	8,343	6,438	1,91
APOL3	Homo sapiens apolipoprotein L, 3 (APOL3), transcript variant beta/a, mRNA [NM_145641]	7,292	5,387	1,90
FLJ12993	Homo sapiens cDNA FLJ12993 fis, clone NT2RP3000197. [AK023055]	7,213	5,31	1,90
APOL4	Homo sapiens apolipoprotein L, 4 (APOL4), transcript variant a, mRNA [NM_030643]	6,048	4,148	1,90
LPPR4	Homo sapiens plasticity related gene 1 (LPPR4), mRNA [NM_014839]	11,169	9,269	1,90
TMED9	Homo sapiens transmembrane emp24 protein transport domain containing 9 (TMED9), mRNA [NM_017510]	11,149	9,251	1,90
BTN3A2	Homo sapiens butyrophilin, subfamily 3, member A2 (BTN3A2), mRNA [NM_007047]	9,217	7,32	1,90
MOBKL2C	Homo sapiens MOB1, Mps One Binder kinase activator-like 2C (yeast) (MOBKL2C), transcript variant 1, mRNA [NM_145279]	9,062	7,167	1,90
THC2765833	chr1:227198131-227198072	5,8	3,907	1,89
SGCA	Homo sapiens sarcoglycan, alpha (50kDa dystrophin-associated glycoprotein) (SGCA), mRNA [NM_000023]	7,118	5,226	1,89

Supplemental Table 1
Morandi et al

BC014346	Homo sapiens, clone IMAGE:4042988, mRNA, partial cds. [BC014346]	5,97	4,078	1,89
EVA1	Homo sapiens epithelial V-like antigen 1 (EVA1), transcript variant 2, mRNA [NM_144765]	6,539	4,648	1,89
CD47	Homo sapiens CD47 molecule (CD47), transcript variant 1, mRNA [NM_001777]	8,811	6,921	1,89
AK093729	Homo sapiens cDNA FLJ36410 fis, clone THYMU2010637. [AK093729]	11,642	9,752	1,89
ADAMTS4	Homo sapiens ADAM metallopeptidase with thrombospondin type 1 motif, 4 (ADAMTS4), mRNA [NM_005099]	10,572	8,683	1,89
CBX7	Homo sapiens chromobox homolog 7 (CBX7), mRNA [NM_175709]	11,009	9,122	1,89
NES	Homo sapiens nestin (NES), mRNA [NM_006617]	12,527	10,641	1,89
ENST00000327574	Pyridoxal kinase (EC 2.7.1.35) (Pyridoxine kinase). [Source:Uniprot/SWISSPROT;Acc:O00764] [ENST00000327574]	5,697	3,812	1,89
HDAC11	Homo sapiens histone deacetylase 11 (HDAC11), mRNA [NM_024827]	7,104	5,222	1,88
TUBB4	Homo sapiens tubulin, beta 4 (TUBB4), mRNA [NM_006087]	6,962	5,079	1,88
-	chr19:002034517-002034576	5,351	3,469	1,88
MICALL2	Homo sapiens MICAL-like 2 (MICALL2), transcript variant 1, mRNA [NM_182924]	12,57	10,692	1,88
AK021933	Homo sapiens cDNA FLJ11871 fis, clone HEMBA1007052. [AK021933]	5,981	4,106	1,88
EPHB4	Homo sapiens EPH receptor B4 (EPHB4), mRNA [NM_004444]	6,261	4,388	1,87
PRKAG1	Homo sapiens protein kinase, AMP-activated, gamma 1 non-catalytic subunit (PRKAG1), transcript variant 2, mRNA [NM_212461]	8,577	6,705	1,87
IL17RE	Homo sapiens interleukin 17 receptor E (IL17RE), transcript variant 5, mRNA [NM_153483]	7,474	5,604	1,87
THC2530075	chr1:61697248-61697189	6,464	4,595	1,87
KIAA1614	Homo sapiens mRNA for KIAA1614 protein, partial cds. [AB046834]	5,979	4,11	1,87
TNFSF4	Homo sapiens tumor necrosis factor (ligand) superfamily, member 4 (tax-transcriptionally activated glycoprotein 1, 34kDa) (TNFSF4), mRNA [NM_003326]	6,312	4,443	1,87
KIAA0574	Homo sapiens mRNA for KIAA0574 protein, partial cds. [AB011146]	7,141	5,274	1,87
THC2558594	U92819 unnamed HERV-H protein {Homo sapiens} (exp=-1; wgp=0; cg=0), partial (25%) [THC2558594]	6,686	4,819	1,87
AK090416	Homo sapiens mRNA for FLJ00318 protein. [AK090416]	6,896	5,031	1,87
CCDC128	Homo sapiens coiled-coil domain containing 128 (CCDC128), mRNA [NM_152994]	9,846	7,984	1,86
DRD2	Homo sapiens dopamine receptor D2 (DRD2), transcript variant 1, mRNA [NM_000795]	6,73	4,87	1,86
CLU	Homo sapiens clusterin (CLU), transcript variant 2, mRNA [NM_203339]	13,983	12,127	1,86
DKFZp434K191	Homo sapiens hypothetical protein DKFZp434K191 (DKFZp434K191), mRNA [NM_001029950]	8,053	6,2	1,85
AMIGO1	Amphoterin-induced protein 1 precursor (AMIGO-1) (Alivin-2). [Source:Uniprot/SWISSPROT;Acc:Q86WK6] [ENST00000369864]	7,54	5,687	1,85
HSA251708	Homo sapiens partial mRNA for putative microtubule-binding protein. [AJ251708]	6,015	4,162	1,85
THC2531058	Q47K95_THEFY (Q47K95) Single-strand binding protein, partial (6%) [THC2531058]	7,119	5,267	1,85
TMEM19	Homo sapiens transmembrane protein 19 (TMEM19), mRNA [NM_018279]	6,162	4,311	1,85
ZNF337	Homo sapiens zinc finger protein 337 (ZNF337), mRNA [NM_015655]	9,5	7,65	1,85
BM703463	UI-E-CL1-afe-e-20-0-UI.r1 UI-E-CL1 Homo sapiens cDNA clone UI-E-CL1-afe-e-20-0-UI 5', mRNA sequence [BM703463]	6,474	4,623	1,85
FAM70B	Homo sapiens family with sequence similarity 70, member B (FAM70B), mRNA [NM_182614]	7,13	5,282	1,85
SNPH	Homo sapiens syntaphilin (SNPH), mRNA [NM_014723]	10,31	8,464	1,85
LOC284570	Homo sapiens, clone IMAGE:4941949, mRNA. [BC040156]	7,574	5,729	1,85
-	chr7:022653000-022652941	7,017	5,172	1,85

Supplemental Table 1
Morandi et al

PRKCBP1	Homo sapiens mRNA; cDNA DKFZp564P1772 (from clone DKFZp564P1772). [AL137703]	7,978	6,135	1,84
KRTAP13-4	Homo sapiens keratin associated protein 13-4 (KRTAP13-4), mRNA [NM_181600]	5,904	4,063	1,84
UGT2B10	Homo sapiens UDP glucuronosyltransferase 2 family, polypeptide B10 (UGT2B10), mRNA [NM_001075]	6,782	4,941	1,84
METTL7A	Homo sapiens methyltransferase like 7A (METTL7A), mRNA [NM_014033]	11,064	9,224	1,84
SFTPC	Homo sapiens surfactant, pulmonary-associated protein C, mRNA (cDNA clone MGC:14509 IMAGE:4043169), complete cds. [BC005913]	7,756	5,922	1,83
THC2739159	ALU8_HUMAN (P39195) Alu subfamily SX sequence contamination warning entry, partial (8%) [THC2739159]	10,128	8,297	1,83
SLC9A3R2	Homo sapiens solute carrier family 9 (sodium/hydrogen exchanger), member 3 regulator 2 (SLC9A3R2), mRNA [NM_004785]	10,629	8,798	1,83
MAGED4	Homo sapiens melanoma antigen family D, 4 (MAGED4), transcript variant 1, mRNA [NM_030801]	13,497	11,666	1,83
MFHAS1	Homo sapiens malignant fibrous histiocytoma amplified sequence 1 (MFHAS1), mRNA [NM_004225]	7,057	5,228	1,83
AI915259	AI915259 tx06b08.x1 NCI_CGAP_Ut4 Homo sapiens cDNA clone IMAGE:2268375 3' similar to gb:J03278 BETA PLATELET-DERIVED GROWTH FACTOR RECEPTOR PRECURSOR (HUMAN);, mRNA sequence [AI915259]	6,622	4,794	1,83
C1orf90	Homo sapiens chromosome 1 open reading frame 90 (C1orf90), mRNA [NM_032648]	8,291	6,463	1,83
-	chr7:029922363-029922422	8,337	6,509	1,83
GPR37L1	Homo sapiens G protein-coupled receptor 37 like 1 (GPR37L1), mRNA [NM_004767]	6,061	4,238	1,82
MMP28	Homo sapiens matrix metallopeptidase 28 (MMP28), transcript variant 1, mRNA [NM_024302]	6,247	4,431	1,82
-	chr8:008049184-008049125	7,888	6,072	1,82
TTC33	Homo sapiens tetratricopeptide repeat domain 33 (TTC33), mRNA [NM_012382]	6,77	4,956	1,81
SHANK3	SH3 and multiple ankyrin repeat domains protein 3 (Shank3) (Proline-rich synapse-associated protein 2) (ProSAP2) (Fragment). [Source:Uniprot/SWISSPROT;Acc:Q9BYB0] [ENST00000262795]	10,657	8,845	1,81
TMEPAI	Homo sapiens transmembrane, prostate androgen induced RNA (TMEPAI), transcript variant 1, mRNA [NM_020182]	12,329	10,519	1,81
THC2749949	Q292V3_DROPS (Q292V3) GA11845-PA (Fragment), partial (10%) [THC2749949]	5,864	4,057	1,81
KIAA0664	Homo sapiens KIAA0664 (KIAA0664), mRNA [NM_015229]	9,861	8,058	1,80
LAX1	Homo sapiens cDNA FLJ20340 fis, clone HEP12374. [AK000347]	6,538	4,736	1,80
CLIC2	Homo sapiens chloride intracellular channel 2 (CLIC2), mRNA [NM_001289]	6,059	4,257	1,80
DNPEP	Aspartyl aminopeptidase (EC 3.4.11.21). [Source:Uniprot/SWISSPROT;Acc:Q9ULA0] [ENST00000373989]	7,415	5,614	1,80
CR992331	CR992331 RZPD no.9016 Homo sapiens cDNA clone RZPDp9016A0141 5', mRNA sequence [CR992331]	7,373	5,573	1,80
ENDOD1	Endonuclease domain-containing 1 protein precursor (EC 3.1.30.-). [Source:Uniprot/SWISSPROT;Acc:O94919] [ENST00000278505]	10,566	8,767	1,80
ADCY6	Homo sapiens adenylate cyclase 6 (ADCY6), transcript variant 1, mRNA [NM_015270]	9,981	8,182	1,80
RASGEF1C	Homo sapiens RasGEF domain family, member 1C (RASGEF1C), transcript variant 1, mRNA [NM_175062]	6,662	4,863	1,80
KCNMA1	Homo sapiens potassium large conductance calcium-activated channel, subfamily M, alpha member 1 (KCNMA1), transcript variant 1, mRNA [NM_001014797]	11,37	9,581	1,79
APOL2	Homo sapiens apolipoprotein L, 2 (APOL2), transcript variant beta, mRNA [NM_145637]	11,779	9,992	1,79
ELA2B	Homo sapiens elastase 2B (ELA2B), mRNA [NM_015849]	5,557	3,77	1,79

Supplemental Table 1
Morandi et al

PPFIA3	Homo sapiens protein tyrosine phosphatase, receptor type, f polypeptide (PTPRF), interacting protein (liprin), alpha 3 (PPFIA3), mRNA [NM_003660]	9,186	7,399	1,79
LYNX1	Homo sapiens Ly6/neurotoxin 1 (LYNX1), transcript variant 3, mRNA [NM_177457]	10,433	8,647	1,79
U77706	Human laminin alpha 4 chain (LAMA4*-1) mRNA, complete cds. [U77706]	6,792	5,01	1,78
THC2671230	GCU51450 IgNARC {Ginglymostoma cirratum} (exp=-1; wgp=0; cg=0), partial (3%) [THC2671230]	7,969	6,189	1,78
LZTS1	Homo sapiens leucine zipper, putative tumor suppressor 1 (LZTS1), mRNA [NM_021020]	11,858	10,077	1,78
STX6	Homo sapiens cDNA FLJ32095 fis, clone OCBBF2000998. [AK056657]	8,38	6,602	1,78
CDKL2	Homo sapiens cyclin-dependent kinase-like 2 (CDC2-related kinase) (CDKL2), mRNA [NM_003948]	8,726	6,951	1,78
COX6B2	Homo sapiens cytochrome c oxidase subunit VIb polypeptide 2 (testis) (COX6B2), mRNA [NM_144613]	6,689	4,916	1,77
PGBD5	Homo sapiens piggyBac transposable element derived 5 (PGBD5), mRNA [NM_024554]	8,49	6,717	1,77
-	chr12:003141984-003142043	7,99	6,224	1,77
ACVR1B	Homo sapiens activin A receptor, type IB (ACVR1B), transcript variant 1, mRNA [NM_004302]	8,745	6,979	1,77
ZBTB20	Homo sapiens zinc finger and BTB domain containing 20 (ZBTB20), mRNA [NM_015642]	10,905	9,14	1,76
AX781433	Sequence 16 from Patent EP1321519. [AX781433]	5,591	3,827	1,76
PRRT1	Homo sapiens proline-rich transmembrane protein 1 (PRRT1), mRNA [NM_030651]	6,556	4,795	1,76
BC042853	Homo sapiens cDNA clone IMAGE:5271371. [BC042853]	7,384	5,623	1,76
GPR126	Homo sapiens G protein-coupled receptor 126 (GPR126), transcript variant b1, mRNA [NM_198569]	6,042	4,283	1,76
THC2486290	chr3:11822155-11822096	7,032	5,274	1,76
AY010113	Homo sapiens unknown mRNA sequence. [AY010113]	6,011	4,254	1,76
TOM1L1	full-length cDNA clone CS0DD005YN08 of Neuroblastoma Cot 50-normalized of Homo sapiens (human). [CR593166]	6,996	5,243	1,75
ENST0000037867[illegible]	apical protein 2 [Source:RefSeq_peptide;Acc:NP_597713] [ENST00000378679]	7,872	6,124	1,75
AK055838	Homo sapiens cDNA FLJ31276 fis, clone KIDNE2006376, weakly similar to GAMMA-GLUTAMYLTRANSPEPTIDASE 1 PRECURSOR (EC 2.3.2.2). [AK055838]	6,587	4,839	1,75
AL109704	Homo sapiens mRNA full length insert cDNA clone EUROIMAGE 897021. [AL109704]	6,735	4,987	1,75
ATP6V1H	Homo sapiens ATPase, H+ transporting, lysosomal 50/57kDa, V1 subunit H (ATP6V1H), transcript variant 1, mRNA [NM_015941]	8,2	6,454	1,75
KIF3C	Homo sapiens kinesin family member 3C (KIF3C), mRNA [NM_002254]	8,542	6,795	1,75
THC2779700	Q4RVR0_TETNG (Q4RVR0) Chromosome 9 SCAF14991, whole genome shotgun sequence, partial (8%) [THC2784715]	6,219	4,474	1,74
-	chr15:059180312-059180253	6,793	5,052	1,74
AL080072	Homo sapiens mRNA; cDNA DKFZp564M0616 (from clone DKFZp564M0616). [AL080072]	6,521	4,782	1,74
AK129982	Homo sapiens cDNA FLJ26472 fis, clone KDN04506. [AK129982]	8,033	6,296	1,74
AF119842	Homo sapiens PRO1048 mRNA, complete cds. [AF119842]	5,692	3,958	1,74
MUC3A	Homo sapiens mucin (MUC-3) mRNA, partial cds. [M55405]	6,873	5,141	1,73
TAF3	Transcription initiation factor TFIID subunit 3 (TBP-associated factor 3) (Transcription initiation factor TFIID 140 kDa subunit) (140 kDa TATA box-binding protein-associated factor) (TAF140) (TAFII140). [Source:Uniprot/SWISSPROT;Acc:Q5VWG9]...	7,954	6,221	1,73
HIVEP2	Homo sapiens human immunodeficiency virus type I enhancer binding protein 2 (HIVEP2), mRNA [NM_006734]	12,045	10,313	1,73
ENST0000036052[illegible]	Seven transmembrane helix receptor. [Source:Uniprot/SPTREMBL;Acc:Q8NHA3] [ENST00000360523]	7,137	5,406	1,73
SCAMP4	Homo sapiens cDNA FLJ90105 fis, clone HEMBA1006357, highly similar to Secretory carrier membrane protein 4. [AK074586]	6,074	4,346	1,73
PLG	Homo sapiens plasminogen (PLG), mRNA [NM_000301]	6,64	4,912	1,73

Supplemental Table 1
Morandi et al

GUCA1A	Homo sapiens guanylate cyclase activator 1A (retina) (GUCA1A), mRNA [NM_000409]	5,885	4,158	1,73
THC2503819	BC010854 amyloid beta A4 precursor protein-binding, family B, member 1, isoform delta E9 {Homo sapiens} (exp=-1; wgp=0; cg=0), partial (22%) [THC2503819]	10,464	8,741	1,72
C19orf4	Homo sapiens chromosome 19 open reading frame 4 (C19orf4), mRNA [NM_012109]	10,271	8,549	1,72
SLC45A1	Proton-associated sugar transporter A (PAST-A) (Solute carrier family 45 member 1) (Deleted in neuroblastoma 5 protein) (DNb-5). [Source:Uniprot/SWISSPROT;Acc:Q9Y2W3] [ENST00000377479]	6,102	4,381	1,72
KIAA1155	Homo sapiens cDNA FLJ37016 fis, clone BRACE2010632. [AK094335]	7,651	5,93	1,72
SPRYD3	Homo sapiens SPRY domain containing 3 (SPRYD3), mRNA [NM_032840]	10,056	8,337	1,72
MYH14	Homo sapiens myosin, heavy chain 14 (MYH14), transcript variant 1, mRNA [NM_001077186]	7,373	5,658	1,72
FLJ20433	Homo sapiens hypothetical protein FLJ20433 (FLJ20433), mRNA [NM_017820]	7,301	5,589	1,71
PANK2	Homo sapiens mRNA; cDNA DKFZp547J0513 (from clone DKFZp547J0513). [AL713654]	6,111	4,4	1,71
AK027610	Homo sapiens cDNA FLJ14704 fis, clone NT2RP3000526. [AK027610]	6,467	4,757	1,71
MMP23B	Homo sapiens matrix metallopeptidase 23B (MMP23B), mRNA [NM_006983]	8,096	6,386	1,71
-	chr9:035845887-035845946	8,31	6,602	1,71
-	chr12:008259505-008259564	8,489	6,781	1,71
TAAR2	Homo sapiens trace amine associated receptor 2 (TAAR2), transcript variant 1, mRNA [NM_001033080]	5,715	4,009	1,71
AF090926	Homo sapiens clone HQ0456 PRO0456 mRNA, complete cds. [AF090926]	6,346	4,641	1,71
ZNF182	Homo sapiens zinc finger protein 182 (ZNF182), transcript variant 1, mRNA [NM_006962]	10,094	8,39	1,71
ENST0000029941	Q7D724_MYCTU (Q7D724) PE_PGRS family protein (PE-PGRS FAMILY PROTEIN), partial (3%) [THC2672762]	5,586	3,882	1,70
LAMP1	Homo sapiens lysosomal-associated membrane protein 1 (LAMP1), mRNA [NM_005561]	9,506	7,804	1,70
AK024315	Homo sapiens cDNA FLJ14253 fis, clone OVARC1001376. [AK024315]	6,305	4,605	1,70
THC2513403	HUMBCAA Br-cadherin {Homo sapiens} (exp=-1; wgp=0; cg=0), partial (13%) [THC2513403]	9,367	7,668	1,70
LOC91461	Homo sapiens hypothetical protein BC007901 (LOC91461), mRNA [NM_138370]	10,571	8,873	1,70
APOLD1	Homo sapiens apolipoprotein L domain containing 1 (APOLD1), mRNA [NM_030817]	10,745	9,05	1,70
KLK2	Homo sapiens kallikrein-related peptidase 2 (KLK2), transcript variant 1, mRNA [NM_005551]	5,762	4,068	1,69
PMP22	Homo sapiens peripheral myelin protein 22 (PMP22), transcript variant 1, mRNA [NM_000304]	14,474	12,781	1,69
TRIM2	Homo sapiens tripartite motif-containing 2 (TRIM2), mRNA [NM_015271]	11,046	9,353	1,69
-	chr10:111901124-111901065	5,91	4,218	1,69
SLC6A12	Homo sapiens solute carrier family 6 (neurotransmitter transporter, betaine/GABA), member 12 (SLC6A12), mRNA [NM_003044]	6,848	5,157	1,69
FTCD	Homo sapiens formiminotransferase cyclodeaminase (FTCD), transcript variant A, mRNA [NM_206965]	7,489	5,799	1,69
CALM1	Homo sapiens calmodulin 1 (phosphorylase kinase, delta) (CALM1), mRNA [NM_006888]	11,776	10,089	1,69
BSPRY	Homo sapiens B-box and SPRY domain containing (BSPRY), mRNA [NM_017688]	6,372	4,686	1,69
IGF2R	Homo sapiens insulin-like growth factor 2 receptor (IGF2R), mRNA [NM_000876]	10,267	8,583	1,69
GPLD1	Homo sapiens glycosylphosphatidylinositol specific phospholipase D1 (GPLD1), transcript variant 1, mRNA [NM_001503]	6,148	4,464	1,68

Supplemental Table 1
Morandi et al

LOC150763	Homo sapiens hypothetical protein LOC150763 (LOC150763), mRNA [NM_207328]	9,941	8,258	1,68
PSEN2	Homo sapiens presenilin 2 (Alzheimer disease 4) (PSEN2), transcript variant 2, mRNA [NM_012486]	7,472	5,79	1,68
ZNF282	Homo sapiens zinc finger protein 282 (ZNF282), mRNA [NM_003575]	7,261	5,579	1,68
ACAD10	Homo sapiens acyl-Coenzyme A dehydrogenase family, member 10 (ACAD10), mRNA [NM_025247]	8,179	6,501	1,68
STARD9	StAR-related lipid transfer protein 9 (StARD9) (START domain-containing protein 9) (Fragment). [Source:Uniprot/SWISSPROT;Acc:Q9P2P6] [ENST00000290607]	7,32	5,646	1,68
THC2647765	chr10:105212189-105212248	6,195	4,52	1,68
EDG1	Homo sapiens endothelial differentiation, sphingolipid G-protein-coupled receptor, 1 (EDG1), mRNA [NM_001400]	8,24	6,566	1,67
SUOX	Homo sapiens sulfite oxidase (SUOX), nuclear gene encoding mitochondrial protein, transcript variant 1, mRNA [NM_000456]	8,644	6,97	1,67
KIAA1919	KIAA1919 (KIAA1919), mRNA [Source:RefSeq_dna;Acc:NM_153369] [ENST00000368847]	9,914	8,241	1,67
SFRS16	Homo sapiens cDNA FLJ90109 fis, clone HEMBA1006544. [AK074590]	8,112	6,441	1,67
AK125173	Homo sapiens cDNA FLJ43183 fis, clone FCBBF3018826. [AK125173]	7,386	5,72	1,67
HNRPAB	Homo sapiens heterogeneous nuclear ribonucleoprotein A/B (HNRPAB), transcript variant 2, mRNA [NM_004499]	10,239	8,574	1,67
SEMA4F	Homo sapiens sema domain, immunoglobulin domain (Ig), transmembrane domain (TM) and short cytoplasmic domain, (semaphorin) 4F (SEMA4F), mRNA [NM_004263]	9,668	8,005	1,66
ZNF652	Homo sapiens zinc finger protein 652 (ZNF652), mRNA [NM_014897]	9,308	7,645	1,66
NAP1L4	Homo sapiens nucleosome assembly protein 1-like 4 (NAP1L4), mRNA [NM_005969]	10,709	9,047	1,66
PIP5K1A	Homo sapiens phosphatidylinositol-4-phosphate 5-kinase, type I, alpha (PIP5K1A), mRNA [NM_003557]	11,23	9,571	1,66
RXFP3	Homo sapiens relaxin/insulin-like family peptide receptor 3 (RXFP3), mRNA [NM_016568]	5,401	3,745	1,66
BICD1	Homo sapiens bicaudal D homolog 1 (Drosophila) (BICD1), transcript variant 2, mRNA [NM_001003398]	8,68	7,025	1,66
IL9R	Homo sapiens interleukin 9 receptor (IL9R), transcript variant 2, mRNA [NM_176786]	6,025	4,373	1,65
BX104999	BX104999 Soares fetal liver spleen 1NFLS Homo sapiens cDNA clone IMAGp998L22392, mRNA sequence [BX104999]	7,783	6,133	1,65
NPFFR1	Homo sapiens neuropeptide FF receptor 1 (NPFFR1), mRNA [NM_022146]	5,475	3,826	1,65
RAD23B	Homo sapiens RAD23 homolog B (S. cerevisiae) (RAD23B), mRNA [NM_002874]	7,305	5,656	1,65
LOC654780	Homo sapiens cDNA FLJ38012 fis, clone CTONG2012554. [AK095331]	6,178	4,531	1,65
AK096778	Homo sapiens cDNA FLJ39459 fis, clone PROST2011439. [AK096778]	7,666	6,022	1,65
FLJ41603	Homo sapiens FLJ41603 protein (FLJ41603), mRNA [NM_001001669]	8,593	6,949	1,64
-	chr9:130434933-130434990	10,528	8,887	1,64
CLEC10A	Homo sapiens C-type lectin domain family 10, member A (CLEC10A), transcript variant 2, mRNA [NM_006344]	6,374	4,734	1,64
TMEM140	Homo sapiens transmembrane protein 140 (TMEM140), mRNA [NM_018295]	7,96	6,321	1,64
SYT9	Homo sapiens synaptotagmin IX (SYT9), mRNA [NM_175733]	5,92	4,282	1,64
APOL1	Homo sapiens apolipoprotein L, 1 (APOL1), transcript variant 2, mRNA [NM_145343]	5,687	4,052	1,64
C8orf53	Homo sapiens cDNA FLJ30717 fis, clone FCBBF2001672. [AK055279]	7,462	5,827	1,64
DOHH	Homo sapiens deoxyhypusine hydroxylase/monooxygenase (DOHH), mRNA [NM_031304]	10,634	9	1,63
AF086527	Homo sapiens full length insert cDNA clone ZE05A03. [AF086527]	5,821	4,187	1,63

OSBPL1A	Homo sapiens oxysterol binding protein-like 1A (OSBPL1A), transcript variant OSBPL1B, mRNA [NM_080597]	9,792	8,158	1,63
NDUFC2	Homo sapiens NADH dehydrogenase (ubiquinone) 1, subcomplex unknown, 2, 14.5kDa (NDUFC2), mRNA [NM_004549]	9,198	7,565	1,63
NOTCH1	Homo sapiens Notch homolog 1, translocation-associated (Drosophila) (NOTCH1), mRNA [NM_017617]	6,879	5,245	1,63
SCARA5	Homo sapiens scavenger receptor class A, member 5 (putative) (SCARA5), mRNA [NM_173833]	7,731	6,098	1,63
HOXA10	HOX C6=class I homeodomain {fragment M13, homeodomain} [human, MCF7 cells, mRNA Partial, 102 nt]. [S69027]	7,291	5,661	1,63
-	chr1:202864346-202864287	5,901	4,27	1,63
ALDH5A1	Homo sapiens aldehyde dehydrogenase 5 family, member A1 (succinate-semialdehyde dehydrogenase) (ALDH5A1), nuclear gene encoding mitochondrial protein, transcript variant 1, mRNA [NM_170740]	9,064	7,434	1,63
ASB8	Homo sapiens ankyrin repeat and SOCS box-containing 8 (ASB8), mRNA [NM_024095]	7,587	5,957	1,63
-	chr6:168982226-168982167	6,303	4,674	1,63
GNG13	Homo sapiens guanine nucleotide binding protein (G protein), gamma 13 (GNG13), mRNA [NM_016541]	6,146	4,519	1,63
PACSIN2	Homo sapiens protein kinase C and casein kinase substrate in neurons 2 (PACSIN2), mRNA [NM_007229]	10,101	8,474	1,63
DOCK9	Homo sapiens dedicator of cytokinesis 9 (DOCK9), mRNA [NM_015296]	8,875	7,249	1,63
DNAJC7	Homo sapiens DnaJ (Hsp40) homolog, subfamily C, member 7 (DNAJC7), mRNA [NM_003315]	12,309	10,683	1,63
-	chr8:012083172-012083231	8,89	7,265	1,63
DTNB	Homo sapiens dystrobrevin, beta (DTNB), transcript variant 3, mRNA [NM_033148]	6,69	5,065	1,62
HES5	Homo sapiens hairy and enhancer of split 5 (Drosophila) (HES5), mRNA [NM_001010926]	7,082	5,458	1,62
NOTCH3	Homo sapiens Notch homolog 3 (Drosophila) (NOTCH3), mRNA [NM_000435]	5,749	4,126	1,62
MYO18A	Homo sapiens myosin XVIIIA (MYO18A), transcript variant 1, mRNA [NM_078471]	9,858	8,236	1,62
CLCN1	Homo sapiens chloride channel 1, skeletal muscle (Thomsen disease, autosomal dominant) (CLCN1), mRNA [NM_000083]	5,944	4,324	1,62
THC2593103	Q6NT14_HUMAN (Q6NT14) ZNF80 protein (Fragment), partial (6%) [THC2593103]	8,58	6,961	1,62
THC2517566	chr7:5015308-5015367	7,768	6,15	1,62
BC045098	Homo sapiens, clone IMAGE:5171383, mRNA. [BC045098]	5,395	3,779	1,62
COL7A1	Homo sapiens collagen, type VII, alpha 1 (epidermolysis bullosa, dystrophic, dominant and recessive) (COL7A1), mRNA [NM_000094]	9,715	8,1	1,62
KRTAP2-1	Homo sapiens partial mRNA for keratin associated protein (KRTAP2.1A gene). [AJ296345]	6,509	4,894	1,62
FLJ10159	Homo sapiens hypothetical protein FLJ10159 (FLJ10159), mRNA [NM_018013]	10,677	9,064	1,61
INSR	Homo sapiens insulin receptor (INSR), transcript variant 1, mRNA [NM_000208]	8,514	6,903	1,61
IFNA4	Homo sapiens interferon, alpha 4 (IFNA4), mRNA [NM_021068]	5,681	4,07	1,61
LENG9	Homo sapiens leukocyte receptor cluster (LRC) member 9 (LENG9), mRNA [NM_198988]	6,217	4,606	1,61
AK091784	Homo sapiens cDNA FLJ34465 fis, clone HLUNG2003061. [AK091784]	9,73	8,123	1,61
CD2BP2	Homo sapiens mRNA for KIAA1178 protein, partial cds. [AB033004]	11,402	9,794	1,61
BC009036	Homo sapiens cDNA clone IMAGE:4155841, partial cds. [BC009036]	7,376	5,769	1,61
AP3M1	Homo sapiens adaptor-related protein complex 3, mu 1 subunit (AP3M1), transcript variant 1, mRNA [NM_207012]	7,649	6,045	1,60
AK094623	Homo sapiens cDNA FLJ37304 fis, clone BRAMY2016070. [AK094623]	9,763	8,161	1,60

Supplemental Table 1
Morandi et al

FRY	Homo sapiens furry homolog (Drosophila) (FRY), mRNA [NM_023037]	11,259	9,656	1,60
TMEM37	Homo sapiens transmembrane protein 37 (TMEM37), mRNA [NM_183240]	7,585	5,985	1,60
HSPA12A	Homo sapiens heat shock 70kDa protein 12A (HSPA12A), mRNA [NM_025015]	11,868	10,271	1,60
RGMA	Homo sapiens RGM domain family, member A (RGMA), mRNA [NM_020211]	9,449	7,853	1,60
MOSC2	Homo sapiens MOCO sulphurase C-terminal domain containing 2 (MOSC2), mRNA [NM_017898]	7,762	6,169	1,59
BF969441	602271732F1 NIH_MGC_84 Homo sapiens cDNA clone IMAGE:4360158 5', mRNA sequence [BF969441]	6,83	5,239	1,59
GPR161	Homo sapiens G protein-coupled receptor 161 (GPR161), transcript variant 2, mRNA [NM_153832]	9,669	8,077	1,59
-	chr19:034746601-034746651	7,215	5,624	1,59
POPDC2	Homo sapiens popeye domain containing 2 (POPDC2), mRNA [NM_022135]	5,73	4,144	1,59
TNRC6B	Homo sapiens trinucleotide repeat containing 6B (TNRC6B), transcript variant 1, mRNA [NM_015088]	10,606	9,019	1,59
DCAMKL2	Homo sapiens doublecortin and CaM kinase-like 2 (DCAMKL2), transcript variant 1, mRNA [NM_001040260]	9,702	8,117	1,58
GPR146	Homo sapiens G protein-coupled receptor 146 (GPR146), mRNA [NM_138445]	8,123	6,539	1,58
THC2562062	chr7:101786045-101786104	6,849	5,274	1,58
ALS2CL	Homo sapiens ALS2 C-terminal like (ALS2CL), transcript variant 1, mRNA [NM_147129]	8,132	6,558	1,57
WNT10A	Homo sapiens wingless-type MMTV integration site family, member 10A (WNT10A), mRNA [NM_025216]	7,891	6,318	1,57
PHF20	Homo sapiens PHD finger protein 20 (PHF20), mRNA [NM_016436]	9,372	7,8	1,57
AF268617	Homo sapiens POU 5 domain protein (POU5FLC12) mRNA, complete cds. [AF268617]	5,912	4,34	1,57
C21orf2	Homo sapiens chromosome 21 open reading frame 2 (C21orf2), mRNA [NM_004928]	8,83	7,259	1,57
-	chr12:122291180-122291121	5,937	4,371	1,57
AY358705	Homo sapiens clone DNA71234 HHSL751 (UNQ751) mRNA, complete cds. [AY358705]	5,701	4,136	1,57
SLC25A27	Homo sapiens solute carrier family 25, member 27 (SLC25A27), nuclear gene encoding mitochondrial protein, mRNA [NM_004277]	6,923	5,359	1,56
TNIP2	Homo sapiens TNFAIP3 interacting protein 2 (TNIP2), mRNA [NM_024309]	8,808	7,244	1,56
ENST0000035673(	chr8:144861173-144861114	5,676	4,115	1,56
HMCN2	Homo sapiens cDNA FLJ36264 fis, clone THYMU2002815, weakly similar to AXONIN-1 PRECURSOR. [AK093583]	6,191	4,631	1,56
C14orf113	Homo sapiens cDNA FLJ20034 fis, clone COL00195. [AK000041]	5,774	4,218	1,56
TLN2	Homo sapiens talin 2 (TLN2), mRNA [NM_015059]	10,251	8,695	1,56
GJA12	Homo sapiens gap junction protein, alpha 12, 47kDa (GJA12), mRNA [NM_020435]	9,366	7,812	1,56
MRPL30	Homo sapiens mitochondrial ribosomal protein L30 (MRPL30), nuclear gene encoding mitochondrial protein, transcript variant 1, mRNA [NM_145212]	6,091	4,536	1,56
THC2718406	chr1:176819413-176819354	5,804	4,25	1,56
-	chr21:031918525-031918466	10,777	9,222	1,55
PTK2	Homo sapiens PTK2 protein tyrosine kinase 2 (PTK2), transcript variant 1, mRNA [NM_153831]	9,653	8,1	1,55
-	chr3:075801154-075801213	6,407	4,855	1,55
THC2729899	chr4:83623649-83623590	10,254	8,702	1,55
BC032332	Homo sapiens cDNA clone MGC:40288 IMAGE:5169056, complete cds. [BC032332]	8,936	7,387	1,55
AK094447	Homo sapiens cDNA FLJ37128 fis, clone BRACE2022928. [AK094447]	6,153	4,602	1,55
UNC84B	Homo sapiens unc-84 homolog B (C. elegans) (UNC84B), mRNA [NM_015374]	9,129	7,579	1,55

Supplemental Table 1
Morandi et al

THC2693398	ALU1_HUMAN (P39188) Alu subfamily J sequence contamination warning entry, partial (11%) [THC2693398]	7,734	6,185	1,55
THC2741996	ALU2_HUMAN (P39189) Alu subfamily SB sequence contamination warning entry, partial (6%) [THC2741996]	5,872	4,323	1,55
AIRE	Homo sapiens autoimmune regulator (autoimmune polyendocrinopathy candidiasis ectodermal dystrophy) (AIRE), transcript variant AIRE-2, mRNA [NM_000658]	5,72	4,172	1,55
CDSN	Homo sapiens corneodesmosin (CDSN), mRNA [NM_001264]	6,304	4,758	1,55
POLE	Homo sapiens DNA polymerase epsilon catalytic subunit isoform a (POLE1) mRNA, partial cds. [AF128541]	5,992	4,445	1,55
LOC340508	Homo sapiens hypothetical protein LOC340508 (LOC340508) on chromosome 9 [NR_002942]	7,839	6,292	1,55
RPL23AP13	Homo sapiens ribosomal protein L23a pseudogene 13 (RPL23AP13) on chromosome 2 [NR_002229]	9,518	7,973	1,55
EBI3	Homo sapiens Epstein-Barr virus induced gene 3 (EBI3), mRNA [NM_005755]	6,627	5,082	1,55
GTF2H4	Homo sapiens general transcription factor IIH, polypeptide 4, 52kDa (GTF2H4), mRNA [NM_001517]	8,58	7,037	1,54
ZBTB38	Homo sapiens cDNA FLJ36882 fis, clone BLADE2000340, weakly similar to ZINC FINGER PROTEIN 177. [AK094201]	11,12	9,579	1,54
COL23A1	Homo sapiens collagen, type XXIII, alpha 1 (COL23A1), mRNA [NM_173465]	6,419	4,878	1,54
EDG8	Homo sapiens endothelial differentiation, sphingolipid G-protein-coupled receptor, 8 (EDG8), mRNA [NM_030760]	6,415	4,874	1,54
LBX2	Homo sapiens ladybird homeobox homolog 2 (Drosophila) (LBX2), mRNA [NM_001009812]	5,661	4,121	1,54
LSS	Homo sapiens lanosterol synthase (2,3-oxidosqualene-lanosterol cyclase) (LSS), transcript variant 2, mRNA [NM_001001438]	8,244	6,705	1,54
CYP2B6	Homo sapiens cytochrome P450, family 2, subfamily B, polypeptide 6 (CYP2B6), mRNA [NM_000767]	5,747	4,209	1,54
NEUROD2	Homo sapiens neurogenic differentiation 2 (NEUROD2), mRNA [NM_006160]	5,64	4,104	1,54
TRIM47	Homo sapiens tripartite motif-containing 47 (TRIM47), mRNA [NM_033452]	10,414	8,88	1,54
CR619805	full-length cDNA clone CS0DI022YH23 of Placenta Cot 25-normalized of Homo sapiens (human). [CR619805]	6,857	5,323	1,53
CORO2B	Homo sapiens coronin, actin binding protein, 2B (CORO2B), mRNA [NM_006091]	8,319	6,786	1,53
PRMT2	Homo sapiens protein arginine methyltransferase 2 (PRMT2), transcript variant 1, mRNA [NM_206962]	10,935	9,403	1,53
LOC644974	Homo sapiens cDNA FLJ11976 fis, clone HEMBB1001253. [AK022038]	7,88	6,35	1,53
MYO1D	Homo sapiens myosin ID (MYO1D), mRNA [NM_015194]	9,276	7,745	1,53
-	chr8:000735716-000735657	7,904	6,378	1,53
SLC8A2	Homo sapiens solute carrier family 8 (sodium-calcium exchanger), member 2 (SLC8A2), mRNA [NM_015063]	6,45	4,925	1,53
M69012	Human guanine nucleotide-binding regulatory protein (G-y-2-alpha) mRNA, partial cds. [M69012]	7,964	6,438	1,53
THC2697162	chr3:115767656-115767597	9,37	7,845	1,53
-	chr6:137288357-137288298	6,081	4,557	1,53
AK123107	Homo sapiens cDNA FLJ41112 fis, clone BRACE1000239. [AK123107]	8,479	6,954	1,52
ADRA1D	Homo sapiens adrenergic, alpha-1D-, receptor (ADRA1D), mRNA [NM_000678]	7,313	5,79	1,52
AF161340	Homo sapiens HSPC077 mRNA, partial cds. [AF161340]	5,922	4,4	1,52
MGC5457	Homo sapiens hypothetical protein MGC5457, mRNA (cDNA clone MGC:5457 IMAGE:3450898), complete cds. [BC000988]	6,559	5,04	1,52
ACACB	Homo sapiens acetyl-Coenzyme A carboxylase beta (ACACB), mRNA [NM_001093]	5,579	4,062	1,52
PDIA3	Homo sapiens protein disulfide isomerase family A, member 3 (PDIA3), mRNA [NM_005313]	12,212	10,695	1,52
THC2614421	chr15:25460642-25460701	7,257	5,743	1,52
LOC401022	Homo sapiens hypothetical LOC401022, mRNA (cDNA clone IMAGE:4827714). [BC030713]	6,494	4,98	1,51

Supplemental Table 1
Morandi et al

ZFP90	Homo sapiens zinc finger protein 90 homolog (mouse) (ZFP90), mRNA [NM_133458]	7,903	6,389	1,51
-	chr11:001372532-001372473	6,988	5,475	1,51
AK226066	Homo sapiens mRNA for lysosomal-associated membrane protein 2 precursor variant, clone: fj10535. [AK226066]	10,079	8,567	1,51
SFTPA1	Homo sapiens surfactant, pulmonary-associated protein A1 (SFTPA1), mRNA [NM_005411]	6,026	4,515	1,51
AK056449	Homo sapiens cDNA FLJ31887 fis, clone NT2RP7003050. [AK056449]	6,221	4,713	1,51
DKFZp761B107	Homo sapiens hypothetical protein DKFZp761B107 (DKFZp761B107), mRNA [NM_173463]	6,865	5,357	1,51
ST3GAL4	Homo sapiens ST3 beta-galactoside alpha-2,3-sialyltransferase 4 (ST3GAL4), mRNA [NM_006278]	8,3	6,795	1,51
MFI2	Homo sapiens antigen p97 (melanoma associated) identified by monoclonal antibodies 133.2 and 96.5 (MFI2), transcript variant 1, mRNA [NM_005929]	5,767	4,265	1,50
ACADS	Homo sapiens acyl-Coenzyme A dehydrogenase, C-2 to C-3 short chain (ACADS), nuclear gene encoding mitochondrial protein, mRNA [NM_000017]	7,665	6,164	1,50
ABCA5	Homo sapiens ATP-binding cassette, sub-family A (ABC1), member 5 (ABCA5), transcript variant 1, mRNA [NM_018672]	10,154	8,657	1,50
LOC153346	Homo sapiens cDNA FLJ14284 fis, clone PLACE1005898. [AK024346]	6,348	4,851	1,50
HYAL1	Homo sapiens hyaluronoglucosaminidase 1 (HYAL1), transcript variant 1, mRNA [NM_007312]	6,467	4,97	1,50
SH3BGR	Homo sapiens SH3 domain binding glutamic acid-rich protein (SH3BGR), transcript variant 1, mRNA [NM_007341]	8,081	6,584	1,50
DFNB31	Homo sapiens deafness, autosomal recessive 31 (DFNB31), mRNA [NM_015404]	5,603	4,107	1,50
GANAB	Homo sapiens glucosidase, alpha; neutral AB (GANAB), transcript variant 3, mRNA [NM_198335]	12,781	11,284	1,50
MADCAM1	Homo sapiens mucosal vascular addressin cell adhesion molecule 1 (MADCAM1), transcript variant 1, mRNA [NM_130760]	6,454	4,959	1,50
OR52K2	Homo sapiens olfactory receptor, family 52, subfamily K, member 2 (OR52K2), mRNA [NM_001005172]	6,403	4,908	1,50
LTBP4	Homo sapiens latent transforming growth factor beta binding protein 4 (LTBP4), transcript variant 1, mRNA [NM_001042544]	7,345	5,852	1,49
PDAP1	Homo sapiens PDGFA associated protein 1 (PDAP1), mRNA [NM_014891]	10,711	9,219	1,49
SARDH	Homo sapiens sarcosine dehydrogenase (SARDH), mRNA [NM_007101]	7,309	5,817	1,49
TAOK3	Homo sapiens TAO kinase 3 (TAOK3), mRNA [NM_016281]	8,839	7,348	1,49
PBXIP1	Homo sapiens pre-B-cell leukemia transcription factor interacting protein 1 (PBXIP1), mRNA [NM_020524]	8,937	7,448	1,49
TEF	Homo sapiens thyrotrophic embryonic factor (TEF), mRNA [NM_003216]	8,715	7,226	1,49
GABBR1	Homo sapiens gamma-aminobutyric acid (GABA) B receptor, 1 (GABBR1), transcript variant 1, mRNA [NM_001470]	10,232	8,744	1,49
MST1R	Homo sapiens macrophage stimulating 1 receptor (c-met-related tyrosine kinase) (MST1R), mRNA [NM_002447]	6,763	5,275	1,49
KIAA0913	full-length cDNA clone CS0DI059YG13 of Placenta Cot 25-normalized of Homo sapiens (human). [CR610954]	6,879	5,394	1,49
HSD17B1	Homo sapiens hydroxysteroid (17-beta) dehydrogenase 1 (HSD17B1), mRNA [NM_000413]	7,244	5,759	1,49
BI828537	603078424F1 NIH_MGC_119 Homo sapiens cDNA clone IMAGE:5170397 5', mRNA sequence [BI828537]	5,544	4,06	1,48
BX095032	BX095032 Soares breast 2NbHBst Homo sapiens cDNA clone IMAGp998E17244, mRNA sequence [BX095032]	6,101	4,618	1,48
BC055421	Homo sapiens cDNA clone IMAGE:5269522. [BC055421]	5,808	4,325	1,48
BX362821	BX362821 BX362821 Homo sapiens HELA CELLS COT 25-NORMALIZED Homo sapiens cDNA clone CS0DK004YJ19 3-PRIME, mRNA sequence [BX362821]	6,379	4,898	1,48
THC2479944	AB042328 membrane-type 6 matrix metalloproteinase {Homo sapiens} (exp=-1; wgp=0; cg=0), partial (37%) [THC2479944]	5,68	4,2	1,48

Supplemental Table 1
Morandi et al

AK123431	Homo sapiens cDNA FLJ41437 fis, clone BRHIP2007928. [AK123431]	8,008	6,529	1,48
AF086468	Homo sapiens full length insert cDNA clone ZD86H05. [AF086468]	5,586	4,108	1,48
HAAO	Homo sapiens 3-hydroxyanthranilate 3,4-dioxygenase (HAAO), mRNA [NM_012205]	6,191	4,717	1,47
LOC653319	Homo sapiens cDNA FLJ34984 fis, clone OCBBF2001639. [AK092303]	6,331	4,857	1,47
THC2584954	BC008194 importin 13 {Homo sapiens} (exp=-1; wgp=0; cg=0), partial (7%) [THC2584954]	6,046	4,573	1,47
SLC4A11	Homo sapiens solute carrier family 4, sodium bicarbonate transporter-like, member 11 (SLC4A11), mRNA [NM_032034]	6,655	5,182	1,47
KCNIP2	Homo sapiens Kv channel interacting protein 2 (KCNIP2), transcript variant 8, mRNA [NM_173342]	6,837	5,365	1,47
DCST2	Homo sapiens DC-STAMP domain containing 2 (DCST2), mRNA [NM_144622]	6,057	4,586	1,47
HSD17B14	Homo sapiens hydroxysteroid (17-beta) dehydrogenase 14 (HSD17B14), mRNA [NM_016246]	6,875	5,405	1,47
PODXL	Homo sapiens podocalyxin-like (PODXL), transcript variant 1, mRNA [NM_001018111]	12,942	11,473	1,47
TNNT3	Homo sapiens troponin T type 3 (skeletal, fast) (TNNT3), transcript variant 1, mRNA [NM_001042780]	6,464	4,996	1,47
TRAF1	Homo sapiens TNF receptor-associated factor 1 (TRAF1), mRNA [NM_005658]	7,811	6,345	1,47
DMN	Homo sapiens desmuslin (DMN), transcript variant A, mRNA [NM_145728]	9,913	8,448	1,46
MAP9	Homo sapiens microtubule-associated protein 9 (MAP9), mRNA [NM_001039580]	8,49	7,029	1,46
CLDN7	Homo sapiens claudin 7 (CLDN7), mRNA [NM_001307]	5,715	4,255	1,46
LOC492311	Homo sapiens similar to bovine IgA regulatory protein (LOC492311), mRNA [NM_001007189]	9,189	7,731	1,46
RPL32P3	Homo sapiens cDNA FLJ39270 fis, clone OCBBF2010557. [AK096589]	8,471	7,016	1,46
FUS	Homo sapiens fusion (involved in t(12;16) in malignant liposarcoma) (FUS), transcript variant 1, mRNA [NM_004960]	12,713	11,257	1,46
-	chr12:011311938-011311997	7,403	5,948	1,46
CHRNB2	Homo sapiens cholinergic receptor, nicotinic, beta 2 (neuronal) (CHRNB2), mRNA [NM_000748]	6,884	5,43	1,45
TSPAN1	Homo sapiens tetraspanin 1 (TSPAN1), mRNA [NM_005727]	6,849	5,395	1,45
-	chr2:218560837-218560896	5,932	4,478	1,45
ZNF447	Homo sapiens zinc finger protein 447 (ZNF447), mRNA [NM_023926]	8,334	6,884	1,45
PPP1R3F	Homo sapiens protein phosphatase 1, regulatory (inhibitor) subunit 3F (PPP1R3F), mRNA [NM_033215]	8,027	6,578	1,45
KIAA0738	Homo sapiens KIAA0738 gene product (KIAA0738), mRNA [NM_014719]	10,428	8,98	1,45
THC2514053	FABPE_HUMAN (Q01469) Fatty acid-binding protein, epidermal (E-FABP) (Psoriasis-associated fatty acid-binding protein homolog) (PA-FABP), partial (19%) [THC2514053]	7,046	5,599	1,45
ETNK2	Homo sapiens ethanolamine kinase 2 (ETNK2), mRNA [NM_018208]	7,002	5,555	1,45
HCP5	Homo sapiens HLA complex P5 (HCP5), mRNA [NM_006674]	6,033	4,586	1,45
PEX5	Homo sapiens peroxisomal biogenesis factor 5 (PEX5), mRNA [NM_000319]	7,236	5,789	1,45
C6orf89	Homo sapiens chromosome 6 open reading frame 89 (C6orf89), mRNA [NM_152734]	9,383	7,937	1,45
TMPRSS6	Homo sapiens transmembrane protease, serine 6 (TMPRSS6), mRNA [NM_153609]	5,616	4,17	1,45
MEF2C	Homo sapiens MADS box transcription enhancer factor 2, polypeptide C (myocyte enhancer factor 2C) (MEF2C), mRNA [NM_002397]	9,253	7,809	1,44
TOLLIP	Homo sapiens toll interacting protein (TOLLIP), mRNA [NM_019009]	6,875	5,432	1,44
CELSR2	Homo sapiens cadherin, EGF LAG seven-pass G-type receptor 2 (flamingo homolog, Drosophila) (CELSR2), mRNA [NM_001408]	6,667	5,225	1,44

Supplemental Table 1
Morandi et al

ATRN	Homo sapiens attractin (ATRN), transcript variant 1, mRNA [NM_139321]	9,832	8,391	1,44
C10orf26	Homo sapiens chromosome 10 open reading frame 26 (C10orf26), mRNA [NM_017787]	6,341	4,901	1,44
PRPS1	Homo sapiens phosphoribosyl pyrophosphate synthetase 1 (PRPS1), mRNA [NM_002764]	9,551	8,113	1,44
CABP4	Homo sapiens calcium binding protein 4 (CABP4), mRNA [NM_145200]	6,268	4,832	1,44
LOC146346	Homo sapiens cDNA FLJ32797 fis, clone TESTI2002467. [AK057359]	10,776	9,34	1,44
ADAM15	Homo sapiens ADAM metallopeptidase domain 15 (metargidin) (ADAM15), transcript variant 1, mRNA [NM_207191]	8,315	6,882	1,43
SMARCD2	Homo sapiens SWI/SNF related, matrix associated, actin dependent regulator of chromatin, subfamily d, member 2 (SMARCD2), mRNA [NM_003077]	9,85	8,418	1,43
AKAP12	Homo sapiens A kinase (PRKA) anchor protein (gravin) 12 (AKAP12), transcript variant 2, mRNA [NM_144497]	9,253	7,822	1,43
AK057015	Homo sapiens cDNA FLJ32453 fis, clone SKMUS2001703. [AK057015]	9,01	7,578	1,43
C20orf194	Homo sapiens chromosome 20 open reading frame 194, mRNA (cDNA clone IMAGE:6189246), complete cds. [BC106086]	9,792	8,364	1,43
LGR6	Homo sapiens leucine-rich repeat-containing G protein-coupled receptor 6 (LGR6), transcript variant 1, mRNA [NM_001017403]	7,171	5,743	1,43
LOC441120	Homo sapiens similar to LOC285679 protein (LOC441120), mRNA [NM_001013718]	6,331	4,903	1,43
-	chr15_random:000081180-000081237	5,885	4,457	1,43
SYT15	Homo sapiens cDNA FLJ29001 fis, clone CBL08678. [AK131036]	6,639	5,213	1,43
CR591776	full-length cDNA clone CS0DI034YN06 of Placenta Cot 25-normalized of Homo sapiens (human). [CR591776]	7,656	6,23	1,43
-	chr12:047367883-047367824	7,454	6,029	1,43
PFKFB2	Homo sapiens 6-phosphofructo-2-kinase/fructose-2,6-biphosphatase 2 (PFKFB2), transcript variant 1, mRNA [NM_006212]	6,014	4,589	1,42
BX648950	Homo sapiens mRNA; cDNA DKFZp686E1648 (from clone DKFZp686E1648). [BX648950]	10,981	9,557	1,42
SLC23A2	Homo sapiens solute carrier family 23 (nucleobase transporters), member 2 (SLC23A2), transcript variant 2, mRNA [NM_203327]	9,483	8,061	1,42
ENST00000270201	Nucleolar preribosomal-associated protein 1 (Fragment). [Source:Uniprot/SWISSPROT;Acc:O60287] [ENST00000270201]	7,33	5,909	1,42
PNMA2	Homo sapiens paraneoplastic antigen MA2 (PNMA2), mRNA [NM_007257]	11,101	9,682	1,42
KRTAP10-10	Homo sapiens keratin associated protein 10-10 (KRTAP10-10), mRNA [NM_181688]	8,099	6,682	1,42
AQP11	Homo sapiens aquaporin 11 (AQP11), mRNA [NM_173039]	6,831	5,415	1,42
POLD2	Homo sapiens cDNA: FLJ21623 fis, clone COL07915. [AK025276]	6,796	5,38	1,42
SLC2A11	Homo sapiens solute carrier family 2 (facilitated glucose transporter), member 11 (SLC2A11), transcript variant 1, mRNA [NM_030807]	8,115	6,7	1,42
-	chr11:123414968-123414909	5,831	4,416	1,42
CHST10	Homo sapiens carbohydrate sulfotransferase 10 (CHST10), mRNA [NM_004854]	8,433	7,018	1,41
ASB4	Homo sapiens ankyrin repeat and SOCS box-containing 4 (ASB4), transcript variant 1, mRNA [NM_016116]	5,923	4,511	1,41
TNFAIP8L1	Homo sapiens tumor necrosis factor, alpha-induced protein 8-like 1 (TNFAIP8L1), mRNA [NM_152362]	6,63	5,218	1,41
CR608907	full-length cDNA clone CS0DM002YA18 of Fetal liver of Homo sapiens (human). [CR608907]	12,375	10,964	1,41
RXRB	Homo sapiens retinoid X receptor, beta (RXRB), mRNA [NM_021976]	9,941	8,53	1,41
SAP18	Homo sapiens Sin3A-associated protein, 18kDa (SAP18), mRNA [NM_005870]	9,487	8,077	1,41
KIAA1908	Homo sapiens mRNA for KIAA1908 protein, partial cds. [AB067495]	7,014	5,606	1,41

Supplemental Table 1
Morandi et al

-	chrX:049533337-049533396	9,191	7,783	1,41
BF246504	601854749F1 NIH_MGC_57 Homo sapiens cDNA clone IMAGE:4074517 5', mRNA sequence [BF246504]	7,502	6,096	1,41
ZNF500	Homo sapiens mRNA for KIAA0557 protein, partial cds. [AB011129]	6,673	5,266	1,41
NR4A1	Homo sapiens nuclear receptor subfamily 4, group A, member 1 (NR4A1), transcript variant 3, mRNA [NM_173158]	5,684	4,278	1,41
QTRT1	Homo sapiens queuine tRNA-ribosyltransferase 1 (tRNA-guanine transglycosylase) (QTRT1), mRNA [NM_031209]	9,412	8,005	1,41
ASMTL	Homo sapiens acetylserotonin O-methyltransferase-like (ASMTL), mRNA [NM_004192]	9,137	7,733	1,41
AK092888	Homo sapiens cDNA FLJ35569 fis, clone SPLEN2005783. [AK092888]	9,081	7,676	1,41
ENST00000358378	Mucin-5B precursor (Mucin-5 subtype B, tracheobronchial) (High molecular weight salivary mucin MG1) (Sublingual gland mucin). [Source:Uniprot/SWISSPROT;Acc:Q9HC84] [ENST00000358378]	5,699	4,297	1,40
ZDHHC1	Homo sapiens zinc finger, DHHC-type containing 1 (ZDHHC1), mRNA [NM_013304]	6,394	4,993	1,40
DEFB4	Homo sapiens defensin, beta 4 (DEFB4), mRNA [NM_004942]	5,877	4,477	1,40
THC2694693	chr20:1739840-1739781	5,594	4,194	1,40
SPG7	Homo sapiens spastic paraplegia 7, paraplegin (pure and complicated autosomal recessive) (SPG7), nuclear gene encoding mitochondrial protein, transcript variant 1, mRNA [NM_003119]	9,778	8,379	1,40
AK057830	Homo sapiens cDNA FLJ25101 fis, clone CBR01328. [AK057830]	6,923	5,526	1,40
RBM35B	Homo sapiens RNA binding motif protein 35B (RBM35B), mRNA [NM_024939]	6,493	5,096	1,40
GBA2	Homo sapiens glucosidase, beta (bile acid) 2 (GBA2), mRNA [NM_020944]	8,614	7,22	1,39
HNRPH1	Homo sapiens heterogeneous nuclear ribonucleoprotein H1 (H) (HNRPH1), mRNA [NM_005520]	10,826	9,432	1,39
NPTXR	Homo sapiens neuronal pentraxin receptor (NPTXR), mRNA [NM_014293]	7,112	5,718	1,39
LOC728007	MGC13005 protein. [Source:Uniprot/SPTREMBL;Acc:Q9BSF9] [ENST00000295199]	6,087	4,695	1,39
LOC728450	OTTHUMP00000030286 (Fragment). [Source:Uniprot/SPTREMBL;Acc:Q9NU29] [ENST00000358396]	6,311	4,919	1,39
KRT32	Homo sapiens keratin 32 (KRT32), mRNA [NM_002278]	6,954	5,563	1,39
DKFZP564J102	Homo sapiens DKFZP564J102 protein (DKFZP564J102), transcript variant 1, mRNA [NM_015398]	5,969	4,579	1,39
H1FNT	Homo sapiens H1 histone family, member N, testis-specific (H1FNT), mRNA [NM_181788]	6,406	5,02	1,39
AV647560	AV647560 GLC Homo sapiens cDNA clone GLCAZE12 3', mRNA sequence [AV647560]	6,744	5,36	1,38
ENST00000377515	Homo sapiens mRNA similar to hypothetical protein FLJ21463 (cDNA clone MGC:15793 IMAGE:3504595), complete cds. [BC008001]	6,489	5,105	1,38
POU5F1	Homo sapiens POU domain, class 5, transcription factor 1 (POU5F1), transcript variant 1, mRNA [NM_002701]	5,393	4,009	1,38
PITX3	Homo sapiens paired-like homeodomain transcription factor 3 (PITX3), mRNA [NM_005029]	6,039	4,657	1,38
-	chr6:128943363-128943302	7,085	5,704	1,38
FCHSD1	Homo sapiens FCH and double SH3 domains 1 (FCHSD1), mRNA [NM_033449]	6,148	4,768	1,38
HRH3	Homo sapiens histamine receptor H3 (HRH3), mRNA [NM_007232]	5,822	4,446	1,38
HISPPD2A	Homo sapiens histidine acid phosphatase domain containing 2A (HISPPD2A), transcript variant 4, mRNA [NM_001024463]	8,61	7,236	1,38
ZBED1	Homo sapiens zinc finger, BED-type containing 1 (ZBED1), mRNA [NM_004729]	9,96	8,585	1,38
CR745430	CR745430 Soares_testis_NHT Homo sapiens cDNA clone IMAGp971P1077 ; IMAGE:1839490 5', mRNA sequence [CR745430]	6,37	4,996	1,37

Supplemental Table 1
Morandi et al

VPS35	Homo sapiens vacuolar protein sorting 35 homolog (S. cerevisiae) (VPS35), mRNA [NM_018206]	10,959	9,585	1,37
-	chr4:075082788-075082729	6,006	4,632	1,37
HPN	Homo sapiens hepsin (transmembrane protease, serine 1) (HPN), transcript variant 1, mRNA [NM_182983]	5,572	4,199	1,37
MBNL2	Homo sapiens muscleblind-like 2 (Drosophila) (MBNL2), transcript variant 1, mRNA [NM_144778]	11,848	10,476	1,37
FADD	Homo sapiens Fas (TNFRSF6)-associated via death domain (FADD), mRNA [NM_003824]	8,156	6,785	1,37
KIAA0329	Homo sapiens KIAA0329 (KIAA0329), mRNA [NM_014844]	9,952	8,581	1,37
KIF3B	Homo sapiens kinesin family member 3B (KIF3B), mRNA [NM_004798]	8,783	7,412	1,37
OXTR	Homo sapiens oxytocin receptor (OXTR), mRNA [NM_000916]	6,142	4,772	1,37
PHC1	Homo sapiens polyhomeotic homolog 1 (Drosophila) (PHC1), mRNA [NM_004426]	8,325	6,955	1,37
COVA1	Homo sapiens cytosolic ovarian carcinoma antigen 1 (COVA1), transcript variant 2, mRNA [NM_182314]	5,923	4,554	1,37
JRK	Homo sapiens jerky homolog (mouse) (JRK), transcript variant 1, mRNA [NM_003724]	7,875	6,506	1,37
PNPLA7	Homo sapiens patatin-like phospholipase domain containing 7 (PNPLA7), mRNA [NM_152286]	8,019	6,65	1,37
RAB14	Homo sapiens RAB14, member RAS oncogene family (RAB14), mRNA [NM_016322]	11,224	9,857	1,37
HIF3A	Homo sapiens hypoxia inducible factor 3, alpha subunit (HIF3A), transcript variant 2, mRNA [NM_022462]	6,089	4,723	1,37
LMAN1L	Homo sapiens lectin, mannose-binding, 1 like (LMAN1L), mRNA [NM_021819]	6,405	5,04	1,37
-	chr7:006724586-006724534	5,772	4,409	1,36
CDIPT	Homo sapiens CDP-diacylglycerol--inositol 3-phosphatidyltransferase (phosphatidylinositol synthase) (CDIPT), mRNA [NM_006319]	10,657	9,295	1,36
NEDD4L	Homo sapiens neural precursor cell expressed, developmentally down-regulated 4-like (NEDD4L), mRNA [NM_015277]	12,26	10,898	1,36
GALNT11	Homo sapiens UDP-N-acetyl-alpha-D-galactosamine:polypeptide N-acetylgalactosaminyltransferase 11 (GalNAc-T11) (GALNT11), mRNA [NM_022087]	8,748	7,386	1,36
AGPAT6	Homo sapiens 1-acylglycerol-3-phosphate O-acyltransferase 6 (lysophosphatidic acid acyltransferase, zeta) (AGPAT6), mRNA [NM_178819]	10,848	9,487	1,36
GAL3ST4	Homo sapiens galactose-3-O-sulfotransferase 4 (GAL3ST4), mRNA [NM_024637]	5,892	4,533	1,36
-	chr19:053150680-053150739	6,038	4,679	1,36
MMP19	Homo sapiens matrix metallopeptidase 19 (MMP19), transcript variant 1, mRNA [NM_002429]	7,41	6,051	1,36
LOC148413	Homo sapiens cDNA FLJ35253 fis, clone PROST2003725. [AK092572]	9,48	8,124	1,36
C2orf27	Homo sapiens chromosome 2 open reading frame 27 (C2orf27), mRNA [NM_013310]	7,538	6,182	1,36
C10orf46	Homo sapiens chromosome 10 open reading frame 46 (C10orf46), mRNA [NM_153810]	9,74	8,385	1,36
NBPF1	Homo sapiens cDNA FLJ41266 fis, clone BRAMY2035869. [AK123260]	7,193	5,84	1,35
DKFZP564C196	Homo sapiens mRNA; cDNA DKFZp564C196 (from clone DKFZp564C196). [AL050020]	6,1	4,746	1,35
USP31	Homo sapiens mRNA for KIAA1203 protein, partial cds. [AB033029]	9,239	7,887	1,35
CPE	Homo sapiens carboxypeptidase E (CPE), mRNA [NM_001873]	13,6	12,254	1,35
ENST00000333529	Homo sapiens C21orf86 protein (C21orf86) mRNA, complete cds. [AF426264]	5,99	4,646	1,34
PTP4A3	Homo sapiens protein tyrosine phosphatase type IVA, member 3 (PTP4A3), transcript variant 1, mRNA [NM_032611]	8,169	6,825	1,34
PKNOX2	Homo sapiens PBX/knotted 1 homeobox 2 (PKNOX2), mRNA [NM_022062]	7,011	5,669	1,34
ESCO1	Homo sapiens establishment of cohesion 1 homolog 1 (S. cerevisiae) (ESCO1), mRNA [NM_052911]	7,002	5,661	1,34

Supplemental Table 1
Morandi et al

STK32C	Homo sapiens serine/threonine kinase 32C (STK32C), mRNA [NM_173575]	10,392	9,051	1,34
AKT3	RAC-gamma serine/threonine-protein kinase (EC 2.7.11.1) (RAC-PK-gamma) (Protein kinase Akt-3) (Protein kinase B, gamma) (PKB gamma) (STK-2). [Source:Uniprot/SWISSPROT;Acc:Q9Y243] [ENST00000366539]	10,657	9,316	1,34
MYOM1	Homo sapiens myomesin 1 (skelemin) 185kDa (MYOM1), mRNA [NM_003803]	8,295	6,955	1,34
CTDSPL	Homo sapiens CTD (carboxy-terminal domain, RNA polymerase II, polypeptide A) small phosphatase-like (CTDSPL), transcript variant 1, mRNA [NM_001008392]	8,712	7,375	1,34
SH3BGRL2	Homo sapiens SH3 domain binding glutamic acid-rich protein like 2 (SH3BGRL2), mRNA [NM_031469]	8,892	7,554	1,34
ZAN	Homo sapiens zonadhesin (ZAN), transcript variant 6, mRNA [NM_173059]	5,955	4,621	1,33
LOC147650	Homo sapiens clone DNA92254 ALLA2487 (UNQ2487) mRNA, complete cds. [AY358799]	6,485	5,153	1,33
KRTAP4-12	Homo sapiens keratin associated protein 4-12 (KRTAP4-12), mRNA [NM_031854]	5,207	3,875	1,33
NCOA7	Homo sapiens nuclear receptor coactivator 7 (NCOA7), mRNA [NM_181782]	9,624	8,292	1,33
WDR23	Homo sapiens WD repeat domain 23 (WDR23), transcript variant 1, mRNA [NM_025230]	10,309	8,977	1,33
-	chr1:013252840-013252779	6,032	4,7	1,33
GPR42	Homo sapiens G protein-coupled receptor 42 (GPR42), mRNA [NM_005305]	5,702	4,37	1,33
BC033829	Homo sapiens cDNA clone IMAGE:3856003, partial cds. [BC033829]	12,509	11,179	1,33
SLC6A18	Homo sapiens solute carrier family 6, member 18 (SLC6A18), mRNA [NM_182632]	5,958	4,628	1,33
-	chr2:001516473-001516532	5,91	4,58	1,33
FLT4	Homo sapiens fms-related tyrosine kinase 4 (FLT4), transcript variant 1, mRNA [NM_182925]	6,565	5,238	1,33
-	chr11:095664657-095664598	6,59	5,261	1,33
HAGHL	Homo sapiens hydroxyacylglutathione hydrolase-like (HAGHL), transcript variant 2, mRNA [NM_032304]	8,817	7,49	1,33
RP11-34P13.4	Homo sapiens hypothetical protein (F379) mRNA, complete cds. [AF346307]	5,897	4,57	1,33
THC2706493	GLUQ_CHRVO (Q7NSJ1) Glutamyl-Q tRNA(Asp) synthetase (Glu-Q-RSs) , partial (5%) [THC2706493]	5,78	4,454	1,33
GPD1	Homo sapiens glycerol-3-phosphate dehydrogenase 1 (soluble) (GPD1), mRNA [NM_005276]	5,427	4,104	1,32
MAPK3	Homo sapiens mitogen-activated protein kinase 3 (MAPK3), transcript variant 1, mRNA [NM_002746]	8,856	7,533	1,32
THC2738359	chr16:030152700-030152641	5,644	4,321	1,32
PLCH2	Homo sapiens phospholipase C, eta 2 (PLCH2), mRNA [NM_014638]	6,989	5,667	1,32
PTGDR	Homo sapiens prostaglandin D2 receptor (DP) (PTGDR), mRNA [NM_000953]	5,684	4,363	1,32
INPP5E	Homo sapiens inositol polyphosphate-5-phosphatase, 72 kDa (INPP5E), mRNA [NM_019892]	6,123	4,805	1,32
SCARF1	Homo sapiens scavenger receptor class F, member 1 (SCARF1), transcript variant 4, mRNA [NM_145351]	7,437	6,118	1,32
BC010426	Homo sapiens cDNA clone MGC:15478 IMAGE:2967661, complete cds. [BC010426]	5,548	4,23	1,32
GPR144	Probable G-protein coupled receptor 144 (G-protein coupled receptor PGR24). [Source:Uniprot/SWISSPROT;Acc:Q7Z7M1] [ENST00000334810]	5,797	4,48	1,32
BF718543	BF718543 EST316 microdissected normal human epidermis Homo sapiens cDNA clone S90328.NIH-329-R 3', mRNA sequence [BF718543]	6,193	4,877	1,32
KIAA0082	Homo sapiens KIAA0082 (KIAA0082), mRNA [NM_015050]	10,591	9,275	1,32
LOC401010	Homo sapiens hypothetical LOC401010 (LOC401010) on chromosome 2 [NR_002826]	6,153	4,838	1,32

Supplemental Table 1
Morandi et al

AK095904	Homo sapiens cDNA FLJ38585 fis, clone HCHON2009191. [AK095904]	6,878	5,566	1,31
LPHN3	Homo sapiens latrophilin 3 (LPHN3), mRNA [NM_015236]	10,971	9,658	1,31
NRIP2	Homo sapiens nuclear receptor interacting protein 2 (NRIP2), mRNA [NM_031474]	6,076	4,767	1,31
FRMD1	Homo sapiens FERM domain containing 1 (FRMD1), mRNA [NM_024919]	5,483	4,173	1,31
OXCT2	Homo sapiens mRNA for FLJ00030 protein, partial cds. [AK024440]	8,03	6,723	1,31
TEX2	Homo sapiens testis expressed sequence 2 (TEX2), mRNA [NM_018469]	11,627	10,32	1,31
ING5	Homo sapiens inhibitor of growth family, member 5 (ING5), mRNA [NM_032329]	6,402	5,097	1,31
RER1	Homo sapiens RER1 retention in endoplasmic reticulum 1 homolog (S. cerevisiae) (RER1), mRNA [NM_007033]	7,505	6,2	1,31
FBXO34	Homo sapiens CGI-301 protein mRNA, complete cds. [AF531436]	10,977	9,673	1,30
C13orf16	Homo sapiens chromosome 13 open reading frame 16 (C13orf16), mRNA [NM_152324]	5,238	3,934	1,30
CNDP2	Homo sapiens CNDP dipeptidase 2 (metallopeptidase M20 family) (CNDP2), mRNA [NM_018235]	10,394	9,093	1,30
DUB3	Homo sapiens deubiquitinating enzyme 3 (DUB3), mRNA [NM_201402]	6,608	5,307	1,30
PKN3	Homo sapiens protein kinase N3 (PKN3), mRNA [NM_013355]	6,786	5,486	1,30
ADCY5	Homo sapiens adenylate cyclase 5 (ADCY5), mRNA [NM_183357]	7,16	5,861	1,30
H6PD	Homo sapiens hexose-6-phosphate dehydrogenase (glucose 1-dehydrogenase) (H6PD), mRNA [NM_004285]	6,704	5,407	1,30
MAGEE1	Homo sapiens melanoma antigen family E, 1 (MAGEE1), mRNA [NM_020932]	8,539	7,242	1,30
NUMA1	Homo sapiens nuclear mitotic apparatus protein 1 (NUMA1), mRNA [NM_006185]	10,234	8,938	1,30
CILP2	Homo sapiens cartilage intermediate layer protein 2 (CILP2), mRNA [NM_153221]	6,236	4,941	1,30
FLJ32679	Homo sapiens golgin-like hypothetical protein LOC440321 (FLJ32679), mRNA [NM_001012452]	7,821	6,527	1,29
-	chr8:142398210-142398151	6,832	5,539	1,29
TNNI1	Homo sapiens troponin I type 1 (skeletal, slow) (TNNI1), mRNA [NM_003281]	5,68	4,388	1,29
C1QTNF6	Homo sapiens C1q and tumor necrosis factor related protein 6 (C1QTNF6), transcript variant 1, mRNA [NM_031910]	9,125	7,835	1,29
RAB35	Homo sapiens RAB35, member RAS oncogene family (RAB35), mRNA [NM_006861]	13,187	11,896	1,29
ZNF808	Homo sapiens zinc finger protein 808 (ZNF808), mRNA [NM_001039886]	8,362	7,072	1,29
ZCCHC12	Homo sapiens zinc finger, CCHC domain containing 12 (ZCCHC12), mRNA [NM_173798]	9,773	8,483	1,29
UPK2	Homo sapiens uroplakin 2 (UPK2), mRNA [NM_006760]	6,046	4,758	1,29
CISH	Homo sapiens cytokine inducible SH2-containing protein (CISH), mRNA [NM_145071]	5,532	4,245	1,29
EDG5	Homo sapiens endothelial differentiation, sphingolipid G-protein-coupled receptor, 5 (EDG5), mRNA [NM_004230]	5,914	4,626	1,29
TBC1D2B	Homo sapiens mRNA; cDNA DKFZp434O086 (from clone DKFZp434O086). [AL137303]	5,455	4,17	1,29
IRF2BP2	Homo sapiens interferon regulatory factor 2 binding protein 2 (IRF2BP2), transcript variant 1, mRNA [NM_182972]	11,297	10,013	1,28
THC2713663	chr4:80448740-80448799	6,355	5,07	1,28
NDRG3	Homo sapiens NDRG family member 3 (NDRG3), transcript variant 1, mRNA [NM_032013]	9,839	8,556	1,28
RAD50	Homo sapiens RAD50 homolog (S. cerevisiae) (RAD50), transcript variant 1, mRNA [NM_005732]	6,765	5,483	1,28
LOC151534	Homo sapiens cDNA clone IMAGE:3940360. [BC009264]	7,672	6,39	1,28
-	chr8:140837676-140837735	5,862	4,58	1,28
SLC17A7	Homo sapiens solute carrier family 17 (sodium-dependent inorganic phosphate cotransporter), member 7 (SLC17A7), mRNA [NM_020309]	7,127	5,848	1,28

UMOD	Homo sapiens uromodulin (uromucoid, Tamm-Horsfall glycoprotein) (UMOD), transcript variant 1, mRNA [NM_003361]	6,108	4,828	1,28
BC035518	Homo sapiens, clone IMAGE:4214654, mRNA. [BC035518]	10,427	9,147	1,28
KCTD2	Homo sapiens potassium channel tetramerisation domain containing 2 (KCTD2), mRNA [NM_015353]	11,296	10,017	1,28
SLC6A13	Homo sapiens solute carrier family 6 (neurotransmitter transporter, GABA), member 13 (SLC6A13), mRNA [NM_016615]	5,499	4,22	1,28
LOC283143	Homo sapiens cDNA FLJ33283 fis, clone ASTRO2009177. [AK090602]	8,208	6,929	1,28
THC2612620	chr22:36695140-36695199	8,851	7,572	1,28
FBXO15	Homo sapiens F-box protein 15 (FBXO15), mRNA [NM_152676]	8,083	6,808	1,28
INHBC	Homo sapiens inhibin, beta C (INHBC), mRNA [NM_005538]	6,541	5,268	1,27
AW138098	UI-H-BI1-abw-g-06-0-UI.s1 NCI_CGAP_Sub3 Homo sapiens cDNA clone IMAGE:2713522 3', mRNA sequence [AW138098]	5,404	4,13	1,27
-	chr17:077775381-077775440	5,516	4,242	1,27
PLCXD2	Homo sapiens phosphatidylinositol-specific phospholipase C, X domain containing 2 (PLCXD2), mRNA [NM_153268]	6,22	4,948	1,27
AI906074	AI906074 RC-BT105-270399-130 BT105 Homo sapiens cDNA, mRNA sequence [AI906074]	7,167	5,896	1,27
C3orf40	Homo sapiens chromosome 3 open reading frame 40 (C3orf40), mRNA [NM_144635]	9,635	8,364	1,27
AK090481	Homo sapiens mRNA for FLJ00403 protein. [AK090481]	6,251	4,981	1,27
PANK4	Homo sapiens pantothenate kinase 4 (PANK4), mRNA [NM_018216]	8,864	7,594	1,27
PRPF40B	Homo sapiens PRP40 pre-mRNA processing factor 40 homolog B (S. cerevisiae) (PRPF40B), transcript variant 1, mRNA [NM_001031698]	10,781	9,512	1,27
LOC390610	PREDICTED: Homo sapiens similar to Keratin, type II cytoskeletal 8 (Cytokeratin-8) (CK-8) (Keratin-8) (K8) (LOC390610), mRNA [XR_017341]	6,455	5,187	1,27
SAMD14	Homo sapiens sterile alpha motif domain containing 14 (SAMD14), mRNA [NM_174920]	8,974	7,707	1,27
VPS13D	Homo sapiens vacuolar protein sorting 13 homolog D (S. cerevisiae) (VPS13D), transcript variant 1, mRNA [NM_015378]	6,443	5,178	1,26
WNT11	Homo sapiens wingless-type MMTV integration site family, member 11 (WNT11), mRNA [NM_004626]	5,952	4,688	1,26
MECP2	Homo sapiens methyl CpG binding protein 2 (Rett syndrome) (MECP2), mRNA [NM_004992]	9,408	8,145	1,26
C15orf17	Homo sapiens mRNA for FLJ00005 protein, partial cds. [AK000005]	9,545	8,281	1,26
KCNQ3	Homo sapiens potassium voltage-gated channel, KQT-like subfamily, member 3 (KCNQ3), mRNA [NM_004519]	6,207	4,944	1,26
-	chr9:005408444-005408385	6,377	5,114	1,26
ORMDL3	Homo sapiens ORM1-like 3 (S. cerevisiae) (ORMDL3), mRNA [NM_139280]	6,977	5,717	1,26
PROP1	Homo sapiens prophet of Pit1, paired-like homeodomain transcription factor (PROP1), mRNA [NM_006261]	7,552	6,292	1,26
FLJ00049	Homo sapiens mRNA for FLJ00049 protein, partial cds. [AK024457]	6,531	5,272	1,26
LOC256273	Homo sapiens cDNA FLJ10046 fis, clone HEMBA1001133. [AK000908]	8	6,742	1,26
CLDN9	Homo sapiens claudin 9 (CLDN9), mRNA [NM_020982]	6,451	5,194	1,26
FLOT1	Homo sapiens flotillin 1 (FLOT1), mRNA [NM_005803]	9,206	7,949	1,26
TMEM115	Homo sapiens transmembrane protein 115 (TMEM115), mRNA [NM_007024]	7,964	6,707	1,26
ASB13	Homo sapiens ankyrin repeat and SOCS box-containing 13 (ASB13), mRNA [NM_024701]	7,647	6,392	1,26
SIRT3	Homo sapiens sirtuin (silent mating type information regulation 2 homolog) 3 (S. cerevisiae) (SIRT3), transcript variant 1, mRNA [NM_012239]	9,283	8,029	1,26
DLST	Homo sapiens dihydrolipoamide S-succinyltransferase (E2 component of 2-oxo-glutarate complex) (DLST), mRNA [NM_001933]	6,644	5,391	1,25

Supplemental Table 1
Morandi et al

UPF3A	Homo sapiens UPF3 regulator of nonsense transcripts homolog A (yeast) (UPF3A), transcript variant 1, mRNA [NM_023011]	8,464	7,211	1,25
ZFP95	Homo sapiens zinc finger protein 95 homolog (mouse) (ZFP95), transcript variant 1, mRNA [NM_014569]	8,086	6,835	1,25
HS1BP3	Homo sapiens HCLS1 binding protein 3 (HS1BP3), mRNA [NM_022460]	8,721	7,472	1,25
SUFU	Homo sapiens suppressor of fused homolog (Drosophila) (SUFU), mRNA [NM_016169]	7,784	6,535	1,25
ZNF346	Homo sapiens zinc finger protein 346 (ZNF346), mRNA [NM_012279]	5,818	4,568	1,25
KIAA1414	Homo sapiens KIAA1414 protein (KIAA1414), mRNA [NM_019024]	8,792	7,545	1,25
DKFZp434F142	Homo sapiens mRNA; cDNA DKFZp434F142 (from clone DKFZp434F142). [AL136837]	5,622	4,375	1,25
THC2643320	Q306F7_HUMAN (Q306F7) Down syndrome encephalopathy related protein 1, partial (11%) [THC2643320]	9,004	7,757	1,25
LOC162427	Homo sapiens hypothetical protein LOC162427 (LOC162427), mRNA [NM_178126]	10,486	9,24	1,25
LZTS2	Homo sapiens leucine zipper, putative tumor suppressor 2 (LZTS2), mRNA [NM_032429]	11,24	9,995	1,25
UPK3B	Homo sapiens uroplakin 3B (UPK3B), transcript variant 1, mRNA [NM_030570]	6,314	5,071	1,24
KLHL22	Homo sapiens kelch-like 22 (Drosophila) (KLHL22), mRNA [NM_032775]	9,026	7,783	1,24
-	chr17:039662079-039661719	6,919	5,677	1,24
TFF1	Homo sapiens trefoil factor 1 (breast cancer, estrogen-inducible sequence expressed in) (TFF1), mRNA [NM_003225]	6,455	5,215	1,24
FHOD3	Homo sapiens formin homology 2 domain containing 3 (FHOD3), mRNA [NM_025135]	11,559	10,32	1,24
AK090442	Homo sapiens mRNA for FLJ00363 protein. [AK090442]	5,765	4,526	1,24
KRT31	Homo sapiens keratin 31 (KRT31), mRNA [NM_002277]	6,589	5,351	1,24
HRH2	Homo sapiens histamine receptor H2 (HRH2), mRNA [NM_022304]	5,841	4,604	1,24
ABHD6	Homo sapiens abhydrolase domain containing 6 (ABHD6), mRNA [NM_020676]	9,759	8,525	1,23
SF3B3	Homo sapiens splicing factor 3b, subunit 3, 130kDa (SF3B3), mRNA [NM_012426]	10,653	9,419	1,23
-	chr4:119334082-119334023	7,466	6,232	1,23
SNX24	Homo sapiens sorting nexing 24 (SNX24), mRNA [NM_014035]	8,672	7,439	1,23
THC2683530	chr1:224613398-224613339	6,827	5,595	1,23
AA282192	AA282192 zs89b10.r1 NCI_CGAP_GCB1 Homo sapiens cDNA clone IMAGE:704635 5', mRNA sequence [AA282192]	6,948	5,715	1,23
LGALS3BP	Homo sapiens lectin, galactoside-binding, soluble, 3 binding protein (LGALS3BP), mRNA [NM_005567]	9,793	8,562	1,23
KIAA0152	Homo sapiens KIAA0152 (KIAA0152), mRNA [NM_014730]	8,22	6,991	1,23
PRSS8	Homo sapiens protease, serine, 8 (prostasin) (PRSS8), mRNA [NM_002773]	6,499	5,27	1,23
-	chr16:054937059-054937000	6,236	5,007	1,23
KRTAP19-1	Homo sapiens partial mRNA for high tyrosine glycine keratin associated protein 19.1 (KRTAP19.1 gene). [AJ457067]	6,16	4,934	1,23
THC2654381	chr8:006971328-006971269	7,009	5,784	1,23
BC021053	Homo sapiens cDNA clone IMAGE:2960979, **** WARNING: chimeric clone ****. [BC021053]	8,459	7,234	1,23
AP3S2	Homo sapiens adaptor-related protein complex 3, sigma 2 subunit (AP3S2), mRNA [NM_005829]	6,739	5,515	1,22
C19orf19	Homo sapiens chromosome 19 open reading frame 19 (C19orf19), mRNA [NM_182577]	7,422	6,198	1,22
TUT1	Homo sapiens terminal uridylyl transferase 1, U6 snRNA-specific (TUT1), mRNA [NM_022830]	8,402	7,178	1,22
CHD8	Homo sapiens chromodomain helicase DNA binding protein 8 (CHD8), mRNA [NM_020920]	8,759	7,537	1,22
EPN3	Homo sapiens epsin 3 (EPN3), mRNA [NM_017957]	5,459	4,239	1,22
FLJ23569	Homo sapiens cDNA: FLJ23569 fis, clone LNG11861. [AK027222]	8,584	7,366	1,22

Supplemental Table 1
Morandi et al

AP2B1	Homo sapiens adaptor-related protein complex 2, beta 1 subunit (AP2B1), transcript variant 1, mRNA [NM_001030006]	11,74	10,522	1,22
MGC16824	Homo sapiens esophageal cancer associated protein (MGC16824), mRNA [NM_020314]	9,14	7,921	1,22
WDR7	Homo sapiens WD repeat domain 7 (WDR7), transcript variant 1, mRNA [NM_015285]	10,458	9,241	1,22
AF086139	Homo sapiens full length insert cDNA clone ZA91F08. [AF086139]	10,184	8,969	1,22
BC014395	Homo sapiens, clone IMAGE:3029191, mRNA. [BC014395]	10,83	9,614	1,22
FLJ38379	Homo sapiens cDNA FLJ38379 fis, clone FEBRA2002986. [AK095698]	7,15	5,936	1,22
EGLN2	Homo sapiens egl nine homolog 2 (C. elegans) (EGLN2), transcript variant 3, mRNA [NM_080732]	11,048	9,833	1,22
KLHDC5	Homo sapiens kelch domain containing 5 (KLHDC5), mRNA [NM_020782]	10,842	9,628	1,22
ALMS1	Homo sapiens Alstrom syndrome 1 (ALMS1), mRNA [NM_015120]	8,701	7,487	1,21
AK057719	Homo sapiens cDNA FLJ33157 fis, clone UTERU2000393. [AK057719]	6,373	5,161	1,21
C14orf152	Homo sapiens chromosome 14 open reading frame 152 (C14orf152), mRNA [NM_138344]	6,358	5,147	1,21
THC2681718	chr14:65944004-65943945	6,345	5,133	1,21
AK124344	Homo sapiens cDNA FLJ42353 fis, clone UTERU2007520. [AK124344]	7,355	6,144	1,21
RSHL1	Homo sapiens radial spokehead-like 1 (RSHL1), mRNA [NM_030785]	6,115	4,903	1,21
ANKRD33	Homo sapiens ankyrin repeat domain 33 (ANKRD33), mRNA [NM_182608]	6,976	5,766	1,21
CC2D1B	Homo sapiens coiled-coil and C2 domain containing 1B (CC2D1B), mRNA [NM_032449]	9,54	8,331	1,21
BG547692	602575538F1 NIH_MGC_77 Homo sapiens cDNA clone IMAGE:4703745 5', mRNA sequence [BG547692]	6,048	4,84	1,21
ACR	Homo sapiens acrosin (ACR), mRNA [NM_001097]	6,606	5,398	1,21
ADARB1	Homo sapiens adenosine deaminase, RNA-specific, B1 (RED1 homolog rat) (ADARB1), transcript variant 1, mRNA [NM_001112]	10,611	9,406	1,21
AK096196	Homo sapiens cDNA FLJ38877 fis, clone MESAN2015277. [AK096196]	6,182	4,977	1,21
BC015370	Homo sapiens cDNA clone IMAGE:3916023, partial cds. [BC015370]	8,612	7,408	1,20
GPR12	Homo sapiens G protein-coupled receptor 12 (GPR12), mRNA [NM_005288]	6,873	5,67	1,20
ALAD	Homo sapiens aminolevulinate, delta-, dehydratase (ALAD), transcript variant 1, mRNA [NM_001003945]	9,547	8,344	1,20
ENST0000037367(	Membrane-associated nucleic acid-binding protein (RING finger protein 164). [Source:Uniprot/SWISSPROT;Acc:Q9HBD1] [ENST00000373670]	10,202	9	1,20
MYADML	Homo sapiens myeloid-associated differentiation marker-like (MYADML) on chromosome 2 [NR_003143]	5,827	4,626	1,20
LRRC27	Homo sapiens leucine rich repeat containing 27 (LRRC27), mRNA [NM_030626]	8,257	7,057	1,20
TAOK2	Homo sapiens TAO kinase 2 (TAOK2), transcript variant 1, mRNA [NM_004783]	9,503	8,303	1,20
AK124263	Homo sapiens cDNA FLJ42269 fis, clone TKIDN2015285. [AK124263]	9,381	8,182	1,20
ZNF572	Homo sapiens zinc finger protein 572 (ZNF572), mRNA [NM_152412]	8,809	7,611	1,20
THC2675117	D90774 RecT protein (P33). {Escherichia coli} (exp=-1; wgp=0; cg=0), partial (5%) [THC2675117]	6,963	5,769	1,19
FIBCD1	Homo sapiens fibrinogen C domain containing 1 (FIBCD1), mRNA [NM_032843]	5,673	4,479	1,19
FLJ12331	Homo sapiens hypothetical protein FLJ12331 (FLJ12331), mRNA [NM_024986]	5,739	4,545	1,19
FBXW4	Homo sapiens F-box and WD-40 domain protein 4 (FBXW4), mRNA [NM_022039]	8,597	7,404	1,19

UBE2Z	Homo sapiens ubiquitin-conjugating enzyme E2Z (putative) (UBE2Z), mRNA [NM_023079]	8,721	7,53	1,19
LOC388323	Homo sapiens hypothetical LOC388323 (LOC388323), mRNA [NM_001014985]	6,141	4,95	1,19
CTGLF4	Homo sapiens centaurin, gamma-like family, member 4 (CTGLF4), mRNA [NM_001077685]	8,68	7,491	1,19
ENST00000339968	Amyotrophic lateral sclerosis 2 chromosomal region candidate gene 16 protein. [Source:Uniprot/SWISSPROT;Acc:Q6ZP36] [ENST00000339968]	8,852	7,664	1,19
SCNN1B	Homo sapiens sodium channel, nonvoltage-gated 1, beta (Liddle syndrome) (SCNN1B), mRNA [NM_000336]	6,01	4,822	1,19
THC2611204	chr11:122339225-122339284	7,604	6,415	1,19
CD40	Homo sapiens CD40 molecule, TNF receptor superfamily member 5 (CD40), transcript variant 1, mRNA [NM_001250]	7,573	6,386	1,19
HNRPUL2	Homo sapiens heterogeneous nuclear ribonucleoprotein U-like 2 (HNRPUL2), mRNA [NM_001079559]	7,798	6,611	1,19
THC2681047	chr15:98221141-98221200	5,341	4,157	1,19
AQP5	Homo sapiens aquaporin 5 (AQP5), mRNA [NM_001651]	7,307	6,125	1,18
BC013679	Homo sapiens cDNA clone IMAGE:3857956, partial cds. [BC013679]	6,235	5,053	1,18
SPATA20	Homo sapiens spermatogenesis associated 20 (SPATA20), mRNA [NM_022827]	10,535	9,353	1,18
ADAMTS13	Homo sapiens ADAM metallopeptidase with thrombospondin type 1 motif, 13 (ADAMTS13), transcript variant 2, mRNA [NM_139027]	6,817	5,636	1,18
TCEAL6	Homo sapiens transcription elongation factor A (SII)-like 6 (TCEAL6), mRNA [NM_001006938]	13,408	12,227	1,18
CCDC113	Homo sapiens coiled-coil domain containing 113 (CCDC113), mRNA [NM_014157]	6,189	5,008	1,18
CHMP4B	Homo sapiens chromatin modifying protein 4B (CHMP4B), mRNA [NM_176812]	11,157	9,979	1,18
CKAP5	Homo sapiens cytoskeleton associated protein 5 (CKAP5), transcript variant 1, mRNA [NM_001008938]	9,054	7,875	1,18
AK095300	Homo sapiens cDNA FLJ37981 fis, clone CTONG2010566. [AK095300]	6,548	5,371	1,18
IL18BP	Homo sapiens interleukin 18 binding protein (IL18BP), transcript variant A, mRNA [NM_173042]	5,963	4,787	1,18
CASKIN2	Homo sapiens CASK interacting protein 2 (CASKIN2), mRNA [NM_020753]	8,825	7,65	1,18
PRMT7	Homo sapiens protein arginine methyltransferase 7 (PRMT7), mRNA [NM_019023]	7,159	5,984	1,18
SLC36A3	Homo sapiens solute carrier family 36 (proton/amino acid symporter), member 3 (SLC36A3), mRNA [NM_181774]	5,556	4,381	1,18
DBT	Homo sapiens dihydrolipoamide branched chain transacylase E2 (DBT), nuclear gene encoding mitochondrial protein, mRNA [NM_001918]	9,241	8,067	1,17
TRGV5	Homo sapiens T cell receptor gamma variable 5, mRNA (cDNA clone MGC:45453 IMAGE:5575279), complete cds. [BC035592]	6,321	5,147	1,17
LOC644079	PREDICTED: Homo sapiens similar to Breakpoint cluster region protein (NY-REN-26 antigen) (LOC644079), mRNA [XR_017173]	8,181	7,009	1,17
ENST00000252134	Uncharacterized protein KIAA0819. [Source:Uniprot/SWISSPROT;Acc:O94909] [ENST00000252134]	10,654	9,481	1,17
BBC3	Homo sapiens BCL2 binding component 3 (BBC3), mRNA [NM_014417]	8,609	7,437	1,17
THC2709441	Q4VIX2_DROBU (Q4VIX2) Dbuz\abd-A-PB, partial (4%) [THC2709441]	5,851	4,679	1,17
FAM63A	family with sequence similarity 63, member A (FAM63A), transcript variant 1, mRNA [Source:RefSeq_dna;Acc:NM_018379] [ENST00000368945]	8,231	7,06	1,17
PLCB3	Homo sapiens phospholipase C, beta 3 (phosphatidylinositol-specific) (PLCB3), mRNA [NM_000932]	8,441	7,273	1,17
LOC349196	Homo sapiens mRNA for FLJ00326 protein. [AK090418]	8,665	7,499	1,17
SPTLC2	Homo sapiens serine palmitoyltransferase, long chain base subunit 2 (SPTLC2), mRNA [NM_004863]	7,335	6,169	1,17
IRGC	Homo sapiens immunity-related GTPase family, cinema (IRGC), mRNA [NM_019612]	5,644	4,479	1,17

Supplemental Table 1
Morandi et al

FKHL18	Homo sapiens forkhead-like 18 (Drosophila) (FKHL18), mRNA [NM_004118]	7,378	6,215	1,16
PLCG1	Homo sapiens phospholipase C, gamma 1 (PLCG1), transcript variant 1, mRNA [NM_002660]	9,334	8,17	1,16
MPP5	Homo sapiens membrane protein, palmitoylated 5 (MAGUK p55 subfamily member 5) (MPP5), mRNA [NM_022474]	6,585	5,422	1,16
PRIMA1	Homo sapiens proline rich membrane anchor 1 (PRIMA1), mRNA [NM_178013]	7,177	6,015	1,16
KIAA0515	Homo sapiens cDNA: FLJ22509 fis, clone HRC11803. [AK026162]	8,644	7,484	1,16
INTS5	Homo sapiens integrator complex subunit 5 (INTS5), mRNA [NM_030628]	7,604	6,444	1,16
FAM119B	Homo sapiens family with sequence similarity 119, member B (FAM119B), transcript variant 2, mRNA [NM_206914]	6,38	5,22	1,16
GJB3	Homo sapiens connexin 31 (GJB3) mRNA, complete cds. [AF052692]	6,555	5,397	1,16
COL4A3BP	Homo sapiens collagen, type IV, alpha 3 (Goodpasture antigen) binding protein (COL4A3BP), transcript variant 1, mRNA [NM_005713]	11,056	9,899	1,16
SEC63	Translocation protein SEC63 homolog. [Source:Uniprot/SWISSPROT;Acc:Q9UGP8] [ENST00000369002]	8,744	7,586	1,16
CTGLF5	Homo sapiens centaurin, gamma-like family, member 5 (CTGLF5), mRNA [NM_001077686]	8,583	7,428	1,16
SLC16A11	Homo sapiens solute carrier family 16, member 11 (monocarboxylic acid transporter 11) (SLC16A11), mRNA [NM_153357]	6,522	5,366	1,16
ZNF251	Zinc finger protein 251 (Fragment). [Source:Uniprot/SWISSPROT;Acc:Q9BRH9] [ENST00000292562]	8,041	6,886	1,16
ENST00000328411	Homo sapiens cDNA clone IMAGE:40031134. [BC104192]	5,463	4,309	1,15
GOLGA3	Homo sapiens golgi autoantigen, golgin subfamily a, 3 (GOLGA3), mRNA [NM_005895]	7,218	6,066	1,15
SNAP29	Homo sapiens synaptosomal-associated protein, 29kDa (SNAP29), mRNA [NM_004782]	6,856	5,703	1,15
PEAR1	Novel protein similar to mouse Jedi soluble isoform 736 protein. [Source:Uniprot/SPTREMBL;Acc:Q5VY43] [ENST00000292357]	6,806	5,652	1,15
FKBP4	Homo sapiens FK506 binding protein 4, 59kDa (FKBP4), mRNA [NM_002014]	7,921	6,769	1,15
ZNF638	Homo sapiens zinc finger protein 638 (ZNF638), transcript variant 1, mRNA [NM_014497]	9,784	8,632	1,15
NEK8	Homo sapiens NIMA (never in mitosis gene a)- related kinase 8 (NEK8), mRNA [NM_178170]	6,664	5,514	1,15
-	chr2:242356178-242356233	9,084	7,934	1,15
DAB1	Homo sapiens disabled homolog 1 (Drosophila) (DAB1), mRNA [NM_021080]	6,059	4,91	1,15
KIDINS220	Homo sapiens kinase D-interacting substance of 220 kDa (KIDINS220), mRNA [NM_020738]	12,084	10,936	1,15
TCEAL3	Homo sapiens transcription elongation factor A (SII)-like 3 (TCEAL3), transcript variant 1, mRNA [NM_001006933]	13,707	12,559	1,15
AK057720	Homo sapiens cDNA FLJ33158 fis, clone UTERU2000418. [AK057720]	7,705	6,559	1,15
-	chr15_random:000033401-000033345	6,037	4,893	1,14
C6orf106	Homo sapiens chromosome 6 open reading frame 106 (C6orf106), transcript variant 1, mRNA [NM_024294]	8,897	7,753	1,14
PCDHB14	Homo sapiens protocadherin beta 14 (PCDHB14), mRNA [NM_018934]	9,551	8,409	1,14
-	chr10:006608677-006608618	7,059	5,922	1,14
OBSCN	Homo sapiens obscurin, cytoskeletal calmodulin and titin-interacting RhoGEF (OBSCN), mRNA [NM_052843]	7,221	6,084	1,14
TIGD7	Homo sapiens tigger transposable element derived 7 (TIGD7), mRNA [NM_033208]	7,068	5,931	1,14
GOT2	Human mitochondrial aspartate aminotransferase mRNA, complete cds. [M22632]	9,643	8,507	1,14
C9orf114	Homo sapiens chromosome 9 open reading frame 114 (C9orf114), mRNA [NM_016390]	7,771	6,635	1,14
DST	Homo sapiens dystonin (DST), transcript variant 1eA, mRNA [NM_015548]	8,423	7,288	1,14

Supplemental Table 1
Morandi et al

ANKRD50	Homo sapiens ankyrin repeat domain 50 (ANKRD50), mRNA [NM_020337]	7,106	5,972	1,14
CDKL5	Homo sapiens cyclin-dependent kinase-like 5 (CDKL5), transcript variant I, mRNA [NM_003159]	11,244	10,109	1,14
-	chr10:124887537-124887599	5,98	4,845	1,14
MUC6	Homo sapiens cDNA FLJ39453 fis, clone PROST2010046, highly similar to Homo sapiens secretory mucin MUC6 (MUC6) mRNA. [AK096772]	7,385	6,251	1,13
LOC146517	PREDICTED: Homo sapiens hypothetical protein LOC146517 (LOC146517), mRNA [XM_928464]	5,642	4,508	1,13
TRIM7	Homo sapiens tripartite motif-containing 7 (TRIM7), transcript variant 6, mRNA [NM_033342]	6,085	4,953	1,13
ANKZF1	Homo sapiens ankyrin repeat and zinc finger domain containing 1 (ANKZF1), transcript variant 1, mRNA [NM_018089]	9,688	8,558	1,13
THC2697920	chr2:29071099-29071158	5,576	4,445	1,13
ANKRD12	Homo sapiens ankyrin repeat domain 12 (ANKRD12), mRNA [NM_015208]	11,017	9,886	1,13
EMILIN3	Homo sapiens elastin microfibril interfacer 3 (EMILIN3), mRNA [NM_052846]	7,203	6,073	1,13
C1QDC1	Homo sapiens C1q domain containing 1 (C1QDC1), transcript variant 2, mRNA [NM_023925]	7,922	6,796	1,13
THC2699738	BC031068 AADAT protein {Homo sapiens} (exp=-1; wgp=0; cg=0), partial (8%) [THC2699738]	5,772	4,645	1,13
KIF13B	Homo sapiens kinesin family member 13B (KIF13B), mRNA [NM_015254]	11,891	10,765	1,13
RTN4RL2	Homo sapiens reticulon 4 receptor-like 2 (RTN4RL2), mRNA [NM_178570]	6,981	5,855	1,13
THC2597357	Q6MG17_RAT (Q6MG17) Flotillin 1, partial (54%) [THC2597357]	9,218	8,097	1,12
LOC286526	Homo sapiens Ras-like GTPase-like (LOC286526), mRNA [NM_001031834]	7,443	6,323	1,12
GPR68	Homo sapiens G protein-coupled receptor 68 (GPR68), mRNA [NM_003485]	5,509	4,39	1,12
HDHD3	Homo sapiens haloacid dehalogenase-like hydrolase domain containing 3 (HDHD3), mRNA [NM_031219]	6,078	4,959	1,12
DGCR2	Homo sapiens DiGeorge syndrome critical region gene 2 (DGCR2), mRNA [NM_005137]	13,319	12,2	1,12
TBCD	Homo sapiens tubulin folding cofactor D (TBCD), mRNA [NM_005993]	10,929	9,811	1,12
DLG2	Homo sapiens discs, large homolog 2, chapsyn-110 (Drosophila) (DLG2), mRNA [NM_001364]	6,228	5,111	1,12
BC011942	Homo sapiens cDNA clone IMAGE:4336144, partial cds. [BC011942]	7,081	5,965	1,12
TBC1D16	Homo sapiens mRNA; cDNA DKFZp451F1311 (from clone DKFZp451F1311). [CR936670]	11,408	10,293	1,12
-	chr18:011624446-011624505	5,573	4,458	1,12
ELF5	Homo sapiens cDNA FLJ90152 fis, clone HEMBB1002051, weakly similar to Homo sapiens Ets transcription factor ESE-2b mRNA. [AK074633]	6,415	5,301	1,11
C17orf58	Homo sapiens chromosome 17 open reading frame 58 (C17orf58), transcript variant 1, mRNA [NM_181655]	8,487	7,374	1,11
PEMT	Homo sapiens, clone IMAGE:3030210, mRNA, partial cds. [BC007572]	7,506	6,393	1,11
CR610885	full-length cDNA clone CS0DC019YC18 of Neuroblastoma Cot 25-normalized of Homo sapiens (human). [CR610885]	8,51	7,397	1,11
JAK3	Homo sapiens Janus kinase 3 (a protein tyrosine kinase, leukocyte) (JAK3), mRNA [NM_000215]	7,006	5,894	1,11
BC039457	Homo sapiens cDNA clone IMAGE:5312122. [BC039457]	11,743	10,632	1,11
GRB2	Homo sapiens growth factor receptor-bound protein 2 (GRB2), transcript variant 1, mRNA [NM_002086]	11,449	10,338	1,11
PARD3	Homo sapiens atypical PKC isotype-specific interacting protein long variant mRNA, complete cds. [AF196185]	8,563	7,454	1,11
PRCD	Homo sapiens progressive rod-cone degeneration (PRCD), mRNA [NM_001077620]	6,347	5,238	1,11
VGLL4	Homo sapiens vestigial like 4 (Drosophila) (VGLL4), mRNA [NM_014667]	11,374	10,265	1,11

Supplemental Table 1
Morandi et al

ADCY3	Homo sapiens adenylate cyclase 3 (ADCY3), mRNA [NM_004036]	12,818	11,711	1,11
KIAA0146	full-length cDNA clone CS0DK002YC19 of HeLa cells Cot 25-normalized of Homo sapiens (human). [CR614430]	9,782	8,676	1,11
CDC42BPA	Homo sapiens CDC42 binding protein kinase alpha (DMPK-like) (CDC42BPA), transcript variant A, mRNA [NM_014826]	9,748	8,645	1,10
FLJ35220	Homo sapiens hypothetical protein FLJ35220 (FLJ35220), mRNA [NM_173627]	9,268	8,167	1,10
IGSF8	Homo sapiens immunoglobulin superfamily, member 8 (IGSF8), mRNA [NM_052868]	9,158	8,056	1,10
OLIG1	Homo sapiens oligodendrocyte transcription factor 1 (OLIG1), mRNA [NM_138983]	5,381	4,28	1,10
SYT2	Homo sapiens synaptotagmin II (SYT2), mRNA [NM_177402]	5,732	4,633	1,10
THC2548256	chr3:75547461-75547402	6,251	5,152	1,10
KIAA0280	Human mRNA for KIAA0280 gene, partial cds. [D87470]	8,749	7,655	1,09
CLCN3	Homo sapiens chloride channel 3 (CLCN3), transcript variant b, mRNA [NM_001829]	8,38	7,288	1,09
MAPK11	Homo sapiens mitogen-activated protein kinase 11 (MAPK11), mRNA [NM_002751]	12,467	11,376	1,09
SHFM3P1	Homo sapiens F-box protein Fbw3 (FBW3) mRNA, complete cds. [AF174606]	8,159	7,071	1,09
CRYBB3	Homo sapiens crystallin, beta B3 (CRYBB3), mRNA [NM_004076]	6,846	5,758	1,09
FLJ40113	Homo sapiens golgi autoantigen, golgin subfamily a-like pseudogene (FLJ40113) on chromosome 15 [NR_003246]	7,714	6,629	1,09
RHEBL1	Homo sapiens Ras homolog enriched in brain like 1 (RHEBL1), mRNA [NM_144593]	6,97	5,888	1,08
DNAL4	Homo sapiens dynein, axonemal, light chain 4 (DNAL4), mRNA [NM_005740]	7,866	6,784	1,08
BG151149	BG151149 nai34c03.y1 NCI_CGAP_HN20 Homo sapiens cDNA clone IMAGE:4261997 5' similar to contains Alu repetitive element;, mRNA sequence [BG151149]	6,15	5,07	1,08
KIAA0828	Homo sapiens adenosylhomocysteinase 3 (KIAA0828), mRNA [NM_015328]	9,802	8,724	1,08
LOC134357	PREDICTED: Homo sapiens similar to aconitase 2 precursor (LOC134357), mRNA [XR_018355]	9,29	8,213	1,08
BCDIN3	Homo sapiens bin3, bicoid-interacting 3, homolog (Drosophila) (BCDIN3), mRNA [NM_019606]	8,933	7,855	1,08
LOC552889	Homo sapiens cDNA FLJ32080 fis, clone OCBBF2000015. [AK056642]	10,224	9,147	1,08
ENST00000383285	NCTR3_HUMAN Isoform 3 of O14931 - Homo sapiens (Human) [Source:Uniprot/Varsplic;Acc:O14931-3] [ENST00000383285]	5,558	4,482	1,08
SETX	Homo sapiens senataxin (SETX), mRNA [NM_015046]	10,19	9,114	1,08
PLEKHG6	Homo sapiens pleckstrin homology domain containing, family G (with RhoGef domain) member 6 (PLEKHG6), mRNA [NM_018173]	5,427	4,354	1,07
RFK	Homo sapiens riboflavin kinase (RFK), mRNA [NM_018339]	10,301	9,227	1,07
BM725480	BM725480 UI-E-EJ0-aie-p-22-0-UI.r1 UI-E-EJ0 Homo sapiens cDNA clone UI-E-EJ0-aie-p-22-0-UI 5', mRNA sequence [BM725480]	9,378	8,305	1,07
MOGAT3	Homo sapiens monoacylglycerol O-acyltransferase 3 (MOGAT3), mRNA [NM_178176]	5,629	4,556	1,07
-	chr3:150858500-150858559	5,669	4,596	1,07
CYP3A43	Homo sapiens cytochrome P450, family 3, subfamily A, polypeptide 43 (CYP3A43), transcript variant 3, mRNA [NM_057096]	5,37	4,299	1,07
DNAJA4	Homo sapiens DnaJ (Hsp40) homolog, subfamily A, member 4 (DNAJA4), mRNA [NM_018602]	8,629	7,558	1,07
KCTD15	Homo sapiens potassium channel tetramerisation domain containing 15 (KCTD15), mRNA [NM_024076]	9,03	7,961	1,07
PPYR1	Homo sapiens pancreatic polypeptide receptor 1 (PPYR1), mRNA [NM_005972]	5,761	4,692	1,07
ZNF589	Homo sapiens zinc finger protein 589 (ZNF589), mRNA [NM_016089]	8,878	7,809	1,07
ENST00000333310	CDNA FLJ12567 fis, clone NT2RM4000855. [Source:Uniprot/SPTREMBL;Acc:Q9H9T1] [ENST00000333310]	9,332	8,266	1,07

CR590302	full-length cDNA clone CS0DF019YL13 of Fetal brain of Homo sapiens (human). [CR590302]	10,898	9,832	1,07
LOC441964	PREDICTED: Homo sapiens similar to sperm protein SSP411 (LOC441964), mRNA [XM_497783]	8,616	7,551	1,07
D2HGDH	Homo sapiens D-2-hydroxyglutarate dehydrogenase, mRNA (cDNA clone MGC:87530 IMAGE:30334929), complete cds. [BC071598]	6,074	5,011	1,06
ARHGEF4	Homo sapiens Rho guanine nucleotide exchange factor (GEF) 4 (ARHGEF4), transcript variant 1, mRNA [NM_015320]	6,299	5,237	1,06
TOB2	Homo sapiens transducer of ERBB2, 2 (TOB2), mRNA [NM_016272]	9,905	8,843	1,06
ST13	Homo sapiens suppression of tumorigenicity 13 (colon carcinoma) (Hsp70 interacting protein) (ST13), mRNA [NM_003932]	11,699	10,639	1,06
LCE1F	Homo sapiens late cornified envelope 1F (LCE1F), mRNA [NM_178354]	7,025	5,965	1,06
AK096566	Homo sapiens cDNA FLJ39247 fis, clone OCBBF2008520. [AK096566]	5,618	4,559	1,06
UBE2J2	Homo sapiens ubiquitin-conjugating enzyme E2, J2 (UBC6 homolog, yeast) (UBE2J2), transcript variant 3, mRNA [NM_194458]	7,913	6,855	1,06
PCYT2	Homo sapiens phosphate cytidylyltransferase 2, ethanolamine (PCYT2), mRNA [NM_002861]	8,968	7,912	1,06
FBXW11	Homo sapiens F-box and WD-40 domain protein 11 (FBXW11), transcript variant 3, mRNA [NM_012300]	12,147	11,093	1,05
BCAP31	Homo sapiens B-cell receptor-associated protein 31 (BCAP31), mRNA [NM_005745]	11,348	10,295	1,05
MAGI3	Homo sapiens membrane associated guanylate kinase, WW and PDZ domain containing 3 (MAGI3), transcript variant 1, mRNA [NM_020965]	7,424	6,372	1,05
CHST4	Homo sapiens carbohydrate (N-acetylglucosamine 6-O) sulfotransferase 4 (CHST4), mRNA [NM_005769]	5,703	4,652	1,05
C7orf42	Homo sapiens chromosome 7 open reading frame 42 (C7orf42), mRNA [NM_017994]	13,21	12,16	1,05
LLGL2	Homo sapiens lethal giant larvae homolog 2 (Drosophila) (LLGL2), transcript variant 1, mRNA [NM_004524]	6,2	5,151	1,05
DLG5	Homo sapiens discs, large homolog 5 (Drosophila) (DLG5), mRNA [NM_004747]	11,031	9,985	1,05
TMEM9	Homo sapiens transmembrane protein 9 (TMEM9), mRNA [NM_016456]	11,817	10,772	1,05
CLCN4	Homo sapiens chloride channel 4 (CLCN4), mRNA [NM_001830]	6,738	5,692	1,05
HCG8	Homo sapiens clone DNA165054 PAMP6501 (UNQ6501) mRNA, complete cds. [AY358246]	5,781	4,736	1,05
TERF2IP	Homo sapiens telomeric repeat binding factor 2, interacting protein (TERF2IP), mRNA [NM_018975]	14,375	13,331	1,04
ANKRD17	Homo sapiens ankyrin repeat domain 17 (ANKRD17), transcript variant 1, mRNA [NM_032217]	10,048	9,008	1,04
TULP4	Homo sapiens tubby like protein 4 (TULP4), transcript variant 1, mRNA [NM_020245]	12,499	11,459	1,04
LOC647768	PREDICTED: Homo sapiens similar to Tetratricopeptide repeat protein 3 (TPR repeat protein 3) (TPR repeat protein D) (LOC647768), mRNA [XR_018202]	11,866	10,828	1,04
-	chr6:127718761-127718820	10,976	9,938	1,04
SUPV3L1	Homo sapiens suppressor of var1, 3-like 1 (S. cerevisiae) (SUPV3L1), mRNA [NM_003171]	9,868	8,831	1,04
WWP1	Homo sapiens WW domain containing E3 ubiquitin protein ligase 1 (WWP1), mRNA [NM_007013]	8,946	7,913	1,03
THC2745287	Q6C3B8_YARLI (Q6C3B8) Similarity, partial (10%) [THC2745287]	6,971	5,938	1,03
NOL6	Homo sapiens nucleolar protein family 6 (RNA-associated) (NOL6), transcript variant alpha, mRNA [NM_022917]	6,203	5,171	1,03
ARF1	Homo sapiens ADP-ribosylation factor 1 (ARF1), transcript variant 1, mRNA [NM_001024227]	10,197	9,166	1,03
ARSD	Homo sapiens arylsulfatase D (ARSD), transcript variant 2, mRNA [NM_009589]	6,315	5,285	1,03
ZNF783	Homo sapiens zinc finger protein 783 (ZNF783), mRNA [NM_001004302]	9,931	8,903	1,03

Supplemental Table 1
Morandi et al

IPO8	Homo sapiens importin 8 (IPO8), mRNA [NM_006390]	6,004	4,976	1,03
ENST00000221462	Homo sapiens hypothetical protein LOC284352, mRNA (cDNA clone IMAGE:4779950), with apparent retained intron. [BC039061]	8,664	7,637	1,03
ZMYM3	Homo sapiens zinc finger, MYM-type 3 (ZMYM3), transcript variant 1, mRNA [NM_005096]	8,297	7,271	1,03
GALT	Homo sapiens galactose-1-phosphate uridylyltransferase (GALT), mRNA [NM_000155]	11,965	10,943	1,02
ZFYVE16	Zinc finger FYVE domain-containing protein 16 (Endofin) (Endosome-associated FYVE domain protein). [Source:Uniprot/SWISSPROT;Acc:Q7Z3T8] [ENST00000380248]	8,924	7,902	1,02
CXorf23	Homo sapiens chromosome X open reading frame 23 (CXorf23), mRNA [NM_198279]	6,436	5,417	1,02
CR598098	full-length cDNA clone CS0DI007YL24 of Placenta Cot 25-normalized of Homo sapiens (human). [CR598098]	8,155	7,139	1,02
AI942297	AI942297 wo79c01.x1 NCI_CGAP_Kid11 Homo sapiens cDNA clone IMAGE:2461536 3', mRNA sequence [AI942297]	5,49	4,478	1,01
LOC56757	Homo sapiens cDNA: FLJ23595 fis, clone LNG15262. [AK027248]	9,775	8,765	1,01
PRKAR1A	Homo sapiens protein kinase, cAMP-dependent, regulatory, type I, alpha (tissue specific extinguisher 1) (PRKAR1A), transcript variant 3, mRNA [NM_212472]	10,219	9,21	1,01
AP3M2	Homo sapiens adaptor-related protein complex 3, mu 2 subunit (AP3M2), mRNA [NM_006803]	10,461	9,453	1,01
LOC388284	Homo sapiens hypothetical gene supported by BC032064; BC041612, mRNA (cDNA clone IMAGE:5163879). [BC032064]	6,685	5,678	1,01
BLCAP	Homo sapiens bladder cancer associated protein (BLCAP), mRNA [NM_006698]	10,965	9,959	1,01
BEXL1	Homo sapiens cDNA FLJ10097 fis, clone HEMBA1002458, weakly similar to OVARIAN GRANULOSA CELL 13.0 KD PROTEIN HGR74. [AK000959]	12,203	11,2	1,00
LOC284402	Homo sapiens cDNA FLJ36176 fis, clone TESTI2026491. [AK093495]	6,046	5,044	1,00
THC2650367	chr6:31477038-31476979	5,95	4,946	1,00
FLJ23865	Homo sapiens cDNA FLJ23865 fis, clone LNG09225. [AK074445]	5,52	4,518	1,00
HD	Homo sapiens huntingtin (Huntington disease) (HD), mRNA [NM_002111]	11,219	10,221	1,00
SLC22A5	Homo sapiens solute carrier family 22 (organic cation transporter), member 5 (SLC22A5), mRNA [NM_003060]	9,679	8,682	1,00
LOC158960	Homo sapiens, clone IMAGE:3613029, mRNA, partial cds. [BC009467]	8,283	7,286	1,00
PQLC1	Homo sapiens PQ loop repeat containing 1 (PQLC1), mRNA [NM_025078]	9,667	8,673	0,99
RABGAP1	Homo sapiens RAB GTPase activating protein 1 (RABGAP1), mRNA [NM_012197]	10,07	9,076	0,99
DISP1	Homo sapiens dispatched homolog 1 (Drosophila) (DISP1), mRNA [NM_032890]	9,308	8,315	0,99
LOC400036	PREDICTED: Homo sapiens similar to DEAD (Asp-Glu-Ala-Asp) box polypeptide 23 (LOC400036), mRNA [XR_016505]	9,369	8,378	0,99
THC2519072	Q28V56_JANSC (Q28V56) Methyltransferase small, partial (6%) [THC2519072]	6,096	5,104	0,99
TMEM63B	Homo sapiens transmembrane protein 63B (TMEM63B), mRNA [NM_018426]	9,287	8,297	0,99
SDCCAG8	Homo sapiens serologically defined colon cancer antigen 8 (SDCCAG8), mRNA [NM_006642]	7,689	6,701	0,99
LOC729173	Homo sapiens cDNA FLJ39662 fis, clone SMINT2007140. [AK096981]	5,62	4,632	0,99
BQ880155	AGENCOURT_7981024 Lupski_dorsal_root_ganglion Homo sapiens cDNA clone IMAGE:6185670 5', mRNA sequence [BQ880155]	5,577	4,59	0,99
ENST00000324808	coiled-coil domain containing 57 [Source:RefSeq_peptide;Acc:NP_932348] [ENST00000324808]	6,694	5,707	0,99
ENST00000291567	Glucose-6-phosphate 1-dehydrogenase (EC 1.1.1.49) (G6PD). [Source:Uniprot/SWISSPROT;Acc:P11413] [ENST00000291567]	5,719	4,734	0,99

Supplemental Table 1
Morandi et al

LOC727768	Homo sapiens cDNA FLJ39633 fis, clone SMINT2002457, weakly similar to Tektin A1. [AK096952]	5,701	4,721	0,98
KRT85	Homo sapiens keratin 85 (KRT85), mRNA [NM_002283]	6,362	5,383	0,98
IKZF4	Homo sapiens mRNA; cDNA DKFZp686K2231 (from clone DKFZp686K2231). [BX647761]	8,846	7,867	0,98
FAM109A	Homo sapiens family with sequence similarity 109, member A (FAM109A), mRNA [NM_144671]	8,623	7,648	0,98
AGPAT7	Homo sapiens 1-acylglycerol-3-phosphate O-acyltransferase 7 (lysophosphatidic acid acyltransferase, eta) (AGPAT7), mRNA [NM_153613]	8,409	7,434	0,98
SETD4	Homo sapiens SET domain containing 4 (SETD4), transcript variant 1, mRNA [NM_017438]	8,95	7,975	0,98
PNPO	Homo sapiens pyridoxamine 5'-phosphate oxidase (PNPO), mRNA [NM_018129]	6,148	5,175	0,97
NP285481	GB|AJ278917.1|CAC07210.1 putative ATP-binding cassette protein [Homo sapiens] [NP285481]	5,728	4,757	0,97
MYEOV	Homo sapiens myeloma overexpressed gene (in a subset of t(11;14) positive multiple myelomas) (MYEOV), mRNA [NM_138768]	6,398	5,426	0,97
C20orf117	Homo sapiens cDNA FLJ32494 fis, clone SKNSH2000151. [AK057056]	9,83	8,861	0,97
COASY	Homo sapiens Coenzyme A synthase (COASY), transcript variant 1, mRNA [NM_025233]	11,821	10,854	0,97
LYPLA2P1	Homo sapiens lysophospholipase II pseudogene 1 (LYPLA2P1) on chromosome 6 [NR_001444]	8,315	7,348	0,97
BU625797	UI-H-FG1-bgo-l-02-0-UI.s1 NCI_CGAP_FG1 Homo sapiens cDNA clone UI-H-FG1-bgo-l-02-0-UI 3', mRNA sequence [BU625797]	6,765	5,798	0,97
NAT8B	Homo sapiens N-acetyltransferase 8B (gene/pseudogene) (NAT8B), mRNA [NM_016347]	5,765	4,798	0,97
CLASP1	Homo sapiens cytoplasmic linker associated protein 1 (CLASP1), mRNA [NM_015282]	10,21	9,245	0,97
ENST00000255741	GTP binding protein (Fragment). [Source:Uniprot/SPTREMBL;Acc:Q06976] [ENST00000255741]	10,192	9,231	0,96
BC010535	Homo sapiens cDNA clone IMAGE:3456494, partial cds. [BC010535]	8,14	7,181	0,96
NKX2-5	Homo sapiens NK2 transcription factor related, locus 5 (Drosophila) (NKX2-5), mRNA [NM_004387]	7,452	6,495	0,96
RIPK4	Homo sapiens receptor-interacting serine-threonine kinase 4 (RIPK4), mRNA [NM_020639]	5,552	4,596	0,96
ASB16	Homo sapiens ankyrin repeat and SOCS box-containing 16 (ASB16), mRNA [NM_080863]	6,937	5,982	0,96
SLC22A7	Homo sapiens solute carrier family 22 (organic anion transporter), member 7 (SLC22A7), transcript variant 2, mRNA [NM_153320]	6,458	5,503	0,96
PIK3R4	Homo sapiens phosphoinositide-3-kinase, regulatory subunit 4, p150 (PIK3R4), mRNA [NM_014602]	8,441	7,486	0,95
AP3D1	Homo sapiens adaptor-related protein complex 3, delta 1 subunit (AP3D1), transcript variant 2, mRNA [NM_003938]	10,565	9,62	0,95
RALA	Homo sapiens v-ral simian leukemia viral oncogene homolog A (ras related) (RALA), mRNA [NM_005402]	10,51	9,567	0,94
YLPM1	Homo sapiens YLP motif containing 1, mRNA (cDNA clone IMAGE:3835908), complete cds. [BC023570]	11,695	10,763	0,93
AP2A2	Homo sapiens adaptor-related protein complex 2, alpha 2 subunit (AP2A2), mRNA [NM_012305]	9,261	8,334	0,93
ENST00000379877	Full-length cDNA clone CS0DL004YM19 of B cells (Ramos cell line) of Homo sapiens (human) (Fragment). [Source:Uniprot/SPTREMBL;Acc:Q86SX2] [ENST00000379877]	5,96	5,035	0,93
FTSJ3	Homo sapiens FtsJ homolog 3 (E. coli) (FTSJ3), mRNA [NM_017647]	11,813	10,89	0,92
PCFT	Homo sapiens proton-coupled folate transporter (PCFT), mRNA [NM_080669]	6,789	5,867	0,92
APTX	Homo sapiens aprataxin (APTX), transcript variant 1, mRNA [NM_175073]	10,368	9,446	0,92
MGC10334	Homo sapiens hypothetical protein MGC10334 (MGC10334), mRNA [NM_001029885]	6,357	5,438	0,92

FLJ30092	Homo sapiens mRNA for KIAA0614 protein, partial cds. [AB014514]	11,113	10,199	0,91
DDHD2	DDHD domain containing 2 [Source:RefSeq_peptide;Acc:NP_056029] [ENST00000319246]	11,048	10,134	0,91
PAX5	Human B cell specific activator protein BSAP (PAX5) mRNA, partial cds. [U62539]	5,994	5,081	0,91
ZNF341	Homo sapiens zinc finger protein 341 (ZNF341), mRNA [NM_032819]	6,679	5,766	0,91
GBF1	Homo sapiens golgi-specific brefeldin A resistance factor 1 (GBF1), mRNA [NM_004193]	11,607	10,695	0,91
DHRS1	Homo sapiens dehydrogenase/reductase (SDR family) member 1 (DHRS1), mRNA [NM_138452]	10,209	9,299	0,91
RAB5B	Homo sapiens RAB5B, member RAS oncogene family (RAB5B), mRNA [NM_002868]	9,501	8,591	0,91
WDR59	Homo sapiens WD repeat domain 59 (WDR59), mRNA [NM_030581]	10,786	9,877	0,91
PATZ1	Homo sapiens POZ (BTB) and AT hook containing zinc finger 1 (PATZ1), transcript variant 4, mRNA [NM_032051]	7,132	6,224	0,91
RUVBL1	Homo sapiens RuvB-like 1 (E. coli) (RUVBL1), mRNA [NM_003707]	5,791	4,886	0,91
C17orf62	Homo sapiens chromosome 17 open reading frame 62 (C17orf62), mRNA [NM_001033046]	10,791	9,89	0,90
COG1	Homo sapiens component of oligomeric golgi complex 1 (COG1), mRNA [NM_018714]	11,621	10,721	0,90
GPR77	Homo sapiens G protein-coupled receptor 77 (GPR77), mRNA [NM_018485]	5,775	4,876	0,90
C22orf25	Homo sapiens mRNA; cDNA DKFZp686O2142 (from clone DKFZp686O2142). [CR627452]	6,061	5,163	0,90
DDX54	Homo sapiens DEAD (Asp-Glu-Ala-Asp) box polypeptide 54 (DDX54), mRNA [NM_024072]	9,437	8,54	0,90
SART3	Homo sapiens squamous cell carcinoma antigen recognized by T cells 3 (SART3), mRNA [NM_014706]	10,087	9,19	0,90
ATXN7L3	Homo sapiens ataxin 7-like 3, mRNA (cDNA clone IMAGE:5499446), partial cds. [BC037418]	9,516	8,622	0,89
THC2669419	ALU7_HUMAN (P39194) Alu subfamily SQ sequence contamination warning entry, partial (13%) [THC2669419]	10,06	9,17	0,89
CDS2	Homo sapiens CDP-diacylglycerol synthase (phosphatidate cytidylyltransferase) 2 (CDS2), mRNA [NM_003818]	10,249	9,36	0,89
LY6D	Homo sapiens lymphocyte antigen 6 complex, locus D (LY6D), mRNA [NM_003695]	5,323	4,434	0,89
ZNF289	Homo sapiens zinc finger protein 289, ID1 regulated (ZNF289), mRNA [NM_032389]	8,878	7,991	0,89
ATG4B	Homo sapiens ATG4 autophagy related 4 homolog B (S. cerevisiae) (ATG4B), transcript variant 2, mRNA [NM_178326]	12,028	11,142	0,89
SOX21	Homo sapiens SRY (sex determining region Y)-box 21 (SOX21), mRNA [NM_007084]	6,549	5,669	0,88
ALDH6A1	Homo sapiens aldehyde dehydrogenase 6 family, member A1 (ALDH6A1), nuclear gene encoding mitochondrial protein, mRNA [NM_005589]	9,282	8,407	0,88
ENST00000273582	full-length cDNA clone CS0DI044YK15 of Placenta Cot 25-normalized of Homo sapiens (human). [CR601728]	11,687	10,818	0,87
LBH	Homo sapiens limb bud and heart development homolog (mouse) (LBH), mRNA [NM_030915]	10,819	9,958	0,86
KIAA1450	Homo sapiens mRNA for KIAA1450 protein, partial cds. [AB040883]	10,2	9,339	0,86
BF514513	UI-H-BW1-anf-g-07-0-UI.s1 NCI_CGAP_Sub7 Homo sapiens cDNA clone IMAGE:3082188 3', mRNA sequence [BF514513]	10,03	9,172	0,86
PAK1IP1	Homo sapiens PAK1 interacting protein 1 (PAK1IP1), mRNA [NM_017906]	10,64	9,784	0,86
PPIL2	Homo sapiens peptidylprolyl isomerase (cyclophilin)-like 2 (PPIL2), transcript variant 2, mRNA [NM_148175]	9,788	8,933	0,86
BC040412	Homo sapiens, clone IMAGE:5184855, mRNA. [BC040412]	7,746	6,891	0,86
CSNK2A2	Homo sapiens casein kinase 2, alpha prime polypeptide (CSNK2A2), mRNA [NM_001896]	9,289	8,437	0,85
BC022417	Homo sapiens cDNA clone IMAGE:4243782, partial cds. [BC022417]	10,299	9,447	0,85

Supplemental Table 1
Morandi et al

ARHGEF11	Homo sapiens Rho guanine nucleotide exchange factor (GEF) 11 (ARHGEF11), transcript variant 2, mRNA [NM_198236]	9,453	8,603	0,85
AXIN1	Homo sapiens axin 1 (AXIN1), transcript variant 1, mRNA [NM_003502]	8,724	7,884	0,84
REXO4	Homo sapiens REX4, RNA exonuclease 4 homolog (S. cerevisiae) (REXO4), mRNA [NM_020385]	9,318	8,482	0,84
SH2B1	Homo sapiens SH2B adaptor protein 1 (SH2B1), mRNA [NM_015503]	8,91	8,077	0,83
TRAF2	Homo sapiens TNF receptor-associated factor 2 (TRAF2), mRNA [NM_021138]	10,271	9,442	0,83
LOC343508	PREDICTED: Homo sapiens similar to aconitase 2 precursor (LOC343508), mRNA [XR_017446]	9,841	9,014	0,83
HSP90AB1	Homo sapiens heat shock protein 90kDa alpha (cytosolic), class B member 1 (HSP90AB1), mRNA [NM_007355]	13,803	12,979	0,82
IKBKB	Homo sapiens inhibitor of kappa light polypeptide gene enhancer in B-cells, kinase beta (IKBKB), mRNA [NM_001556]	5,831	5,013	0,82
SLC9A6	Homo sapiens solute carrier family 9 (sodium/hydrogen exchanger), member 6 (SLC9A6), transcript variant 1, mRNA [NM_001042537]	11,291	10,475	0,82
CEP164	Homo sapiens centrosomal protein 164kDa (CEP164), mRNA [NM_014956]	9,414	8,598	0,82
THC2647660	chr16:82517108-82517167	5,635	4,823	0,81
CROCC	Homo sapiens ciliary rootlet coiled-coil, rootletin (CROCC), mRNA [NM_014675]	8,123	7,319	0,80
NUDT3	Homo sapiens nudix (nucleoside diphosphate linked moiety X)-type motif 3 (NUDT3), mRNA [NM_006703]	9,868	9,069	0,80
KLHL12	Homo sapiens kelch-like 12 (Drosophila) (KLHL12), mRNA [NM_021633]	10,728	9,933	0,80
DNAJC5	DnaJ homolog subfamily C member 5 (Cysteine string protein) (CSP). [Source:Uniprot/SWISSPROT;Acc:Q9H3Z4] [ENST00000369914]	10,037	9,247	0,79
BANP	Homo sapiens BTG3 associated nuclear protein (BANP), transcript variant 1, mRNA [NM_017869]	7,687	6,901	0,79
YWHAB	Homo sapiens tyrosine 3-monooxygenase/tryptophan 5-monooxygenase activation protein, beta polypeptide (YWHAB), transcript variant 1, mRNA [NM_003404]	14,015	13,233	0,78
CHMP7	Homo sapiens CHMP family, member 7 (CHMP7), mRNA [NM_152272]	10,128	9,346	0,78
AF086126	Homo sapiens full length insert cDNA clone ZA79F02. [AF086126]	11,66	10,88	0,78
C14orf172	Homo sapiens chromosome 14 open reading frame 172 (C14orf172), mRNA [NM_152307]	6,495	5,716	0,78
AW204925	UI-H-BI1-aer-a-07-0-UI.s1 NCI_CGAP_Sub3 Homo sapiens cDNA clone IMAGE:2720149 3', mRNA sequence [AW204925]	6,971	6,193	0,78
NFAT5	Homo sapiens nuclear factor of activated T-cells 5, tonicity-responsive (NFAT5), transcript variant 1, mRNA [NM_138714]	11,633	10,857	0,78
RUNDC2C	Homo sapiens RUN domain containing 2C (RUNDC2C) on chromosome 16 [NR_002939]	7,547	6,771	0,78
STK11IP	Homo sapiens cDNA FLJ34644 fis, clone KIDNE2017040. [AK091963]	7,603	6,85	0,75
ZNF446	Homo sapiens zinc finger protein 446 (ZNF446), mRNA [NM_017908]	7,071	6,32	0,75
KLHDC3	Homo sapiens kelch domain containing 3 (KLHDC3), mRNA [NM_057161]	9,626	8,877	0,75
HMHB1	Homo sapiens histocompatibility (minor) HB-1 (HMHB1), mRNA [NM_021182]	6,414	5,675	0,74
FAM73B	Homo sapiens family with sequence similarity 73, member B (FAM73B), mRNA [NM_032809]	8,653	7,933	0,72
GAPVD1	Homo sapiens GTPase activating protein and VPS9 domains 1 (GAPVD1), mRNA [NM_015635]	9,983	9,294	0,69
FAM20B	Homo sapiens family with sequence similarity 20, member B (FAM20B), mRNA [NM_014864]	13,16	12,507	0,65
CXXC1	Homo sapiens CXXC finger 1 (PHD domain) (CXXC1), mRNA [NM_014593]	10,191	9,547	0,64
CR622308	full-length cDNA clone CS0DI030YH01 of Placenta Cot 25-normalized of Homo sapiens (human). [CR622308]	11,867	11,242	0,63

Supplemental Table 1
Morandi et al

DHX36	Homo sapiens DEAH (Asp-Glu-Ala-His) box polypeptide 36 (DHX36), mRNA [NM_020865]	10,873	11,523	-0,65
BXDC5	Homo sapiens brix domain containing 5 (BXDC5), mRNA [NM_025065]	10,175	10,827	-0,65
PCBD2	Homo sapiens pterin-4 alpha-carbinolamine dehydratase/dimerization cofactor of hepatocyte nuclear factor 1 alpha (TCF1) 2 (PCBD2), mRNA [NM_032151]	8,481	9,158	-0,68
USP10	Homo sapiens ubiquitin specific peptidase 10 (USP10), mRNA [NM_005153]	9,647	10,353	-0,71
DDX20	Homo sapiens DEAD (Asp-Glu-Ala-Asp) box polypeptide 20 (DDX20), mRNA [NM_007204]	9,343	10,053	-0,71
C14orf153	Homo sapiens chromosome 14 open reading frame 153 (C14orf153), mRNA [NM_032374]	11,045	11,777	-0,73
RFWD2	Homo sapiens ring finger and WD repeat domain 2 (RFWD2), transcript variant 1, mRNA [NM_022457]	10,541	11,277	-0,74
CSE1L	Homo sapiens CSE1 chromosome segregation 1-like (yeast) (CSE1L), mRNA [NM_001316]	11,963	12,704	-0,74
PSMC4	Homo sapiens proteasome (prosome, macropain) 26S subunit, ATPase, 4 (PSMC4), transcript variant 1, mRNA [NM_006503]	10,97	11,717	-0,75
C16orf70	Homo sapiens chromosome 16 open reading frame 70 (C16orf70), mRNA [NM_025187]	9,308	10,063	-0,76
NME6	Homo sapiens non-metastatic cells 6, protein expressed in (nucleoside-diphosphate kinase) (NME6), mRNA [NM_005793]	7,796	8,559	-0,76
GTPBP10	Homo sapiens GTP-binding protein 10 (putative) (GTPBP10), transcript variant 2, mRNA [NM_033107]	9,091	9,855	-0,76
LARP2	Homo sapiens La ribonucleoprotein domain family, member 2 (LARP2), transcript variant 2, mRNA [NM_178043]	6,696	7,466	-0,77
C6orf66	Homo sapiens chromosome 6 open reading frame 66 (C6orf66), mRNA [NM_014165]	9,033	9,805	-0,77
ZNF639	Homo sapiens zinc finger protein 639 (ZNF639), mRNA [NM_016331]	8,56	9,33	-0,77
DRB1	Homo sapiens developmentally regulated RNA-binding protein 1 (DRB1), mRNA [NM_152945]	9,657	10,43	-0,77
LSM14A	Homo sapiens LSM14A, SCD6 homolog A (S. cerevisiae) (LSM14A), mRNA [NM_015578]	11,784	12,56	-0,78
NFYC	Homo sapiens nuclear transcription factor Y, gamma (NFYC), mRNA [NM_014223]	8,307	9,086	-0,78
TMEM14B	Homo sapiens transmembrane protein 14B (TMEM14B), mRNA [NM_030969]	11,776	12,568	-0,79
PRO1853	Homo sapiens hypothetical protein PRO1853 (PRO1853), transcript variant 2, mRNA [NM_018607]	9,825	10,622	-0,80
ENST0000023973(	Septin. [Source:Uniprot/SPTREMBL;Acc:Q5W160] [ENST00000239730]	7,204	8,006	-0,80
TMEM85	Homo sapiens transmembrane protein 85 (TMEM85), mRNA [NM_016454]	12,216	13,016	-0,80
C10orf84	Homo sapiens chromosome 10 open reading frame 84 (C10orf84), mRNA [NM_022063]	8,565	9,371	-0,81
C1orf124	Homo sapiens chromosome 1 open reading frame 124 (C1orf124), transcript variant 1, mRNA [NM_032018]	6,615	7,425	-0,81
NDUFV2	Homo sapiens NADH dehydrogenase (ubiquinone) flavoprotein 2, 24kDa (NDUFV2), mRNA [NM_021074]	12,151	12,965	-0,81
DDHD1	Homo sapiens DDHD domain containing 1 (DDHD1), mRNA [NM_030637]	6,12	6,938	-0,82
PMPCB	Homo sapiens peptidase (mitochondrial processing) beta (PMPCB), nuclear gene encoding mitochondrial protein, mRNA [NM_004279]	12,153	12,972	-0,82
C19orf53	Homo sapiens chromosome 19 open reading frame 53 (C19orf53), mRNA [NM_014047]	9,849	10,669	-0,82
KLHL18	Homo sapiens kelch-like 18 (Drosophila) (KLHL18), mRNA [NM_025010]	8,224	9,044	-0,82
ZNF8	Homo sapiens zinc finger protein 8 (ZNF8), mRNA [NM_021089]	8,153	8,975	-0,82
PRKRA	Homo sapiens protein kinase, interferon-inducible double stranded RNA dependent activator (PRKRA), mRNA [NM_003690]	10,684	11,508	-0,82
TXNL4B	Homo sapiens thioredoxin-like 4B (TXNL4B), mRNA [NM_017853]	6,773	7,603	-0,83

ZBTB41	Homo sapiens zinc finger and BTB domain containing 41 (ZBTB41), mRNA [NM_194314]	4,19	5,025	-0,84
CEP57	Homo sapiens centrosomal protein 57kDa (CEP57), mRNA [NM_014679]	8,883	9,721	-0,84
MRPL47	Homo sapiens mitochondrial ribosomal protein L47 (MRPL47), nuclear gene encoding mitochondrial protein, transcript variant 1, mRNA [NM_020409]	11,396	12,237	-0,84
-	chr1:015390989-015390926	9,534	10,377	-0,84
TIMM50	Homo sapiens translocase of inner mitochondrial membrane 50 homolog (S. cerevisiae) (TIMM50), mRNA [NM_001001563]	10,462	11,305	-0,84
HNRPD	Homo sapiens heterogeneous nuclear ribonucleoprotein D (AU-rich element RNA binding protein 1, 37kDa) (HNRPD), transcript variant 1, mRNA [NM_031370]	8,907	9,754	-0,85
TIAL1	Homo sapiens TIA1 cytotoxic granule-associated RNA binding protein-like 1 (TIAL1), transcript variant 2, mRNA [NM_001033925]	9,411	10,26	-0,85
MLH1	Homo sapiens mutL homolog 1, colon cancer, nonpolyposis type 2 (E. coli) (MLH1), mRNA [NM_000249]	12,12	12,979	-0,86
C4orf14	Homo sapiens chromosome 4 open reading frame 14 (C4orf14), mRNA [NM_032313]	10,46	11,322	-0,86
MRPL44	Homo sapiens mitochondrial ribosomal protein L44 (MRPL44), nuclear gene encoding mitochondrial protein, mRNA [NM_022915]	9,81	10,675	-0,86
MRPL32	Homo sapiens mitochondrial ribosomal protein L32 (MRPL32), nuclear gene encoding mitochondrial protein, mRNA [NM_031903]	13,055	13,92	-0,87
TSN	Homo sapiens translin (TSN), mRNA [NM_004622]	10,115	10,98	-0,87
NGLY1	Homo sapiens N-glycanase 1 (NGLY1), mRNA [NM_018297]	11,371	12,237	-0,87
TXN	Homo sapiens thioredoxin (TXN), mRNA [NM_003329]	12,862	13,729	-0,87
PPWD1	Homo sapiens peptidylprolyl isomerase domain and WD repeat containing 1 (PPWD1), mRNA [NM_015342]	10,294	11,165	-0,87
FBXL12	Homo sapiens F-box and leucine-rich repeat protein 12 (FBXL12), mRNA [NM_017703]	10,016	10,889	-0,87
CLPX	Homo sapiens ClpX caseinolytic peptidase X homolog (E. coli) (CLPX), mRNA [NM_006660]	9,702	10,577	-0,88
C1orf174	Homo sapiens chromosome 1 open reading frame 174 (C1orf174), mRNA [NM_207356]	11,257	12,134	-0,88
PXMP4	Homo sapiens peroxisomal membrane protein 4, 24kDa (PXMP4), transcript variant 1, mRNA [NM_007238]	7,636	8,515	-0,88
PPFIA1	Homo sapiens protein tyrosine phosphatase, receptor type, f polypeptide (PTPRF), interacting protein (liprin), alpha 1 (PPFIA1), transcript variant 1, mRNA [NM_177423]	7,361	8,247	-0,89
METAP2	Homo sapiens methionyl aminopeptidase 2 (METAP2), mRNA [NM_006838]	12,889	13,776	-0,89
BM979607	BM979607 UI-CF-DU1-adt-k-11-0-UI.s1 UI-CF-DU1 Homo sapiens cDNA clone UI-CF-DU1-adt-k-11-0-UI 3', mRNA sequence [BM979607]	7,415	8,304	-0,89
METTL6	Homo sapiens methyltransferase like 6 (METTL6), mRNA [NM_152396]	8,511	9,399	-0,89
ING2	Homo sapiens inhibitor of growth family, member 2 (ING2), mRNA [NM_001564]	9,538	10,427	-0,89
BQ014494	BQ014494 UI-H-ED1-axt-o-17-0-UI.s1 NCI_CGAP_ED1 Homo sapiens cDNA clone IMAGE:5833576 3', mRNA sequence [BQ014494]	5,105	6,003	-0,90
HDAC8	Homo sapiens histone deacetylase 8 (HDAC8), mRNA [NM_018486]	7,743	8,641	-0,90
TIMM17A	Homo sapiens translocase of inner mitochondrial membrane 17 homolog A (yeast) (TIMM17A), mRNA [NM_006335]	12,746	13,646	-0,90
ARD1A	Homo sapiens ARD1 homolog A, N-acetyltransferase (S. cerevisiae) (ARD1A), mRNA [NM_003491]	9,958	10,861	-0,90
C10orf78	Homo sapiens chromosome 10 open reading frame 78 (C10orf78), transcript variant 2, mRNA [NM_145247]	8,327	9,231	-0,90
DNTTIP1	Homo sapiens deoxynucleotidyltransferase, terminal, interacting protein 1 (DNTTIP1), mRNA [NM_052951]	11,435	12,34	-0,91

Supplemental Table 1
Morandi et al

LZIC	Homo sapiens leucine zipper and CTNNBIP1 domain containing (LZIC), mRNA [NM_032368]	10,857	11,762	-0,91
AK2	Homo sapiens adenylate kinase 2 (AK2), transcript variant AK2B, mRNA [NM_013411]	9,744	10,65	-0,91
CHCHD5	Homo sapiens coiled-coil-helix-coiled-coil-helix domain containing 5 (CHCHD5), mRNA [NM_032309]	8,839	9,746	-0,91
ETNK1	Homo sapiens ethanolamine kinase 1 (ETNK1), transcript variant 1, mRNA [NM_018638]	7,362	8,27	-0,91
FBXO9	Homo sapiens F-box protein 9 (FBXO9), transcript variant 2, mRNA [NM_033480]	11,144	12,053	-0,91
FAM35A	Homo sapiens family with sequence similarity 35, member A (FAM35A), mRNA [NM_019054]	5,979	6,889	-0,91
SWS1	Homo sapiens SWIM-domain containing Srs2 interacting protein 1 (SWS1), transcript variant 2, mRNA [NM_001042698]	10,29	11,203	-0,91
CTDSPL2	Homo sapiens CTD (carboxy-terminal domain, RNA polymerase II, polypeptide A) small phosphatase like 2 (CTDSPL2), mRNA [NM_016396]	8,018	8,934	-0,92
SEPHS1	Homo sapiens selenophosphate synthetase 1 (SEPHS1), mRNA [NM_012247]	10,633	11,55	-0,92
RCBTB1	Homo sapiens regulator of chromosome condensation (RCC1) and BTB (POZ) domain containing protein 1 (RCBTB1), mRNA [NM_018191]	5,462	6,381	-0,92
ALG9	Homo sapiens asparagine-linked glycosylation 9 homolog (S. cerevisiae, alpha- 1,2-mannosyltransferase) (ALG9), transcript variant 3, mRNA [NM_001077691]	8,591	9,511	-0,92
ACN9	Homo sapiens ACN9 homolog (S. cerevisiae) (ACN9), mRNA [NM_020186]	9,718	10,64	-0,92
OTUB1	Homo sapiens OTU domain, ubiquitin aldehyde binding 1 (OTUB1), transcript variant 1, mRNA [NM_017670]	8,457	9,379	-0,92
WDR36	Homo sapiens WD repeat domain 36 (WDR36), mRNA [NM_139281]	7,543	8,469	-0,93
THC2468883	ACL6A_HUMAN (O96019) Actin-like protein 6A (53 kDa BRG1-associated factor A) (Actin-related protein Baf53a) (ArpNbeta), partial (83%) [THC2468883]	5,576	6,503	-0,93
DNAJB11	Homo sapiens DnaJ (Hsp40) homolog, subfamily B, member 11 (DNAJB11), mRNA [NM_016306]	11,552	12,479	-0,93
ZNF586	Homo sapiens zinc finger protein 586 (ZNF586), transcript variant 1, mRNA [NM_017652]	5,598	6,525	-0,93
PSMB3	Homo sapiens proteasome (prosome, macropain) subunit, beta type, 3 (PSMB3), mRNA [NM_002795]	11,896	12,825	-0,93
-	chr14:069422336-069422164	13,172	14,102	-0,93
PPP2R2A	Homo sapiens protein phosphatase 2 (formerly 2A), regulatory subunit B (PR 52), alpha isoform (PPP2R2A), mRNA [NM_002717]	9,826	10,756	-0,93
SF3B1	Homo sapiens splicing factor 3b, subunit 1, 155kDa (SF3B1), transcript variant 1, mRNA [NM_012433]	12,75	13,681	-0,93
NUDCD2	Homo sapiens NudC domain containing 2 (NUDCD2), mRNA [NM_145266]	11,153	12,084	-0,93
BUD31	Homo sapiens BUD31 homolog (yeast) (BUD31), mRNA [NM_003910]	11,329	12,261	-0,93
LOC390612	PREDICTED: Homo sapiens similar to ribosomal protein L18 (LOC390612), mRNA [XR_018903]	11,33	12,264	-0,93
MGC71993	Homo sapiens similar to DNA segment, Chr 11, Brigham & Womens Genetics 0434 expressed (MGC71993), mRNA [NM_001004333]	11,482	12,416	-0,93
ORMDL2	Homo sapiens ORM1-like 2 (S. cerevisiae) (ORMDL2), mRNA [NM_014182]	10,935	11,869	-0,93
PRPF18	Homo sapiens PRP18 pre-mRNA processing factor 18 homolog (S. cerevisiae) (PRPF18), mRNA [NM_003675]	10,696	11,63	-0,93
SNRPA	Homo sapiens small nuclear ribonucleoprotein polypeptide A (SNRPA), mRNA [NM_004596]	10,498	11,432	-0,93
TIPRL	Homo sapiens TIP41, TOR signalling pathway regulator-like (S. cerevisiae) (TIPRL), transcript variant 1, mRNA [NM_152902]	10,482	11,416	-0,93
MRPL53	Homo sapiens mitochondrial ribosomal protein L53 (MRPL53), nuclear gene encoding mitochondrial protein, mRNA [NM_053050]	12,567	13,503	-0,94

Supplemental Table 1
Morandi et al

RECQL	Homo sapiens RecQ protein-like (DNA helicase Q1-like) (RECQL), transcript variant 2, mRNA [NM_032941]	9,387	10,322	-0,94
ZNF558	Homo sapiens zinc finger protein 558 (ZNF558), mRNA [NM_144693]	8,93	9,868	-0,94
BTBD7	Homo sapiens BTB (POZ) domain containing 7 (BTBD7), transcript variant 2, mRNA [NM_018167]	6,744	7,683	-0,94
PAPOLA	Homo sapiens poly(A) polymerase alpha (PAPOLA), mRNA [NM_032632]	6,348	7,289	-0,94
LOC128977	Homo sapiens hypothetical protein LOC128977 (LOC128977), mRNA [NM_173793]	9,245	10,189	-0,94
THC2650296	RL18A_MOUSE (P62717) 60S ribosomal protein L18a, complete [THC2650296]	13,171	14,115	-0,94
UBE2I	Homo sapiens ubiquitin-conjugating enzyme E2I (UBC9 homolog, yeast) (UBE2I), transcript variant 2, mRNA [NM_194259]	12,801	13,746	-0,95
CDK7	Homo sapiens cyclin-dependent kinase 7 (MO15 homolog, Xenopus laevis, cdk-activating kinase) (CDK7), mRNA [NM_001799]	10,768	11,72	-0,95
IMPDH1	Human IMP dehydrogenase type 1 mRNA complete cds. [J05272]	5,357	6,31	-0,95
CDK5RAP2	Homo sapiens CDK5 regulatory subunit associated protein 2 (CDK5RAP2), transcript variant 1, mRNA [NM_018249]	10,142	11,096	-0,95
NUP88	Homo sapiens nucleoporin 88kDa (NUP88), mRNA [NM_002532]	10,449	11,403	-0,95
C9orf156	Homo sapiens chromosome 9 open reading frame 156 (C9orf156), mRNA [NM_016481]	8,872	9,827	-0,96
SDCCAG3	Homo sapiens serologically defined colon cancer antigen 3 (SDCCAG3), transcript variant 1, mRNA [NM_001039707]	10,41	11,371	-0,96
ZDHHC3	Homo sapiens zinc finger, DHHC-type containing 3 (ZDHHC3), mRNA [NM_016598]	7,842	8,803	-0,96
SEP15	Homo sapiens 15 kDa selenoprotein), transcript variant 1, mRNA [NM_004261]	12,561	13,525	-0,96
TMEM93	Homo sapiens transmembrane protein 93 (TMEM93), transcript variant 1, mRNA [NM_001014764]	11,975	12,938	-0,96
MGC21675	Homo sapiens hypothetical protein MGC21675 (MGC21675), mRNA [NM_052861]	7,186	8,151	-0,97
JTB	Homo sapiens jumping translocation breakpoint (JTB), mRNA [NM_006694]	12,932	13,9	-0,97
TXNL2	Homo sapiens thioredoxin-like 2 (TXNL2), mRNA [NM_006541]	10,819	11,791	-0,97
CPSF6	Homo sapiens cleavage and polyadenylation specific factor 6, 68kDa (CPSF6), mRNA [NM_007007]	9,115	10,09	-0,98
TFAM	Homo sapiens transcription factor A, mitochondrial (TFAM), mRNA [NM_003201]	9,361	10,338	-0,98
MTMR6	Homo sapiens myotubularin related protein 6 (MTMR6), mRNA [NM_004685]	6,384	7,363	-0,98
OSBPL8	Homo sapiens oxysterol binding protein-like 8 (OSBPL8), transcript variant 1, mRNA [NM_020841]	9,211	10,193	-0,98
TIMM9	Homo sapiens translocase of inner mitochondrial membrane 9 homolog (yeast) (TIMM9), mRNA [NM_012460]	11,269	12,251	-0,98
TBC1D7	Homo sapiens TBC1 domain family, member 7 (TBC1D7), mRNA [NM_016495]	10,709	11,692	-0,98
PPARBP	Homo sapiens PPAR binding protein, mRNA (cDNA clone IMAGE:4822636), complete cds. [BC060758]	8,519	9,503	-0,98
COPS8	Homo sapiens COP9 constitutive photomorphogenic homolog subunit 8 (Arabidopsis) (COPS8), transcript variant 2, mRNA [NM_198189]	10,396	11,381	-0,99
ABCB7	Homo sapiens ATP-binding cassette, sub-family B (MDR/TAP), member 7 (ABCB7), nuclear gene encoding mitochondrial protein, mRNA [NM_004299]	9,432	10,423	-0,99
PNRC1	Homo sapiens proline-rich nuclear receptor coactivator 1 (PNRC1), mRNA [NM_006813]	11,988	12,984	-1,00
PPP1CC	Homo sapiens protein phosphatase 1, catalytic subunit, gamma isoform (PPP1CC), mRNA [NM_002710]	11,177	12,175	-1,00
C17orf49	Homo sapiens chromosome 17 open reading frame 49 (C17orf49), mRNA [NM_174893]	10,703	11,701	-1,00
LOC643744	PREDICTED: Homo sapiens similar to D-PCa-2 protein isoform c (LOC643744), mRNA [XR_017206]	11,495	12,493	-1,00
UCHL3	Homo sapiens ubiquitin carboxyl-terminal esterase L3 (ubiquitin thiolesterase) (UCHL3), mRNA [NM_006002]	9,659	10,659	-1,00

UBA52	Homo sapiens ubiquitin A-52 residue ribosomal protein fusion product 1 (UBA52), transcript variant 1, mRNA [NM_001033930]	10,935	11,939	-1,00
LOC730556	PREDICTED: Homo sapiens similar to Coiled-coil-helix-coiled-coil-helix domain-containing protein 2 (HCV NS2 trans-regulated protein) (NS2TP) (LOC730556), mRNA [XR_015322]	12,777	13,783	-1,01
AFF4	Homo sapiens AF4/FMR2 family, member 4 (AFF4), mRNA [NM_014423]	6,563	7,57	-1,01
ZNF548	Homo sapiens zinc finger protein 548 (ZNF548), mRNA [NM_152909]	6,452	7,46	-1,01
BRP44	Homo sapiens brain protein 44 (BRP44), mRNA [NM_015415]	11,533	12,543	-1,01
HRB	Homo sapiens HIV-1 Rev binding protein (HRB), mRNA [NM_004504]	10,214	11,223	-1,01
LOC731937	PREDICTED: Homo sapiens similar to suppressor of initiator codon mutations, related sequence 1 (LOC731937), mRNA [XM_001131332]	11,539	12,548	-1,01
POLR2D	Homo sapiens polymerase (RNA) II (DNA directed) polypeptide D (POLR2D), mRNA [NM_004805]	9,508	10,519	-1,01
C1QBP	Homo sapiens complement component 1, q subcomponent binding protein (C1QBP), nuclear gene encoding mitochondrial protein, mRNA [NM_001212]	13,07	14,083	-1,01
MRPL54	Homo sapiens mitochondrial ribosomal protein L54 (MRPL54), nuclear gene encoding mitochondrial protein, mRNA [NM_172251]	10,511	11,524	-1,01
USP39	Homo sapiens ubiquitin specific peptidase 39 (USP39), mRNA [NM_006590]	12,913	13,928	-1,01
SPCS1	Homo sapiens signal peptidase complex subunit 1 homolog (S. cerevisiae) (SPCS1), mRNA [NM_014041]	11,333	12,349	-1,02
LOC649843	PREDICTED: Homo sapiens similar to cytoplasmic beta-actin (LOC649843), mRNA [XR_018979]	9,71	10,726	-1,02
BCAS2	Homo sapiens breast carcinoma amplified sequence 2 (BCAS2), mRNA [NM_005872]	10,632	11,65	-1,02
BQ008507	BQ008507 UI-H-ED1-ayk-d-19-0-UI.s1 NCI_CGAP_ED1 Homo sapiens cDNA clone IMAGE:5839842 3', mRNA sequence [BQ008507]	4,952	5,97	-1,02
ZNF780B	Homo sapiens zinc finger protein 780B (ZNF780B), mRNA [NM_001005851]	7,322	8,34	-1,02
ANP32E	Homo sapiens acidic (leucine-rich) nuclear phosphoprotein 32 family, member E (ANP32E), mRNA [NM_030920]	7,834	8,854	-1,02
TDG	Homo sapiens thymine-DNA glycosylase (TDG), mRNA [NM_003211]	11,277	12,297	-1,02
MED28	Homo sapiens tumor-related protein mRNA, complete cds. [AF317680]	8,373	9,395	-1,02
MGC70863	Homo sapiens similar to RPL23AP7 protein (MGC70863), transcript variant 1, mRNA [NM_203477]	12,295	13,318	-1,02
CCNK	Homo sapiens cyclin K (CCNK), mRNA [NM_003858]	8,182	9,207	-1,03
C17orf39	Homo sapiens chromosome 17 open reading frame 39 (C17orf39), mRNA [NM_024052]	7,173	8,199	-1,03
ZNF256	Homo sapiens zinc finger protein 256 (ZNF256), mRNA [NM_005773]	8,783	9,81	-1,03
LOC388524	Homo sapiens similar to Laminin receptor 1 (LOC388524), mRNA [NM_001005472]	12,299	13,326	-1,03
LOC643013	PREDICTED: Homo sapiens similar to laminin receptor 1 (ribosomal protein SA) (LOC643013), mRNA [XR_018155]	12,885	13,913	-1,03
YBX1	Homo sapiens Y box binding protein 1 (YBX1), mRNA [NM_004559]	12,238	13,267	-1,03
MGC13017	Homo sapiens similar to RIKEN cDNA A430101B06 gene (MGC13017), mRNA [NM_080656]	10,677	11,707	-1,03
MGC4093	Homo sapiens hypothetical protein MGC4093 (MGC4093), mRNA [NM_030578]	8,916	9,948	-1,03
ENST0000033249ε	chr8:33946689-33946630	10,385	11,419	-1,03
STX18	Homo sapiens syntaxin 18 (STX18), mRNA [NM_016930]	8,21	9,245	-1,04
PAPD4	Homo sapiens PAP associated domain containing 4 (PAPD4), mRNA [NM_173797]	6,813	7,85	-1,04
THC2592963	Q9BIU9_ARGTR (Q9BIU9) Flagelliform silk protein (Fragment), partial (4%) [THC2592963]	11,483	12,523	-1,04

Supplemental Table 1
Morandi et al

NCOA4	Homo sapiens nuclear receptor coactivator 4 (NCOA4), mRNA [NM_005437]	9,37	10,413	-1,04
CCDC72	Homo sapiens coiled-coil domain containing 72 (CCDC72), mRNA [NM_015933]	13,435	14,479	-1,04
HIGD2A	Homo sapiens HIG1 domain family, member 2A (HIGD2A), mRNA [NM_138820]	10,057	11,101	-1,04
UBE1L2	Homo sapiens ubiquitin-activating enzyme E1-like 2 (UBE1L2), mRNA [NM_018227]	6,464	7,508	-1,04
BOLA2	Homo sapiens bolA homolog 2 (E. coli) (BOLA2), transcript variant 2, mRNA [NM_001031833]	10,21	11,255	-1,05
ANKRD49	Homo sapiens ankyrin repeat domain 49 (ANKRD49), mRNA [NM_017704]	8,739	9,786	-1,05
CNOT1	Homo sapiens CCR4-NOT transcription complex, subunit 1 (CNOT1), transcript variant 2, mRNA [NM_206999]	5,516	6,563	-1,05
ARHGDIA	Homo sapiens Rho GDP dissociation inhibitor (GDI) alpha (ARHGDIA), mRNA [NM_004309]	7,511	8,559	-1,05
ASCC3	Homo sapiens activating signal cointegrator 1 complex subunit 3 (ASCC3), transcript variant 2, mRNA [NM_022091]	7,797	8,846	-1,05
LSM8	Homo sapiens LSM8 homolog, U6 small nuclear RNA associated (S. cerevisiae) (LSM8), mRNA [NM_016200]	11,073	12,122	-1,05
ANKRD13A	Homo sapiens ankyrin repeat domain 13A (ANKRD13A), mRNA [NM_033121]	9,923	10,973	-1,05
C7orf44	Homo sapiens cDNA clone IMAGE:3537461, **** WARNING: chimeric clone ****. [BC001743]	6,681	7,733	-1,05
CCNE1	Homo sapiens cyclin E1 (CCNE1), transcript variant 1, mRNA [NM_001238]	10,555	11,606	-1,05
BE932552	BE932552 QV3-HT0637-310800-319-h10 HT0637 Homo sapiens cDNA, mRNA sequence [BE932552]	4,897	5,949	-1,05
C20orf20	Homo sapiens chromosome 20 open reading frame 20 (C20orf20), mRNA [NM_018270]	6,815	7,87	-1,05
TIPIN	Homo sapiens TIMELESS interacting protein (TIPIN), mRNA [NM_017858]	9,362	10,418	-1,06
E2F3	Homo sapiens E2F transcription factor 3 (E2F3), mRNA [NM_001949]	10,368	11,426	-1,06
CGGBP1	Homo sapiens CGG triplet repeat binding protein 1 (CGGBP1), transcript variant 1, mRNA [NM_001008390]	10,846	11,905	-1,06
YY1	Homo sapiens YY1 transcription factor (YY1), mRNA [NM_003403]	10,402	11,462	-1,06
AA725860	AA725860 ai23f08.s1 Soares_testis_NHT Homo sapiens cDNA clone 1343655 3', mRNA sequence [AA725860]	5,299	6,359	-1,06
IREB2	Homo sapiens iron-responsive element binding protein 2 (IREB2), mRNA [NM_004136]	7,117	8,18	-1,06
ODF2	Homo sapiens outer dense fiber of sperm tails 2 (ODF2), transcript variant 1, mRNA [NM_002540]	9,313	10,378	-1,07
LOC344423	Homo sapiens cDNA FLJ42751 fis, clone BRAWH3000491, moderately similar to 40S ribosomal protein S12. [AK124741]	12,165	13,232	-1,07
LOC390791	TC102790 Human breast cancer tissue, large insert, pCMV expression library Homo sapiens cDNA clone TC102790 5' similar to Homo sapiens similar to peptidylprolyl isomerase A (cyclophilin A) (LOC125110), mRNA sequence [DN996556]	11,244	12,311	-1,07
DR1	Homo sapiens down-regulator of transcription 1, TBP-binding (negative cofactor 2) (DR1), mRNA [NM_001938]	8,498	9,566	-1,07
SFRS11	Homo sapiens splicing factor, arginine/serine-rich 11 (SFRS11), mRNA [NM_004768]	11,954	13,023	-1,07
GMPPB	Homo sapiens mRNA for KIAA1851 protein, partial cds. [AB058754]	7,64	8,712	-1,07
-	chrX:112652614-112652673	5,966	7,038	-1,07
FKBP1A	Homo sapiens FK506 binding protein 1A, 12kDa (FKBP1A), transcript variant 12A, mRNA [NM_054014]	8,176	9,247	-1,07
BC037740	Homo sapiens cDNA clone IMAGE:5263531. [BC037740]	8,885	9,958	-1,07
MCM6	Homo sapiens minichromosome maintenance deficient 6 homolog (S. cerevisiae) (MCM6), mRNA [NM_005915]	11,767	12,84	-1,07
-	chrX:065093402-065093339	8,167	9,242	-1,08
CHRAC1	Homo sapiens chromatin accessibility complex 1 (CHRAC1), mRNA [NM_017444]	7,603	8,68	-1,08

Supplemental Table 1
Morandi et al

ARIH1	Homo sapiens ariadne homolog, ubiquitin-conjugating enzyme E2 binding protein, 1 (Drosophila) (ARIH1), mRNA [NM_005744]	7,013	8,094	-1,08
ENST00000332696	similar to 60S ribosomal protein L23a (LOC644384), mRNA [Source:RefSeq_dna;Acc:XR_017413] [ENST00000332696]	12,614	13,695	-1,08
ING3	Homo sapiens inhibitor of growth family, member 3 (ING3), transcript variant 1, mRNA [NM_019071]	10,67	11,751	-1,08
MGC27348	Homo sapiens ribosomal protein S2 pseudogene, mRNA (cDNA clone MGC:27348 IMAGE:4671259), complete cds. [BC026177]	13,377	14,458	-1,08
AX721252	Sequence 212 from Patent WO0220754. [AX721252]	11,666	12,748	-1,08
RPS19	Homo sapiens ribosomal protein S19 (RPS19), mRNA [NM_001022]	13,68	14,763	-1,08
SETD8	Homo sapiens SET domain containing (lysine methyltransferase) 8 (SETD8), mRNA [NM_020382]	6,432	7,519	-1,09
SH3GLB1	Homo sapiens SH3-domain GRB2-like endophilin B1 (SH3GLB1), mRNA [NM_016009]	11,163	12,25	-1,09
CREB1	Homo sapiens cAMP responsive element binding protein 1 (CREB1), transcript variant B, mRNA [NM_134442]	5,946	7,035	-1,09
-	chr7:043280410-043280469	12,322	13,411	-1,09
C19orf52	Homo sapiens chromosome 19 open reading frame 52 (C19orf52), mRNA [NM_138358]	9,693	10,785	-1,09
LOC387787	PREDICTED: Homo sapiens similar to CG9804-PA, transcript variant 1 (LOC387787), mRNA [XM_370636]	6,418	7,512	-1,09
DOCK7	Homo sapiens dedicator of cytokinesis 7 (DOCK7), mRNA [NM_033407]	5,487	6,583	-1,10
ZNF207	Homo sapiens zinc finger protein 207 (ZNF207), transcript variant 2, mRNA [NM_001032293]	10,042	11,139	-1,10
DW443340	HHAGE041070 Human liver regeneration after partial hepatectomy Homo sapiens cDNA, mRNA sequence [DW443340]	7,562	8,66	-1,10
SAAL1	Homo sapiens serum amyloid A-like 1 (SAAL1), mRNA [NM_138421]	9,116	10,215	-1,10
SFXN1	Homo sapiens sideroflexin 1 (SFXN1), mRNA [NM_022754]	8,493	9,595	-1,10
N75427	za82f07.s1 Soares_fetal_lung_NbHL19W Homo sapiens cDNA clone IMAGE:299077 3' similar to gb:X69654 40S RIBOSOMAL PROTEIN S26 (HUMAN);, mRNA sequence [N75427]	9,543	10,648	-1,11
C13orf27	Homo sapiens chromosome 13 open reading frame 27 (C13orf27), mRNA [NM_138779]	9,464	10,573	-1,11
C12orf47	Homo sapiens chromosome 12 open reading frame 47, mRNA (cDNA clone IMAGE:4122851). [BC007973]	8,238	9,348	-1,11
FAM96A	Homo sapiens family with sequence similarity 96, member A (FAM96A), transcript variant 1, mRNA [NM_032231]	11,153	12,262	-1,11
-	chr2:065169252-065169311	6,317	7,429	-1,11
CD46	Homo sapiens CD46 molecule, complement regulatory protein (CD46), transcript variant n, mRNA [NM_172350]	6,825	7,937	-1,11
SMG1	Homo sapiens PI-3-kinase-related kinase SMG-1 (SMG1), mRNA [NM_015092]	8,411	9,526	-1,12
CA337741	CA337741 NISC_lw05f11.y1 COGENE 4PA1 Homo sapiens cDNA clone IMAGE:5609612 5', mRNA sequence [CA337741]	5,881	6,998	-1,12
PANK3	Homo sapiens pantothenate kinase 3 (PANK3), mRNA [NM_024594]	7,818	8,936	-1,12
SPATA5L1	Homo sapiens spermatogenesis associated 5-like 1 (SPATA5L1), mRNA [NM_024063]	9,965	11,083	-1,12
DHX15	Homo sapiens DEAH (Asp-Glu-Ala-His) box polypeptide 15 (DHX15), mRNA [NM_001358]	11,222	12,343	-1,12
POLE3	Homo sapiens polymerase (DNA directed), epsilon 3 (p17 subunit) (POLE3), mRNA [NM_017443]	10,874	11,995	-1,12
C16orf33	Homo sapiens chromosome 16 open reading frame 33 (C16orf33), mRNA [NM_024571]	10,642	11,765	-1,12
EFCAB4A	Homo sapiens EF-hand calcium binding domain 4A (EFCAB4A), mRNA [NM_173584]	9,031	10,155	-1,12
PIAS2	Homo sapiens protein inhibitor of activated STAT, 2 (PIAS2), transcript variant alpha, mRNA [NM_173206]	6,797	7,92	-1,12
PPM1A	Homo sapiens protein phosphatase 1A (formerly 2C), magnesium-dependent, alpha isoform (PPM1A), transcript variant 1, mRNA [NM_021003]	11,2	12,324	-1,12

LOC732268	PREDICTED: Homo sapiens similar to Glyceraldehyde-3-phosphate dehydrogenase (GAPDH) (LOC732268), mRNA [XR_015796]	8,295	9,421	-1,13
SCYL2	Homo sapiens SCY1-like 2 (S. cerevisiae) (SCYL2), mRNA [NM_017988]	10,448	11,574	-1,13
ATP5B	Homo sapiens ATP synthase, H+ transporting, mitochondrial F1 complex, beta polypeptide (ATP5B), nuclear gene encoding mitochondrial protein, mRNA [NM_001686]	12,762	13,892	-1,13
ATPAF2	Homo sapiens ATP synthase mitochondrial F1 complex assembly factor 2 (ATPAF2), nuclear gene encoding mitochondrial protein, mRNA [NM_145691]	8,891	10,022	-1,13
-	chr15:042434782-042434841	10,291	11,426	-1,14
BNIP2	Homo sapiens BCL2/adenovirus E1B 19kDa interacting protein 2 (BNIP2), mRNA [NM_004330]	9,241	10,376	-1,14
EIF3S2	Homo sapiens eukaryotic translation initiation factor 3, subunit 2 beta, 36kDa (EIF3S2), mRNA [NM_003757]	11,885	13,02	-1,14
DUSP12	Homo sapiens dual specificity phosphatase 12 (DUSP12), mRNA [NM_007240]	11,811	12,949	-1,14
KIAA1333	Homo sapiens KIAA1333 (KIAA1333), mRNA [NM_017769]	7,442	8,581	-1,14
SGPL1	Homo sapiens sphingosine-1-phosphate lyase 1 (SGPL1), mRNA [NM_003901]	6,159	7,301	-1,14
SDHB	Homo sapiens succinate dehydrogenase complex, subunit B, iron sulfur (Ip) (SDHB), mRNA [NM_003000]	11,048	12,192	-1,15
-	chr18:055642588-055642973	13,28	14,425	-1,15
BRWD3	Homo sapiens bromodomain and WD repeat domain containing 3 (BRWD3), mRNA [NM_153252]	5,07	6,217	-1,15
KIAA1143	Homo sapiens KIAA1143 (KIAA1143), mRNA [NM_020696]	8,756	9,905	-1,15
BQ233242	BQ233242 AGENCOURT_7283259 NIH_MGC_70 Homo sapiens cDNA clone IMAGE:6017276 5', mRNA sequence [BQ233242]	8,917	10,067	-1,15
LOC391566	PREDICTED: Homo sapiens similar to Histone H2B 291B (LOC391566), mRNA [XR_018583]	5,806	6,957	-1,15
AI267321	AI267321 aq63f01.x1 Stanley Frontal SN pool 2 Homo sapiens cDNA clone IMAGE:2035609, mRNA sequence [AI267321]	5,097	6,249	-1,15
RNMT	Homo sapiens RNA (guanine-7-) methyltransferase (RNMT), mRNA [NM_003799]	7,301	8,453	-1,15
WDFY3	Homo sapiens WD repeat and FYVE domain containing 3 (WDFY3), transcript variant 2, mRNA [NM_178583]	8,089	9,242	-1,15
AW875618	AW875618 QV2-PT0012-040400-124-b11 PT0012 Homo sapiens cDNA, mRNA sequence [AW875618]	4,968	6,122	-1,15
C2orf13	Homo sapiens chromosome 2 open reading frame 13 (C2orf13), mRNA [NM_173545]	5,513	6,667	-1,15
EXOSC8	Homo sapiens exosome component 8 (EXOSC8), mRNA [NM_181503]	11,677	12,831	-1,15
UBE2V1	Homo sapiens ubiquitin-conjugating enzyme E2 variant 1 (UBE2V1), transcript variant 4, mRNA [NM_001032288]	9,024	10,178	-1,16
ATP5J2	Homo sapiens ATP synthase, H+ transporting, mitochondrial F0 complex, subunit F2 (ATP5J2), nuclear gene encoding mitochondrial protein, transcript variant 3, mRNA [NM_001003714]	11,83	12,987	-1,16
EIF3S5	Homo sapiens eukaryotic translation initiation factor 3, subunit 5 epsilon, 47kDa (EIF3S5), mRNA [NM_003754]	12,655	13,813	-1,16
YWHAE	Homo sapiens tyrosine 3-monooxygenase/tryptophan 5-monooxygenase activation protein, epsilon polypeptide (YWHAE), mRNA [NM_006761]	8,452	9,61	-1,16
LOC131691	PREDICTED: Homo sapiens similar to peptidylprolyl isomerase A isoform 1 (LOC131691), mRNA [XM_929754]	10,978	12,139	-1,16
APRT	Homo sapiens adenine phosphoribosyltransferase (APRT), transcript variant 1, mRNA [NM_000485]	12,306	13,471	-1,16
NOLA3	Homo sapiens nucleolar protein family A, member 3 (H/ACA small nucleolar RNPs) (NOLA3), mRNA [NM_018648]	12,829	13,993	-1,16
ZNF614	Homo sapiens zinc finger protein 614 (ZNF614), mRNA [NM_025040]	6,689	7,857	-1,17
C1orf58	Homo sapiens chromosome 1 open reading frame 58 (C1orf58), mRNA [NM_144695]	5,232	6,401	-1,17

RMND1	Homo sapiens required for meiotic nuclear division 1 homolog (S. cerevisiae) (RMND1), mRNA [NM_017909]	7,419	8,589	-1,17
SMC5	Homo sapiens structural maintenance of chromosomes 5 (SMC5), mRNA [NM_015110]	7,264	8,435	-1,17
C11orf51	Homo sapiens chromosome 11 open reading frame 51 (C11orf51), mRNA [NM_014042]	8,203	9,375	-1,17
ZCCHC8	Homo sapiens zinc finger, CCHC domain containing 8 (ZCCHC8), mRNA [NM_017612]	10,37	11,542	-1,17
C4orf28	Homo sapiens chromosome 4 open reading frame 28 (C4orf28), mRNA [NM_145048]	7,032	8,205	-1,17
GSR	Homo sapiens glutathione reductase (GSR), mRNA [NM_000637]	8,523	9,697	-1,17
AF118084	Homo sapiens PRO1914 mRNA, complete cds. [AF118084]	5,085	6,262	-1,18
LOC645256	PREDICTED: Homo sapiens similar to Glyceraldehyde-3-phosphate dehydrogenase (GAPDH) (LOC645256), mRNA [XR_016875]	11,659	12,837	-1,18
AA973155	AA973155 on93d01.s1 Soares_NFL_T_GBC_S1 Homo sapiens cDNA clone IMAGE:1564225 3' similar to TR:Q92564 Q92564 MYELOBLAST KIAA0276 ;, mRNA sequence [AA973155]	4,405	5,583	-1,18
SAR1B	Homo sapiens SAR1 gene homolog B (S. cerevisiae) (SAR1B), transcript variant 1, mRNA [NM_001033503]	8,791	9,969	-1,18
RASA2	Homo sapiens RAS p21 protein activator 2 (RASA2), mRNA [NM_006506]	5,799	6,98	-1,18
IER3IP1	Homo sapiens PRO2309 mRNA, complete cds. [AF119875]	7,356	8,538	-1,18
PEX13	Homo sapiens peroxisome biogenesis factor 13 (PEX13), mRNA [NM_002618]	8,723	9,905	-1,18
RPL35	Homo sapiens ribosomal protein L35 (RPL35), mRNA [NM_007209]	13,1	14,285	-1,18
AI698357	tx64a04.x1 NCI_CGAP_Ut1 Homo sapiens cDNA clone IMAGE:2274318 3', mRNA sequence [AI698357]	7,692	8,879	-1,19
C16orf74	Homo sapiens chromosome 16 open reading frame 74, mRNA (cDNA clone MGC:17624 IMAGE:3855543), complete cds. [BC009078]	6,329	7,519	-1,19
TXNDC10	Homo sapiens thioredoxin domain containing 10 (TXNDC10), mRNA [NM_019022]	6,574	7,767	-1,19
AI630435	AI630435 ad10b05.y1 Hembase; Erythroid Progenitor Cells (LCB:ad library) Homo sapiens cDNA clone ad10b05 random, mRNA sequence [AI630435]	10,104	11,298	-1,19
ALDOAP2	Human aldolase pseudogene mRNA, complete cds. [M21191]	9,921	11,115	-1,19
ENST00000306024	U6 snRNA-associated Sm-like protein LSm3. [Source:Uniprot/SWISSPROT;Acc:P62310] [ENST00000306024]	11,135	12,33	-1,19
NAT13	Homo sapiens N-acetyltransferase 13 (NAT13), mRNA [NM_025146]	9,497	10,691	-1,19
ORMDL1	Homo sapiens ORM1-like 1 (S. cerevisiae) (ORMDL1), mRNA [NM_016467]	11,799	12,993	-1,19
CANT1	Homo sapiens calcium activated nucleotidase 1 (CANT1), mRNA [NM_138793]	5,983	7,181	-1,20
CAT	Homo sapiens catalase (CAT), mRNA [NM_001752]	9,915	11,115	-1,20
RLF	Homo sapiens rearranged L-myc fusion (RLF), mRNA [NM_012421]	9,525	10,725	-1,20
RP4-747L4.3	Homo sapiens hypothetical protein MGC12538, mRNA (cDNA clone MGC:12538 IMAGE:3839075), complete cds. [BC007072]	7,525	8,725	-1,20
ILF2	Homo sapiens interleukin enhancer binding factor 2, 45kDa (ILF2), mRNA [NM_004515]	13,427	14,629	-1,20
SMAP1	Homo sapiens stromal membrane-associated protein 1 (SMAP1), transcript variant 1, mRNA [NM_001044305]	8,315	9,519	-1,20
GRPEL2	Homo sapiens GrpE-like 2, mitochondrial (E. coli) (GRPEL2), nuclear gene encoding mitochondrial protein, mRNA [NM_152407]	8,145	9,353	-1,21
EEF1B2	Homo sapiens eukaryotic translation elongation factor 1 beta 2 (EEF1B2), transcript variant 1, mRNA [NM_001959]	12,959	14,171	-1,21
W05707	W05707 za87h03.r1 Soares_fetal_lung_NbHL19W Homo sapiens cDNA clone IMAGE:299573 5' similar to SW:TCPD_MOUSE P80315 T-COMPLEX PROTEIN 1, DELTA SUBUNIT ;, mRNA sequence [W05707]	4,875	6,086	-1,21

Supplemental Table 1
Morandi et al

CYP4F2	Homo sapiens cytochrome P450, family 4, subfamily F, polypeptide 2 (CYP4F2), mRNA [NM_001082]	5,516	6,728	-1,21
-	chr6:160097086-160097027	9,283	10,498	-1,22
HP1BP3	Homo sapiens heterochromatin protein 1, binding protein 3 (HP1BP3), mRNA [NM_016287]	8,077	9,292	-1,22
RPL26L1	Homo sapiens ribosomal protein L26-like 1 (RPL26L1), mRNA [NM_016093]	10,247	11,463	-1,22
ZDHHC20	Homo sapiens zinc finger, DHHC-type containing 20 (ZDHHC20), mRNA [NM_153251]	5,864	7,079	-1,22
TALDO1	Homo sapiens transaldolase 1 (TALDO1), mRNA [NM_006755]	13,173	14,39	-1,22
THC2645975	BF930236 IL5-NT0227-111200-319-g04 NT0227 Homo sapiens cDNA, mRNA sequence [BF930236]	4,856	6,075	-1,22
MPHOSPH9	Homo sapiens M-phase phosphoprotein 9 (MPHOSPH9), mRNA [NM_022782]	7,129	8,347	-1,22
CREBBP	Homo sapiens CREB binding protein (Rubinstein-Taybi syndrome) (CREBBP), transcript variant 1, mRNA [NM_004380]	8,109	9,328	-1,22
MAPK1	Homo sapiens mitogen-activated protein kinase 1 (MAPK1), transcript variant 2, mRNA [NM_138957]	7,854	9,074	-1,22
HSPC159	Homo sapiens galectin-related protein (HSPC159), mRNA [NM_014181]	7,869	9,092	-1,22
SFRS3	Homo sapiens splicing factor, arginine/serine-rich 3 (SFRS3), mRNA [NM_003017]	13,627	14,851	-1,22
LOC441743	Homo sapiens similar to C367G8.3 (novel protein similar to RPL23A (60S ribosomal protein L23A)) (LOC441743), mRNA [NM_001045548]	13,191	14,416	-1,23
SFRS7	Homo sapiens splicing factor, arginine/serine-rich 7, 35kDa (SFRS7), mRNA [NM_001031684]	13,02	14,246	-1,23
ENST00000328644	CSL-type zinc finger-containing protein 1. [Source:Uniprot/SWISSPROT;Acc:Q9H4G8] [ENST00000328644]	7,269	8,495	-1,23
PPIH	Homo sapiens peptidylprolyl isomerase H (cyclophilin H) (PPIH), mRNA [NM_006347]	6,892	8,118	-1,23
MTHFSD	Homo sapiens cDNA FLJ43025 fis, clone BRTHA2018707. [AK125015]	7,386	8,613	-1,23
BUB3	Homo sapiens BUB3 budding uninhibited by benzimidazoles 3 homolog (yeast) (BUB3), transcript variant 2, mRNA [NM_001007793]	9,899	11,126	-1,23
THC2578949	AY918495 mitochondrial cytochrome c somatic {Macaca sylvanus} (exp=-1; wgp=0; cg=0), complete [THC2578949]	11,25	12,478	-1,23
VRK1	Homo sapiens vaccinia related kinase 1 (VRK1), mRNA [NM_003384]	9,193	10,422	-1,23
-	chr6:150341190-150341253	10,702	11,931	-1,23
-	chr5:118337992-118338051	10,45	11,682	-1,23
CEP76	Homo sapiens centrosomal protein 76kDa (CEP76), mRNA [NM_024899]	6,964	8,198	-1,23
BC007306	Homo sapiens 5-aminoimidazole-4-carboxamide ribonucleotide formyltransferase/IMP cyclohydrolase, mRNA (cDNA clone IMAGE:3352215). [BC007306]	5,651	6,886	-1,24
MGC5370	Homo sapiens hypothetical protein MGC5370, mRNA (cDNA clone IMAGE:3049213), partial cds. [BC006795]	7,792	9,028	-1,24
-	chr4:079098880-079098821	7,35	8,588	-1,24
LOC388272	Homo sapiens similar to RIKEN cDNA 4921524J17 (LOC388272), mRNA [NM_001001436]	9,84	11,078	-1,24
RBM25	Homo sapiens RNA binding motif protein 25 (RBM25), mRNA [NM_021239]	10,32	11,559	-1,24
CDC42SE2	Homo sapiens CDC42 small effector 2 (CDC42SE2), transcript variant 1, mRNA [NM_020240]	7,399	8,641	-1,24
C16orf61	Homo sapiens chromosome 16 open reading frame 61 (C16orf61), mRNA [NM_020188]	11,425	12,669	-1,24
SUV39H2	Homo sapiens suppressor of variegation 3-9 homolog 2 (Drosophila) (SUV39H2), mRNA [NM_024670]	8,589	9,833	-1,24
-	chr14:076422785-076422717	12,785	14,03	-1,25
THC2672768	chr3:31547505-31547446	5,876	7,121	-1,25
ZNF440	Homo sapiens cDNA FLJ35240 fis, clone PROST2002425, moderately similar to ZINC FINGER PROTEIN 136. [AK092559]	6,187	7,432	-1,25

Supplemental Table 1
Morandi et al

SMARCD1	Homo sapiens SWI/SNF related, matrix associated, actin dependent regulator of chromatin, subfamily d, member 1 (SMARCD1), transcript variant 2, mRNA [NM_139071]	5,359	6,605	-1,25
C1orf144	Homo sapiens chromosome 1 open reading frame 144 (C1orf144), mRNA [NM_015609]	10,364	11,614	-1,25
-	chr14:103216468-103216409	8,694	9,944	-1,25
TMEM33	Homo sapiens transmembrane protein 33 (TMEM33), mRNA [NM_018126]	8,695	9,945	-1,25
C18orf17	Homo sapiens chromosome 18 open reading frame 17, mRNA (cDNA clone IMAGE:5582870), partial cds. [BC032684]	8,268	9,52	-1,25
SFRS10	Homo sapiens splicing factor, arginine/serine-rich 10 (transformer 2 homolog, Drosophila) (SFRS10), mRNA [NM_004593]	12,169	13,421	-1,25
COPS3	Homo sapiens COP9 constitutive photomorphogenic homolog subunit 3 (Arabidopsis) (COPS3), mRNA [NM_003653]	12,142	13,397	-1,26
H2AFZ	Homo sapiens H2A histone family, member Z (H2AFZ), mRNA [NM_002106]	13,006	14,261	-1,26
NAPG	Homo sapiens N-ethylmaleimide-sensitive factor attachment protein, gamma (NAPG), mRNA [NM_003826]	7,562	8,82	-1,26
CDCA7L	Homo sapiens cell division cycle associated 7-like (CDCA7L), mRNA [NM_018719]	8,48	9,738	-1,26
LOC137886	Homo sapiens hypothetical protein LOC137886 (LOC137886), mRNA [NM_001077619]	6,368	7,626	-1,26
CSNK1G3	Homo sapiens casein kinase 1, gamma 3 (CSNK1G3), transcript variant 4, mRNA [NM_001044723]	7,118	8,378	-1,26
-	chr6:118427073-118427132	13,213	14,474	-1,26
DLEU1	Homo sapiens deleted in lymphocytic leukemia, 1 (DLEU1) on chromosome 13 [NR_002605]	7,173	8,435	-1,26
FLJ37953	Homo sapiens hypothetical protein FLJ37953 (FLJ37953), mRNA [NM_001039693]	5,724	6,986	-1,26
HSPA4	Homo sapiens heat shock 70kDa protein 4 (HSPA4), transcript variant 1, mRNA [NM_002154]	12,515	13,777	-1,26
CBLL1	Homo sapiens Cas-Br-M (murine) ecotropic retroviral transforming sequence-like 1 (CBLL1), mRNA [NM_024814]	6,876	8,141	-1,26
U63542	Human putative FAP protein mRNA, partial cds. [U63542]	4,872	6,137	-1,26
SLC25A40	Homo sapiens solute carrier family 25, member 40 (SLC25A40), mRNA [NM_018843]	6,573	7,838	-1,27
ING1	Homo sapiens inhibitor of growth family, member 1 (ING1), transcript variant 1, mRNA [NM_198219]	8,929	10,195	-1,27
LOC391738	PREDICTED: Homo sapiens similar to 60S ribosomal protein L29 (Cell surface heparin-binding protein HIP) (LOC391738), mRNA [XR_019052]	9,798	11,064	-1,27
THC2782843	RL18A_HUMAN (Q02543) 60S ribosomal protein L18a, partial (89%) [THC2782843]	11,837	13,103	-1,27
-	chr7:006371630-006371676	10,715	11,982	-1,27
THC2651723	HUMUBCP pro-ubiquitin {Homo sapiens} (exp=-1; wgp=0; cg=0), partial (39%) [THC2651723]	11,953	13,22	-1,27
PX19	Homo sapiens px19-like protein (PX19), mRNA [NM_013237]	11,823	13,091	-1,27
AK124072	Homo sapiens cDNA FLJ42078 fis, clone SYNOV2020085. [AK124072]	7,65	8,919	-1,27
AK097724	Homo sapiens cDNA FLJ40405 fis, clone TESTI2037382. [AK097724]	6,277	7,547	-1,27
APOM	Homo sapiens apolipoprotein M (APOM), mRNA [NM_019101]	5,952	7,224	-1,27
SP1	Homo sapiens Sp1 transcription factor (SP1), mRNA [NM_138473]	5,408	6,68	-1,27
BX360933	BX360933 BX360933 Homo sapiens PLACENTA COT 25-NORMALIZED Homo sapiens cDNA clone CS0DI077YB17 3-PRIME, mRNA sequence [BX360933]	6,364	7,638	-1,27
UBE2D3	Homo sapiens ubiquitin-conjugating enzyme E2D 3 (UBC4/5 homolog, yeast) (UBE2D3), transcript variant 2, mRNA [NM_181886]	9,935	11,211	-1,28
BC039097	Homo sapiens cDNA clone IMAGE:4823416. [BC039097]	7,682	8,961	-1,28
AK094969	Homo sapiens cDNA FLJ37650 fis, clone BRHIP2000553, weakly similar to UBIQUITIN-ACTIVATING ENZYME E1 1. [AK094969]	6,609	7,888	-1,28

Supplemental Table 1
Morandi et al

CCDC59	Homo sapiens coiled-coil domain containing 59 (CCDC59), mRNA [NM_014167]	11,049	12,33	-1,28
IFRD1	Homo sapiens interferon-related developmental regulator 1 (IFRD1), transcript variant 2, mRNA [NM_001007245]	10,864	12,146	-1,28
GPSM2	Homo sapiens G-protein signalling modulator 2 (AGS3-like, C. elegans) (GPSM2), mRNA [NM_013296]	8,193	9,476	-1,28
BF513730	BF513730 UI-H-BW1-amy-e-05-0-UI.s1 NCI_CGAP_Sub7 Homo sapiens cDNA clone IMAGE:3071696 3', mRNA sequence [BF513730]	6,199	7,483	-1,28
CNIH4	Homo sapiens cornichon homolog 4 (Drosophila) (CNIH4), mRNA [NM_014184]	11,386	12,671	-1,29
UFM1	Homo sapiens ubiquitin-fold modifier 1 (UFM1), mRNA [NM_016617]	7,883	9,167	-1,29
RHOF	Homo sapiens ras homolog gene family, member F (in filopodia) (RHOF), mRNA [NM_019034]	5,279	6,565	-1,29
RNF170	Homo sapiens ring finger protein 170, mRNA (cDNA clone MGC:40251 IMAGE:5200847), complete cds. [BC032393]	5,221	6,507	-1,29
DTYMK	Homo sapiens deoxythymidylate kinase (thymidylate kinase) (DTYMK), mRNA [NM_012145]	9,862	11,15	-1,29
YTHDF3	Homo sapiens YTH domain family, member 3 (YTHDF3), mRNA [NM_152758]	6,376	7,664	-1,29
PDK3	Homo sapiens pyruvate dehydrogenase kinase, isozyme 3 (PDK3), mRNA [NM_005391]	5,577	6,867	-1,29
UBE2D2	Homo sapiens ubiquitin-conjugating enzyme E2D 2 (UBC4/5 homolog, yeast) (UBE2D2), transcript variant 2, mRNA [NM_181838]	9,927	11,217	-1,29
ANKRD2	Homo sapiens ankyrin repeat domain 2 (stretch responsive muscle) (ANKRD2), mRNA [NM_020349]	8,103	9,395	-1,29
AA779434	AA779434 af22b10.s1 Soares_total_fetus_Nb2HF8_9w Homo sapiens cDNA clone IMAGE:1032379 3', mRNA sequence [AA779434]	7,939	9,232	-1,29
THC2756939	Q206M1_9ARAC (Q206M1) Major ampullate spidroin 2 (Fragment), partial (3%) [THC2756939]	8,475	9,769	-1,29
PSEN1	Homo sapiens PSN1 gene, alternative transcript. [AJ008005]	6,084	7,378	-1,29
ASB7	Homo sapiens ankyrin repeat and SOCS box-containing 7 (ASB7), transcript variant 1, mRNA [NM_024708]	6,057	7,352	-1,30
AK054718	Homo sapiens cDNA FLJ30156 fis, clone BRACE2000487. [AK054718]	6,94	8,236	-1,30
LIN7C	Homo sapiens lin-7 homolog C (C. elegans) (LIN7C), mRNA [NM_018362]	7,301	8,597	-1,30
BE165955	BE165955 MR3-HT0487-140200-112-g02 HT0487 Homo sapiens cDNA, mRNA sequence [BE165955]	6,201	7,499	-1,30
C10orf137	Homo sapiens chromosome 10 open reading frame 137 (C10orf137), mRNA [NM_015608]	4,856	6,16	-1,30
WEE1	Homo sapiens WEE1 homolog (S. pombe) (WEE1), mRNA [NM_003390]	7,276	8,58	-1,30
HNRPA3	Homo sapiens heterogeneous nuclear ribonucleoprotein A3 (HNRPA3), mRNA [NM_194247]	12,924	14,229	-1,31
XRN1	Homo sapiens 5'-3' exoribonuclease 1 (XRN1), transcript variant 1, mRNA [NM_019001]	4,752	6,058	-1,31
RP3-377H14.5	Homo sapiens cDNA FLJ35429 fis, clone SMINT2002126. [AK092748]	12,472	13,779	-1,31
USF2	Homo sapiens clone TCCCIA00046 mRNA sequence. [AY007087]	4,615	5,921	-1,31
LOC644651	PREDICTED: Homo sapiens similar to High mobility group protein B1 (High mobility group protein 1) (HMG-1) (Amphoterin) (Heparin-binding protein p30) (LOC644651), mRNA [XR_016752]	10,511	11,819	-1,31
UXT	Homo sapiens ubiquitously-expressed transcript (UXT), transcript variant 2, mRNA [NM_004182]	11,873	13,182	-1,31
-	chr4:039651569-039651508	6,43	7,739	-1,31
FAM76B	Homo sapiens family with sequence similarity 76, member B (FAM76B), mRNA [NM_144664]	7,626	8,936	-1,31
AW298233	AW298233 UI-H-BW0-ajt-g-07-0-UI.s1 NCI_CGAP_Sub6 Homo sapiens cDNA clone IMAGE:2733109 3', mRNA sequence [AW298233]	5,375	6,688	-1,31

Supplemental Table 1
Morandi et al

EIF5A	Homo sapiens eukaryotic translation initiation factor 5A (EIF5A), mRNA [NM_001970]	8,529	9,844	-1,32
RAF1	Homo sapiens v-raf-1 murine leukemia viral oncogene homolog 1 (RAF1), mRNA [NM_002880]	10,124	11,442	-1,32
TMEM70	Homo sapiens transmembrane protein 70 (TMEM70), transcript variant 2, mRNA [NM_001040613]	10,004	11,322	-1,32
ALS2CR4	Homo sapiens amyotrophic lateral sclerosis 2 (juvenile) chromosome region, candidate 4 (ALS2CR4), transcript variant 1, mRNA [NM_001044385]	10,066	11,385	-1,32
ENST00000383513	PREDICTED: Homo sapiens similar to peptidylprolyl isomerase A isoform 1 (LOC128192), mRNA [XM_060887]	9,754	11,073	-1,32
DNAH11	Homo sapiens dynein, axonemal, heavy chain 11 (DNAH11), mRNA [NM_003777]	4,77	6,091	-1,32
2'-PDE	Homo sapiens 2'-phosphodiesterase (2'-PDE), mRNA [NM_177966]	4,874	6,197	-1,32
LOC203547	Homo sapiens hypothetical protein LOC203547 (LOC203547), mRNA [NM_001017980]	7,998	9,321	-1,32
C16orf69	Homo sapiens chromosome 16 open reading frame 69 (C16orf69), mRNA [NM_153261]	5,825	7,15	-1,33
-	chr1:015992198-015992263	9,487	10,816	-1,33
C2orf25	Homo sapiens chromosome 2 open reading frame 25 (C2orf25), mRNA [NM_015702]	11,151	12,48	-1,33
BC013025	Homo sapiens cDNA clone IMAGE:3528660, **** WARNING: chimeric clone ****. [BC013025]	6,836	8,167	-1,33
ENST00000356170	FKBP1A protein. [Source:Uniprot/SPTREMBL;Acc:Q0VDC5] [ENST00000356170]	8,361	9,691	-1,33
MAPKAPK3	Homo sapiens mitogen-activated protein kinase-activated protein kinase 3 (MAPKAPK3), mRNA [NM_004635]	8,991	10,322	-1,33
LOC643981	PREDICTED: Homo sapiens similar to 40S ribosomal protein S3a (V-fos transformation effector protein) (LOC643981), mRNA [XR_018444]	9,835	11,166	-1,33
IMPACT	Homo sapiens Impact homolog (mouse) (IMPACT), mRNA [NM_018439]	8,605	9,94	-1,34
RPL34	Homo sapiens ribosomal protein L34 (RPL34), transcript variant 2, mRNA [NM_033625]	12,059	13,395	-1,34
AW858928	AW858928 RC3-CT0347-210400-016-c07 CT0347 Homo sapiens cDNA, mRNA sequence [AW858928]	5,442	6,779	-1,34
THC2492936	BG978199 RC4-CI0194-060201-015-g03 CI0194 Homo sapiens cDNA, mRNA sequence [BG978199]	8,436	9,774	-1,34
H2AFV	Homo sapiens H2A histone family, member V (H2AFV), transcript variant 1, mRNA [NM_012412]	13,159	14,499	-1,34
RMND5A	Homo sapiens required for meiotic nuclear division 5 homolog A (S. cerevisiae) (RMND5A), mRNA [NM_022780]	6,108	7,448	-1,34
LOC256374	PREDICTED: Homo sapiens similar to peptidylprolyl isomerase A isoform 1 (LOC256374), mRNA [XM_001131005]	11,099	12,44	-1,34
tcag7.441	PREDICTED: Homo sapiens similar to Rieske iron-sulfur protein (LOC647123), mRNA [XM_930141]	8,186	9,528	-1,34
THC2591750	AF238866 LNR42 {Mus musculus} (exp=-1; wgp=0; cg=0), partial (34%) [THC2591750]	5,38	6,722	-1,34
LOC440180	PREDICTED: Homo sapiens similar to zinc finger, CCHC domain containing 7 (LOC440180), mRNA [XR_018312]	5,156	6,499	-1,34
MAPK14	Homo sapiens mitogen-activated protein kinase 14 (MAPK14), transcript variant 3, mRNA [NM_139013]	5,038	6,382	-1,34
GTF3C3	Homo sapiens transcription factor IIIC102 short isoform mRNA, complete cds. [AF465407]	5,781	7,125	-1,34
SAP30	Homo sapiens Sin3A-associated protein, 30kDa (SAP30), mRNA [NM_003864]	11,005	12,349	-1,34
BC039021	Homo sapiens cDNA clone IMAGE:6043059, partial cds. [BC039021]	7,856	9,203	-1,35
CKAP2	Homo sapiens cytoskeleton associated protein 2 (CKAP2), mRNA [NM_018204]	9,769	11,119	-1,35
SLC25A5	Homo sapiens solute carrier family 25 (mitochondrial carrier; adenine nucleotide translocator), member 5 (SLC25A5), mRNA [NM_001152]	12,837	14,187	-1,35
THC2556772	chr14:076422777-076422719	12,137	13,488	-1,35

Supplemental Table 1
Morandi et al

C4orf15	Homo sapiens chromosome 4 open reading frame 15 (C4orf15), mRNA [NM_024511]	7,462	8,814	-1,35
LRIG2	Homo sapiens leucine-rich repeats and immunoglobulin-like domains 2 (LRIG2), mRNA [NM_014813]	7,529	8,882	-1,35
-	chr15:041347566-041347505	9,331	10,684	-1,35
C14orf138	Homo sapiens chromosome 14 open reading frame 138 (C14orf138), transcript variant 1, mRNA [NM_024558]	8,475	9,828	-1,35
CLEC2D	Homo sapiens C-type lectin domain family 2, member D (CLEC2D), transcript variant 2, mRNA [NM_001004419]	11,635	12,992	-1,36
THC2532862	chr3:10309228-10309287	6,097	7,453	-1,36
AL136830	Homo sapiens mRNA; cDNA DKFZp434D1428 (from clone DKFZp434D1428). [AL136830]	5,359	6,716	-1,36
SFRS6	Homo sapiens splicing factor, arginine/serine-rich 6 (SFRS6), mRNA [NM_006275]	8,537	9,895	-1,36
C1orf96	Homo sapiens chromosome 1 open reading frame 96, mRNA (cDNA clone MGC:32857 IMAGE:4731004), complete cds. [BC039241]	5,301	6,66	-1,36
PIAS2	Homo sapiens protein inhibitor of activated STAT, 2 (PIAS2), transcript variant beta, mRNA [NM_004671]	7,689	9,049	-1,36
TAF10	Homo sapiens TAF10 RNA polymerase II, TATA box binding protein (TBP)-associated factor, 30kDa (TAF10), mRNA [NM_006284]	10,495	11,855	-1,36
MAD2L2	Homo sapiens MAD2 mitotic arrest deficient-like 2 (yeast) (MAD2L2), mRNA [NM_006341]	10,126	11,488	-1,36
TACC3	Homo sapiens transforming, acidic coiled-coil containing protein 3 (TACC3), mRNA [NM_006342]	8,48	9,842	-1,36
CAMTA1	Homo sapiens hypothetical protein SB141 mRNA, complete cds. [AY037153]	11,753	13,115	-1,36
CR603215	full-length cDNA clone CS0DI023YC17 of Placenta Cot 25-normalized of Homo sapiens (human). [CR603215]	10,208	11,572	-1,36
LYPLA1	Homo sapiens lysophospholipase I (LYPLA1), mRNA [NM_006330]	8,964	10,328	-1,36
HACL1	Homo sapiens 2-hydroxyacyl-CoA lyase 1 (HACL1), mRNA [NM_012260]	10,047	11,411	-1,36
LOC440983	Homo sapiens hypothetical gene supported by BC066916, mRNA (cDNA clone IMAGE:4838452). [BC066916]	10,839	12,203	-1,36
STK10	Homo sapiens serine/threonine kinase 10 (STK10), mRNA [NM_005990]	9,354	10,718	-1,36
AI885257	AI885257 wl91f02.x1 NCI_CGAP_Brn25 Homo sapiens cDNA clone IMAGE:2432283 3', mRNA sequence [AI885257]	4,832	6,2	-1,37
RHOG	Homo sapiens ras homolog gene family, member G (rho G) (RHOG), mRNA [NM_001665]	9,61	10,978	-1,37
TKT	Homo sapiens transketolase (Wernicke-Korsakoff syndrome) (TKT), mRNA [NM_001064]	13,456	14,824	-1,37
HIST1H2AG	Homo sapiens histone cluster 1, H2ag (HIST1H2AG), mRNA [NM_021064]	10,99	12,359	-1,37
LSM11	Homo sapiens LSM11, U7 small nuclear RNA associated (LSM11), mRNA [NM_173491]	6,563	7,931	-1,37
C20orf24	Homo sapiens chromosome 20 open reading frame 24 (C20orf24), transcript variant 1, mRNA [NM_018840]	11,607	12,978	-1,37
NFYA	Homo sapiens nuclear transcription factor Y, alpha (NFYA), transcript variant 1, mRNA [NM_002505]	5,668	7,041	-1,37
RXRA	Homo sapiens retinoid X receptor, alpha (RXRA), mRNA [NM_002957]	6,69	8,062	-1,37
-	chr5:065320262-065320218	5,491	6,876	-1,39
ENST00000306716	PREDICTED: Homo sapiens similar to peptidylprolyl isomerase A isoform 1 (LOC643997), mRNA [XM_292963]	10,805	12,19	-1,39
LOC51136	Homo sapiens PTD016 protein (LOC51136), mRNA [NM_016125]	6,778	8,163	-1,39
WDR51A	Homo sapiens WD repeat domain 51A (WDR51A), mRNA [NM_015426]	6,496	7,881	-1,39
AI936672	wp68d04.x1 NCI_CGAP_Brn25 Homo sapiens cDNA clone IMAGE:2466919 3' similar to TR:Q15559 Q15559 ;, mRNA sequence [AI936672]	9,746	11,134	-1,39
AV698092	AV698092 GKC Homo sapiens cDNA clone GKCGUA09 5', mRNA sequence [AV698092]	9,025	10,413	-1,39

Supplemental Table 1
Morandi et al

AY129015	Homo sapiens clone FP17889 unknown mRNA. [AY129015]	5,448	6,837	-1,39
FUSIP1	Homo sapiens FUS interacting protein (serine/arginine-rich) 1 (FUSIP1), transcript variant 1, mRNA [NM_006625]	8,323	9,714	-1,39
LIN7A	Homo sapiens lin-7 homolog A (C. elegans) (LIN7A), mRNA [NM_004664]	6,432	7,824	-1,39
NCAPD2	Homo sapiens non-SMC condensin I complex, subunit D2 (NCAPD2), mRNA [NM_014865]	10,008	11,4	-1,39
AA065042	AA065042 zm12g12.s1 Stratagene pancreas (#937208) Homo sapiens cDNA clone IMAGE:525478 3', mRNA sequence [AA065042]	5,456	6,848	-1,39
ENST0000036150(	chr12:074644880-074644939	8,512	9,905	-1,39
LOC732199	PREDICTED: Homo sapiens similar to 60S ribosomal protein L29 (Cell surface heparin-binding protein HIP) (LOC732199), mRNA [XR_015762]	11,096	12,49	-1,39
-	chr17:040946223-040946159	5,268	6,662	-1,40
CHAF1B	Homo sapiens chromatin assembly factor 1, subunit B (p60) (CHAF1B), mRNA [NM_005441]	9,262	10,657	-1,40
LOC647094	PREDICTED: Homo sapiens similar to acidic ribosomal phosphoprotein P0 (LOC647094), mRNA [XR_017377]	11,894	13,289	-1,40
NDUFA4	Homo sapiens NADH dehydrogenase (ubiquinone) 1 alpha subcomplex, 4, 9kDa (NDUFA4), nuclear gene encoding mitochondrial protein, mRNA [NM_002489]	13,276	14,675	-1,40
FLJ10213	Homo sapiens hypothetical protein FLJ10213 (FLJ10213), mRNA [NM_018029]	9,711	11,112	-1,40
TMEM170	Homo sapiens transmembrane protein 170 (TMEM170), mRNA [NM_145254]	9,19	10,59	-1,40
LOC392497	PREDICTED: Homo sapiens similar to 40S ribosomal protein S6 (LOC392497), mRNA [XR_018138]	12,858	14,261	-1,40
RHOA	Homo sapiens ras homolog gene family, member A (RHOA), mRNA [NM_001664]	12,06	13,464	-1,40
AA743218	AA743218 ny21c02.s1 NCI_CGAP_GCB1 Homo sapiens cDNA clone IMAGE:1272386 3' similar to gb:S87759 PROTEIN PHOSPHATASE 2C ALPHA ISOFORM (HUMAN);, mRNA sequence [AA743218]	7,209	8,614	-1,41
C14orf32	Homo sapiens chromosome 14 open reading frame 32 (C14orf32), mRNA [NM_144578]	8,204	9,612	-1,41
RPL29	Homo sapiens ribosomal protein L29 (RPL29), mRNA [NM_000992]	13,435	14,843	-1,41
BC040702	Homo sapiens, clone IMAGE:5736845, mRNA. [BC040702]	8,546	9,955	-1,41
ZMYM5	Homo sapiens zinc finger, MYM-type 5 (ZMYM5), transcript variant 1, mRNA [NM_001039650]	7,214	8,623	-1,41
BC000604	Homo sapiens mRNA similar to cisplatin resistance-associated overexpressed protein (cDNA clone IMAGE:3346964). [BC000604]	5,554	6,965	-1,41
LOC654170	PREDICTED: Homo sapiens similar to 40S ribosomal protein S16 (LOC654170), mRNA [XR_019059]	13,091	14,502	-1,41
MALT1	Homo sapiens mucosa associated lymphoid tissue lymphoma translocation gene 1 (MALT1), transcript variant 1, mRNA [NM_006785]	7,329	8,742	-1,41
LOC441893	PREDICTED: Homo sapiens similar to Glyceraldehyde-3-phosphate dehydrogenase (GAPDH) (LOC441893), mRNA [XR_018352]	7,981	9,394	-1,41
USP6NL	Homo sapiens USP6 N-terminal like, mRNA (cDNA clone IMAGE:4047207), partial cds. [BC010351]	4,905	6,319	-1,41
CA5B	Homo sapiens carbonic anhydrase VB, mitochondrial (CA5B), nuclear gene encoding mitochondrial protein, mRNA [NM_007220]	5,66	7,075	-1,42
ENST0000026929(	Homo sapiens HSPC254 mRNA, partial cds. [AF161372]	7,211	8,626	-1,42
-	chr22:023413926-023413985	4,41	5,828	-1,42
FAM44A	Homo sapiens family with sequence similarity 44, member A, mRNA (cDNA clone IMAGE:5503684), with apparent retained intron. [BC043603]	7,483	8,901	-1,42

THC2588312	AI292051 qm77f10.x1 Soares_placenta_8to9weeks_2NbHP8to9W Homo sapiens cDNA clone IMAGE:1894795 3' similar to gb:X69654 40S RIBOSOMAL PROTEIN S26 (HUMAN);, mRNA sequence [AI292051]	4,446	5,864	-1,42
SEPT11	Homo sapiens septin 11 (), mRNA [NM_018243]	8,702	10,128	-1,43
BC107765	Homo sapiens cDNA clone IMAGE:4762811. [BC107765]	5,873	7,302	-1,43
HELLS	Homo sapiens helicase, lymphoid-specific (HELLS), mRNA [NM_018063]	6,607	8,036	-1,43
ENST00000331979	PREDICTED: Homo sapiens similar to 60S ribosomal protein L29 (Cell surface heparin-binding protein HIP) (LOC730837), mRNA [XR_015551]	11,083	12,513	-1,43
LOC646980	PREDICTED: Homo sapiens similar to 60S ribosomal protein L7a (LOC646980), mRNA [XR_018451]	10,473	11,903	-1,43
PURB	Homo sapiens purine-rich element binding protein B (PURB), mRNA [NM_033224]	8,273	9,707	-1,43
UBE2J1	Homo sapiens ubiquitin-conjugating enzyme E2, J1 (UBC6 homolog, yeast) (UBE2J1), mRNA [NM_016021]	7,773	9,207	-1,43
PAFAH1B1	Homo sapiens platelet-activating factor acetylhydrolase, isoform Ib, alpha subunit 45kDa (PAFAH1B1), mRNA [NM_000430]	10,215	11,65	-1,44
THC2685373	chr14:066922345-066922286	7,228	8,663	-1,44
-	chr15:047875305-047875366	5,911	7,35	-1,44
MCL1	Homo sapiens myeloid cell leukemia sequence 1 (BCL2-related) (MCL1), transcript variant 1, mRNA [NM_021960]	7,168	8,608	-1,44
SIAH1	Homo sapiens seven in absentia homolog 1 (Drosophila) (SIAH1), transcript variant 1, mRNA [NM_003031]	9,08	10,519	-1,44
THC2537179	NC_006324 100K {Bovine adenovirus A} (exp=-1; wgp=0; cg=1), partial (4%) [THC2537179]	5,318	6,758	-1,44
AW079854	AW079854 xe54e05.x1 NCI_CGAP_Ut3 Homo sapiens cDNA clone IMAGE:2611712 3', mRNA sequence [AW079854]	8,464	9,905	-1,44
PHTF2	Homo sapiens putative homeodomain transcription factor 2 (PHTF2), mRNA [NM_020432]	6,129	7,571	-1,44
-	chr3:025766034-025766097	12,39	13,832	-1,44
MIER1	Homo sapiens mesoderm induction early response 1 homolog (Xenopus laevis) (MIER1), transcript variant 4, mRNA [NM_001077702]	8,829	10,271	-1,44
THC2769342	chr5:122978357-122978298	4,999	6,444	-1,45
ZNF313	Homo sapiens zinc finger protein 313 (ZNF313), mRNA [NM_018683]	9,855	11,3	-1,45
RPL23A	Homo sapiens ribosomal protein L23a (RPL23A), mRNA [NM_000984]	10,478	11,923	-1,45
KCNN4	Homo sapiens potassium intermediate/small conductance calcium-activated channel, subfamily N, member 4 (KCNN4), mRNA [NM_002250]	8,209	9,658	-1,45
RPS2	Homo sapiens OK/KNS-cl.7 mRNA for ribosomal protein S2, complete cds. [AB065089]	8,916	10,365	-1,45
PPIA	Homo sapiens peptidylprolyl isomerase A (cyclophilin A) (PPIA), mRNA [NM_021130]	13,42	14,872	-1,45
MYCBP	Homo sapiens c-myc binding protein (MYCBP), mRNA [NM_012333]	8,285	9,738	-1,45
BF879636	BF879636 RC2-ET0188-271100-011-c09 ET0188 Homo sapiens cDNA, mRNA sequence [BF879636]	5,19	6,644	-1,45
BE826587	BE826587 QV1-EN0042-300500-224-e04 EN0042 Homo sapiens cDNA, mRNA sequence [BE826587]	5,682	7,137	-1,46
DERL2	Homo sapiens Der1-like domain family, member 2 (DERL2), mRNA [NM_016041]	10,078	11,533	-1,46
-	chr1:233561752-233561693	5,985	7,44	-1,46
-	chr2:108677677-108677736	8,475	9,931	-1,46
HIST1H2BK	Homo sapiens histone cluster 1, H2bk (HIST1H2BK), mRNA [NM_080593]	9,393	10,85	-1,46
MAP2K3	Homo sapiens mitogen-activated protein kinase kinase 3 (MAP2K3), transcript variant B, mRNA [NM_145109]	8,902	10,36	-1,46
AY170823	Homo sapiens transformation-related protein 2 mRNA, complete cds. [AY170823]	10,114	11,572	-1,46

Supplemental Table 1
Morandi et al

ERO1LB	Homo sapiens ERO1-like beta (S. cerevisiae) (ERO1LB), mRNA [NM_019891]	5,144	6,603	-1,46
C15orf29	Homo sapiens chromosome 15 open reading frame 29 (C15orf29), mRNA [NM_024713]	8,825	10,285	-1,46
AL555100	AL555100 AL555100 Homo sapiens HELA CELLS COT 25-NORMALIZED Homo sapiens cDNA clone CS0DK007YP02 3-PRIME, mRNA sequence [AL555100]	8,623	10,085	-1,46
BTG1	Homo sapiens B-cell translocation gene 1, anti-proliferative (BTG1), mRNA [NM_001731]	11,622	13,084	-1,46
TRDMT1	Homo sapiens tRNA aspartic acid methyltransferase 1 (TRDMT1), transcript variant a, mRNA [NM_004412]	5,591	7,054	-1,46
VTI1A	Homo sapiens vesicle transport through interaction with t-SNAREs homolog 1A (yeast) (VTI1A), transcript variant 2, mRNA [NM_145206]	7,357	8,82	-1,46
BE646426	BE646426 7e86f01.x1 NCI_CGAP_CLL1 Homo sapiens cDNA clone IMAGE:3292057 3', mRNA sequence [BE646426]	9,768	11,231	-1,46
THC2683231	Q77CB1_9ALPH (Q77CB1) UL6 protein, partial (3%) [THC2683231]	6,325	7,789	-1,46
AA805504	AA805504 oc12e06.s1 NCI_CGAP_GCB1 Homo sapiens cDNA clone IMAGE:1340674 3' similar to contains Alu repetitive element;, mRNA sequence [AA805504]	5,143	6,608	-1,47
ACSL1	Homo sapiens acyl-CoA synthetase long-chain family member 1 (ACSL1), mRNA [NM_001995]	10,094	11,56	-1,47
ELOVL5	Homo sapiens ELOVL family member 5, elongation of long chain fatty acids (FEN1/Elo2, SUR4/Elo3-like, yeast) (ELOVL5), mRNA [NM_021814]	5,07	6,537	-1,47
RBM3	Homo sapiens RNA binding motif (RNP1, RRM) protein 3 (RBM3), transcript variant 2, mRNA [NM_001017430]	11,214	12,682	-1,47
CMTM7	Homo sapiens CKLF-like MARVEL transmembrane domain containing 7 (CMTM7), transcript variant 1, mRNA [NM_138410]	10,241	11,709	-1,47
RSHL2	Homo sapiens radial spokehead-like 2 (RSHL2), mRNA [NM_031924]	7,825	9,294	-1,47
THC2561267	O00193_HUMAN (O00193) Small acidic protein (SMAP protein) (C11orf58 protein), partial (11%) [THC2561267]	5,06	6,53	-1,47
DDX3X	Homo sapiens DEAD (Asp-Glu-Ala-Asp) box polypeptide 3, X-linked (DDX3X), mRNA [NM_001356]	6,795	8,266	-1,47
CCNT2	Homo sapiens cyclin T2 (CCNT2), transcript variant b, mRNA [NM_058241]	5,769	7,242	-1,47
LOC644422	PREDICTED: Homo sapiens similar to arginine [XR_019449]	7,527	9	-1,47
C14orf106	Homo sapiens chromosome 14 open reading frame 106 (C14orf106), mRNA [NM_018353]	7,002	8,475	-1,47
BF366211	BF366211 IL2-NT0099-300500-096-H08 NT0099 Homo sapiens cDNA, mRNA sequence [BF366211]	6,16	7,634	-1,48
LRRC57	Homo sapiens leucine rich repeat containing 57 (LRRC57), mRNA [NM_153260]	7,619	9,098	-1,48
NAT12	Homo sapiens N-acetyltransferase 12 (NAT12), mRNA [NM_001011713]	6,24	7,72	-1,48
SEPX1	Homo sapiens selenoprotein X, 1 (SEPX1), mRNA [NM_016332]	10,383	11,862	-1,48
TRIM59	Homo sapiens tripartite motif-containing 59 (TRIM59), mRNA [NM_173084]	7,94	9,419	-1,48
CKLF	Homo sapiens chemokine-like factor (CKLF), transcript variant 4, mRNA [NM_181641]	9,361	10,841	-1,48
DPH3	Homo sapiens DPH3, KTI11 homolog (S. cerevisiae) (DPH3), transcript variant 1, mRNA [NM_206831]	8,756	10,237	-1,48
AF116620	Homo sapiens PRO1068 mRNA, complete cds. [AF116620]	4,899	6,382	-1,48
RPL23AP7	Homo sapiens ribosomal protein L23a pseudogene 7 (RPL23AP7) on chromosome 2 [NR_000029]	13,509	14,992	-1,48
CAPN1	Homo sapiens cDNA FLJ12257 fis, clone MAMMA1001501, highly similar to CALPAIN 1, LARGE [CATALYTIC] SUBUNIT (EC 3.4.22.17). [AK022319]	8,015	9,5	-1,49
CR612178	full-length cDNA clone CS0DI015YM13 of Placenta Cot 25-normalized of Homo sapiens (human). [CR612178]	12,759	14,244	-1,49
RPL22L1	Homo sapiens ribosomal protein L22-like 1, mRNA (cDNA clone IMAGE:4865966). [BC049823]	12,458	13,945	-1,49

Supplemental Table 1
Morandi et al

AI090167	AI090167 qb33g12.x1 Soares_pregnant_uterus_NbHPU Homo sapiens cDNA clone IMAGE:1698118 3', mRNA sequence [AI090167]	6,421	7,909	-1,49
H2AFX	Homo sapiens H2A histone family, member X (H2AFX), mRNA [NM_002105]	11,902	13,391	-1,49
MGC24039	Homo sapiens hypothetical protein MGC24039, mRNA (cDNA clone IMAGE:4286826), complete cds. [BC020855]	6,425	7,912	-1,49
BU753102	BU753102 UI-1-BB1-aii-b-10-0-UI.s1 NCI_CGAP_Pl5 Homo sapiens cDNA clone UI-1-BB1-aii-b-10-0-UI 3', mRNA sequence [BU753102]	4,91	6,401	-1,49
BX457728	BX457728 BX457728 Homo sapiens NEUROBLASTOMA Homo sapiens cDNA clone CS0DA003YD09 3-PRIME, mRNA sequence [BX457728]	7,098	8,589	-1,49
ZBTB44	Homo sapiens zinc finger and BTB domain containing 44 (ZBTB44), mRNA [NM_014155]	6,088	7,579	-1,49
ABHD5	Homo sapiens abhydrolase domain containing 5 (ABHD5), mRNA [NM_016006]	8,075	9,566	-1,49
AI096653	AI096653 qb36a09.x1 Soares_pregnant_uterus_NbHPU Homo sapiens cDNA clone IMAGE:1698328 3', mRNA sequence [AI096653]	8,456	9,949	-1,49
GPX7	Homo sapiens glutathione peroxidase 7 (GPX7), mRNA [NM_015696]	10,436	11,928	-1,49
STK4	Homo sapiens serine/threonine kinase 4 (STK4), mRNA [NM_006282]	4,775	6,266	-1,49
U2AF1	Homo sapiens cDNA FLJ13527 fis, clone PLACE1006076. [AK023589]	5,823	7,317	-1,49
ZBTB25	Homo sapiens zinc finger and BTB domain containing 25 (ZBTB25), mRNA [NM_006977]	5,556	7,05	-1,49
AF354444	Homo sapiens IFP38 (IFP38) mRNA, complete cds. [AF354444]	6,907	8,403	-1,50
C20orf199	Homo sapiens cDNA FLJ42181 fis, clone THYMU2031368. [AK124175]	5,622	7,118	-1,50
MRPS30	Homo sapiens mitochondrial ribosomal protein S30 (MRPS30), nuclear gene encoding mitochondrial protein, mRNA [NM_016640]	7,574	9,07	-1,50
RP6-213H19.1	Homo sapiens serine/threonine protein kinase MST4 (MST4), transcript variant 1, mRNA [NM_016542]	8,399	9,894	-1,50
CNOT6	Homo sapiens CCR4-NOT transcription complex, subunit 6 (CNOT6), mRNA [NM_015455]	5,811	7,308	-1,50
THC2774217	AF264779 sporulation-induced transcript 4-associated protein SAPLa {Homo sapiens} (exp=-1; wgp=0; cg=0), partial (3%) [THC2774217]	8,594	10,092	-1,50
MDM4	Homo sapiens Mdm4, transformed 3T3 cell double minute 4, p53 binding protein (mouse) (MDM4), mRNA [NM_002393]	8,411	9,91	-1,50
EIF4A2	Homo sapiens eukaryotic translation initiation factor 4A, isoform 2, mRNA (cDNA clone IMAGE:5269135). [BC039344]	4,806	6,307	-1,50
AL036098	AL036098 DKFZp564N0923_r1 564 (synonym: hfbr2) Homo sapiens cDNA clone DKFZp564N0923 5', mRNA sequence [AL036098]	5,571	7,078	-1,51
MASTL	Homo sapiens microtubule associated serine/threonine kinase-like (MASTL), mRNA [NM_032844]	4,59	6,1	-1,51
UBE2D1	Homo sapiens ubiquitin-conjugating enzyme E2D 1 (UBC4/5 homolog, yeast) (UBE2D1), mRNA [NM_003338]	7,722	9,232	-1,51
C15orf23	Homo sapiens chromosome 15 open reading frame 23, mRNA (cDNA clone IMAGE:3952251), partial cds. [BC004543]	10,495	12,006	-1,51
-	chr2:174616493-174616674	7,616	9,129	-1,51
ENAH	Homo sapiens enabled homolog (Drosophila) (ENAH), transcript variant 2, mRNA [NM_018212]	6,712	8,225	-1,51
RABGEF1	Homo sapiens RAB guanine nucleotide exchange factor (GEF) 1 (RABGEF1), mRNA [NM_014504]	8,296	9,809	-1,51
CFLAR	Homo sapiens FLAME-1 mRNA, complete cds. [AF009616]	8,429	9,943	-1,51
THC2620401	AV696077 AV696077 GKC Homo sapiens cDNA clone GKCDTB08 5', mRNA sequence [AV696077]	5,756	7,27	-1,51
KCTD9	Homo sapiens clone FLB9630 PRO2603 mRNA, complete cds. [AF130091]	7,36	8,875	-1,52
NHLRC2	Homo sapiens mRNA; cDNA DKFZp451L096 (from clone DKFZp451L096). [BX647641]	6,374	7,889	-1,52

Supplemental Table 1
Morandi et al

SEPT9	Homo sapiens septin 9 (), mRNA [NM_006640]	6,972	8,488	-1,52
-	chr12:038147358-038147297	5,663	7,179	-1,52
-	chr2:232028068-232028127	7,069	8,59	-1,52
BE816155	BE816155 RC0-BN0198-220500-021-f04 BN0198 Homo sapiens cDNA, mRNA sequence [BE816155]	8,508	10,032	-1,52
CYBA	Homo sapiens cytochrome b-245, alpha polypeptide (CYBA), mRNA [NM_000101]	9,876	11,4	-1,52
AL562818	AL562818 Homo sapiens NEUROBLASTOMA COT 25-NORMALIZED Homo sapiens cDNA clone CS0DC017YH14 3-PRIME, mRNA sequence [AL562818]	5,933	7,46	-1,53
BQ374929	BQ374929 MR1-TN0045-060900-005-d11 TN0045 Homo sapiens cDNA, mRNA sequence [BQ374929]	10,052	11,579	-1,53
CCDC99	Homo sapiens coiled-coil domain containing 99 (CCDC99), mRNA [NM_017785]	7,669	9,196	-1,53
ANKRD44	Homo sapiens ankyrin repeat domain 44 (ANKRD44), mRNA [NM_153697]	6,139	7,667	-1,53
STK38L	Homo sapiens serine/threonine kinase 38 like (STK38L), mRNA [NM_015000]	7,742	9,271	-1,53
AA418814	AA418814 zw01a02.s1 Soares_NhHMPu_S1 Homo sapiens cDNA clone IMAGE:767978 3', mRNA sequence [AA418814]	9,324	10,854	-1,53
MNS1	Homo sapiens meiosis-specific nuclear structural 1 (MNS1), mRNA [NM_018365]	7,072	8,601	-1,53
HIST1H2BM	Homo sapiens histone cluster 1, H2bm (HIST1H2BM), mRNA [NM_003521]	8,739	10,273	-1,53
-	chr5:108092085-108092144	7,427	8,963	-1,54
AK023559	Homo sapiens cDNA FLJ13497 fis, clone PLACE1004518. [AK023559]	6,358	7,893	-1,54
NR2C1	Homo sapiens nuclear receptor subfamily 2, group C, member 1 (NR2C1), transcript variant 2, mRNA [NM_001032287]	7,438	8,973	-1,54
BF085047	BF085047 PM0-GN0018-130900-003-c12 GN0018 Homo sapiens cDNA, mRNA sequence [BF085047]	5,31	6,846	-1,54
-	chr10:044888819-044888878	8,021	9,558	-1,54
LOC402069	PREDICTED: Homo sapiens similar to 40S ribosomal protein S16 (LOC402069), mRNA [XR_019634]	10,523	12,06	-1,54
POLR2A	Homo sapiens polymerase (RNA) II (DNA directed) polypeptide A, 220kDa (POLR2A), mRNA [NM_000937]	4,33	5,867	-1,54
-	chr12:015484028-015484087	8,36	9,898	-1,54
ATAD2	Homo sapiens ATPase family, AAA domain containing 2 (ATAD2), mRNA [NM_014109]	6,955	8,493	-1,54
ARF1	Homo sapiens cDNA FLJ13741 fis, clone PLACE3000208. [AK023803]	8,805	10,345	-1,54
RAB27A	Homo sapiens RAB27A, member RAS oncogene family (RAB27A), transcript variant 1, mRNA [NM_004580]	5,746	7,286	-1,54
AJ009817	Homo sapiens mRNA for AMMECR1 protein, alternative exon 2. [AJ009817]	4,834	6,377	-1,54
C16orf55	Homo sapiens chromosome 16 open reading frame 55 (C16orf55), mRNA [NM_153025]	7,605	9,147	-1,54
AV739664	AV739664 CB Homo sapiens cDNA clone CBNAWB09 5', mRNA sequence [AV739664]	6,498	8,041	-1,54
LOC391367	PREDICTED: Homo sapiens similar to Glyceraldehyde-3-phosphate dehydrogenase (GAPDH) (LOC391367), mRNA [XR_019184]	6,453	7,997	-1,54
UBE2T	Homo sapiens ubiquitin-conjugating enzyme E2T (putative) (UBE2T), mRNA [NM_014176]	11,543	13,088	-1,55
SLC6A6	Homo sapiens solute carrier family 6 (neurotransmitter transporter, taurine), member 6 (SLC6A6), mRNA [NM_003043]	8,829	10,377	-1,55
LSM3	Homo sapiens LSM3 homolog, U6 small nuclear RNA associated (S. cerevisiae) (LSM3), mRNA [NM_014463]	11,61	13,16	-1,55
SYNCRIP	Homo sapiens synaptotagmin binding, cytoplasmic RNA interacting protein (SYNCRIP), mRNA [NM_006372]	10,416	11,966	-1,55
CENPH	Homo sapiens centromere protein H (CENPH), mRNA [NM_022909]	7,911	9,461	-1,55
GMNN	Homo sapiens geminin, DNA replication inhibitor (GMNN), mRNA [NM_015895]	10,747	12,298	-1,55

Supplemental Table 1
Morandi et al

AL079999	AL079999 DKFZp586P2018_r1 586 (synonym: hute1) Homo sapiens cDNA clone DKFZp586P2018 5', mRNA sequence [AL079999]	5,869	7,422	-1,55
X05126	Human fibroblast mRNA fragment with Alu sequence (pRHF11). [X05126]	6,26	7,815	-1,56
-	chr7:072331752-072331693	5,753	7,31	-1,56
AI857589	AI857589 wk95b07.x1 NCI_CGAP_Lu19 Homo sapiens cDNA clone IMAGE:2423125 3' similar to contains Alu repetitive element;contains element MER22 repetitive element ;, mRNA sequence [AI857589]	5,454	7,011	-1,56
FAM119A	Homo sapiens family with sequence similarity 119, member A (FAM119A), mRNA [NM_145280]	7,902	9,459	-1,56
GALNACT-2	Homo sapiens chondroitin sulfate GalNAcT-2 (GALNACT-2), mRNA [NM_018590]	4,677	6,235	-1,56
-	chrX_random:000899134-000899193	5,718	7,276	-1,56
BX329117	BX329117 BX329117 Homo sapiens PLACENTA COT 25-NORMALIZED Homo sapiens cDNA clone CS0DI084YH16 3-PRIME, mRNA sequence [BX329117]	5,292	6,85	-1,56
STT3B	Homo sapiens STT3, subunit of the oligosaccharyltransferase complex, homolog B (S. cerevisiae) (STT3B), mRNA [NM_178862]	6,908	8,467	-1,56
EAF2	Homo sapiens ELL associated factor 2 (EAF2), mRNA [NM_018456]	6,574	8,133	-1,56
ANKHD1	Homo sapiens ankyrin repeat and KH domain containing 1 (ANKHD1), transcript variant 3, mRNA [NM_024668]	8,933	10,497	-1,56
FLJ25416	Homo sapiens hypothetical protein FLJ25416 (FLJ25416), mRNA [NM_145018]	6,803	8,366	-1,56
BX415272	BX415272 Homo sapiens THYMUS Homo sapiens cDNA clone CS0CAP005YK02 3-PRIME, mRNA sequence [BX415272]	4,892	6,463	-1,57
ITGA4	Homo sapiens integrin, alpha 4 (antigen CD49D, alpha 4 subunit of VLA-4 receptor) (ITGA4), mRNA [NM_000885]	5	6,572	-1,57
TBRG1	Homo sapiens transforming growth factor beta regulator 1, mRNA (cDNA clone IMAGE:5212572), complete cds. [BC032312]	8,883	10,456	-1,57
ZNF516	Zinc finger protein 516. [Source:Uniprot/SWISSPROT;Acc:Q92618] [ENST00000217537]	7,564	9,14	-1,58
EIF4EBP1	Homo sapiens eukaryotic translation initiation factor 4E binding protein 1 (EIF4EBP1), mRNA [NM_004095]	11,493	13,069	-1,58
ZFY	Homo sapiens zinc finger protein, Y-linked (ZFY), mRNA [NM_003411]	5,554	7,13	-1,58
THC2701748	Q5SVL1_HUMAN (Q5SVL1) Caspase 7, apoptosis-related cysteine protease, partial (25%) [THC2701748]	4,962	6,54	-1,58
BU684362	BU684362 UI-CF-EN0-aco-n-23-0-UI.s1 UI-CF-EN0 Homo sapiens cDNA clone UI-CF-EN0-aco-n-23-0-UI 3', mRNA sequence [BU684362]	4,948	6,531	-1,58
THC2517184	1405340A protein 40kD. {Mus musculus} (exp=-1; wgp=0; cg=0), partial (48%) [THC2594767]	8,37	9,954	-1,58
ECT2	Homo sapiens epithelial cell transforming sequence 2 oncogene (ECT2), mRNA [NM_018098]	10,258	11,844	-1,59
THC2525408	AV738929 AV738929 CB Homo sapiens cDNA clone CBCCMG04 5', mRNA sequence [AV738929]	6,82	8,407	-1,59
THC2552359	Q4RCF0_TETNG (Q4RCF0) Chromosome undetermined SCAF19066, whole genome shotgun sequence. (Fragment), partial (5%) [THC2552359]	7,716	9,302	-1,59
RNASEH2A	Homo sapiens ribonuclease H2, subunit A (RNASEH2A), mRNA [NM_006397]	10,469	12,058	-1,59
SMPDL3A	Homo sapiens sphingomyelin phosphodiesterase, acid-like 3A (SMPDL3A), mRNA [NM_006714]	5,565	7,154	-1,59
BF378976	BF378976 QV1-UM0099-020400-141-b12_1 UM0099 Homo sapiens cDNA, mRNA sequence [BF378976]	8,635	10,23	-1,60
ENST0000022927(	Triosephosphate isomerase (EC 5.3.1.1) (TIM) (Triose-phosphate isomerase). [Source:Uniprot/SWISSPROT;Acc:P60174] [ENST00000229270]	10,64	12,237	-1,60
BC007809	Homo sapiens cDNA clone IMAGE:4298560. [BC007809]	9,185	10,787	-1,60
NR2C2	Homo sapiens nuclear receptor subfamily 2, group C, member 2 (NR2C2), mRNA [NM_003298]	6,929	8,531	-1,60

Supplemental Table 1
Morandi et al

KNTC2	Homo sapiens kinetochore associated 2 (KNTC2), mRNA [NM_006101]	10,327	11,932	-1,60
PHC3	Homo sapiens polyhomeotic homolog 3 (Drosophila) (PHC3), mRNA [NM_024947]	6,518	8,123	-1,61
ENST00000294383	Ubiquitin carboxyl-terminal hydrolase 24 (EC 3.1.2.15) (Ubiquitin thioesterase 24) (Ubiquitin-specific-processing protease 24) (Deubiquitinating enzyme 24). [Source:Uniprot/SWISSPROT;Acc:Q9UPU5] [ENST00000294383]	8,327	9,935	-1,61
SIN3B	Homo sapiens SIN3 homolog B, transcription regulator (yeast), mRNA (cDNA clone IMAGE:3923074), partial cds. [BC025026]	7,952	9,559	-1,61
HIST1H2AJ	Homo sapiens histone cluster 1, H2aj (HIST1H2AJ), mRNA [NM_021066]	9,603	11,211	-1,61
CA311162	CA311162 UI-CF-FN0-afc-l-13-0-UI.s1 UI-CF-FN0 Homo sapiens cDNA clone UI-CF-FN0-afc-l-13-0-UI 3', mRNA sequence [CA311162]	6,006	7,616	-1,61
TGFB1	Homo sapiens transforming growth factor, beta 1 (Camurati-Engelmann disease) (TGFB1), mRNA [NM_000660]	7,153	8,765	-1,61
TRIO	Homo sapiens triple functional domain (PTPRF interacting) (TRIO), mRNA [NM_007118]	6,631	8,244	-1,61
THC2674691	BOLA2_HUMAN (Q9H3K6) BolA-like protein 2, partial (33%) [THC2674691]	7,638	9,253	-1,62
ENST00000357303	CDNA FLJ37034 fis, clone BRACE2011478 (Hypothetical protein FLJ37034). [Source:Uniprot/SPTREMBL;Acc:Q8N9J7] [ENST00000357303]	5,514	7,129	-1,62
-	chr2:058332840-058332781	4,964	6,581	-1,62
SPFH1	Homo sapiens SPFH domain family, member 1 (SPFH1), mRNA [NM_006459]	5,802	7,419	-1,62
-	chrX:003645501-003645562	7,8	9,418	-1,62
AI537201	AI537201 tp06e06.x1 NCI_CGAP_Gas4 Homo sapiens cDNA clone IMAGE:2187010 3', mRNA sequence [AI537201]	8,676	10,294	-1,62
AK026485	Homo sapiens cDNA: FLJ22832 fis, clone KAIA4195. [AK026485]	4,716	6,334	-1,62
KIAA1794	Homo sapiens KIAA1794 (KIAA1794), mRNA [NM_018193]	9,404	11,022	-1,62
KTI12	Homo sapiens KTI12 homolog, chromatin associated (S. cerevisiae) (KTI12), mRNA [NM_138417]	6,831	8,449	-1,62
THC2578055	Q53HR0_HUMAN (Q53HR0) Eukaryotic translation initiation factor 3, subunit 3 gamma, 40kDa variant (Fragment), partial (39%) [THC2578055]	6,422	8,041	-1,62
RPSAP10	PREDICTED: Homo sapiens ribosomal protein SA pseudogene 10 (RPSAP10), mRNA [XR_019235]	10,533	12,156	-1,62
BE970058	BE970058 601680172F1 NIH_MGC_78 Homo sapiens cDNA clone IMAGE:3950464 5', mRNA sequence [BE970058]	8,928	10,554	-1,63
-	chr13:049571595-049571654	7,394	9,02	-1,63
TMEM167	Homo sapiens transmembrane protein 167 (TMEM167), mRNA [NM_174909]	8,849	10,475	-1,63
NUDT1	Homo sapiens nudix (nucleoside diphosphate linked moiety X)-type motif 1 (NUDT1), transcript variant 2B, mRNA [NM_198949]	11,881	13,51	-1,63
UBE2S	Homo sapiens ubiquitin-conjugating enzyme E2S (UBE2S), mRNA [NM_014501]	12,701	14,331	-1,63
FLJ20186	Homo sapiens hypothetical protein FLJ20186 (FLJ20186), transcript variant 2, mRNA [NM_017702]	9,878	11,509	-1,63
LOC644728	PREDICTED: Homo sapiens similar to Glyceraldehyde-3-phosphate dehydrogenase (GAPDH) (38 kDa BFA-dependent ADP-ribosylation substrate) (BARS-38) (LOC644728), mRNA [XR_018251]	9,497	11,129	-1,63
MAN1A1	Homo sapiens mannosidase, alpha, class 1A, member 1 (MAN1A1), mRNA [NM_005907]	5,543	7,175	-1,63
MAX	Homo sapiens MYC associated factor X (MAX), transcript variant 4, mRNA [NM_145114]	5,283	6,915	-1,63
PTTG3	Homo sapiens pituitary tumor-transforming 3 (PTTG3) on chromosome 8 [NR_002734]	5,615	7,248	-1,63
HIST1H3J	Homo sapiens histone cluster 1, H3j (HIST1H3J), mRNA [NM_003535]	6,721	8,355	-1,63
GNG5	Homo sapiens guanine nucleotide binding protein (G protein), gamma 5 (GNG5), mRNA [NM_005274]	9,477	11,112	-1,64

Supplemental Table 1
Morandi et al

TPM1	Homo sapiens tropomyosin 1 (alpha) (TPM1), transcript variant 5, mRNA [NM_000366]	10,938	12,573	-1,64
AL571926	AL571926 Homo sapiens PLACENTA COT 25-NORMALIZED Homo sapiens cDNA clone CS0DI029YJ06 3-PRIME, mRNA sequence [AL571926]	7,696	9,336	-1,64
BE168511	BE168511 QV3-HT0514-220300-127-d06 HT0514 Homo sapiens cDNA, mRNA sequence [BE168511]	5,237	6,879	-1,64
HIST2H2BE	Homo sapiens histone cluster 2, H2be (HIST2H2BE), mRNA [NM_003528]	8,409	10,05	-1,64
PDK1	Homo sapiens pyruvate dehydrogenase kinase, isozyme 1 (PDK1), nuclear gene encoding mitochondrial protein, mRNA [NM_002610]	5,05	6,693	-1,64
SMCHD1	Homo sapiens cDNA FLJ44350 fis, clone TRACH3006228. [AK126324]	9,292	10,936	-1,64
WDR76	Homo sapiens WD repeat domain 76 (WDR76), mRNA [NM_024908]	4,958	6,601	-1,64
BM968705	BM968705 UI-CF-DU1-aak-f-20-0-UI.s1 UI-CF-DU1 Homo sapiens cDNA clone UI-CF-DU1-aak-f-20-0-UI 3', mRNA sequence [BM968705]	5,861	7,505	-1,64
BE739632	BE739632 601556329T1 NIH_MGC_58 Homo sapiens cDNA clone IMAGE:3826255 3', mRNA sequence [BE739632]	6,859	8,507	-1,65
THC2501636	ALU1_HUMAN (P39188) Alu subfamily J sequence contamination warning entry, partial (14%) [THC2501636]	6,606	8,256	-1,65
THC2502237	Q69QZ1_ORYSA (Q69QZ1) F-box protein-like, partial (7%) [THC2502237]	7,083	8,734	-1,65
TMPO	Homo sapiens thymopoietin (TMPO), transcript variant 2, mRNA [NM_001032283]	6,409	8,063	-1,65
EHD1	Homo sapiens EH-domain containing protein testilin mRNA, complete cds. [AF099011]	7,149	8,804	-1,66
LOC285074	Homo sapiens hypothetical protein LOC285074 (LOC285074), mRNA [NM_001012626]	9,439	11,094	-1,66
FAM54A	Homo sapiens family with sequence similarity 54, member A (FAM54A), mRNA [NM_138419]	6,829	8,485	-1,66
THC2717907	Q4IZP3_AZOVI (Q4IZP3) Outer membrane porin, partial (3%) [THC2717907]	8,097	9,753	-1,66
TMEM48	Homo sapiens transmembrane protein 48 (TMEM48), mRNA [NM_018087]	7,662	9,318	-1,66
CA948108	CA948108 iq21e07.x1 HR85 islet Homo sapiens cDNA clone IMAGE: 3', mRNA sequence [CA948108]	5,025	6,682	-1,66
CDC40	Homo sapiens cell division cycle 40 homolog (S. cerevisiae) (CDC40), mRNA [NM_015891]	7,248	8,905	-1,66
WBSCR16	Homo sapiens Williams-Beuren syndrome chromosome region 16, mRNA (cDNA clone MGC:49849 IMAGE:5785681), complete cds. [BC040695]	5,407	7,072	-1,67
-	chr17:037939683-037939742	11,459	13,126	-1,67
LOC642203	PREDICTED: Homo sapiens similar to Glyceraldehyde-3-phosphate dehydrogenase (GAPDH) (LOC642203), mRNA [XR_018113]	10,599	12,266	-1,67
MSI2	Homo sapiens musashi homolog 2 (Drosophila) (MSI2), transcript variant 1, mRNA [NM_138962]	7,922	9,589	-1,67
BC068044	Homo sapiens cDNA clone IMAGE:6380649, containing frame-shift errors. [BC068044]	8,37	10,039	-1,67
HIST3H2A	Homo sapiens histone cluster 3, H2a (HIST3H2A), mRNA [NM_033445]	10,479	12,149	-1,67
BID	Homo sapiens BH3 interacting domain death agonist (BID), transcript variant 1, mRNA [NM_197966]	11,135	12,806	-1,67
PGAP1	Homo sapiens GPI deacylase (PGAP1), mRNA [NM_024989]	5,358	7,029	-1,67
CKS1B	Homo sapiens CDC28 protein kinase regulatory subunit 1B (CKS1B), mRNA [NM_001826]	12,511	14,186	-1,67
PCGF5	Homo sapiens polycomb group ring finger 5, mRNA (cDNA clone IMAGE:3640258), complete cds. [BC007377]	6,032	7,706	-1,67
PIF1	Homo sapiens PIF1 5'-to-3' DNA helicase homolog (S. cerevisiae) (PIF1), mRNA [NM_025049]	9,044	10,718	-1,67
HUS1B	Homo sapiens HUS1 checkpoint homolog b (S. pombe) (HUS1B), mRNA [NM_148959]	5,147	6,824	-1,68

Supplemental Table 1
Morandi et al

ODC1	Homo sapiens ornithine decarboxylase 1 (ODC1), mRNA [NM_002539]	12,92	14,598	-1,68
RPS15A	Homo sapiens ribosomal protein S15a (RPS15A), transcript variant 2, mRNA [NM_001019]	13,493	15,171	-1,68
-	chr15:059932948-059933007	5,964	7,643	-1,68
ROCK1	Homo sapiens Rho-associated, coiled-coil containing protein kinase 1 (ROCK1), mRNA [NM_005406]	6,496	8,176	-1,68
U52054	Human S6 H-8 mRNA expressed in chromosome 6-suppressed melanoma cells. [U52054]	7,112	8,792	-1,68
LOC130865	PREDICTED: Homo sapiens similar to 60S ribosomal protein L26 (Silica-induced gene 20 protein) (SIG-20) (LOC130865), mRNA [XR_019454]	8,332	10,013	-1,68
U25029	Human glucocorticoid receptor alpha mRNA, variant 3' UTR. [U25029]	7,695	9,378	-1,68
LOC645161	PREDICTED: Homo sapiens similar to 60S ribosomal protein L12 (LOC645161), mRNA [XM_928198]	8,505	10,188	-1,68
HIST1H2BN	Homo sapiens histone cluster 1, H2bn (HIST1H2BN), mRNA [NM_003520]	9,053	10,738	-1,69
PPP4R2	Homo sapiens protein phosphatase 4, regulatory subunit 2 (PPP4R2), mRNA [NM_174907]	6,171	7,856	-1,69
LOC646626	PREDICTED: Homo sapiens hypothetical LOC646626 (LOC646626), mRNA [XM_942822]	7,241	8,929	-1,69
BCL2L13	Homo sapiens BCL2-like 13 (apoptosis facilitator) (BCL2L13), nuclear gene encoding mitochondrial protein, mRNA [NM_015367]	6,438	8,128	-1,69
CN391963	CN391963 17000599942841 GRN_PRENEU Homo sapiens cDNA 5', mRNA sequence [CN391963]	4,735	6,425	-1,69
EDEM1	Homo sapiens ER degradation enhancer, mannosidase alpha-like 1 (EDEM1), mRNA [NM_014674]	6,168	7,859	-1,69
RNF141	Homo sapiens ring finger protein 141 (RNF141), mRNA [NM_016422]	8,564	10,255	-1,69
SHCBP1	Homo sapiens SHC SH2-domain binding protein 1 (SHCBP1), mRNA [NM_024745]	5,999	7,692	-1,69
ZMYM2	Homo sapiens mRNA; cDNA DKFZp564B162 (from clone DKFZp564B162). [AL136621]	8,512	10,204	-1,69
AI922845	wo14g02.x1 NCI_CGAP_Pan1 Homo sapiens cDNA clone IMAGE:2455346 3' similar to gb:J04543 ANNEXIN VII (HUMAN);, mRNA sequence [AI922845]	6,631	8,324	-1,69
RPL39L	Homo sapiens ribosomal protein L39-like (RPL39L), mRNA [NM_052969]	9,084	10,782	-1,70
EIF2C4	Homo sapiens eukaryotic translation initiation factor 2C, 4 (EIF2C4), mRNA [NM_017629]	7,01	8,709	-1,70
HIST1H2BE	Homo sapiens histone cluster 1, H2be (HIST1H2BE), mRNA [NM_003523]	10,094	11,796	-1,70
ARPC1B	603081610T1 NIH_MGC_120 Homo sapiens cDNA clone IMAGE:5220795 3', mRNA sequence [BI521983]	5,876	7,58	-1,70
CEP27	Homo sapiens centrosomal protein 27kDa (CEP27), mRNA [NM_018097]	6,971	8,675	-1,70
PLP2	Homo sapiens proteolipid protein 2 (colonic epithelium-enriched) (PLP2), mRNA [NM_002668]	8,771	10,476	-1,71
THC2517558	BC026690 CD97 antigen, isoform 2 precursor {Homo sapiens} (exp=-1; wgp=0; cg=0), partial (15%) [THC2517558]	7,59	9,294	-1,71
AA291137	AA291137 zs46b08.s1 NCI_CGAP_GCB1 Homo sapiens cDNA clone IMAGE:700503 3', mRNA sequence [AA291137]	5,794	7,5	-1,71
SERPINB8	Homo sapiens serpin peptidase inhibitor, clade B (ovalbumin), member 8 (SERPINB8), transcript variant 2, mRNA [NM_198833]	8,449	10,155	-1,71
THC2758091	chr1:169722063-169722004	5,168	6,874	-1,71
NIN	Homo sapiens ninein (GSK3B interacting protein) (NIN), transcript variant 4, mRNA [NM_016350]	7,713	9,421	-1,71
ENST0000038299(	chr16:221400-221459	4,688	6,398	-1,71
GLRX	Homo sapiens glutaredoxin (thioltransferase) (GLRX), mRNA [NM_002064]	10,863	12,573	-1,71
CDCA3	Homo sapiens cell division cycle associated 3 (CDCA3), mRNA [NM_031299]	7,362	9,077	-1,72

Supplemental Table 1
Morandi et al

CDKN3	Homo sapiens cyclin-dependent kinase inhibitor 3 (CDK2-associated dual specificity phosphatase) (CDKN3), mRNA [NM_005192]	8,674	10,389	-1,72
T12588	CHR90108 Chromosome 9 exon II Homo sapiens cDNA clone P94_53 5' and 3', mRNA sequence [T12588]	10,738	12,455	-1,72
CA314451	CA314451 UI-CF-FN0-afh-a-09-0-UI.s1 UI-CF-FN0 Homo sapiens cDNA clone UI-CF-FN0-afh-a-09-0-UI 3', mRNA sequence [CA314451]	8,305	10,024	-1,72
THC2651047	chr12:67327784-67327725	4,769	6,488	-1,72
DBF4	Homo sapiens DBF4 homolog (S. cerevisiae) (DBF4), mRNA [NM_006716]	7,237	8,958	-1,72
FAM101B	Homo sapiens family with sequence similarity 101, member B (FAM101B), mRNA [NM_182705]	7,67	9,392	-1,72
LOC652411	PREDICTED: Homo sapiens similar to laminin receptor 1 (ribosomal protein SA) (LOC652411), mRNA [XR_019314]	8,233	9,956	-1,72
THC2640030	chr2:32244187-32244128	4,602	6,324	-1,72
UBE2W	Homo sapiens ubiquitin-conjugating enzyme E2W (putative) (UBE2W), transcript variant 1, mRNA [NM_001001481]	6,396	8,119	-1,72
-	chr15:056766772-056766831	6,841	8,564	-1,72
C10orf12	Homo sapiens chromosome 10 open reading frame 12 (C10orf12), mRNA [NM_015652]	4,579	6,303	-1,72
FANCB	Homo sapiens Fanconi anemia, complementation group B (FANCB), transcript variant 1, mRNA [NM_001018113]	5,665	7,391	-1,73
KLHDC7B	Homo sapiens kelch domain containing 7B (KLHDC7B), mRNA [NM_138433]	7,055	8,781	-1,73
BE156557	BE156557 QV0-HT0368-090200-099-d10 HT0368 Homo sapiens cDNA, mRNA sequence [BE156557]	6,406	8,136	-1,73
PRC1	Homo sapiens protein regulator of cytokinesis 1 (PRC1), transcript variant 1, mRNA [NM_003981]	10,892	12,623	-1,73
PTPRJ	Homo sapiens protein tyrosine phosphatase, receptor type, J (PTPRJ), mRNA [NM_002843]	5,913	7,646	-1,73
RP11-114G1.1	Homo sapiens Sp3 transcription factor pseudogene, mRNA (cDNA clone MGC:43345 IMAGE:5266022), complete cds. [BC036697]	6,405	8,139	-1,73
THC2713795	Q9F8M7_CARHY (Q9F8M7) DTDP-glucose 4,6-dehydratase (Fragment), partial (11%) [THC2713795]	5,364	7,098	-1,73
COTL1	Homo sapiens coactosin-like 1 (Dictyostelium) (COTL1), mRNA [NM_021149]	7,238	8,974	-1,74
NP1247838	GB|AL162389.21|CAH73163.1 ribosomal protein L36a pseudogene 6 [Homo sapiens] [NP1247838]	4,66	6,396	-1,74
BF475893	BF475893 nac42c01.x1 Lupski_sciatic_nerve Homo sapiens cDNA clone IMAGE:3395713 3', mRNA sequence [BF475893]	6,035	7,777	-1,74
IER5	Homo sapiens immediate early response 5 (IER5), mRNA [NM_016545]	12,793	14,536	-1,74
PRKAA1	Homo sapiens protein kinase, AMP-activated, alpha 1 catalytic subunit (PRKAA1), transcript variant 2, mRNA [NM_206907]	7,283	9,025	-1,74
ENST00000247761	chr3:188100807-188100866	6,71	8,453	-1,74
PABPC3	Homo sapiens poly(A) binding protein, cytoplasmic 3 (PABPC3), mRNA [NM_030979]	7,778	9,521	-1,74
BE696323	BE696323 RC3-CT0347-010700-019-c08 CT0347 Homo sapiens cDNA, mRNA sequence [BE696323]	5,372	7,116	-1,74
-	chr6:114290669-114290610	8,11	9,855	-1,75
THC2585854	1T5A_A Chain A, Human Pyruvate Kinase M2. {Homo sapiens} (exp=-1; wgp=0; cg=0), partial (11%) [THC2585854]	6,647	8,392	-1,75
-	chr19:063419579-063419638	6,048	7,796	-1,75
EIF2C2	Homo sapiens eukaryotic translation initiation factor 2C, 2 (EIF2C2), mRNA [NM_012154]	9,106	10,855	-1,75
-	chr1:119471116-119471056	7,401	9,152	-1,75
ATP11C	Homo sapiens ATPase, Class VI, type 11C (ATP11C), transcript variant 2, mRNA [NM_001010986]	5,428	7,179	-1,75
BG001037	BG001037 RC5-GN0132-131100-012-E05 GN0132 Homo sapiens cDNA, mRNA sequence [BG001037]	6,856	8,607	-1,75
CENPM	Homo sapiens centromere protein M (CENPM), transcript variant 2, mRNA [NM_001002876]	7,093	8,847	-1,75
SKIL	Homo sapiens SKI-like oncogene (SKIL), mRNA [NM_005414]	6,395	8,149	-1,75

GLIPR1	Homo sapiens GLI pathogenesis-related 1 (glioma) (GLIPR1), mRNA [NM_006851]	7,321	9,076	-1,76
PHCA	Homo sapiens phytoceramidase, alkaline (PHCA), mRNA [NM_018367]	4,187	5,945	-1,76
ALG13	Homo sapiens asparagine-linked glycosylation 13 homolog (S. cerevisiae) (ALG13), mRNA [NM_018466]	8,216	9,975	-1,76
ZWINT	Homo sapiens ZW10 interactor (ZWINT), transcript variant 4, mRNA [NM_001005414]	9,343	11,105	-1,76
BF803156	BF803156 CM0-CI0139-011100-675-h03 CI0139 Homo sapiens cDNA, mRNA sequence [BF803156]	6,465	8,228	-1,76
LOC648378	PREDICTED: Homo sapiens similar to ribosomal protein S14 (LOC648378), mRNA [XR_018303]	10,593	12,36	-1,77
BX449754	BX449754 Homo sapiens NEUROBLASTOMA Homo sapiens cDNA clone CS0DA007YA13 3-PRIME, mRNA sequence [BX449754]	4,619	6,39	-1,77
WDR62	Homo sapiens WD repeat domain 62 (WDR62), mRNA [NM_173636]	7,061	8,832	-1,77
-	chr7:032478055-032478204	9,795	11,569	-1,77
CNN2	Homo sapiens calponin 2 (CNN2), transcript variant 1, mRNA [NM_004368]	7,535	9,308	-1,77
FLJ20186	Homo sapiens hypothetical protein FLJ20186 (FLJ20186), transcript variant 2, mRNA [NM_017702]	11,079	12,854	-1,78
NFIL3	Homo sapiens nuclear factor, interleukin 3 regulated (NFIL3), mRNA [NM_005384]	10,96	12,739	-1,78
CV339166	CV339166 MR0-BT2503-050601-101-a04 BT2503 Homo sapiens cDNA, mRNA sequence [CV339166]	4,788	6,571	-1,78
-	chrX:134988318-134988377	5,917	7,703	-1,79
HIST1H2BL	Homo sapiens histone cluster 1, H2bl (HIST1H2BL), mRNA [NM_003519]	11,035	12,82	-1,79
CEP152	Homo sapiens centrosomal protein 152kDa (CEP152), mRNA [NM_014985]	6,436	8,223	-1,79
AL566187	AL566187 Homo sapiens FETAL BRAIN Homo sapiens cDNA clone CS0DF022YK06 3-PRIME, mRNA sequence [AL566187]	8,846	10,639	-1,79
LOC196264	QQRG2966 (Hypothetical protein LOC196264). [Source:Uniprot/SPTREMBL;Acc:Q6UWV2] [ENST00000278949]	6,508	8,301	-1,79
SMC4	Homo sapiens structural maintenance of chromosomes 4 (SMC4), transcript variant 1, mRNA [NM_005496]	8,894	10,689	-1,80
THC2694227	chr2:149166620-149166679	4,828	6,623	-1,80
DYNLL2	Homo sapiens dynein, light chain, LC8-type 2 (DYNLL2), mRNA [NM_080677]	10,54	12,338	-1,80
AJ293393	Homo sapiens mRNA differentially expressed in malignant melanoma, clone MM D3. [AJ293393]	6,034	7,834	-1,80
CEBPG	Homo sapiens CCAAT/enhancer binding protein (C/EBP), gamma (CEBPG), mRNA [NM_001806]	10,102	11,902	-1,80
-	chr9:094036521-094036462	5,324	7,126	-1,80
CF529502	CF529502 UI-1-BC1p-ash-d-10-0-UI.s1 NCI_CGAP_Pl3 Homo sapiens cDNA clone UI-1-BC1p-ash-d-10-0-UI 3', mRNA sequence [CF529502]	9,337	11,143	-1,81
-	chr5:032258029-032258093	7,268	9,075	-1,81
-	chr15:068128339-068128398	7,359	9,165	-1,81
-	chr1:042730340-042730399	9,973	11,781	-1,81
LOC730850	PREDICTED: Homo sapiens similar to peptidylprolyl isomerase A isoform 1 (LOC730850), mRNA [XR_015255]	7,377	9,186	-1,81
FEM1C	Homo sapiens fem-1 homolog c (C. elegans) (FEM1C), mRNA [NM_020177]	4,422	6,233	-1,81
ENDOG	Homo sapiens endonuclease G (ENDOG), nuclear gene encoding mitochondrial protein, mRNA [NM_004435]	10,283	12,095	-1,81
HIST1H2BF	Homo sapiens histone cluster 1, H2bf (HIST1H2BF), mRNA [NM_003522]	10,285	12,097	-1,81
THC2620595	chr9:7067159-7067218	6,214	8,026	-1,81
HIST1H2AH	Homo sapiens histone cluster 1, H2ah (HIST1H2AH), mRNA [NM_080596]	10,295	12,114	-1,82
CCNL1	Homo sapiens cyclin L1 (CCNL1), mRNA [NM_020307]	10,667	12,488	-1,82
PA2G4	Homo sapiens proliferation-associated 2G4, 38kDa (PA2G4), mRNA [NM_006191]	5,331	7,15	-1,82

Supplemental Table 1
Morandi et al

PIGA	Homo sapiens phosphatidylinositol glycan anchor biosynthesis, class A (paroxysmal nocturnal hemoglobinuria) (PIGA), transcript variant 1, mRNA [NM_002641]	4,389	6,209	-1,82
CD44	Homo sapiens CD44 molecule (Indian blood group) (CD44), transcript variant 1, mRNA [NM_000610]	11,208	13,033	-1,83
FAM83D	Homo sapiens family with sequence similarity 83, member D (FAM83D), mRNA [NM_030919]	6,64	8,466	-1,83
AF116702	Homo sapiens PRO2446 mRNA, complete cds. [AF116702]	6,642	8,468	-1,83
MICB	Homo sapiens MHC class I polypeptide-related sequence B (MICB), mRNA [NM_005931]	6,851	8,683	-1,83
GMFB	Homo sapiens glia maturation factor, beta (GMFB), mRNA [NM_004124]	6,386	8,218	-1,83
THC2519484	S72008 CDC10 homolog {Homo sapiens} (exp=-1; wgp=0; cg=0), partial (9%) [THC2519484]	4,643	6,476	-1,83
CRSP3	Homo sapiens cofactor required for Sp1 transcriptional activation, subunit 3, 130kDa (CRSP3), transcript variant 2, mRNA [NM_015979]	5,699	7,535	-1,84
THC2497326	chr12:51687676-51687617	4,856	6,691	-1,84
THC2768767	XM_362611 predicted protein {Magnaporthe grisea 70-15} (exp=0; wgp=1; cg=0), partial (13%) [THC2768767]	8,456	10,293	-1,84
MTP18	Homo sapiens mitochondrial protein 18 kDa (MTP18), nuclear gene encoding mitochondrial protein, transcript variant 1, mRNA [NM_016498]	7,69	9,527	-1,84
-	chr19:000642078-000642137	8,306	10,144	-1,84
IL17RA	Homo sapiens interleukin 17 receptor A (IL17RA), mRNA [NM_014339]	8,65	10,49	-1,84
BAG4	Homo sapiens BCL2-associated athanogene 4 (BAG4), mRNA [NM_004874]	4,76	6,606	-1,85
FLJ39660	Homo sapiens mRNA; cDNA DKFZp434P055 (from clone DKFZp434P055). [AL834537]	7,022	8,872	-1,85
TCP11L2	Homo sapiens t-complex 11 (mouse)-like 2 (TCP11L2), mRNA [NM_152772]	5,174	7,024	-1,85
EAF1	Homo sapiens ELL associated factor 1 (EAF1), mRNA [NM_033083]	4,773	6,625	-1,85
SPBC24	Homo sapiens spindle pole body component 24 homolog (S. cerevisiae) (SPBC24), mRNA [NM_182513]	6,75	8,602	-1,85
-	chr3:151184897-151184960	5,893	7,746	-1,85
AK021546	Homo sapiens cDNA FLJ11484 fis, clone HEMBA1001835. [AK021546]	5,193	7,049	-1,86
-	chrX:067908109-067908168	6,551	8,409	-1,86
FANCA	Homo sapiens Fanconi anemia, complementation group A (FANCA), transcript variant 1, mRNA [NM_000135]	7,582	9,441	-1,86
RPE	Homo sapiens ribulose-5-phosphate-3-epimerase (RPE), transcript variant 2, mRNA [NM_006916]	8,217	10,075	-1,86
AURKA	Homo sapiens aurora kinase A (AURKA), transcript variant 1, mRNA [NM_198433]	8,127	9,987	-1,86
PPP1R15B	Homo sapiens protein phosphatase 1, regulatory (inhibitor) subunit 15B (PPP1R15B), mRNA [NM_032833]	6,041	7,903	-1,86
AK093691	Homo sapiens cDNA FLJ36372 fis, clone THYMU2008072. [AK093691]	6,348	8,211	-1,86
SPECC1	Homo sapiens sperm antigen with calponin homology and coiled-coil domains 1 (SPECC1), transcript variant NSP5beta3alpha, mRNA [NM_152904]	8,66	10,529	-1,87
AV739735	AV739735 CB Homo sapiens cDNA clone CBCCMH08 5', mRNA sequence [AV739735]	8,42	10,292	-1,87
FTHL17	Homo sapiens ferritin, heavy polypeptide-like 17 (FTHL17), mRNA [NM_031894]	10,164	12,038	-1,87
ENST00000323198	similar to Ubiquitin-conjugating enzyme E2 C (Ubiquitin-protein ligase C) (Ubiquitin carrier protein C) (UbcH10) (LOC648937), mRNA [Source:RefSeq_dna;Acc:XR_018466] [ENST00000323198]	4,794	6,671	-1,88
-	chr22:019635752-019635693	5,823	7,701	-1,88
CA306742	CA306742 UI-H-FT1-bht-f-03-0-UI.s1 NCI_CGAP_FT1 Homo sapiens cDNA clone UI-H-FT1-bht-f-03-0-UI 3', mRNA sequence [CA306742]	5,335	7,218	-1,88

Supplemental Table 1
Morandi et al

EZH2	Homo sapiens enhancer of zeste homolog 2 (Drosophila) (EZH2), transcript variant 1, mRNA [NM_004456]	10,566	12,453	-1,89
LOC392522	PREDICTED: Homo sapiens similar to ribosomal protein L18a (LOC392522), mRNA [XR_018292]	11,255	13,143	-1,89
ZNF197	Homo sapiens zinc finger protein 197 (ZNF197), transcript variant 1, mRNA [NM_006991]	6,189	8,077	-1,89
MELK	Homo sapiens maternal embryonic leucine zipper kinase (MELK), mRNA [NM_014791]	7,226	9,117	-1,89
HIST1H2AK	Homo sapiens histone cluster 1, H2ak (HIST1H2AK), mRNA [NM_003510]	10,963	12,855	-1,89
AJ227863	Homo sapiens partial mRNA; ID YG39-2B. [AJ227863]	9,019	10,915	-1,90
BG007597	BG007597 QV4-GN0250-281100-608-g10 GN0250 Homo sapiens cDNA, mRNA sequence [BG007597]	7,074	8,971	-1,90
C20orf106	Homo sapiens chromosome 20 open reading frame 106 (C20orf106), mRNA [NM_001012971]	6,648	8,545	-1,90
TSPO	Homo sapiens translocator protein (18kDa) (TSPO), transcript variant PBR, mRNA [NM_000714]	9,804	11,706	-1,90
APBB1IP	Homo sapiens amyloid beta (A4) precursor protein-binding, family B, member 1 interacting protein (APBB1IP), mRNA [NM_019043]	8,897	10,8	-1,90
C1orf135	Homo sapiens chromosome 1 open reading frame 135 (C1orf135), mRNA [NM_024037]	5,72	7,628	-1,91
AL566187	AL566187 Homo sapiens FETAL BRAIN Homo sapiens cDNA clone CS0DF022YK06 3-PRIME, mRNA sequence [AL566187]	8,159	10,07	-1,91
FGD3	Homo sapiens FYVE, RhoGEF and PH domain containing 3 (FGD3), mRNA [NM_033086]	5,022	6,934	-1,91
POLQ	Homo sapiens polymerase (DNA directed), theta (POLQ), mRNA [NM_199420]	6,683	8,595	-1,91
THC2560357	chr8:26570651-26570592	8,723	10,635	-1,91
ZBTB7B	Homo sapiens zinc finger and BTB domain containing 7B (ZBTB7B), mRNA [NM_015872]	5,942	7,855	-1,91
AF131777	Homo sapiens clone 25061 mRNA sequence. [AF131777]	5,141	7,056	-1,92
GRIN2D	Homo sapiens glutamate receptor, ionotropic, N-methyl D-aspartate 2D (GRIN2D), mRNA [NM_000836]	12,036	13,952	-1,92
-	chrX:149912851-149912991	9,467	11,384	-1,92
SERPINB1	Homo sapiens serpin peptidase inhibitor, clade B (ovalbumin), member 1 (SERPINB1), mRNA [NM_030666]	9,98	11,9	-1,92
TIA1	Homo sapiens TIA1 cytotoxic granule-associated RNA binding protein (TIA1), transcript variant 1, mRNA [NM_022037]	8,195	10,115	-1,92
SGOL1	Homo sapiens shugoshin-like 1 (S. pombe) (SGOL1), transcript variant A1, mRNA [NM_001012409]	5,482	7,406	-1,92
CCDC109B	Homo sapiens coiled-coil domain containing 109B (CCDC109B), mRNA [NM_017918]	7,787	9,712	-1,93
MIDN	Homo sapiens midnolin (MIDN), mRNA [NM_177401]	4,529	6,459	-1,93
ORC6L	Homo sapiens origin recognition complex, subunit 6 like (yeast) (ORC6L), mRNA [NM_014321]	7,564	9,494	-1,93
THC2646626	chr2:225091016-225090957	4,845	6,783	-1,94
LOC440731	PREDICTED: Homo sapiens hypothetical LOC440731, transcript variant 2 (LOC440731), mRNA [XM_933693]	5,83	7,772	-1,94
AU184995	AU184995 AU184995 Homo sapiens T-cell library (Sugita Y) Homo sapiens cDNA clone B00751-019, mRNA sequence [AU184995]	6,244	8,189	-1,95
C1orf55	Homo sapiens chromosome 1 open reading frame 55 (C1orf55), mRNA [NM_152608]	6,855	8,802	-1,95
FAM8A1	Homo sapiens family with sequence similarity 8, member A1 (FAM8A1), mRNA [NM_016255]	5,031	6,979	-1,95
BHLHB4	Homo sapiens basic helix-loop-helix domain containing, class B, 4 (BHLHB4), mRNA [NM_080606]	9,639	11,594	-1,96
-	chr1:120341166-120340857	9,82	11,777	-1,96
BC030992	Homo sapiens cDNA clone IMAGE:4273559, partial cds. [BC030992]	5,733	7,69	-1,96
CR617865	full-length cDNA clone CS0DF021YD16 of Fetal brain of Homo sapiens (human). [CR617865]	7,184	9,143	-1,96

Supplemental Table 1
Morandi et al

COX6A2	Homo sapiens cytochrome c oxidase subunit VIa polypeptide 2 (COX6A2), nuclear gene encoding mitochondrial protein, mRNA [NM_005205]	13,612	15,576	-1,96
GOPC	Homo sapiens golgi associated PDZ and coiled-coil motif containing (GOPC), transcript variant 1, mRNA [NM_020399]	6,923	8,89	-1,97
HIST1H2BO	Homo sapiens histone cluster 1, H2bo (HIST1H2BO), mRNA [NM_003527]	9,726	11,693	-1,97
TSPAN10	Homo sapiens tetraspanin 10 (TSPAN10), mRNA [NM_031945]	11,943	13,91	-1,97
-	chr19:047057048-047056751	8,918	10,888	-1,97
ACSS2	Homo sapiens acyl-CoA synthetase short-chain family member 2 (ACSS2), transcript variant 1, mRNA [NM_018677]	6,696	8,666	-1,97
MNAB	Homo sapiens mRNA; cDNA DKFZp667B165 (from clone DKFZp667B165). [AL833177]	5,385	7,355	-1,97
CENTB1	Homo sapiens centaurin, beta 1 (CENTB1), mRNA [NM_014716]	5,056	7,029	-1,97
C12orf48	Homo sapiens chromosome 12 open reading frame 48 (C12orf48), mRNA [NM_017915]	7,67	9,644	-1,97
SPCS3	Homo sapiens signal peptidase complex subunit 3 homolog (S. cerevisiae) (SPCS3), mRNA [NM_021928]	10,219	12,195	-1,98
CD79A	Homo sapiens CD79a molecule, immunoglobulin-associated alpha (CD79A), transcript variant 1, mRNA [NM_001783]	9,021	10,998	-1,98
JMY	Homo sapiens junction-mediating and regulatory protein (JMY), mRNA [NM_152405]	6,299	8,278	-1,98
AV705991	AV705991 ADB Homo sapiens cDNA clone ADBCFC01 5', mRNA sequence [AV705991]	9,09	11,069	-1,98
BC020539	Homo sapiens, clone IMAGE:3048919, mRNA, partial cds. [BC020539]	8,643	10,623	-1,98
E2F7	Homo sapiens E2F transcription factor 7 (E2F7), mRNA [NM_203394]	7,012	8,992	-1,98
THC2591397	1617101C ribosomal protein S4. {Rattus norvegicus} (exp=-1; wgp=0; cg=0), partial (29%) [THC2591397]	9,2	11,184	-1,98
CDC20	Homo sapiens cell division cycle 20 homolog (S. cerevisiae) (CDC20), mRNA [NM_001255]	7,861	9,845	-1,98
LOC645592	PREDICTED: Homo sapiens similar to peptidylprolyl isomerase A isoform 1 (LOC645592), mRNA [XR_019387]	7,146	9,133	-1,99
RAD54L	Homo sapiens RAD54-like (S. cerevisiae) (RAD54L), mRNA [NM_003579]	8,285	10,273	-1,99
RECK	Homo sapiens cDNA clone MGC:71628 IMAGE:30336414, complete cds. [BC060806]	6,988	8,977	-1,99
ADAM9	Homo sapiens ADAM metallopeptidase domain 9 (meltrin gamma) (ADAM9), transcript variant 2, mRNA [NM_001005845]	6,391	8,381	-1,99
IFITM5	Homo sapiens interferon induced transmembrane protein 5 (IFITM5), mRNA [NM_001025295]	13,132	15,126	-1,99
-	chr1:243092211-243092270	6,474	8,473	-2,00
SPBC25	Homo sapiens spindle pole body component 25 homolog (S. cerevisiae) (SPBC25), mRNA [NM_020675]	7,621	9,622	-2,00
ATP13A3	Probable cation-transporting ATPase 13A3 (EC 3.6.3.-) (ATPase family homolog up-regulated in senescence cells 1). [Source:Uniprot/SWISSPROT;Acc:Q9H7F0] [ENST00000256031]	5,533	7,537	-2,00
C10orf54	Homo sapiens chromosome 10 open reading frame 54 (C10orf54), mRNA [NM_022153]	9,42	11,424	-2,00
HIST1H2BC	Homo sapiens histone cluster 1, H2bc (HIST1H2BC), mRNA [NM_003526]	6,559	8,565	-2,01
ASF1B	Homo sapiens ASF1 anti-silencing function 1 homolog B (S. cerevisiae) (ASF1B), mRNA [NM_018154]	7,361	9,368	-2,01
AK090405	Homo sapiens mRNA for FLJ00291 protein. [AK090405]	5,437	7,446	-2,01
THC2690931	AF235005 suppression of tumorigenicity 16 protein {Homo sapiens} (exp=-1; wgp=0; cg=0), partial (13%) [THC2690931]	4,973	6,989	-2,02
BX350256	BX350256 BX350256 Homo sapiens PLACENTA COT 25-NORMALIZED Homo sapiens cDNA clone CS0DI081YM18 3-PRIME, mRNA sequence [BX350256]	6,137	8,154	-2,02
-	chr5:151164351-151164292	8,029	10,048	-2,02
TNFRSF19L	Homo sapiens tumor necrosis factor receptor superfamily, member 19-like (TNFRSF19L), transcript variant 1, mRNA [NM_032871]	8,893	10,912	-2,02

Supplemental Table 1
Morandi et al

BF858887	BF858887 RC5-FT0194-071200-023-E10 FT0194 Homo sapiens cDNA, mRNA sequence [BF858887]	4,152	6,172	-2,02
CEP55	Homo sapiens centrosomal protein 55kDa (CEP55), mRNA [NM_018131]	7,71	9,733	-2,02
USP3	Homo sapiens ubiquitin specific peptidase 3 (USP3), mRNA [NM_006537]	8,303	10,327	-2,02
-	chr11:064962002-064961943	4,813	6,841	-2,03
AI216457	qh07g09.x1 Soares_NFL_T_GBC_S1 Homo sapiens cDNA clone IMAGE:1844032 3' similar to SW:RL39_HUMAN P02404 60S RIBOSOMAL PROTEIN L39. [2] SW:RL39_HUMAN ;, mRNA sequence [AI216457]	6,213	8,241	-2,03
HIST1H2BB	Homo sapiens histone cluster 1, H2bb (HIST1H2BB), mRNA [NM_021062]	8,245	10,274	-2,03
MLSTD2	Homo sapiens male sterility domain containing 2 (MLSTD2), mRNA [NM_032228]	5,967	8	-2,03
-	chr2:204345675-204345616	5,694	7,735	-2,04
PHF19	Homo sapiens PHD finger protein 19 (PHF19), transcript variant 2, mRNA [NM_001009936]	9,63	11,672	-2,04
LOC728688	PREDICTED: Homo sapiens hypothetical protein LOC728688 (LOC728688), mRNA [XM_001130587]	5,544	7,589	-2,05
CDT1	Homo sapiens chromatin licensing and DNA replication factor 1 (CDT1), mRNA [NM_030928]	10,729	12,775	-2,05
GINS2	Homo sapiens GINS complex subunit 2 (Psf2 homolog) (GINS2), mRNA [NM_016095]	9,363	11,414	-2,05
HDAC4	Homo sapiens histone deacetylase 4 (HDAC4), mRNA [NM_006037]	6,115	8,167	-2,05
ENST00000328474	Homo sapiens PRO1843 mRNA, complete cds. [AF119854]	7,873	9,93	-2,06
HIST1H2BH	Homo sapiens histone cluster 1, H2bh (HIST1H2BH), mRNA [NM_003524]	9,934	11,994	-2,06
SNHG1	Homo sapiens small nucleolar RNA host gene (non-protein coding) 1 (SNHG1) on chromosome 11 [NR_003098]	11,653	13,713	-2,06
BX409884	BX409884 Homo sapiens NEUROBLASTOMA Homo sapiens cDNA clone CL0BB005ZC10 5-PRIME, mRNA sequence [BX409884]	5,673	7,741	-2,07
DKFZp762E1312	Homo sapiens hypothetical protein DKFZp762E1312 (DKFZp762E1312), mRNA [NM_018410]	6,515	8,585	-2,07
GABPB2	Homo sapiens GA binding protein transcription factor, beta subunit 2 (GABPB2), transcript variant gamma-1, mRNA [NM_002041]	7,485	9,554	-2,07
AV722457	AV722457 HTB Homo sapiens cDNA clone HTBAMH03 5', mRNA sequence [AV722457]	5,472	7,543	-2,07
WNK1	Homo sapiens WNK lysine deficient protein kinase 1, mRNA (cDNA clone IMAGE:4650586), complete cds. [BC094862]	4,961	7,032	-2,07
CR749652	Homo sapiens mRNA; cDNA DKFZp686I07120 (from clone DKFZp686I07120). [CR749652]	8,851	10,924	-2,07
TMC8	Homo sapiens mRNA for FLJ00400 protein. [AK090478]	8,195	10,271	-2,08
BC009353	Homo sapiens immediate early response 2, mRNA (cDNA clone IMAGE:4297793), **** WARNING: chimeric clone ****. [BC009353]	4,938	7,016	-2,08
ENST00000322831	Zinc finger CCHC domain-containing protein 7. [Source:Uniprot/SWISSPROT;Acc:Q8N3Z6] [ENST00000322831]	4,367	6,448	-2,08
-	chr17:025067488-025067427	7,596	9,679	-2,08
DHFR	Homo sapiens dihydrofolate reductase (DHFR), mRNA [NM_000791]	6,237	8,325	-2,09
PTP4A1	Homo sapiens protein tyrosine phosphatase type IVA, member 1 (PTP4A1), mRNA [NM_003463]	6,308	8,397	-2,09
PTTG2	Homo sapiens pituitary tumor-transforming 2 (PTTG2), mRNA [NM_006607]	8,964	11,054	-2,09
NUB1	full-length cDNA clone CS0DJ007YF12 of T cells (Jurkat cell line) Cot 10-normalized of Homo sapiens (human). [CR606629]	5,801	7,892	-2,09
GALR3	Homo sapiens galanin receptor 3 (GALR3), mRNA [NM_003614]	12,774	14,866	-2,09
TRIB1	Homo sapiens tribbles homolog 1 (Drosophila) (TRIB1), mRNA [NM_025195]	5,775	7,869	-2,09
-	chr4:078089427-078089486	9,741	11,835	-2,09
TOB1	Homo sapiens, clone IMAGE:4133978, mRNA. [BC015064]	7,171	9,267	-2,10

Supplemental Table 1
Morandi et al

EMP3	Homo sapiens epithelial membrane protein 3 (EMP3), mRNA [NM_001425]	9,42	11,518	-2,10
AA593970	AA593970 nn01c05.s1 NCI_CGAP_Co9 Homo sapiens cDNA clone IMAGE:1076456 3', mRNA sequence [AA593970]	8,829	10,935	-2,11
CKS2	Homo sapiens CDC28 protein kinase regulatory subunit 2 (CKS2), mRNA [NM_001827]	10,064	12,17	-2,11
RAP2C	Homo sapiens RAP2C, member of RAS oncogene family (RAP2C), mRNA [NM_021183]	5,216	7,322	-2,11
CENPE	Homo sapiens centromere protein E, 312kDa (CENPE), mRNA [NM_001813]	7,55	9,658	-2,11
SNORD22	Homo sapiens small nucleolar RNA, C/D box 22 (SNORD22) on chromosome 11 [NR_000008]	6,696	8,808	-2,11
LOC137107	PREDICTED: Homo sapiens similar to ribosomal protein L10a (LOC137107), mRNA [XM_070233]	6,443	8,558	-2,12
BG950086	BG950086 CM1-CT0377-020101-710-g05 CT0377 Homo sapiens cDNA, mRNA sequence [BG950086]	5,898	8,017	-2,12
HIST1H2BG	Homo sapiens histone cluster 1, H2bg (HIST1H2BG), mRNA [NM_003518]	7,038	9,158	-2,12
VCPIP1	Homo sapiens valosin containing protein (p97)/p47 complex interacting protein 1 (VCPIP1), mRNA [NM_025054]	6,165	8,286	-2,12
FAM72A	Homo sapiens family with sequence similarity 72, member A (FAM72A), mRNA [NM_207418]	6,658	8,779	-2,12
THC2726281	P97868_MOUSE (P97868) Proliferation potential-related protein, partial (4%) [THC2726281]	8,266	10,388	-2,12
HIST3H2BB	DB010344 TCOLN2 Homo sapiens cDNA clone TCOLN2001340 5', mRNA sequence [DB010344]	9,025	11,148	-2,12
-	chr19:050721774-050721715	6,1	8,228	-2,13
AF275804	Homo sapiens PNAS-108 mRNA, partial sequence. [AF275804]	9,348	11,475	-2,13
TNFRSF10C	Homo sapiens tumor necrosis factor receptor superfamily, member 10c, decoy without an intracellular domain (TNFRSF10C), mRNA [NM_003841]	6,397	8,526	-2,13
ADM	Homo sapiens adrenomedullin (ADM), mRNA [NM_001124]	11,864	13,994	-2,13
KLHL7	Homo sapiens kelch-like 7 (Drosophila), mRNA (cDNA clone IMAGE:3899090), complete cds. [BC009555]	7,399	9,539	-2,14
SERP1	Homo sapiens stress-associated endoplasmic reticulum protein 1 (SERP1), mRNA [NM_014445]	5,736	7,877	-2,14
THC2520867	ALU1_HUMAN (P39188) Alu subfamily J sequence contamination warning entry, partial (6%) [THC2520867]	6,017	8,161	-2,14
RP5-1022P6.2	Homo sapiens hypothetical protein KIAA1434 (KIAA1434), mRNA [NM_019593]	9,832	11,981	-2,15
-	chr22:018092797-018092738	12,16	14,317	-2,16
TPX2	Homo sapiens TPX2, microtubule-associated, homolog (Xenopus laevis) (TPX2), mRNA [NM_012112]	8,701	10,857	-2,16
GTSE1	Homo sapiens G-2 and S-phase expressed 1 (GTSE1), mRNA [NM_016426]	7,538	9,707	-2,17
BC028232	Homo sapiens, clone IMAGE:5221276, mRNA, partial cds. [BC028232]	11,167	13,339	-2,17
SPAG5	Homo sapiens sperm associated antigen 5 (SPAG5), mRNA [NM_006461]	10,619	12,791	-2,17
ZFAND5	Homo sapiens zinc finger, AN1-type domain 5 (ZFAND5), mRNA [NM_006007]	10,749	12,926	-2,18
CENTG3	Homo sapiens centaurin, gamma 3 (CENTG3), transcript variant 2, mRNA [NM_001042535]	11,731	13,909	-2,18
SERTAD2	Homo sapiens SERTA domain containing 2 (SERTAD2), mRNA [NM_014755]	4,637	6,815	-2,18
C6orf173	Homo sapiens chromosome 6 open reading frame 173 (C6orf173), mRNA [NM_001012507]	9,571	11,753	-2,18
PTPLAD2	Homo sapiens protein tyrosine phosphatase-like A domain containing 2 (PTPLAD2), mRNA [NM_001010915]	5,292	7,473	-2,18
DEPDC1	Homo sapiens DEP domain containing 1 (DEPDC1), mRNA [NM_017779]	7,774	9,96	-2,19
RP2	Homo sapiens retinitis pigmentosa 2 (X-linked recessive) (RP2), mRNA [NM_006915]	4,819	7,012	-2,19
C13orf3	Homo sapiens chromosome 13 open reading frame 3, mRNA (cDNA clone MGC:4832 IMAGE:3604003), complete cds. [BC013418]	7,947	10,149	-2,20

TTK	Homo sapiens TTK protein kinase (TTK), mRNA [NM_003318]	6,673	8,877	-2,20
FANCD2	Homo sapiens Fanconi anemia, complementation group D2 (FANCD2), transcript variant 2, mRNA [NM_001018115]	7,851	10,057	-2,21
HIST1H2BD	AGENCOURT_8209273 NIH_MGC_112 Homo sapiens cDNA clone IMAGE:6265521 5', mRNA sequence [BQ683489]	9,086	11,291	-2,21
KIAA0179	Homo sapiens KIAA0179 (KIAA0179), mRNA [NM_015056]	6,803	9,015	-2,21
THC2689175	AB002334 Start codon is not identified {Homo sapiens} (exp=-1; wgp=0; cg=0), partial (9%) [THC2689175]	6,311	8,524	-2,21
RAB27A	Homo sapiens RAB27A, member RAS oncogene family (RAB27A), transcript variant 1, mRNA [NM_004580]	6,351	8,57	-2,22
C21orf96	Homo sapiens cDNA: FLJ20856 fis, clone ADKA01509. [AK024509]	5,409	7,63	-2,22
ARHGAP11A	Homo sapiens Rho GTPase activating protein 11A (ARHGAP11A), transcript variant 1, mRNA [NM_014783]	6,052	8,275	-2,22
AURKB	Homo sapiens aurora kinase B (AURKB), mRNA [NM_004217]	6,143	8,369	-2,23
BE138567	xr77d10.x2 NCI_CGAP_Ov26 Homo sapiens cDNA clone IMAGE:2766163 3', mRNA sequence [BE138567]	6,671	8,903	-2,23
PKMYT1	Homo sapiens protein kinase, membrane associated tyrosine/threonine 1 (PKMYT1), transcript variant 2, mRNA [NM_182687]	10,314	12,547	-2,23
IRAK3	Homo sapiens interleukin-1 receptor-associated kinase 3 (IRAK3), mRNA [NM_007199]	5,958	8,193	-2,24
NCF2	Homo sapiens neutrophil cytosolic factor 2 (65kDa, chronic granulomatous disease, autosomal 2) (NCF2), mRNA [NM_000433]	8,921	11,16	-2,24
SKP2	Homo sapiens S-phase kinase-associated protein 2 (p45) (SKP2), transcript variant 2, mRNA [NM_032637]	6,381	8,622	-2,24
PPIG	Homo sapiens peptidylprolyl isomerase G (cyclophilin G), mRNA (cDNA clone IMAGE:3461499), complete cds. [BC001555]	5,597	7,844	-2,25
AL832717	Homo sapiens mRNA; cDNA DKFZp313B039 (from clone DKFZp313B039). [AL832717]	7,189	9,439	-2,25
TRIM35	Homo sapiens tripartite motif-containing 35 (TRIM35), transcript variant 2, mRNA [NM_171982]	7,564	9,813	-2,25
HIST2H2AC	Homo sapiens histone cluster 2, H2ac (HIST2H2AC), mRNA [NM_003517]	10,876	13,133	-2,26
FAM122B	Homo sapiens family with sequence similarity 122B (FAM122B), mRNA [NM_145284]	6,244	8,503	-2,26
FEM1B	Homo sapiens fem-1 homolog b (C. elegans) (FEM1B), mRNA [NM_015322]	6,744	9,005	-2,26
IRX3	Homo sapiens iroquois homeobox protein 3 (IRX3), mRNA [NM_024336]	9,042	11,304	-2,26
LOC642123	Homo sapiens cDNA FLJ46881 fis, clone UTERU3015647, moderately similar to Embigin precursor. [AK128714]	8,113	10,38	-2,27
SLPI	Homo sapiens secretory leukocyte peptidase inhibitor (SLPI), mRNA [NM_003064]	6,742	9,013	-2,27
HIST1H3D	Homo sapiens histone cluster 1, H3d (HIST1H3D), mRNA [NM_003530]	6,511	8,791	-2,28
CDC2	Homo sapiens cell division cycle 2, G1 to S and G2 to M (CDC2), transcript variant 1, mRNA [NM_001786]	8,232	10,515	-2,28
ATP11B	Homo sapiens ATPase, Class VI, type 11B (ATP11B), mRNA [NM_014616]	5,853	8,136	-2,28
LOC731997	PREDICTED: Homo sapiens hypothetical protein LOC731997 (LOC731997), mRNA [XM_001131542]	8,813	11,099	-2,29
SUSD3	Homo sapiens sushi domain containing 3 (SUSD3), mRNA [NM_145006]	8,887	11,176	-2,29
KIF2C	Homo sapiens kinesin family member 2C (KIF2C), mRNA [NM_006845]	9,674	11,964	-2,29
ROPN1L	Homo sapiens ropporin 1-like (ROPN1L), mRNA [NM_031916]	5,45	7,74	-2,29
TLE3	Homo sapiens transducin-like enhancer of split 3 (E(sp1) homolog, Drosophila) (TLE3), mRNA [NM_005078]	7,257	9,557	-2,30
-	chr6:086853121-086853059	7,913	10,215	-2,30
DLG7	Homo sapiens discs, large homolog 7 (Drosophila) (DLG7), mRNA [NM_014750]	7,712	10,017	-2,31
BX641010	Homo sapiens mRNA; cDNA DKFZp686G01227 (from clone DKFZp686G01227). [BX641010]	6,591	8,904	-2,31

Supplemental Table 1
Morandi et al

HIST1H1D	Homo sapiens histone cluster 1, H1d (HIST1H1D), mRNA [NM_005320]	8,7	11,017	-2,32
PYGL	Homo sapiens phosphorylase, glycogen; liver (Hers disease, glycogen storage disease type VI) (PYGL), mRNA [NM_002863]	8,456	10,774	-2,32
LRRC25	Homo sapiens leucine rich repeat containing 25 (LRRC25), mRNA [NM_145256]	7,527	9,845	-2,32
BTBD14A	Homo sapiens BTB (POZ) domain containing 14A (BTBD14A), mRNA [NM_144653]	7,188	9,507	-2,32
PTGS2	Homo sapiens prostaglandin-endoperoxide synthase 2 (prostaglandin G/H synthase and cyclooxygenase) (PTGS2), mRNA [NM_000963]	5,367	7,688	-2,32
AK092271	Homo sapiens cDNA FLJ34952 fis, clone NTONG2000531. [AK092271]	11,043	13,367	-2,32
ABL1	Homo sapiens v-abl Abelson murine leukemia viral oncogene homolog 1 (ABL1), transcript variant b, mRNA [NM_007313]	4,651	6,977	-2,33
ESPL1	Homo sapiens extra spindle pole bodies homolog 1 (S. cerevisiae) (ESPL1), mRNA [NM_012291]	8,649	10,979	-2,33
THC2577566	chr8:030060416-030060475	10,103	12,438	-2,34
RAC2	Homo sapiens ras-related C3 botulinum toxin substrate 2 (rho family, small GTP binding protein Rac2) (RAC2), mRNA [NM_002872]	10,004	12,339	-2,34
SYTL1	Homo sapiens synaptotagmin-like 1 (SYTL1), mRNA [NM_032872]	7,117	9,454	-2,34
AK092681	Homo sapiens cDNA FLJ35362 fis, clone SKMUS2000330. [AK092681]	4,778	7,117	-2,34
CEBPE	Homo sapiens CCAAT/enhancer binding protein (C/EBP), epsilon (CEBPE), mRNA [NM_001805]	7,115	9,454	-2,34
HIST1H2AM	Homo sapiens histone cluster 1, H2am (HIST1H2AM), mRNA [NM_003514]	5,842	8,182	-2,34
AMICA1	Homo sapiens adhesion molecule, interacts with CXADR antigen 1 (AMICA1), mRNA [NM_153206]	6,318	8,659	-2,34
FOXM1	Homo sapiens forkhead box M1 (FOXM1), transcript variant 1, mRNA [NM_202002]	8,397	10,739	-2,34
-	chr19:038524297-038524356	6,934	9,28	-2,35
HIST1H2BJ	Homo sapiens histone cluster 1, H2bj (HIST1H2BJ), mRNA [NM_021058]	6,991	9,342	-2,35
ELF4	Homo sapiens E74-like factor 4 (ets domain transcription factor) (ELF4), mRNA [NM_001421]	9,075	11,426	-2,35
CHEK1	Homo sapiens CHK1 checkpoint homolog (S. pombe) (CHEK1), mRNA [NM_001274]	7,479	9,833	-2,35
AW797858	AW797858 CM0-UM0042-130300-280-d11 UM0042 Homo sapiens cDNA, mRNA sequence [AW797858]	6,585	8,947	-2,36
HIST1H1E	Homo sapiens histone cluster 1, H1e (HIST1H1E), mRNA [NM_005321]	8,444	10,808	-2,36
NUAK2	Homo sapiens NUAK family, SNF1-like kinase, 2 (NUAK2), mRNA [NM_030952]	6,927	9,29	-2,36
HMMR	Homo sapiens hyaluronan-mediated motility receptor (RHAMM) (HMMR), transcript variant 1, mRNA [NM_012484]	8,292	10,66	-2,37
NCF4	Homo sapiens neutrophil cytosolic factor 4, 40kDa (NCF4), transcript variant 1, mRNA [NM_000631]	6,049	8,417	-2,37
PBK	Homo sapiens PDZ binding kinase (PBK), mRNA [NM_018492]	8,904	11,279	-2,38
NFKBIZ	Homo sapiens nuclear factor of kappa light polypeptide gene enhancer in B-cells inhibitor, zeta (NFKBIZ), transcript variant 1, mRNA [NM_031419]	7,69	10,067	-2,38
NUF2	Homo sapiens NUF2, NDC80 kinetochore complex component, homolog (S. cerevisiae) (NUF2), transcript variant 1, mRNA [NM_145697]	9,175	11,552	-2,38
UTS2R	Homo sapiens urotensin 2 receptor (UTS2R), mRNA [NM_018949]	12,163	14,545	-2,38
ZCCHC6	Homo sapiens zinc finger, CCHC domain containing 6 (ZCCHC6), mRNA [NM_024617]	4,72	7,104	-2,38
FCRLB	Homo sapiens Fc receptor-like B, mRNA (cDNA clone MGC:71141 IMAGE:3529386), complete cds. [BC067080]	11,848	14,236	-2,39
BUB1	Homo sapiens BUB1 budding uninhibited by benzimidazoles 1 homolog (yeast) (BUB1), mRNA [NM_004336]	6,93	9,329	-2,40

Supplemental Table 1
Morandi et al

CEACAM4	Homo sapiens carcinoembryonic antigen-related cell adhesion molecule 4 (CEACAM4), mRNA [NM_001817]	5,69	8,098	-2,41
CEBPB	Homo sapiens CCAAT/enhancer binding protein (C/EBP), beta (CEBPB), mRNA [NM_005194]	12,305	14,714	-2,41
MXD1	Homo sapiens MAX dimerization protein 1 (MXD1), mRNA [NM_002357]	7,503	9,917	-2,41
AK092421	Homo sapiens cDNA FLJ35102 fis, clone PLACE6006474, weakly similar to ADHESIVE PLAQUE MATRIX PROTEIN PRECURSOR. [AK092421]	8,808	11,224	-2,42
SERPINA1	Homo sapiens serpin peptidase inhibitor, clade A (alpha-1 antiproteinase, antitrypsin), member 1 (SERPINA1), transcript variant 2, mRNA [NM_001002236]	7,482	9,904	-2,42
AI381562	AI381562 te76g06.x1 Soares_NFL_T_GBC_S1 Homo sapiens cDNA clone IMAGE:2092666 3', mRNA sequence [AI381562]	4,658	7,083	-2,43
TYMS	Homo sapiens thymidylate synthetase (TYMS), mRNA [NM_001071]	10,467	12,897	-2,43
RRM2	Homo sapiens ribonucleotide reductase M2 polypeptide (RRM2), mRNA [NM_001034]	6,426	8,862	-2,44
FNDC3B	Homo sapiens fibronectin type III domain containing 3B, mRNA (cDNA clone IMAGE:3882800), complete cds. [BC012204]	4,683	7,126	-2,44
LRRK2	Homo sapiens leucine-rich repeat kinase 2 (LRRK2), mRNA [NM_198578]	5,77	8,212	-2,44
THC2559929	Q9UP60_HUMAN (Q9UP60) SNC73 protein, partial (54%) [THC2559929]	8,144	10,591	-2,45
NCAPH	Homo sapiens non-SMC condensin I complex, subunit H (NCAPH), mRNA [NM_015341]	7,868	10,317	-2,45
PDE4D	Homo sapiens phosphodiesterase 4D, cAMP-specific (phosphodiesterase E3 dunce homolog, Drosophila) (PDE4D), mRNA [NM_006203]	4,777	7,226	-2,45
-	chr17:037971521-037971466	5,266	7,715	-2,45
BM547196	BM547196 AGENCOURT_6499364 NIH_MGC_124 Homo sapiens cDNA clone IMAGE:5730270 5', mRNA sequence [BM547196]	10,661	13,11	-2,45
THC2723346	Q5VZL5_HUMAN (Q5VZL5) Zinc finger protein 262, complete [THC2723346]	10,549	13,004	-2,46
KIAA1545	Homo sapiens XTP9 (XTP9) mRNA, complete cds. [AF490258]	9,111	11,569	-2,46
NPAS3	Homo sapiens neuronal PAS domain protein 3 (NPAS3), transcript variant 2, mRNA [NM_173159]	11,903	14,367	-2,46
BUB1B	Homo sapiens BUB1 budding uninhibited by benzimidazoles 1 homolog beta (yeast) (BUB1B), mRNA [NM_001211]	7,468	9,939	-2,47
E2F1	Homo sapiens E2F transcription factor 1 (E2F1), mRNA [NM_005225]	8,066	10,538	-2,47
XRCC6BP1	Homo sapiens XRCC6 binding protein 1, mRNA (cDNA clone IMAGE:5742519), complete cds. [BC033881]	4,92	7,395	-2,48
KLF6	Homo sapiens Kruppel-like factor 6 (KLF6), transcript variant 1, mRNA [NM_001008490]	6,1	8,577	-2,48
OTUD1	Homo sapiens mRNA, clone: TH020D07. [AB188491]	5,91	8,386	-2,48
IRX5	Homo sapiens iroquois homeobox protein 5 (IRX5), mRNA [NM_005853]	5,688	8,166	-2,48
BF089603	BF089603 CM2-HT0946-140900-364-f11 HT0946 Homo sapiens cDNA, mRNA sequence [BF089603]	6,119	8,601	-2,48
BM979049	BM979049 UI-CF-DU1-adl-i-11-0-UI.s1 UI-CF-DU1 Homo sapiens cDNA clone UI-CF-DU1-adl-i-11-0-UI 3', mRNA sequence [BM979049]	6,37	8,853	-2,48
SNX5	Homo sapiens sorting nexin 5, mRNA (cDNA clone IMAGE:3629947), complete cds. [BC002724]	6,68	9,164	-2,48
IKZF1	Homo sapiens PRO0758 mRNA, complete cds. [AF116605]	5,802	8,287	-2,49
KIF15	Homo sapiens kinesin family member 15 (KIF15), mRNA [NM_020242]	7,564	10,049	-2,49
DKFZP434I0714	PREDICTED: Homo sapiens hypothetical protein DKFZP434I0714 (DKFZP434I0714), mRNA [XM_929673]	9,888	12,376	-2,49
NUSAP1	Homo sapiens nucleolar and spindle associated protein 1 (NUSAP1), transcript variant 1, mRNA [NM_016359]	6,84	9,328	-2,49
THC2707492	chr3:13430459-13430400	6,092	8,581	-2,49
CENPN	Homo sapiens centromere protein N (CENPN), mRNA [NM_018455]	9,301	11,794	-2,49

KIF4A	Homo sapiens kinesin family member 4A (KIF4A), mRNA [NM_012310]	6,826	9,322	-2,50
MAD2L1	Homo sapiens MAD2 mitotic arrest deficient-like 1 (yeast) (MAD2L1), mRNA [NM_002358]	8,838	11,334	-2,50
LOC401975	PREDICTED: Homo sapiens similar to ribosomal protein S3a (LOC401975), mRNA [XR_017247]	5,895	8,393	-2,50
THC2670384	chr13:24775303-24775362	8,891	11,388	-2,50
BANK1	Homo sapiens B-cell scaffold protein with ankyrin repeats 1 (BANK1), mRNA [NM_017935]	5,389	7,886	-2,50
SOD2	Homo sapiens superoxide dismutase 2, mitochondrial (SOD2), nuclear gene encoding mitochondrial protein, transcript variant 2, mRNA [NM_001024465]	10,326	12,825	-2,50
Y10152	H.sapiens mRNA for CRF2 receptor, beta isoform, aberrantly spliced, (94bp deletion). [Y10152]	9,458	11,963	-2,51
ARHGAP15	Homo sapiens Rho GTPase activating protein 15 (ARHGAP15), mRNA [NM_018460]	5,096	7,605	-2,51
KIF11	Homo sapiens kinesin family member 11 (KIF11), mRNA [NM_004523]	5,864	8,372	-2,51
OIP5	Homo sapiens Opa interacting protein 5 (OIP5), mRNA [NM_007280]	8,258	10,768	-2,51
HIST2H2AB	Homo sapiens histone cluster 2, H2ab (HIST2H2AB), mRNA [NM_175065]	8,88	11,392	-2,51
THC2620175	chr14:72673264-72673205	11,888	14,407	-2,52
HK2	Homo sapiens hexokinase 2 (HK2), mRNA [NM_000189]	5,499	8,028	-2,53
HIST1H1C	Homo sapiens histone cluster 1, H1c (HIST1H1C), mRNA [NM_005319]	11,015	13,546	-2,53
EMR1	Homo sapiens egf-like module containing, mucin-like, hormone receptor-like 1 (EMR1), mRNA [NM_001974]	5,643	8,175	-2,53
NOD2	Homo sapiens nucleotide-binding oligomerization domain containing 2 (NOD2), mRNA [NM_022162]	5,245	7,78	-2,54
F5	Homo sapiens coagulation factor V (proaccelerin, labile factor) (F5), mRNA [NM_000130]	6,233	8,769	-2,54
RIN3	Homo sapiens cDNA FLJ11700 fis, clone HEMBA1005050. [AK021762]	5,27	7,818	-2,55
ID2	Homo sapiens inhibitor of DNA binding 2, dominant negative helix-loop-helix protein (ID2), mRNA [NM_002166]	9,617	12,168	-2,55
TROAP	Homo sapiens trophinin associated protein (tastin) (TROAP), mRNA [NM_005480]	9,624	12,181	-2,56
UCN2	Homo sapiens urocortin 2 (UCN2), mRNA [NM_033199]	9,67	12,228	-2,56
AI571129	AI571129 tn85e01.x1 NCI_CGAP_Ut2 Homo sapiens cDNA clone IMAGE:2176344 3', mRNA sequence [AI571129]	5,973	8,537	-2,56
C15orf42	Homo sapiens chromosome 15 open reading frame 42 (C15orf42), mRNA [NM_152259]	7,731	10,295	-2,56
STXBP2	Homo sapiens syntaxin binding protein 2 (STXBP2), mRNA [NM_006949]	4,308	6,891	-2,58
TK1	Homo sapiens thymidine kinase 1, soluble (TK1), mRNA [NM_003258]	12,154	14,748	-2,59
HIST2H2AA4	Homo sapiens histone cluster 2, H2aa4 (HIST2H2AA4), mRNA [NM_001040874]	12,075	14,678	-2,60
RAB11FIP1	Homo sapiens RAB11 family interacting protein 1 (class I) (RAB11FIP1), transcript variant 2, mRNA [NM_001002233]	7,011	9,616	-2,61
CEBPA	Homo sapiens CCAAT/enhancer binding protein (C/EBP), alpha (CEBPA), mRNA [NM_004364]	11,308	13,914	-2,61
PBEF1	Homo sapiens pre-B-cell colony enhancing factor 1 (PBEF1), mRNA [NM_005746]	9,351	11,957	-2,61
FAM129C	Homo sapiens family with sequence similarity 129, member C (FAM129C), mRNA [NM_173544]	6,359	8,975	-2,62
KIF20A	Homo sapiens kinesin family member 20A (KIF20A), mRNA [NM_005733]	7,176	9,793	-2,62
G0S2	Homo sapiens G0/G1switch 2 (G0S2), mRNA [NM_015714]	10,211	12,831	-2,62
PTTG1	Homo sapiens pituitary tumor-transforming 1 (PTTG1), mRNA [NM_004219]	11,755	14,375	-2,62
THC2544903	TXD13_HUMAN (Q9H1E5) Thioredoxin domain-containing protein 13 precursor, partial (90%) [THC2544903]	4,846	7,493	-2,65

Supplemental Table 1
Morandi et al

LOC728965	Homo sapiens mRNA; cDNA DKFZp666D074 (from clone DKFZp666D074) [AL833005]	8,878	11,53	-2,65
ALOX5	Homo sapiens arachidonate 5-lipoxygenase (ALOX5), mRNA [NM_000698]	8,775	11,428	-2,65
PRAM1	Homo sapiens PML-RARA regulated adaptor molecule 1 (PRAM1), mRNA [NM_032152]	8,181	10,838	-2,66
HIST1H4A	Homo sapiens histone cluster 1, H4a (HIST1H4A), mRNA [NM_003538]	5,323	7,983	-2,66
ITGAL	Homo sapiens integrin, alpha L (antigen CD11A (p180), lymphocyte function-associated antigen 1; alpha polypeptide) (ITGAL), mRNA [NM_002209]	5,818	8,486	-2,67
CCNA2	Homo sapiens cyclin A2 (CCNA2), mRNA [NM_001237]	6,102	8,772	-2,67
PLEKHK1	Homo sapiens pleckstrin homology domain containing, family K member 1 (PLEKHK1), mRNA [NM_145307]	5,94	8,614	-2,67
SPI1	Homo sapiens spleen focus forming virus (SFFV) proviral integration oncogene spi1 (SPI1), mRNA [NM_003120]	4,65	7,342	-2,69
PSORS1C2	Homo sapiens psoriasis susceptibility 1 candidate 2 (PSORS1C2), mRNA [NM_014069]	8,337	11,033	-2,70
CENPF	Homo sapiens centromere protein F, 350/400ka (mitosin) (CENPF), mRNA [NM_016343]	10,551	13,257	-2,71
BST1	Homo sapiens bone marrow stromal cell antigen 1 (BST1), mRNA [NM_004334]	7,522	10,232	-2,71
UHRF1	Homo sapiens ubiquitin-like, containing PHD and RING finger domains, 1 (UHRF1), transcript variant 2, mRNA [NM_013282]	8,296	11,008	-2,71
NCAPG	Homo sapiens non-SMC condensin I complex, subunit G (NCAPG), mRNA [NM_022346]	8,967	11,69	-2,72
SLC11A1	Homo sapiens solute carrier family 11 (proton-coupled divalent metal ion transporters), member 1 (SLC11A1), mRNA [NM_000578]	5,754	8,477	-2,72
THC2643352	Q2RZ67_SALRD (Q2RZ67) Glycosyl transferase, group 1 family protein , partial (6%) [THC2643352]	4,313	7,035	-2,72
CCNB1	Homo sapiens cyclin B1 (CCNB1), mRNA [NM_031966]	8,951	11,676	-2,73
ANXA8	Homo sapiens annexin A8 (ANXA8), mRNA [NM_001630]	5,202	7,932	-2,73
FLJ22662	Homo sapiens hypothetical protein FLJ22662 (FLJ22662), mRNA [NM_024829]	8,763	11,497	-2,73
PLAC8	Homo sapiens placenta-specific 8 (PLAC8), mRNA [NM_016619]	6,241	8,98	-2,74
AF034187	Homo sapiens clone 2.2H12 Ndr Ser/Thr kinase-like protein mRNA, partial cds. [AF034187]	6,282	9,03	-2,75
CDC45L	Homo sapiens CDC45 cell division cycle 45-like (S. cerevisiae) (CDC45L), mRNA [NM_003504]	9,781	12,537	-2,76
ENST0000035835[illegible]	Runt-related transcription factor 1 (Core-binding factor, alpha 2 subunit) (CBF-alpha 2) (Acute myeloid leukemia 1 protein) (Oncogene AML-1) (Polyomavirus enhancer-binding protein 2 alpha B subunit) (PEBP2-alpha B) (PEA2-alpha B) (SL3-3 enhancer...	5,413	8,185	-2,77
SLC30A1	Homo sapiens solute carrier family 30 (zinc transporter), member 1 (SLC30A1), mRNA [NM_021194]	6,762	9,539	-2,78
HIST1H2AD	Homo sapiens histone cluster 1, H2ad (HIST1H2AD), mRNA [NM_021065]	10,456	13,237	-2,78
ZNF206	Homo sapiens zinc finger protein 206 (ZNF206), mRNA [NM_032805]	9,808	12,595	-2,79
MLF1IP	Homo sapiens MLF1 interacting protein (MLF1IP), mRNA [NM_024629]	8,548	11,336	-2,79
MLC1	Homo sapiens megalencephalic leukoencephalopathy with subcortical cysts 1 (MLC1), transcript variant 1, mRNA [NM_015166]	6,073	8,88	-2,81
KIAA0101	Homo sapiens KIAA0101 (KIAA0101), transcript variant 1, mRNA [NM_014736]	9,288	12,099	-2,81
GSG2	Homo sapiens cDNA FLJ32129 fis, clone PEBLM2000213, weakly similar to Mus musculus genes for integrin aM290, hapsin. [AK056691]	7,256	10,068	-2,81
CDA	Homo sapiens cytidine deaminase (CDA), mRNA [NM_001785]	6,065	8,882	-2,82
THC2539541	MUSTCAYK T-cell receptor alpha chain {Mus musculus} (exp=-1; wgp=0; cg=0), partial (11%) [THC2539541]	4,917	7,734	-2,82

Supplemental Table 1
Morandi et al

TAGAP	Homo sapiens T-cell activation GTPase activating protein (TAGAP), transcript variant 2, mRNA [NM_054114]	6,361	9,182	-2,82
GPR84	Homo sapiens G protein-coupled receptor 84 (GPR84), mRNA [NM_020370]	4,612	7,434	-2,82
PLAUR	Homo sapiens plasminogen activator, urokinase receptor (PLAUR), transcript variant 3, mRNA [NM_001005377]	7,548	10,38	-2,83
LOC392617	PREDICTED: Homo sapiens similar to slit homolog 1 (LOC392617), mRNA [XM_374386]	10,108	12,963	-2,86
ASPM	Homo sapiens asp (abnormal spindle) homolog, microcephaly associated (Drosophila) (ASPM), mRNA [NM_018136]	7,917	10,774	-2,86
CDCA8	Homo sapiens cell division cycle associated 8 (CDCA8), mRNA [NM_018101]	9,679	12,548	-2,87
IGHM	human full-length cDNA clone CS0DD006YL02 of Neuroblastoma of Homo sapiens (human). [BX161420]	8,963	11,832	-2,87
ICAM3	Homo sapiens intercellular adhesion molecule 3 (ICAM3), mRNA [NM_002162]	9,72	12,592	-2,87
FGR	Homo sapiens Gardner-Rasheed feline sarcoma viral (v-fgr) oncogene homolog (FGR), transcript variant 2, mRNA [NM_001042747]	5,237	8,113	-2,88
ENST00000324677	Protein phosphatase Slingshot homolog 2 (EC 3.1.3.48) (EC 3.1.3.16) (SSH-2L) (hSSH-2L). [Source:Uniprot/SWISSPROT;Acc:Q76I76] [ENST00000324677]	5,803	8,689	-2,89
ZC3H12A	Homo sapiens zinc finger CCCH-type containing 12A (ZC3H12A), mRNA [NM_025079]	7,372	10,272	-2,90
TNNT1	Homo sapiens troponin T type 1 (skeletal, slow), mRNA (cDNA clone MGC:104241 IMAGE:4247379), complete cds. [BC107798]	5,374	8,275	-2,90
LOC158830	Homo sapiens similar to Ab2-183 (LOC158830), mRNA [NM_001025265]	5,976	8,88	-2,90
-	chr11:062379527-062379586	3,502	6,407	-2,91
CDC6	Homo sapiens cell division cycle 6 homolog (S. cerevisiae) (CDC6), mRNA [NM_001254]	7,877	10,788	-2,91
PTPRE	Homo sapiens protein tyrosine phosphatase, receptor type, E (PTPRE), transcript variant 1, mRNA [NM_006504]	7,647	10,585	-2,94
AL522024	AL522024 AL522024 Homo sapiens NEUROBLASTOMA COT 10-NORMALIZED Homo sapiens cDNA clone CS0DB007YI07 3-PRIME, mRNA sequence [AL522024]	5,081	8,023	-2,94
C6orf25	G6b protein precursor. [Source:Uniprot/SWISSPROT;Acc:O95866] [ENST00000375806]	5,506	8,453	-2,95
POU2AF1	Homo sapiens POU domain, class 2, associating factor 1 (POU2AF1), mRNA [NM_006235]	6,916	9,863	-2,95
HIST1H2AL	Homo sapiens histone cluster 1, H2al (HIST1H2AL), mRNA [NM_003511]	4,732	7,69	-2,96
CDCA2	Homo sapiens cell division cycle associated 2 (CDCA2), mRNA [NM_152562]	7,686	10,648	-2,96
THC2534530	AF235023 chromosome condensation protein G {Homo sapiens} (exp=0; wgp=1; cg=0), partial (3%) [THC2534530]	6,484	9,477	-2,99
ANPEP	Homo sapiens alanyl (membrane) aminopeptidase (aminopeptidase N, aminopeptidase M, microsomal aminopeptidase, CD13, p150) (ANPEP), mRNA [NM_001150]	6,465	9,502	-3,04
CD69	Homo sapiens CD69 molecule (CD69), mRNA [NM_001781]	7,813	10,887	-3,07
UBE2C	Homo sapiens ubiquitin-conjugating enzyme E2C (UBE2C), transcript variant 6, mRNA [NM_181803]	10,374	13,474	-3,10
PLK1	Homo sapiens polo-like kinase 1 (Drosophila) (PLK1), mRNA [NM_005030]	8,678	11,793	-3,12
MBD2	Homo sapiens methyl-CpG binding domain protein 2 (MBD2), transcript variant testis-specific, mRNA [NM_015832]	5,369	8,487	-3,12
TMEM154	Homo sapiens transmembrane protein 154 (TMEM154), mRNA [NM_152680]	4,801	7,926	-3,13
MYO1G	Homo sapiens mRNA for FLJ00121 protein. [AK074050]	5,648	8,785	-3,14
IL1RN	Homo sapiens interleukin 1 receptor antagonist (IL1RN), transcript variant 1, mRNA [NM_173842]	4,586	7,734	-3,15
ZBTB24	Homo sapiens zinc finger and BTB domain containing 24 (ZBTB24), mRNA [NM_014797]	5,824	8,972	-3,15
LGALS12	Homo sapiens lectin, galactoside-binding, soluble, 12 (galectin 12) (LGALS12), mRNA [NM_033101]	4,813	7,967	-3,15

Supplemental Table 1
Morandi et al

AY358224	Homo sapiens clone DNA213069 RTFV9368 (UNQ9368) mRNA, complete cds. [AY358224]	4,74	7,903	-3,16
BC007606	Homo sapiens cDNA clone IMAGE:3351130, complete cds. [BC007606]	8,007	11,184	-3,18
CXCL1	Homo sapiens chemokine (C-X-C motif) ligand 1 (melanoma growth stimulating activity, alpha) (CXCL1), mRNA [NM_001511]	7,279	10,475	-3,20
PRG1	Homo sapiens proteoglycan 1, secretory granule (PRG1), mRNA [NM_002727]	10,848	14,058	-3,21
CST7	Homo sapiens cystatin F (leukocystatin) (CST7), mRNA [NM_003650]	4,536	7,748	-3,21
FCRL5	Homo sapiens Fc receptor-like 5 (FCRL5), mRNA [NM_031281]	5,296	8,511	-3,22
KIF23	Homo sapiens kinesin family member 23 (KIF23), transcript variant 1, mRNA [NM_138555]	7,965	11,182	-3,22
TREM1	Homo sapiens triggering receptor expressed on myeloid cells 1 (TREM1), mRNA [NM_018643]	4,561	7,778	-3,22
ENST00000251375	Leukocyte immunoglobulin-like receptor subfamily A member 1 precursor (Leukocyte immunoglobulin-like receptor 6) (LIR-6) (CD85i antigen). [Source:Uniprot/SWISSPROT;Acc:O75019] [ENST00000251375]	5,178	8,406	-3,23
MGST1	Homo sapiens microsomal glutathione S-transferase 1 (MGST1), transcript variant 1c, mRNA [NM_145791]	5,992	9,293	-3,30
CD37	Homo sapiens CD37 molecule (CD37), transcript variant 1, mRNA [NM_001774]	7,019	10,328	-3,31
NCF1	Homo sapiens neutrophil cytosolic factor 1, (chronic granulomatous disease, autosomal 1) (NCF1), mRNA [NM_000265]	7,417	10,756	-3,34
CCNB2	Homo sapiens cyclin B2 (CCNB2), mRNA [NM_004701]	9,27	12,634	-3,36
KRT23	Homo sapiens keratin 23 (histone deacetylase inducible) (KRT23), mRNA [NM_015515]	4,473	7,868	-3,39
BCL2A1	Homo sapiens BCL2-related protein A1 (BCL2A1), mRNA [NM_004049]	6,103	9,504	-3,40
CD19	Homo sapiens CD19 molecule (CD19), mRNA [NM_001770]	7,129	10,56	-3,43
C11orf21	Homo sapiens C11orf21 mRNA, complete cds. [AB029488]	6,512	9,946	-3,43
IL18RAP	Homo sapiens interleukin 18 receptor accessory protein (IL18RAP), mRNA [NM_003853]	6,582	10,019	-3,44
NFE2	Homo sapiens nuclear factor (erythroid-derived 2), 45kDa (NFE2), mRNA [NM_006163]	7,16	10,615	-3,46
P2RY2	Homo sapiens purinergic receptor P2Y, G-protein coupled, 2 (P2RY2), transcript variant 1, mRNA [NM_176072]	5,313	8,771	-3,46
STK17B	Homo sapiens serine/threonine kinase 17b (apoptosis-inducing) (STK17B), mRNA [NM_004226]	7,062	10,527	-3,46
ALOX5AP	Homo sapiens arachidonate 5-lipoxygenase-activating protein (ALOX5AP), mRNA [NM_001629]	6,483	9,95	-3,47
MS4A1	Homo sapiens membrane-spanning 4-domains, subfamily A, member 1 (MS4A1), transcript variant 1, mRNA [NM_152866]	4,793	8,268	-3,47
SLC16A3	Homo sapiens pp10472 mRNA, complete cds. [AF318321]	5,423	8,912	-3,49
THC2541839	chr7:105693173-105693232	4,943	8,467	-3,52
BIRC5	Homo sapiens baculoviral IAP repeat-containing 5 (survivin) (BIRC5), transcript variant 3, mRNA [NM_001012271]	10,845	14,37	-3,53
APOBEC3B	Homo sapiens apolipoprotein B mRNA editing enzyme, catalytic polypeptide-like 3B (APOBEC3B), mRNA [NM_004900]	5,723	9,258	-3,54
LILRA2	Homo sapiens leukocyte immunoglobulin-like receptor, subfamily A (with TM domain), member 2 (LILRA2), mRNA [NM_006866]	4,673	8,243	-3,57
SIGLEC5	Homo sapiens sialic acid binding Ig-like lectin 5 (SIGLEC5), mRNA [NM_003830]	6,286	9,855	-3,57
FPR1	Homo sapiens formyl peptide receptor 1 (FPR1), mRNA [NM_002029]	7,567	11,147	-3,58
AV756170	AV756170 BM Homo sapiens cDNA clone BMFBGA09 5', mRNA sequence [AV756170]	5,675	9,281	-3,61
GPR109B	Homo sapiens G protein-coupled receptor 109B (GPR109B), mRNA [NM_006018]	5,529	9,142	-3,61
IL1B	Homo sapiens interleukin 1, beta (IL1B), mRNA [NM_000576]	6,976	10,638	-3,66
FAM64A	Homo sapiens family with sequence similarity 64, member A (FAM64A), mRNA [NM_019013]	8,795	12,474	-3,68

Supplemental Table 1
Morandi et al

SERPINB2	Plasminogen activator inhibitor 2 precursor (PAI-2) (Placental plasminogen activator inhibitor) (Monocyte Arg-serpin) (Urokinase inhibitor). [Source:Uniprot/SWISSPROT;Acc:P05120] [ENST00000299502]	5,906	9,593	-3,69
CSTA	Homo sapiens cystatin A (stefin A) (CSTA), mRNA [NM_005213]	7,933	11,621	-3,69
VNN2	Homo sapiens vanin 2 (VNN2), transcript variant 1, mRNA [NM_004665]	7,085	10,783	-3,70
PPBP	Homo sapiens pro-platelet basic protein (chemokine (C-X-C motif) ligand 7) (PPBP), mRNA [NM_002704]	5,227	8,942	-3,72
BLK	Homo sapiens B lymphoid tyrosine kinase (BLK), mRNA [NM_001715]	4,938	8,713	-3,78
PRG3	Homo sapiens proteoglycan 3 (PRG3), mRNA [NM_006093]	4,871	8,725	-3,85
HSH2D	Homo sapiens hematopoietic SH2 domain containing (HSH2D), mRNA [NM_032855]	6,144	10,097	-3,95
FCN2	Homo sapiens ficolin (collagen/fibrinogen domain containing lectin) 2 (hucolin) (FCN2), transcript variant SV0, mRNA [NM_004108]	4,439	8,72	-4,28
THC2530077	ALU5_HUMAN (P39192) Alu subfamily SC sequence contamination warning entry, partial (9%) [THC2530077]	7,963	12,314	-4,35
LYZ	Homo sapiens lysozyme (renal amyloidosis) (LYZ), mRNA [NM_000239]	7,447	11,831	-4,39
LILRA3	Homo sapiens leukocyte immunoglobulin-like receptor, subfamily A (without TM domain), member 3 (LILRA3), mRNA [NM_006865]	5,042	9,438	-4,40
CTSG	Homo sapiens cathepsin G (CTSG), mRNA [NM_001911]	6,832	11,328	-4,50
RETN	Homo sapiens resistin (RETN), mRNA [NM_020415]	5,149	9,788	-4,64
RGR	Homo sapiens Ral-GDS related protein Rgr (Rgr), mRNA [NM_153615]	7,114	11,823	-4,71
MNDA	Homo sapiens myeloid cell nuclear differentiation antigen (MNDA), mRNA [NM_002432]	7,355	12,112	-4,76
DEFA4	Homo sapiens defensin, alpha 4, corticostatin (DEFA4), mRNA [NM_001925]	4,678	9,672	-4,99
LTF	Homo sapiens lactotransferrin (LTF), mRNA [NM_002343]	5,41	10,445	-5,04
THC2662610	ALU1_HUMAN (P39188) Alu subfamily J sequence contamination warning entry, partial (5%) [THC2662610]	4,464	9,511	-5,05
FCN1	Homo sapiens ficolin (collagen/fibrinogen domain containing) 1 (FCN1), mRNA [NM_002003]	6,802	11,884	-5,08
AQP9	Homo sapiens aquaporin 9 (AQP9), mRNA [NM_020980]	5,155	10,584	-5,43
MPO	Homo sapiens myeloperoxidase (MPO), nuclear gene encoding mitochondrial protein, mRNA [NM_000250]	4,959	10,633	-5,67
S100P	Homo sapiens S100 calcium binding protein P (S100P), mRNA [NM_005980]	6,634	12,385	-5,75
CAMP	Homo sapiens cathelicidin antimicrobial peptide (CAMP), mRNA [NM_004345]	5,554	11,337	-5,78
S100A8	Homo sapiens S100 calcium binding protein A8 (S100A8), mRNA [NM_002964]	8,664	14,969	-6,31
DEFA3	Homo sapiens defensin, alpha 3, neutrophil-specific (DEFA3), mRNA [NM_005217]	7,002	13,722	-6,72
S100A9	Homo sapiens S100 calcium binding protein A9 (S100A9), mRNA [NM_002965]	5,866	12,929	-7,06
