# Supplementary figures and images for: Bone Marrow-Infiltrating Human Neuroblastoma Cells Express High Levels of Calprotectin and HLA-G Proteins
Source: PLoS One. 2012 Jan 9;7(1):e29922. doi: 10.1371/journal.pone.0029922 (PMC3253802; doi:10.1371/journal.pone.0029922)

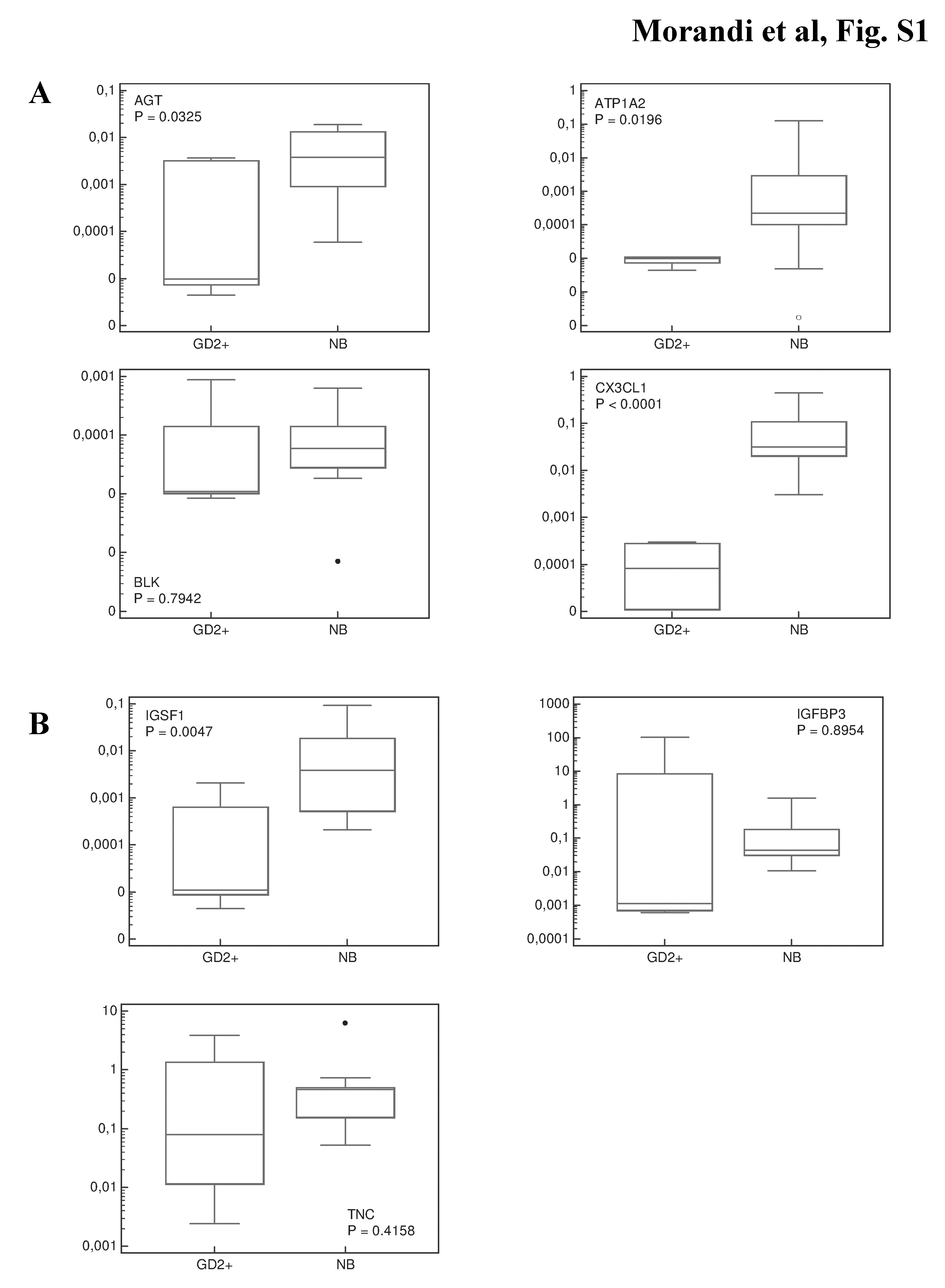

Supplement: Figure S1 — Box-and-Whisker plots of AGT, ATP1A2, BLK, CX3CL1, IGSF1, IGFBP3, and TNC gene expression values by qPCR (normalized to ATP5B, and after logarithmic transformation of original measures) in 5 GD2 positive cells (GD2+) and 5 primary tumors (NB) previously hybridized to microarray. Each box represents the values from the 25th to 75th percentile, the middle line represents the median, and a line extends from the minimum to the maximum value, excluding outliers which are displayed as blue dots. (TIF) [file pone.0029922.s003.tif]

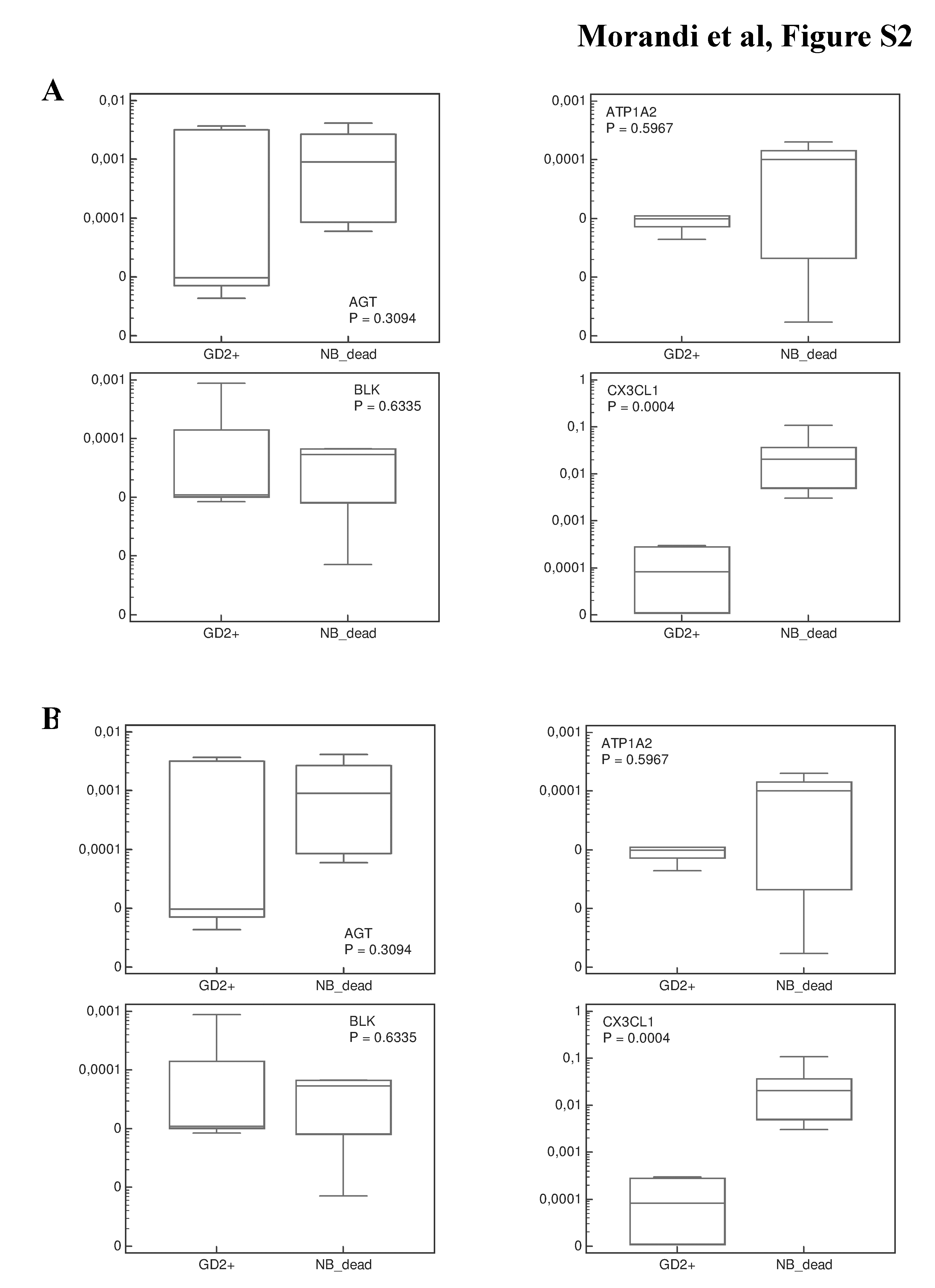

Supplement: Figure S2 — Box-and-Whisker plots of AGT, ATP1A2, BLK, CX3CL1, IGSF1, IGFBP3, and TNC gene expression values by qPCR (normalized to ATP5B, and after logarithmic transformation of original measures) in 5 GD2 positive cells (GD2+) and 5 primary tumors from patients dead of disease (NB_dead), previously hybridized to microarrays. Each box represents the values from the 25th to 75th percentile, the middle line represents the median, and a line extends from the minimum to the maximum value. (TIF) [file pone.0029922.s004.tif]

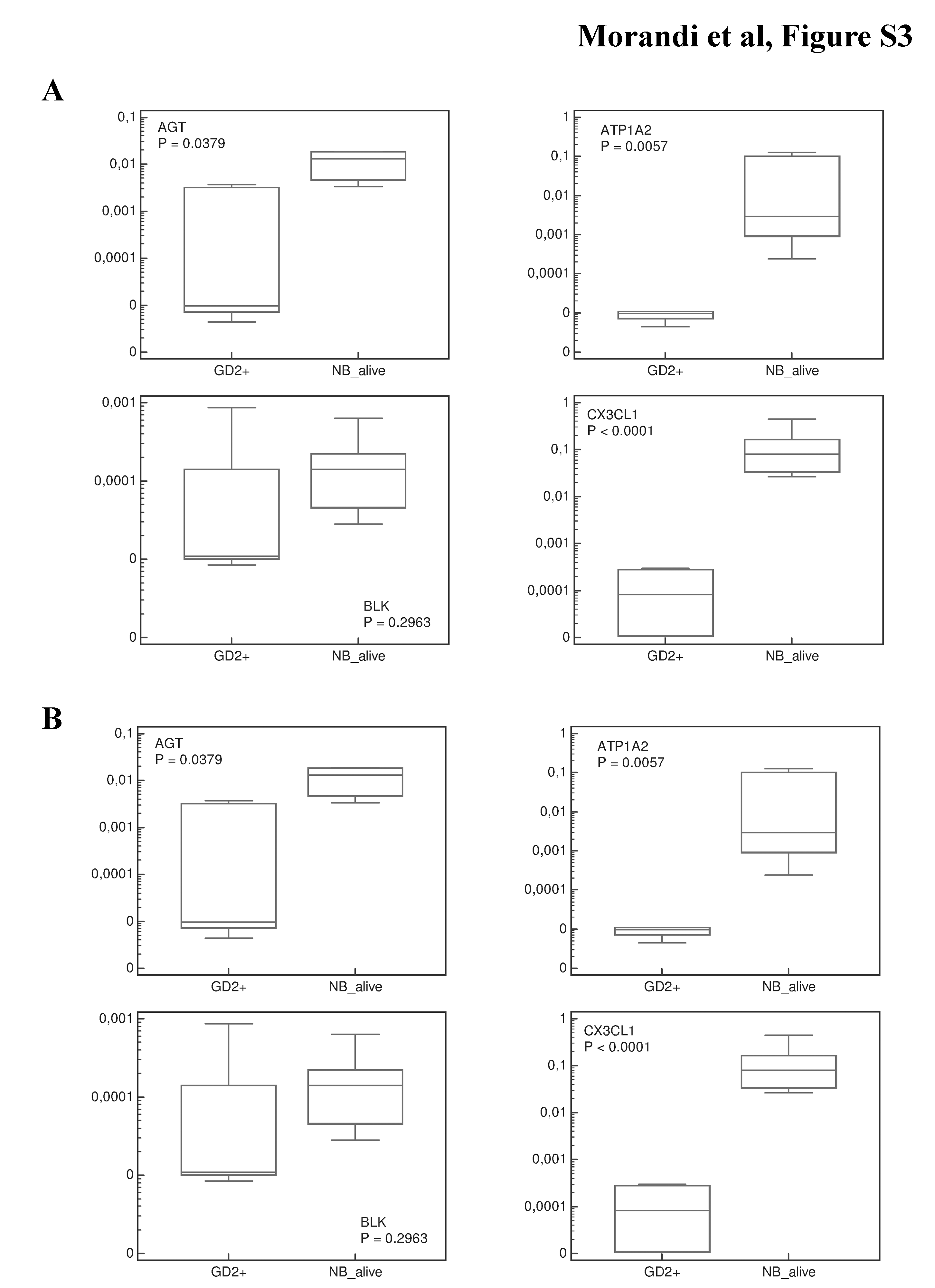

Supplement: Figure S3 — Box-and-Whisker plots of AGT, ATP1A2, BLK, CX3CL1, IGSF1, IGFBP3, and TNC gene expression values by qPCR (normalized to ATP5B, and after logarithmic transformation of original measures) in 5 GD2 positive cells (GD2+) and 5 primary tumors from alive patients (NB_alive), previously hybridized to microarrays. Each box represents the values from the 25th to 75th percentile, the middle line represents the median, and a line extends from the minimum to the maximum value, excluding outliers which are displayed as blue dots. (TIF) [file pone.0029922.s005.tif]

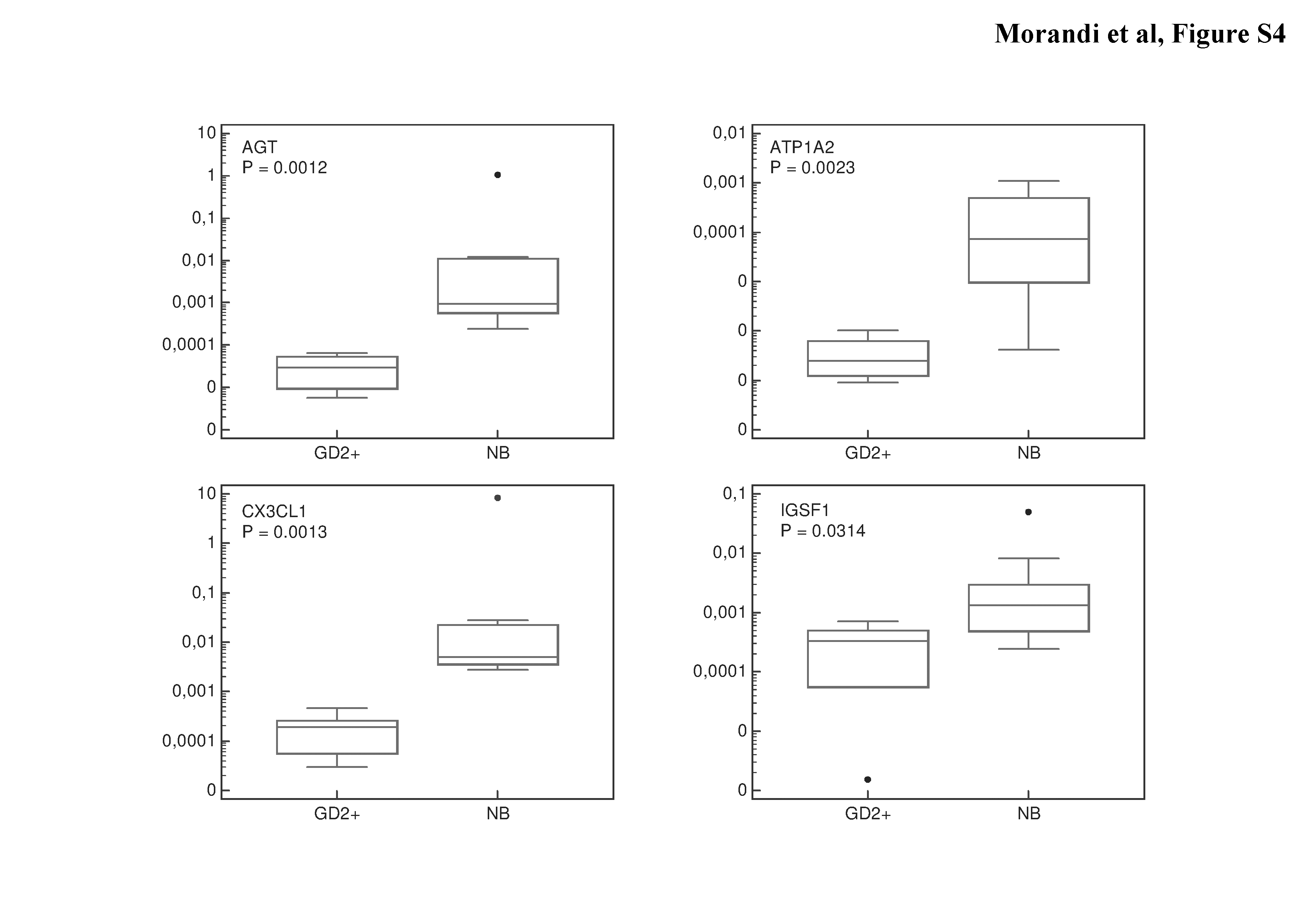

Supplement: Figure S4 — Box-and-Whisker plots of AGT, ATP1A2, CX3CL1, and IGSF1 gene expression values by qPCR (normalized to ATP5B, and after logarithmic transformation of original measures) in 5 GD2 positive cells (GD2+) and 5 primary tumors from stage 4 patients (NB) (independent sample set). Each box represents the values from the 25th to 75th percentile, the middle line represents the median, and a line extends from the minimum to the maximum value, excluding outliers which are displayed as blue dots. (TIF) [file pone.0029922.s006.tif]

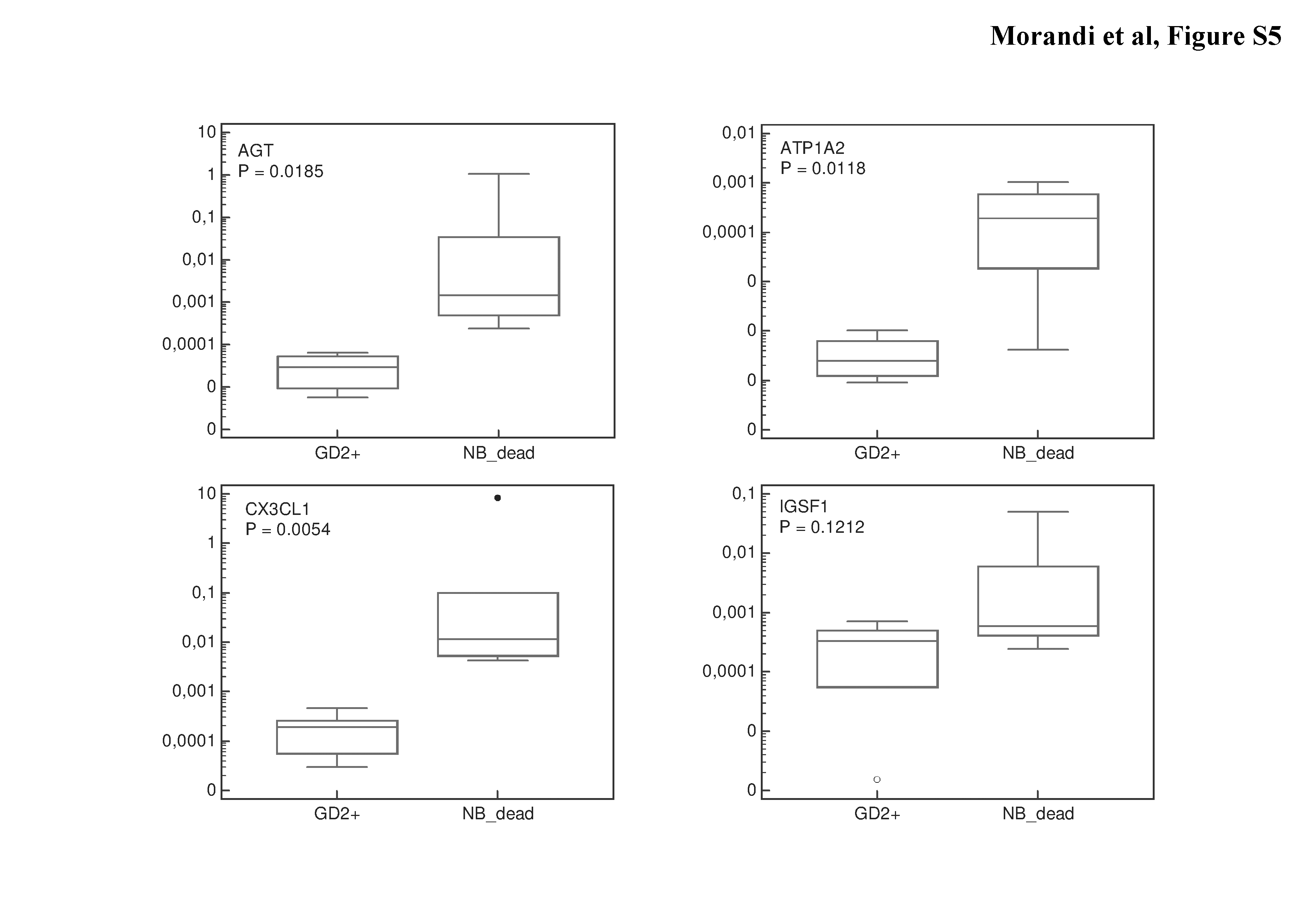

Supplement: Figure S5 — Box-and-Whisker plots of AGT, ATP1A2, CX3CL1, and IGSF1 gene expression values by qPCR (normalized to ATP5B, and after logarithmic transformation of original measures) in 5 GD2 positive cells (GD2+) and 5 primary tumors from stage 4 patients dead of disease (NB_dead) (independent sample set). Each box represents the values from the 25th to 75th percentile, the middle line represents the median, and a line extends from the minimum to the maximum value, excluding outliers which are displayed as blue dots. (TIF) [file pone.0029922.s007.tif]

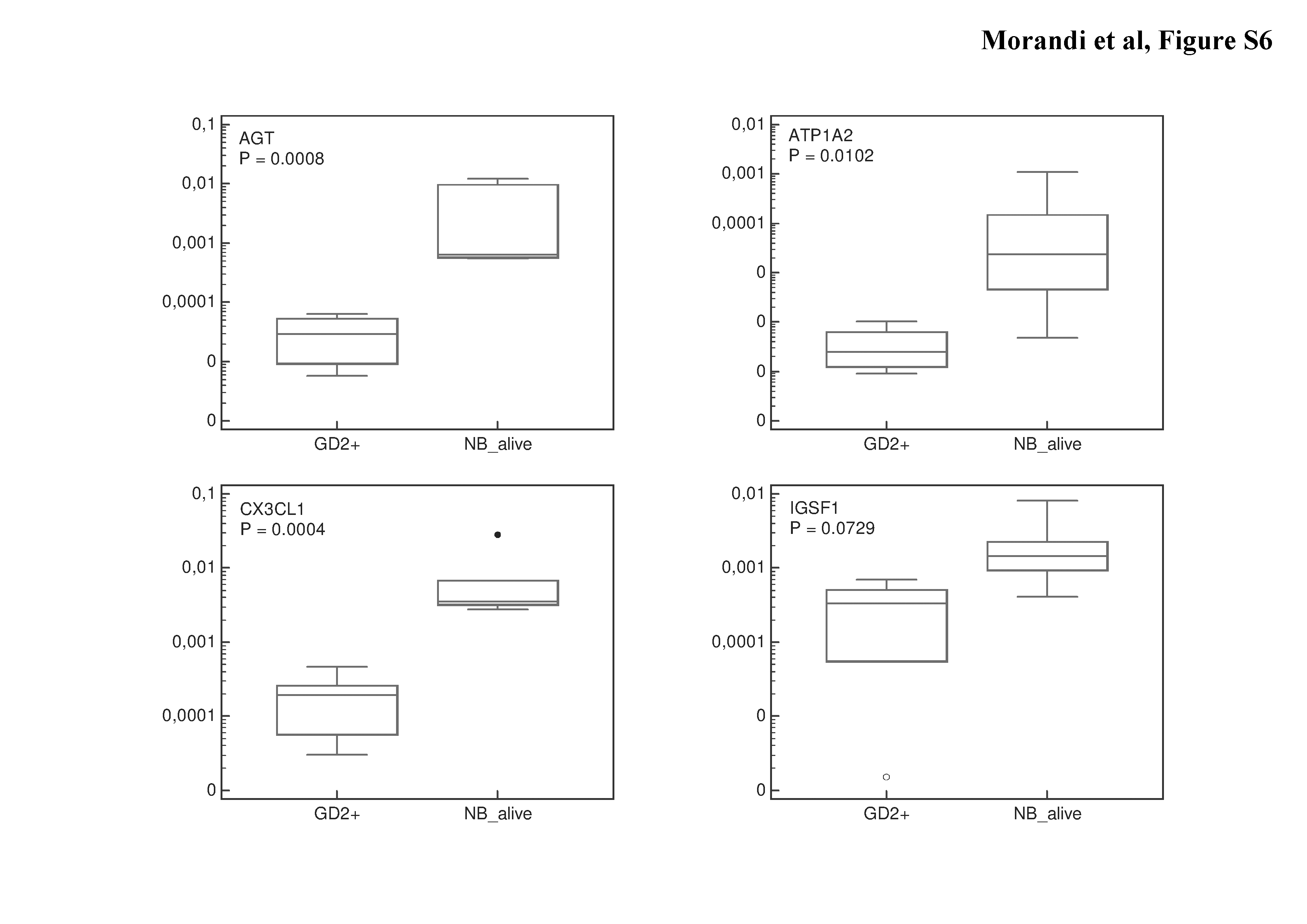

Supplement: Figure S6 — Box-and-Whisker plots of AGT, ATP1A2, CX3CL1, and IGSF1 gene expression values by qPCR (normalized to ATP5B, and after logarithmic transformation of original measures) in 5 GD2 positive cells (GD2+) and 5 primary tumors from alive stage 4 patients (NB_alive) (independent sample set). Each box represents the values from the 25th to 75th percentile, the middle line represents the median, and a line extends from the minimum to the maximum value, excluding outliers which are displayed as blue dots. (TIF) [file pone.0029922.s008.tif]
